# Development of a Method for Clinical Evaluation of Artificial Intelligence–Based Digital Wound Assessment Tools

**DOI:** 10.1001/jamanetworkopen.2021.7234

**Published:** 2021-05-19

**Authors:** Raelina S. Howell, Helen H. Liu, Aziz A. Khan, Jon S. Woods, Lawrence J. Lin, Mayur Saxena, Harshit Saxena, Michael Castellano, Patrizio Petrone, Eric Slone, Ernest S. Chiu, Brian M. Gillette, Scott A. Gorenstein

**Affiliations:** 1 Department of Surgery, NYU Langone Hospital Long Island, Mineola, New York; 2 Department of Surgery, NYU Long Island School of Medicine, Mineola, New York; 3 Department of Foundations of Medicine, NYU Long Island School of Medicine, Mineola, New York; 4 NYU Kimmel Hyperbaric and Advanced Wound Healing Center, New York, New York; 5 Droice Labs, New York, New York

## Abstract

**Importance:**

Accurate assessment of wound area and percentage of granulation tissue (PGT) are important for optimizing wound care and healing outcomes. Artificial intelligence (AI)–based wound assessment tools have the potential to improve the accuracy and consistency of wound area and PGT measurement, while improving efficiency of wound care workflows.

**Objective:**

To develop a quantitative and qualitative method to evaluate AI-based wound assessment tools compared with expert human assessments.

**Design, Setting, and Participants:**

This diagnostic study was performed across 2 independent wound centers using deidentified wound photographs collected for routine care (site 1, 110 photographs taken between May 1 and 31, 2018; site 2, 89 photographs taken between January 1 and December 31, 2019). Digital wound photographs of patients were selected chronologically from the electronic medical records from the general population of patients visiting the wound centers. For inclusion in the study, the complete wound edge and a ruler were required to be visible; circumferential ulcers were specifically excluded. Four wound specialists (2 per site) and an AI-based wound assessment service independently traced wound area and granulation tissue.

**Main Outcomes and Measures:**

The quantitative performance of AI tracings was evaluated by statistically comparing error measure distributions between test AI traces and reference human traces (AI vs human) with error distributions between independent traces by 2 humans (human vs human). Quantitative outcomes included statistically significant differences in error measures of false-negative area (FNA), false-positive area (FPA), and absolute relative error (ARE) between AI vs human and human vs human comparisons of wound area and granulation tissue tracings. Six masked attending physician reviewers (3 per site) viewed randomized area tracings for AI and human annotators and qualitatively assessed them. Qualitative outcomes included statistically significant difference in the absolute difference between AI-based PGT measurements and mean reviewer visual PGT estimates compared with PGT estimate variability measures (ie, range, standard deviation) across reviewers.

**Results:**

A total of 199 photographs were selected for the study across both sites; mean (SD) patient age was 64 (18) years (range, 17-95 years) and 127 (63.8%) were women. The comparisons of AI vs human with human vs human for FPA and ARE were not statistically significant. AI vs human FNA was slightly elevated compared with human vs human FNA (median [IQR], 7.7% [2.7%-21.2%] vs 5.7% [1.6%-14.9%]; *P* < .001), indicating that AI traces tended to slightly underestimate the human reference wound boundaries compared with human test traces. Two of 6 reviewers had a statistically higher frequency in agreement that human tracings met the standard area definition, but overall agreement was moderate (352 yes responses of 583 total responses [60.4%] for AI and 793 yes responses of 1166 total responses [68.0%] for human tracings). AI PGT measurements fell in the typical range of variation in interreviewer visual PGT estimates; however, visual PGT estimates varied considerably (mean range, 34.8%; mean SD, 19.6%).

**Conclusions and Relevance:**

This study provides a framework for evaluating AI-based digital wound assessment tools that can be extended to automated measurements of other wound features or adapted to evaluate other AI-based digital image diagnostic tools. As AI-based wound assessment tools become more common across wound care settings, it will be important to rigorously validate their performance in helping clinicians obtain accurate wound assessments to guide clinical care.

## Key Points

**Question:**

How does an artificial intelligence (AI)–based wound assessment algorithm compare with expert human annotations of wound area and granulation tissue?

**Findings:**

This diagnostic study of 199 photographs of wounds developed a method to quantitatively and qualitatively evaluate AI wound annotations. Error measure distributions comparing AI with human tracings were generally statistically similar to those comparing 2 independent humans, suggesting similar tracing performance.

**Meaning:**

These findings suggest that AI-based wound annotation algorithms can perform similarly to human wound specialists; however, the degree of agreement regarding wound features among expert physicians can vary substantially, presenting challenges for defining a criterion standard.

## Teaser

This diagnostic study develops a quantitative and qualitative method to evaluate artificial intelligence–based wound assessment tools compared with expert human assessments.

## Introduction

Chronic wounds cause significant morbidity and mortality and cost the US health care system approximately $25 billion annually.^1^ Patients with chronic wounds require frequent visits for management by an interprofessional team. The primary indicator of healing is a decrease in wound surface area, which helps clinicians determine healing progress and choose appropriate therapy.^2,3^ Accurate measurements of wound area are thus critical for optimizing outcomes for patients with chronic wounds.

While numerous methods can be used to quantify wound area, many clinics still use manual ruler-based measurements, which are subject to high variability and can overestimate the true surface area by as much as 40%.^4,5,6,7^ Another common method of wound measurement is contact acetate tracing, but the contact on a patient’s wound can alter the contour of the border, introduce a source of contamination to patients with increased risk of infection, and induce pain.^8,9^ Manual digital planimetry of wound photographs improves accuracy but is still subject to interclinician variability and can be too time consuming to integrate into a high-volume wound care center.^7,10,11^

In addition to wound area, the percentage of healthy granulation tissue in the wound bed is important for determining whether a wound is likely to heal or is ready for definitive closure by skin graft or flap. Clinicians estimate the percentage of granulation tissue (PGT) visually based on color as an indicator of healing.^12,13,14^ Exuberant dark red granulation could indicate infection, while pale granulation tissue can indicate poor angiogenesis and blood supply in the wound bed.^15^ However, visual PGT estimation is imprecise and subject to high interclinician variability. Algorithms that accurately quantify granulation tissue could improve wound treatment decisions; for example, recent work has demonstrated that color image analysis of granulation tissue can predict healing outcomes for pressure ulcers.^16,17^

There is currently no criterion standard wound assessment method. However, promising strides in the field of artificial intelligence (AI) are enabling automated analysis of diagnostic images.^18^ Advances in wound imaging devices and software, such as the Silhouette (Aranz Medical) and inSight (Ekare), are helping clinicians measure wounds more quickly, accurately, and reproducibly, leading to better clinical decisions and patient outcomes.^19,20^ Such tools will require rigorous validation to ensure equivalent performance with standard measurement methods, but there is currently no accepted methodological framework for clinical evaluation of AI-based digital wound assessment tools.

In this article, we developed a method to evaluate the performance of AI-based software for wound assessment against manual wound assessments performed by wound care clinicians. We quantitatively assessed AI performance in wound area and granulation tissue tracings by statistically comparing error measure distributions between a human reference trace and the AI trace with error measure distributions between 2 human traces. Because wound assessment is subjective by nature, we also developed a qualitative approach to assessing AI performance through masked review of AI and human tracings by expert wound care attending physicians.

## Methods

### Selection of Digital Wound Images and Associated Data

This diagnostic study was an institutional review board–approved retrospective medical record review performed at 2 independently operated tertiary-care hospital wound care centers (referred to as site 1 and site 2) within a large academic medical center system. The study was determined to meet institutional review board exemption criteria, as there was no direct patient contact and no identifying patient information included in the data. This article is reported according to Standards for Reporting of Diagnostic Accuracy (STARD) reporting guideline. A total of 199 wound photographs from 199 patients were selected across both sites. The study was conducted independently at each site using photographs only from that site. Photographs had been taken for routine clinical care by several different wound center clinicians using various devices. For a photograph to be included in the study, the complete wound edge and a ruler must have been visible. Any identifying data present in the wound image were removed prior to analysis. Deidentified age, sex, and wound type data were also retrieved. Patient demographic characteristics and wound type distribution are summarized in Table 1.

**Table 1.** Patient Demographic Characteristics

Characteristic	No. (%) (N = 199)
Women	127 (63.8)
Men	72 (36.2)
Age, mean (SD) [range], y	64 (18) [17-95]
Wound types	
Venous leg ulcer	47 (23.6)
Pressure ulcer	41 (20.6)
Surgical wound	32 (16.1)
Trauma wound	25 (12.5)
Diabetic foot ulcer	21 (10.5)
Arterial ulcer	7 (3.5)
Abscess	5 (2.5)
Lymphedema	3 (1.5)
Radiation	3 (1.5)
Burn	2 (1.0)
Other	13 (6.5)

### Definition of Wound Area and Granulation Tissue

Wound area is commonly defined as any nonepithelized skin area; however, because patients can have multiple adjacent nonepithelialized satellite wound areas or multiple large wounds within a single photograph, a standardized definition was required to determine which wound areas should be considered a single wound for tracing. For this study, we defined the wound area for tracing as the largest nonepithelialized wound area and any satellite lesions within 2 cm of the nonepithelialized edge. Granulation tissue area was defined as any apparent red granular areas within the wound area.

### Wound Area and Granulation Tissue Tracing

**Figure 1.**
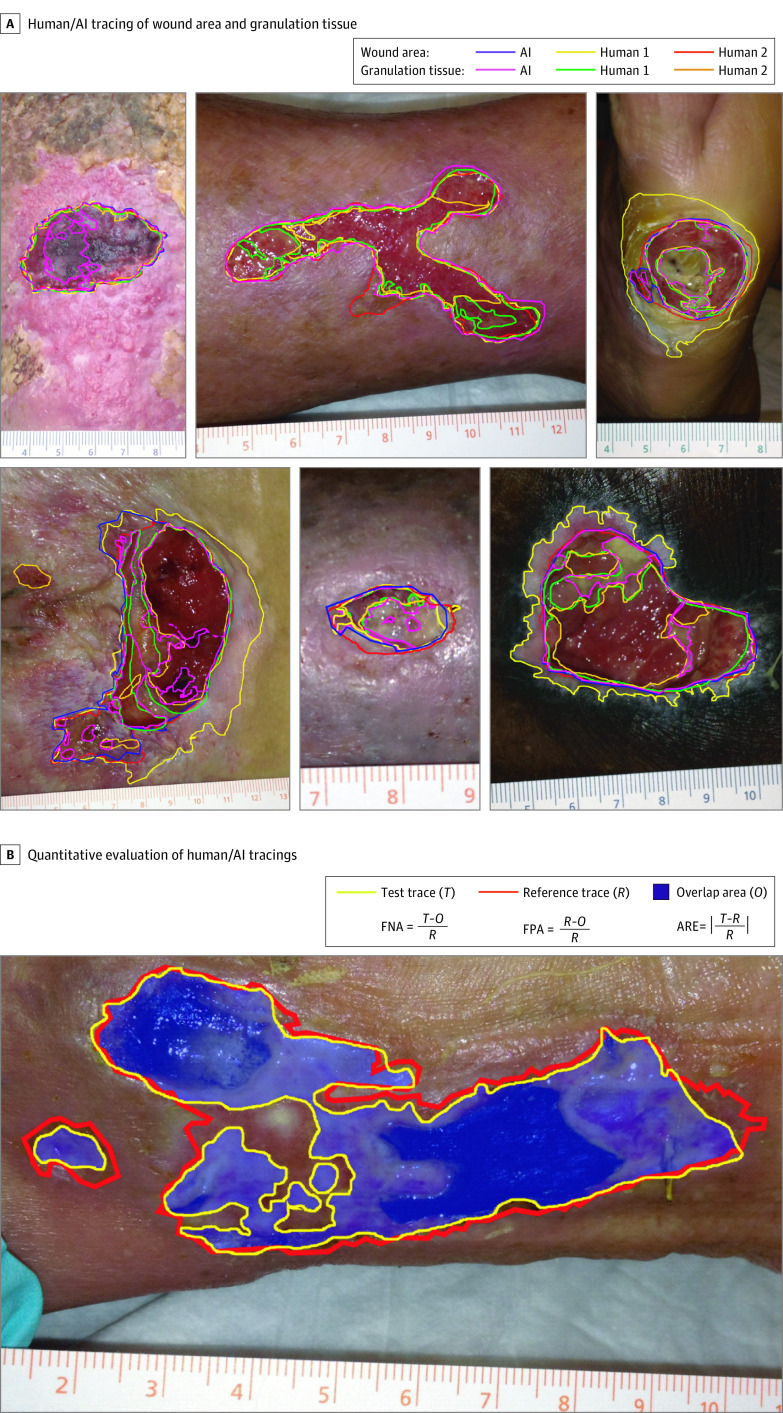
Overview of Methodology for Evaluation of Artificial Intelligence (AI)–Based Digital Wound Assessment Tools A, Example images of wound area and granulation tissue tracings by humans and AI for wounds of diverse types, shapes, and sizes. B, Illustration of quantitative method for comparing wound area and granulation tissue tracings between humans and between humans and AI. One human tracing was selected as a reference trace (*R*), a second tracing (other human or AI) was selected as the test trace (*T*), and the overlapping area (*O*) was determined. The error measures (false-negative area [FNA], false positive area [FPA], and absolute relative error [ARE]) between the reference and test tracings were then calculated.

Four physician wound care specialists (2 at each site, referred to as human 1 [H1] and human 2 [H2] at each site) independently performed manual tracings using the freehand tool in ImageJ software (National Institutes of Health) following a standardized protocol.^21,22^ The tracing regions of interest (ROI) were exported as ImageJ ROI files for quantitative comparisons between traces. Human tracers recorded the time taken for each wound area tracing and then separately traced granulation tissue for a subset of 25 photographs at site 1 and 22 photographs at site 2. For AI-based measurements, digital images were uploaded to Droice Labs wound analytics service (Droice Labs). The algorithm uses a boundary detection algorithm applied to a coarse ROI drawn around the wound given as input. AI software–based traces were exported as ImageJ ROI files for quantitative comparison with human traces using ImageJ. Example wound photographs, annotated with area and granulation tissue tracings, by H1, H2, and AI are shown in Figure 1A.

### Quantitative Analysis of Wound Tracings

To evaluate the quantitative performance of AI-based wound area and granulation tissue tracings, we statistically compared error distributions between a test AI trace and a reference human trace (AI vs human) with the error distributions between 2 independent human tracings (human vs human) (Figure 1B). For each photograph, each of the human tracings served as both reference and test, resulting in 4 test vs reference comparisons, ie, AI vs H1, AI vs H2, H1 vs H2, and H2 vs H1. To quantify error measures, ROIs for each image were imported into ImageJ and the AND command was used to create a new ROI for the overlapping regions within the test and reference traces. Areas of the test (*T*), reference (*R*), and overlap (*O*) ROIs were measured in pixels, and 3 separate error metrics were used to quantitatively compare the pairs of tracings.

The false-negative area (FNA) was defined as the area in the reference trace that was not part of the area in the test trace, normalized by reference trace total area:
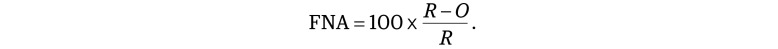
The false-positive area (FPA) was defined as the area in the test trace that was not part of the area in the reference trace, normalized by reference trace total area: 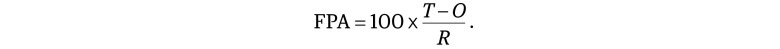
The relative error (RE) was the absolute difference in trace area, normalized by the reference trace total area:
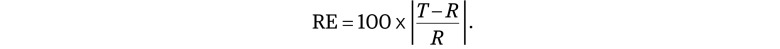
We chose the 3 error measures to quantify different aspects of tracing differences. Absolute RE (ARE) quantifies the overall relative difference in the area measurements but does not reflect differences in the location of wound boundaries (eg, ARE can still be small or zero if annotators traced completely separate areas that happened to be the same size). FNA and FPA compare the locations of traces by quantifying the degree of overlap and underlap of the test trace compared with the reference trace. Therefore, they provide additional insight into the nature of errors compared with the Jaccard index and Dice coefficient, which consolidate both types of errors.^23,24^

When a tracer (AI or human) determined the wound was completely epithelialized,^25^ there was no trace for that tracer in a given photograph. If 2 tracings were available for a photograph (only 1 tracer determined complete epithelization or lack of granulation tissue), the comparison between the other 2 traces was included in the data. If 2 or all 3 tracers determined complete epithelialization, then no comparisons could be made for that photograph.

### Qualitative Analysis of Wound Tracings

Three independent attending wound care clinicians at each site performed a masked qualitative review of photographs and tracings. To qualitatively assess AI-based PGT measurements against expert reviewer visual PGT estimates, reviewers first viewed the original untraced photographand were asked to visually estimate PGT to the nearest 10% to provide a standard reference for comparison. To compare AI PGT measurements to expert visual estimates, we calculated the absolute difference between the AI PGT measurement and the mean of the 3 visual PGT estimates for each photograph and then assessed whether the distribution of differences was similar to the distribution of interreviewer variability measures by paired *t* tests (described in the Statistical Analysis section).

To qualitatively assess performance of AI-based wound area tracings vs human tracings, reviewers then viewed the 3 wound area tracings in randomized order and answered 3 survey questions. To assess overall quality of the tracings, reviewers were asked whether they agreed the tracing met the standardized definition of wound area (question 1). To examine whether there were differences in perception of tracing quality across annotators, reviewers were asked which tracing they thought was most accurate (question 2). To examine whether there was a perceptible difference in appearance of the tracings between AI and humans, reviewers were asked which tracing they thought was performed by AI (question 3).

### Statistical Analysis

For quantitative comparisons of wound area and granulation tissue tracings, Wilcoxon matched pairs signed rank tests (2-tailed, α = .05) were performed to determine whether distributions of error measures (FPA, FNA, ARE) between AI vs human and human vs human comparisons for wound and granulation area were statistically different (*P* < .05). For masked reviewer responses to question 1, the Fisher exact test was performed to determine whether there was a statistically different frequency in reviewer agreement that the tracing met the wound area definition between AI and human tracings. For masked reviewer responses to questions 2 and 3, χ^2^ tests were performed to determine statistically significant bias in frequency of annotator selection vs random selection (*P* < .05). For qualitative assessment of PGT measurements, *t* tests (paired, 2-tailed, α = .05) were performed to determine whether the absolute difference between AI PGT measurement and mean reviewer PGT estimates were significantly different from interreviewer variability measures, expressed as mean range and mean SD. Statistical tests were performed in GraphPad Prism 6 (GraphPad Software, San Diego, CA).

## Results

### Patient Demographic Characteristics and Wound Tracings

A total of 199 photographs from 199 patients were included. The mean patient (SD) age was 64 (18) years (range, 17- 95 years), and 172 patients (63.8%) were women. The eligible wound photographs represented a wide spectrum of wound types, with venous leg ulcers (VLU), pressure ulcers (PU), surgical wounds, traumatic wounds, and diabetic foot ulcers (DFU) making up the majority (166 [83%]) (Table 1).

Manual tracing of the wound area took an mean of 105 seconds (SD, 73 seconds; range, 10-524 seconds) across all 4 wound care specialists. Representative wound area and granulation tissue tracings by AI, H1, and H2 are shown in Figure 1A, and the complete set of photographs and tracing for both sites are available in eAppendix 1 and eAppendix 2 in the Supplement. For several wound photographs, 1 or more tracers did not detect any nonepithelized wound area (photographs 17, 28, 34, 43, 47, and 74 at site 1) (eAppendix 1 in the Supplement), and there was only 1 photograph that all annotators agreed was healed (photograph 34 at site 1) (eAppendix 1 in the Supplement). In 1 photograph (photograph 91 at site 1) (eAppendix 1 in the Supplement), completely nonoverlapping traces were observed between H1 and the H2/AI tracings where multiple satellite wounds were present.

### Quantitative Evaluation of Wound Area and Granulation Tissue Measurements

**Figure 2.**
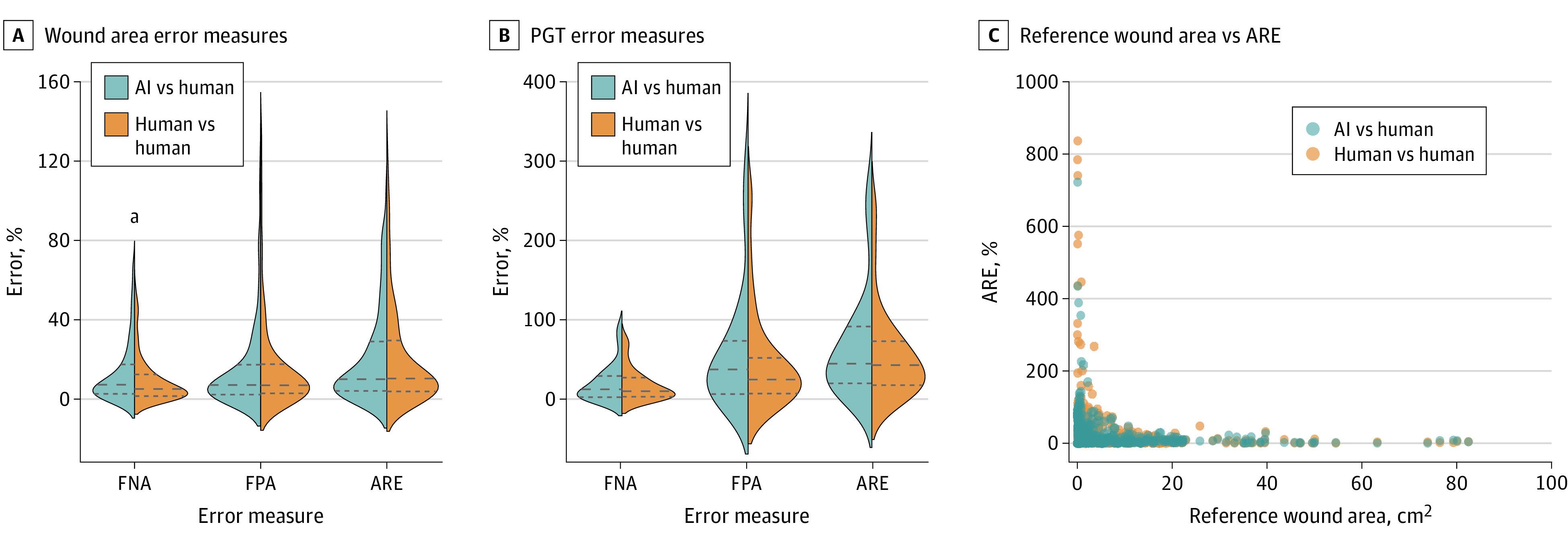
Quantitative Evaluation of Human and Artificial Intelligence (AI) Wound Area and Percent Granulation Tissue (PGT) Measurements A and B, Violin plots showing distributions of wound area (A) and PGT (B) error measures of false-negative area (FNA), false-positive area (FPA), and absolute relative error (ARE) for AI vs human and human vs human comparisons. Dashed lines indicate the median and quartiles of the error measure distributions. Outliers above the 98th percentile are not shown to aid visualization of the distributions but were included in the statistical analysis. C, Scatter plot showing ARE vs the reference wound area for AI vs human and human vs human comparisons. ^a^*P* < .05.

Figure 2 shows violin plots visualizing the error measure distributions for AI vs human and human vs human comparisons for tracings of wound area (Figure 2A) and granulation tissue (Figure 2B). Wound area FPA and ARE were not statistically significant between comparisons (median [interquartile range {IQR}] FPA, 7.8% [2.3%-23.2%] vs 7.7% [3.1%-20.1%]; *P* = .84; ARE, 11.5% [4.4%-33.5%] vs 11.2% [4.2%-35.9%]; *P* = .47). There was a statistically significant slight elevation in FNA for AI vs human comparisons compared with human vs human comparisons (median (IQR) FNA, 7.7% [2.7%-21.2%] vs 5.7% [1.6%-14.9%]; *P* < .001). None of the granulation tissue error measure comparisons were statistically significant comparing AI vs human with human vs human (median [IQR] FNA, 14.4% [2.4%-33.4%] vs 10.7% [3.2%-28.8%]; *P* = .44; FPA, 40.9% [7.5%-94.6%] vs 27.3% [7.5%-68.0%]; *P* = .10; ARE, 47.7% [20.2%-94.8%] vs 46.3% [18.3%-77.7%]; *P* = .58). Smaller wounds tended to have much higher relative error between tracings compared with large wounds for both comparisons (Figure 2C).

### Qualitative Evaluation of Wound Area Tracings

Masked reviewer survey responses are summarized in Table 2. Two of 6 reviewers had a statistically higher frequency in agreement that human tracings met the standard area definition, but overall agreement was moderate (352 yes responses of 583 total responses [60.4%] for AI and 793 yes responses of 1166 total responses [68.0%] for human tracings). At site 1, reviewers found that a significant fraction of the wound area tracings did not meet the definition of wound area (42 of 100 [42.0%] to 82 of 110 [74.5%] of traces met the definition), while at site 2, reviewer 2 found most area tracings agreed with the definition (78 of 85 [91.8%] to 82 of 85 [96.5%]) and reviewers 1 and 3 found more moderate agreement (reviewer 1, 65 of 89 [73.0%] to 67 of 89 [75.3%]; reviewer 3, 47 of 89 [52.8%] to 63 of 89 [70.8%]). Two of the 6 reviewers (reviewer 1 at site 1, reviewer 3 at site 2) had a statistically significant lower rate of agreement that AI tracings met the definition vs human tracings, and there were no significant differences for the other 4 reviewers.

**Table 2.** Masked Reviewer Survey Responses for the Qualitative Evaluation of Digital Wound Assessments

Question	Annotator	No./total No. (%)					
		Site 1			Site 2		
		R1	R2	R3	R1	R2	R3
1. Area tracing meets definition?	AI	42/100 (42.0)	53/110 (48.2)	67/110 (60.9)	65/89 (73.0)	78/85 (91.8)	47/89 (52.8)
	H1	65/100 (65.0)	59/110 (53.6)	82/110 (74.5)	67/89 (75.3)	79/85 (92.9)	63/89 (70.8)
	H2	51/100 (51.0)	53/110 (48.2)	72/110 (65.5)	65/89 (73.0)	82/85 (96.5)	55/89 (61.8)
	*P* value	.01^a^	.73	.11	.88	.41	.04^a^
2. Which is AI?	AI	37/105 (35.2)	42/109 (38.5)	42/109 (38.5)	3/89 (3.4)	42/85 (49.4)	24/89 (27.0)
	H1	39/105 (37.1)	27/109 (24.8)	33/109 (30.3)	36/89 (40.4)	20/85 (23.5)	44/89 (49.4)
	H2	29/105 (27.6)	40/109 (36.7)	34/109 (31.2)	50/89 (56.2)	23/85 (27.1)	21/89 (23.6)
	*P* value	.51	.21	.48	<.001^a^	.004^a^	.004^a^
3. Which is most accurate?	AI	32/91 (35.2)	39/108 (36.1)	35/109 (32.1)	19/89 (21.3)	25/85 (29.4)	24/89 (27.0)
	H1	27/91 (29.7)	32/108 (29.6)	42/109 (38.5)	48/89 (53.9)	38/85 (44.7)	44/89 (49.4)
	H2	32/91 (35.2)	37/108 (34.3)	32/109 (29.4)	22/89 (24.7)	22/85 (25.9)	21/89 (23.6)
	*P* value	.78	.76	.45	<.001^a^	.04^a^	.004^a^

Abbreviations: AI, artificial intelligence; H, human; R, reviewer.

^a^ Statistically significant differences in frequency of yes answers for Q1 between AI and human traces for Fisher exact test *P* values (*P* < .05) and statistically significant bias in frequency of selection vs random selection for χ^2^ *P* values (*P* < .05).

For questions 2 and 3, reviewers at site 1 were not significantly biased toward any human or AI annotations in frequency of selecting which tracing was performed by AI and which was most accurate, while all reviewers at site 2 showed a statistically significantly bias (eg, question 3 for reviewer 1, 19 of 89 [21.3%] for AI; 48 of 89 [53.9%] for H1; 22 of 89 [24.7%] for H2; *P* < .001). At the second site, 2 of 3 reviewers identified human tracers as AI more frequently than AI. For example, reviewer 1 identified 3 of 89 AI tracings (3.4%) as AI and 50 of 89 tracings by H2 (56.2%) as AI (*P* < .001).

### Qualitative Evaluation of PGT Measurement

**Figure 3.**
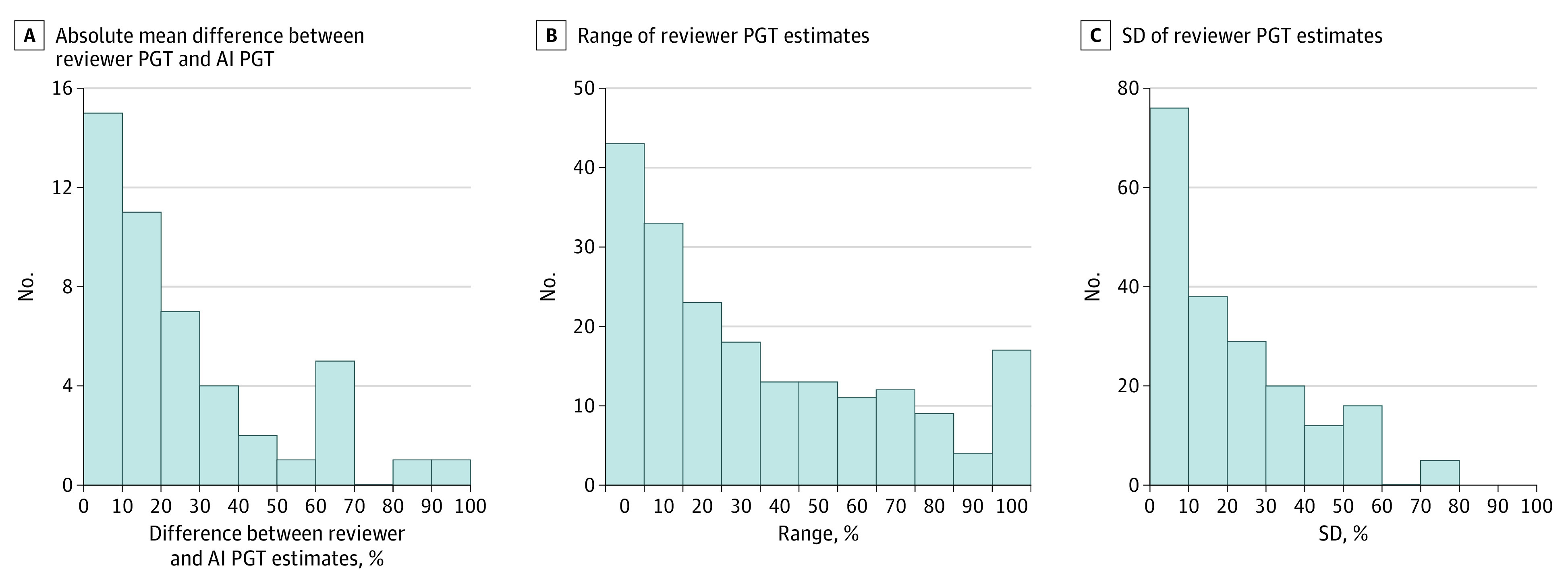
Quantitative Evaluation of Blinded Reviewer and Artificial Intelligence (AI) Percent Granulation Tissue (PGT) Assessments A, Histogram of absolute difference between the mean of the 3 reviewers’ visual PGT estimates and the AI PGT measurement for the subset of photographs with AI granulation tissue tracings at each site. B-C, Histograms of variability measures (range and SD) of the 3 reviewers’ visual PGT estimates for all photographs at each site.

Figure 3 shows histograms visualizing the distributions of absolute differences between AI PGT measurements and mean reviewer visual PGT estimates (Figure 3A) as well as the interreviewer PGT estimate variability measures (range and SD) (Figure 3B and 3C). Paired *t* tests indicated the mean absolute difference between AI PGT and mean reviewer PGT was significantly lower than the mean [SD] range across reviewer estimates (25% [24%] vs 35% [29%]; *P* = .03) and not significantly different from the mean [SD] SDs across reviewers (25% [24%] vs 19% [16%]; *P* = .14). For 17 photographs (8.5%), the visual PGT estimate range was 100% (ie, 1 reviewer found no granulation tissue and ≥1 other reviewer perceived the wound bed to be fully granulated), while for 99 photographs (49.7%) the range was within 20%, with complete agreement observed for 43 photographs (21.6%).

## Discussion

The major drivers of costs and outcomes in chronic wound care are healing time, treatment frequency, and wound complications.^26^ These factors all depend on accurate wound assessments, which are critical for guiding treatment plans. AI is playing an increasingly large role in optimizing diagnostic and therapeutic workflows and is starting to affect the area of wound care.^18,19,27,28,29,30^ Ongoing research and development in wound assessment devices and software aims to improve technologies and standardize practices. The eTable in the Supplement summarizes the potential advantages and disadvantages of AI-based digital wound assessment tools, which will be important to consider as these technologies become more prevalent across diverse wound care settings.

In this study, we developed an approach to quantitatively and qualitatively evaluate AI-based digital wound assessment tools using a large test set of wound photographs captured during routine patient encounters at 2 independent wound centers. The interpretation of wound area and granulation tissue in digital wound photographs by humans requires significant expertise developed over years of experience with wound care, and nevertheless, the wound boundary and other features are subject to significant variability between expert clinicians, as was shown in this study and in previous work.^13,31,32^ We found that while there was reasonable agreement in interpretation of the wound edge between 2 human annotators for most wounds, there were major discrepancies for a significant fraction of photographs, even though annotators followed a standardized area definition and were trained in a standardized tracing protocol (subgroups of discrepancy types are outlined in eAppendix 3 in the Supplement). Error measure distributions were generally similar between AI and human tracings compared with a human reference tracing for both wound area and granulation tissue, indicating AI performed similarly to the physician wound care specialists. Further research into cases with the highest error could give insight into which features of wound photographs pose challenges for consistent interpretation of the wound boundary by both humans and algorithms.

This study also found that relative error between tracings tended to increase significantly for smaller absolute wound sizes. Wound area is commonly quantified as a relative change from baseline or previous measurements. Percentage area change thresholds are often set for clinical trial end points or inclusion criteria, and a 40% decrease within 4 weeks is an often-cited prognostic marker for appropriately healing wounds.^2^ Based on these results, it may be important to consider that relative area changes may be less accurate when assessing small wounds (eg, when wounds are approaching closure) using these methods. Improving wound photograph quality and increasing image resolution would likely improve the accuracy of measurement of small wounds.

Visual wound assessment and manual annotation of wound boundaries are subjective; thus, it is important to consider the qualitative impressions of annotation accuracy by highly experienced clinicians. Here, qualitative assessment of area tracings further demonstrated the challenges in defining wound area. Reviewer 2 at site 2 found that nearly all tracings (91.8%-96.5%) met the wound area definition, but the remaining 5 reviewers found only moderate agreement of tracings with the set definition (range, 42.0%-75.3%), and the overall frequency of agreement was only 60.4% for AI and 68.0% for humans. Reviewers at site 1 did not show a significant bias in selecting which tracing they thought was most accurate, while reviewers at site 2 all showed bias toward tracings by H1, potentially indicating different skill levels of the human tracers at site 2. To qualitatively assess how well the appearance of AI tracings matched human tracings, reviewers were asked which tracing they thought was AI. While reviewers at site 1 showed no statistically significant bias in which tracing they thought was performed by AI, reviewers at site 2 did show a bias, with 2 of 3 reviewers picking human tracings as AI more frequently than the AI tracing. Thus, there may have been subtle but perceptible differences in the appearance of the tracings between AI and humans.

Accurate assessment of PGT area is also important for providing optimal wound care, but most studies and clinical practices rely on visual estimation. While there is generally a high-contrast edge at the wound boundary to aid in identifying the wound area, the area of granulation tissue may be particularly challenging to define. Healthy granulation is described as pink to a varying degree of red.^21,22^ Unhealthy tissue can also be red, but typically ranges from dusky to white or yellow. Photographic color accuracy can be affected by factors including poor lighting or the quality of the camera. In this study, visual approximation of PGT varied considerably across reviewers, and granulation tracing error measures also varied more widely compared with area tracings. However, AI granulation tracing errors were statistically similar to human tracings, and the difference between the AI PGT measurements and reviewer visual estimates were of a similar magnitude to interreviewer differences, suggesting equivalent performance to human annotators. Even if challenges remain to accurately identifying granulation tissue in wound images, the use of software tools will in theory provide greater consistency and reproducibility compared with manual visual estimation.

Together, these results indicate that defining criterion-standard wound area and granulation tissue annotations for a broad range of wound types is challenging. However, AI technologies have the capacity to perform wound annotations with proficiency similar to human wound care specialists.

Future directions include expanding the assessment methods for other wound features and prospectively tracking the same wounds over time with relevant demographic and clinical wound characteristics to evaluate their association with wound progression. There is potential to use machine learning to detect wounds that may be slow to heal or require prompt medical attention,^27^ allowing triage of care while decreasing strain on health care resources.

### Limitations

This study has limitations. We observed considerable variability in photograph quality, as there was no standard device or protocol used for photography. Consequently, epithelialized and granulated areas may be difficult to interpret in some photographs. There is a need for standardization in wound photography using devices and algorithms to improve data quality.

## Conclusions

This study developed a framework for evaluating AI-based digital wound assessment tools. It is challenging for expert clinicians to agree on wound assessments, which has made it difficult to accurately quantify key wound healing end points. Clinicians need clear, quick, and precise wound analysis to guide best clinical practices. Therefore, further work to standardize the testing and use of AI-based digital wound assessment tools is warranted. We hypothesize that a structured approach to wound assessment using advanced technology, such as AI, can lead to greater treatment efficacy and improved outcomes for patients with chronic wounds.

## Notes

Supplement. **eTable****.** Summary Table of the Advantages and Disadvantages of Using AI-Based Digital Wound Assessment Tools **eAppendix 1.** Individual Photographs From Site 1 **eAppendix 2.** Individual Photographs From Site 2 **eAppendix 3.** Supplementary Discussion

## References

[1] Olsson M, Järbrink K, Divakar U, . The humanistic and economic burden of chronic wounds: a systematic review. Wound Repair Regen. 2019;27(1):114-125. doi:10.1111/wrr.12683 30362646

[2] Sheehan P, Jones P, Caselli A, Giurini JM, Veves A. Percent change in wound area of diabetic foot ulcers over a 4-week period is a robust predictor of complete healing in a 12-week prospective trial. Diabetes Care. 2003;26(6):1879-1882. doi:10.2337/diacare.26.6.1879 12766127

[3] Foltynski P, Wojcicki JM, Ladyzynski P, Sabalinska S. A Comparison of Three Techniques for Wound Area Measurement. In: XIII Mediterranean Conference on Medical and Biological Engineering and Computing 2013. IFMBE; 2014: 1071–1074. doi:10.1007/978-3-319-00846-2_265

[4] Langemo DK, Melland H, Hanson D, Olson B, Hunter S, Henly SJ. Two-dimensional wound measurement: comparison of 4 techniques. Adv Wound Care. 1998;11(7):337-343. 10326350

[5] Bryant JL, Brooks TL, Schmidt B, Mostow EN. Reliability of wound measuring techniques in an outpatient wound center. Ostomy Wound Manage. 2001;47(4):44-51. 11890088

[6] Shah A, Wollak C, Shah JBB. Wound measurement techniques: comparing the use of ruler method, 2D imaging and 3D scanner. J Am Coll Clin Wound Spec. 2015;5(3):52-57. doi:10.1016/j.jccw.2015.02.001 26199893 PMC4495754

[7] Rogers LC, Bevilacqua NJ, Armstrong DG, Andros G. Digital planimetry results in more accurate wound measurements: a comparison to standard ruler measurements. J Diabetes Sci Technol. 2010;4(4):799-802. doi:10.1177/193229681000400405 20663440 PMC2909508

[8] Gethin G, Cowman S. Wound measurement comparing the use of acetate tracings and Visitrak digital planimetry. J Clin Nurs. 2006;15(4):422-427. doi:10.1111/j.1365-2702.2006.01364.x 16553755

[9] Hammond CE, Nixon MA. The reliability of a handheld wound measurement and documentation device in clinical practice. J Wound Ostomy Continence Nurs. 2011;38(3):260-264. doi:10.1097/WON.0b013e318215fc60 21483270

[10] Wendelken ME, Berg WT, Lichtenstein P, Markowitz L, Comfort C, Alvarez OM. Wounds measured from digital photographs using photographs using photo-digital planimetry software: validation and rater reliability. Wounds. 2011;23(9):267-275. 25879267

[11] Bilgin M, Güneş ÜY. A comparison of 3 wound measurement techniques: effects of pressure ulcer size and shape. J Wound Ostomy Continence Nurs. 2013;40(6):590-593. doi:10.1097/01.WON.0000436668.79024.f9 24202222

[12] Flanagan M. The characteristics and formation of granulation tissue. J Wound Care. 1998;7(10):508-510. doi:10.12968/jowc.1998.7.10.508 10188445

[13] Vermeulen H, Ubbink DT, Schreuder SM, Lubbers MJ. Inter- and intra-observer (dis)agreement among nurses and doctors to classify colour and exudation of open surgical wounds according to the Red-Yellow-Black scheme. J Clin Nurs. 2007;16(7):1270-1277. doi:10.1111/j.1365-2702.2007.01789.x 17584345

[14] McGuiness W, Dunn SV, Jones MJ. Developing an accurate system of measuring colour in a venous leg ulcer in order to assess healing. J Wound Care. 2005;14(6):249-254. doi:10.12968/jowc.2005.14.6.26791 15974410

[15] Hampton S. Understanding overgranulation in tissue viability practice. Br J Community Nurs. 2007;12(9):S24-S30. doi:10.12968/bjcn.2007.12.Sup4.43000 18026011

[16] Iizaka S, Sugama J, Nakagami G, . Concurrent validation and reliability of digital image analysis of granulation tissue color for clinical pressure ulcers. Wound Repair Regen. 2011;19(4):455-463. doi:10.1111/j.1524-475X.2011.00686.x 21518090

[17] Iizaka S, Kaitani T, Sugama J, . Predictive validity of granulation tissue color measured by digital image analysis for deep pressure ulcer healing: a multicenter prospective cohort study. Wound Repair Regen. 2013;21(1):25-34. doi:10.1111/j.1524-475X.2012.00841.x 23110386

[18] He J, Baxter SL, Xu J, Xu J, Zhou X, Zhang K. The practical implementation of artificial intelligence technologies in medicine. Nat Med. 2019;25(1):30-36. doi:10.1038/s41591-018-0307-0 30617336 PMC6995276

[19] Mamone V, Fonzo MD, Esposito N, Ferrari M, Ferrari V. Monitoring wound healing with contactless measurements and augmented reality. IEEE J Transl Eng Health Med. 2020;8:2700412. doi:10.1109/JTEHM.2020.2983156 32373400 PMC7198047

[20] Kieser DC, Hammond C. Leading wound care technology: the ARANZ medical silhouette. Adv Skin Wound Care. 2011;24(2):68-70. doi:10.1097/01.ASW.0000394028.64777.f7 21242735

[21] Schneider CA, Rasband WS, Eliceiri KW. NIH Image to ImageJ: 25 years of image analysis. Nat Methods. 2012;9(7):671-675. doi:10.1038/nmeth.2089 22930834 PMC5554542

[22] Abràmoff MD, Magalhães PJ, Ram SJ. Image processing with ImageJ. Biophotonics Int. 2004;11:36-41. doi:10.1201/9781420005615.ax4

[23] Dong H, Yang G, Liu F, Mo Y, Guo Y. Automatic brain tumor detection and segmentation using U-net based fully convolutional networks. In: Medical Image Understanding and Analysis. Springer Verlag, 2017:506-517. doi:10.1007/978-3-319-60964-5_44

[24] Novikov AA, Lenis D, Major D, Hladuvka J, Wimmer M, Buhler K. Fully convolutional architectures for multiclass segmentation in chest radiographs. IEEE Trans Med Imaging. 2018;37(8):1865-1876. doi:10.1109/TMI.2018.2806086 29994439

[25] Gould L, Li WW. Defining complete wound closure: closing the gap in clinical trials and practice. Wound Repair Regen. 2019;27(3):201-224. doi:10.1111/wrr.12707 30767334

[26] Lindholm C, Searle R. Wound management for the 21st century: combining effectiveness and efficiency. Int Wound J. 2016;13(suppl 2):5-15. doi:10.1111/iwj.12623 27460943 PMC7949725

[27] Jung K, Covington S, Sen CK, . Rapid identification of slow healing wounds. Wound Repair Regen. 2016;24(1):181-188. doi:10.1111/wrr.12384 26606167 PMC4820011

[28] Woods JS, Saxena M, Nagamine T, . The future of data-driven wound care. AORN J. 2018;107(4):455-463. doi:10.1002/aorn.12102 29595902

[29] Queen D. Artificial intelligence and machine learning in wound care: the wounded machine! Int Wound J. 2019;16(2):311. doi:10.1111/iwj.13108 30887702 PMC7948899

[30] Queen D, Harding K. Data-driven specialisation of wound care through artificial intelligence. Int Wound J. 2019;16(4):879-880. doi:10.1111/iwj.13166 31328898 PMC7948846

[31] Flanagan M. Improving accuracy of wound measurement in clinical practice. Ostomy Wound Manage. 2003;49(10):28-40. 14652419

[32] Buntinx F, Beckers H, De Keyser G, . Inter-observer variation in the assessment of skin ulceration. J Wound Care. 1996;5(4):166-170. doi:10.12968/jowc.1996.5.4.166 8826261

