# Supplementary material for: Development of a Method for Clinical Evaluation of Artificial Intelligence–Based Digital Wound Assessment Tools
Source: JAMA Netw Open. 2021 May 19;4(5):e217234. doi: 10.1001/jamanetworkopen.2021.7234 (PMC8134996; doi:10.1001/jamanetworkopen.2021.7234)
Supplement: Supplement. — eTable. Summary Table of the Advantages and Disadvantages of Using AI-Based Digital Wound Assessment Tools eAppendix 1. Individual Photographs From Site 1 eAppendix 2. Individual Photographs From Site 2 eAppendix 3. Supplementary Discussion [file jamanetwopen-e217234-s001.pdf]

Supplemental Online Content

Howell RS, Liu HH, Khan AA, et al. Development of a method for clinical evaluation of artificial intelligence–based digital wound assessment tools. *JAMA Netw Open.* 2021;4(5):e217234. doi:10.1001/jamanetworkopen.2021.7234

eTable. Summary Table of the Advantages and Disadvantages of Using AI-Based Digital Wound Assessment Tools

eAppendix 1. Individual Photographs From Site 1

eAppendix 2. Individual Photographs From Site 2

eAppendix 3. Supplementary Discussion

This supplemental material has been provided by the authors to give readers additional information about their work.

© 2021 Howell RS et al. *JAMA Network Open.*

s

eTable. Summary Table of the Advantages and Disadvantages of Using AI-Based Digital Wound Assessment Tools

Advantages	Disadvantages
• Reduced human measurement error<br>• Improved workflow and operation efficiency<br>• Contactless wound assessment<br>• Consistency across all providers	• Lack of standardized guidelines for using AI-based tools in wound assessment applications<br>• A need to trust system programmers<br>• Potential challenges for integration with other health information technology systems

© 2021 Howell RS et al. *JAMA Network Open.*

eAppendix 1. Individual Photographs From Site 1

Wound EMR Information								
Sequential Number	Gender	Age	Wound Type	Wound Location	Wound Length (cm)	Wound Width (cm)	Wound Depth (cm)	Wound Area (cm^2)
1	M	66	VLU	RLE ant	1.5	1.3	0.1	1.95

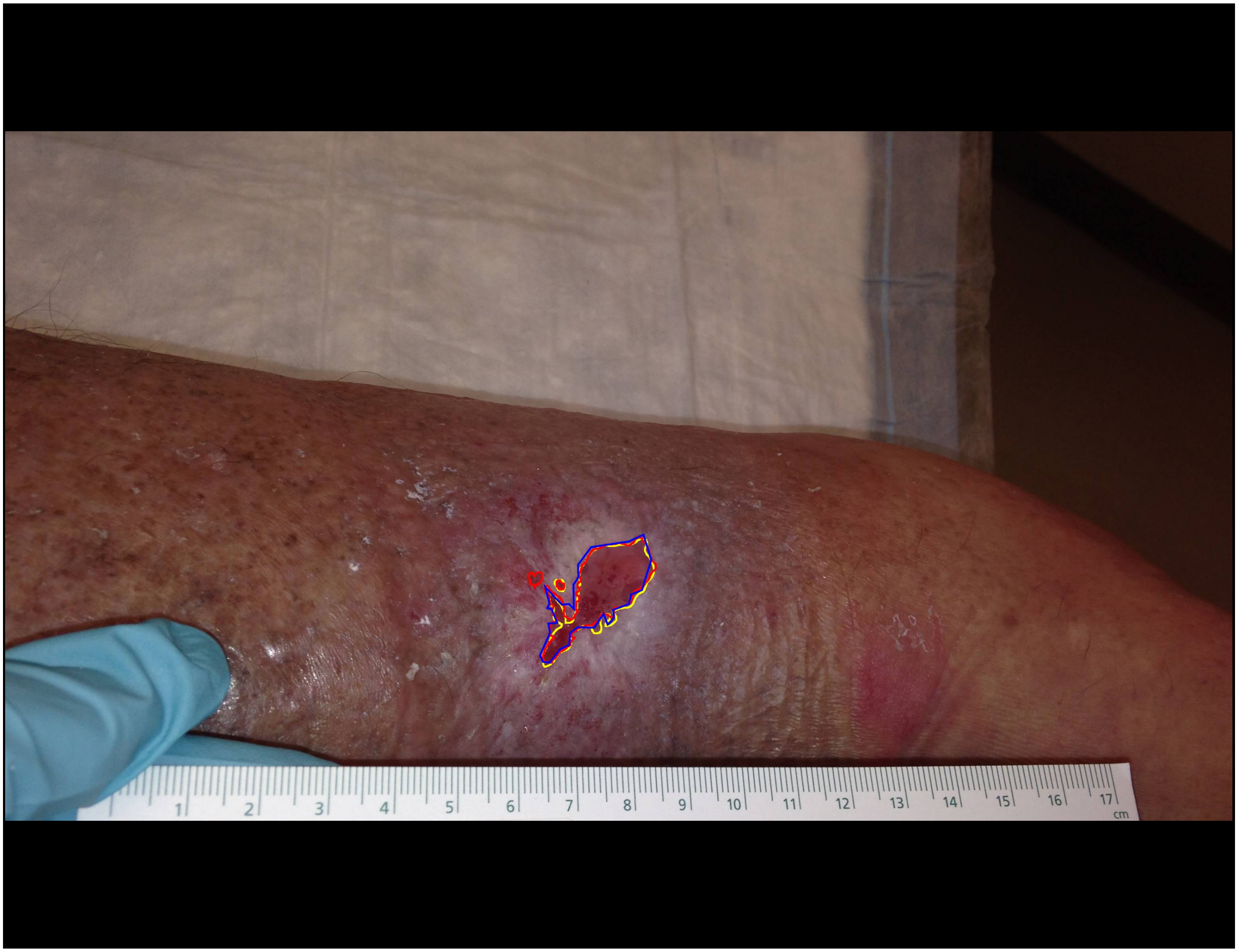

Tracing Data			
Tracer:	Wound Area (px^2)	Ruler Calibration (px/cm)	Wound Area (cm^2)
H1	41144	154.7	1.72
H2	41143	150.6	1.81
AI	42936	189.2	1.20

Tracing Comparisons				
Difference Metric:	Human-Human		Human-AI	
	H1(ref) H2(test)	H2(ref) H1(test)	H1(ref) AI(test)	H2(ref) AI(test)
False Negative Area (%)	8.6	8.6	9.2	7.4
False Positive Area (%)	8.6	8.6	13.5	11.7
|Relative Error (%)|	0.0	0.0	4.4	4.4

Blinded Attending Surgeon Review						
Reviewer	PGT Estimate	H1 meets definition?	H2 meets definition?	AI meets definition?	Which is AI?	Which is most accurate?
1	100	Yes	No	Yes	H1	AI
2	90	Yes	No	No	H1	AI
3	30	Yes	Yes	No	H2	AI

© 2021 Howell RS et al. *JAMA Network Open.*

Wound EMR Information								
Sequential Number	Gender	Age	Wound Type	Wound Location	Wound Length (cm)	Wound Width (cm)	Wound Depth (cm)	Wound Area (cm^2)
2	M	51	VLU	LLE medial	0.6	0.3	0.1	0.18

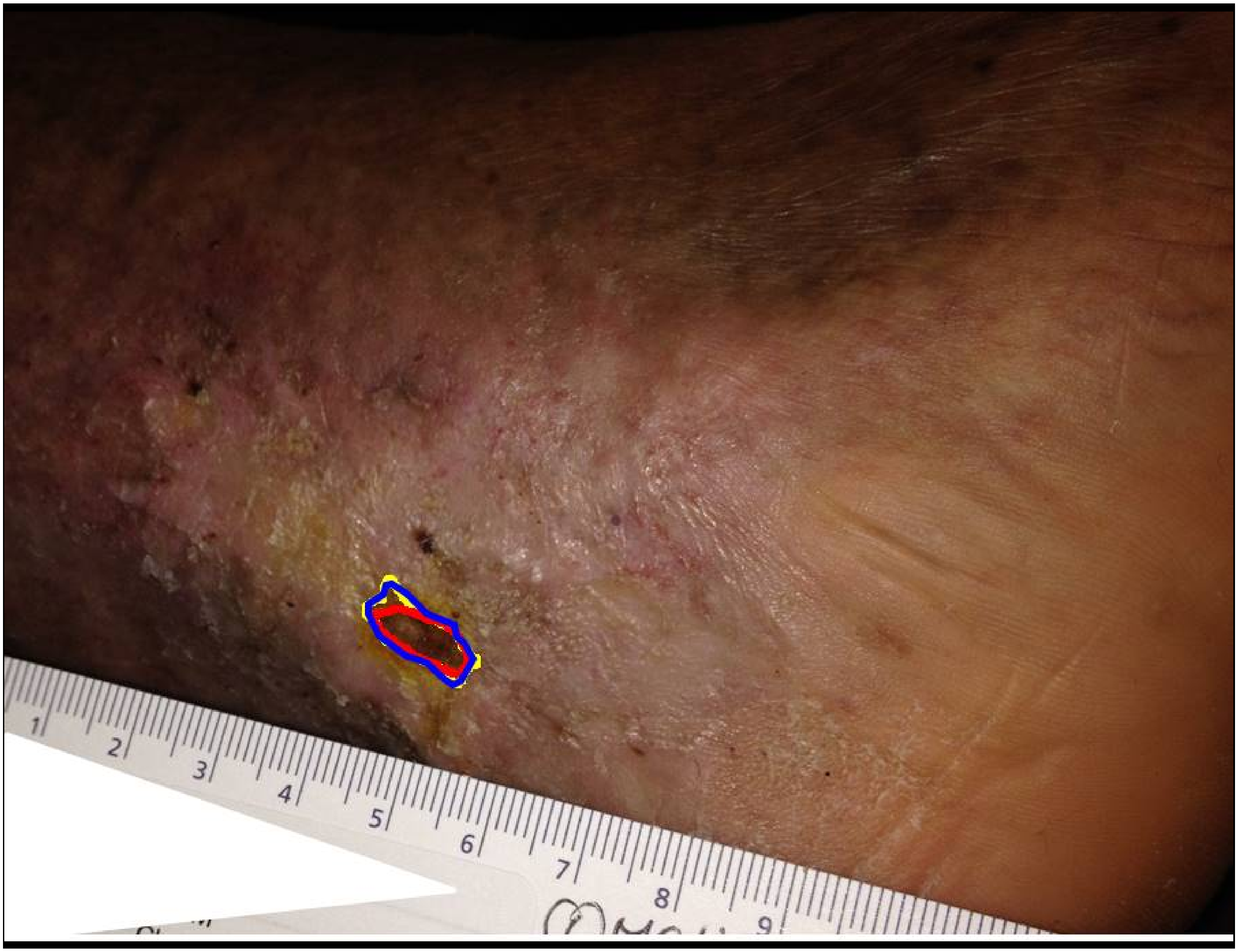

Tracing Data			
Tracer:	Wound Area (px^2)	Ruler Calibration (px/cm)	Wound Area (cm^2)
H1	2395	67.2	0.53
H2	1769	68.1	0.38
AI	2702	62.1	0.70

Tracing Comparisons				
Difference Metric:	Human-Human		Human-AI	
	H1(ref) H2(test)	H2(ref) H1(test)	H1(ref) AI(test)	H2(ref) AI(test)
False Negative Area (%)	27.1	1.2	3.4	0.6
False Positive Area (%)	0.9	36.6	16.2	53.3
|Relative Error (%)|	26.1	35.4	12.8	52.7

Blinded Attending Surgeon Review						
Reviewer	PGT Estimate	H1 meets definition?	H2 meets definition?	AI meets definition?	Which is AI?	Which is most accurate?
1	0	No	Yes	No	H2	H2
2	0	Yes	No	Yes	H2	AI
3	80	Yes	Yes	Yes	AI	H2

© 2021 Howell RS et al. *JAMA Network Open.*

Wound EMR Information								
Sequential Number	Gender	Age	Wound Type	Wound Location	Wound Length (cm)	Wound Width (cm)	Wound Depth (cm)	Wound Area (cm^2)
3	M	56	PU	R heel	2.3	1.9	0.1	4.37

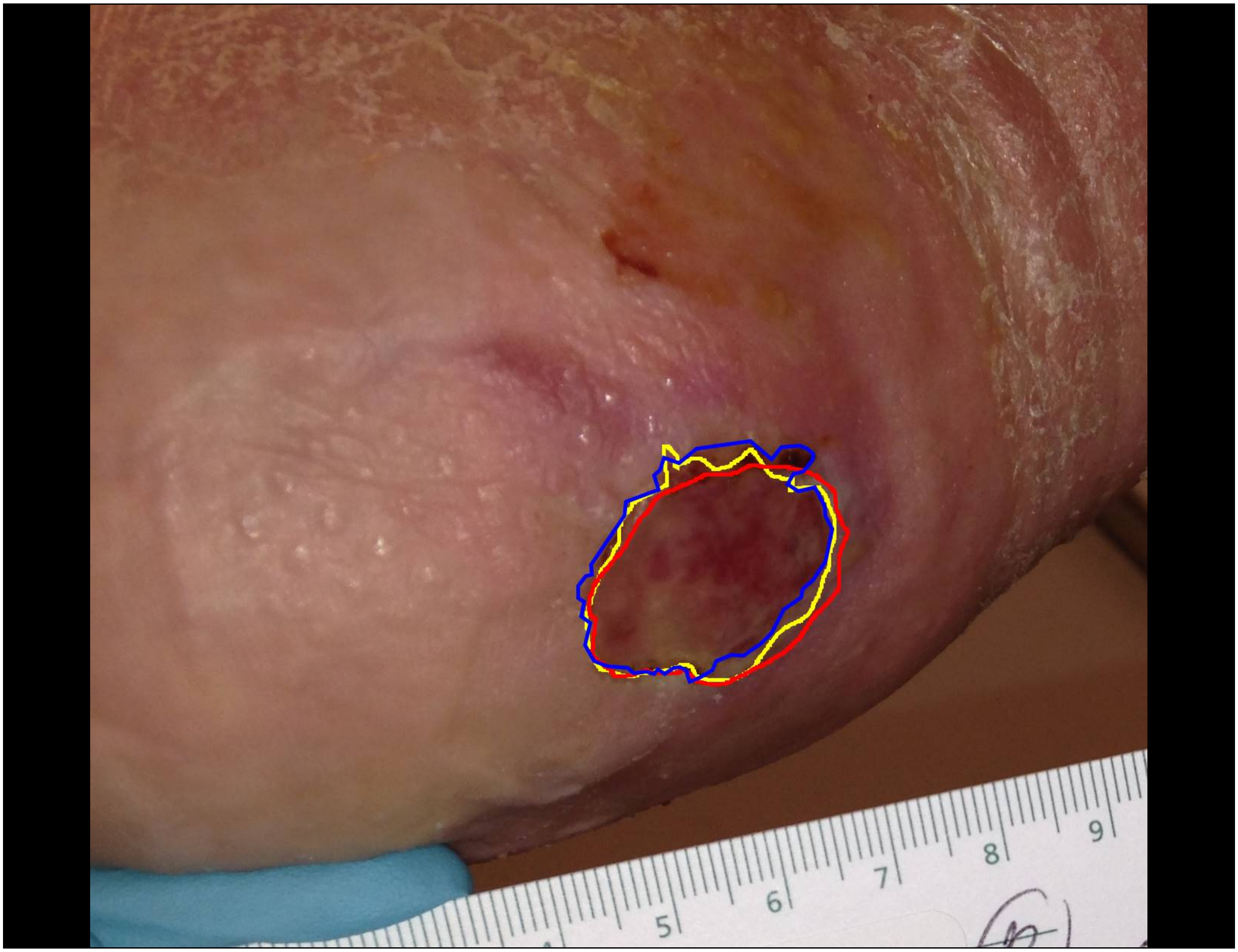

Tracing Data			
Tracer:	Wound Area (px^2)	Ruler Calibration (px/cm)	Wound Area (cm^2)
H1	71040	150.7	3.13
H2	71941	151.7	3.13
AI	72421	155.4	3.00

Tracing Comparisons				
Difference Metric:	Human-Human		Human-AI	
	H1(ref) H2(test)	H2(ref) H1(test)	H1(ref) AI(test)	H2(ref) AI(test)
False Negative Area (%)	7.3	8.4	7.3	13.8
False Positive Area (%)	8.5	7.2	9.2	14.5
|Relative Error (%)|	1.3	1.3	1.9	0.7

Blinded Attending Surgeon Review						
Reviewer	PGT Estimate	H1 meets definition?	H2 meets definition?	AI meets definition?	Which is AI?	Which is most accurate?
1	40	Yes	No	No	AI	H2
2	20	Yes	No	Yes	AI	AI
3	40	Yes	Yes	Yes	AI	H2

© 2021 Howell RS et al. *JAMA Network Open.*

Wound EMR Information								
Sequential Number	Gender	Age	Wound Type	Wound Location	Wound Length (cm)	Wound Width (cm)	Wound Depth (cm)	Wound Area (cm^2)
4	M	62	lymphedem	RLE post	1.0	0.9		0.90

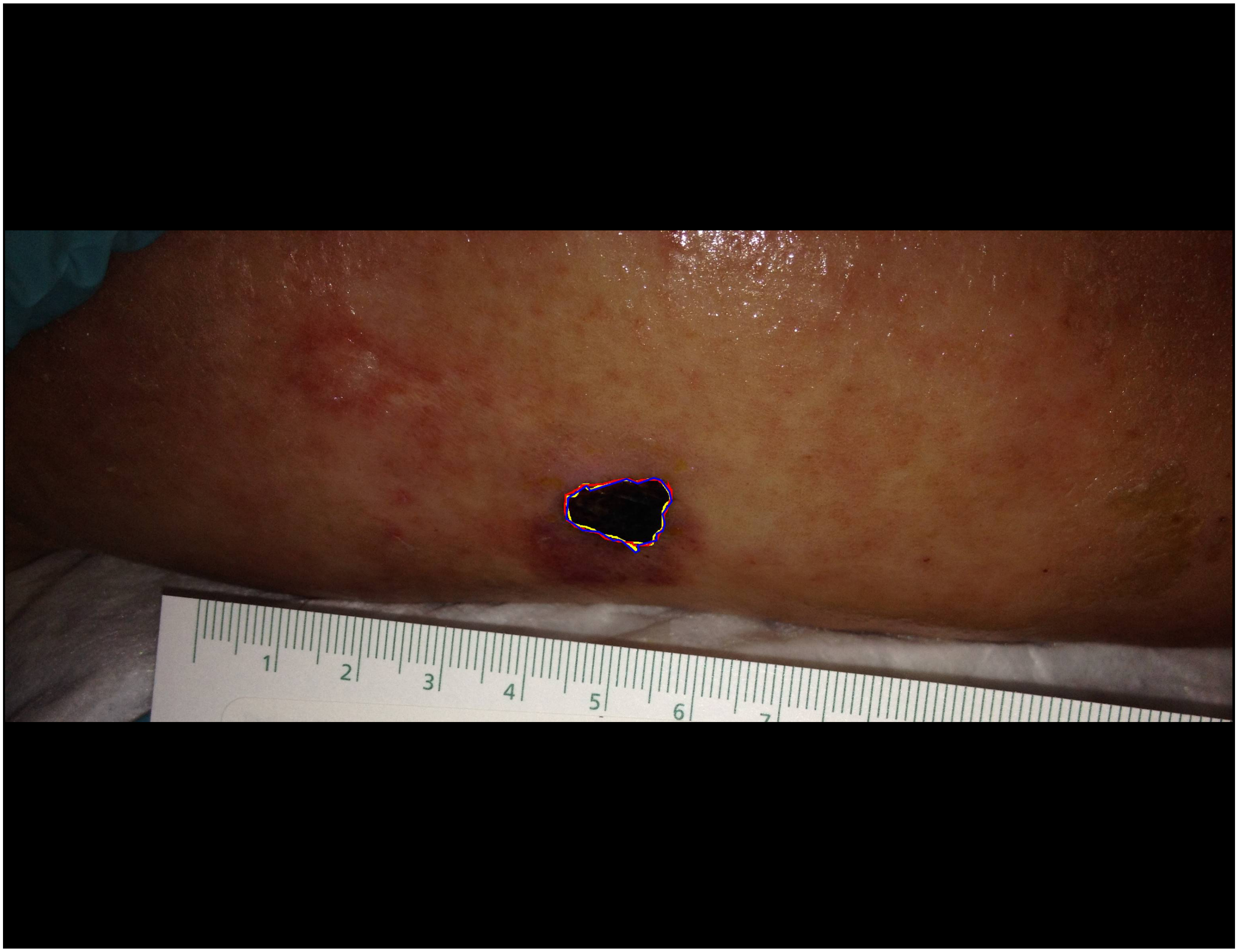

Tracing Data			
Tracer:	Wound Area (px^2)	Ruler Calibration (px/cm)	Wound Area (cm^2)
H1	35028	222.1	0.71
H2	37977	223.5	0.76
AI	36333	213.1	0.80

Tracing Comparisons				
Difference Metric:	Human-Human		Human-AI	
	H1(ref) H2(test)	H2(ref) H1(test)	H1(ref) AI(test)	H2(ref) AI(test)
False Negative Area (%)	0.8	8.5	2.8	6.4
False Positive Area (%)	9.2	0.7	6.5	2.1
|Relative Error (%)|	8.4	7.8	3.7	4.3

Blinded Attending Surgeon Review						
Reviewer	PGT Estimate	H1 meets definition?	H2 meets definition?	AI meets definition?	Which is AI?	Which is most accurate?
1		Yes	Yes	Yes	AI	0
2	0	Yes	No	No	H2	H2
3		No	Yes	No	H2	AI

© 2021 Howell RS et al. *JAMA Network Open.*

Wound EMR Information								
Sequential Number	Gender	Age	Wound Type	Wound Location	Wound Length (cm)	Wound Width (cm)	Wound Depth (cm)	Wound Area (cm^2)
5	F	34	Surgical	Abdomen	0.6	0.4	0.1	0.24

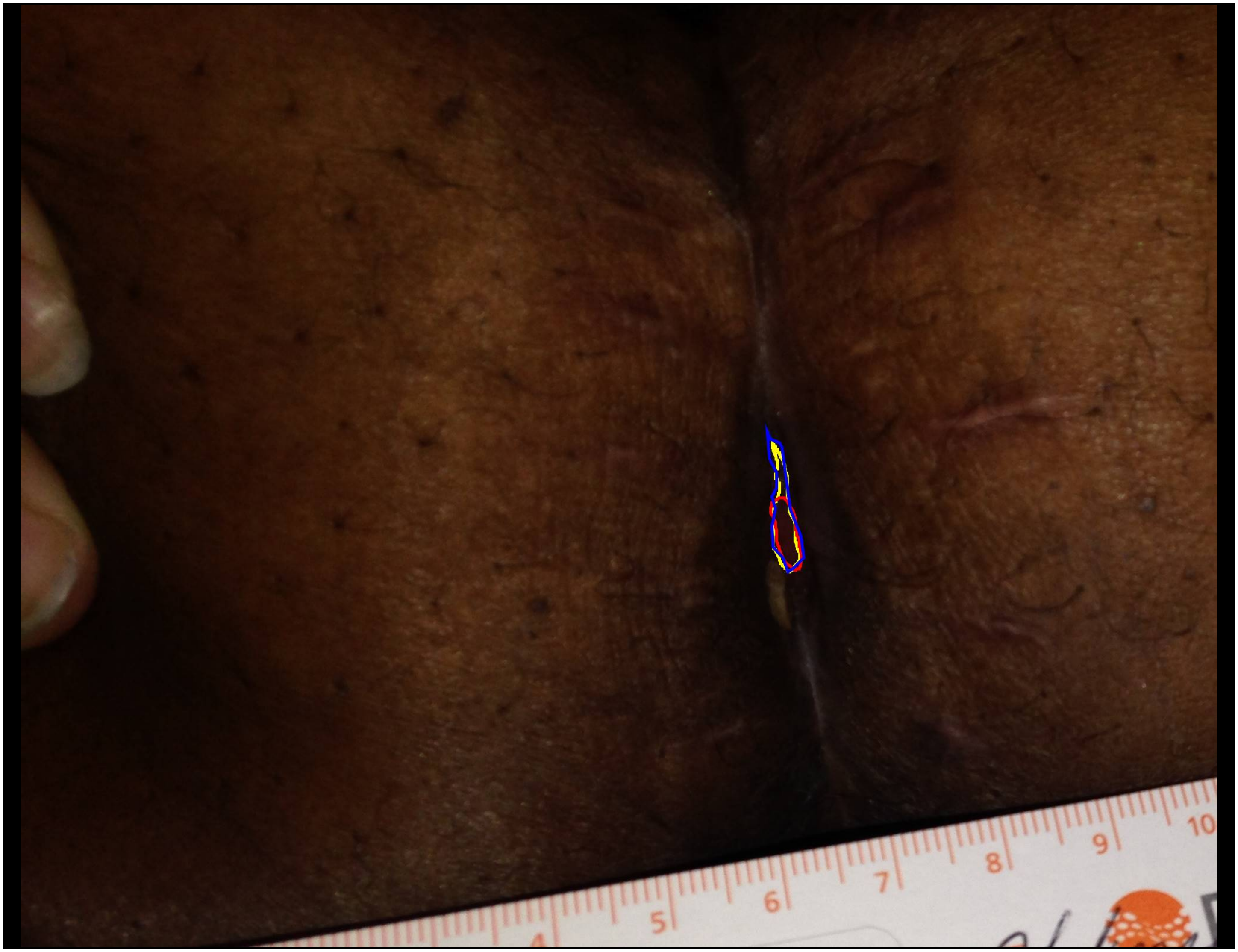

Tracing Data			
Tracer:	Wound Area (px^2)	Ruler Calibration (px/cm)	Wound Area (cm^2)
H1	4997	188.9	0.14
H2	3821	190.8	0.11
AI	6310	251.2	0.10

Tracing Comparisons				
Difference Metric:	Human-Human		Human-AI	
	H1(ref) H2(test)	H2(ref) H1(test)	H1(ref) AI(test)	H2(ref) AI(test)
False Negative Area (%)	34.6	14.4	4.9	7.6
False Positive Area (%)	11.0	45.2	31.2	72.7
|Relative Error (%)|	23.5	30.8	26.3	65.1

Blinded Attending Surgeon Review						
Reviewer	PGT Estimate	H1 meets definition?	H2 meets definition?	AI meets definition?	Which is AI?	Which is most accurate?
1		Yes	Yes	Yes	0	0
2	90	Yes	No	No	H2	H2
3	10	Yes	No	No	AI	H1

© 2021 Howell RS et al. *JAMA Network Open.*

Wound EMR Information								
Sequential Number	Gender	Age	Wound Type	Wound Location	Wound Length (cm)	Wound Width (cm)	Wound Depth (cm)	Wound Area (cm^2)
6	F	36	VLU	LLE lat	0.9	1.0	0.1	0.90

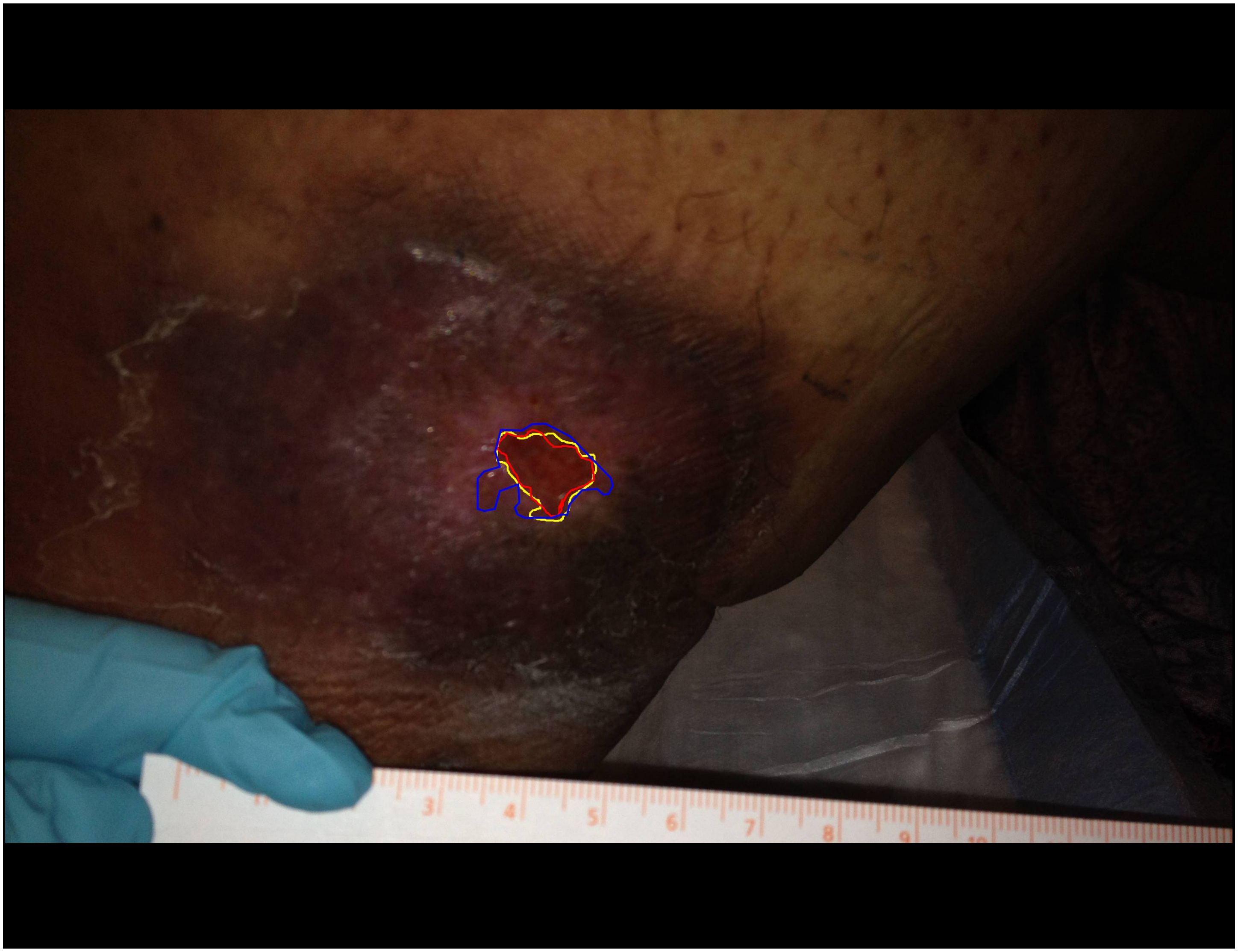

Tracing Data			
Tracer:	Wound Area (px^2)	Ruler Calibration (px/cm)	Wound Area (cm^2)
H1	38563	220.9	0.79
H2	31626	224.6	0.63
AI	56592	226.8	1.10

Tracing Comparisons				
Difference Metric:	Human-Human		Human-AI	
	H1(ref) H2(test)	H2(ref) H1(test)	H1(ref) AI(test)	H2(ref) AI(test)
False Negative Area (%)	19.6	2.0	1.9	0.0
False Positive Area (%)	1.6	23.9	48.6	78.9
|Relative Error (%)|	18.0	21.9	46.8	78.9

Blinded Attending Surgeon Review						
Reviewer	PGT Estimate	H1 meets definition?	H2 meets definition?	AI meets definition?	Which is AI?	Which is most accurate?
1	100	No	Yes	No	H1	H2
2	40	Yes	Yes	No	H2	H1
3	20	No	No	Yes	AI	H1

© 2021 Howell RS et al. *JAMA Network Open.*

Wound EMR Information								
Sequential Number	Gender	Age	Wound Type	Wound Location	Wound Length (cm)	Wound Width (cm)	Wound Depth (cm)	Wound Area (cm^2)
7	M	90	Trauma	LLE ant	1.1	0.7	0.3	0.77

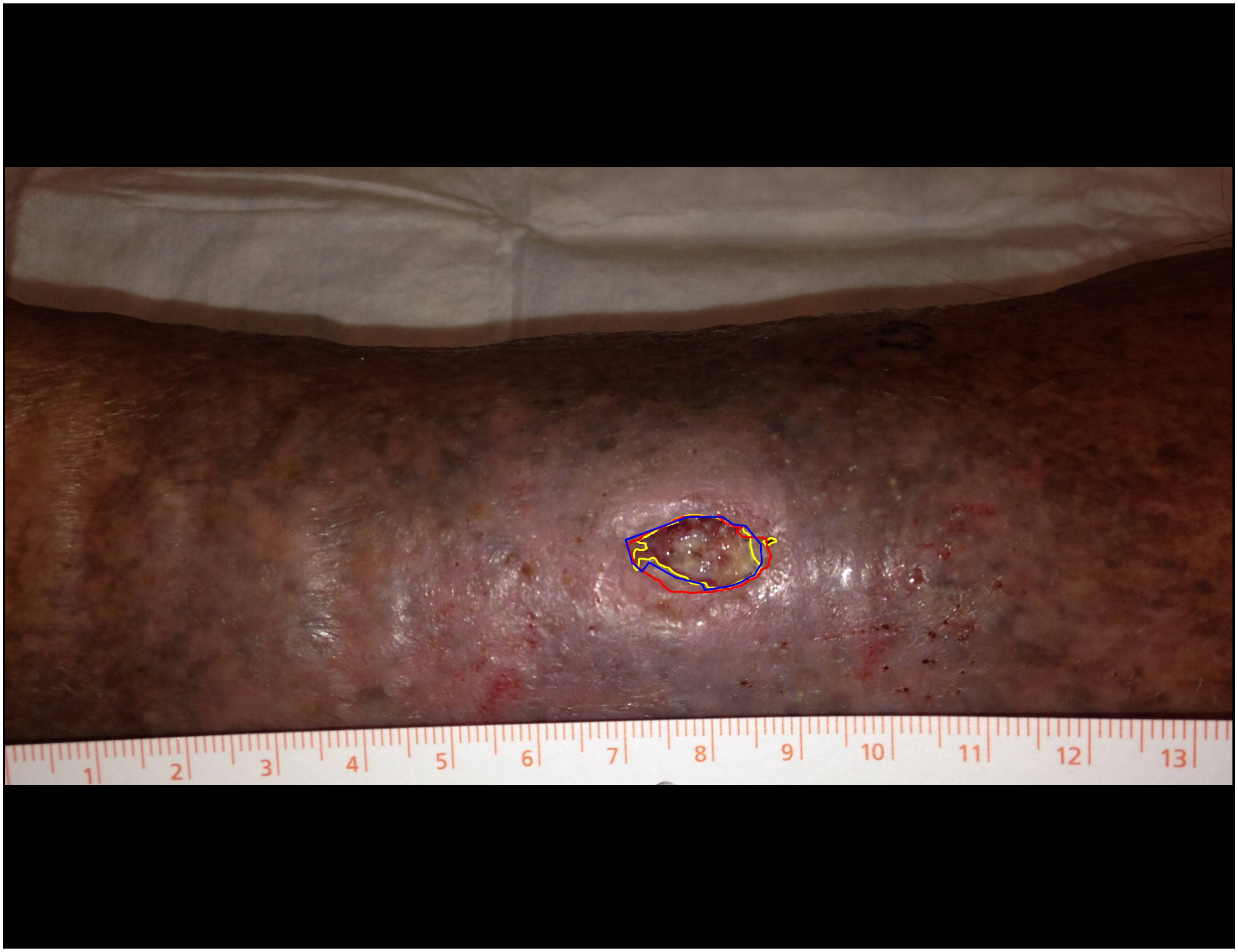

Tracing Data			
Tracer:	Wound Area (px^2)	Ruler Calibration (px/cm)	Wound Area (cm^2)
H1	43528	234.7	0.79
H2	55378	237.4	0.98
AI	49141	247.8	0.80

Tracing Comparisons				
Difference Metric:	Human-Human		Human-AI	
	H1(ref) H2(test)	H2(ref) H1(test)	H1(ref) AI(test)	H2(ref) AI(test)
False Negative Area (%)	2.6	23.4	2.7	14.7
False Positive Area (%)	29.8	2.0	15.6	3.5
|Relative Error (%)|	27.2	21.4	12.9	11.3

Blinded Attending Surgeon Review						
Reviewer	PGT Estimate	H1 meets definition?	H2 meets definition?	AI meets definition?	Which is AI?	Which is most accurate?
1	30	No	Yes	No	H2	H1
2	<10	No	No	Yes	AI	AI
3	50	No	Yes	No	AI	H2

© 2021 Howell RS et al. *JAMA Network Open.*

Wound EMR Information								
Sequential Number	Gender	Age	Wound Type	Wound Location	Wound Length (cm)	Wound Width (cm)	Wound Depth (cm)	Wound Area (cm^2)
8	F	85	VLU	RLE post	7.6	3.8	0.1	28.88

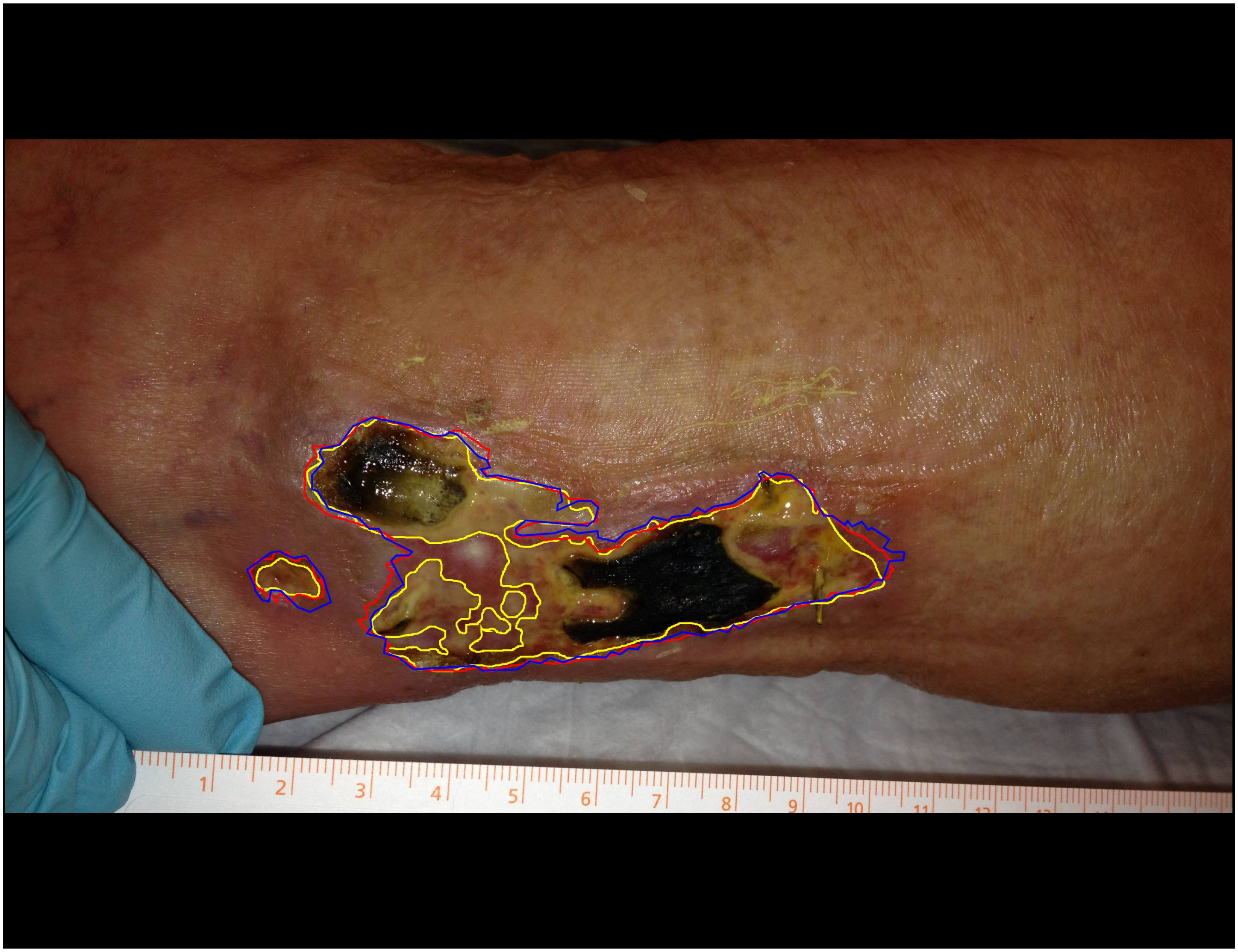

Tracing Data			
Tracer:	Wound Area (px^2)	Ruler Calibration (px/cm)	Wound Area (cm^2)
H1	474567	204.1	11.39
H2	579135	190.4	15.98
AI	582490	212.5	12.90

Tracing Comparisons				
Difference Metric:	Human-Human		Human-AI	
	H1(ref) H2(test)	H2(ref) H1(test)	H1(ref) AI(test)	H2(ref) AI(test)
False Negative Area (%)	1.2	19.0	1.4	3.8
False Positive Area (%)	23.2	1.0	24.1	4.4
|Relative Error (%)|	22.0	18.1	22.7	0.6

Blinded Attending Surgeon Review						
Reviewer	PGT Estimate	H1 meets definition?	H2 meets definition?	AI meets definition?	Which is AI?	Which is most accurate?
1	20	No	No	No	H1	None
2	10	No	Yes	No	H2	H1
3	40	Yes	Yes	Yes	H1	AI

© 2021 Howell RS et al. *JAMA Network Open.*

Wound EMR Information								
Sequential Number	Gender	Age	Wound Type	Wound Location	Wound Length (cm)	Wound Width (cm)	Wound Depth (cm)	Wound Area (cm^2)
9	M	68	lymphedem	LLE lat	3.0	6.0	0.1	18.00

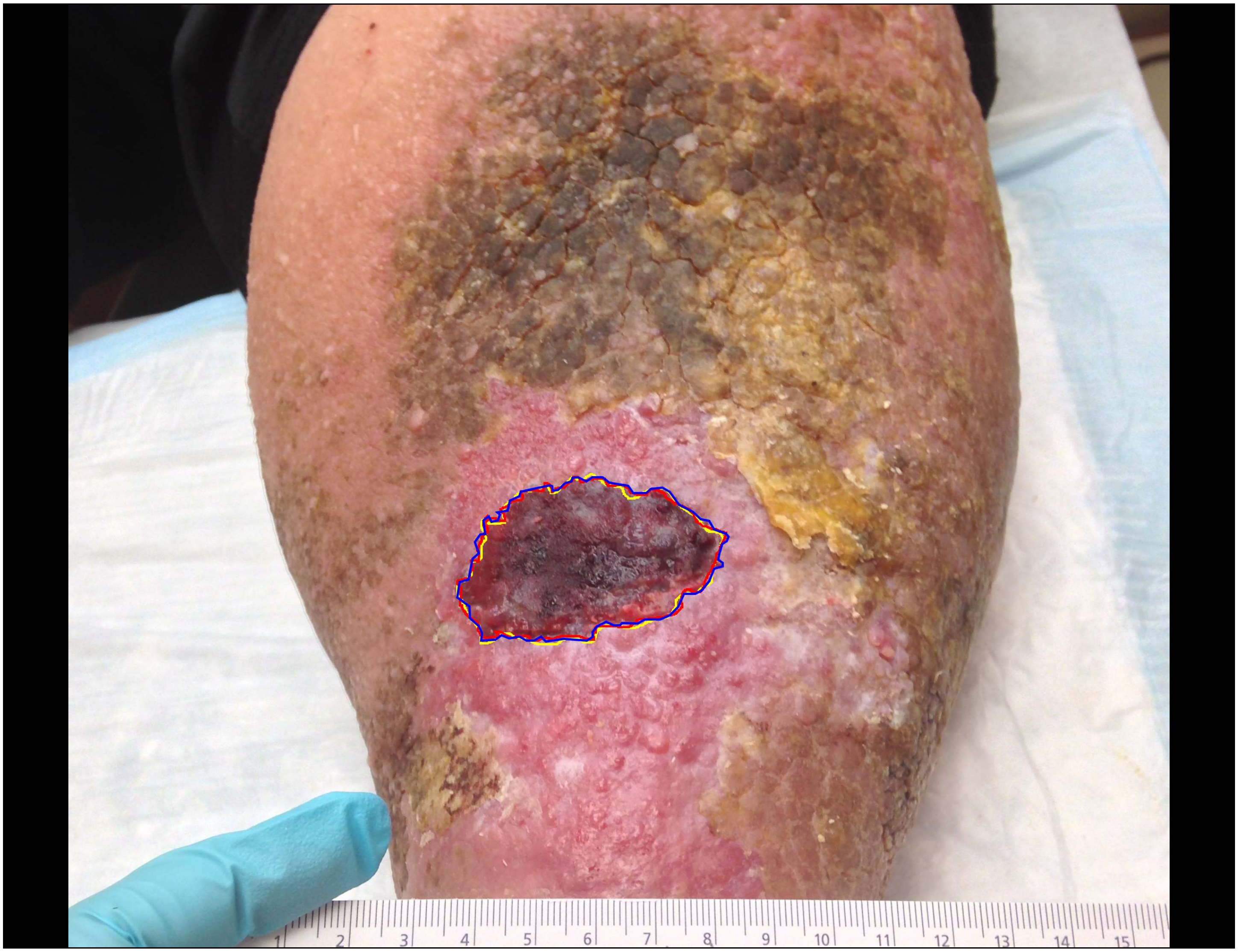

Tracing Data			
Tracer:	Wound Area (px^2)	Ruler Calibration (px/cm)	Wound Area (cm^2)
H1	154471	134.0	8.60
H2	152411	132.1	8.74
AI	156340	138.9	8.10

Tracing Comparisons				
Difference Metric:	Human-Human		Human-AI	
	H1(ref) H2(test)	H2(ref) H1(test)	H1(ref) AI(test)	H2(ref) AI(test)
False Negative Area (%)	3.5	2.2	2.0	1.3
False Positive Area (%)	2.2	3.6	3.3	3.9
|Relative Error (%)|	1.3	1.4	1.2	2.6

Blinded Attending Surgeon Review						
Reviewer	PGT Estimate	H1 meets definition?	H2 meets definition?	AI meets definition?	Which is AI?	Which is most accurate?
1	10	Yes	Yes	Yes	H2	H2
2	<10	Yes	Yes	Yes	AI	AI
3	90	Yes	Yes	Yes	AI	H1

© 2021 Howell RS et al. *JAMA Network Open.*

Wound EMR Information								
Sequential Number	Gender	Age	Wound Type	Wound Location	Wound Length (cm)	Wound Width (cm)	Wound Depth (cm)	Wound Area (cm^2)
10	M	54	DFU	R met head	2.8	2.7	0.3	7.56

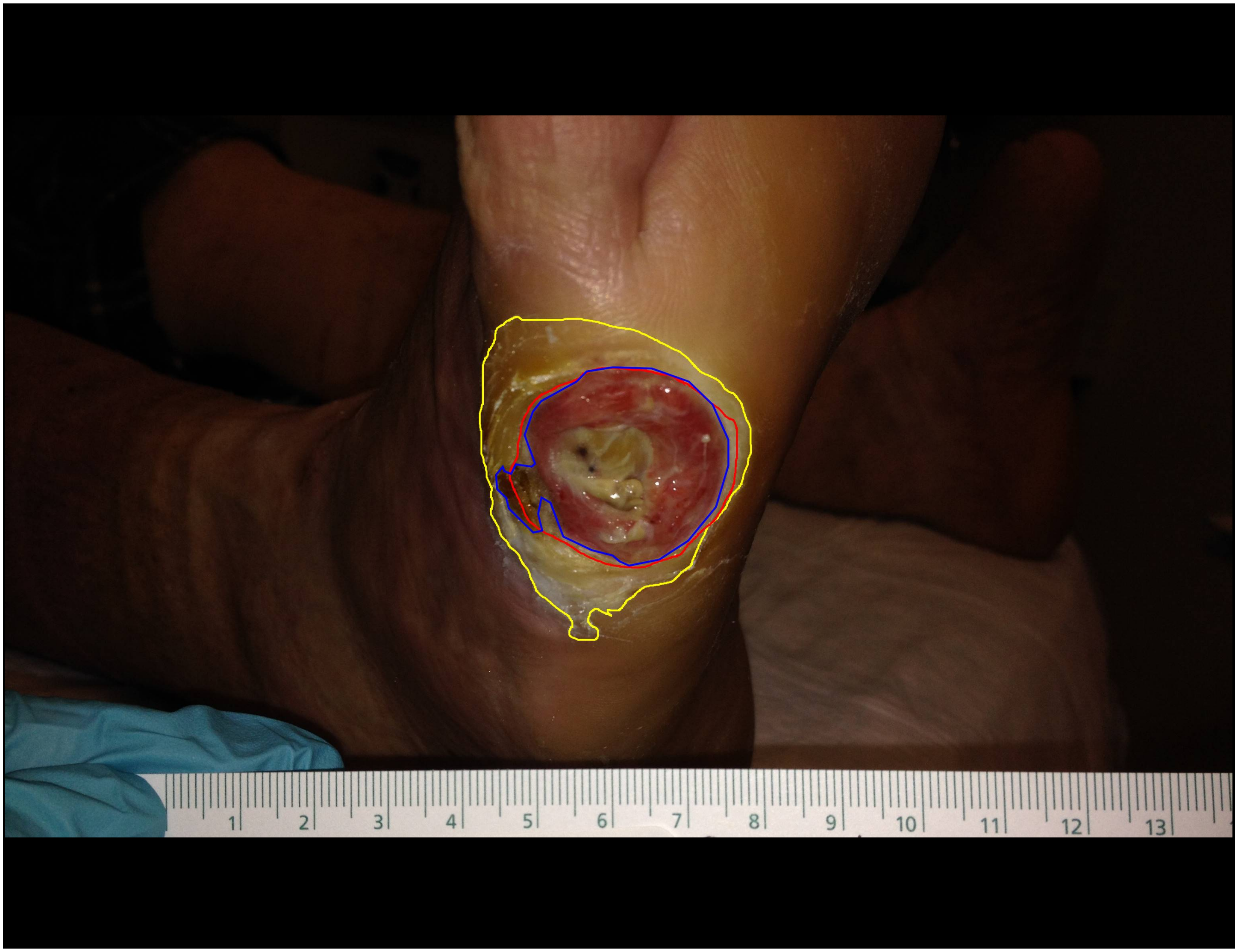

Tracing Data			
Tracer:	Wound Area (px^2)	Ruler Calibration (px/cm)	Wound Area (cm^2)
H1	430043	200.0	10.75
H2	244920	197.3	6.29
AI	227992	196.6	5.90

Tracing Comparisons				
Difference Metric:	Human-Human		Human-AI	
	H1(ref) H2(test)	H2(ref) H1(test)	H1(ref) AI(test)	H2(ref) AI(test)
False Negative Area (%)	43.0	0.0	47.0	9.6
False Positive Area (%)	0.0	75.6	0.0	2.7
|Relative Error (%)|	43.0	75.6	47.0	6.9

Blinded Attending Surgeon Review						
Reviewer	PGT Estimate	H1 meets definition?	H2 meets definition?	AI meets definition?	Which is AI?	Which is most accurate?
1	60	No	No	No	H2	0
2	50	No	No	No	H2	AI
3	40	No	Yes	Yes	H2	H1

© 2021 Howell RS et al. *JAMA Network Open.*

Wound EMR Information								
Sequential Number	Gender	Age	Wound Type	Wound Location	Wound Length (cm)	Wound Width (cm)	Wound Depth (cm)	Wound Area (cm^2)
11	F	89	PU	sacrum	5.0	3.3	2.2	16.50

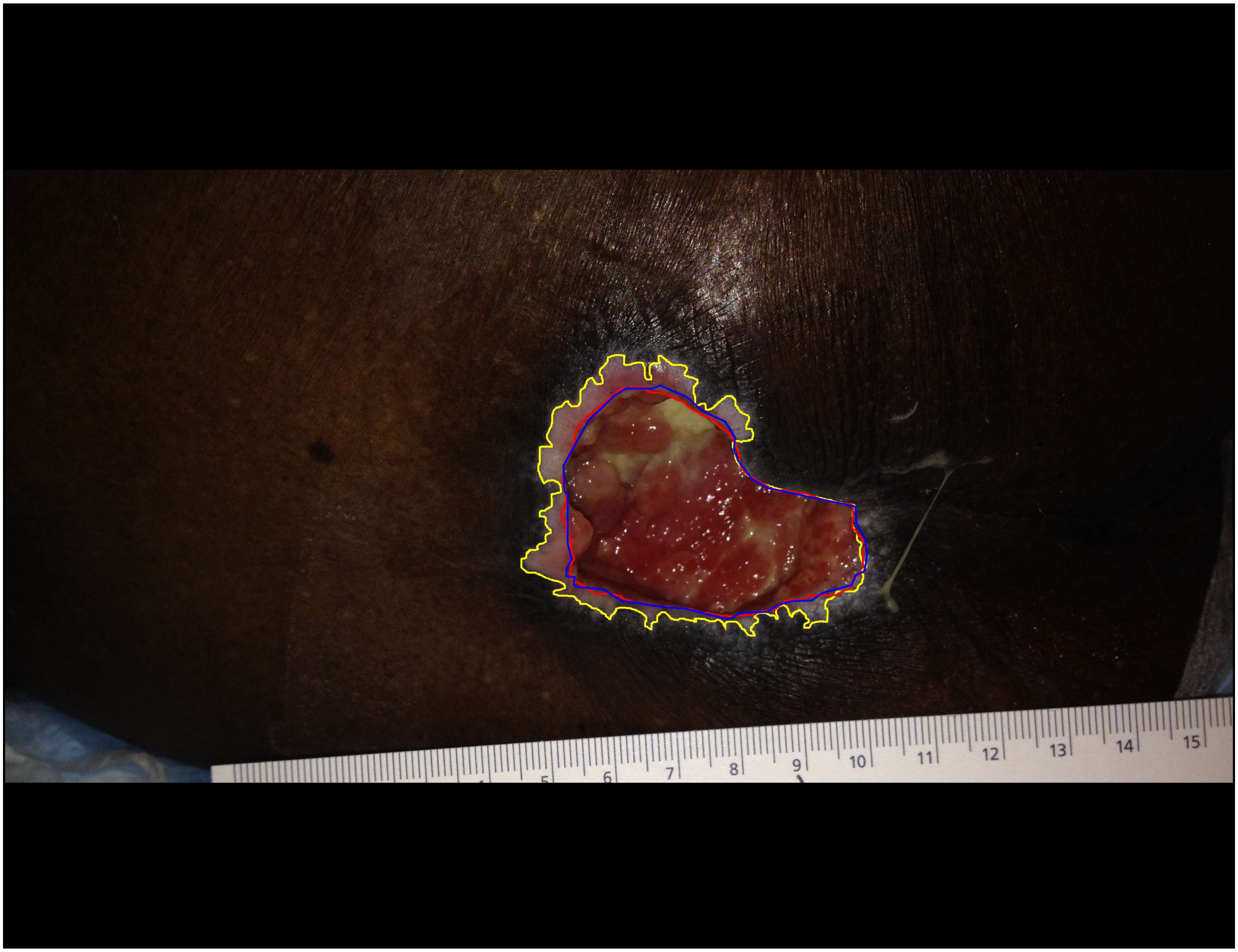

Tracing Data			
Tracer:	Wound Area (px^2)	Ruler Calibration (px/cm)	Wound Area (cm^2)
H1	416865	167.1	14.93
H2	327385	172.4	11.01
AI	329718	166.5	11.90

Tracing Comparisons				
Difference Metric:	Human-Human		Human-AI	
	H1(ref) H2(test)	H2(ref) H1(test)	H1(ref) AI(test)	H2(ref) AI(test)
False Negative Area (%)	21.9	0.5	21.5	1.7
False Positive Area (%)	0.4	27.9	0.6	2.4
|Relative Error (%)|	21.5	27.3	20.9	0.7

Blinded Attending Surgeon Review						
Reviewer	PGT Estimate	H1 meets definition?	H2 meets definition?	AI meets definition?	Which is AI?	Which is most accurate?
1	80	No	Yes	Yes	H1	AI
2	50	No	Yes	Yes	H1	AI
3	80	No	Yes	Yes	H2	AI

© 2021 Howell RS et al. *JAMA Network Open.*

Wound EMR Information								
Sequential Number	Gender	Age	Wound Type	Wound Location	Wound Length (cm)	Wound Width (cm)	Wound Depth (cm)	Wound Area (cm^2)
12	F	50	Trauma	R med thigh	8.5	1.7	0.1	14.45

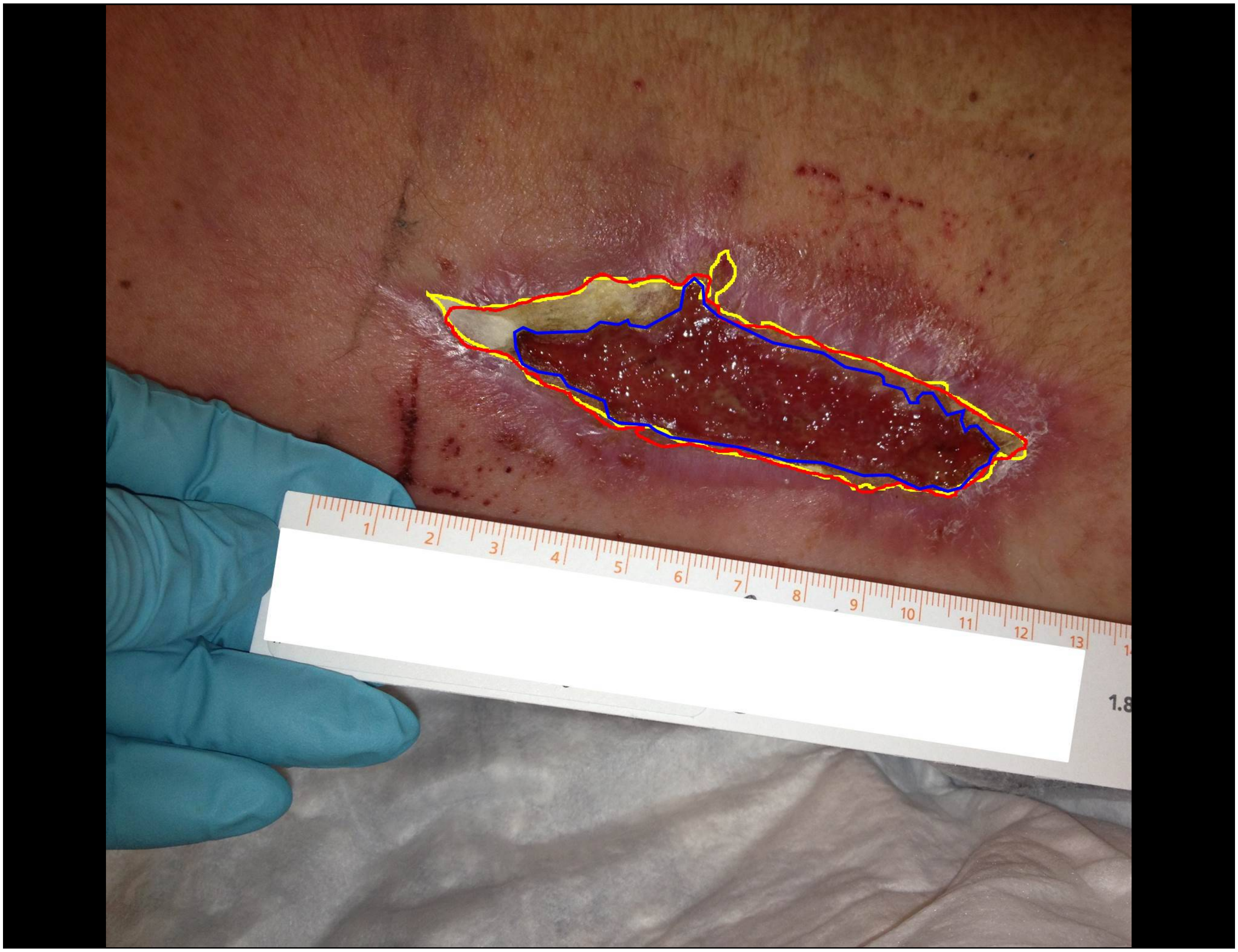

Tracing Data			
Tracer:	Wound Area (px^2)	Ruler Calibration (px/cm)	Wound Area (cm^2)
H1	170100	98.4	17.56
H2	168918	98.9	17.28
AI	120864	104.8	11.00

Tracing Comparisons				
Difference Metric:	Human-Human		Human-AI	
	H1(ref) H2(test)	H2(ref) H1(test)	H1(ref) AI(test)	H2(ref) AI(test)
False Negative Area (%)	4.1	3.5	29.1	28.4
False Positive Area (%)	3.4	4.1	0.1	0.0
|Relative Error (%)|	0.7	0.7	28.9	28.4

Blinded Attending Surgeon Review						
Reviewer	PGT Estimate	H1 meets definition?	H2 meets definition?	AI meets definition?	Which is AI?	Which is most accurate?
1	80	No	No	Yes	AI	H2
2	90	No	No	Yes	H2	H2
3	90	Yes	Yes	No	H2	AI

© 2021 Howell RS et al. *JAMA Network Open.*

Wound EMR Information								
Sequential Number	Gender	Age	Wound Type	Wound Location	Wound Length (cm)	Wound Width (cm)	Wound Depth (cm)	Wound Area (cm^2)
13	F	61	PU	sacrum	3.5	7.0	1.5	24.50

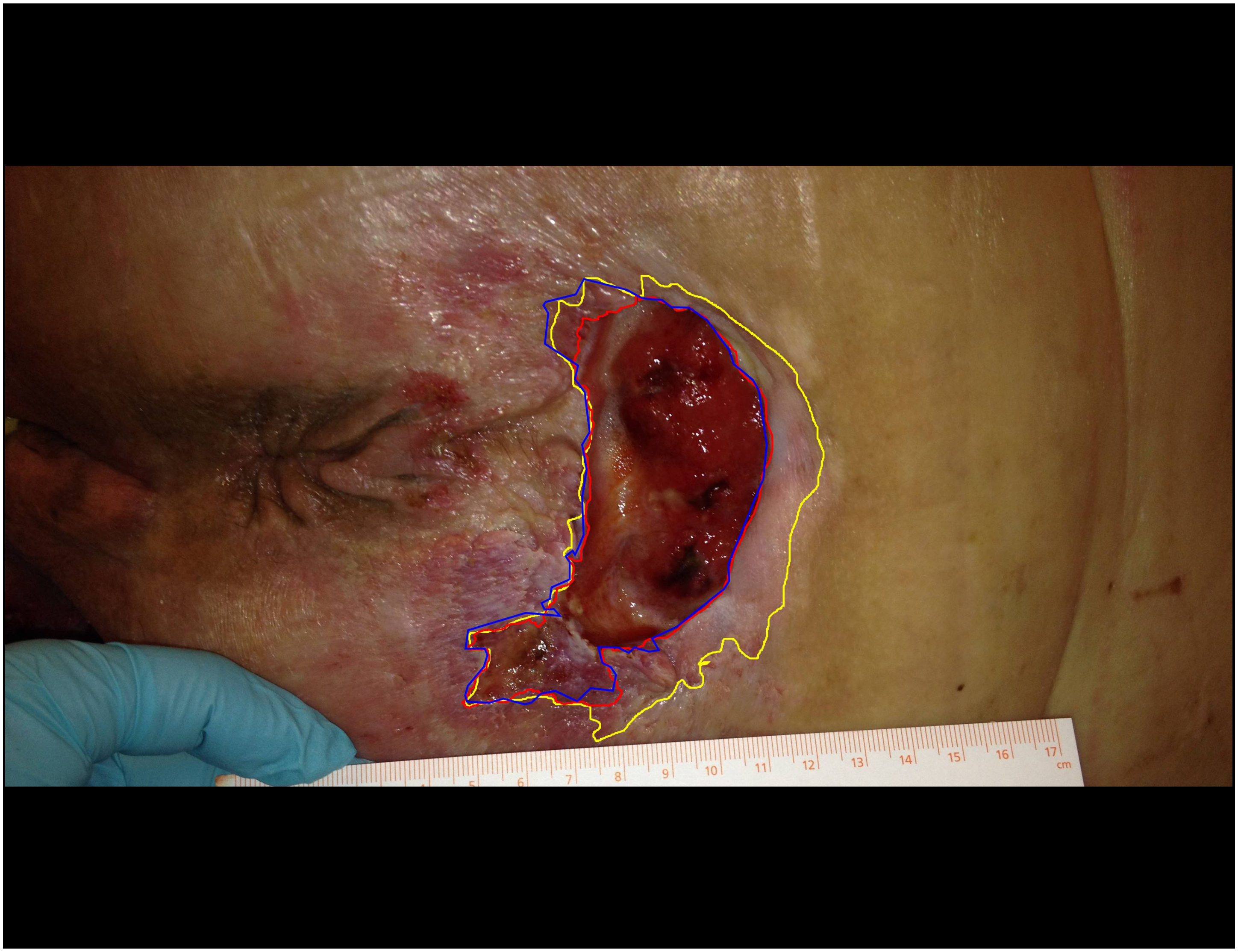

Tracing Data			
Tracer:	Wound Area (px^2)	Ruler Calibration (px/cm)	Wound Area (cm^2)
H1	653689	128.3	39.69
H2	442910	130.9	25.84
AI	469787	129.5	28.00

Tracing Comparisons				
Difference Metric:	Human-Human		Human-AI	
	H1(ref) H2(test)	H2(ref) H1(test)	H1(ref) AI(test)	H2(ref) AI(test)
False Negative Area (%)	32.5	0.4	30.1	3.2
False Positive Area (%)	0.3	48.0	1.9	9.3
|Relative Error (%)|	32.2	47.6	28.1	6.1

Blinded Attending Surgeon Review						
Reviewer	PGT Estimate	H1 meets definition?	H2 meets definition?	AI meets definition?	Which is AI?	Which is most accurate?
1	80	No	No	No	AI	AI
2	50	No	Yes	No	H1	AI
3	60	No	Yes	Yes	H2	AI

© 2021 Howell RS et al. *JAMA Network Open.*

Wound EMR Information								
Sequential Number	Gender	Age	Wound Type	Wound Location	Wound Length (cm)	Wound Width (cm)	Wound Depth (cm)	Wound Area (cm^2)
14	F	78	VLU	LLE ankle	1.9	1.5	0.1	2.85

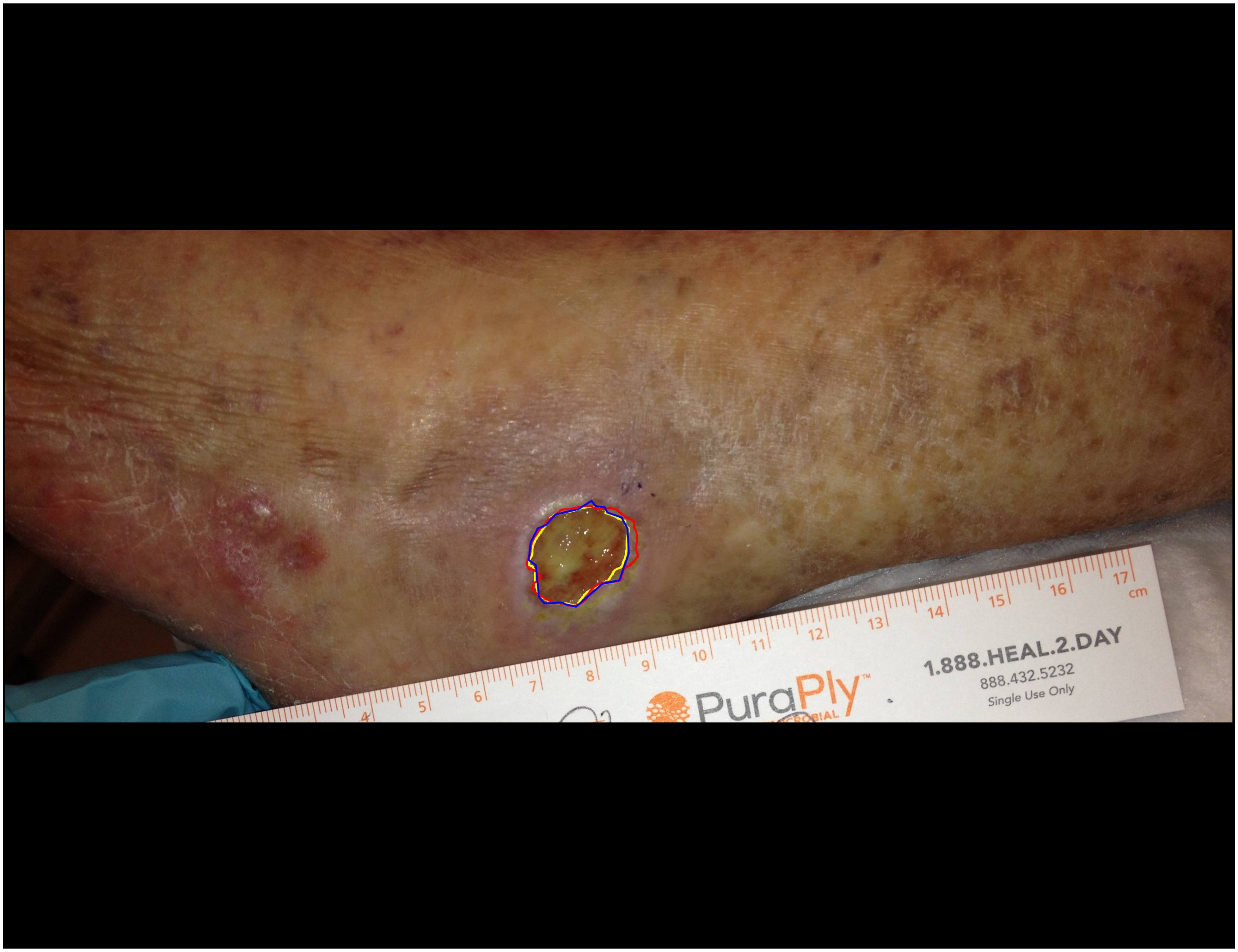

Tracing Data			
Tracer:	Wound Area (px^2)	Ruler Calibration (px/cm)	Wound Area (cm^2)
H1	49629	149.5	2.22
H2	56296	154.1	2.37
AI	52912	148.5	2.40

Tracing Comparisons				
Difference Metric:	Human-Human		Human-AI	
	H1(ref) H2(test)	H2(ref) H1(test)	H1(ref) AI(test)	H2(ref) AI(test)
False Negative Area (%)	1.1	12.8	1.5	8.6
False Positive Area (%)	14.5	1.0	8.1	2.6
|Relative Error (%)|	13.4	11.8	6.6	6.0

Blinded Attending Surgeon Review						
Reviewer	PGT Estimate	H1 meets definition?	H2 meets definition?	AI meets definition?	Which is AI?	Which is most accurate?
1	20	Yes	No	Yes	H1	H1
2	0	Yes	No	Yes	H1	H1
3	80	Yes	No	No	H2	H1

© 2021 Howell RS et al. *JAMA Network Open.*

Wound EMR Information								
Sequential Number	Gender	Age	Wound Type	Wound Location	Wound Length (cm)	Wound Width (cm)	Wound Depth (cm)	Wound Area (cm^2)
15	F	85	Arterial	LLE medial	1.1	0.8	0.1	0.88

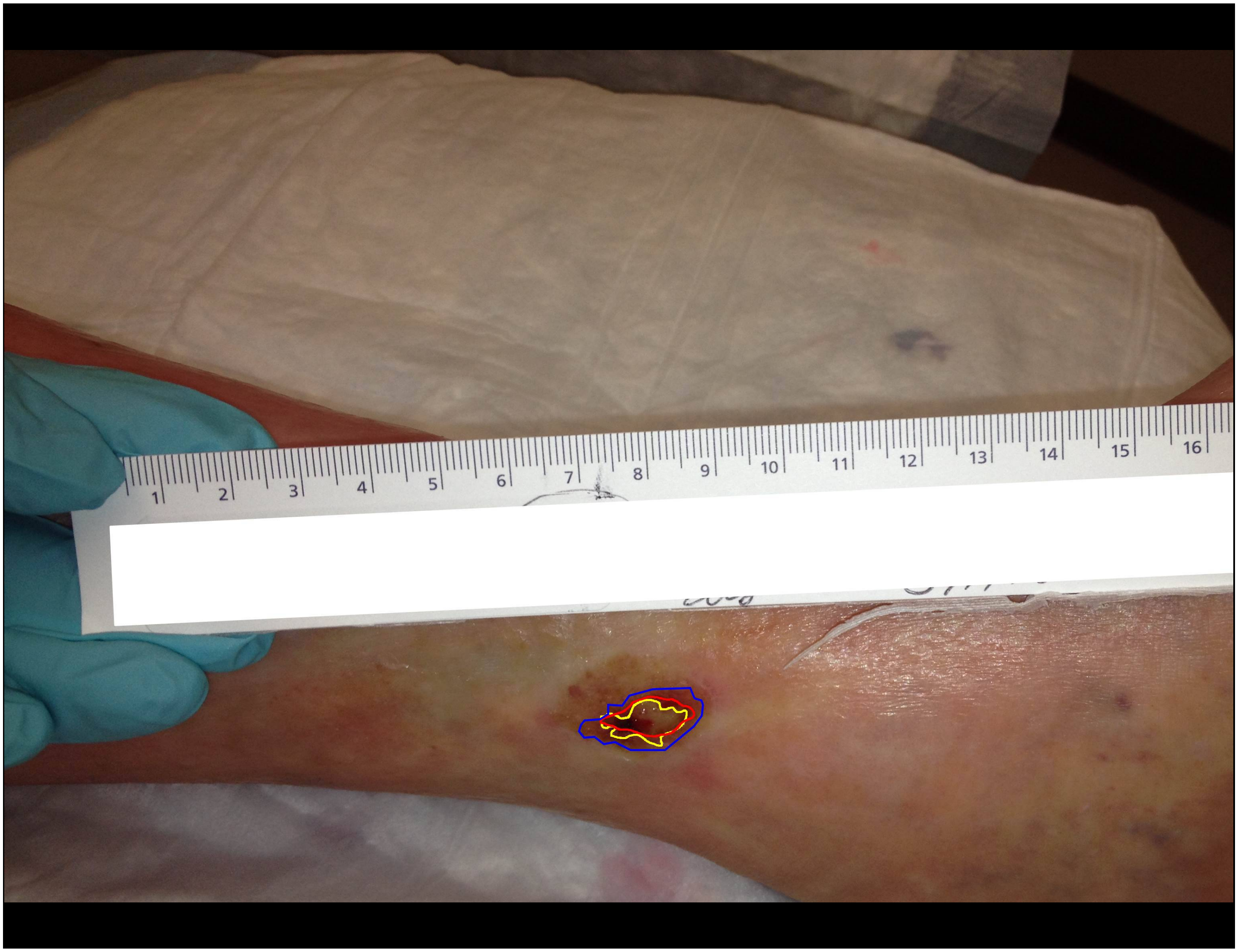

Tracing Data			
Tracer:	Wound Area (px^2)	Ruler Calibration (px/cm)	Wound Area (cm^2)
H1	13374	172.4	0.45
H2	14161	172.1	0.48
AI	31899	163.0	1.20

Tracing Comparisons				
Difference Metric:	Human-Human		Human-AI	
	H1(ref) H2(test)	H2(ref) H1(test)	H1(ref) AI(test)	H2(ref) AI(test)
False Negative Area (%)	18.0	22.5	0.3	0.1
False Positive Area (%)	23.9	17.0	138.8	125.3
|Relative Error (%)|	5.9	5.6	138.5	125.3

Blinded Attending Surgeon Review						
Reviewer	PGT Estimate	H1 meets definition?	H2 meets definition?	AI meets definition?	Which is AI?	Which is most accurate?
1		Yes	Yes	Yes	0	0
2	0	No	Yes	No	AI	H2
3	20	No	Yes	No	AI	H1

© 2021 Howell RS et al. *JAMA Network Open.*

Wound EMR Information								
Sequential Number	Gender	Age	Wound Type	Wound Location	Wound Length (cm)	Wound Width (cm)	Wound Depth (cm)	Wound Area (cm^2)
16	F	80	Trauma	RLE lat	7.0	3.3	0.1	23.10

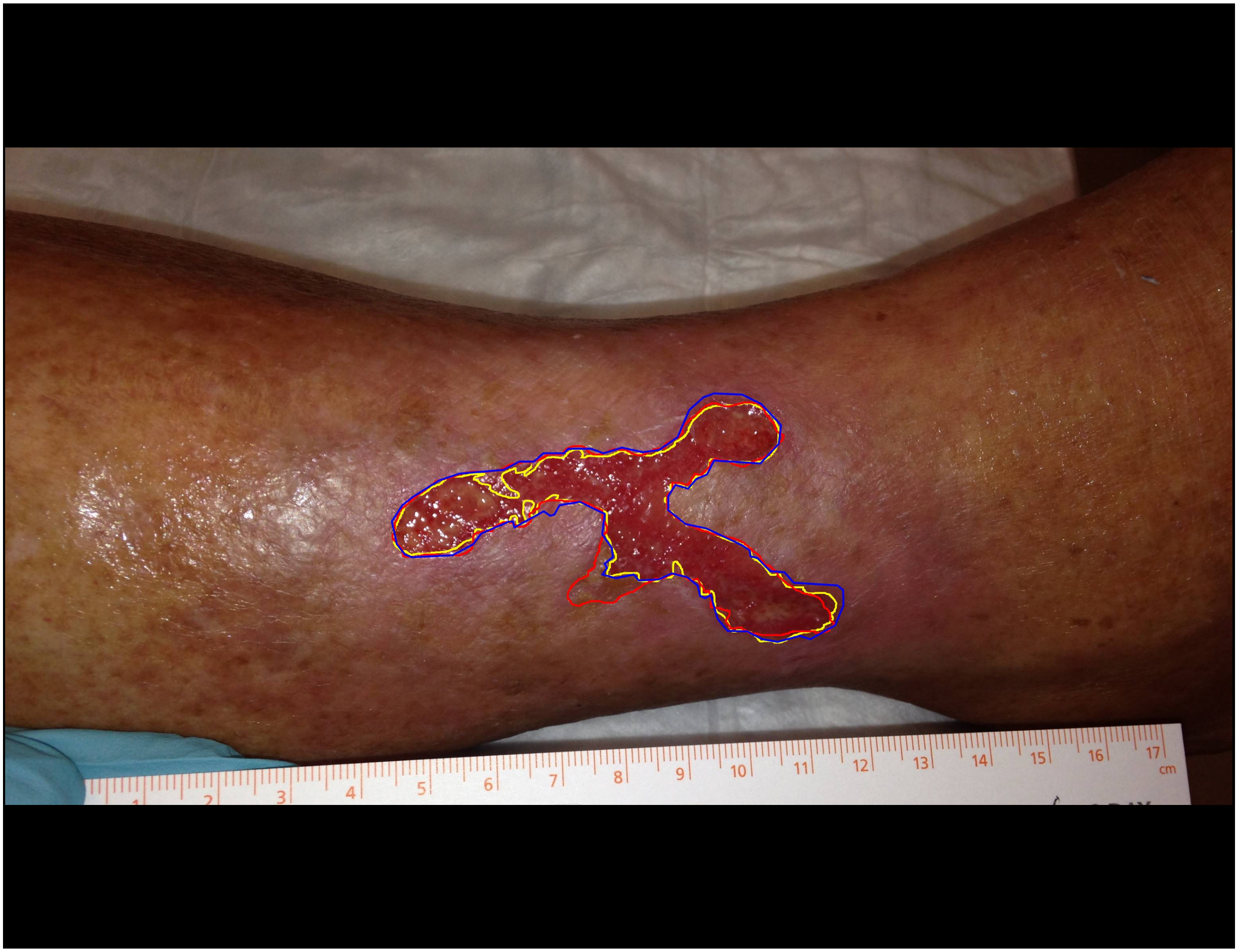

Tracing Data			
Tracer:	Wound Area (px^2)	Ruler Calibration (px/cm)	Wound Area (cm^2)
H1	238779	160.1	9.32
H2	268678	166.2	9.73
AI	276343	183.6	8.20

Tracing Comparisons				
Difference Metric:	Human-Human		Human-AI	
	H1(ref) H2(test)	H2(ref) H1(test)	H1(ref) AI(test)	H2(ref) AI(test)
False Negative Area (%)	3.4	14.2	0.6	7.0
False Positive Area (%)	16.0	3.1	16.3	9.8
|Relative Error (%)|	12.5	11.1	15.7	2.9

Blinded Attending Surgeon Review						
Reviewer	PGT Estimate	H1 meets definition?	H2 meets definition?	AI meets definition?	Which is AI?	Which is most accurate?
1	90	No	Yes	Yes	H2	AI
2	75	Yes	No	Yes	H2	H1
3	40	Yes	No	Yes	H2	AI

© 2021 Howell RS et al. *JAMA Network Open.*

Wound EMR Information								
Sequential Number	Gender	Age	Wound Type	Wound Location	Wound Length (cm)	Wound Width (cm)	Wound Depth (cm)	Wound Area (cm^2)
17	F	69	PU	sacrum	3.0	1.2	2.4	3.60

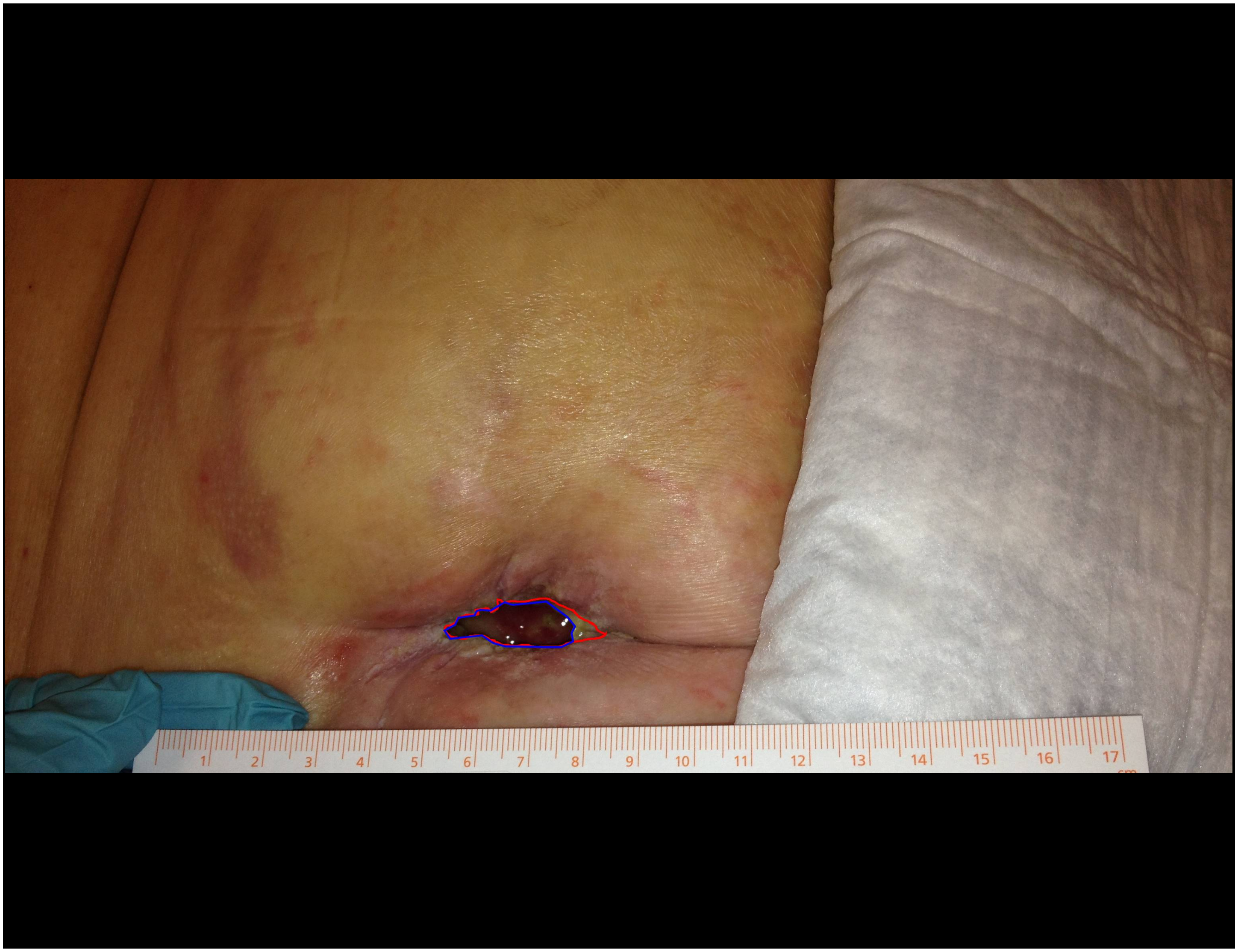

Tracing Data			
Tracer:	Wound Area (px^2)	Ruler Calibration (px/cm)	Wound Area (cm^2)
H1			1.63
H2	35083	142.0	1.74
AI	29478	135.7	1.60

Tracing Comparisons				
Difference Metric:	Human-Human		Human-AI	
	H1(ref) H2(test)	H2(ref) H1(test)	H1(ref) AI(test)	H2(ref) AI(test)
False Negative Area (%)				19.0
False Positive Area (%)				3.1
|Relative Error (%)|				16.0

Blinded Attending Surgeon Review						
Reviewer	PGT Estimate	H1 meets definition?	H2 meets definition?	AI meets definition?	Which is AI?	Which is most accurate?
1		Yes	Yes	No	H1	AI
2	0	No	No	Yes	H2	H2
3	60	Yes	Yes	No	H2	H2

© 2021 Howell RS et al. *JAMA Network Open.*

Wound EMR Information								
Sequential Number	Gender	Age	Wound Type	Wound Location	Wound Length (cm)	Wound Width (cm)	Wound Depth (cm)	Wound Area (cm^2)
18	F	86	Nec Fasc	LLE lat	0.4	0.4	0.2	0.16

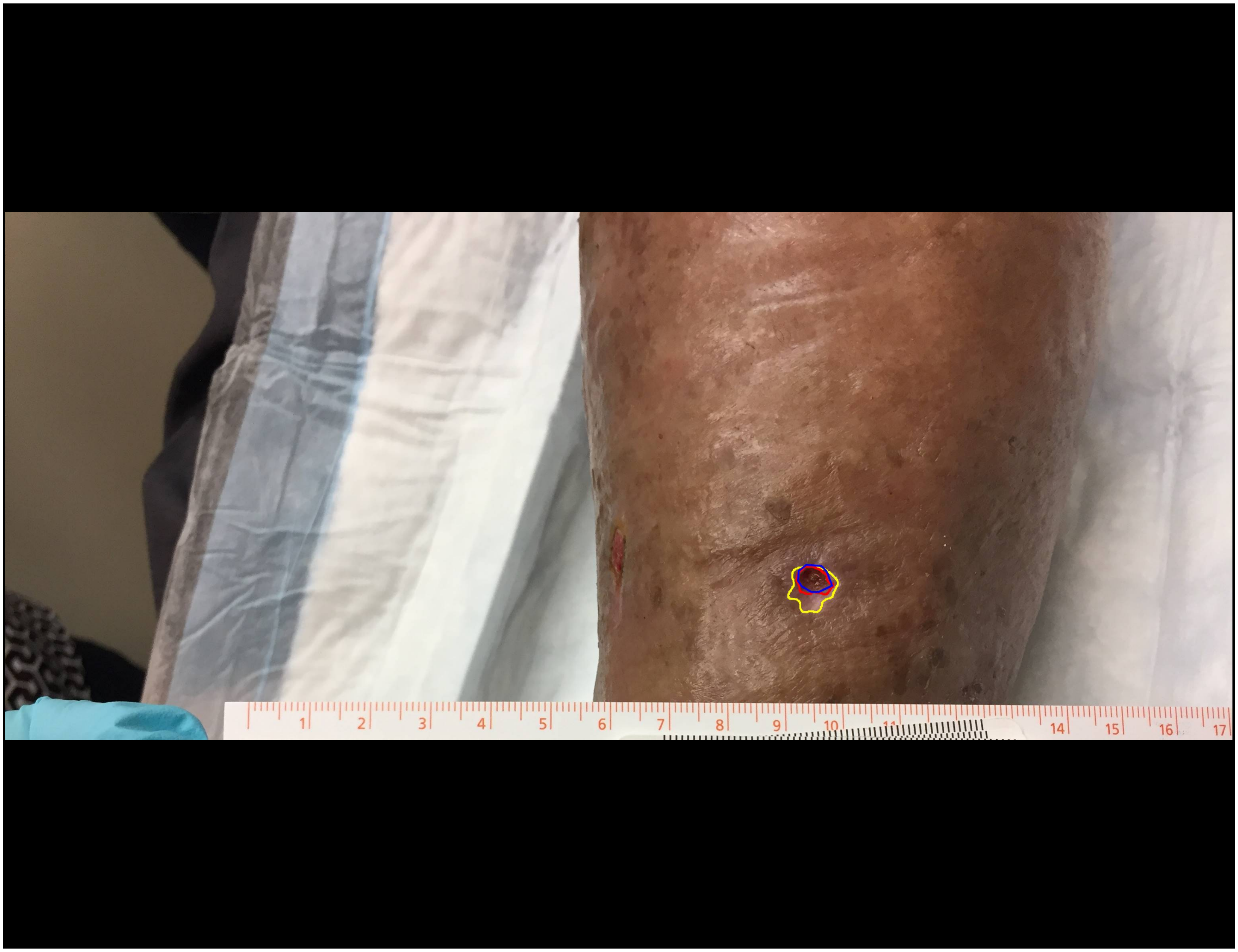

Tracing Data			
Tracer:	Wound Area (px^2)	Ruler Calibration (px/cm)	Wound Area (cm^2)
H1	11232	151.4	0.49
H2	5139	153.2	0.22
AI	4914	156.7	0.20

Tracing Comparisons				
Difference Metric:	Human-Human		Human-AI	
	H1(ref) H2(test)	H2(ref) H1(test)	H1(ref) AI(test)	H2(ref) AI(test)
False Negative Area (%)	54.3	0.1	57.6	17.1
False Positive Area (%)	0.1	118.7	1.3	12.7
|Relative Error (%)|	54.2	118.6	56.3	4.4

Blinded Attending Surgeon Review						
Reviewer	PGT Estimate	H1 meets definition?	H2 meets definition?	AI meets definition?	Which is AI?	Which is most accurate?
1	100	No	Yes	Yes	AI	AI
2	0	No	Yes	Yes	AI	H2
3	0	No	Yes	Yes	AI	H2

© 2021 Howell RS et al. *JAMA Network Open.*

Wound EMR Information								
Sequential Number	Gender	Age	Wound Type	Wound Location	Wound Length (cm)	Wound Width (cm)	Wound Depth (cm)	Wound Area (cm^2)
19	F	83	Trauma	R knee	0.9	1.0		0.90

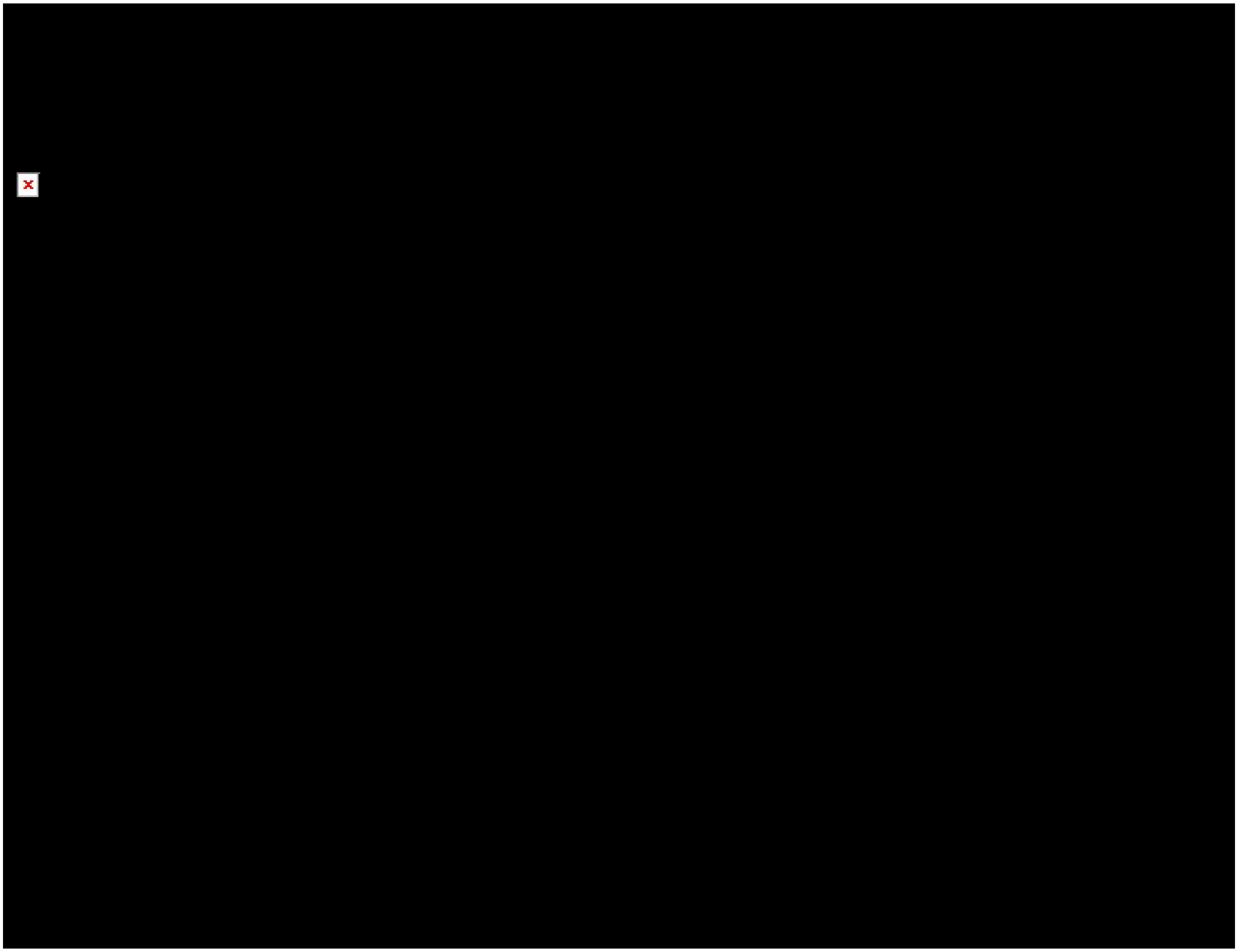

Tracing Data			
Tracer:	Wound Area (px^2)	Ruler Calibration (px/cm)	Wound Area (cm^2)
H1	19330	160.5	0.75
H2	2300	159.9	0.09
AI	12292	175.3	0.40

Tracing Comparisons				
Difference Metric:	Human-Human		Human-AI	
	H1(ref) H2(test)	H2(ref) H1(test)	H1(ref) AI(test)	H2(ref) AI(test)
False Negative Area (%)	89.5	11.6	44.5	24.8
False Positive Area (%)	1.4	752.0	8.1	459.2
|Relative Error (%)|	88.1	740.4	36.4	434.4

Blinded Attending Surgeon Review						
Reviewer	PGT Estimate	H1 meets definition?	H2 meets definition?	AI meets definition?	Which is AI?	Which is most accurate?
1	healed	No	No	No	AI	None
2	0	No	No	Yes	AI	H1
3	10	No	No	Yes	H1	H1

© 2021 Howell RS et al. *JAMA Network Open.*

Wound EMR Information								
Sequential Number	Gender	Age	Wound Type	Wound Location	Wound Length (cm)	Wound Width (cm)	Wound Depth (cm)	Wound Area (cm^2)
20	F	72	VLU	LLE lat	7.0	6.8	0.4	47.60

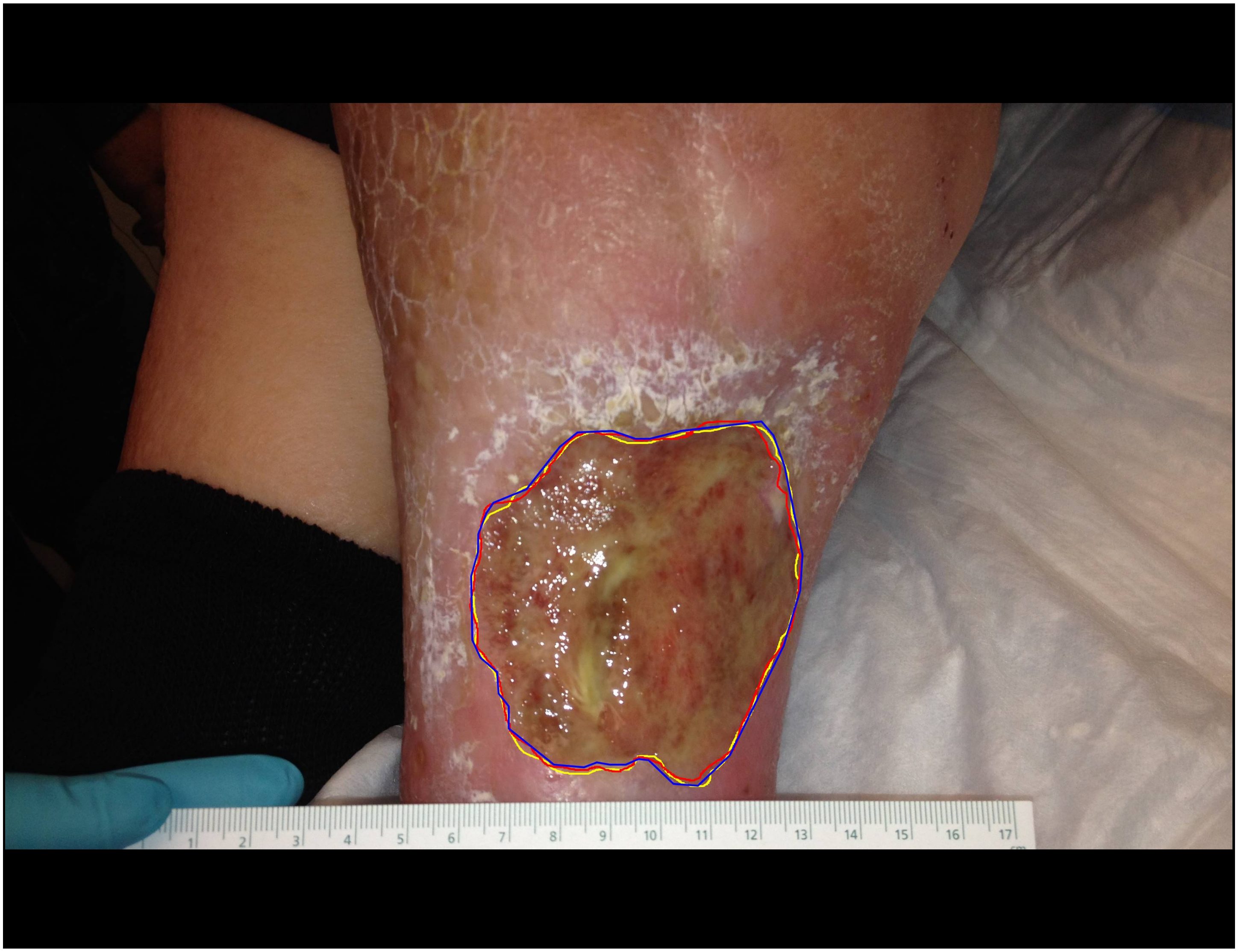

Tracing Data			
Tracer:	Wound Area (px^2)	Ruler Calibration (px/cm)	Wound Area (cm^2)
H1	649860	134.7	35.82
H2	643550	132.0	36.94
AI	659952	139.5	33.90

Tracing Comparisons				
Difference Metric:	Human-Human		Human-AI	
	H1(ref) H2(test)	H2(ref) H1(test)	H1(ref) AI(test)	H2(ref) AI(test)
False Negative Area (%)	2.3	1.4	1.1	0.9
False Positive Area (%)	1.4	2.4	2.6	3.4
|Relative Error (%)|	1.0	1.0	1.6	2.5

Blinded Attending Surgeon Review						
Reviewer	PGT Estimate	H1 meets definition?	H2 meets definition?	AI meets definition?	Which is AI?	Which is most accurate?
1	<10	Yes	Yes	Yes	AI	AI
2	0	Yes	No	No	AI	H2
3	30	Yes	Yes	Yes	H2	H1

© 2021 Howell RS et al. *JAMA Network Open.*

Wound EMR Information								
Sequential Number	Gender	Age	Wound Type	Wound Location	Wound Length (cm)	Wound Width (cm)	Wound Depth (cm)	Wound Area (cm^2)
21	F	73	PU	R ischium	1.6	7.2	2.4	11.52

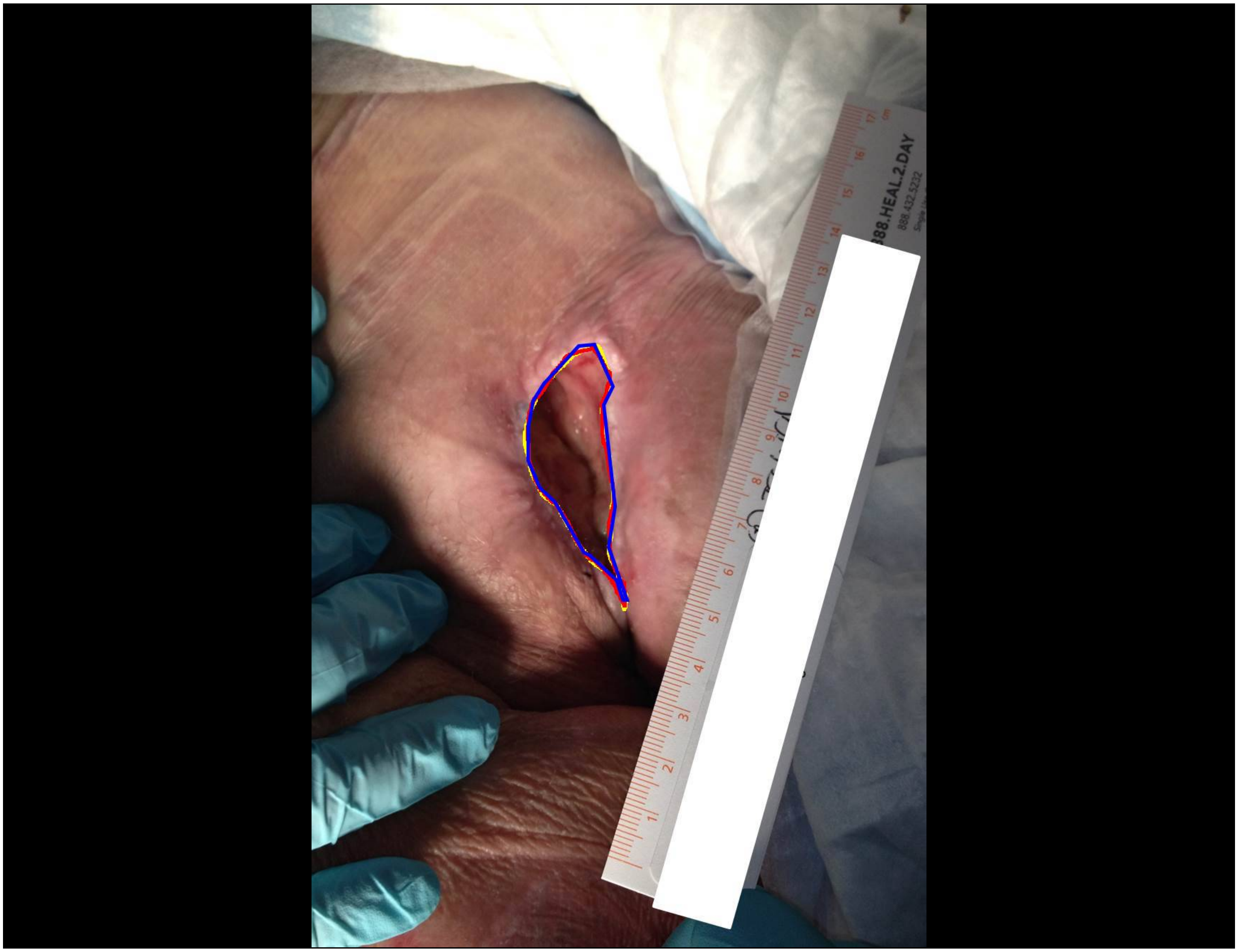

Tracing Data			
Tracer:	Wound Area (px^2)	Ruler Calibration (px/cm)	Wound Area (cm^2)
H1	25879	69.4	5.38
H2	25220	65.8	5.82
AI	25864	61.7	6.80

Tracing Comparisons				
Difference Metric:	Human-Human		Human-AI	
	H1(ref) H2(test)	H2(ref) H1(test)	H1(ref) AI(test)	H2(ref) AI(test)
False Negative Area (%)	4.1	1.6	3.1	2.1
False Positive Area (%)	1.6	4.2	3.0	4.7
|Relative Error (%)|	2.5	2.6	0.1	2.6

Blinded Attending Surgeon Review						
Reviewer	PGT Estimate	H1 meets definition?	H2 meets definition?	AI meets definition?	Which is AI?	Which is most accurate?
1	0	Yes	Yes	Yes	AI	H1
2	0	Yes	Yes	Yes	H2	H1
3	10	Yes	Yes	Yes	H2	H1

© 2021 Howell RS et al. *JAMA Network Open.*

Wound EMR Information								
Sequential Number	Gender	Age	Wound Type	Wound Location	Wound Length (cm)	Wound Width (cm)	Wound Depth (cm)	Wound Area (cm^2)
22	M	64	DFU	L 2nd toe	3.0	2.0	0.1	6.00

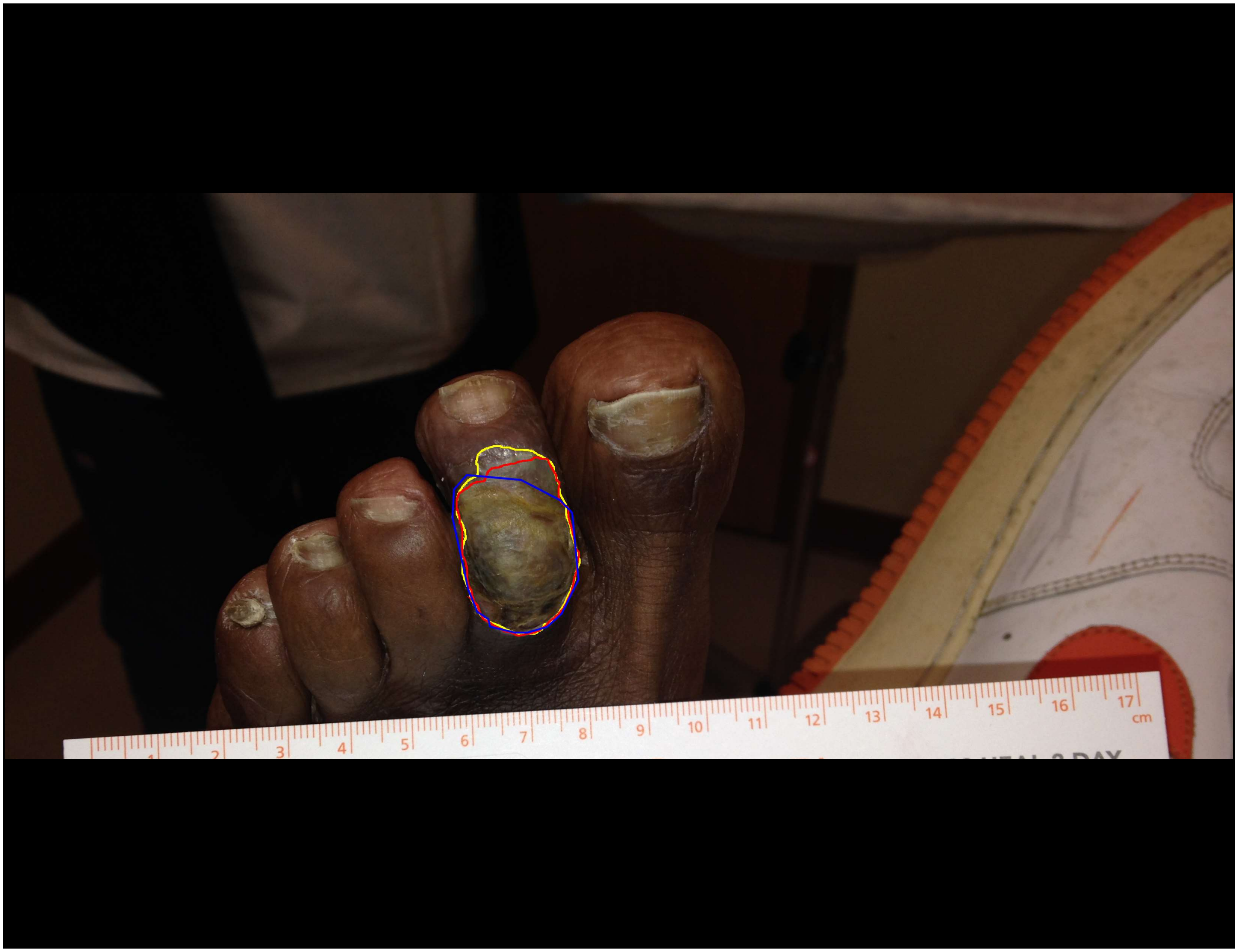

Tracing Data			
Tracer:	Wound Area (px^2)	Ruler Calibration (px/cm)	Wound Area (cm^2)
H1	122306	157.5	4.93
H2	113971	157.5	4.59
AI	109612	159.7	4.30

Tracing Comparisons				
Difference Metric:	Human-Human		Human-AI	
	H1(ref) H2(test)	H2(ref) H1(test)	H1(ref) AI(test)	H2(ref) AI(test)
False Negative Area (%)	8.2	1.4	15.2	9.5
False Positive Area (%)	1.3	8.8	4.8	5.7
|Relative Error (%)|	6.8	7.3	10.4	3.8

Blinded Attending Surgeon Review						
Reviewer	PGT Estimate	H1 meets definition?	H2 meets definition?	AI meets definition?	Which is AI?	Which is most accurate?
1	0	Yes	No	No	H1	AI
2	0	No	No	Yes	AI	H2
3	0	No	No	No	H2	H2

© 2021 Howell RS et al. *JAMA Network Open.*

Wound EMR Information								
**Sequential Number**	**Gender**	**Age**	**Wound Type**	**Wound Location**	**Wound Length (cm)**	**Wound Width (cm)**	**Wound Depth (cm)**	**Wound Area (cm^2)**
23	F	70	VLU	R med ankle	1.8	2.0	0.2	3.60

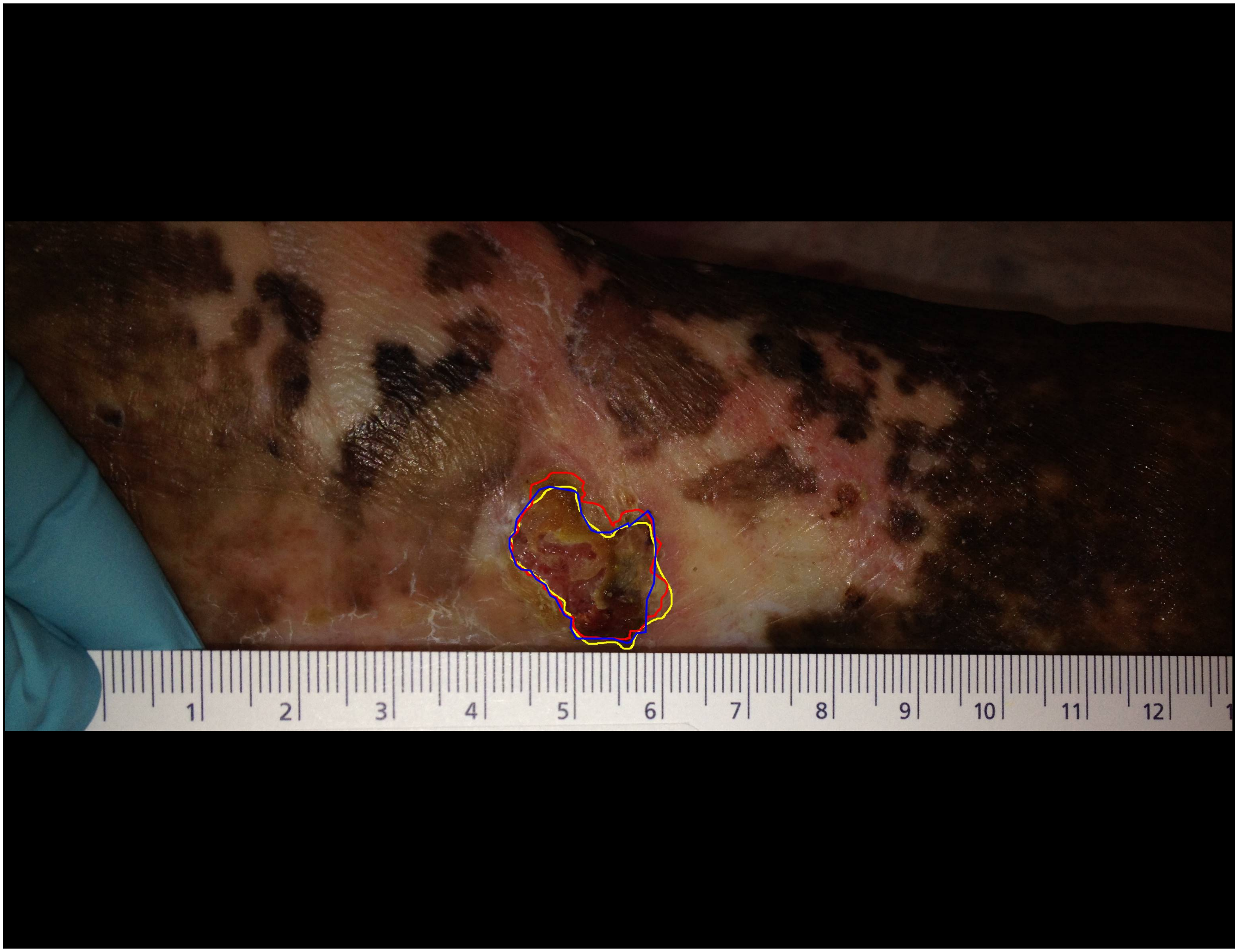

Tracing Data			
**Tracer:**	**Wound Area (px^2)**	**Ruler Calibration (px/cm)**	**Wound Area (cm^2)**
**H1**	106513	237.4	1.89
**H2**	116433	233.0	2.15
**AI**	103069	253.8	1.60

Tracing Comparisons				
**Difference Metric:**	**Human-Human**		**Human-AI**	
	**H1(ref) H2(test)**	**H2(ref) H1(test)**	**H1(ref) AI(test)**	**H2(ref) AI(test)**
**False Negative Area (%)**	5.8	13.8	8.4	14.4
**False Positive Area (%)**	15.1	5.3	5.2	3.0
**|Relative Error (%)|**	9.3	8.5	3.2	11.5

Blinded Attending Surgeon Review						
**Reviewer**	**PGT Estimate**	**H1 meets definition?**	**H2 meets definition?**	**AI meets definition?**	**Which is AI?**	**Which is most accurate?**
**1**	90	No	No	No	AI	0
**2**	30	No	No	No	H1	H2
**3**	30	Yes	Yes	Yes	H1	AI

© 2021 Howell RS et al. *JAMA Network Open.*

Wound EMR Information								
Sequential Number	Gender	Age	Wound Type	Wound Location	Wound Length (cm)	Wound Width (cm)	Wound Depth (cm)	Wound Area (cm^2)
24	M	92	VLU	LLE lat	8.6	7.5	0.2	64.50

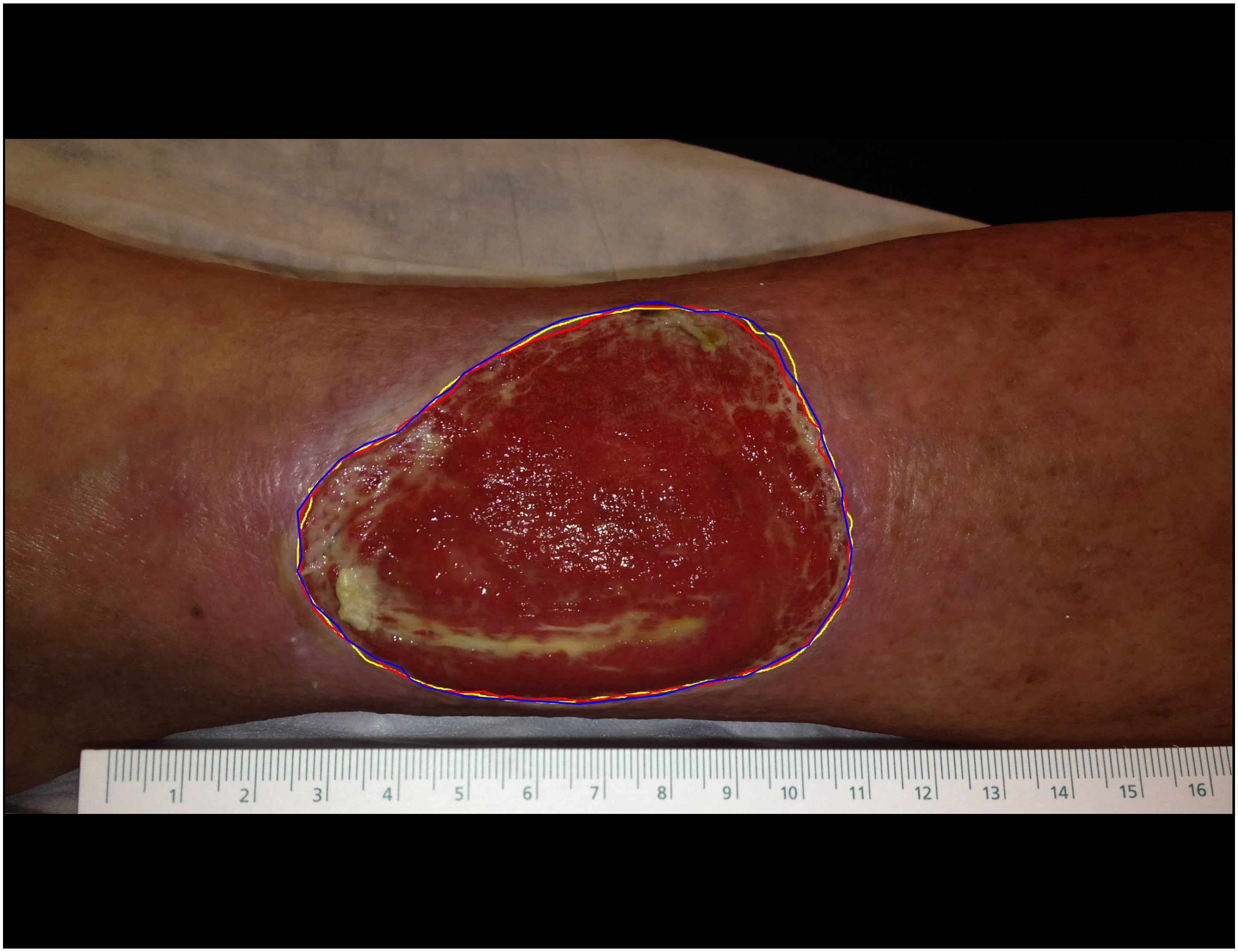

Tracing Data			
Tracer:	Wound Area (px^2)	Ruler Calibration (px/cm)	Wound Area (cm^2)
H1	1173731	182.7	35.18
H2	1157291	178.0	36.53
AI	1176966	192.4	31.80

Tracing Comparisons				
Difference Metric:	Human-Human		Human-AI	
	H1(ref) H2(test)	H2(ref) H1(test)	H1(ref) AI(test)	H2(ref) AI(test)
False Negative Area (%)	2.0	0.6	1.0	0.6
False Positive Area (%)	0.6	2.0	1.3	2.3
|Relative Error (%)|	1.4	1.4	0.3	1.7

Blinded Attending Surgeon Review						
Reviewer	PGT Estimate	H1 meets definition?	H2 meets definition?	AI meets definition?	Which is AI?	Which is most accurate?
1	90	Yes	Yes	Yes	H1	AI
2	80	Yes	Yes	Yes	AI	H2
3	100	Yes	Yes	Yes	AI	H1

© 2021 Howell RS et al. *JAMA Network Open.*

Wound EMR Information								
Sequential Number	Gender	Age	Wound Type	Wound Location	Wound Length (cm)	Wound Width (cm)	Wound Depth (cm)	Wound Area (cm^2)
25	M	53	DFU	L plantar	1.8	1.7	0.2	3.06

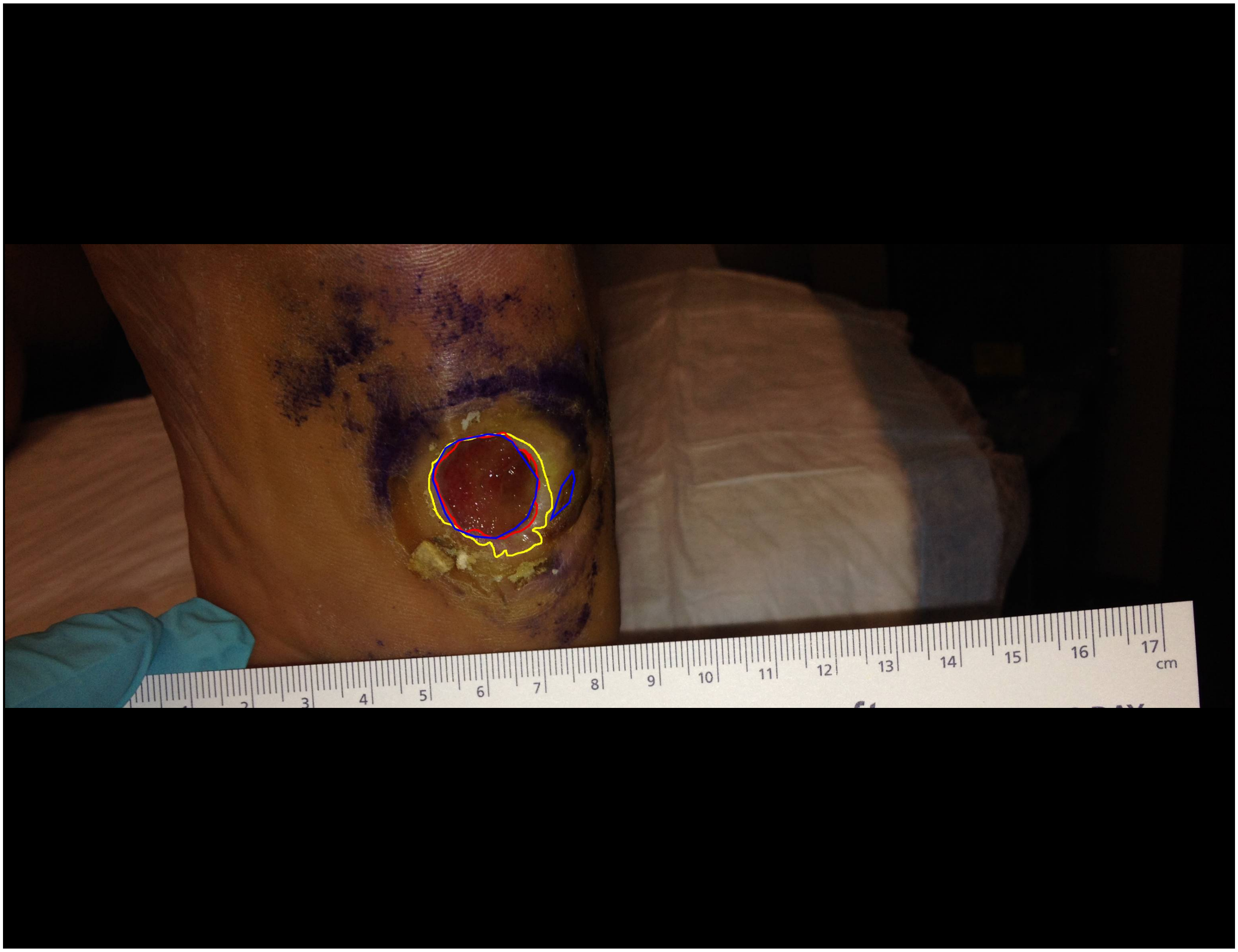

Tracing Data			
Tracer:	Wound Area (px^2)	Ruler Calibration (px/cm)	Wound Area (cm^2)
H1	78726	151.5	3.43
H2	58405	152.3	2.52
AI	60611	155.7	2.50

Tracing Comparisons				
Difference Metric:	Human-Human		Human-AI	
	H1(ref) H2(test)	H2(ref) H1(test)	H1(ref) AI(test)	H2(ref) AI(test)
False Negative Area (%)	26.0	0.3	26.8	6.1
False Positive Area (%)	0.2	35.1	3.8	9.9
|Relative Error (%)|	25.8	34.8	23.0	3.8

Blinded Attending Surgeon Review						
Reviewer	PGT Estimate	H1 meets definition?	H2 meets definition?	AI meets definition?	Which is AI?	Which is most accurate?
1	100	No	Yes	Yes	H1	H1
2	50	No	Yes	No	H2	H1
3	90	No	Yes	No	H2	H1

© 2021 Howell RS et al. *JAMA Network Open.*

Wound EMR Information								
Sequential Number	Gender	Age	Wound Type	Wound Location	Wound Length (cm)	Wound Width (cm)	Wound Depth (cm)	Wound Area (cm^2)
26	M	49	DFU	L plantar	0.2	0.1	0.1	0.02

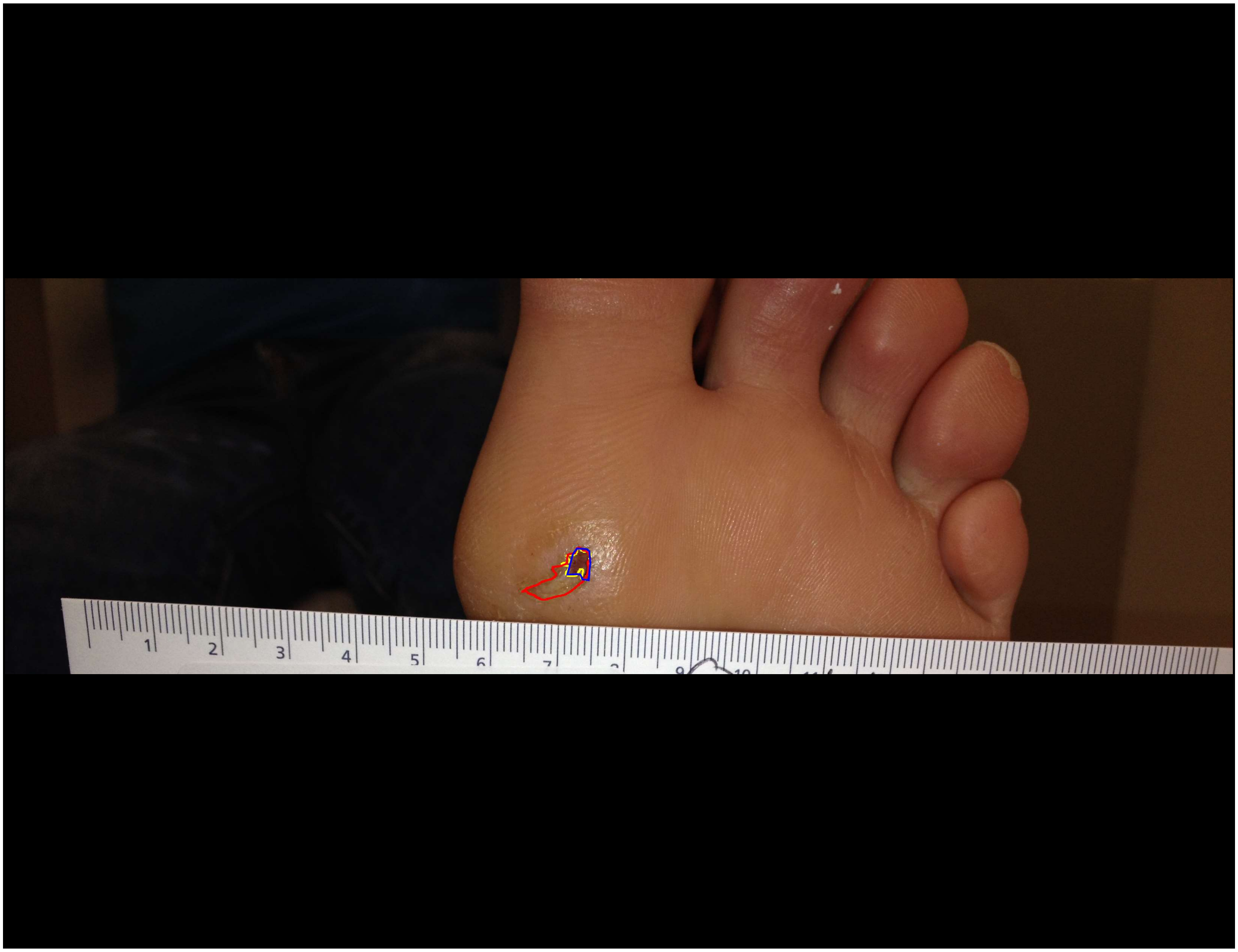

Tracing Data			
Tracer:	Wound Area (px^2)	Ruler Calibration (px/cm)	Wound Area (cm^2)
H1	3623	181.5	0.11
H2	10654	178.3	0.34
AI	3694	192.2	0.10

Tracing Comparisons				
Difference Metric:	Human-Human		Human-AI	
	H1(ref) H2(test)	H2(ref) H1(test)	H1(ref) AI(test)	H2(ref) AI(test)
False Negative Area (%)	6.1	68.1	16.0	70.0
False Positive Area (%)	200.2	2.1	17.9	4.7
|Relative Error (%)|	194.1	66.0	2.0	65.3

Blinded Attending Surgeon Review						
Reviewer	PGT Estimate	H1 meets definition?	H2 meets definition?	AI meets definition?	Which is AI?	Which is most accurate?
1	0	No	Yes	No	AI	H2
2		Yes	No	Yes	AI	0
3	30	Yes	No	Yes	H1	AI

© 2021 Howell RS et al. *JAMA Network Open.*

Wound EMR Information								
Sequential Number	Gender	Age	Wound Type	Wound Location	Wound Length (cm)	Wound Width (cm)	Wound Depth (cm)	Wound Area (cm^2)
27	F	84	VLU	LLE medial	5.1	5.0	0.2	25.50

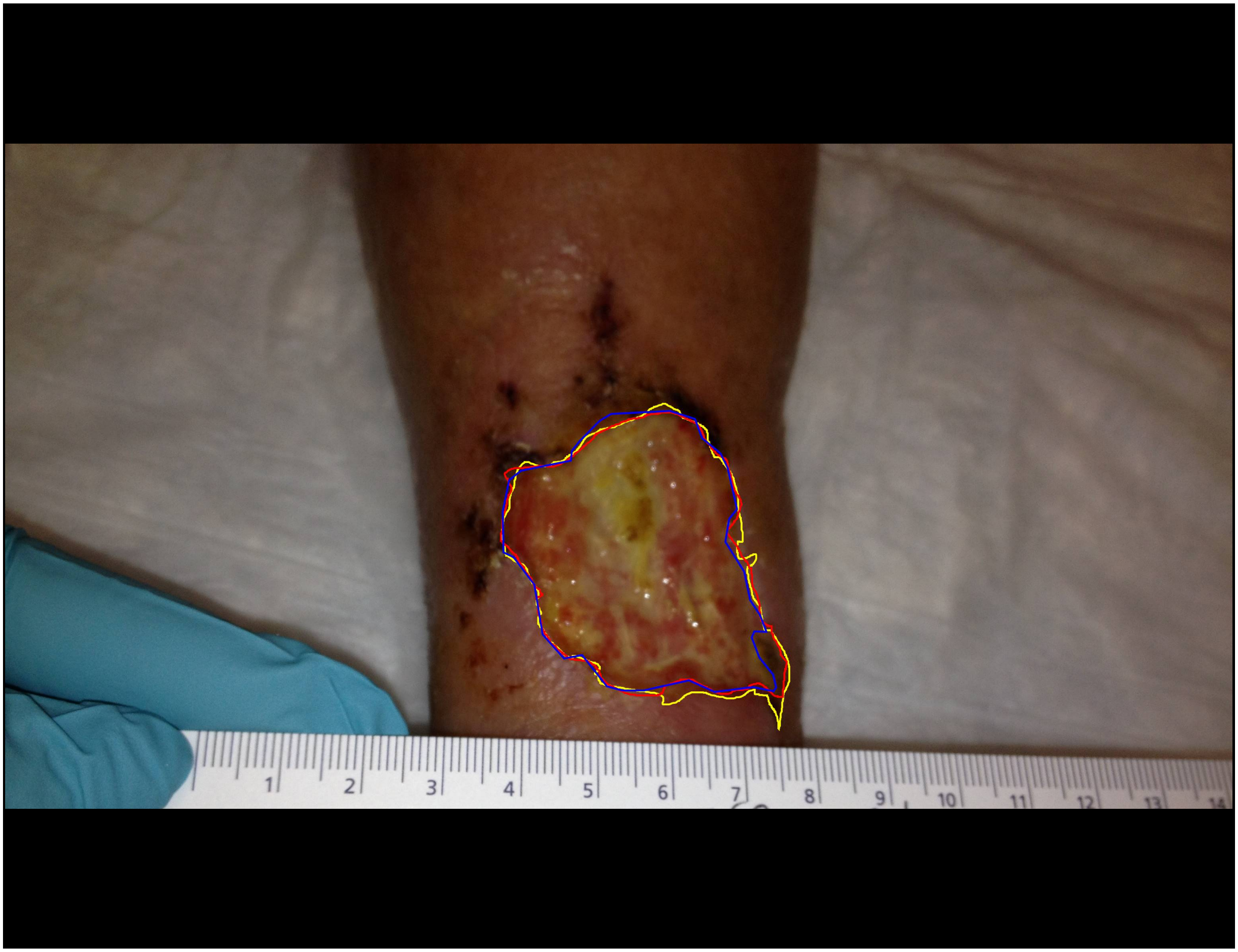

Tracing Data			
Tracer:	Wound Area (px^2)	Ruler Calibration (px/cm)	Wound Area (cm^2)
H1	400606	201.5	9.87
H2	387807	198.2	9.88
AI	380006	205.5	9.00

Tracing Comparisons				
Difference Metric:	Human-Human		Human-AI	
	H1(ref) H2(test)	H2(ref) H1(test)	H1(ref) AI(test)	H2(ref) AI(test)
False Negative Area (%)	4.7	1.5	7.2	4.5
False Positive Area (%)	1.5	4.8	2.1	2.5
|Relative Error (%)|	3.2	3.3	5.1	2.0

Blinded Attending Surgeon Review						
Reviewer	PGT Estimate	H1 meets definition?	H2 meets definition?	AI meets definition?	Which is AI?	Which is most accurate?
1	20	Yes	Yes	No	H1	H1
2	20	Yes	Yes	Yes	AI	AI
3	60	Yes	No	No	H2	AI

© 2021 Howell RS et al. *JAMA Network Open.*

Wound EMR Information								
Sequential Number	Gender	Age	Wound Type	Wound Location	Wound Length (cm)	Wound Width (cm)	Wound Depth (cm)	Wound Area (cm^2)
28	F	76	blister	RLE med	4.7	3.5		16.45

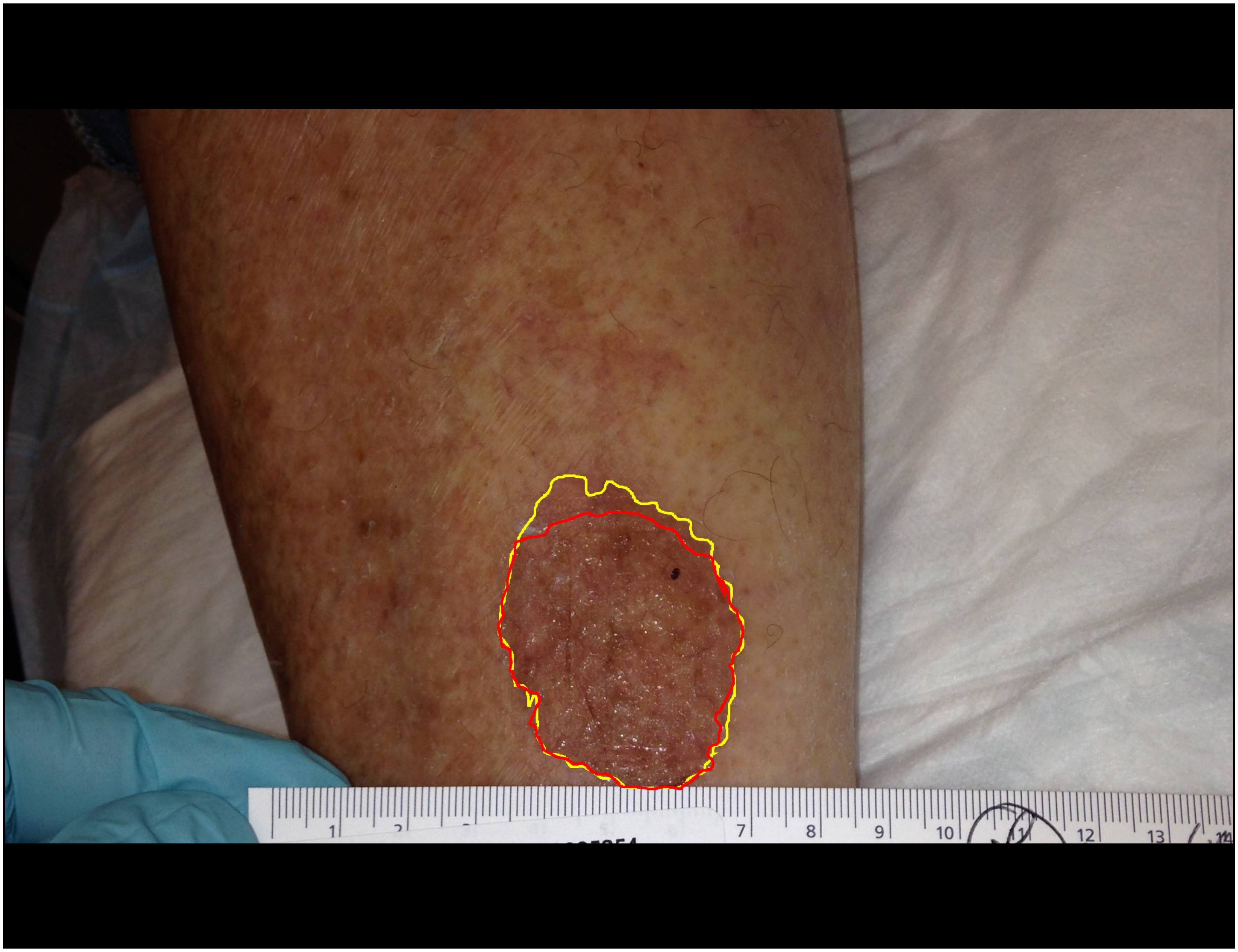

Tracing Data			
Tracer:	Wound Area (px^2)	Ruler Calibration (px/cm)	Wound Area (cm^2)
H1	223618	137.4	11.85
H2	202132	135.0	11.09
AI	0		0.00

Tracing Comparisons				
Difference Metric:	Human-Human		Human-AI	
	H1(ref) H2(test)	H2(ref) H1(test)	H1(ref) AI(test)	H2(ref) AI(test)
False Negative Area (%)	10.3	0.8		
False Positive Area (%)	0.7	11.4		
|Relative Error (%)|	9.6	10.6		

Blinded Attending Surgeon Review						
Reviewer	PGT Estimate	H1 meets definition?	H2 meets definition?	AI meets definition?	Which is AI?	Which is most accurate?
1	0	Yes	Yes	Yes	0	0
2	healed	No	No	Yes	AI	H2
3	0	No	Yes	Yes	AI	H2

© 2021 Howell RS et al. *JAMA Network Open.*

Wound EMR Information								
Sequential Number	Gender	Age	Wound Type	Wound Location	Wound Length (cm)	Wound Width (cm)	Wound Depth (cm)	Wound Area (cm^2)
29	M	59	DFU	L 2nd toe	2.0	2.0	0.1	4.00

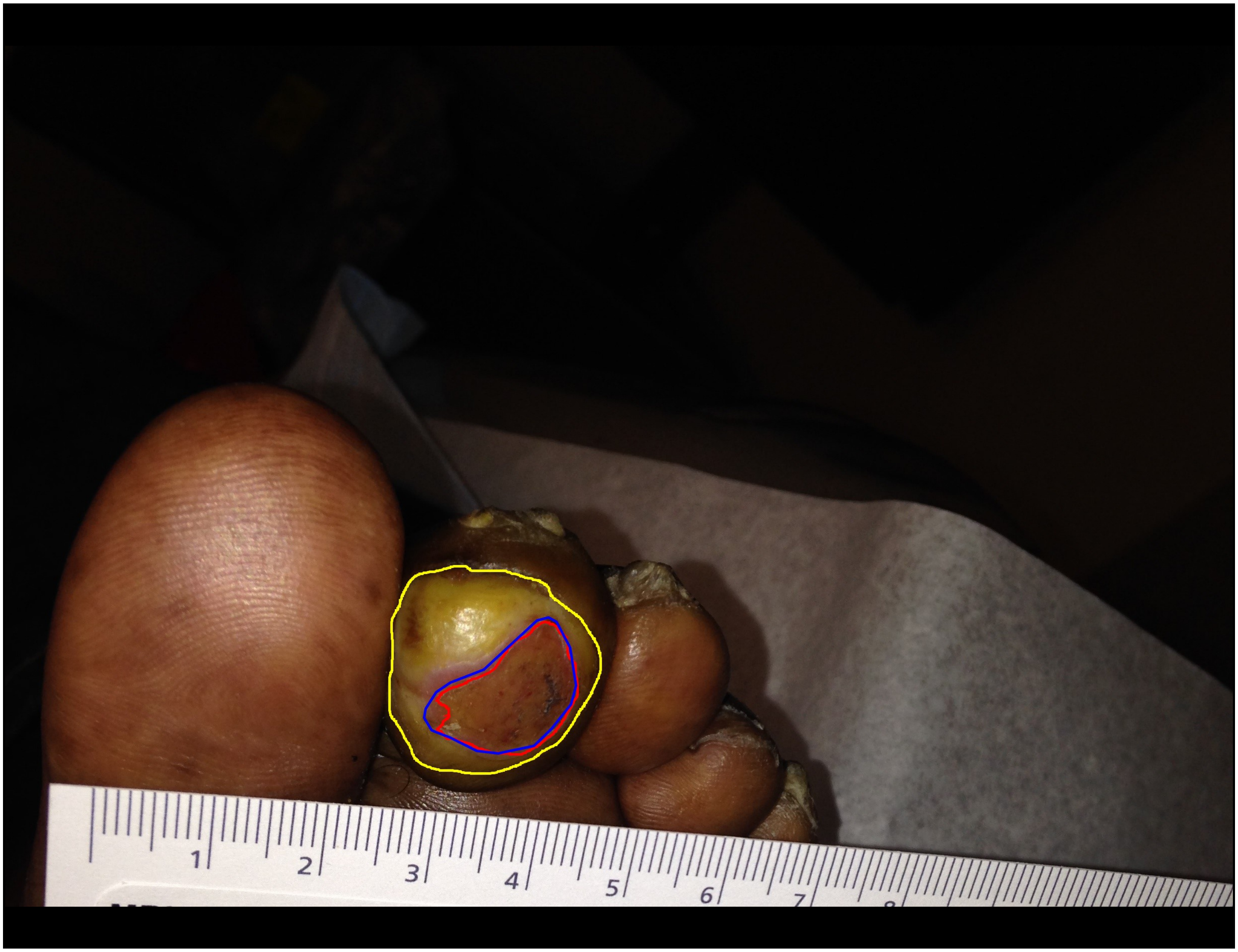

Tracing Data			
Tracer:	Wound Area (px^2)	Ruler Calibration (px/cm)	Wound Area (cm^2)
H1	137280	207.4	3.19
H2	45583	206.2	1.07
AI	49227	233.9	0.90

Tracing Comparisons				
Difference Metric:	Human-Human		Human-AI	
	H1(ref) H2(test)	H2(ref) H1(test)	H1(ref) AI(test)	H2(ref) AI(test)
False Negative Area (%)	66.8	0.1	64.1	3.3
False Positive Area (%)	0.0	201.3	0.0	11.3
|Relative Error (%)|	66.8	201.2	64.1	8.0

Blinded Attending Surgeon Review						
Reviewer	PGT Estimate	H1 meets definition?	H2 meets definition?	AI meets definition?	Which is AI?	Which is most accurate?
1	20	No	Yes	Yes	AI	H2
2	0	No	Yes	Yes	H2	H1
3	40	No	Yes	Yes	AI	H2

© 2021 Howell RS et al. *JAMA Network Open.*

Wound EMR Information								
Sequential Number	Gender	Age	Wound Type	Wound Location	Wound Length (cm)	Wound Width (cm)	Wound Depth (cm)	Wound Area (cm^2)
30	M	66	PU	R ischium	2.0	2.5	0.8	5.00

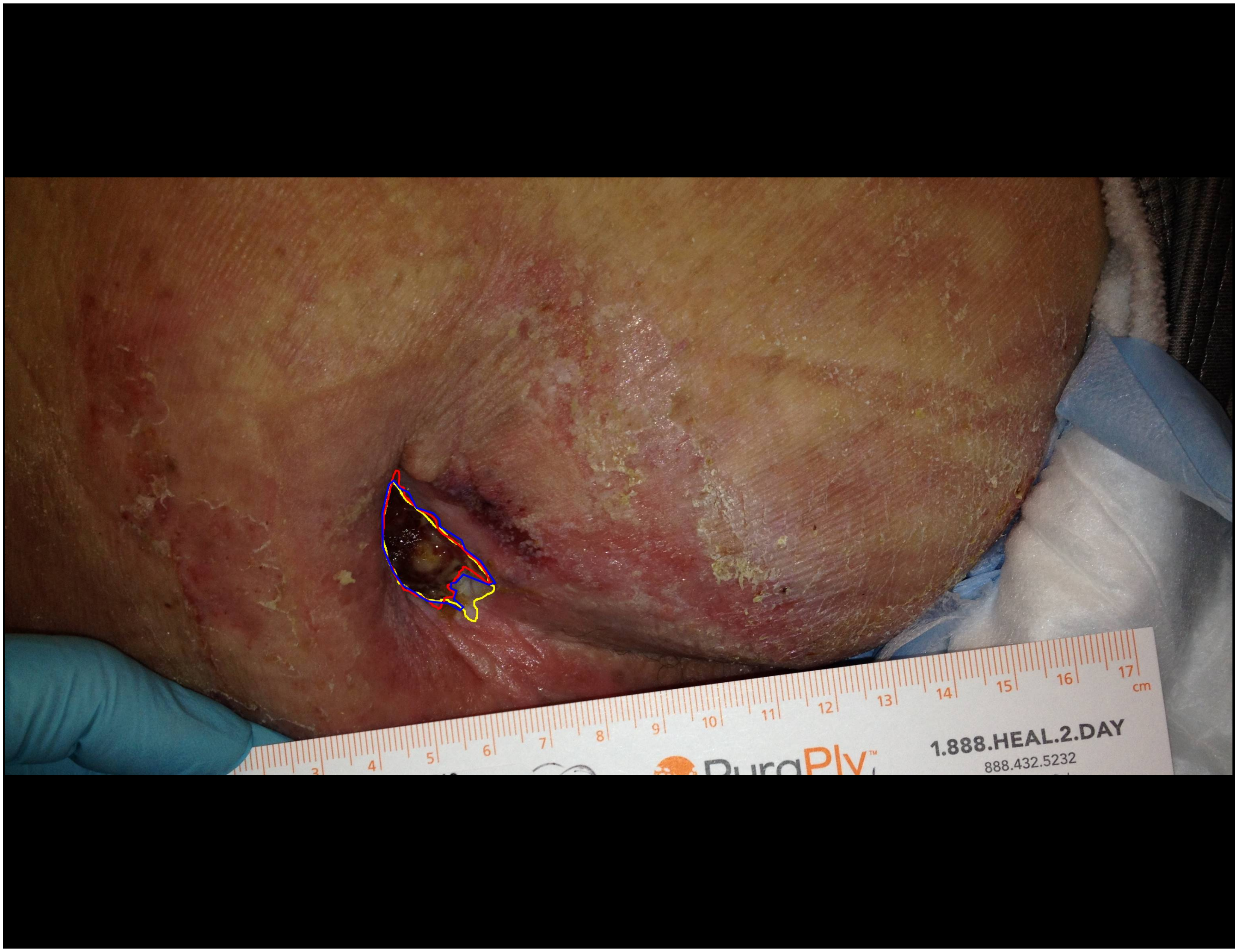

Tracing Data			
Tracer:	Wound Area (px^2)	Ruler Calibration (px/cm)	Wound Area (cm^2)
H1	50954	150.8	2.24
H2	44714	153.3	1.90
AI	49269	153.2	2.10

Tracing Comparisons				
Difference Metric:	Human-Human		Human-AI	
	H1(ref) H2(test)	H2(ref) H1(test)	H1(ref) AI(test)	H2(ref) AI(test)
False Negative Area (%)	18.2	6.8	13.8	2.8
False Positive Area (%)	6.0	20.8	10.5	13.0
|Relative Error (%)|	12.2	14.0	3.3	10.2

Blinded Attending Surgeon Review						
Reviewer	PGT Estimate	H1 meets definition?	H2 meets definition?	AI meets definition?	Which is AI?	Which is most accurate?
1		Yes	Yes	Yes	AI	0
2	0	No	Yes	Yes	H1	H1
3	80	No	Yes	Yes	H2	H2

© 2021 Howell RS et al. *JAMA Network Open.*

Wound EMR Information								
Sequential Number	Gender	Age	Wound Type	Wound Location	Wound Length (cm)	Wound Width (cm)	Wound Depth (cm)	Wound Area (cm^2)
31	F	89	Trauma	LLE	5.6	4.0	0.1	22.40

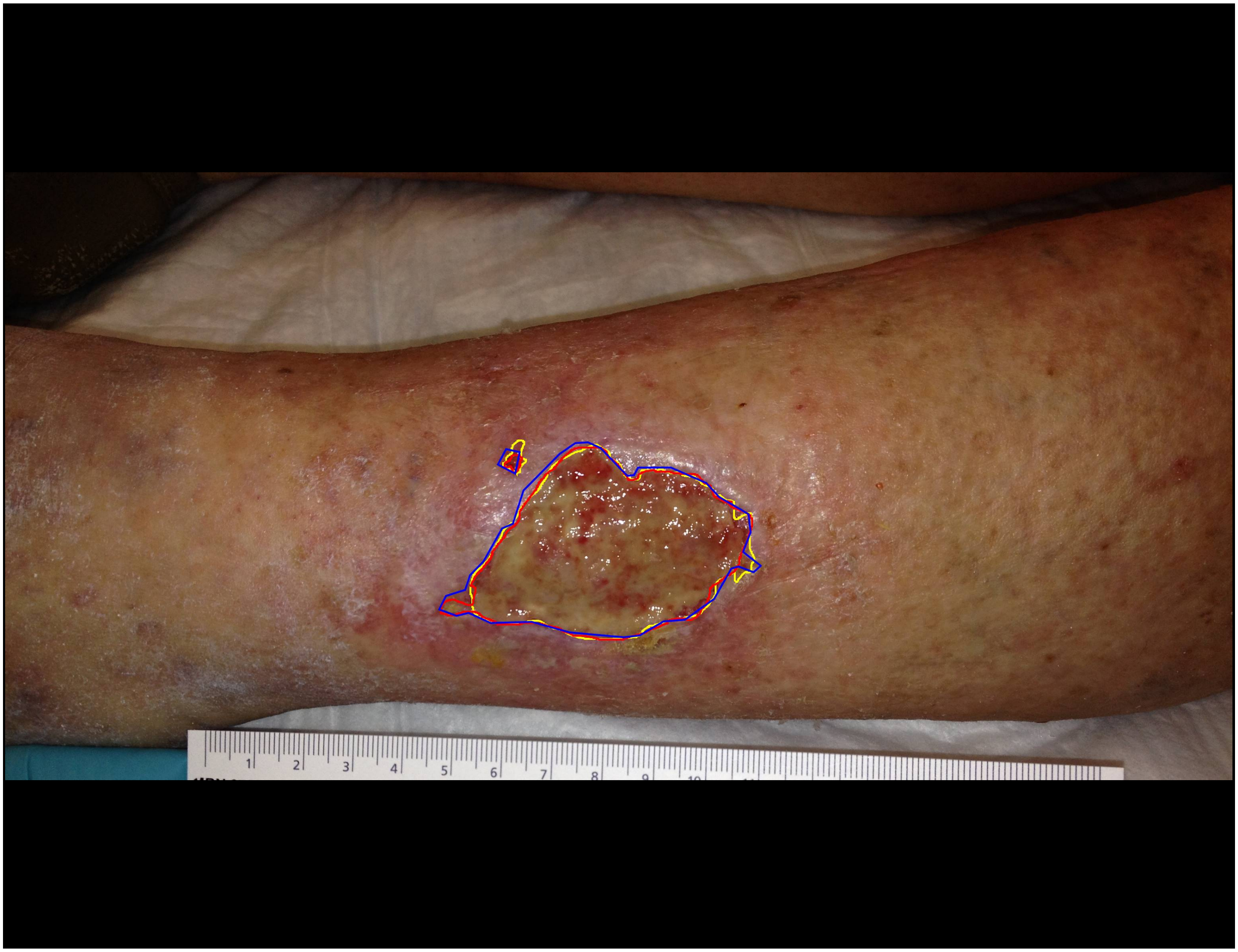

Tracing Data			
Tracer:	Wound Area (px^2)	Ruler Calibration (px/cm)	Wound Area (cm^2)
H1	262453	134.9	14.42
H2	259235	134.1	14.41
AI	272352	130.9	15.90

Tracing Comparisons				
Difference Metric:	Human-Human		Human-AI	
	H1(ref) H2(test)	H2(ref) H1(test)	H1(ref) AI(test)	H2(ref) AI(test)
False Negative Area (%)	3.6	2.4	2.3	1.4
False Positive Area (%)	2.4	3.6	6.1	6.4
|Relative Error (%)|	1.2	1.2	3.8	5.1

Blinded Attending Surgeon Review						
Reviewer	PGT Estimate	H1 meets definition?	H2 meets definition?	AI meets definition?	Which is AI?	Which is most accurate?
1	10	Yes	Yes	Yes	AI	AI
2	10	No	Yes	Yes	H1	H2
3	90	Yes	Yes	Yes	AI	H2

© 2021 Howell RS et al. *JAMA Network Open.*

Wound EMR Information								
Sequential Number	Gender	Age	Wound Type	Wound Location	Wound Length (cm)	Wound Width (cm)	Wound Depth (cm)	Wound Area (cm^2)
32	F	93	VLU	LLE ant	4.7	3.4	0.1	15.98

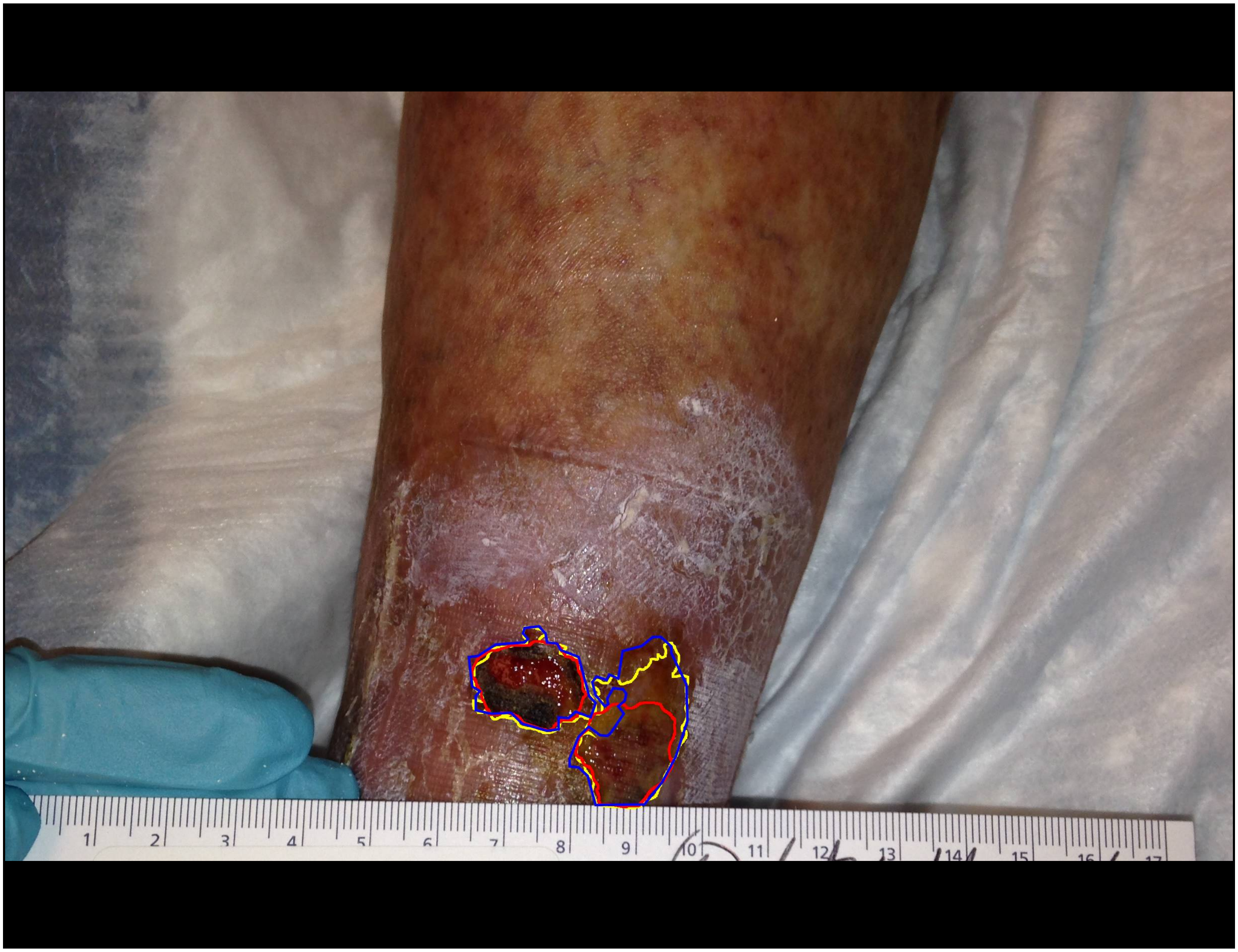

Tracing Data			
Tracer:	Wound Area (px^2)	Ruler Calibration (px/cm)	Wound Area (cm^2)
H1	79719	132.8	4.52
H2	59042	132.0	3.39
AI	81784	72.9	15.40

Tracing Comparisons				
Difference Metric:	Human-Human		Human-AI	
	H1(ref) H2(test)	H2(ref) H1(test)	H1(ref) AI(test)	H2(ref) AI(test)
False Negative Area (%)	26.9	1.2	8.3	5.3
False Positive Area (%)	0.9	36.3	10.9	43.8
|Relative Error (%)|	25.9	35.0	2.6	38.5

Blinded Attending Surgeon Review						
Reviewer	PGT Estimate	H1 meets definition?	H2 meets definition?	AI meets definition?	Which is AI?	Which is most accurate?
1	10	No	Yes	No	H1	H1
2	30	No	No	Yes	H2	AI
3	60	No	Yes	No	AI	H1

© 2021 Howell RS et al. *JAMA Network Open.*

Wound EMR Information								
Sequential Number	Gender	Age	Wound Type	Wound Location	Wound Length (cm)	Wound Width (cm)	Wound Depth (cm)	Wound Area (cm^2)
33	F	79	VLU	LLE medial	3.3	2.2	0.1	7.26

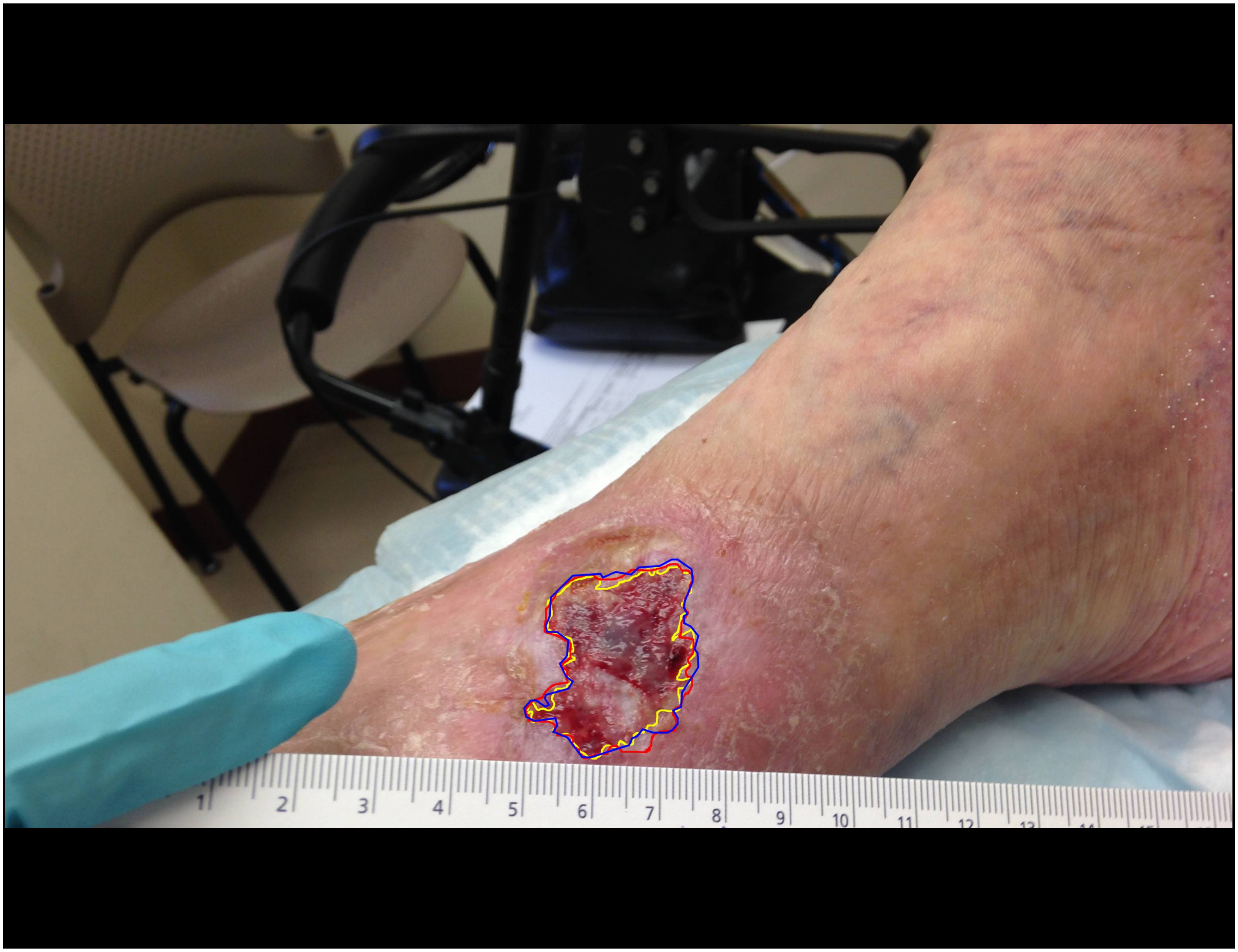

Tracing Data			
Tracer:	Wound Area (px^2)	Ruler Calibration (px/cm)	Wound Area (cm^2)
H1	143239	185.1	4.18
H2	159947	178.8	5.00
AI	160527	195.5	4.20

Tracing Comparisons				
Difference Metric:	Human-Human		Human-AI	
	H1(ref) H2(test)	H2(ref) H1(test)	H1(ref) AI(test)	H2(ref) AI(test)
False Negative Area (%)	1.1	11.4	0.9	4.6
False Positive Area (%)	12.7	1.0	13.0	5.0
|Relative Error (%)|	11.7	10.4	12.1	0.4

Blinded Attending Surgeon Review						
Reviewer	PGT Estimate	H1 meets definition?	H2 meets definition?	AI meets definition?	Which is AI?	Which is most accurate?
1	40	No	No	No	H1	H2
2	30	No	No	No	H1	H1
3	60	Yes	No	No	AI	H1

© 2021 Howell RS et al. *JAMA Network Open.*

Wound EMR Information								
Sequential Number	Gender	Age	Wound Type	Wound Location	Wound Length (cm)	Wound Width (cm)	Wound Depth (cm)	Wound Area (cm^2)
34	M	56	VLU	RLE ant	0.0	0.0	0.0	0.00

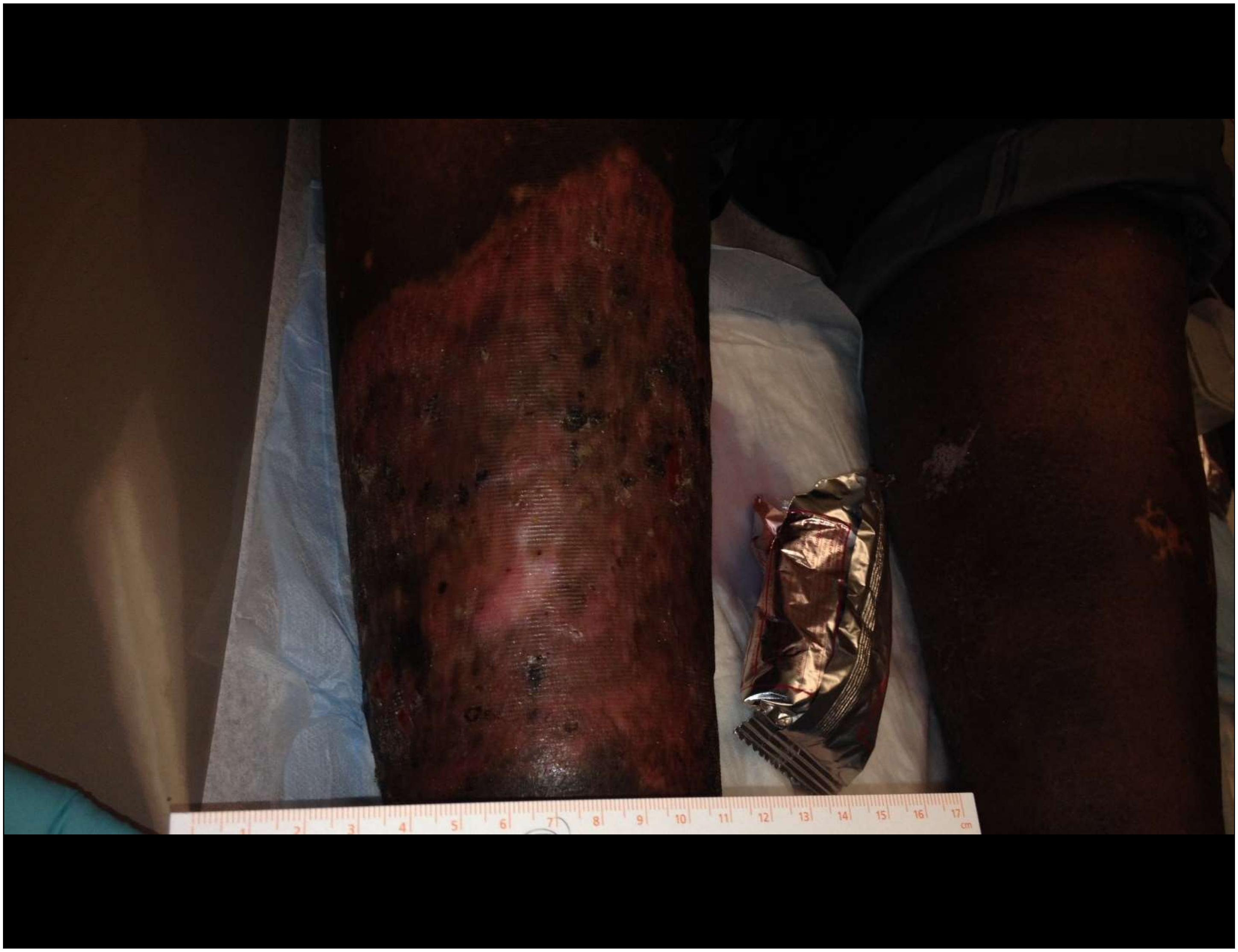

Tracing Data			
Tracer:	Wound Area (px^2)	Ruler Calibration (px/cm)	Wound Area (cm^2)
H1	0		0.00
H2	0		0.00
AI	0		0.00

Tracing Comparisons				
Difference Metric:	Human-Human		Human-AI	
	H1(ref) H2(test)	H2(ref) H1(test)	H1(ref) AI(test)	H2(ref) AI(test)
False Negative Area (%)				
False Positive Area (%)				
|Relative Error (%)|				

Blinded Attending Surgeon Review						
Reviewer	PGT Estimate	H1 meets definition?	H2 meets definition?	AI meets definition?	Which is AI?	Which is most accurate?
1	healed	Yes	Yes	Yes	0	0
2	healed	Yes	Yes	Yes	0	0
3	0	Yes	Yes	Yes	0	0

© 2021 Howell RS et al. *JAMA Network Open.*

Wound EMR Information								
**Sequential Number**	**Gender**	**Age**	**Wound Type**	**Wound Location**	**Wound Length (cm)**	**Wound Width (cm)**	**Wound Depth (cm)**	**Wound Area (cm^2)**
35	F	94	VLU	RLE ant	0.5	1.2	0.1	0.60

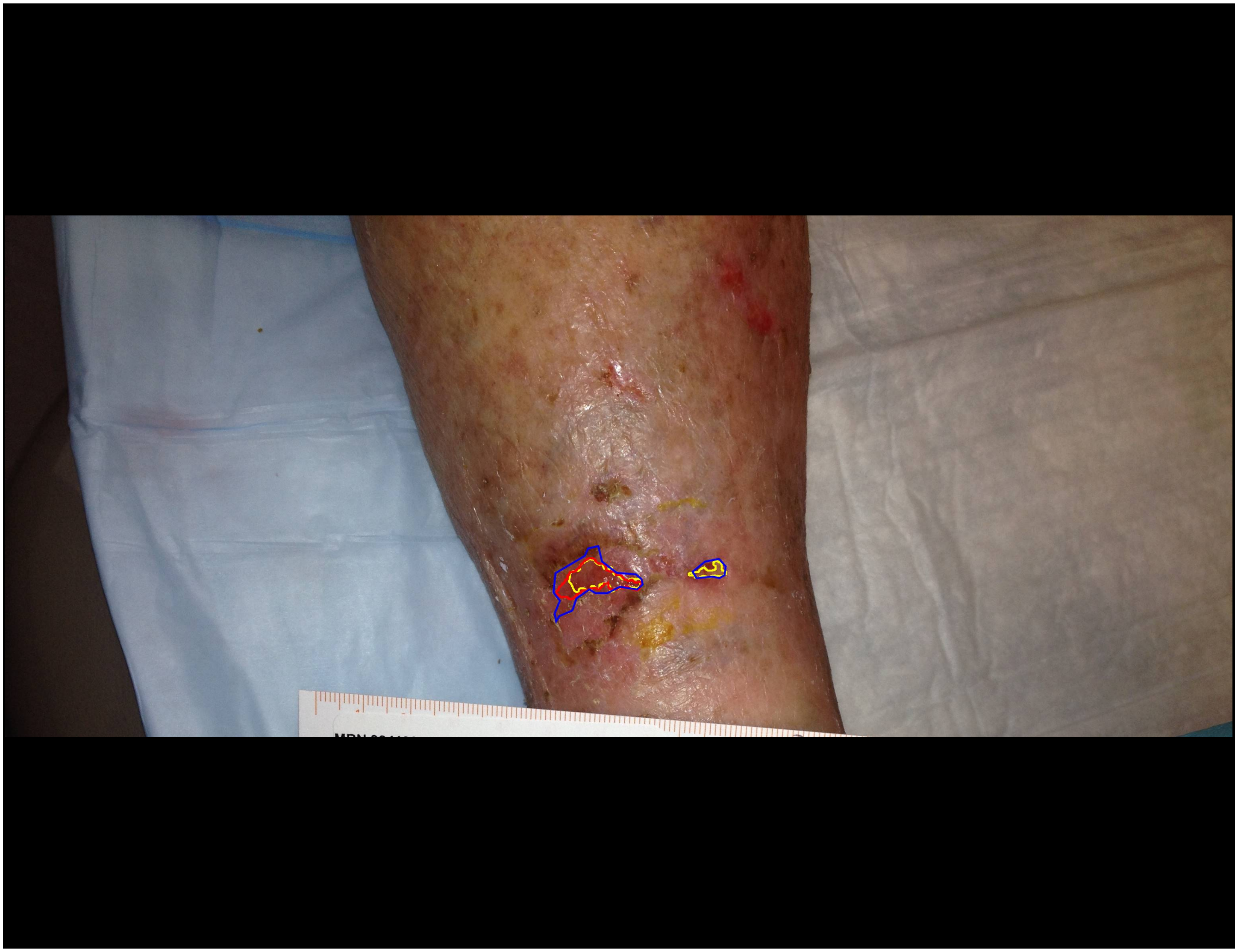

Tracing Data			
**Tracer:**	**Wound Area (px^2)**	**Ruler Calibration (px/cm)**	**Wound Area (cm^2)**
**H1**	10429	125.7	0.66
**H2**	10817	121.1	0.74
**AI**	22106	130.4	1.30

Tracing Comparisons				
**Difference Metric:**	**Human-Human**		**Human-AI**	
	**H1(ref) H2(test)**	**H2(ref) H1(test)**	**H1(ref) AI(test)**	**H2(ref) AI(test)**
**False Negative Area (%)**	22.3	25.1	1.8	0.6
**False Positive Area (%)**	26.0	21.5	113.8	105.0
**|Relative Error (%)|**	3.7	3.6	112.0	104.4

Blinded Attending Surgeon Review						
**Reviewer**	**PGT Estimate**	**H1 meets definition?**	**H2 meets definition?**	**AI meets definition?**	**Which is AI?**	**Which is most accurate?**
**1**	100	Yes	No	No	H2	H1
**2**	80	Yes	Yes	No	H1	AI
**3**	30	Yes	Yes	No	H2	H1

© 2021 Howell RS et al. *JAMA Network Open.*

Wound EMR Information								
Sequential Number	Gender	Age	Wound Type	Wound Location	Wound Length (cm)	Wound Width (cm)	Wound Depth (cm)	Wound Area (cm^2)
36	F	62	Trauma	LLE ant	1.8	2.4	0.6	4.32

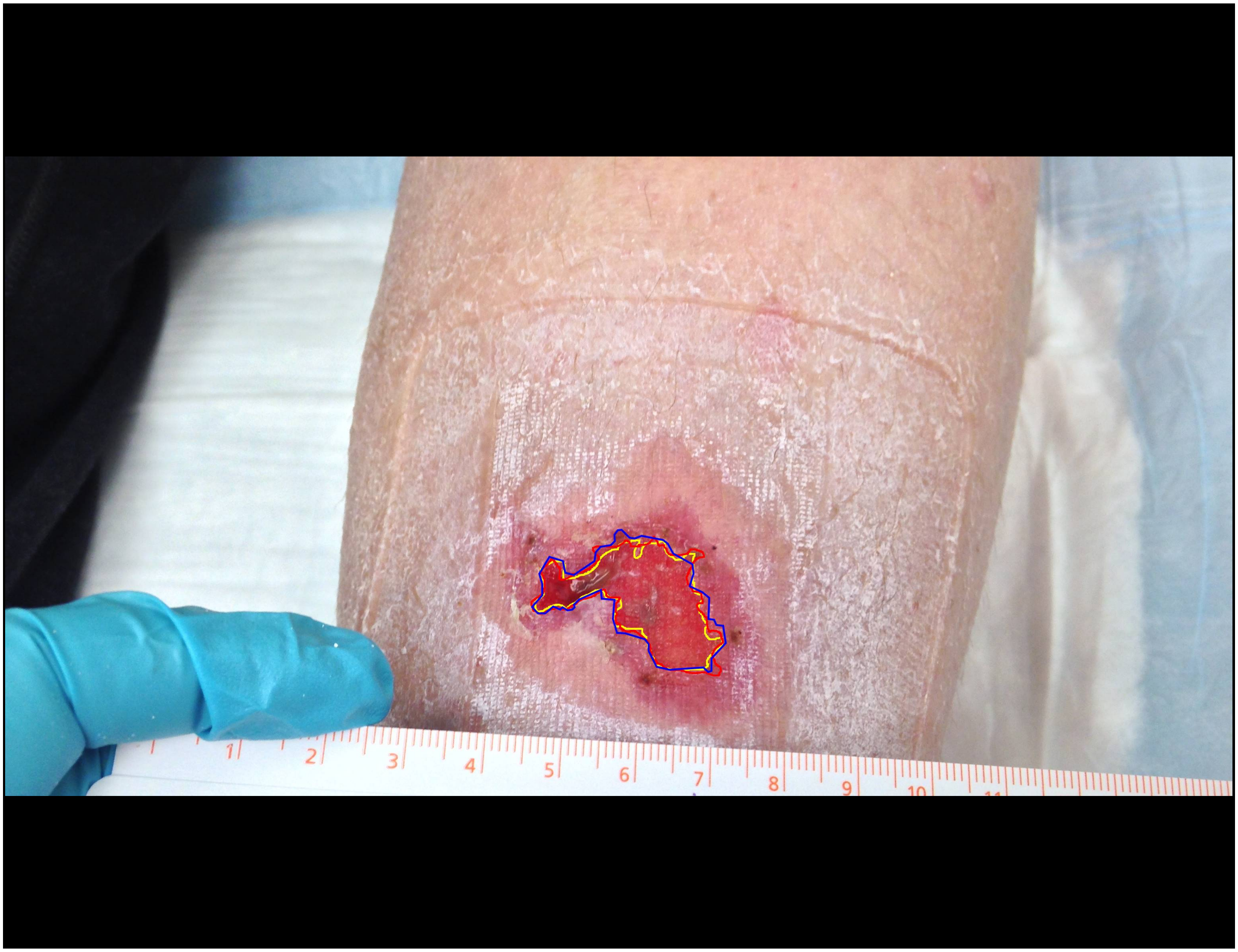

Tracing Data			
Tracer:	Wound Area (px^2)	Ruler Calibration (px/cm)	Wound Area (cm^2)
H1	81400	204.3	1.95
H2	88130	199.1	2.22
AI	95726	208.6	2.20

Tracing Comparisons				
Difference Metric:	Human-Human		Human-AI	
	H1(ref) H2(test)	H2(ref) H1(test)	H1(ref) AI(test)	H2(ref) AI(test)
False Negative Area (%)	3.5	10.9	1.3	4.5
False Positive Area (%)	11.8	3.3	18.9	13.2
|Relative Error (%)|	8.3	7.6	17.6	8.6

Blinded Attending Surgeon Review						
Reviewer	PGT Estimate	H1 meets definition?	H2 meets definition?	AI meets definition?	Which is AI?	Which is most accurate?
1	90	Yes	No	No	H2	H2
2	60	Yes	Yes	Yes	AI	H1
3	50	Yes	Yes	No	AI	H2

© 2021 Howell RS et al. *JAMA Network Open.*

Wound EMR Information								
Sequential Number	Gender	Age	Wound Type	Wound Location	Wound Length (cm)	Wound Width (cm)	Wound Depth (cm)	Wound Area (cm^2)
37	F	84	VLU	RLE ant	8.8	1.5	0.1	13.20

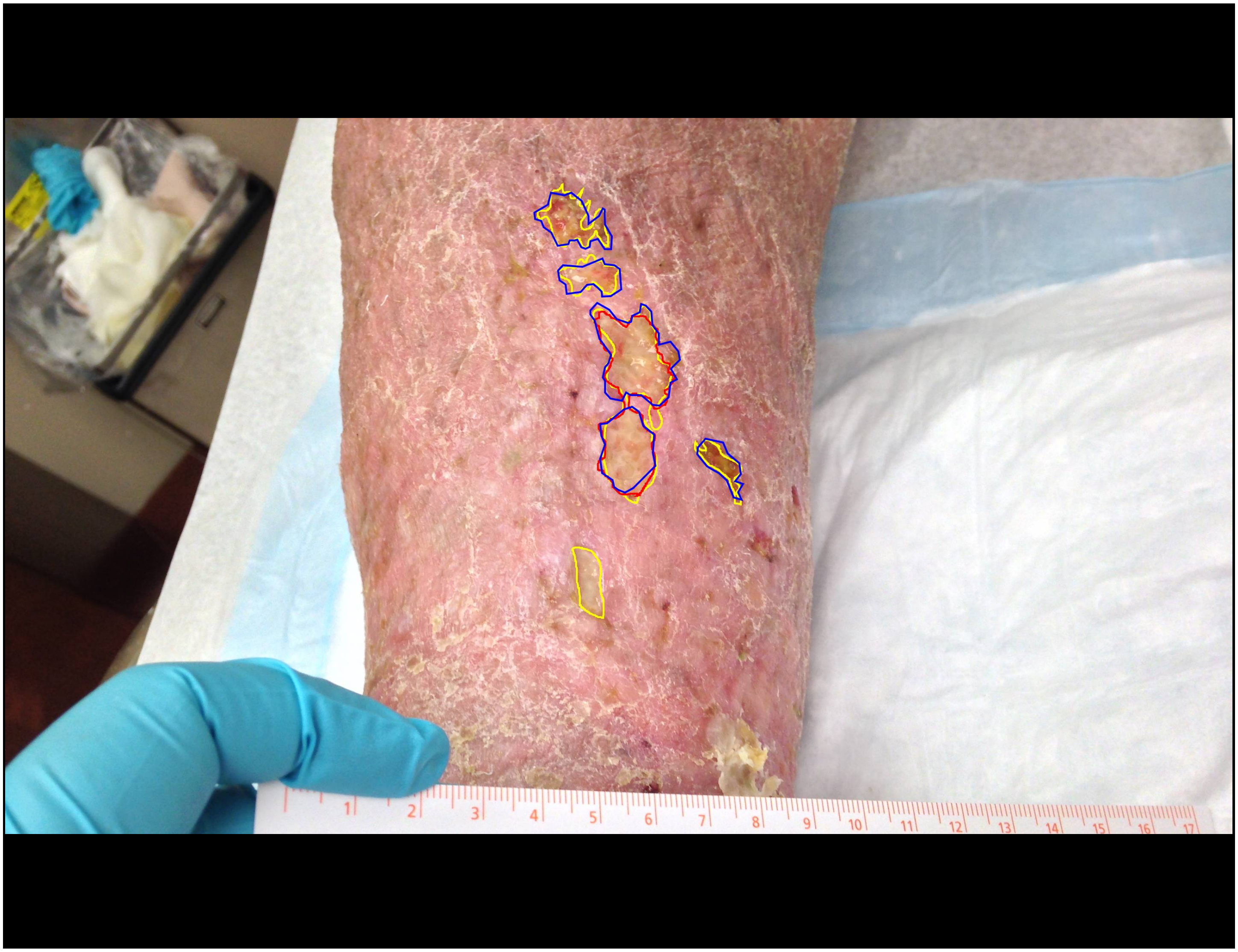

Tracing Data			
Tracer:	Wound Area (px^2)	Ruler Calibration (px/cm)	Wound Area (cm^2)
H1	100790	160.6	3.91
H2	57479	112.7	4.53
AI	101038	151.5	4.40

Tracing Comparisons				
Difference Metric:	Human-Human		Human-AI	
	H1(ref) H2(test)	H2(ref) H1(test)	H1(ref) AI(test)	H2(ref) AI(test)
False Negative Area (%)	47.5	8.0	18.6	5.8
False Positive Area (%)	4.5	83.3	18.8	81.6
|Relative Error (%)|	43.0	75.4	0.2	75.8

Blinded Attending Surgeon Review						
Reviewer	PGT Estimate	H1 meets definition?	H2 meets definition?	AI meets definition?	Which is AI?	Which is most accurate?
1	20	Yes	No	Yes	AI	AI
2	0	No	Yes	No	H2	H2
3	0	Yes	Yes	No	H2	H2

© 2021 Howell RS et al. *JAMA Network Open.*

Wound EMR Information								
Sequential Number	Gender	Age	Wound Type	Wound Location	Wound Length (cm)	Wound Width (cm)	Wound Depth (cm)	Wound Area (cm^2)
38	F	67	VLU	LLE ant	8.5	6.0	0.1	51.00

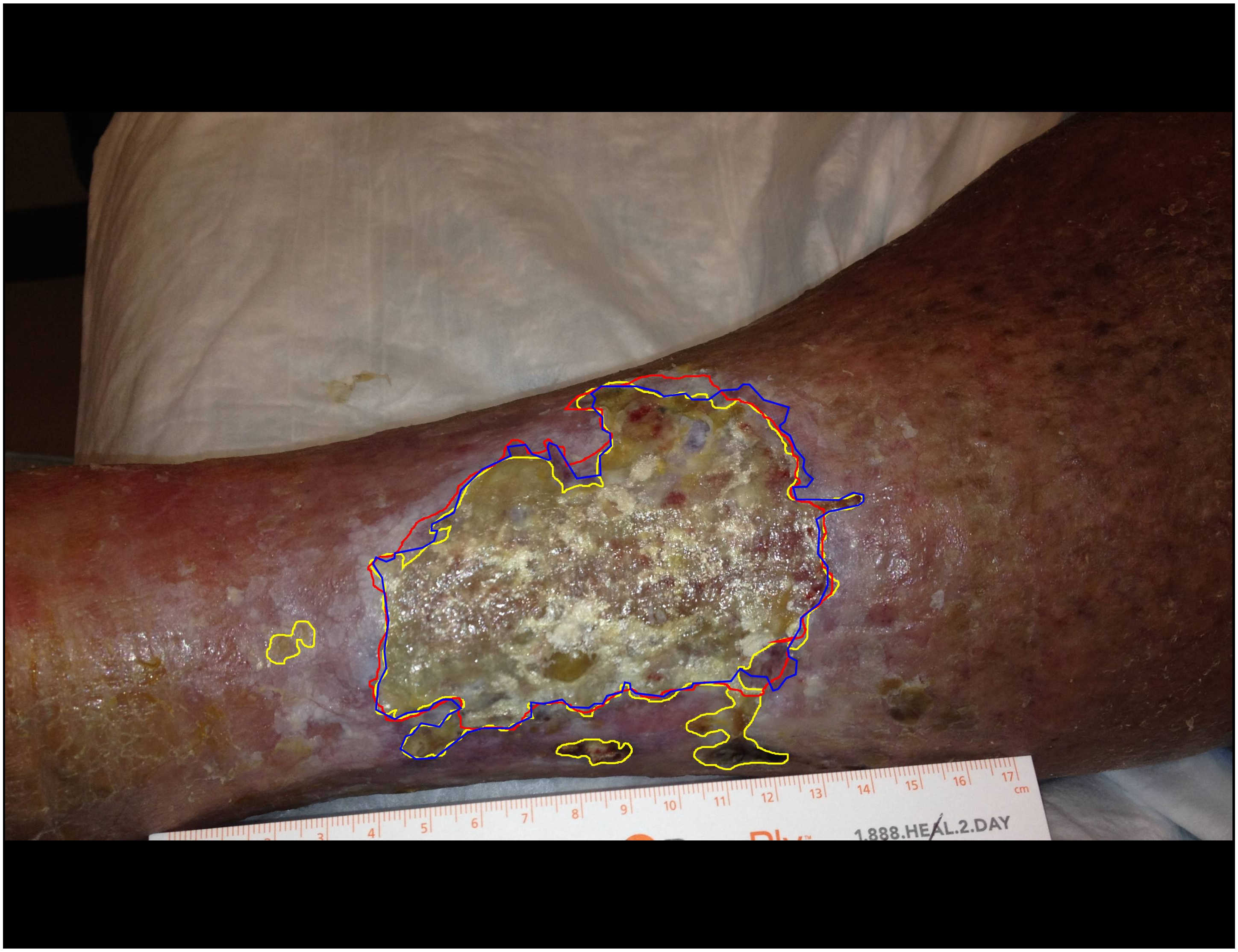

Tracing Data			
Tracer:	Wound Area (px^2)	Ruler Calibration (px/cm)	Wound Area (cm^2)
H1	801636	130.9	46.77
H2	794980	130.2	46.86
AI	782506	136.7	41.90

Tracing Comparisons				
Difference Metric:	Human-Human		Human-AI	
	H1(ref) H2(test)	H2(ref) H1(test)	H1(ref) AI(test)	H2(ref) AI(test)
False Negative Area (%)	8.2	7.4	7.6	5.6
False Positive Area (%)	7.4	8.2	5.2	4.0
|Relative Error (%)|	0.8	0.8	2.4	1.6

Blinded Attending Surgeon Review						
Reviewer	PGT Estimate	H1 meets definition?	H2 meets definition?	AI meets definition?	Which is AI?	Which is most accurate?
1	0	No	No	No	AI	AI
2	0	No	No	No	AI	H2
3	0	Yes	No	Yes	H1	AI

© 2021 Howell RS et al. *JAMA Network Open.*

Wound EMR Information								
Sequential Number	Gender	Age	Wound Type	Wound Location	Wound Length (cm)	Wound Width (cm)	Wound Depth (cm)	Wound Area (cm^2)
39	F	41	Surgical	Abdomen	0.5	1.5	0.1	0.75

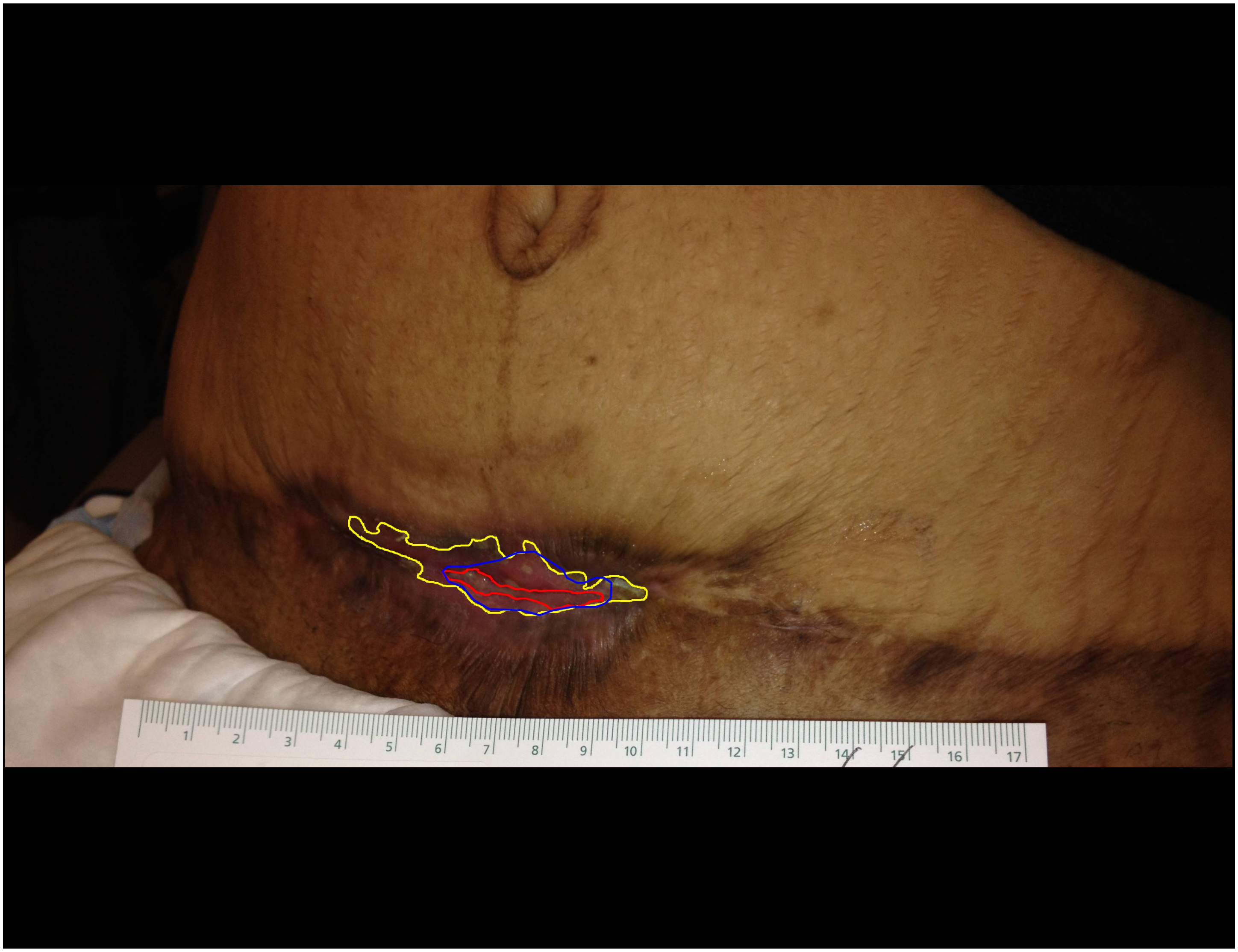

Tracing Data			
Tracer:	Wound Area (px^2)	Ruler Calibration (px/cm)	Wound Area (cm^2)
H1	76087	129.3	4.55
H2	13935	129.0	0.84
AI	45348	134.7	2.50

Tracing Comparisons				
Difference Metric:	Human-Human		Human-AI	
	H1(ref) H2(test)	H2(ref) H1(test)	H1(ref) AI(test)	H2(ref) AI(test)
False Negative Area (%)	81.7	0.0	43.0	0.0
False Positive Area (%)	0.0	446.0	2.6	225.4
|Relative Error (%)|	81.7	446.0	40.4	225.4

Blinded Attending Surgeon Review						
Reviewer	PGT Estimate	H1 meets definition?	H2 meets definition?	AI meets definition?	Which is AI?	Which is most accurate?
1	30	Yes	Yes	Yes	AI	0
2	0	No	Yes	No	H2	AI
3	20	No	Yes	No	H1	AI

© 2021 Howell RS et al. *JAMA Network Open.*

Wound EMR Information								
Sequential Number	Gender	Age	Wound Type	Wound Location	Wound Length (cm)	Wound Width (cm)	Wound Depth (cm)	Wound Area (cm^2)
40	F	72	VLU	R foot dorsal	4.2	4.7	0.2	19.74

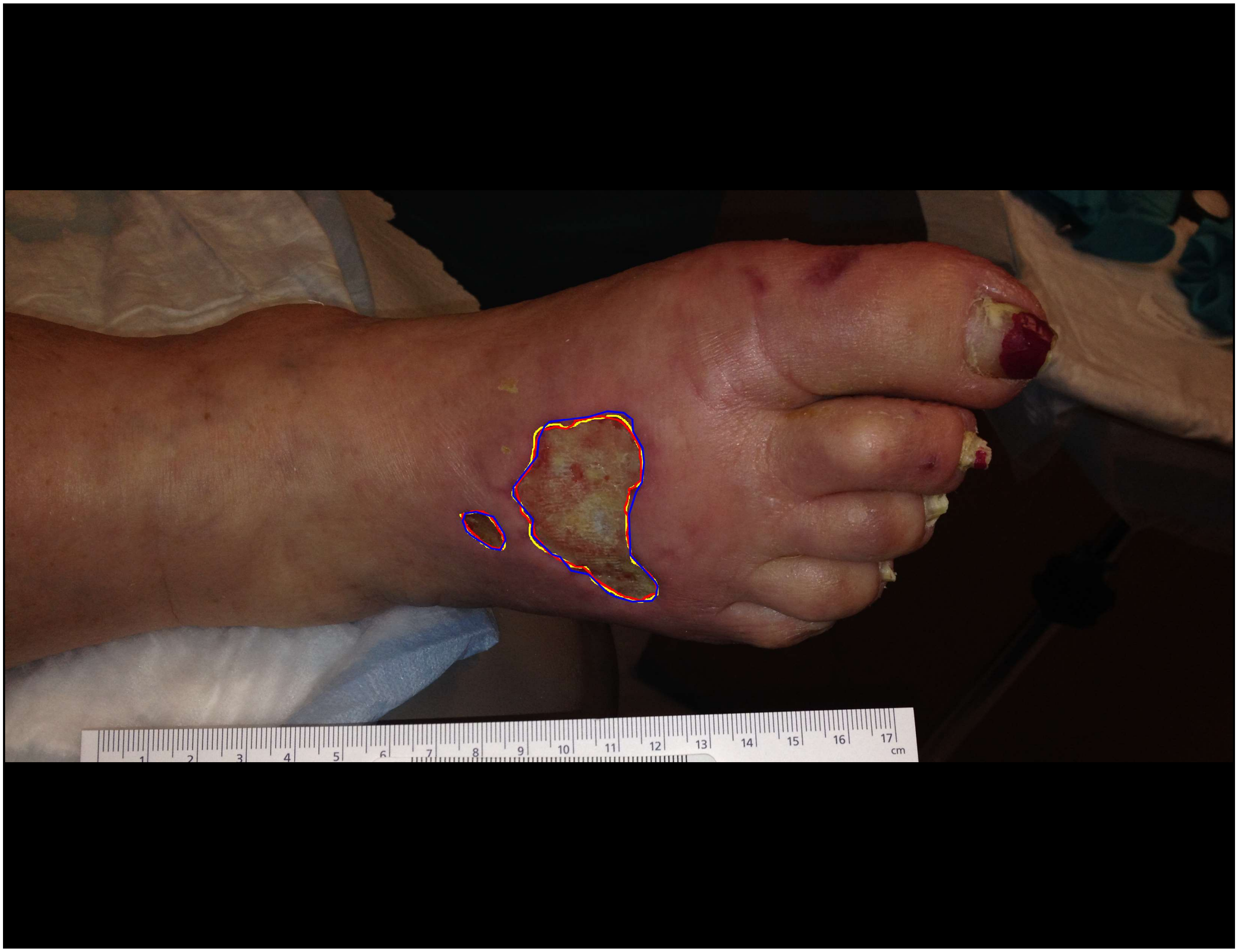

Tracing Data			
Tracer:	Wound Area (px^2)	Ruler Calibration (px/cm)	Wound Area (cm^2)
H1	122612	122.7	8.14
H2	118393	121.3	8.04
AI	127823	121.9	8.60

Tracing Comparisons				
Difference Metric:	Human-Human		Human-AI	
	H1(ref) H2(test)	H2(ref) H1(test)	H1(ref) AI(test)	H2(ref) AI(test)
False Negative Area (%)	4.8	1.4	2.3	1.3
False Positive Area (%)	1.4	5.0	6.6	9.3
|Relative Error (%)|	3.4	3.6	4.3	8.0

Blinded Attending Surgeon Review						
Reviewer	PGT Estimate	H1 meets definition?	H2 meets definition?	AI meets definition?	Which is AI?	Which is most accurate?
1	10	Yes	Yes	Yes	H2	H1
2	0	Yes	Yes	Yes	AI	H2
3	10	Yes	Yes	Yes	AI	H1

© 2021 Howell RS et al. *JAMA Network Open.*

Wound EMR Information								
Sequential Number	Gender	Age	Wound Type	Wound Location	Wound Length (cm)	Wound Width (cm)	Wound Depth (cm)	Wound Area (cm^2)
41	F	74	VLU	LLE medial	3.0	2.8	0.2	8.40

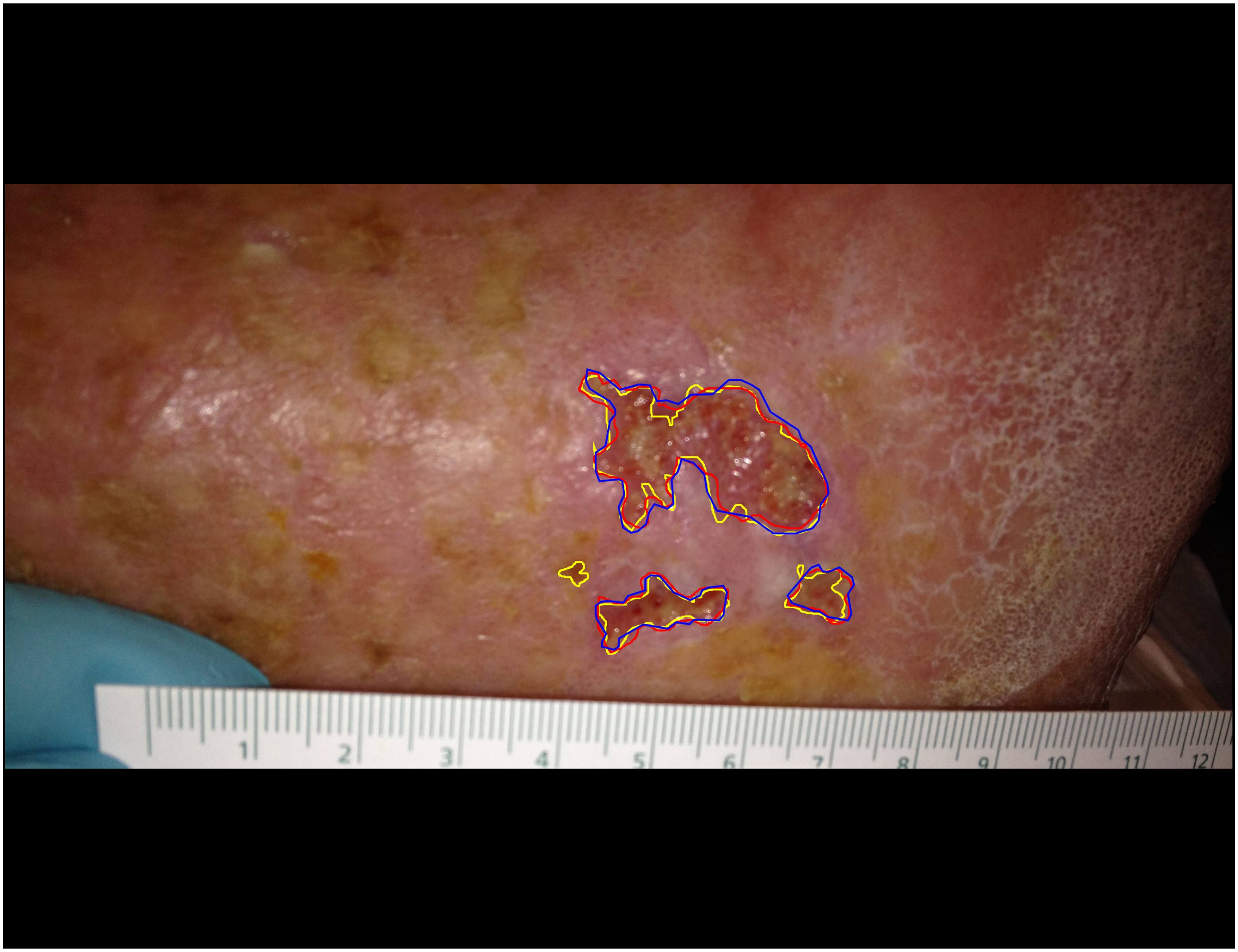

Tracing Data			
Tracer:	Wound Area (px^2)	Ruler Calibration (px/cm)	Wound Area (cm^2)
H1	199393	254.9	3.07
H2	203685	248.2	3.31
AI	218934	265.8	3.10

Tracing Comparisons				
Difference Metric:	Human-Human		Human-AI	
	H1(ref) H2(test)	H2(ref) H1(test)	H1(ref) AI(test)	H2(ref) AI(test)
False Negative Area (%)	8.6	10.6	5.4	4.9
False Positive Area (%)	10.8	8.5	15.2	12.3
|Relative Error (%)|	2.2	2.1	9.8	7.5

Blinded Attending Surgeon Review						
Reviewer	PGT Estimate	H1 meets definition?	H2 meets definition?	AI meets definition?	Which is AI?	Which is most accurate?
1	70	Yes	No	No	H1	H2
2	10	No	No	No	AI	H2
3	30	No	Yes	Yes	AI	AI

© 2021 Howell RS et al. *JAMA Network Open.*

Wound EMR Information								
Sequential Number	Gender	Age	Wound Type	Wound Location	Wound Length (cm)	Wound Width (cm)	Wound Depth (cm)	Wound Area (cm^2)
42	F	87	VLU	RLE med	1.4	0.8	0.1	1.12

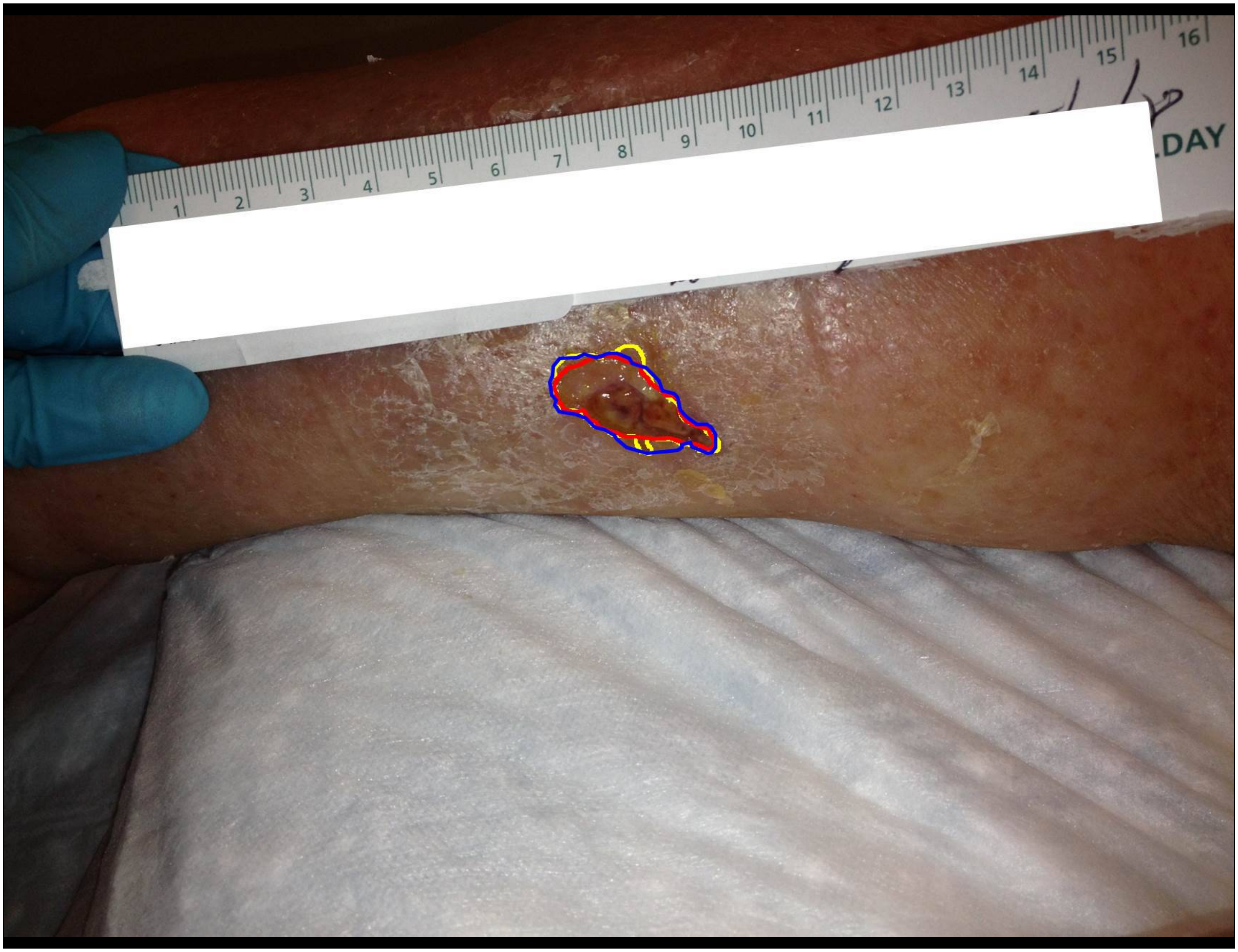

Tracing Data			
Tracer:	Wound Area (px^2)	Ruler Calibration (px/cm)	Wound Area (cm^2)
H1	17507	88.8	2.22
H2	15047	91.1	1.81
AI	19162	87.5	2.50

Tracing Comparisons				
Difference Metric:	Human-Human		Human-AI	
	H1(ref) H2(test)	H2(ref) H1(test)	H1(ref) AI(test)	H2(ref) AI(test)
False Negative Area (%)	15.2	1.4	4.7	0.2
False Positive Area (%)	1.2	17.7	14.1	27.5
|Relative Error (%)|	14.1	16.3	9.5	27.3

Blinded Attending Surgeon Review						
Reviewer	PGT Estimate	H1 meets definition?	H2 meets definition?	AI meets definition?	Which is AI?	Which is most accurate?
1	0	Yes	No	No	H1	H2
2	0	No	No	No	H2	AI
3	10	No	No	No	AI	H1

© 2021 Howell RS et al. *JAMA Network Open.*

Wound EMR Information								
Sequential Number	Gender	Age	Wound Type	Wound Location	Wound Length (cm)	Wound Width (cm)	Wound Depth (cm)	Wound Area (cm^2)
43	F	35	lymphedem	RLE	0.2	2.0	0.1	0.40

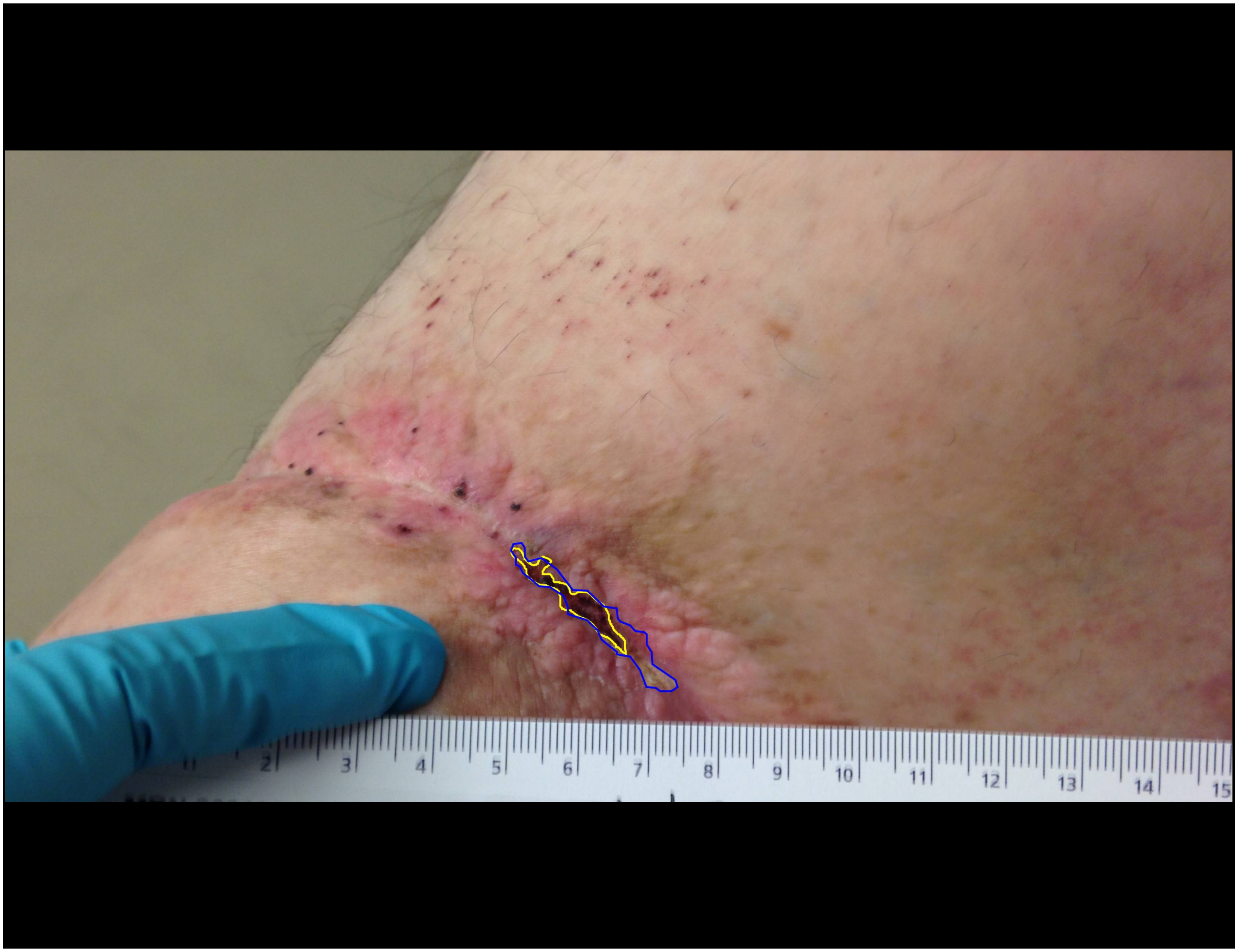

Tracing Data			
Tracer:	Wound Area (px^2)	Ruler Calibration (px/cm)	Wound Area (cm^2)
H1	15493	196.8	0.40
H2	0		0.00
AI	31854	188.1	0.90

Tracing Comparisons				
Difference Metric:	Human-Human		Human-AI	
	H1(ref) H2(test)	H2(ref) H1(test)	H1(ref) AI(test)	H2(ref) AI(test)
False Negative Area (%)			5.7	
False Positive Area (%)			111.3	
|Relative Error (%)|			105.6	

Blinded Attending Surgeon Review						
Reviewer	PGT Estimate	H1 meets definition?	H2 meets definition?	AI meets definition?	Which is AI?	Which is most accurate?
1		Yes	Yes	Yes	H1	0
2		No	No	No	H2	H2
3	0	No	Yes	No	AI	H2

© 2021 Howell RS et al. *JAMA Network Open.*

Wound EMR Information								
Sequential Number	Gender	Age	Wound Type	Wound Location	Wound Length (cm)	Wound Width (cm)	Wound Depth (cm)	Wound Area (cm^2)
44	M	89	DFU	L 4th toe	1.2	1.3	0.1	1.56

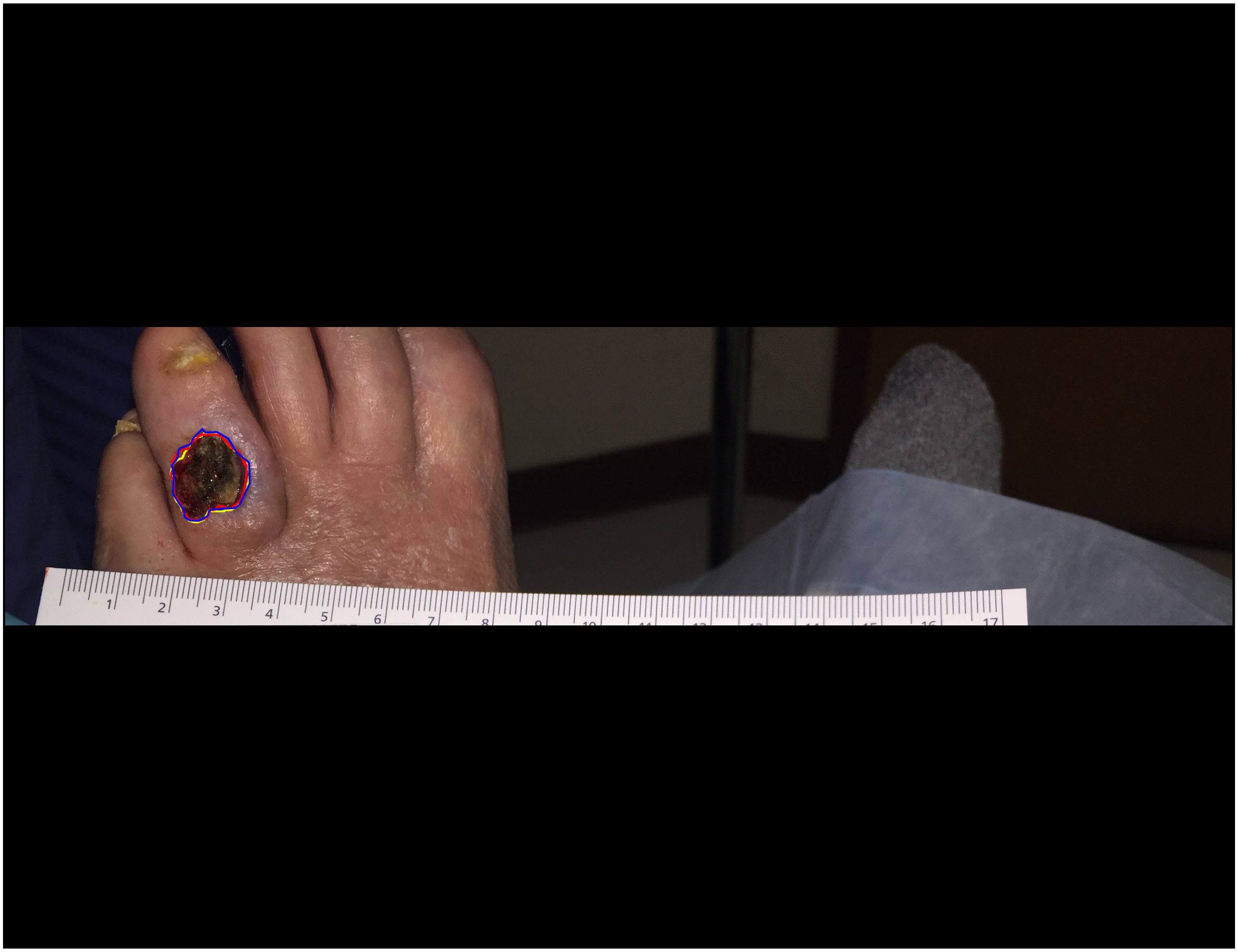

Tracing Data			
Tracer:	Wound Area (px^2)	Ruler Calibration (px/cm)	Wound Area (cm^2)
H1	34496	143.3	1.68
H2	33403	143.7	1.62
AI	35522	144.6	1.70

Tracing Comparisons				
Difference Metric:	Human-Human		Human-AI	
	H1(ref) H2(test)	H2(ref) H1(test)	H1(ref) AI(test)	H2(ref) AI(test)
False Negative Area (%)	5.7	2.6	2.4	1.7
False Positive Area (%)	2.5	5.9	5.3	8.0
|Relative Error (%)|	3.2	3.3	3.0	6.3

Blinded Attending Surgeon Review						
Reviewer	PGT Estimate	H1 meets definition?	H2 meets definition?	AI meets definition?	Which is AI?	Which is most accurate?
1	0	Yes	Yes	Yes	AI	H1
2	0	Yes	Yes	Yes	H1	AI
3	20	Yes	Yes	Yes	H1	AI

© 2021 Howell RS et al. *JAMA Network Open.*

Wound EMR Information								
Sequential Number	Gender	Age	Wound Type	Wound Location	Wound Length (cm)	Wound Width (cm)	Wound Depth (cm)	Wound Area (cm^2)
45	M	85	Trauma	LLE lat	7.6	3.0	0.1	22.80

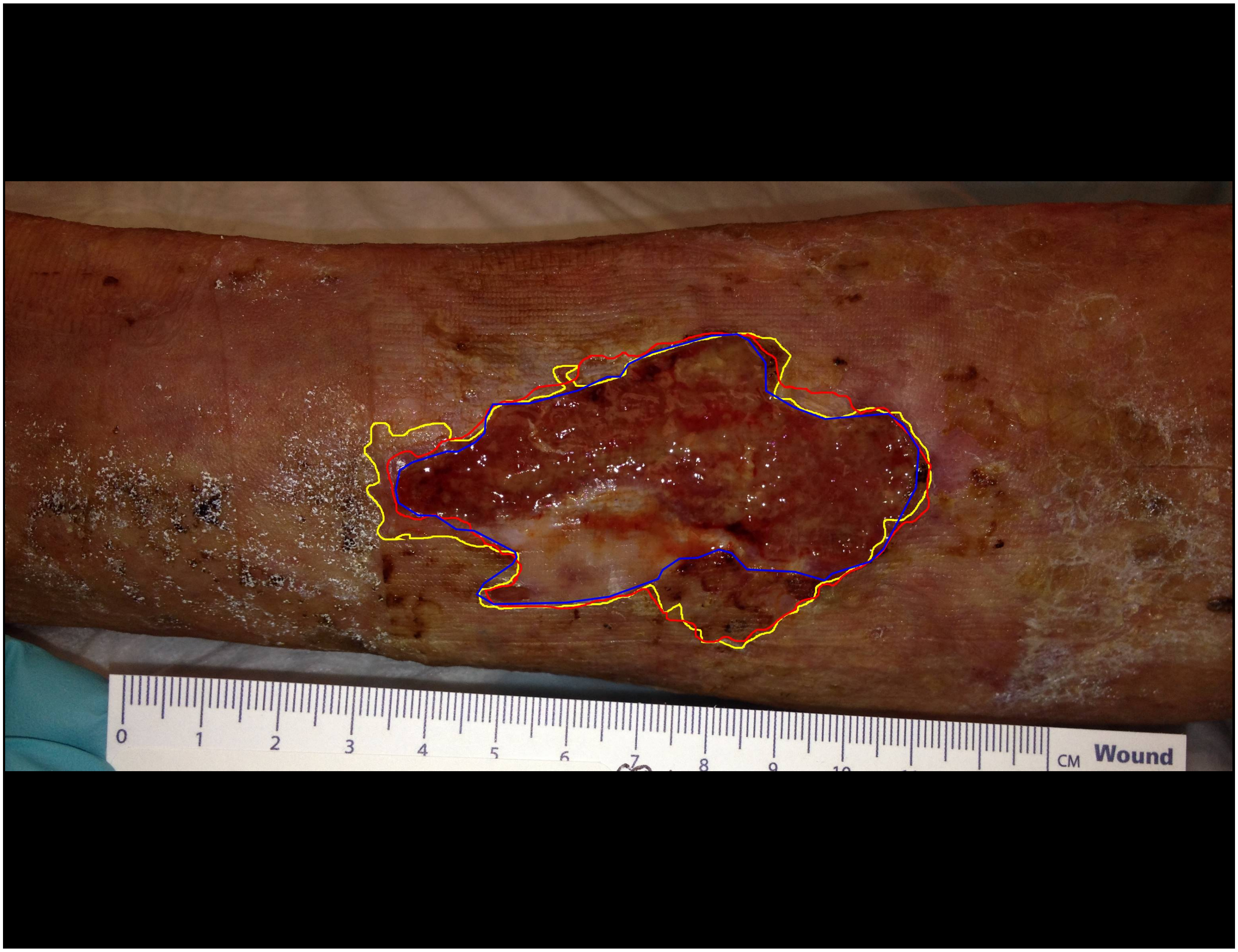

Tracing Data			
Tracer:	Wound Area (px^2)	Ruler Calibration (px/cm)	Wound Area (cm^2)
H1	749519	195.1	19.70
H2	734496	187.1	20.97
AI	613744	210.1	13.90

Tracing Comparisons				
Difference Metric:	Human-Human		Human-AI	
	H1(ref) H2(test)	H2(ref) H1(test)	H1(ref) AI(test)	H2(ref) AI(test)
False Negative Area (%)	6.8	4.9	19.1	17.3
False Positive Area (%)	4.8	7.0	1.0	0.8
|Relative Error (%)|	2.0	2.0	18.1	16.4

Blinded Attending Surgeon Review						
Reviewer	PGT Estimate	H1 meets definition?	H2 meets definition?	AI meets definition?	Which is AI?	Which is most accurate?
1	80	No	No	No	AI	None
2	20	No	No	No	AI	H2
3	80	Yes	Yes	Yes	H2	H2

© 2021 Howell RS et al. *JAMA Network Open.*

Wound EMR Information								
Sequential Number	Gender	Age	Wound Type	Wound Location	Wound Length (cm)	Wound Width (cm)	Wound Depth (cm)	Wound Area (cm^2)
46	F	65	PU	L buttock	7.0	6.5	5.4	45.50

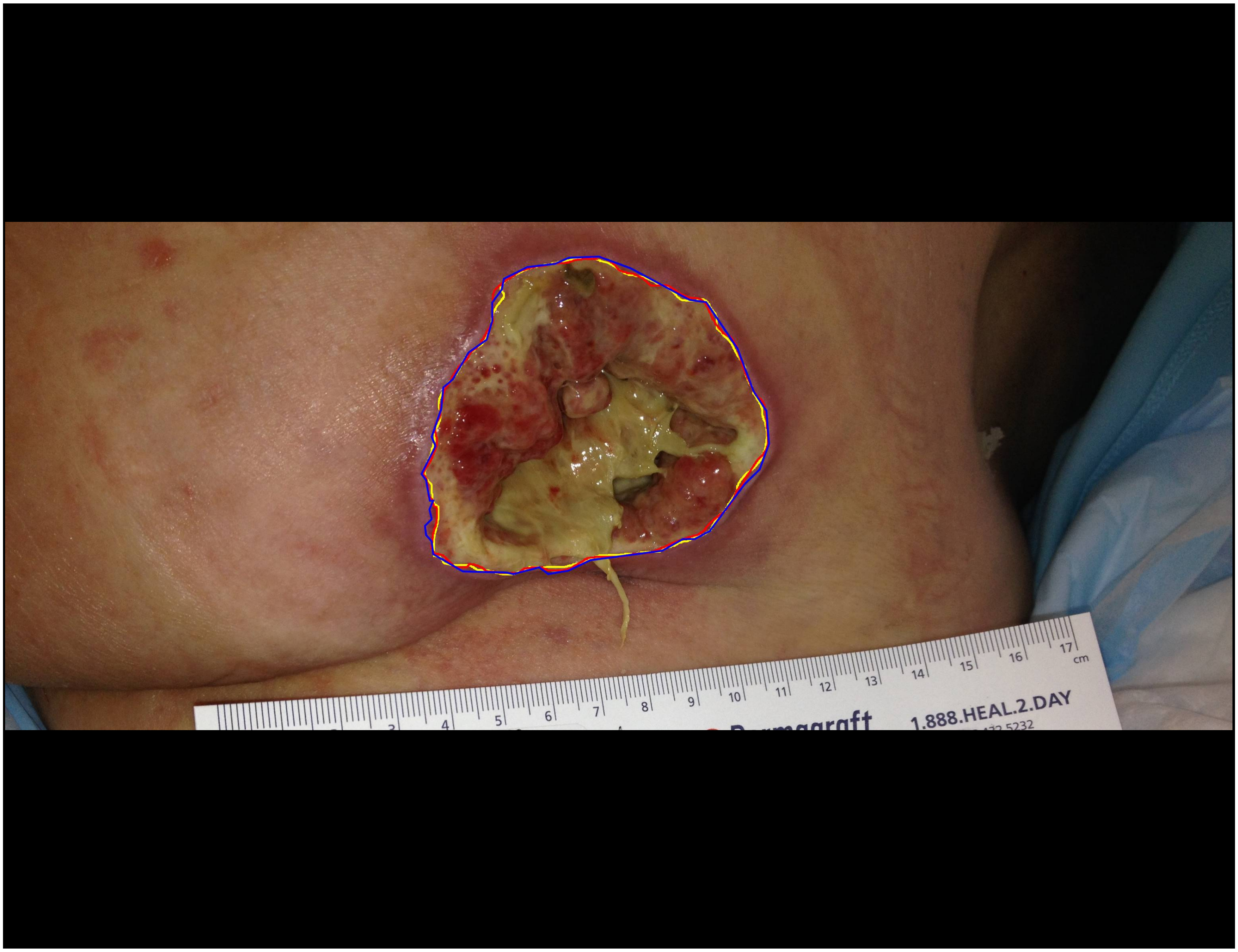

Tracing Data			
Tracer:	Wound Area (px^2)	Ruler Calibration (px/cm)	Wound Area (cm^2)
H1	566235	127.1	35.04
H2	568990	131.0	33.16
AI	579463	148.4	26.30

Tracing Comparisons				
Difference Metric:	Human-Human		Human-AI	
	H1(ref) H2(test)	H2(ref) H1(test)	H1(ref) AI(test)	H2(ref) AI(test)
False Negative Area (%)	1.0	1.4	0.3	0.4
False Positive Area (%)	1.4	1.0	2.6	2.2
|Relative Error (%)|	0.5	0.5	2.3	1.8

Blinded Attending Surgeon Review						
Reviewer	PGT Estimate	H1 meets definition?	H2 meets definition?	AI meets definition?	Which is AI?	Which is most accurate?
1	10	Yes	Yes	Yes	H2	AI
2	0	Yes	Yes	Yes	AI	AI
3	70	Yes	Yes	Yes	H2	H1

© 2021 Howell RS et al. *JAMA Network Open.*

Wound EMR Information								
Sequential Number	Gender	Age	Wound Type	Wound Location	Wound Length (cm)	Wound Width (cm)	Wound Depth (cm)	Wound Area (cm^2)
47	F	82	VLU	LLE ant	0.0	0.0	0.0	0.00

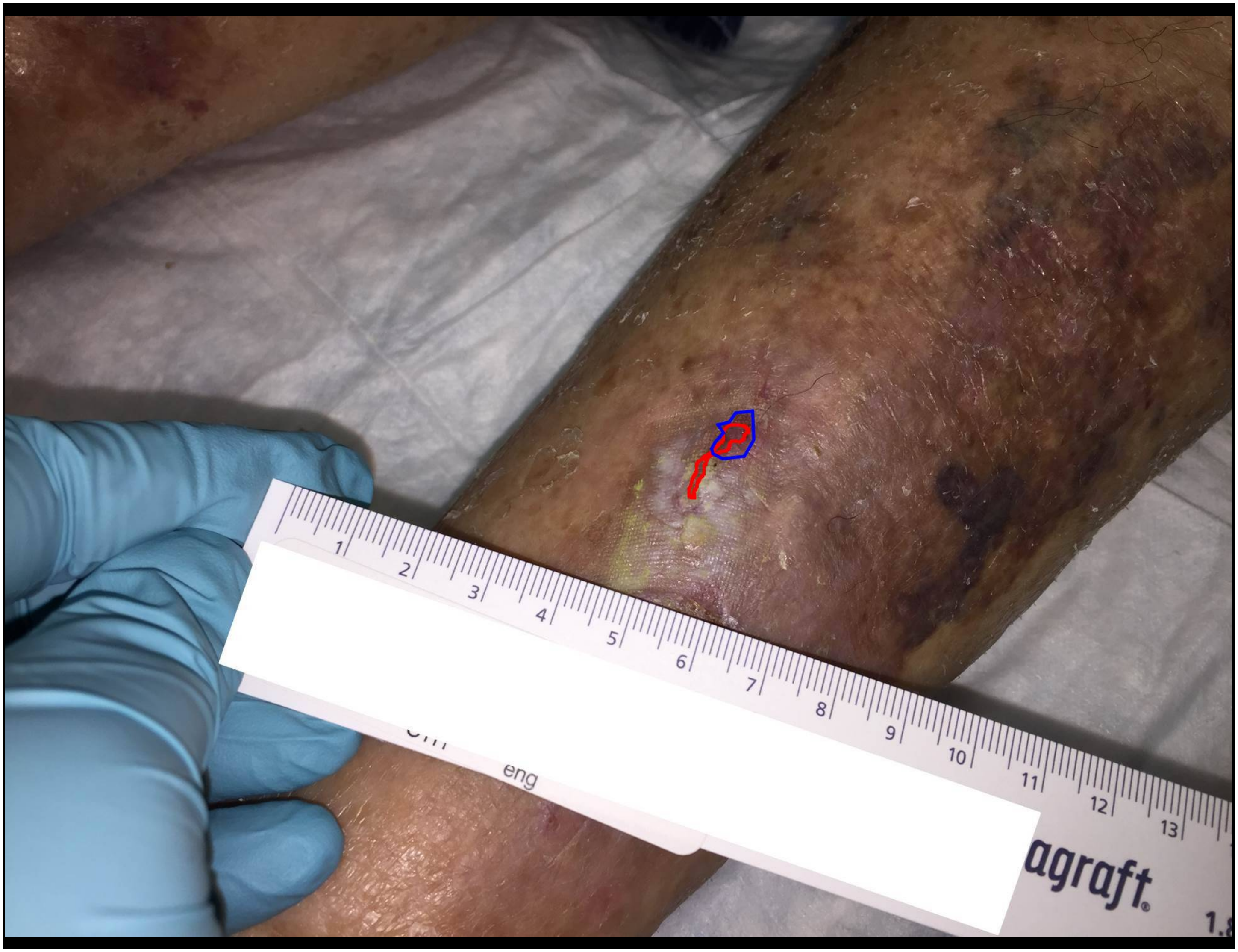

Tracing Data			
Tracer:	Wound Area (px^2)	Ruler Calibration (px/cm)	Wound Area (cm^2)
H1	0		0.00
H2	1926	115.7	0.14
AI	3497	108.0	0.30

Tracing Comparisons				
Difference Metric:	Human-Human		Human-AI	
	H1(ref) H2(test)	H2(ref) H1(test)	H1(ref) AI(test)	H2(ref) AI(test)
False Negative Area (%)				37.7
False Positive Area (%)				119.3
|Relative Error (%)|				81.6

Blinded Attending Surgeon Review						
Reviewer	PGT Estimate	H1 meets definition?	H2 meets definition?	AI meets definition?	Which is AI?	Which is most accurate?
1		Yes	Yes	Yes	H2	0
2	healed	Yes	No	No	AI	AI
3	0	Yes	No	No	H1	AI

© 2021 Howell RS et al. *JAMA Network Open.*

Wound EMR Information								
Sequential Number	Gender	Age	Wound Type	Wound Location	Wound Length (cm)	Wound Width (cm)	Wound Depth (cm)	Wound Area (cm^2)
48	F	69	PU	L heel	1.9	2.0		3.80

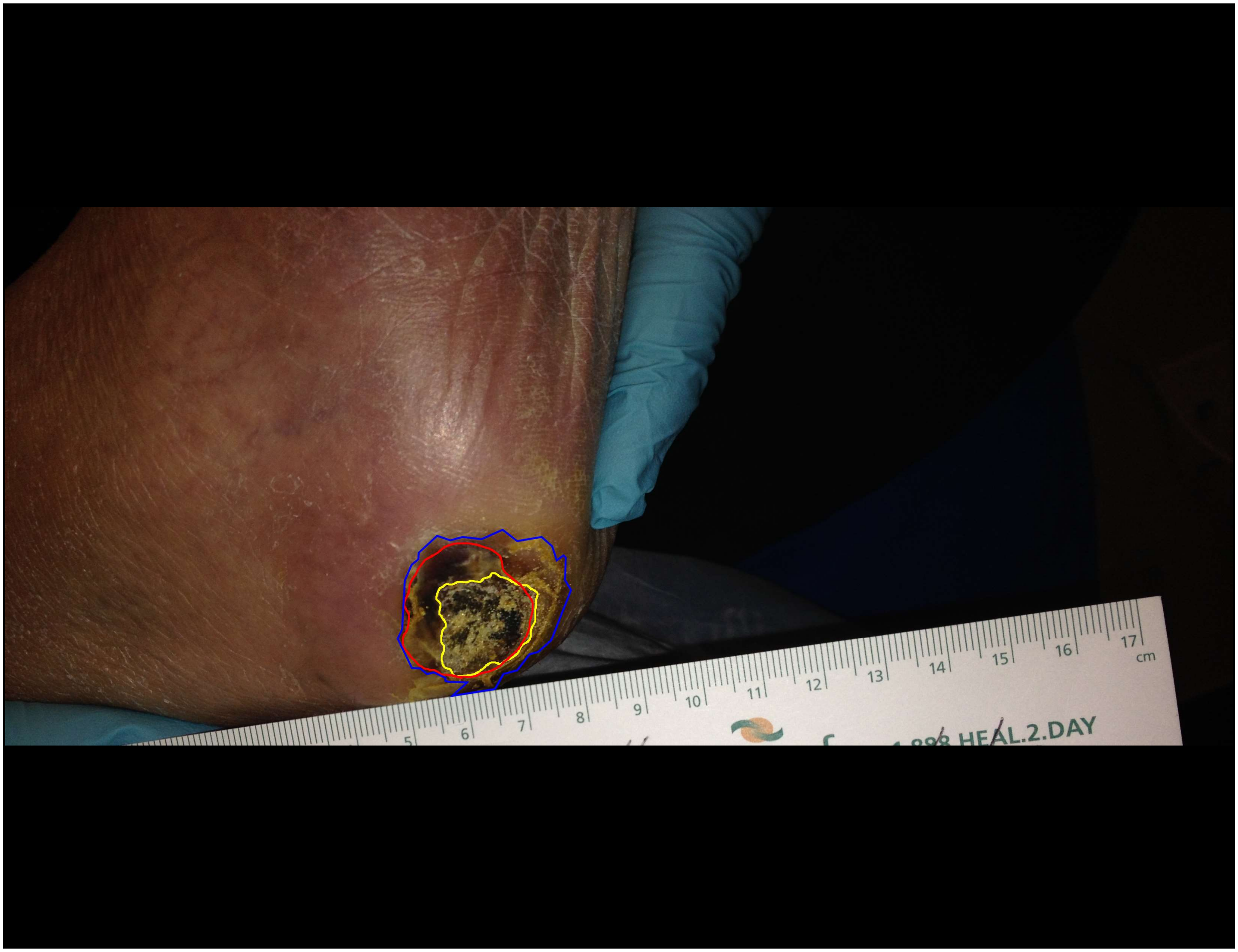

Tracing Data			
Tracer:	Wound Area (px^2)	Ruler Calibration (px/cm)	Wound Area (cm^2)
H1	53917	155.8	2.22
H2	93147	155.4	3.86
AI	145425	157.0	5.90

Tracing Comparisons				
Difference Metric:	Human-Human		Human-AI	
	H1(ref) H2(test)	H2(ref) H1(test)	H1(ref) AI(test)	H2(ref) AI(test)
False Negative Area (%)	4.2	44.6	0.0	0.0
False Positive Area (%)	77.0	2.5	169.7	56.1
|Relative Error (%)|	72.8	42.1	169.7	56.1

Blinded Attending Surgeon Review						
Reviewer	PGT Estimate	H1 meets definition?	H2 meets definition?	AI meets definition?	Which is AI?	Which is most accurate?
1	0	No	No	Yes	H2	AI
2	0	No	Yes	No	AI	H1
3	20	Yes	Yes	No	H1	H2

© 2021 Howell RS et al. *JAMA Network Open.*

Wound EMR Information								
Sequential Number	Gender	Age	Wound Type	Wound Location	Wound Length (cm)	Wound Width (cm)	Wound Depth (cm)	Wound Area (cm^2)
49	M	69	DFU	R plantar	1.0	1.0	0.1	1.00

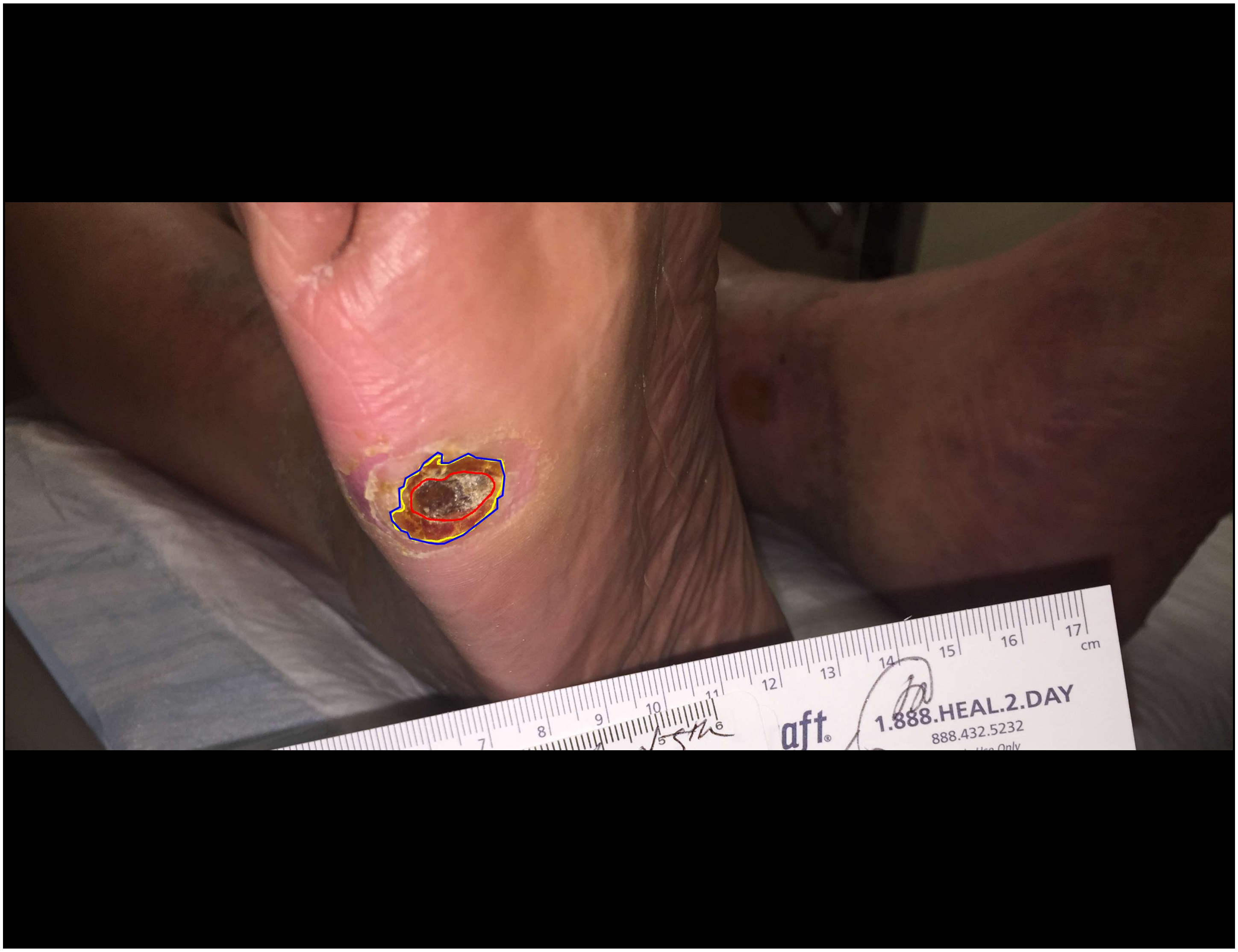

Tracing Data			
Tracer:	Wound Area (px^2)	Ruler Calibration (px/cm)	Wound Area (cm^2)
H1	48169	160.1	1.88
H2	21199	161.1	0.82
AI	51313	160.2	2.00

Tracing Comparisons				
Difference Metric:	Human-Human		Human-AI	
	H1(ref) H2(test)	H2(ref) H1(test)	H1(ref) AI(test)	H2(ref) AI(test)
False Negative Area (%)	56.0	0.0	0.5	0.0
False Positive Area (%)	0.0	127.2	7.1	142.0
|Relative Error (%)|	56.0	127.2	6.5	142.1

Blinded Attending Surgeon Review						
Reviewer	PGT Estimate	H1 meets definition?	H2 meets definition?	AI meets definition?	Which is AI?	Which is most accurate?
1	0	No	Yes	No	H2	H2
2	0	No	Yes	No	H1	H2
3	30	No	Yes	No	H1	H2

© 2021 Howell RS et al. *JAMA Network Open.*

Wound EMR Information								
Sequential Number	Gender	Age	Wound Type	Wound Location	Wound Length (cm)	Wound Width (cm)	Wound Depth (cm)	Wound Area (cm^2)
50	M	70	DFU	L plantar	1.3	1.0	0.1	1.30

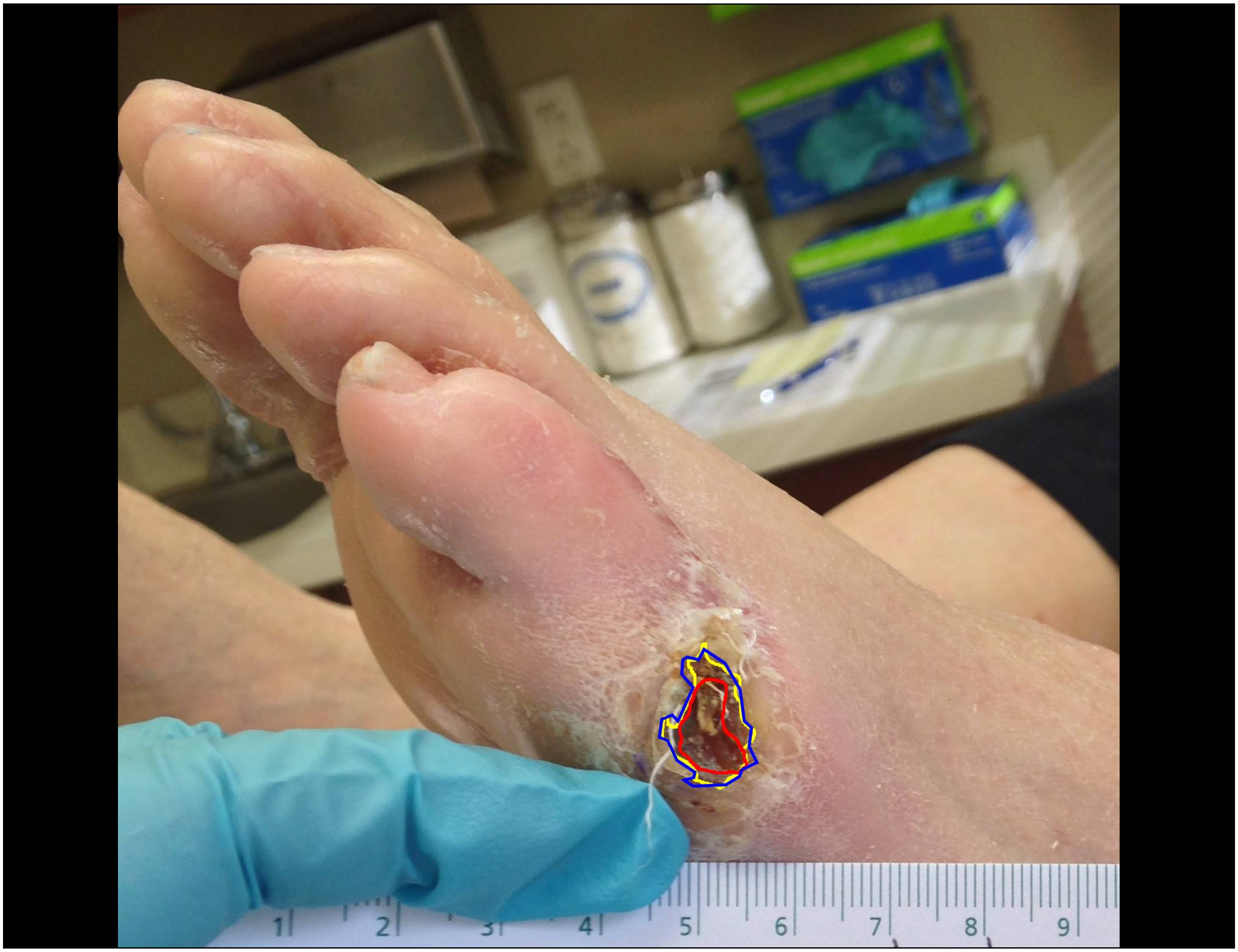

Tracing Data			
Tracer:	Wound Area (px^2)	Ruler Calibration (px/cm)	Wound Area (cm^2)
H1	19416	157.8	0.78
H2	10878	160.7	0.42
AI	22188	157.0	0.90

Tracing Comparisons				
Difference Metric:	Human-Human		Human-AI	
	H1(ref) H2(test)	H2(ref) H1(test)	H1(ref) AI(test)	H2(ref) AI(test)
False Negative Area (%)	44.2	0.4	1.9	0.0
False Positive Area (%)	0.2	78.9	16.2	104.0
|Relative Error (%)|	44.0	78.5	14.3	104.0

Blinded Attending Surgeon Review						
Reviewer	PGT Estimate	H1 meets definition?	H2 meets definition?	AI meets definition?	Which is AI?	Which is most accurate?
1	0	No	Yes	No	H1	H1
2	0	No	No	Yes	H2	AI
3	0	Yes	No	Yes	AI	H2

© 2021 Howell RS et al. *JAMA Network Open.*

Wound EMR Information								
Sequential Number	Gender	Age	Wound Type	Wound Location	Wound Length (cm)	Wound Width (cm)	Wound Depth (cm)	Wound Area (cm^2)
51	F	77	Trauma	RLE	3.9	3.5	0.2	13.65

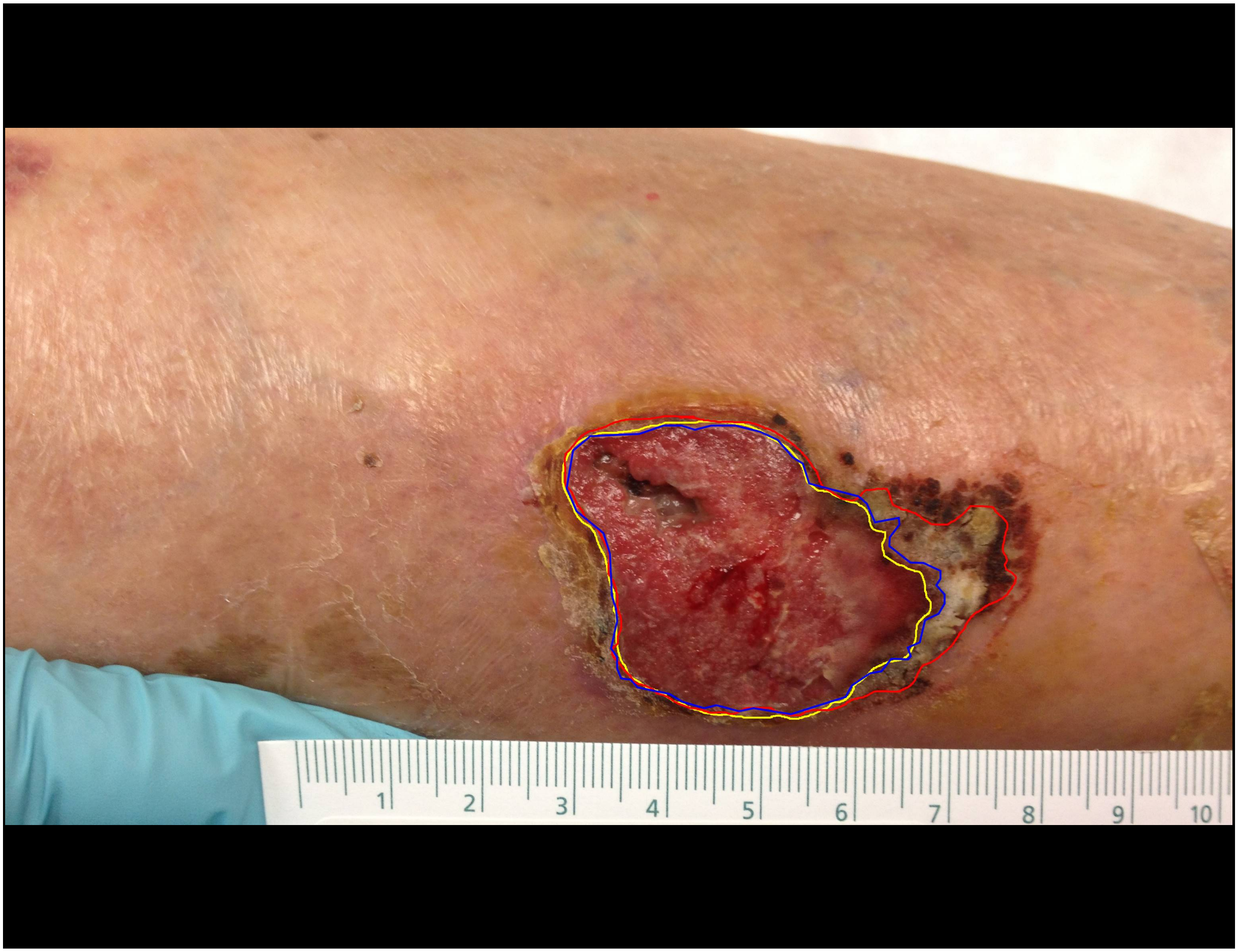

Tracing Data			
Tracer:	Wound Area (px^2)	Ruler Calibration (px/cm)	Wound Area (cm^2)
H1	532921	249.4	8.57
H2	640841	250.7	10.19
AI	533531	243.5	9.00

Tracing Comparisons				
Difference Metric:	Human-Human		Human-AI	
	H1(ref) H2(test)	H2(ref) H1(test)	H1(ref) AI(test)	H2(ref) AI(test)
False Negative Area (%)	1.7	18.3	3.2	17.6
False Positive Area (%)	22.0	1.5	3.4	0.8
|Relative Error (%)|	20.3	16.8	0.1	16.7

Blinded Attending Surgeon Review						
Reviewer	PGT Estimate	H1 meets definition?	H2 meets definition?	AI meets definition?	Which is AI?	Which is most accurate?
1	70	No	Yes	No	H1	AI
2	100	No	No	No	AI	H1
3	90	Yes	No	Yes	H2	H1

© 2021 Howell RS et al. *JAMA Network Open.*

Wound EMR Information								
Sequential Number	Gender	Age	Wound Type	Wound Location	Wound Length (cm)	Wound Width (cm)	Wound Depth (cm)	Wound Area (cm^2)
52	F	62	VLU	RLE med	1.8	2.0	0.2	3.60

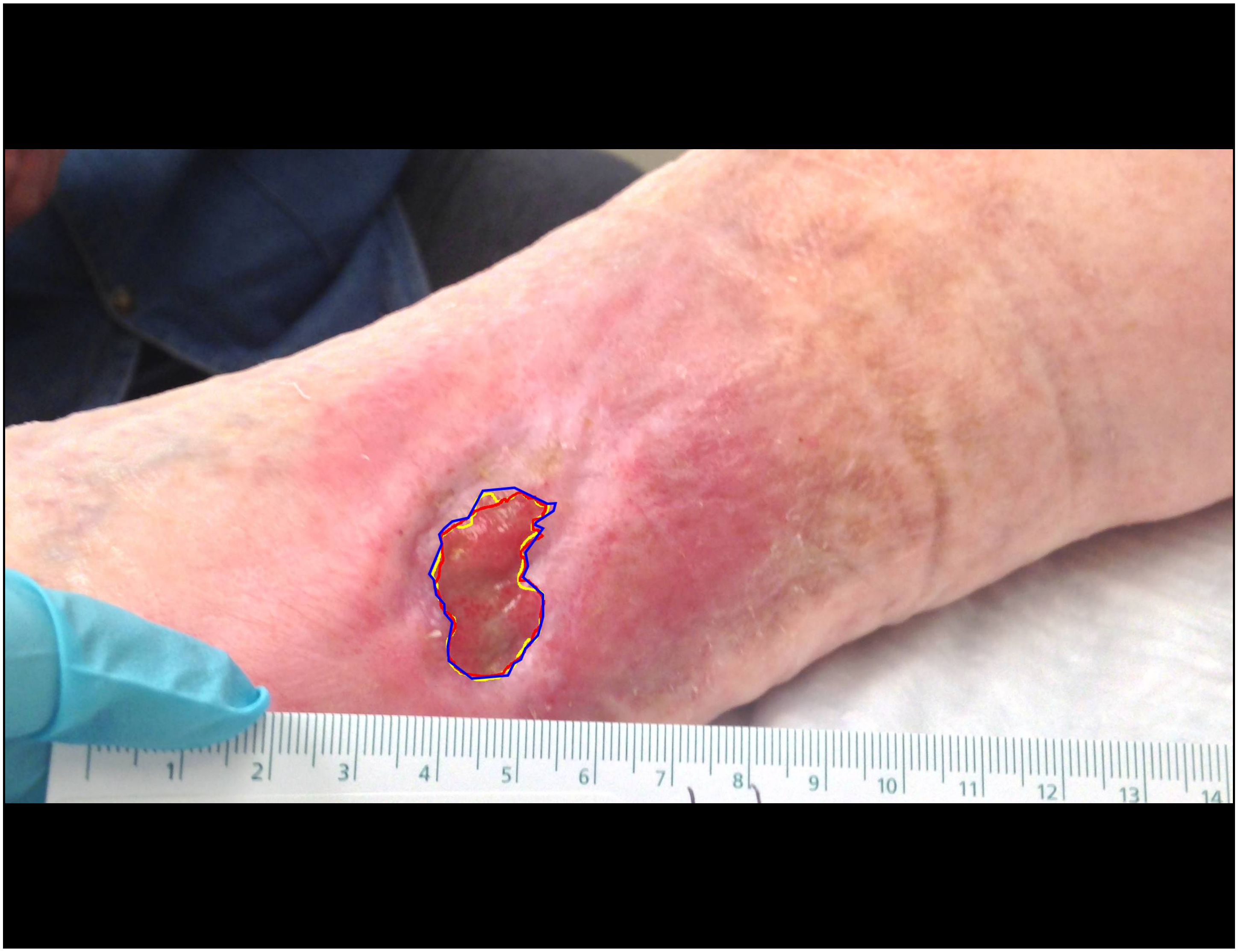

Tracing Data			
Tracer:	Wound Area (px^2)	Ruler Calibration (px/cm)	Wound Area (cm^2)
H1	57333	159.3	2.26
H2	56712	157.1	2.30
AI	62831	169.0	2.20

Tracing Comparisons				
Difference Metric:	Human-Human		Human-AI	
	H1(ref) H2(test)	H2(ref) H1(test)	H1(ref) AI(test)	H2(ref) AI(test)
False Negative Area (%)	5.0	4.0	0.8	0.5
False Positive Area (%)	3.9	5.1	10.4	11.3
|Relative Error (%)|	1.1	1.1	9.6	10.8

Blinded Attending Surgeon Review						
Reviewer	PGT Estimate	H1 meets definition?	H2 meets definition?	AI meets definition?	Which is AI?	Which is most accurate?
1	90	Yes	No	No	AI	H2
2		Yes	Yes	Yes	H2	H1
3	70	Yes	Yes	Yes	H1	H2

© 2021 Howell RS et al. *JAMA Network Open.*

Wound EMR Information								
Sequential Number	Gender	Age	Wound Type	Wound Location	Wound Length (cm)	Wound Width (cm)	Wound Depth (cm)	Wound Area (cm^2)
53	F	79	VLU	LLE	6.0	5.5	0.2	33.00

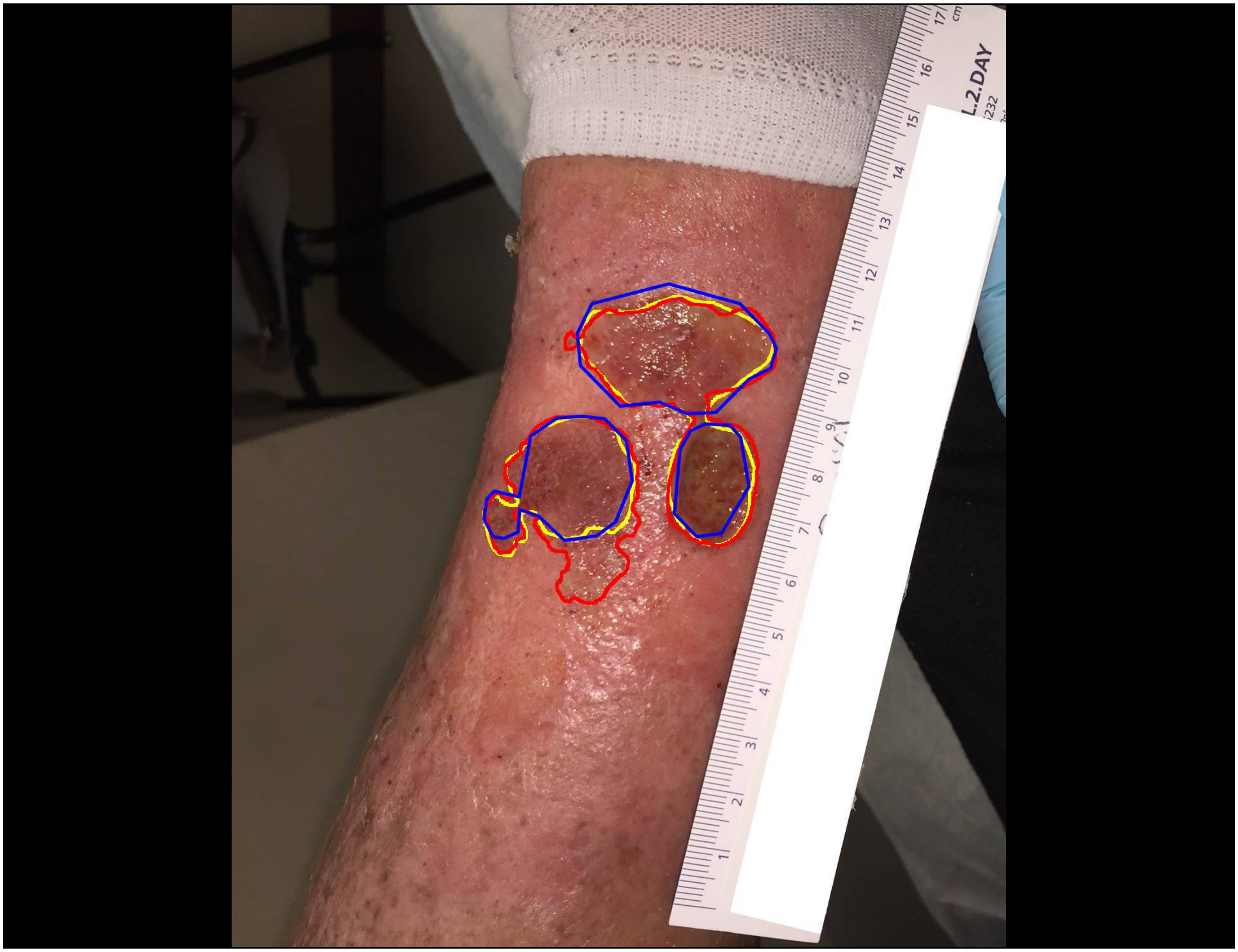

Tracing Data			
Tracer:	Wound Area (px^2)	Ruler Calibration (px/cm)	Wound Area (cm^2)
H1	91865	85.9	12.46
H2	106260	83.9	15.09
AI	91450	81.7	13.70

Tracing Comparisons				
Difference Metric:	Human-Human		Human-AI	
	H1(ref) H2(test)	H2(ref) H1(test)	H1(ref) AI(test)	H2(ref) AI(test)
False Negative Area (%)	3.1	16.3	10.5	21.7
False Positive Area (%)	18.8	2.7	10.1	7.8
|Relative Error (%)|	15.7	13.5	0.5	13.9

Blinded Attending Surgeon Review						
Reviewer	PGT Estimate	H1 meets definition?	H2 meets definition?	AI meets definition?	Which is AI?	Which is most accurate?
1	0	No	Yes	No	AI	H2
2	0	Yes	No	No	H1	H2
3	30	Yes	Yes	Yes	H1	H2

© 2021 Howell RS et al. *JAMA Network Open.*

Wound EMR Information								
Sequential Number	Gender	Age	Wound Type	Wound Location	Wound Length (cm)	Wound Width (cm)	Wound Depth (cm)	Wound Area (cm^2)
54	F	89	VLU	RLE lat	5.0	5.6	0.1	28.00

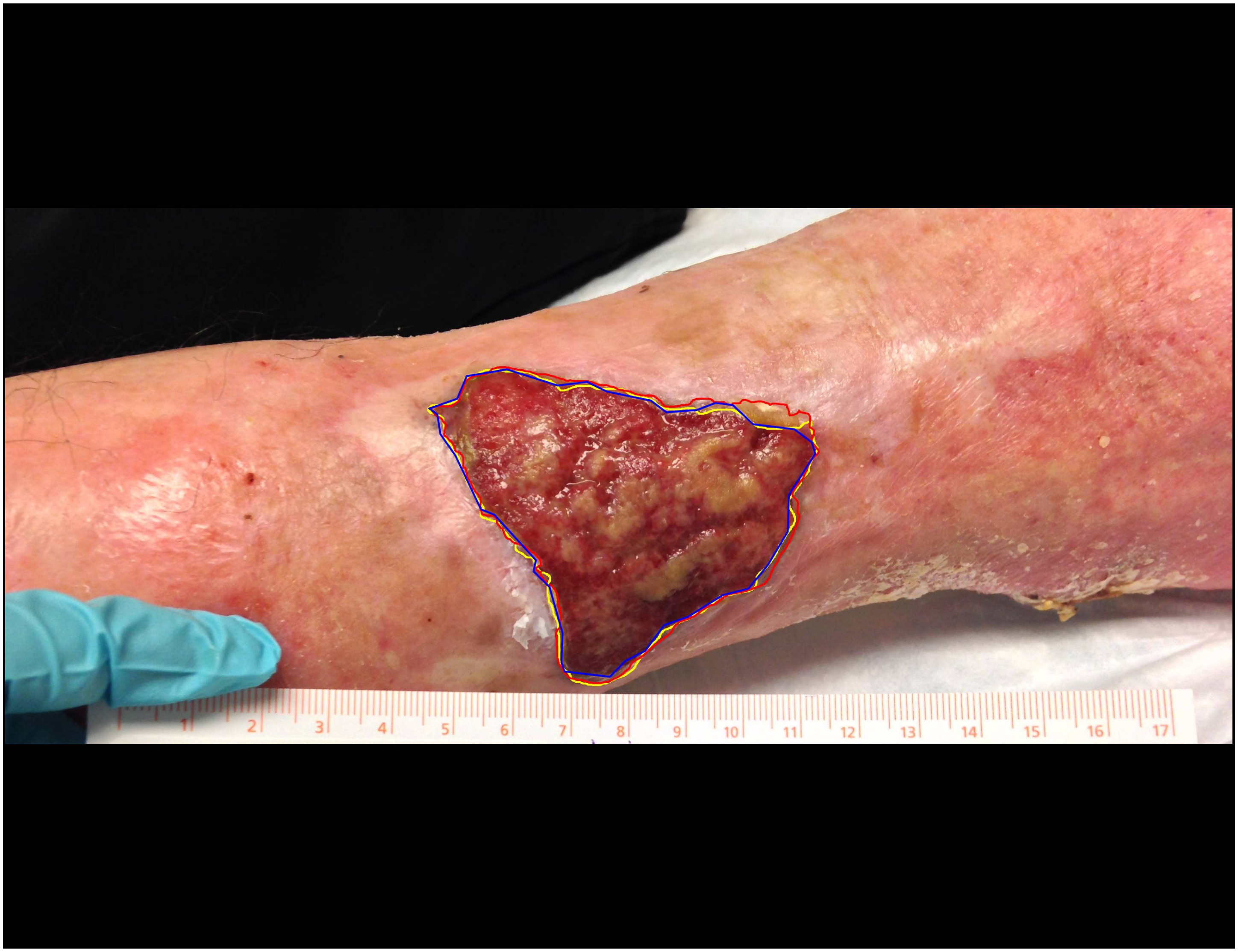

Tracing Data			
Tracer:	Wound Area (px^2)	Ruler Calibration (px/cm)	Wound Area (cm^2)
H1	494265	156.0	20.31
H2	511730	153.3	21.76
AI	482588	170.0	16.70

Tracing Comparisons				
Difference Metric:	Human-Human		Human-AI	
	H1(ref) H2(test)	H2(ref) H1(test)	H1(ref) AI(test)	H2(ref) AI(test)
False Negative Area (%)	1.6	5.0	3.5	6.7
False Positive Area (%)	5.2	1.6	1.1	1.1
|Relative Error (%)|	3.5	3.4	2.4	5.7

Blinded Attending Surgeon Review						
Reviewer	PGT Estimate	H1 meets definition?	H2 meets definition?	AI meets definition?	Which is AI?	Which is most accurate?
1	40	Yes	Yes	Yes	AI	AI
2	70	Yes	No	Yes	H2	AI
3	80	Yes	No	Yes	H2	H2

© 2021 Howell RS et al. *JAMA Network Open.*

Wound EMR Information								
Sequential Number	Gender	Age	Wound Type	Wound Location	Wound Length (cm)	Wound Width (cm)	Wound Depth (cm)	Wound Area (cm^2)
55	M	65	DFU	L plantar	3.1	3.9	0.3	12.09

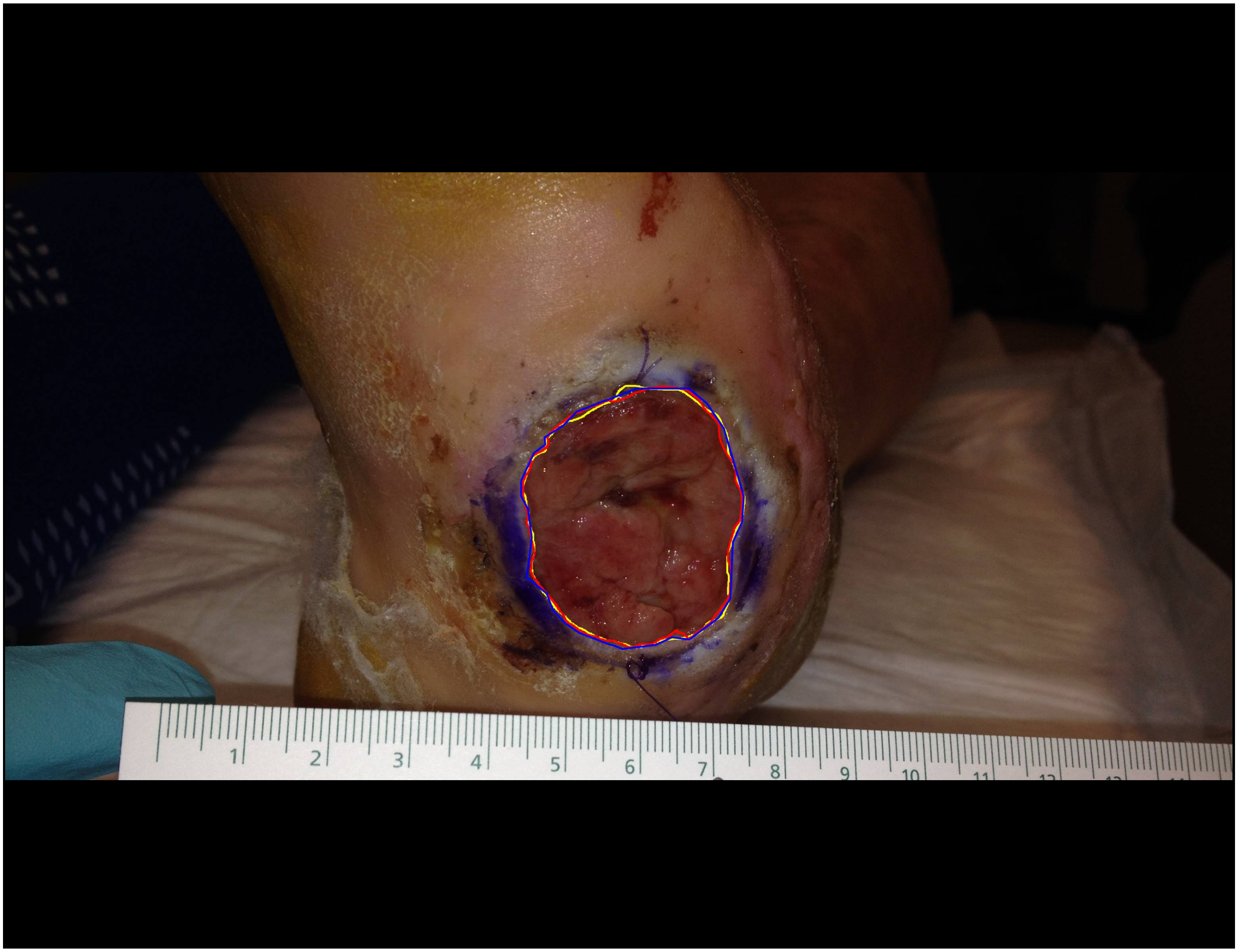

Tracing Data			
Tracer:	Wound Area (px^2)	Ruler Calibration (px/cm)	Wound Area (cm^2)
H1	314343	198.8	7.95
H2	312188	197.4	8.01
AI	323566	206.3	7.60

Tracing Comparisons				
Difference Metric:	Human-Human		Human-AI	
	H1(ref) H2(test)	H2(ref) H1(test)	H1(ref) AI(test)	H2(ref) AI(test)
False Negative Area (%)	2.2	1.5	0.5	0.4
False Positive Area (%)	1.5	2.2	3.5	4.0
|Relative Error (%)|	0.7	0.7	2.9	3.6

Blinded Attending Surgeon Review						
Reviewer	PGT Estimate	H1 meets definition?	H2 meets definition?	AI meets definition?	Which is AI?	Which is most accurate?
1	40	No	No	Yes	H2	H1
2	0	Yes	Yes	Yes	H1	AI
3	100	Yes	Yes	Yes	H1	AI

© 2021 Howell RS et al. *JAMA Network Open.*

Wound EMR Information								
Sequential Number	Gender	Age	Wound Type	Wound Location	Wound Length (cm)	Wound Width (cm)	Wound Depth (cm)	Wound Area (cm^2)
56	F	82	PU	L buttock	2.9	2.4	0.5	6.96

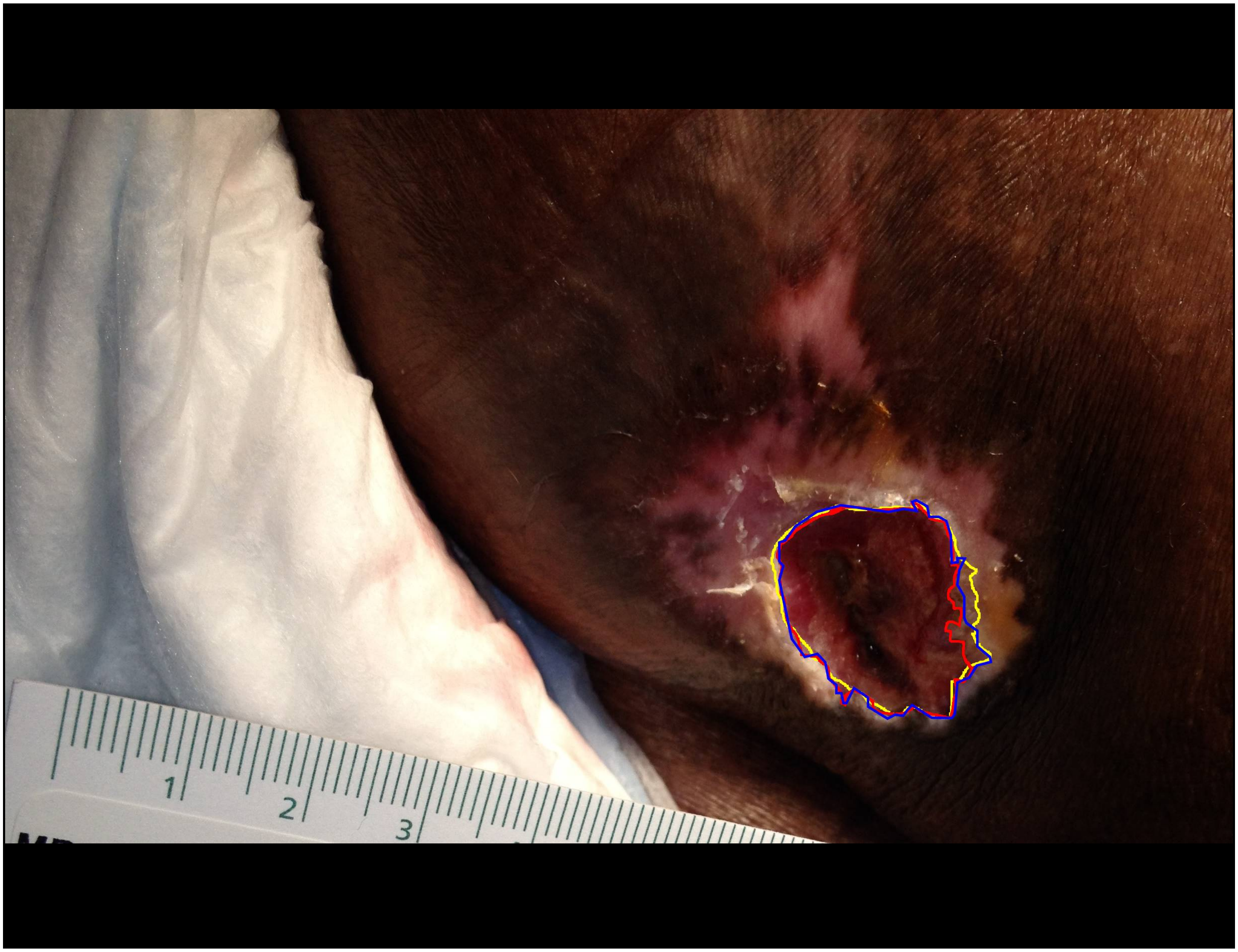

Tracing Data			
Tracer:	Wound Area (px^2)	Ruler Calibration (px/cm)	Wound Area (cm^2)
H1	130449	224.9	2.58
H2	120504	225.9	2.36
AI	128792	267.5	1.80

Tracing Comparisons				
Difference Metric:	Human-Human		Human-AI	
	H1(ref) H2(test)	H2(ref) H1(test)	H1(ref) AI(test)	H2(ref) AI(test)
False Negative Area (%)	9.6	2.1	4.9	0.6
False Positive Area (%)	2.0	10.4	3.6	7.5
|Relative Error (%)|	7.6	8.3	1.3	6.9

Blinded Attending Surgeon Review						
Reviewer	PGT Estimate	H1 meets definition?	H2 meets definition?	AI meets definition?	Which is AI?	Which is most accurate?
1		Yes	No	No	H1	AI
2	<10	No	Yes	Yes	AI	AI
3	80	Yes	Yes	Yes	H2	AI

© 2021 Howell RS et al. *JAMA Network Open.*

Wound EMR Information								
Sequential Number	Gender	Age	Wound Type	Wound Location	Wound Length (cm)	Wound Width (cm)	Wound Depth (cm)	Wound Area (cm^2)
57	F	80	DFU	R hallux	0.5	0.4		0.20

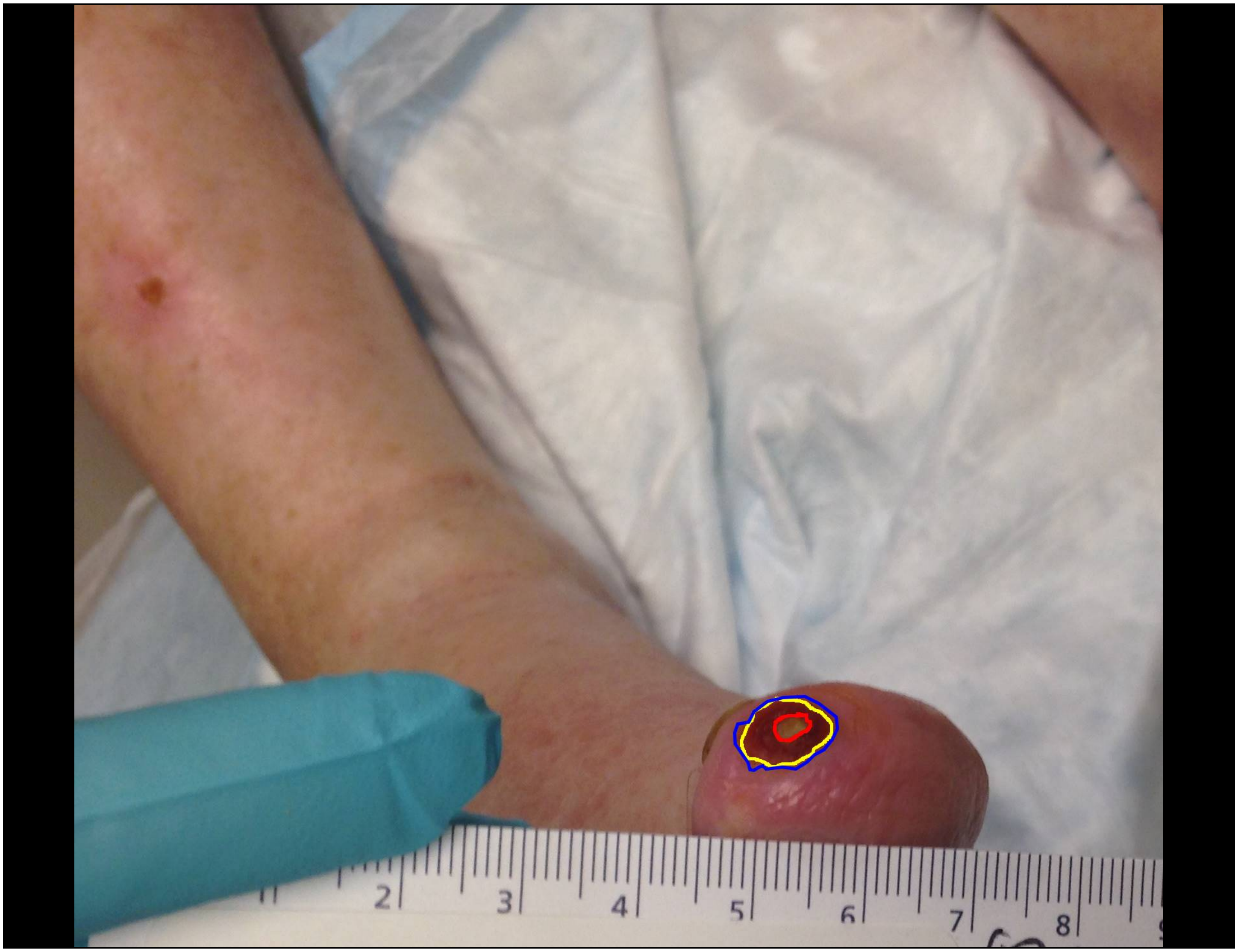

Tracing Data			
Tracer:	Wound Area (px^2)	Ruler Calibration (px/cm)	Wound Area (cm^2)
H1	10556	176.2	0.34
H2	1620	174.8	0.05
AI	13319	163.2	0.50

Tracing Comparisons				
Difference Metric:	Human-Human		Human-AI	
	H1(ref) H2(test)	H2(ref) H1(test)	H1(ref) AI(test)	H2(ref) AI(test)
False Negative Area (%)	84.6	0.0	0.1	0.0
False Positive Area (%)	0.0	551.5	26.3	722.1
|Relative Error (%)|	84.7	551.6	26.2	722.2

Blinded Attending Surgeon Review						
Reviewer	PGT Estimate	H1 meets definition?	H2 meets definition?	AI meets definition?	Which is AI?	Which is most accurate?
1	0	No	Yes	No	H2	H2
2	10	No	No	Yes	H2	H1
3	0	Yes	No	Yes	AI	H1

© 2021 Howell RS et al. *JAMA Network Open.*

Wound EMR Information								
Sequential Number	Gender	Age	Wound Type	Wound Location	Wound Length (cm)	Wound Width (cm)	Wound Depth (cm)	Wound Area (cm^2)
58	M	64	VLU	LLE ant	4.5	5.5	0.1	24.75

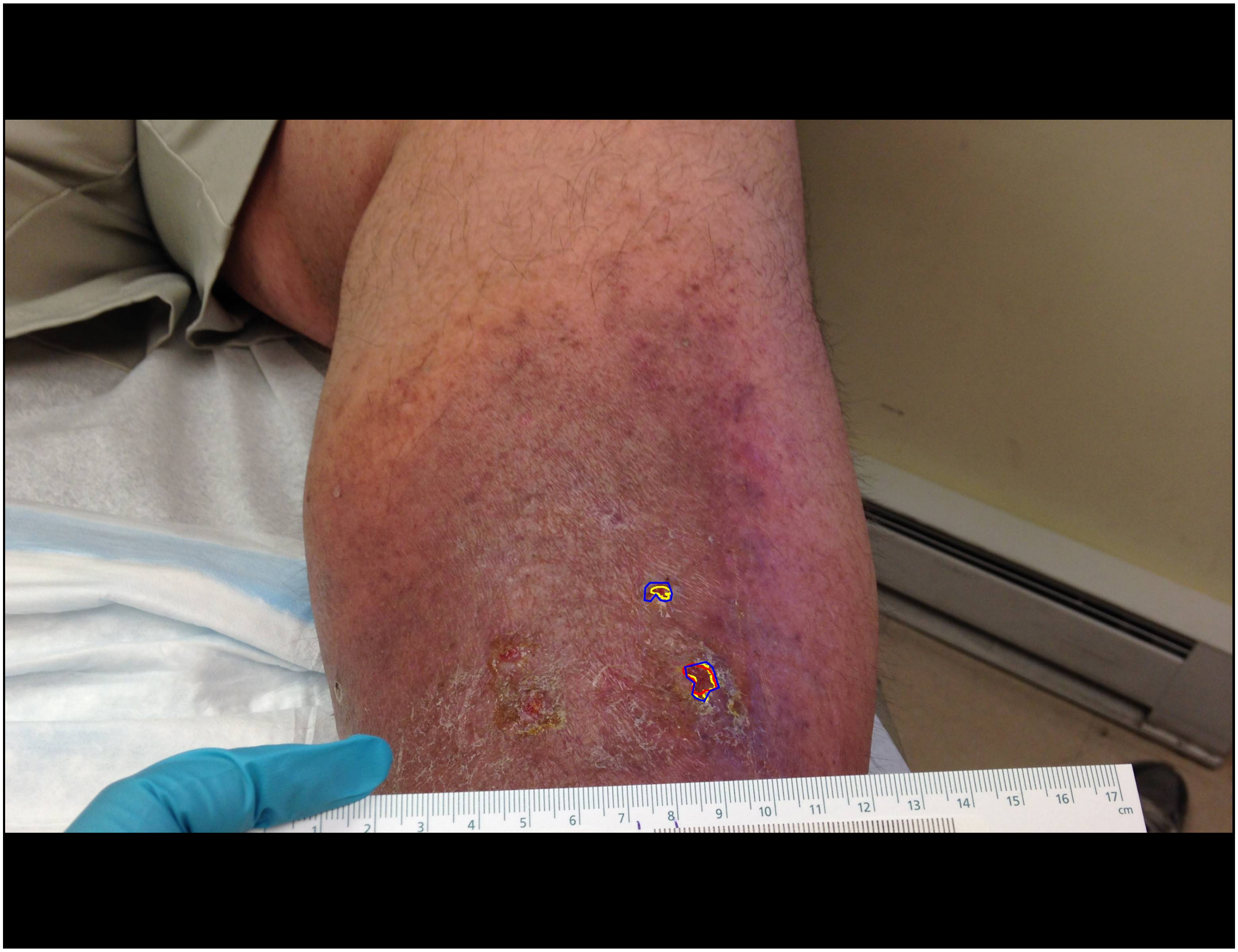

Tracing Data			
Tracer:	Wound Area (px^2)	Ruler Calibration (px/cm)	Wound Area (cm^2)
H1	5450	128.5	0.33
H2	4261	105.8	0.38
AI	8694	111.4	0.70

Tracing Comparisons				
Difference Metric:	Human-Human		Human-AI	
	H1(ref) H2(test)	H2(ref) H1(test)	H1(ref) AI(test)	H2(ref) AI(test)
False Negative Area (%)	31.6	12.5	3.1	5.1
False Positive Area (%)	9.8	40.4	62.6	109.1
|Relative Error (%)|	21.8	27.9	59.5	104.0

Blinded Attending Surgeon Review						
Reviewer	PGT Estimate	H1 meets definition?	H2 meets definition?	AI meets definition?	Which is AI?	Which is most accurate?
1	100	Yes	No	No	H1	H1
2	90	No	No	No	H2	AI
3	20	Yes	Yes	No	AI	H1

© 2021 Howell RS et al. *JAMA Network Open.*

Wound EMR Information								
Sequential Number	Gender	Age	Wound Type	Wound Location	Wound Length (cm)	Wound Width (cm)	Wound Depth (cm)	Wound Area (cm^2)
59	F	60	VLU	LLE medial	10.0	4.0	0.2	40.00

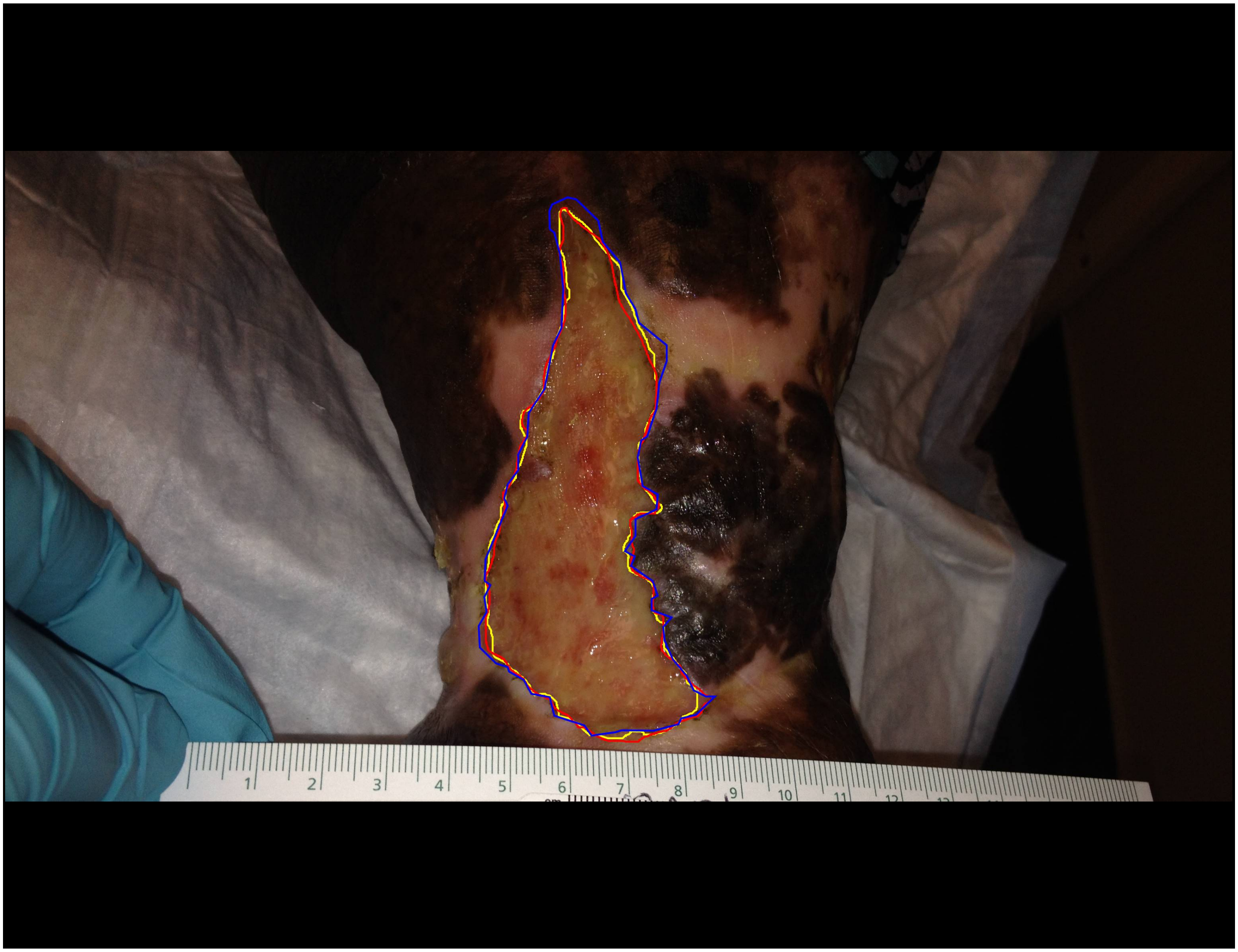

Tracing Data			
Tracer:	Wound Area (px^2)	Ruler Calibration (px/cm)	Wound Area (cm^2)
H1	431293	158.1	17.25
H2	431895	152.1	18.68
AI	454928	175.9	14.70

Tracing Comparisons				
Difference Metric:	Human-Human		Human-AI	
	H1(ref) H2(test)	H2(ref) H1(test)	H1(ref) AI(test)	H2(ref) AI(test)
False Negative Area (%)	2.7	2.8	1.3	1.8
False Positive Area (%)	2.8	2.7	6.8	7.1
|Relative Error (%)|	0.1	0.1	5.5	5.3

Blinded Attending Surgeon Review						
Reviewer	PGT Estimate	H1 meets definition?	H2 meets definition?	AI meets definition?	Which is AI?	Which is most accurate?
1	10	Yes	Yes	No	AI	H2
2	0	Yes	Yes	No	H2	H2
3	10	Yes	Yes	Yes	H1	AI

© 2021 Howell RS et al. *JAMA Network Open.*

Wound EMR Information								
Sequential Number	Gender	Age	Wound Type	Wound Location	Wound Length (cm)	Wound Width (cm)	Wound Depth (cm)	Wound Area (cm^2)
60	M	64	DFU	L plantar	6.0	3.5	0.3	21.00

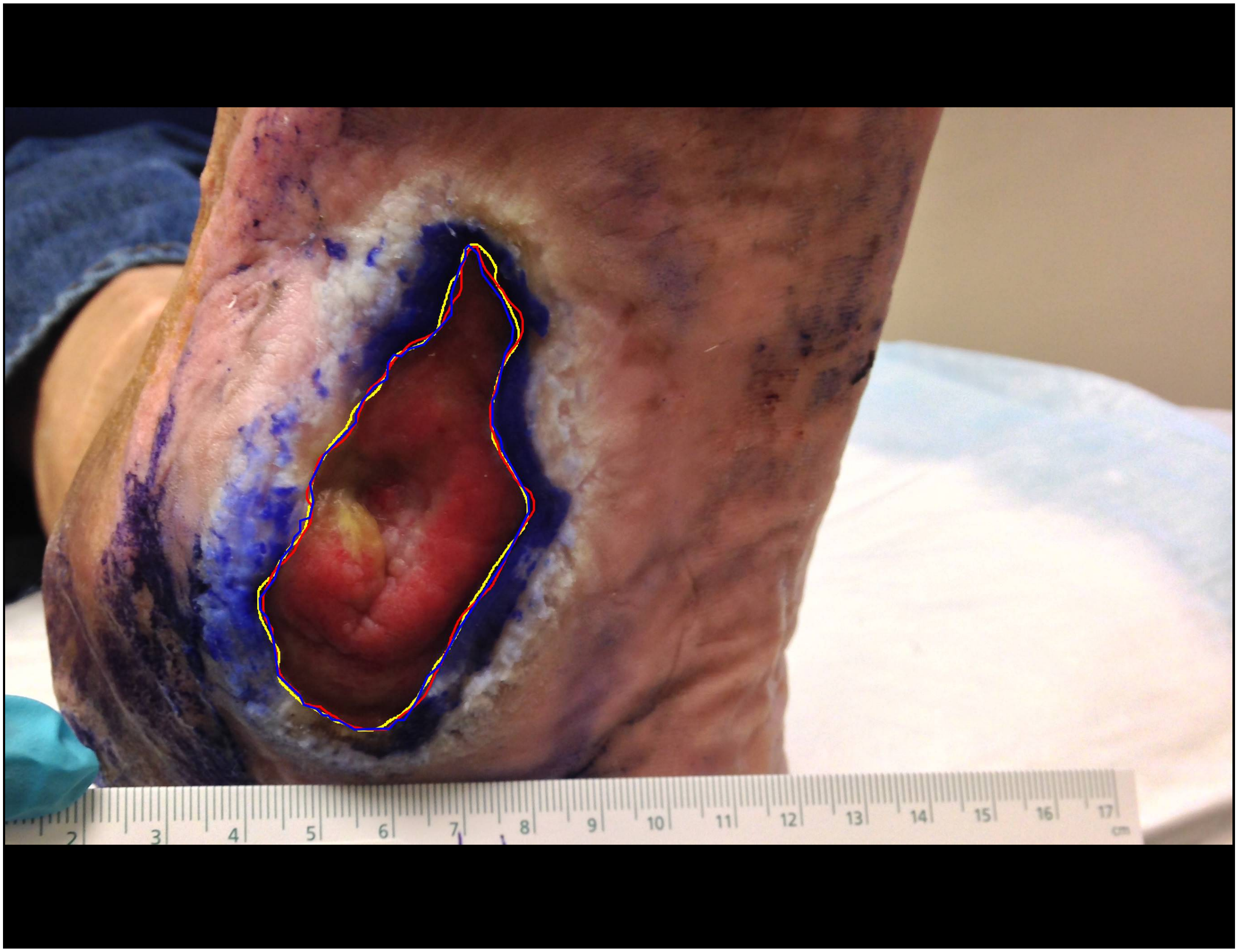

Tracing Data			
Tracer:	Wound Area (px^2)	Ruler Calibration (px/cm)	Wound Area (cm^2)
H1	346190	161.3	13.30
H2	343120	160.0	13.40
AI	342294	155.3	14.20

Tracing Comparisons				
Difference Metric:	Human-Human		Human-AI	
	H1(ref) H2(test)	H2(ref) H1(test)	H1(ref) AI(test)	H2(ref) AI(test)
False Negative Area (%)	3.1	2.3	2.9	2.4
False Positive Area (%)	2.2	3.2	1.8	2.2
|Relative Error (%)|	0.9	0.9	1.1	0.2

Blinded Attending Surgeon Review						
Reviewer	PGT Estimate	H1 meets definition?	H2 meets definition?	AI meets definition?	Which is AI?	Which is most accurate?
1	80	Yes	Yes	Yes	H1	H1
2	60	Yes	Yes	Yes	H2	H1
3	60	Yes	Yes	Yes	H1	H1

© 2021 Howell RS et al. *JAMA Network Open.*

Wound EMR Information								
Sequential Number	Gender	Age	Wound Type	Wound Location	Wound Length (cm)	Wound Width (cm)	Wound Depth (cm)	Wound Area (cm^2)
61	M	70	PU	R foot	1.5	1.8		2.70

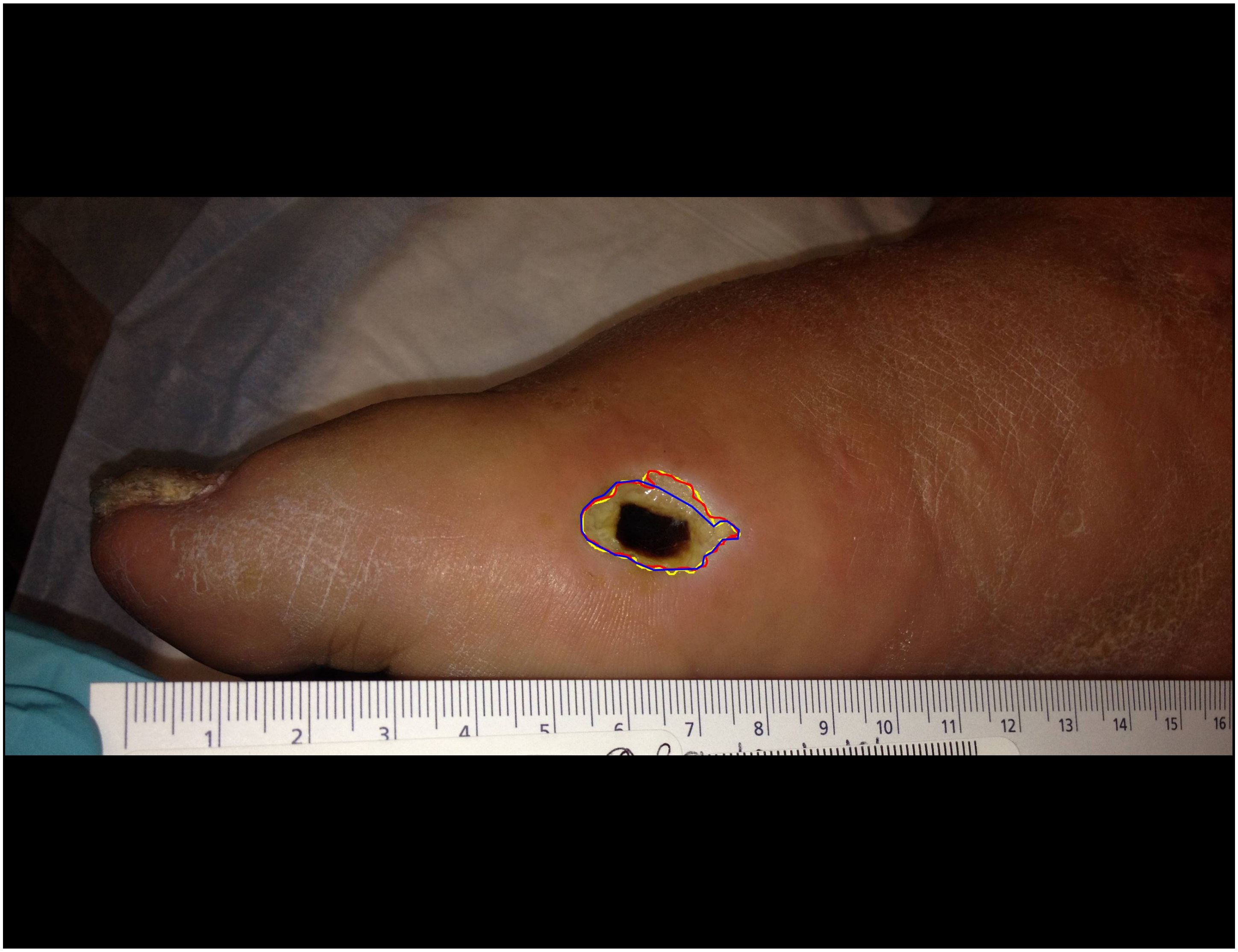

Tracing Data			
Tracer:	Wound Area (px^2)	Ruler Calibration (px/cm)	Wound Area (cm^2)
H1	69407	197.5	1.78
H2	67886	188.0	1.92
AI	62895	212.0	1.40

Tracing Comparisons				
Difference Metric:	Human-Human		Human-AI	
	H1(ref) H2(test)	H2(ref) H1(test)	H1(ref) AI(test)	H2(ref) AI(test)
False Negative Area (%)	5.1	3.0	12.7	11.4
False Positive Area (%)	2.9	5.2	3.4	4.1
|Relative Error (%)|	2.2	2.2	9.4	7.4

Blinded Attending Surgeon Review						
Reviewer	PGT Estimate	H1 meets definition?	H2 meets definition?	AI meets definition?	Which is AI?	Which is most accurate?
1	0	Yes	Yes	No	AI	AI
2	0	Yes	Yes	No	AI	H2
3	50	Yes	Yes	Yes	H1	AI

© 2021 Howell RS et al. *JAMA Network Open.*

Wound EMR Information								
Sequential Number	Gender	Age	Wound Type	Wound Location	Wound Length (cm)	Wound Width (cm)	Wound Depth (cm)	Wound Area (cm^2)
62	F	60	abscess	R med thigh	2.0	1.0	0.1	2.00

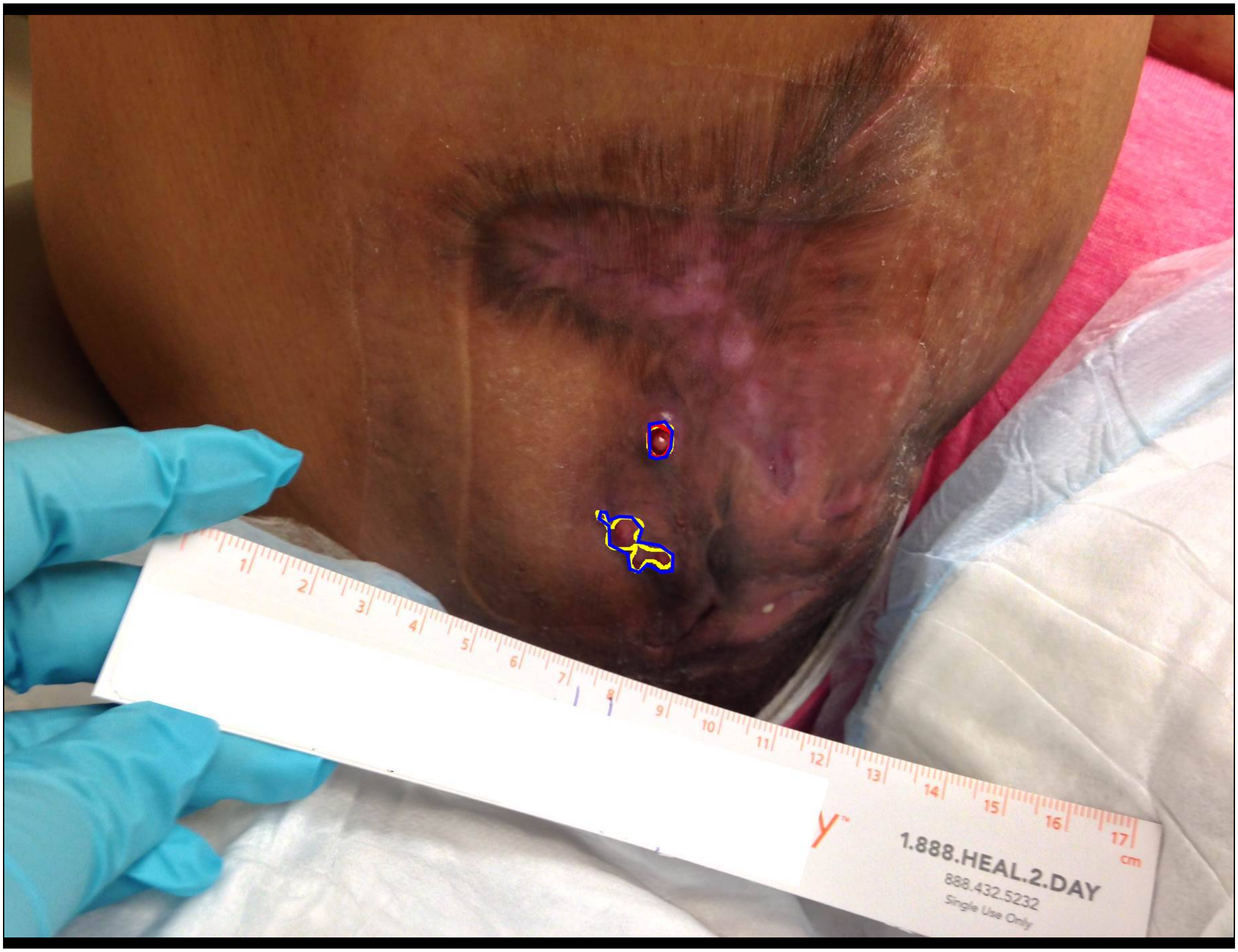

Tracing Data			
Tracer:	Wound Area (px^2)	Ruler Calibration (px/cm)	Wound Area (cm^2)
H1	4943	76.3	0.85
H2	1324	42.9	0.72
AI	6006	92.6	0.70

Tracing Comparisons				
Difference Metric:	Human-Human		Human-AI	
	H1(ref) H2(test)	H2(ref) H1(test)	H1(ref) AI(test)	H2(ref) AI(test)
False Negative Area (%)	74.4	4.4	6.2	3.6
False Positive Area (%)	1.2	277.7	27.7	357.3
|Relative Error (%)|	73.2	273.3	21.5	353.6

Blinded Attending Surgeon Review						
Reviewer	PGT Estimate	H1 meets definition?	H2 meets definition?	AI meets definition?	Which is AI?	Which is most accurate?
1	100	No	No	Yes	H1	AI
2	0	Yes	Yes	Yes	AI	H2
3	30	Yes	Yes	No	AI	H1

© 2021 Howell RS et al. *JAMA Network Open.*

Wound EMR Information								
Sequential Number	Gender	Age	Wound Type	Wound Location	Wound Length (cm)	Wound Width (cm)	Wound Depth (cm)	Wound Area (cm^2)
63	F	37	VLU	RLE med	7.8	2.8		21.84

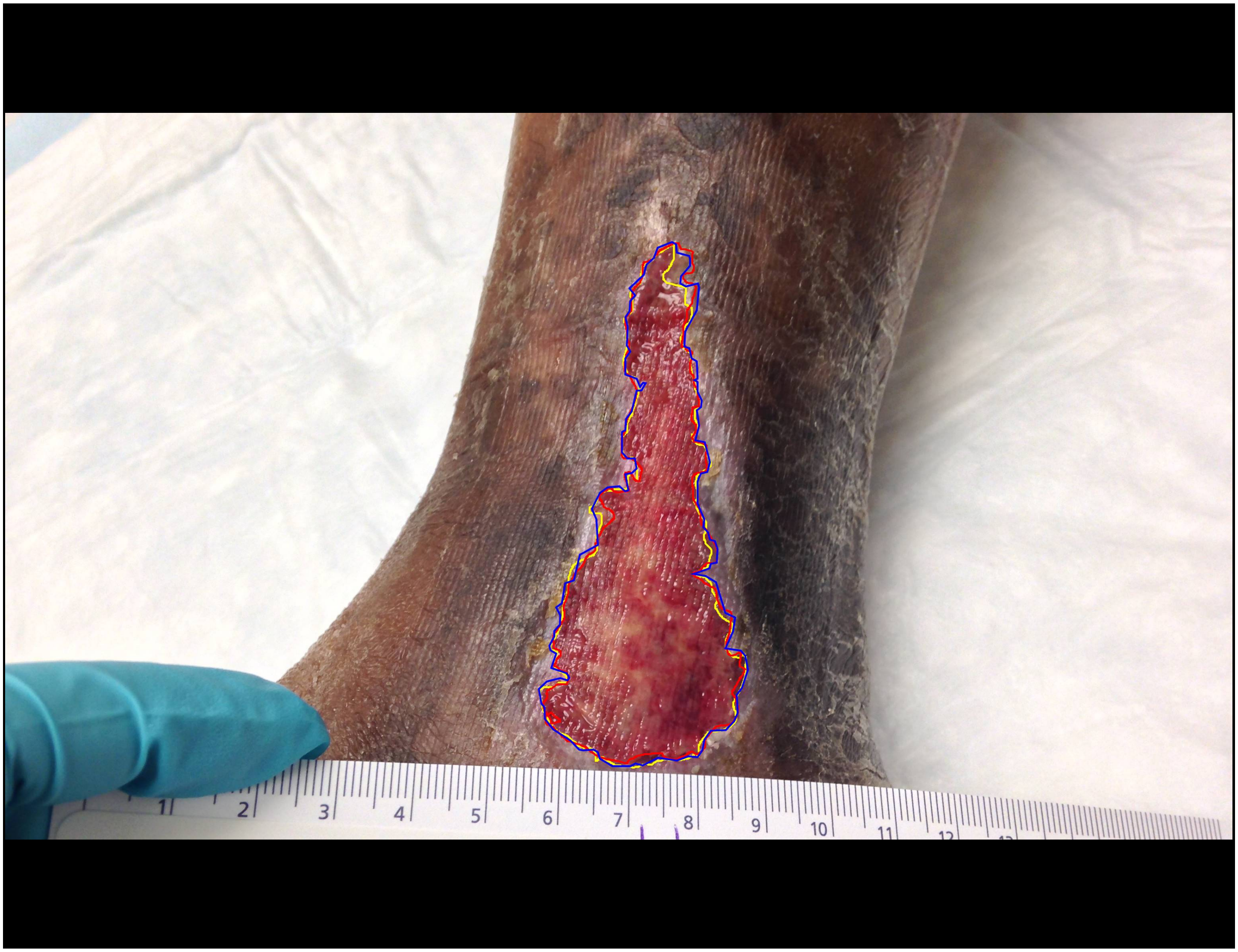

Tracing Data			
Tracer:	Wound Area (px^2)	Ruler Calibration (px/cm)	Wound Area (cm^2)
H1	382176	194.8	10.07
H2	380272	188.2	10.74
AI	401955	200.5	10.00

Tracing Comparisons				
Difference Metric:	Human-Human		Human-AI	
	H1(ref) H2(test)	H2(ref) H1(test)	H1(ref) AI(test)	H2(ref) AI(test)
False Negative Area (%)	4.3	3.8	1.0	1.1
False Positive Area (%)	3.8	4.3	6.2	6.8
|Relative Error (%)|	0.5	0.5	5.2	5.7

Blinded Attending Surgeon Review						
Reviewer	PGT Estimate	H1 meets definition?	H2 meets definition?	AI meets definition?	Which is AI?	Which is most accurate?
1	90	Yes	Yes	Yes	H1	H1
2	60	Yes	Yes	Yes	AI	H1
3	100	Yes	Yes	Yes	H1	AI

© 2021 Howell RS et al. *JAMA Network Open.*

Wound EMR Information								
Sequential Number	Gender	Age	Wound Type	Wound Location	Wound Length (cm)	Wound Width (cm)	Wound Depth (cm)	Wound Area (cm^2)
64	F	84	Arterial	LLE lat	1.2	0.7	0.1	0.84

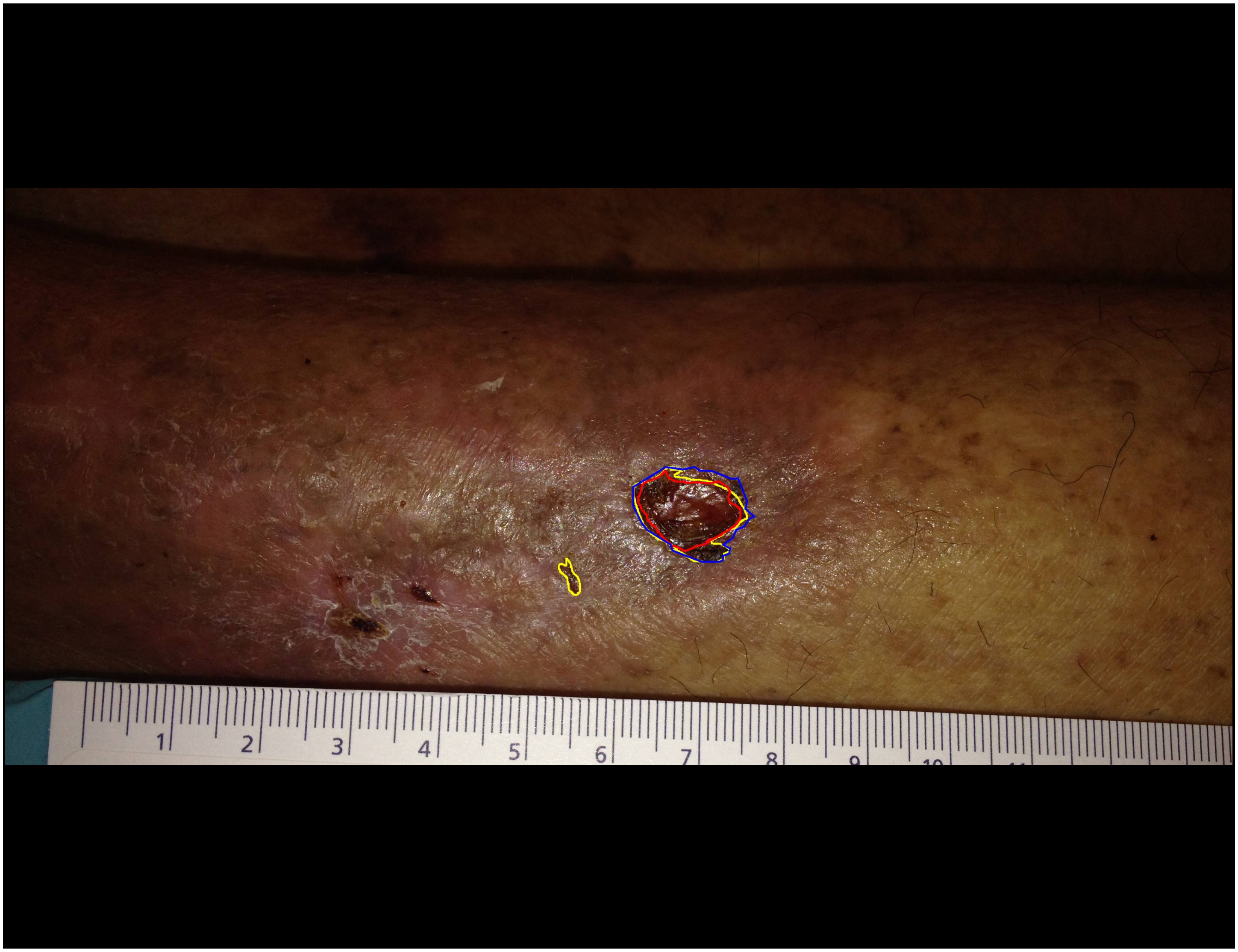

Tracing Data			
Tracer:	Wound Area (px^2)	Ruler Calibration (px/cm)	Wound Area (cm^2)
H1	49869	229.1	0.95
H2	37106	228.1	0.71
AI	56475	237.6	1.00

Tracing Comparisons				
Difference Metric:	Human-Human		Human-AI	
	H1(ref) H2(test)	H2(ref) H1(test)	H1(ref) AI(test)	H2(ref) AI(test)
False Negative Area (%)	26.2	0.8	5.5	0.0
False Positive Area (%)	0.6	35.2	18.7	52.1
|Relative Error (%)|	25.6	34.4	13.2	52.2

Blinded Attending Surgeon Review						
Reviewer	PGT Estimate	H1 meets definition?	H2 meets definition?	AI meets definition?	Which is AI?	Which is most accurate?
1	0	No	No	No	H2	AI
2	0	No	No	Yes	H2	H1
3	100	Yes	Yes	Yes	H1	H2

© 2021 Howell RS et al. *JAMA Network Open.*

Wound EMR Information								
Sequential Number	Gender	Age	Wound Type	Wound Location	Wound Length (cm)	Wound Width (cm)	Wound Depth (cm)	Wound Area (cm^2)
65	F	73	DFU	R foot	1.5	1.1	0.2	1.65

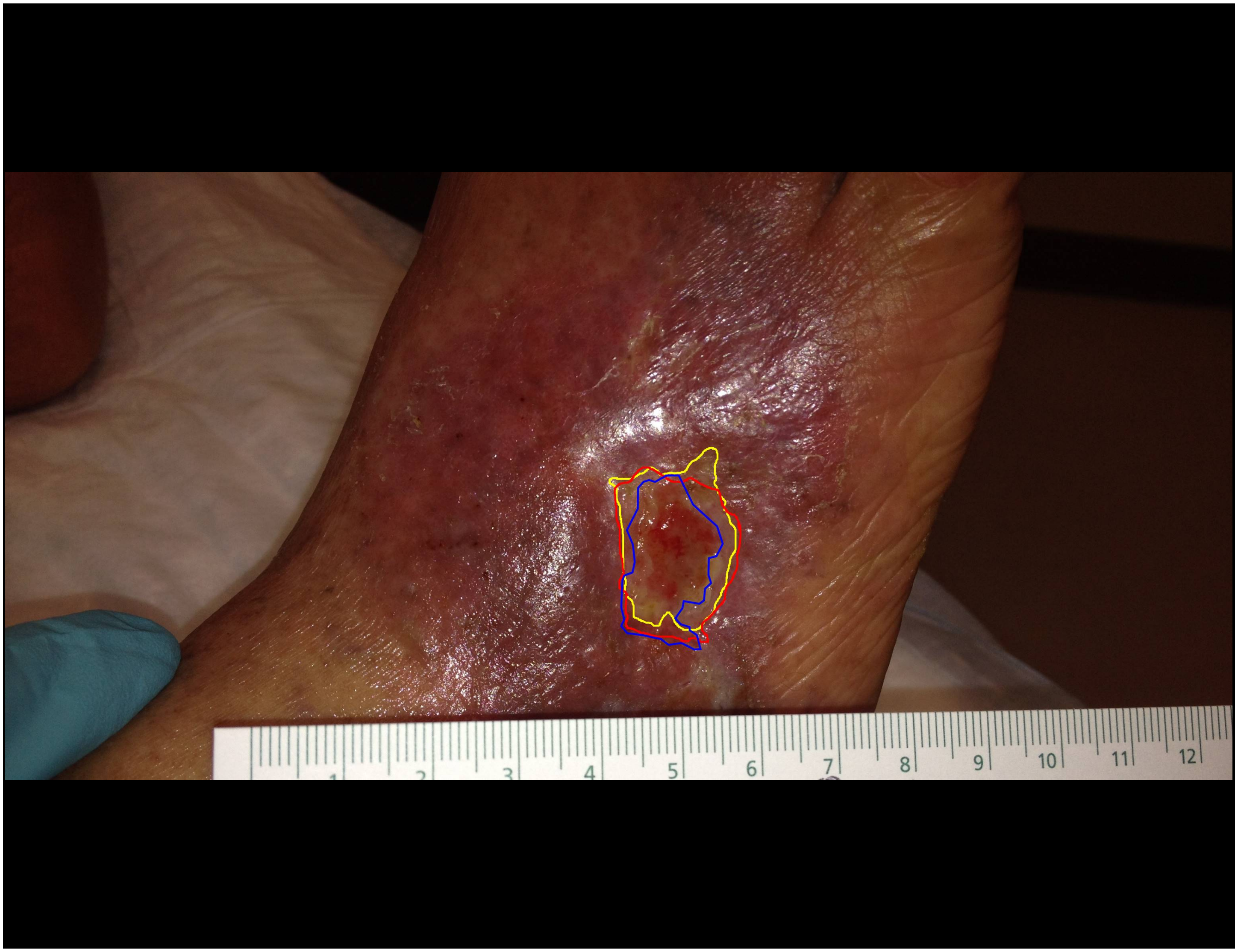

Tracing Data			
Tracer:	Wound Area (px^2)	Ruler Calibration (px/cm)	Wound Area (cm^2)
H1	110360	214.9	2.39
H2	116689	214.7	2.53
AI	81399	241.1	1.40

Tracing Comparisons				
Difference Metric:	Human-Human		Human-AI	
	H1(ref) H2(test)	H2(ref) H1(test)	H1(ref) AI(test)	H2(ref) AI(test)
False Negative Area (%)	7.4	12.4	36.4	33.9
False Positive Area (%)	13.2	7.0	10.2	3.6
|Relative Error (%)|	5.7	5.4	26.2	30.2

Blinded Attending Surgeon Review						
Reviewer	PGT Estimate	H1 meets definition?	H2 meets definition?	AI meets definition?	Which is AI?	Which is most accurate?
1	20	No	Yes	No	H1	AI
2	<10	Yes	No	No	H2	H1
3	80	No	Yes	No	H2	H1

© 2021 Howell RS et al. *JAMA Network Open.*

Wound EMR Information								
Sequential Number	Gender	Age	Wound Type	Wound Location	Wound Length (cm)	Wound Width (cm)	Wound Depth (cm)	Wound Area (cm^2)
66	M	82	Surgical	L BKA	6.8	7.0	0.3	47.60

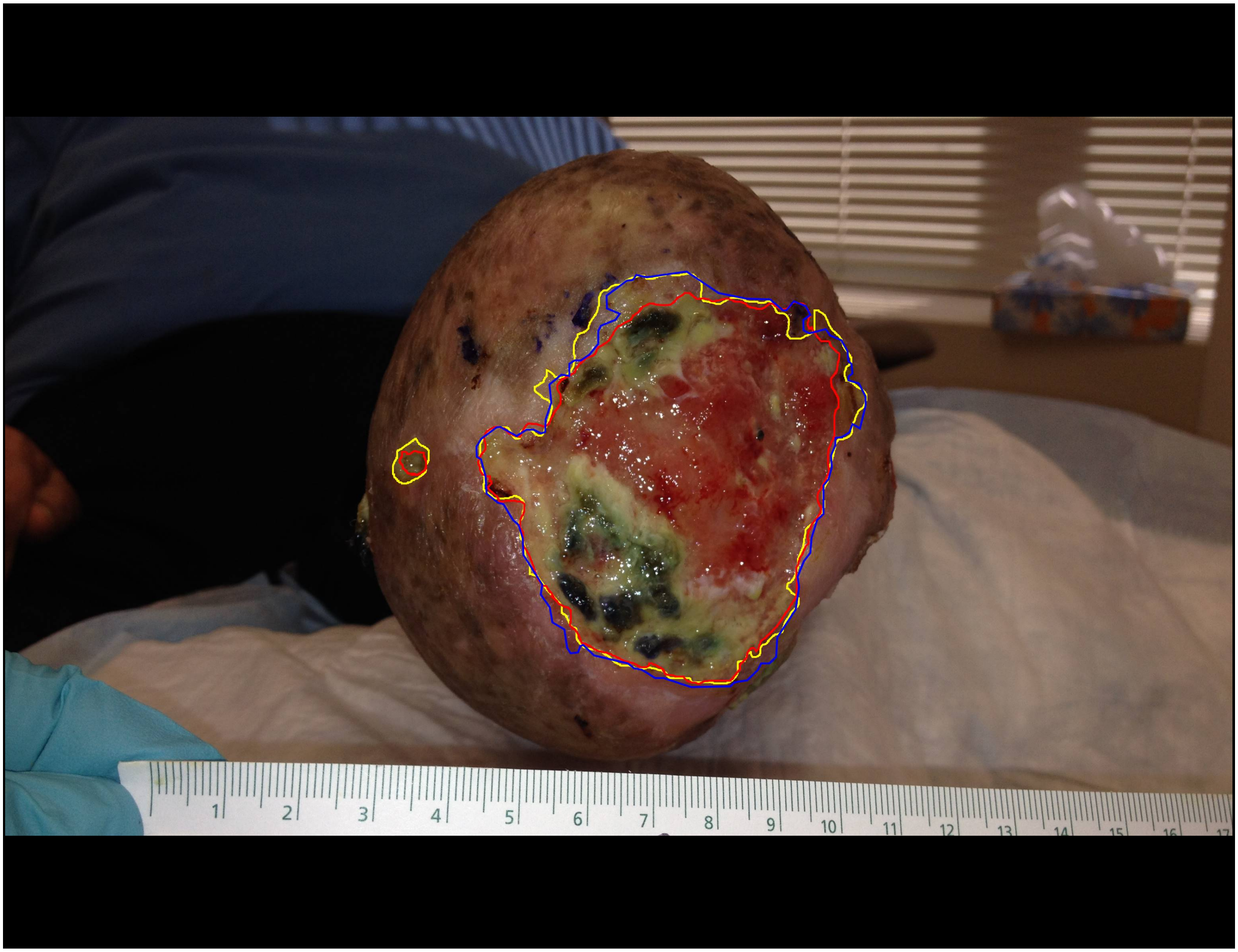

Tracing Data			
Tracer:	Wound Area (px^2)	Ruler Calibration (px/cm)	Wound Area (cm^2)
H1	733307	181.5	22.26
H2	677335	172.1	22.87
AI	745165	199.1	18.80

Tracing Comparisons				
Difference Metric:	Human-Human		Human-AI	
	H1(ref) H2(test)	H2(ref) H1(test)	H1(ref) AI(test)	H2(ref) AI(test)
False Negative Area (%)	9.0	1.5	3.1	0.8
False Positive Area (%)	1.4	9.8	4.7	10.9
|Relative Error (%)|	7.6	8.3	1.6	10.0

Blinded Attending Surgeon Review						
Reviewer	PGT Estimate	H1 meets definition?	H2 meets definition?	AI meets definition?	Which is AI?	Which is most accurate?
1	30	Yes	Yes	No	H1	AI
2	10	Yes	Yes	No	H2	H1
3	70	No	Yes	Yes	AI	H1

© 2021 Howell RS et al. *JAMA Network Open.*

Wound EMR Information								
Sequential Number	Gender	Age	Wound Type	Wound Location	Wound Length (cm)	Wound Width (cm)	Wound Depth (cm)	Wound Area (cm^2)
67	F	80	PU	L buttock	2.1	2.0	0.1	4.20

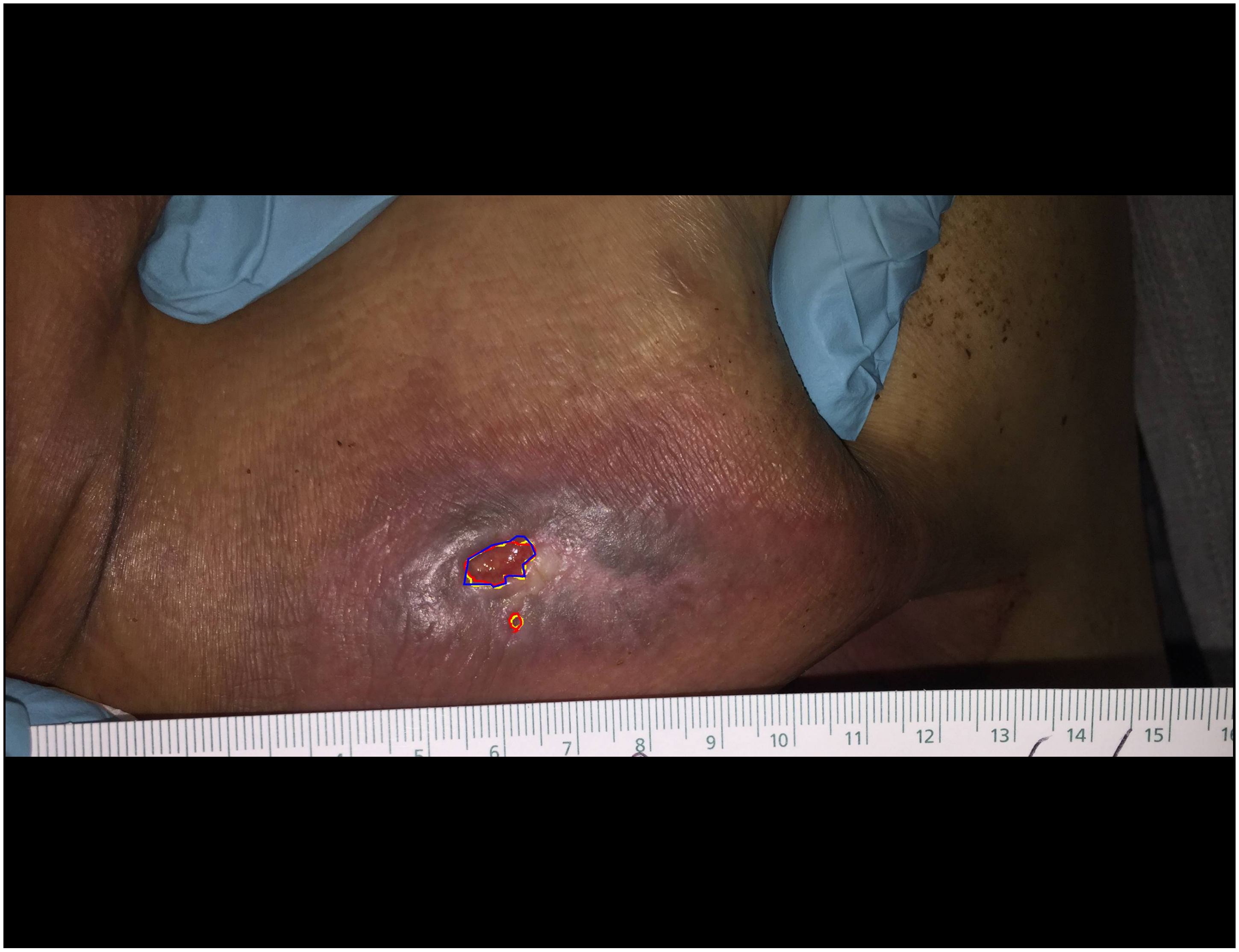

Tracing Data			
Tracer:	Wound Area (px^2)	Ruler Calibration (px/cm)	Wound Area (cm^2)
H1	15694	195.6	0.41
H2	14922	201.6	0.37
AI	15751	229.1	0.30

Tracing Comparisons				
Difference Metric:	Human-Human		Human-AI	
	H1(ref) H2(test)	H2(ref) H1(test)	H1(ref) AI(test)	H2(ref) AI(test)
False Negative Area (%)	9.8	5.1	8.5	9.4
False Positive Area (%)	4.9	10.3	8.8	15.0
|Relative Error (%)|	4.9	5.2	0.4	5.6

Blinded Attending Surgeon Review						
Reviewer	PGT Estimate	H1 meets definition?	H2 meets definition?	AI meets definition?	Which is AI?	Which is most accurate?
1	100	Yes	Yes	No	H2	H2
2	50	Yes	Yes	No	H1	AI
3	40	No	Yes	Yes	H1	H2

© 2021 Howell RS et al. *JAMA Network Open.*

Wound EMR Information								
Sequential Number	Gender	Age	Wound Type	Wound Location	Wound Length (cm)	Wound Width (cm)	Wound Depth (cm)	Wound Area (cm^2)
68	F	78	PU	sacrum	0.0	0.0	0.0	0.00

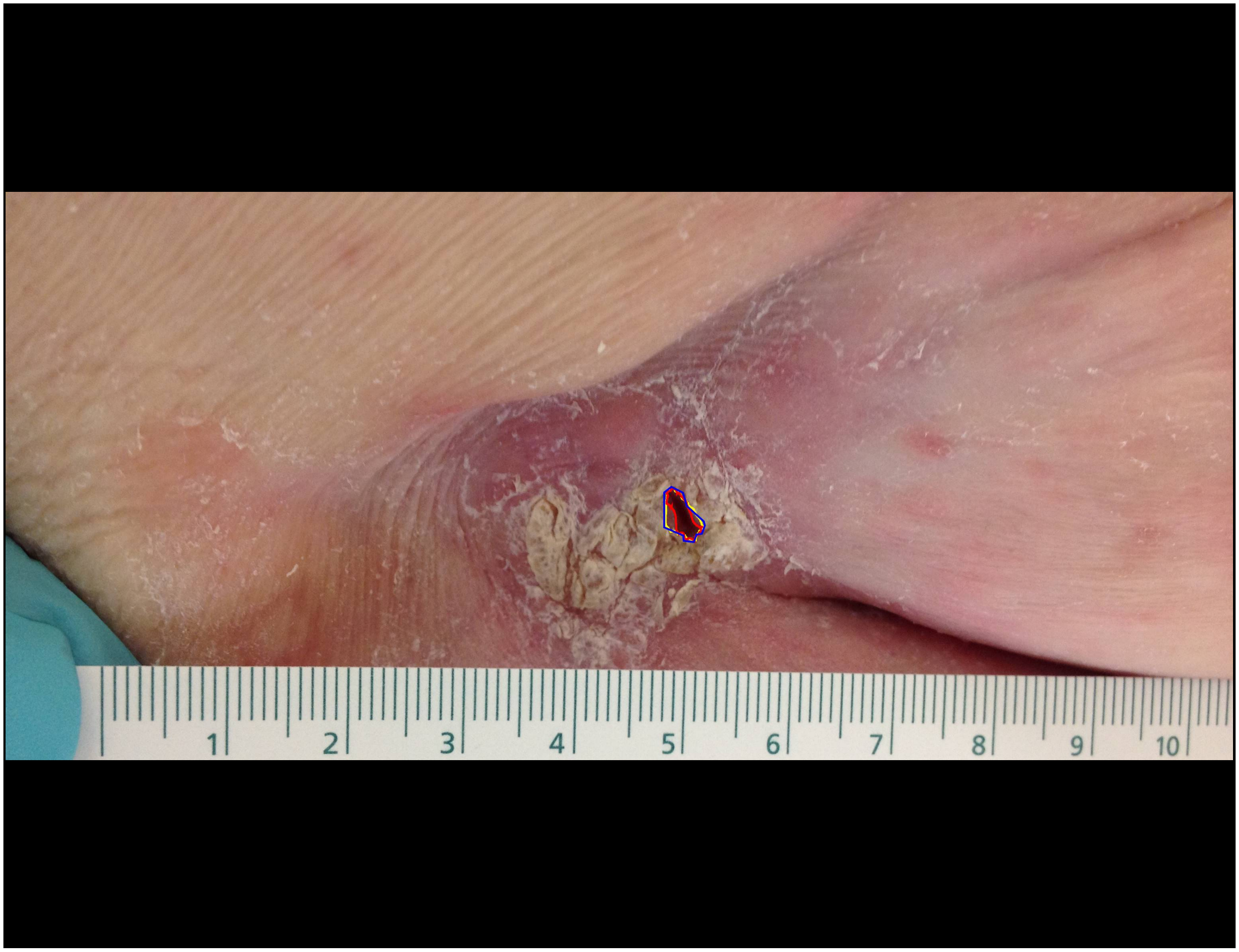

Tracing Data			
Tracer:	Wound Area (px^2)	Ruler Calibration (px/cm)	Wound Area (cm^2)
H1	8421	290.2	0.10
H2	5809	290.2	0.07
AI	9592	309.7	0.10

Tracing Comparisons				
Difference Metric:	Human-Human		Human-AI	
	H1(ref) H2(test)	H2(ref) H1(test)	H1(ref) AI(test)	H2(ref) AI(test)
False Negative Area (%)	32.8	2.5	3.0	0.5
False Positive Area (%)	1.8	47.5	16.9	65.6
|Relative Error (%)|	31.0	45.0	13.9	65.1

Blinded Attending Surgeon Review						
Reviewer	PGT Estimate	H1 meets definition?	H2 meets definition?	AI meets definition?	Which is AI?	Which is most accurate?
1	0	No	Yes	No	AI	AI
2	100	No	Yes	Yes	H2	AI
3	10	No	Yes	No	H1	AI

© 2021 Howell RS et al. *JAMA Network Open.*

Wound EMR Information								
Sequential Number	Gender	Age	Wound Type	Wound Location	Wound Length (cm)	Wound Width (cm)	Wound Depth (cm)	Wound Area (cm^2)
69	M	54	Surgical	achilles	1.9	1.6	0.2	3.04

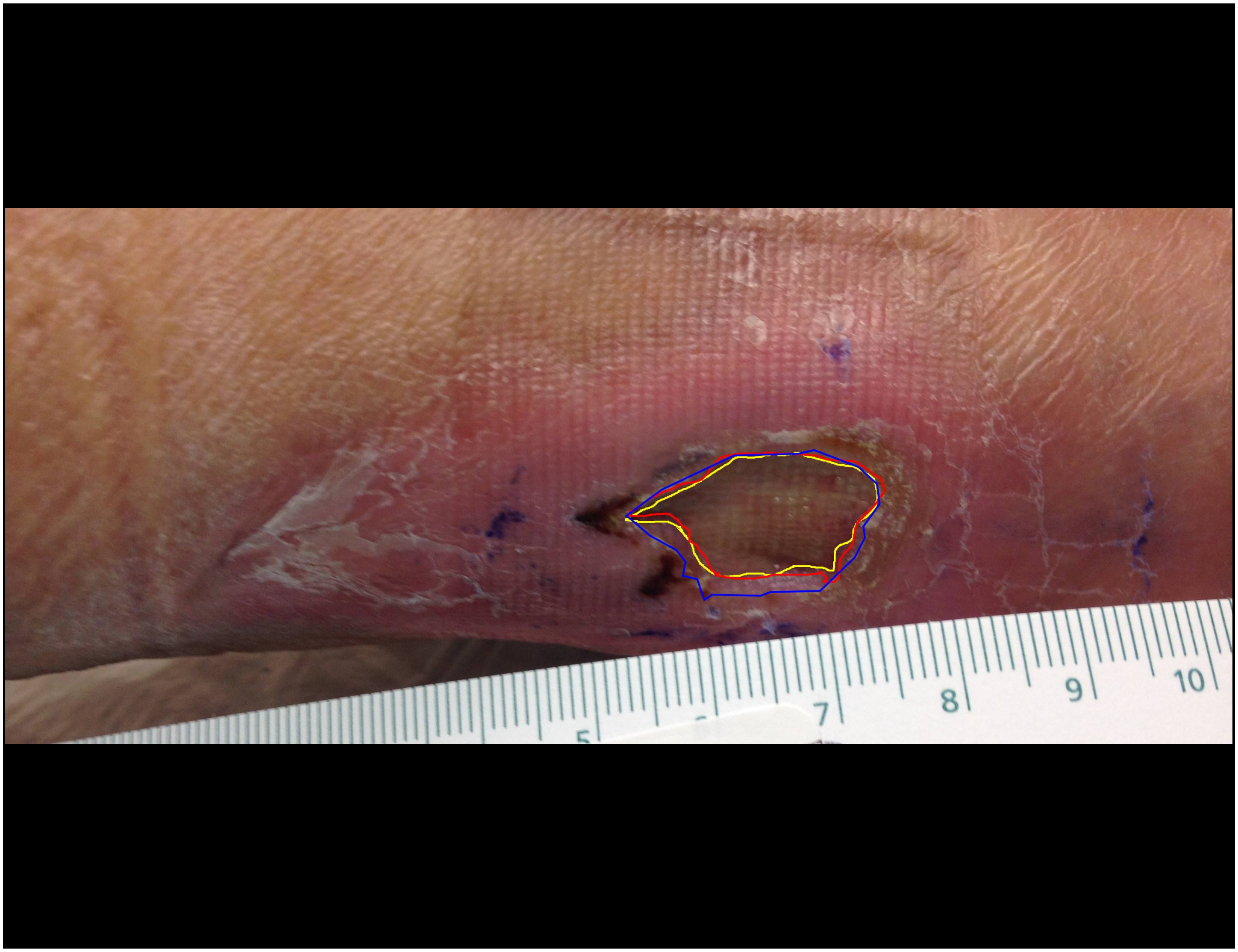

Tracing Data			
Tracer:	Wound Area (px^2)	Ruler Calibration (px/cm)	Wound Area (cm^2)
H1	137974	329.6	1.27
H2	147784	324.8	1.40
AI	182165	294.5	2.10

Tracing Comparisons				
Difference Metric:	Human-Human		Human-AI	
	H1(ref) H2(test)	H2(ref) H1(test)	H1(ref) AI(test)	H2(ref) AI(test)
False Negative Area (%)	3.4	9.8	0.2	2.1
False Positive Area (%)	10.5	3.1	32.2	25.4
|Relative Error (%)|	7.1	6.6	32.0	23.3

Blinded Attending Surgeon Review						
Reviewer	PGT Estimate	H1 meets definition?	H2 meets definition?	AI meets definition?	Which is AI?	Which is most accurate?
1	0	Yes	No	No	H1	H2
2	0	No	No	No	H1	H1
3	0	Yes	Yes	No	AI	H2

© 2021 Howell RS et al. *JAMA Network Open.*

Wound EMR Information								
Sequential Number	Gender	Age	Wound Type	Wound Location	Wound Length (cm)	Wound Width (cm)	Wound Depth (cm)	Wound Area (cm^2)
70	F	58	bursitis	L elbow	0.8	0.4	0.1	0.32

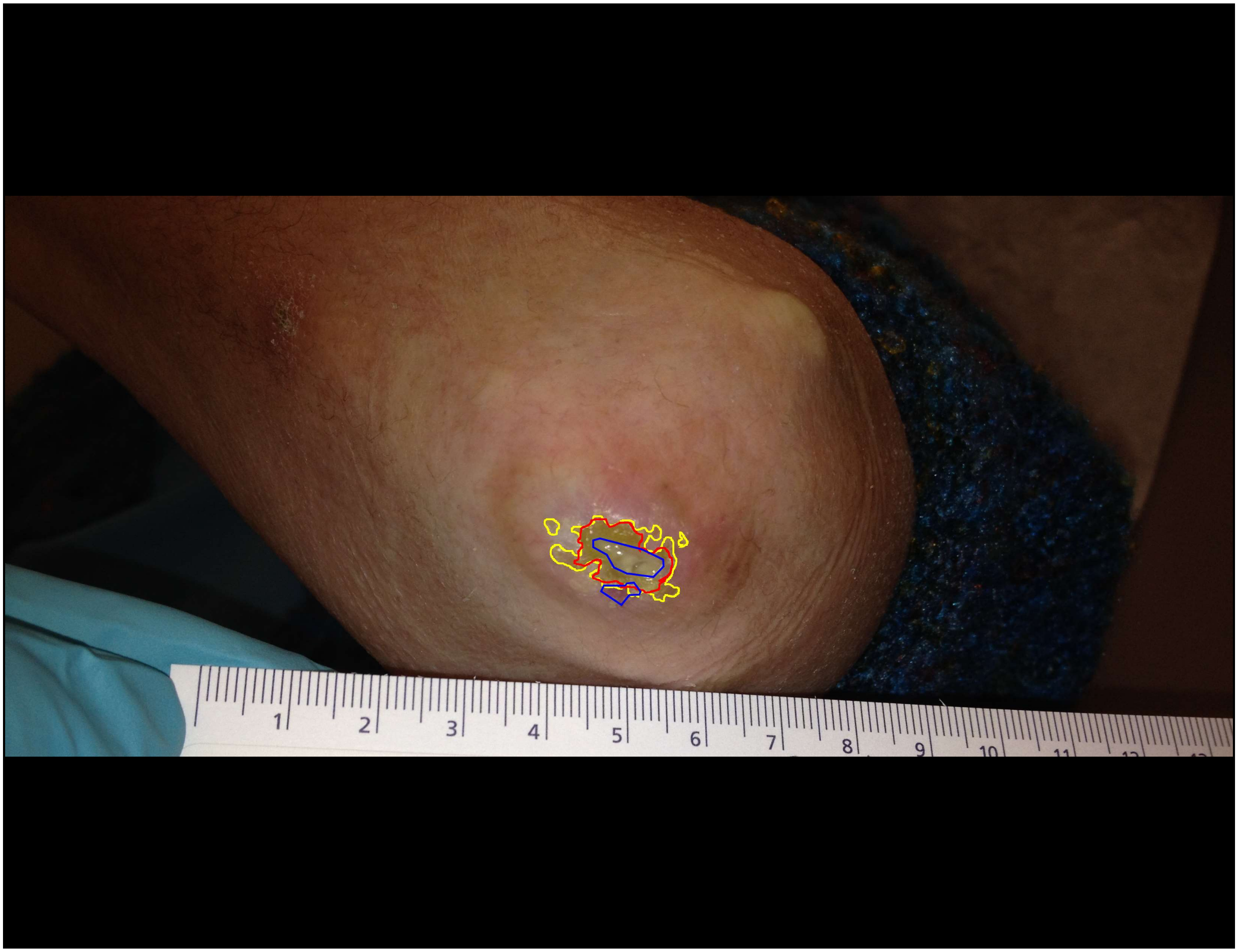

Tracing Data			
Tracer:	Wound Area (px^2)	Ruler Calibration (px/cm)	Wound Area (cm^2)
H1	43395	219.6	0.90
H2	30797	213.3	0.68
AI	13738	214.0	0.30

Tracing Comparisons				
Difference Metric:	Human-Human		Human-AI	
	H1(ref) H2(test)	H2(ref) H1(test)	H1(ref) AI(test)	H2(ref) AI(test)
False Negative Area (%)	30.3	1.7	73.6	65.2
False Positive Area (%)	1.2	42.6	5.2	9.8
|Relative Error (%)|	29.0	40.9	68.3	55.4

Blinded Attending Surgeon Review						
Reviewer	PGT Estimate	H1 meets definition?	H2 meets definition?	AI meets definition?	Which is AI?	Which is most accurate?
1	0	Yes	Yes	No	AI	H1
2	0	Yes	Yes	No	H2	AI
3	0	No	No	Yes	H2	H2

© 2021 Howell RS et al. *JAMA Network Open.*

Wound EMR Information								
Sequential Number	Gender	Age	Wound Type	Wound Location	Wound Length (cm)	Wound Width (cm)	Wound Depth (cm)	Wound Area (cm^2)
71	M	50	PU	LLE post	7.7	3.0	0.3	23.10

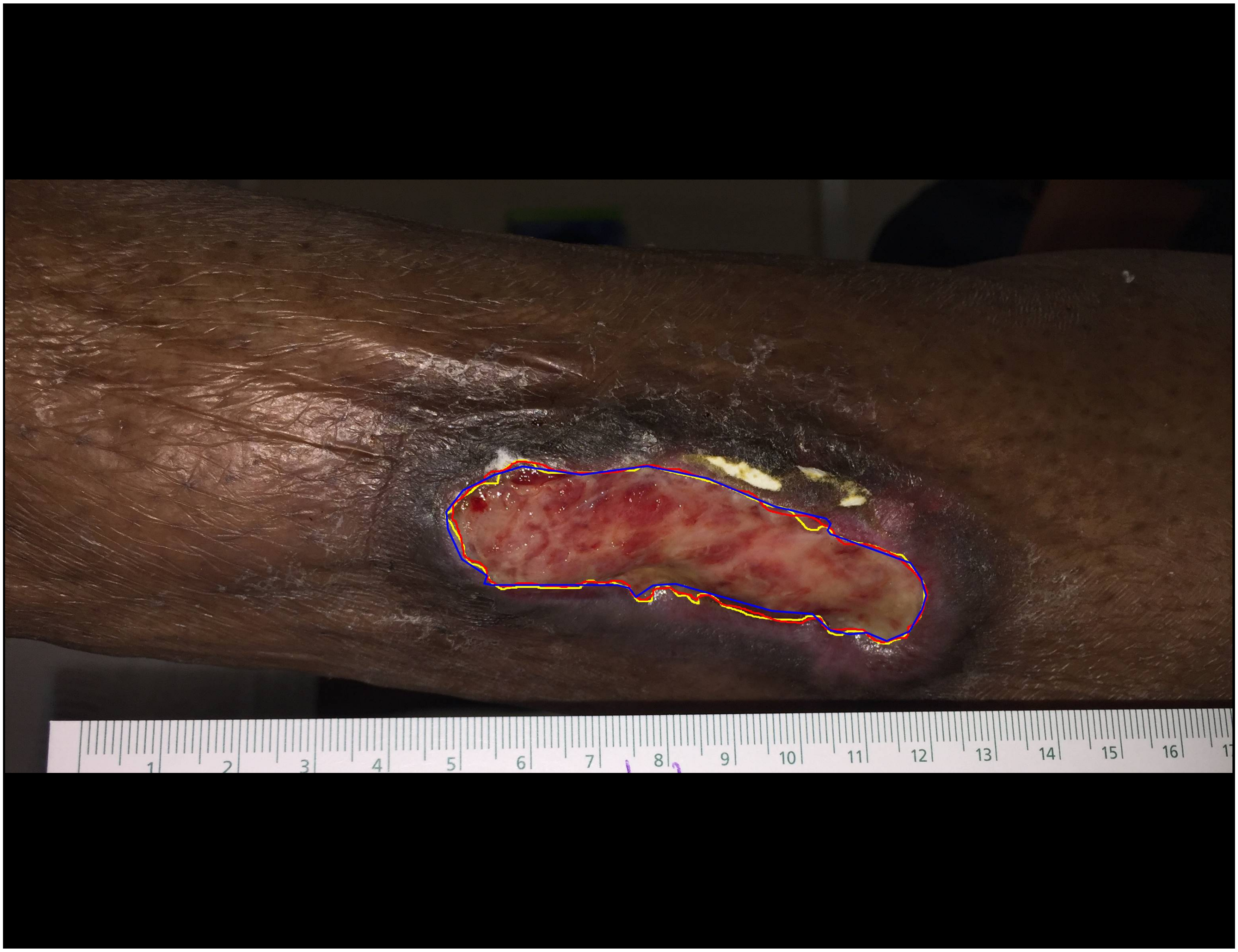

Tracing Data			
Tracer:	Wound Area (px^2)	Ruler Calibration (px/cm)	Wound Area (cm^2)
H1	349865	178.7	10.95
H2	348524	180.0	10.76
AI	345383	188.7	9.70

Tracing Comparisons				
Difference Metric:	Human-Human		Human-AI	
	H1(ref) H2(test)	H2(ref) H1(test)	H1(ref) AI(test)	H2(ref) AI(test)
False Negative Area (%)	2.7	2.3	4.2	4.0
False Positive Area (%)	2.3	2.7	2.9	3.1
|Relative Error (%)|	0.4	0.4	1.3	0.9

Blinded Attending Surgeon Review						
Reviewer	PGT Estimate	H1 meets definition?	H2 meets definition?	AI meets definition?	Which is AI?	Which is most accurate?
1	30	Yes	Yes	Yes	H1	H1
2	10	Yes	Yes	Yes	H1	AI
3	30	Yes	Yes	Yes	H2	H1

© 2021 Howell RS et al. *JAMA Network Open.*

Wound EMR Information								
Sequential Number	Gender	Age	Wound Type	Wound Location	Wound Length (cm)	Wound Width (cm)	Wound Depth (cm)	Wound Area (cm^2)
72	M	83	VLU	RLE lat	2.8	2.0	0.2	5.60

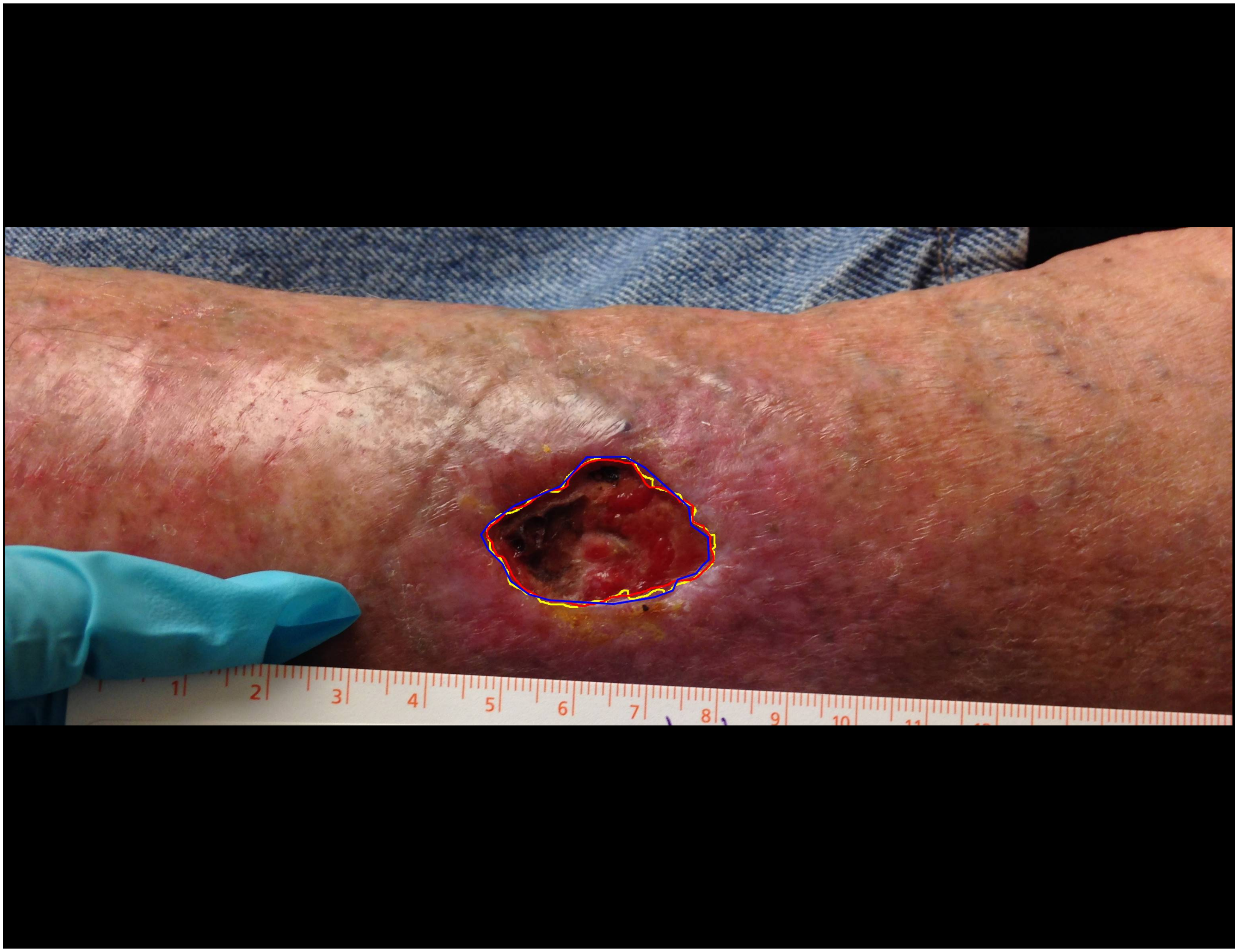

Tracing Data			
Tracer:	Wound Area (px^2)	Ruler Calibration (px/cm)	Wound Area (cm^2)
H1	161211	196.6	4.17
H2	154565	192.2	4.18
AI	162614	194.5	4.30

Tracing Comparisons				
Difference Metric:	Human-Human		Human-AI	
	H1(ref) H2(test)	H2(ref) H1(test)	H1(ref) AI(test)	H2(ref) AI(test)
False Negative Area (%)	4.9	0.8	3.3	2.1
False Positive Area (%)	0.8	5.1	4.2	7.3
|Relative Error (%)|	4.1	4.3	0.9	5.2

Blinded Attending Surgeon Review						
Reviewer	PGT Estimate	H1 meets definition?	H2 meets definition?	AI meets definition?	Which is AI?	Which is most accurate?
1	60	Yes	Yes	Yes	H1	H1
2	50	Yes	Yes	Yes	AI	H2
3	60	Yes	Yes	Yes	AI	AI

© 2021 Howell RS et al. *JAMA Network Open.*

Wound EMR Information								
Sequential Number	Gender	Age	Wound Type	Wound Location	Wound Length (cm)	Wound Width (cm)	Wound Depth (cm)	Wound Area (cm^2)
73	F	79	Surgical	coccyx	2.0	1.8	1.8	3.60

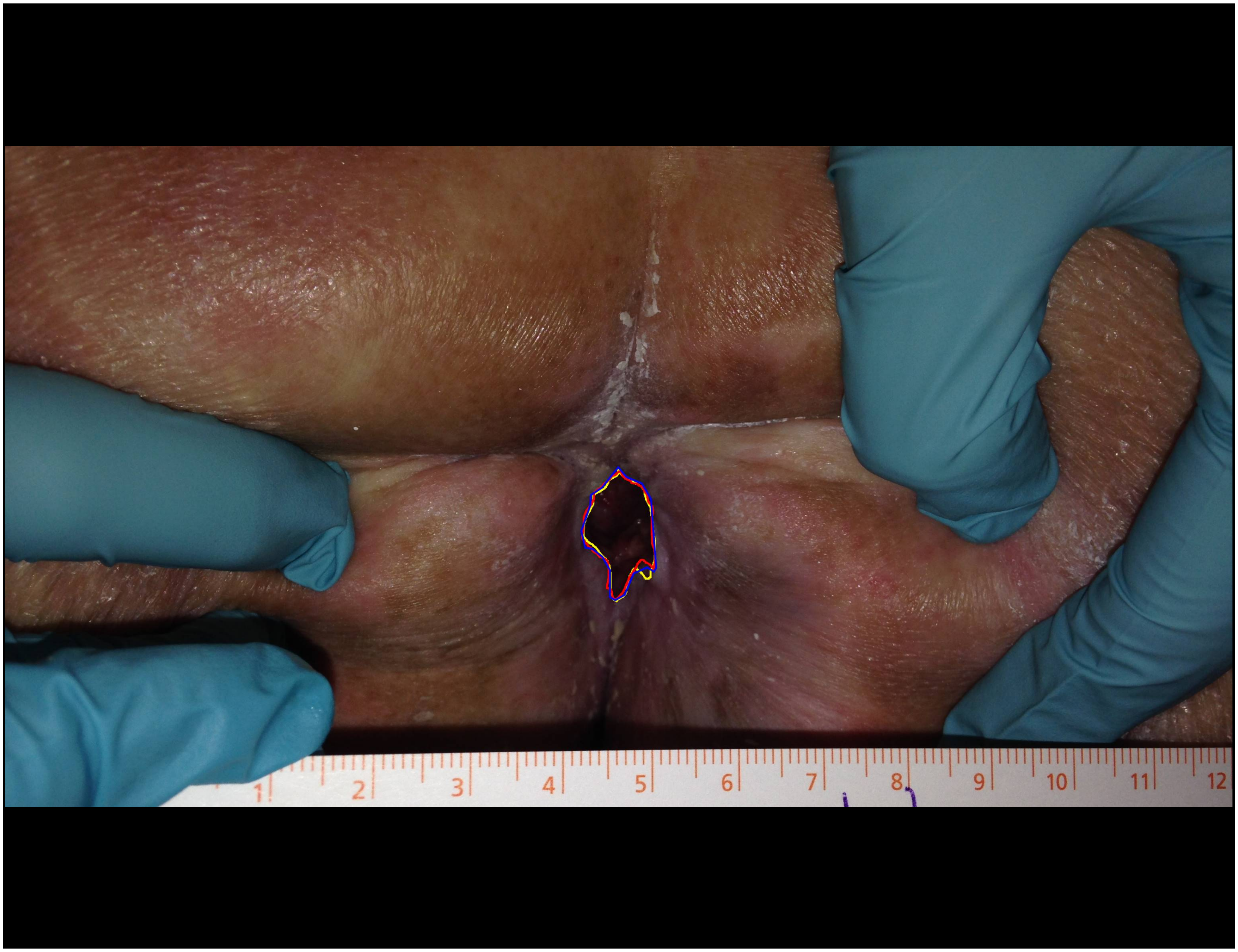

Tracing Data			
Tracer:	Wound Area (px^2)	Ruler Calibration (px/cm)	Wound Area (cm^2)
H1	39668	236.4	0.71
H2	39806	238.1	0.70
AI	42446	266.0	0.60

Tracing Comparisons				
Difference Metric:	Human-Human		Human-AI	
	H1(ref) H2(test)	H2(ref) H1(test)	H1(ref) AI(test)	H2(ref) AI(test)
False Negative Area (%)	5.4	5.7	1.7	1.9
False Positive Area (%)	5.8	5.4	8.7	8.5
|Relative Error (%)|	0.3	0.3	7.0	6.6

Blinded Attending Surgeon Review						
Reviewer	PGT Estimate	H1 meets definition?	H2 meets definition?	AI meets definition?	Which is AI?	Which is most accurate?
1		Yes	Yes	Yes	AI	AI
2		No	No	Yes	H1	H2
3	60	Yes	Yes	Yes	H2	AI

© 2021 Howell RS et al. *JAMA Network Open.*

Wound EMR Information								
Sequential Number	Gender	Age	Wound Type	Wound Location	Wound Length (cm)	Wound Width (cm)	Wound Depth (cm)	Wound Area (cm^2)
74	M	67	Surgical	R 3rd toe	0.5	0.6	0.5	0.30

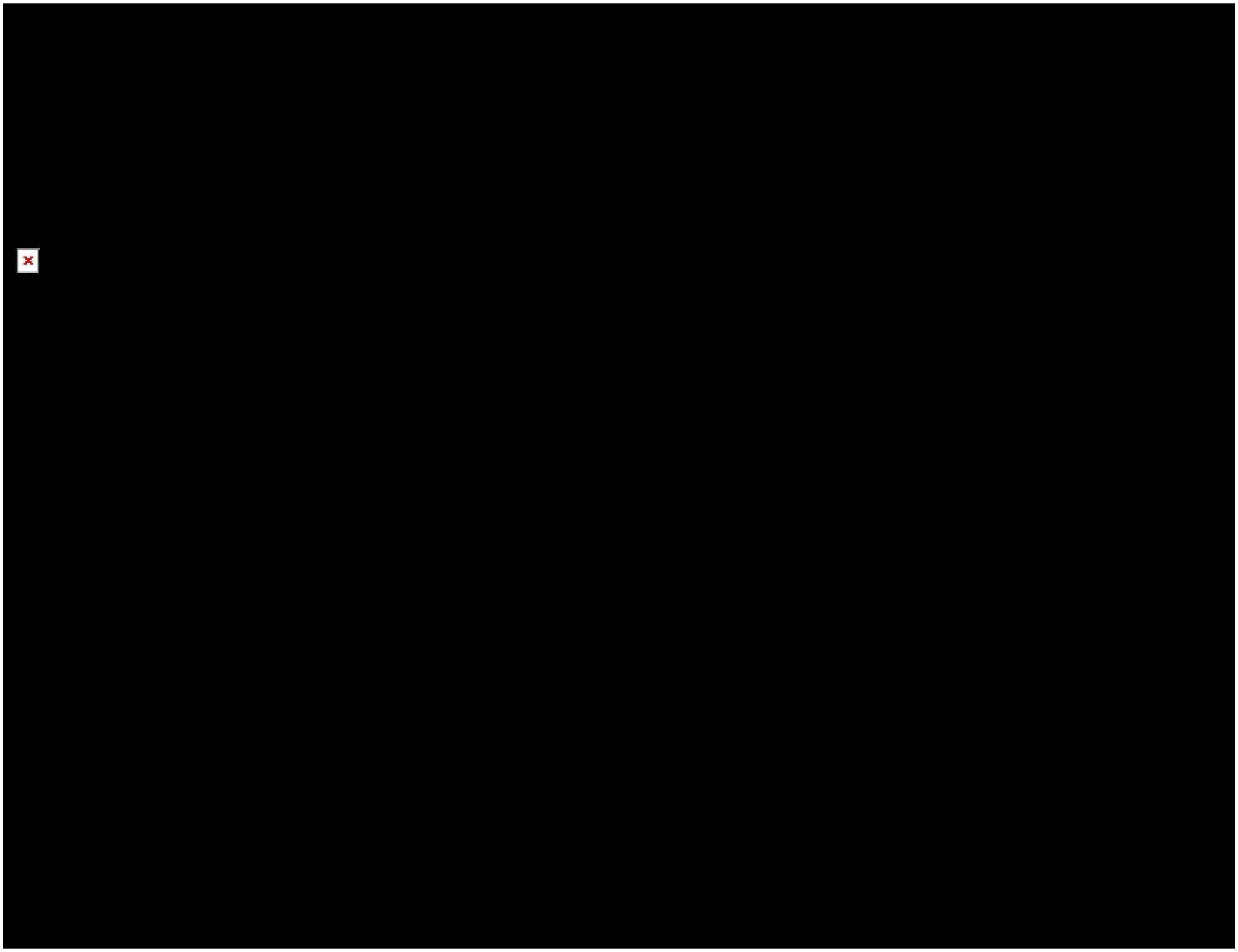

Tracing Data			
Tracer:	Wound Area (px^2)	Ruler Calibration (px/cm)	Wound Area (cm^2)
H1	12691	169.8	0.44
H2	14515	172.6	0.49
AI	0		0.00

Tracing Comparisons				
Difference Metric:	Human-Human		Human-AI	
	H1(ref) H2(test)	H2(ref) H1(test)	H1(ref) AI(test)	H2(ref) AI(test)
False Negative Area (%)	32.1	40.6		
False Positive Area (%)	46.5	28.1		
|Relative Error (%)|	14.4	12.6		

Blinded Attending Surgeon Review						
Reviewer	PGT Estimate	H1 meets definition?	H2 meets definition?	AI meets definition?	Which is AI?	Which is most accurate?
1	0	Yes	Yes	Yes	0	0
2	0	Yes	No	No	H2	AI
3	0	Yes	No	No	H1	AI

© 2021 Howell RS et al. *JAMA Network Open.*

Wound EMR Information								
Sequential Number	Gender	Age	Wound Type	Wound Location	Wound Length (cm)	Wound Width (cm)	Wound Depth (cm)	Wound Area (cm^2)
75	M	74	VLU	LLE lat	2.5	2.2	0.1	5.50

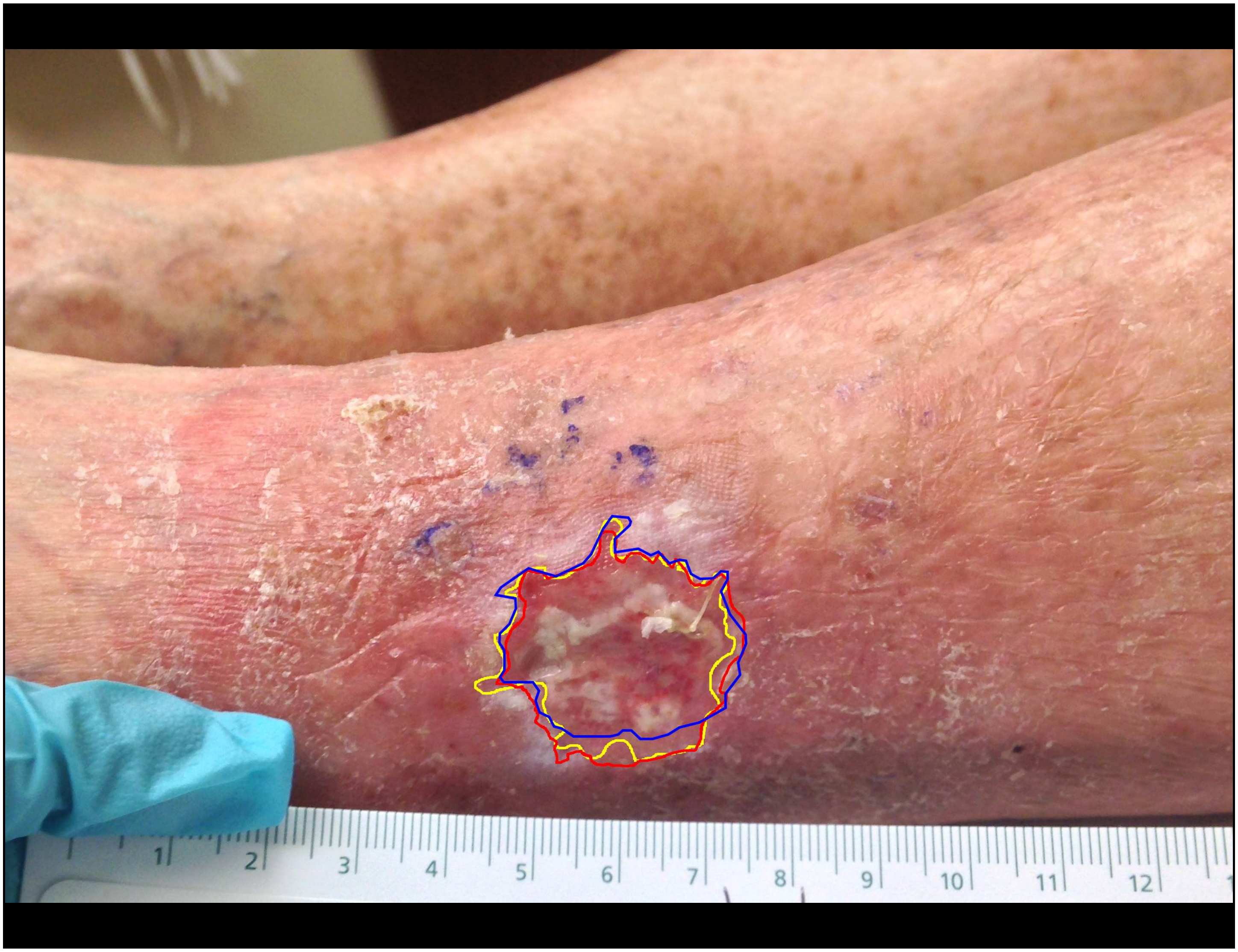

Tracing Data			
Tracer:	Wound Area (px^2)	Ruler Calibration (px/cm)	Wound Area (cm^2)
H1	145828	172.2	4.92
H2	153079	173.1	5.11
AI	145087	177.6	4.60

Tracing Comparisons				
Difference Metric:	Human-Human		Human-AI	
	H1(ref) H2(test)	H2(ref) H1(test)	H1(ref) AI(test)	H2(ref) AI(test)
False Negative Area (%)	4.9	9.4	8.7	11.7
False Positive Area (%)	9.9	4.7	8.2	6.5
|Relative Error (%)|	5.0	4.7	0.5	5.2

Blinded Attending Surgeon Review						
Reviewer	PGT Estimate	H1 meets definition?	H2 meets definition?	AI meets definition?	Which is AI?	Which is most accurate?
1	60	No	No	Yes	H2	H1
2	0	No	No	Yes	H2	H1
3	70	No	No	Yes	H1	H1

© 2021 Howell RS et al. *JAMA Network Open.*

Wound EMR Information								
Sequential Number	Gender	Age	Wound Type	Wound Location	Wound Length (cm)	Wound Width (cm)	Wound Depth (cm)	Wound Area (cm^2)
76	M	63	DFU	L plantar	3.4	2.5	0.3	8.50

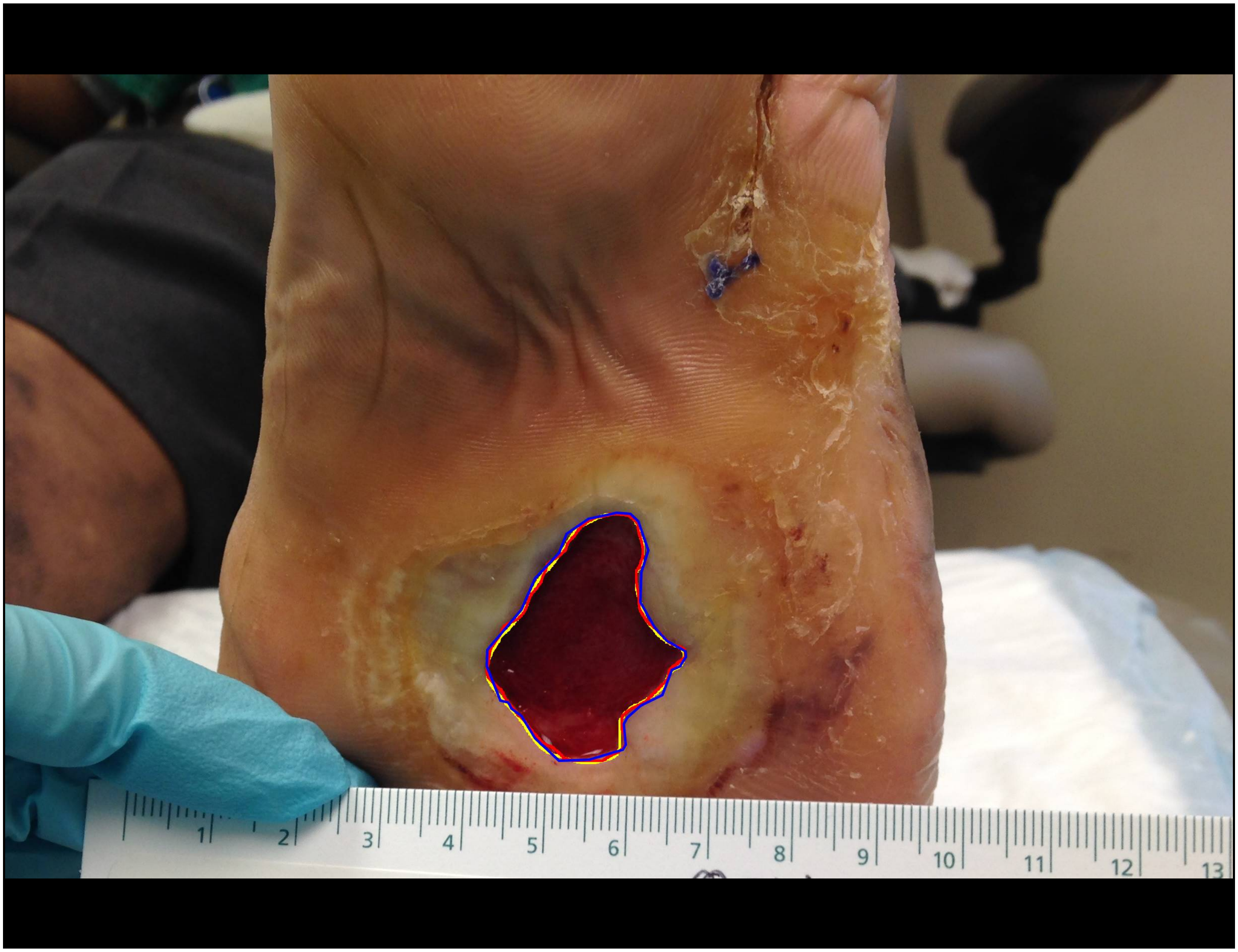

Tracing Data			
Tracer:	Wound Area (px^2)	Ruler Calibration (px/cm)	Wound Area (cm^2)
H1	113616	162.9	4.28
H2	112351	162.1	4.28
AI	117861	160.1	4.60

Tracing Comparisons				
Difference Metric:	Human-Human		Human-AI	
	H1(ref) H2(test)	H2(ref) H1(test)	H1(ref) AI(test)	H2(ref) AI(test)
False Negative Area (%)	2.7	1.6	1.4	0.6
False Positive Area (%)	1.6	2.7	5.2	5.5
|Relative Error (%)|	1.1	1.1	3.7	4.9

Blinded Attending Surgeon Review						
Reviewer	PGT Estimate	H1 meets definition?	H2 meets definition?	AI meets definition?	Which is AI?	Which is most accurate?
1	100	Yes	Yes	Yes	AI	AI
2	100	Yes	Yes	Yes	AI	AI
3	50	Yes	Yes	Yes	H2	H1

© 2021 Howell RS et al. *JAMA Network Open.*

Wound EMR Information								
Sequential Number	Gender	Age	Wound Type	Wound Location	Wound Length (cm)	Wound Width (cm)	Wound Depth (cm)	Wound Area (cm^2)
77	M	95	PU	LLE lat	0.7	0.6	0.2	0.42

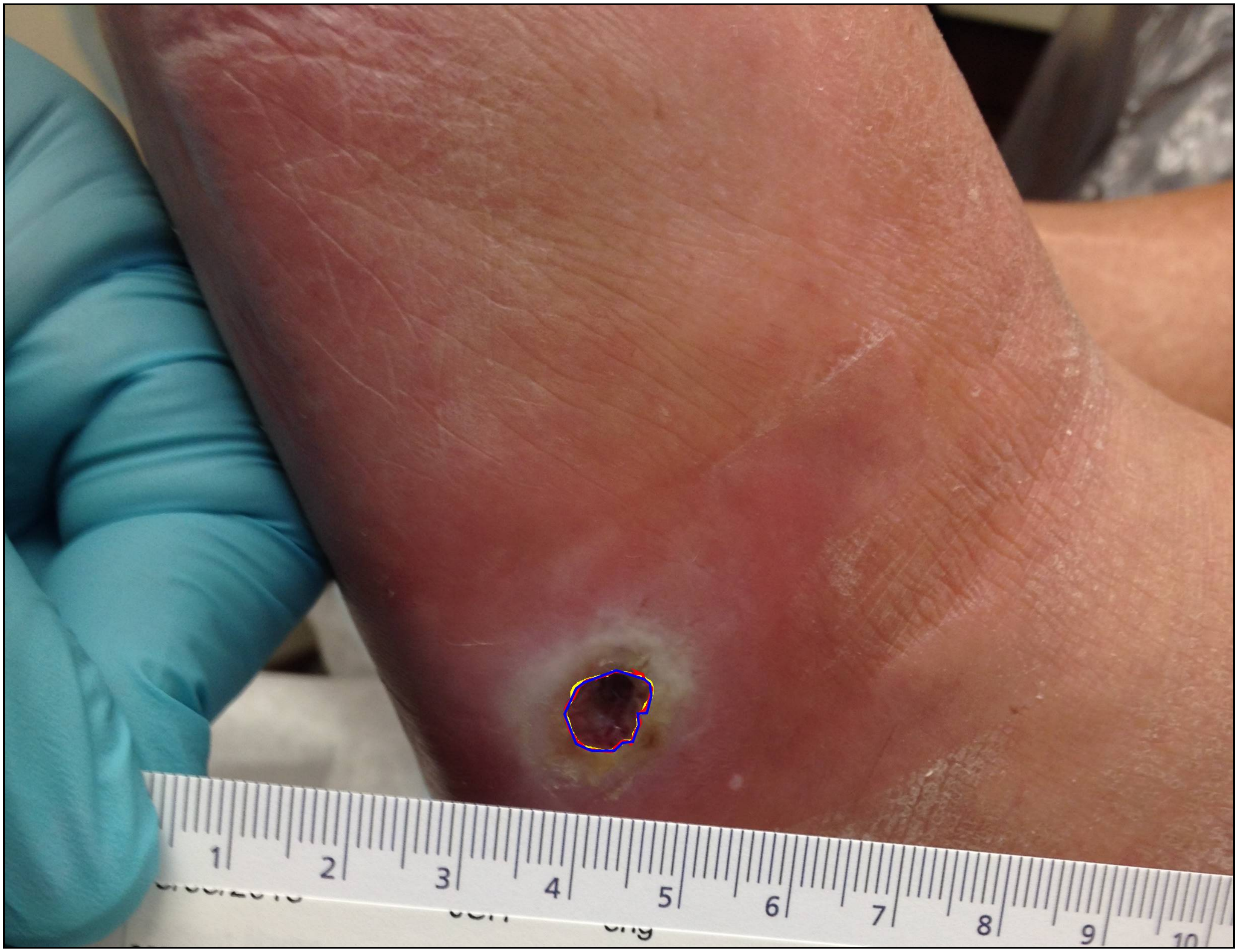

Tracing Data			
Tracer:	Wound Area (px^2)	Ruler Calibration (px/cm)	Wound Area (cm^2)
H1	19801	219.8	0.41
H2	19794	222.2	0.40
AI	20064	224.0	0.40

Tracing Comparisons				
Difference Metric:	Human-Human		Human-AI	
	H1(ref) H2(test)	H2(ref) H1(test)	H1(ref) AI(test)	H2(ref) AI(test)
False Negative Area (%)	3.1	3.1	2.4	2.2
False Positive Area (%)	3.1	3.1	3.7	3.5
|Relative Error (%)|	0.0	0.0	1.3	1.4

Blinded Attending Surgeon Review						
Reviewer	PGT Estimate	H1 meets definition?	H2 meets definition?	AI meets definition?	Which is AI?	Which is most accurate?
1	0	Yes	Yes	Yes	AI	H1
2		Yes	Yes	Yes	AI	H2
3	70	Yes	Yes	Yes	H1	AI

© 2021 Howell RS et al. *JAMA Network Open.*

Wound EMR Information								
Sequential Number	Gender	Age	Wound Type	Wound Location	Wound Length (cm)	Wound Width (cm)	Wound Depth (cm)	Wound Area (cm^2)
78	M	67	Arterial	RLE med	2.2	1.0	0.2	2.20

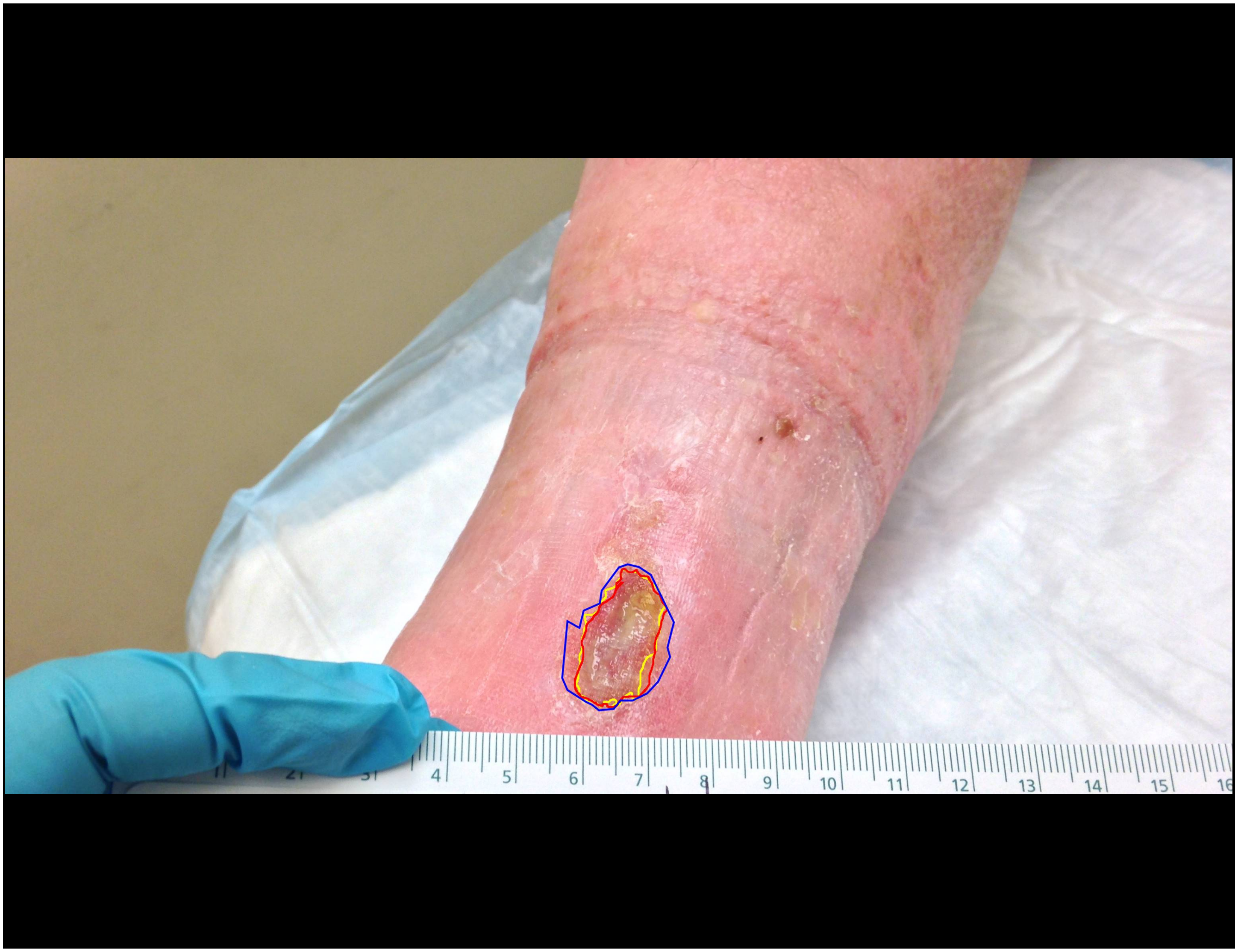

Tracing Data			
Tracer:	Wound Area (px^2)	Ruler Calibration (px/cm)	Wound Area (cm^2)
H1	55722	175.0	1.82
H2	57100	172.7	1.91
AI	78522	180.9	2.40

Tracing Comparisons				
Difference Metric:	Human-Human		Human-AI	
	H1(ref) H2(test)	H2(ref) H1(test)	H1(ref) AI(test)	H2(ref) AI(test)
False Negative Area (%)	4.5	6.8	0.1	0.1
False Positive Area (%)	6.9	4.4	41.0	37.6
|Relative Error (%)|	2.5	2.4	40.9	37.5

Blinded Attending Surgeon Review						
Reviewer	PGT Estimate	H1 meets definition?	H2 meets definition?	AI meets definition?	Which is AI?	Which is most accurate?
1	20	Yes	Yes	No	H2	AI
2	<10	Yes	Yes	No	H2	H2
3	20	Yes	Yes	No	H1	H2

© 2021 Howell RS et al. *JAMA Network Open.*

Wound EMR Information								
**Sequential Number**	**Gender**	**Age**	**Wound Type**	**Wound Location**	**Wound Length (cm)**	**Wound Width (cm)**	**Wound Depth (cm)**	**Wound Area (cm^2)**
79	F	85	Trauma	LLE lat	6.0	4.5	0.4	27.00

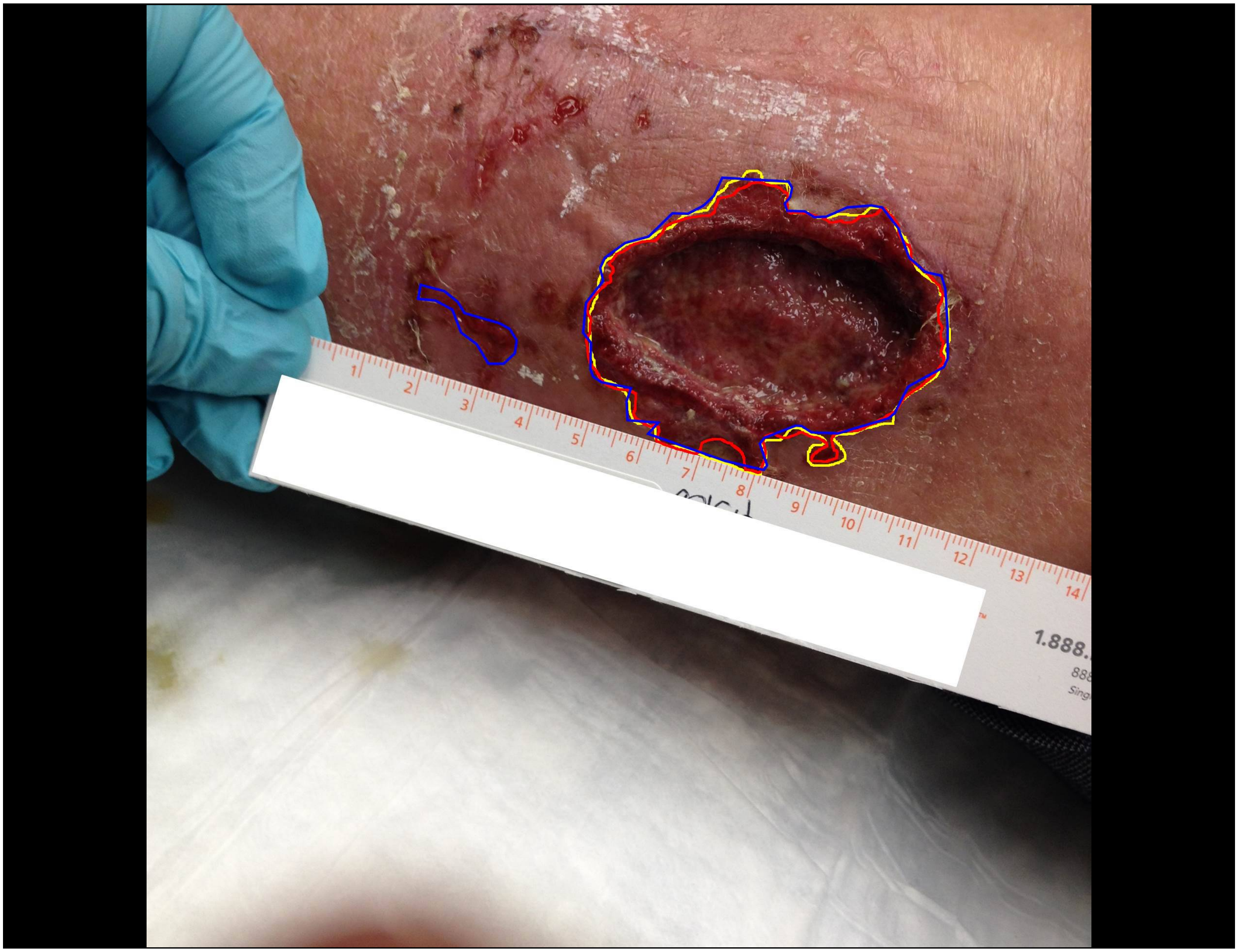

Tracing Data			
**Tracer:**	**Wound Area (px^2)**	**Ruler Calibration (px/cm)**	**Wound Area (cm^2)**
**H1**	309494	123.1	20.41
**H2**	295831	117.3	21.51
**AI**	317544	122.4	21.20

Tracing Comparisons				
**Difference Metric:**	**Human-Human**		**Human-AI**	
	**H1(ref) H2(test)**	**H2(ref) H1(test)**	**H1(ref) AI(test)**	**H2(ref) AI(test)**
**False Negative Area (%)**	5.4	1.0	3.1	2.1
**False Positive Area (%)**	1.0	5.6	5.7	9.4
**|Relative Error (%)|**	4.4	4.6	2.6	7.3

Blinded Attending Surgeon Review						
**Reviewer**	**PGT Estimate**	**H1 meets definition?**	**H2 meets definition?**	**AI meets definition?**	**Which is AI?**	**Which is most accurate?**
**1**		Yes	No	No	AI	H2
**2**	0	No	No	No	H1	AI
**3**	100	Yes	No	No	H1	H2

© 2021 Howell RS et al. *JAMA Network Open.*

Wound EMR Information								
Sequential Number	Gender	Age	Wound Type	Wound Location	Wound Length (cm)	Wound Width (cm)	Wound Depth (cm)	Wound Area (cm^2)
80	F	78	PU	R ankle	4.6	2.9	0.6	13.34

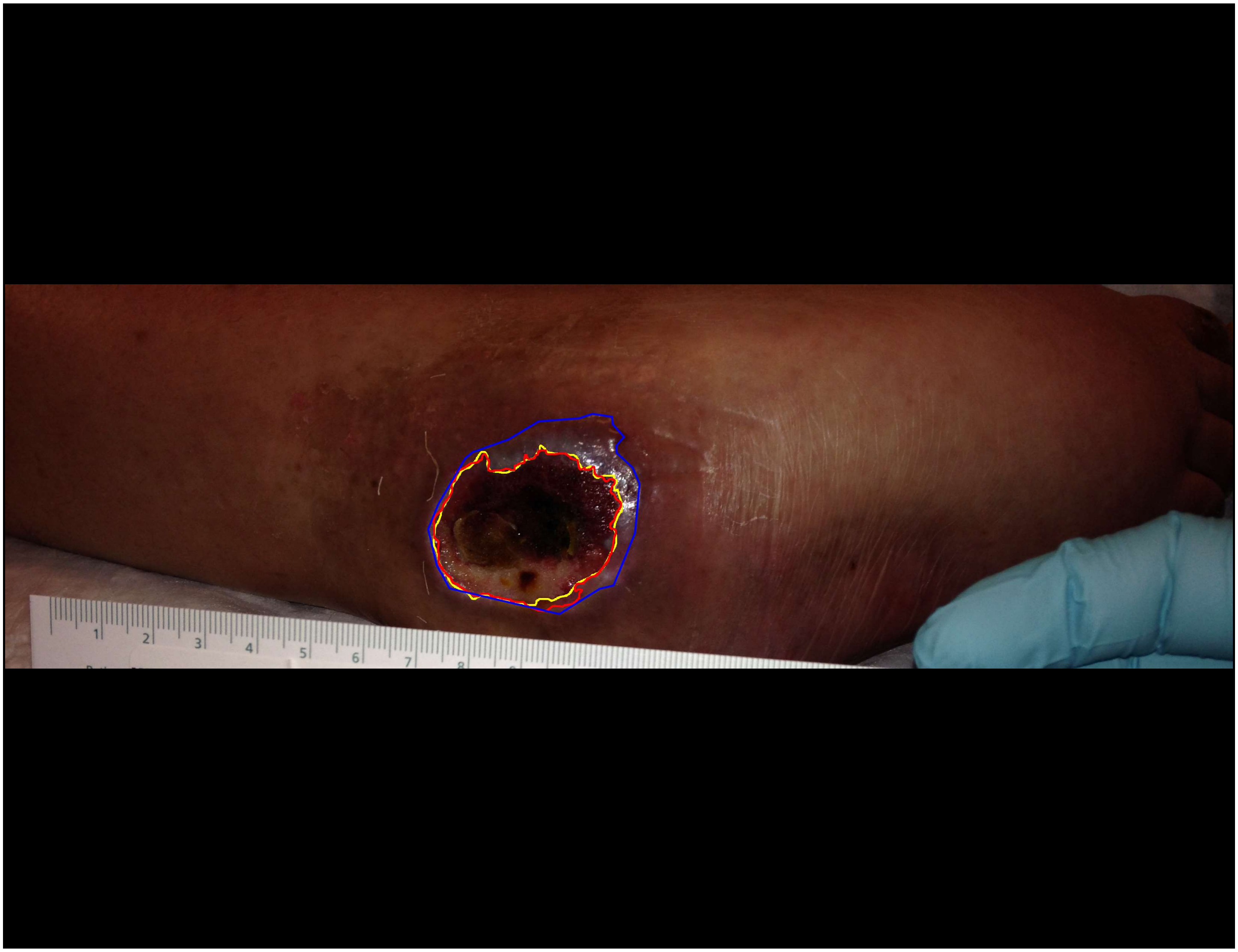

Tracing Data			
Tracer:	Wound Area (px^2)	Ruler Calibration (px/cm)	Wound Area (cm^2)
H1	154475	139.5	7.94
H2	155633	140.5	7.88
AI	219359	139.9	11.20

Tracing Comparisons				
Difference Metric:	Human-Human		Human-AI	
	H1(ref) H2(test)	H2(ref) H1(test)	H1(ref) AI(test)	H2(ref) AI(test)
False Negative Area (%)	2.7	3.4	0.2	0.1
False Positive Area (%)	3.4	2.7	42.2	41.0
|Relative Error (%)|	0.7	0.7	42.0	40.9

Blinded Attending Surgeon Review						
Reviewer	PGT Estimate	H1 meets definition?	H2 meets definition?	AI meets definition?	Which is AI?	Which is most accurate?
1	0	Yes	Yes	No	AI	AI
2	0	No	No	Yes	H2	H2
3	80	Yes	Yes	No	H2	H1

© 2021 Howell RS et al. *JAMA Network Open.*

Wound EMR Information								
Sequential Number	Gender	Age	Wound Type	Wound Location	Wound Length (cm)	Wound Width (cm)	Wound Depth (cm)	Wound Area (cm^2)
81	F	93	PU	L buttock	1.1	1.3	0.8	1.43

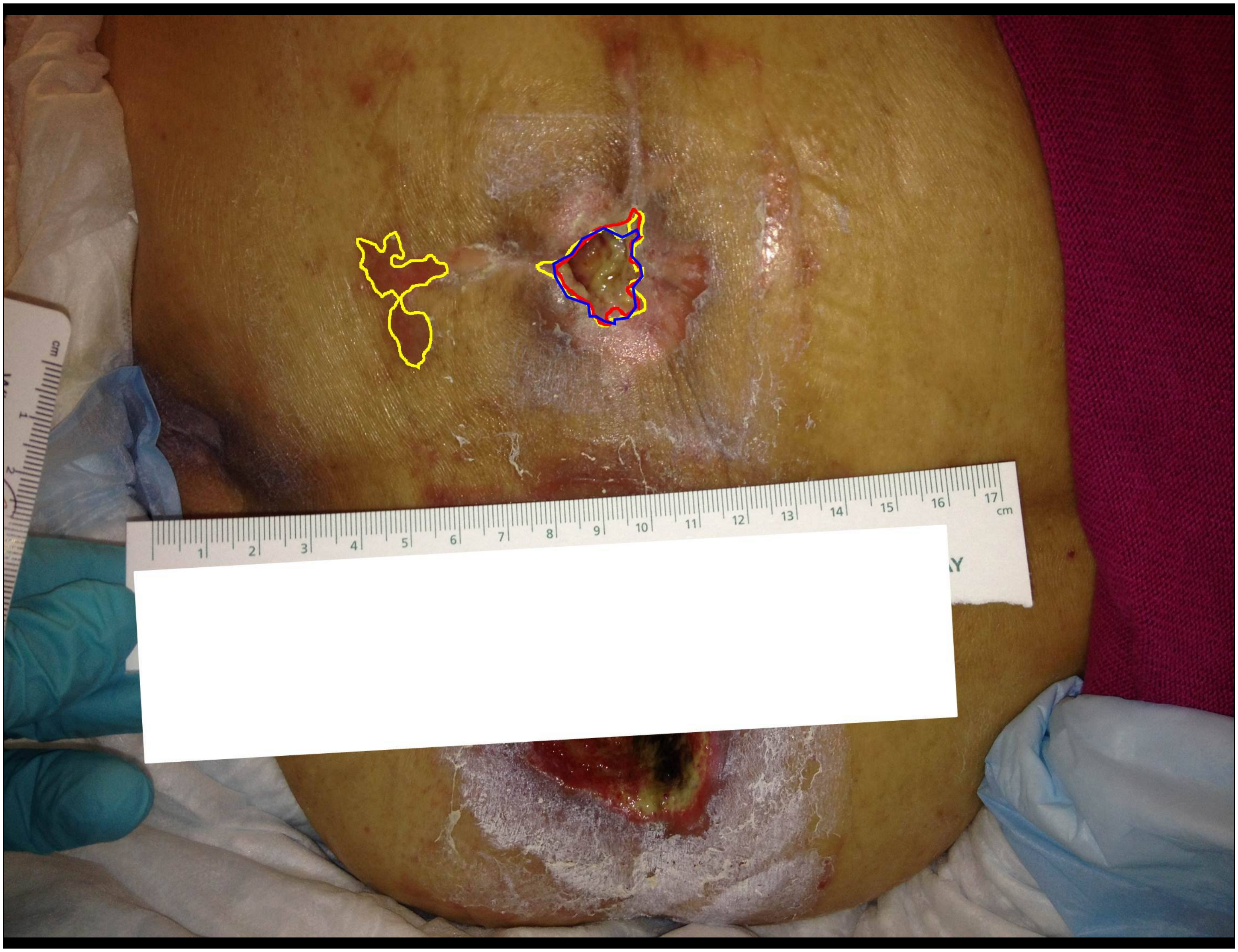

Tracing Data			
Tracer:	Wound Area (px^2)	Ruler Calibration (px/cm)	Wound Area (cm^2)
H1	36573	88.3	4.69
H2	18343	88.2	2.36
AI	18953	88.9	2.40

Tracing Comparisons				
Difference Metric:	Human-Human		Human-AI	
	H1(ref) H2(test)	H2(ref) H1(test)	H1(ref) AI(test)	H2(ref) AI(test)
False Negative Area (%)	53.2	6.7	50.3	9.3
False Positive Area (%)	3.4	106.1	2.1	12.7
|Relative Error (%)|	49.8	99.4	48.2	3.3

Blinded Attending Surgeon Review						
Reviewer	PGT Estimate	H1 meets definition?	H2 meets definition?	AI meets definition?	Which is AI?	Which is most accurate?
1	0	No	No	No	AI	H2
2	0	No	Yes	No	AI	H2
3	0	Yes	Yes	Yes	H1	H2

© 2021 Howell RS et al. *JAMA Network Open.*

Wound EMR Information								
Sequential Number	Gender	Age	Wound Type	Wound Location	Wound Length (cm)	Wound Width (cm)	Wound Depth (cm)	Wound Area (cm^2)
82	F	69	Surgical	chest	2.0	1.5	0.1	3.00

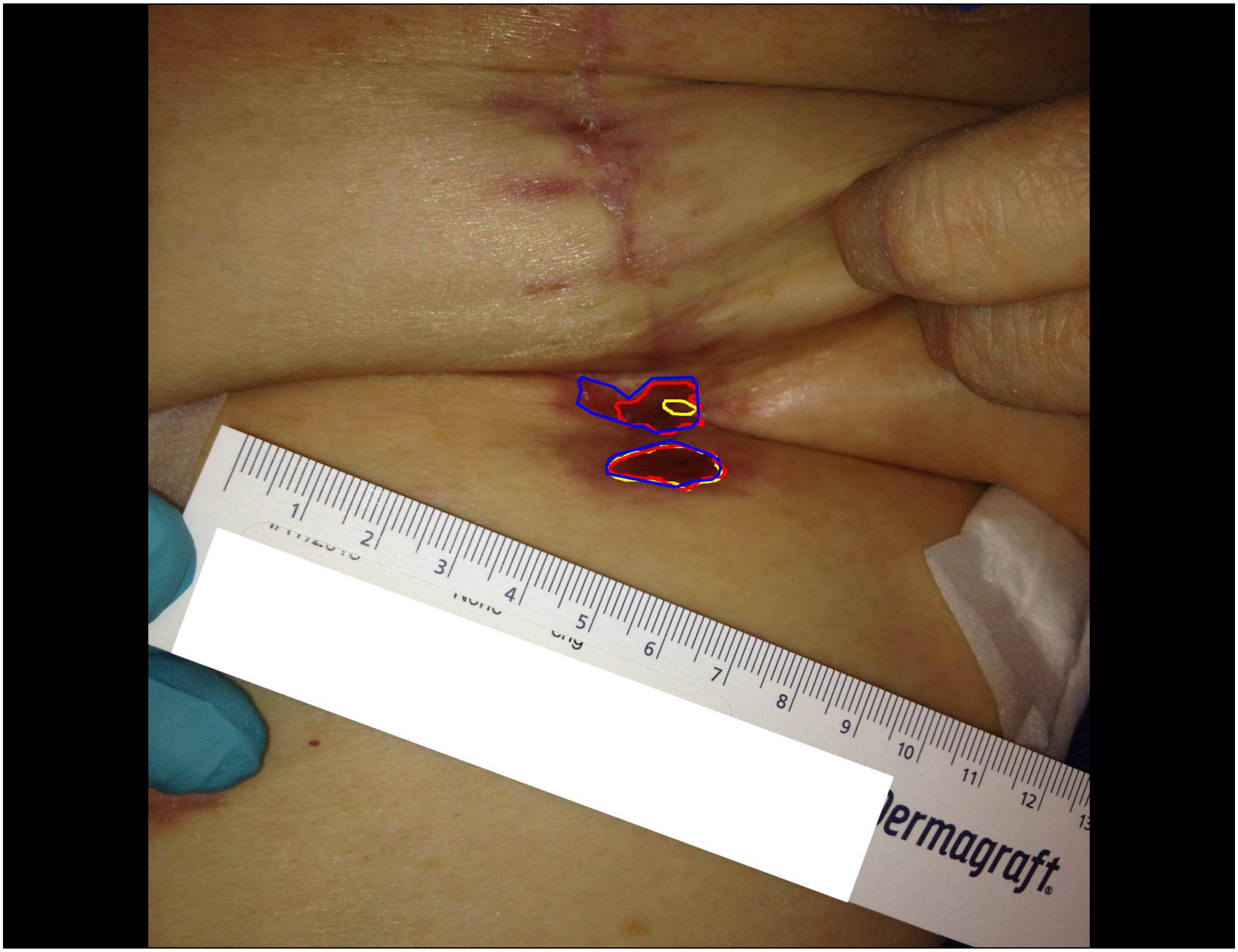

Tracing Data			
Tracer:	Wound Area (px^2)	Ruler Calibration (px/cm)	Wound Area (cm^2)
H1	10516	128.2	0.64
H2	17586	125.3	1.12
AI	24087	136.1	1.30

Tracing Comparisons				
Difference Metric:	Human-Human		Human-AI	
	H1(ref) H2(test)	H2(ref) H1(test)	H1(ref) AI(test)	H2(ref) AI(test)
False Negative Area (%)	8.4	45.2	5.3	4.0
False Positive Area (%)	75.6	5.0	134.3	41.0
|Relative Error (%)|	67.2	40.2	129.1	37.0

Blinded Attending Surgeon Review						
Reviewer	PGT Estimate	H1 meets definition?	H2 meets definition?	AI meets definition?	Which is AI?	Which is most accurate?
1		No	No	No	H1	None
2	0	No	No	Yes	H2	AI
3	30	No	Yes	Yes	AI	AI

© 2021 Howell RS et al. *JAMA Network Open.*

Wound EMR Information								
Sequential Number	Gender	Age	Wound Type	Wound Location	Wound Length (cm)	Wound Width (cm)	Wound Depth (cm)	Wound Area (cm^2)
83	F	87	PU	R ankle	0.6	0.5	0.1	0.30

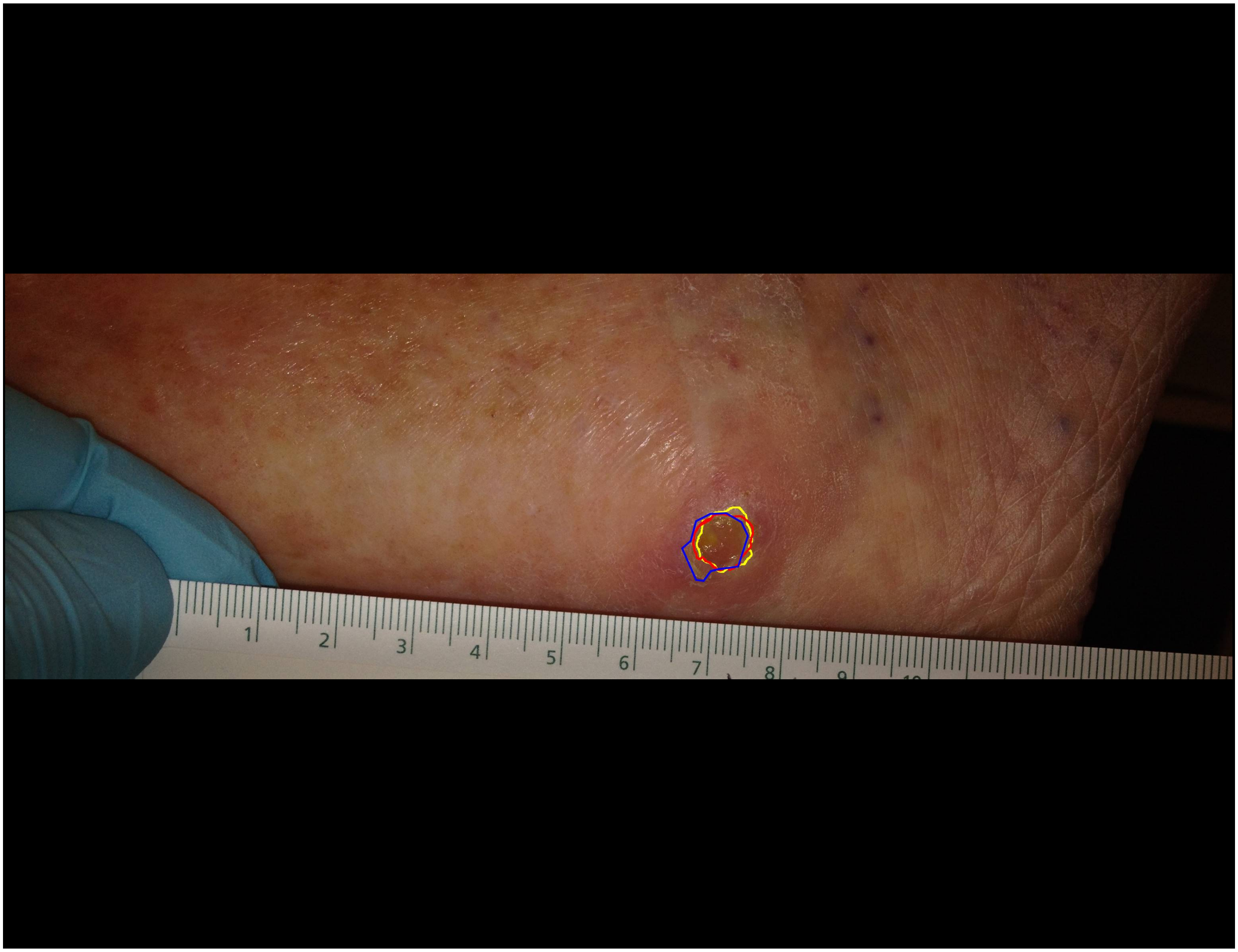

Tracing Data			
Tracer:	Wound Area (px^2)	Ruler Calibration (px/cm)	Wound Area (cm^2)
H1	18240	194.9	0.48
H2	17511	194.5	0.46
AI	20614	203.0	0.50

Tracing Comparisons				
Difference Metric:	Human-Human		Human-AI	
	H1(ref) H2(test)	H2(ref) H1(test)	H1(ref) AI(test)	H2(ref) AI(test)
False Negative Area (%)	12.1	8.4	18.5	10.5
False Positive Area (%)	8.1	12.6	31.6	28.2
|Relative Error (%)|	4.0	4.2	13.0	17.7

Blinded Attending Surgeon Review						
Reviewer	PGT Estimate	H1 meets definition?	H2 meets definition?	AI meets definition?	Which is AI?	Which is most accurate?
1	0	No	Yes	Yes	H1	H1
2	0	No	Yes	No	AI	H1
3	0	Yes	Yes	No	AI	H1

© 2021 Howell RS et al. *JAMA Network Open.*

Wound EMR Information								
Sequential Number	Gender	Age	Wound Type	Wound Location	Wound Length (cm)	Wound Width (cm)	Wound Depth (cm)	Wound Area (cm^2)
84	F	87	Trauma	LLE ant	0.4	0.6	0.1	0.24

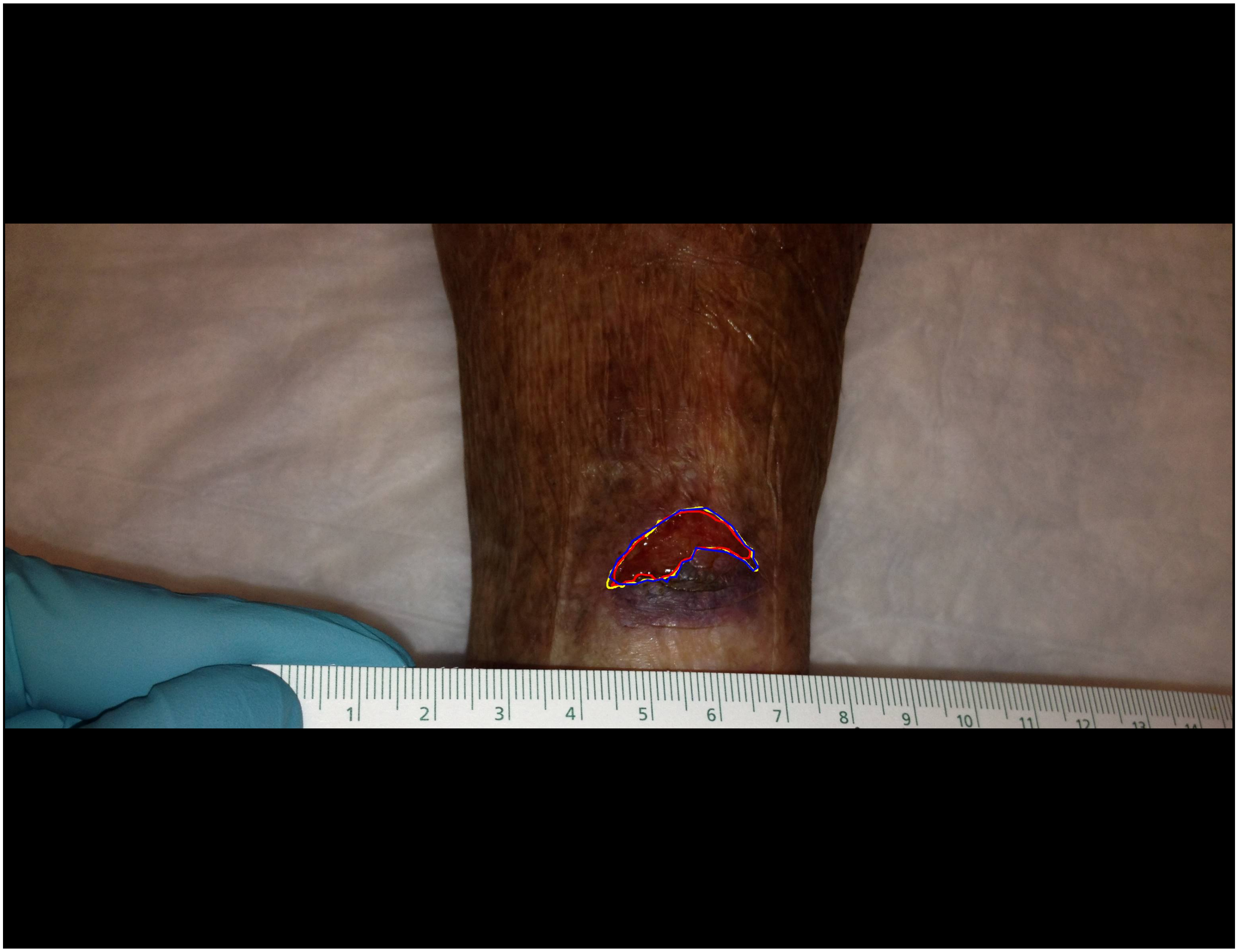

Tracing Data			
Tracer:	Wound Area (px^2)	Ruler Calibration (px/cm)	Wound Area (cm^2)
H1	38774	181.3	1.18
H2	34279	180.7	1.05
AI	40530	201.3	1.00

Tracing Comparisons				
Difference Metric:	Human-Human		Human-AI	
	H1(ref) H2(test)	H2(ref) H1(test)	H1(ref) AI(test)	H2(ref) AI(test)
False Negative Area (%)	12.8	1.3	2.9	0.7
False Positive Area (%)	1.2	14.4	7.4	18.9
|Relative Error (%)|	11.6	13.1	4.5	18.2

Blinded Attending Surgeon Review						
Reviewer	PGT Estimate	H1 meets definition?	H2 meets definition?	AI meets definition?	Which is AI?	Which is most accurate?
1	80	Yes	Yes	Yes	AI	H1
2	10	Yes	Yes	Yes	AI	H2
3	50	Yes	Yes	Yes	H2	AI

© 2021 Howell RS et al. *JAMA Network Open.*

Wound EMR Information								
Sequential Number	Gender	Age	Wound Type	Wound Location	Wound Length (cm)	Wound Width (cm)	Wound Depth (cm)	Wound Area (cm^2)
85	M	43	PU	sacrum	2.1	0.9	1.5	1.89

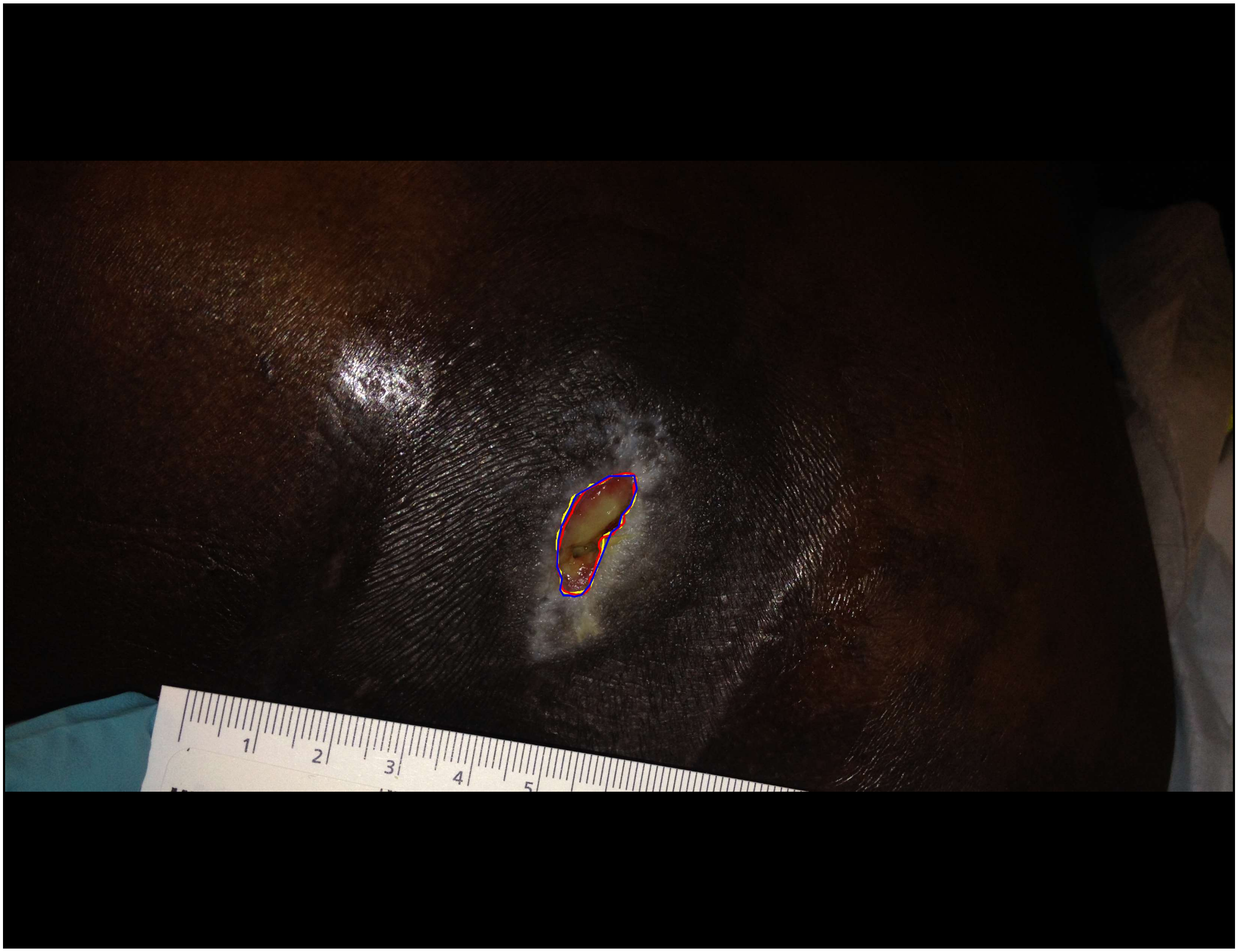

Tracing Data			
Tracer:	Wound Area (px^2)	Ruler Calibration (px/cm)	Wound Area (cm^2)
H1	37979	193.0	1.02
H2	35586	184.4	1.05
AI	38231	195.5	1.00

Tracing Comparisons				
Difference Metric:	Human-Human		Human-AI	
	H1(ref) H2(test)	H2(ref) H1(test)	H1(ref) AI(test)	H2(ref) AI(test)
False Negative Area (%)	8.6	2.5	3.7	2.3
False Positive Area (%)	2.3	9.2	4.4	9.8
|Relative Error (%)|	6.3	6.7	0.7	7.4

Blinded Attending Surgeon Review						
Reviewer	PGT Estimate	H1 meets definition?	H2 meets definition?	AI meets definition?	Which is AI?	Which is most accurate?
1	20	Yes	Yes	Yes	AI	H2
2	<10	No	Yes	No	AI	H2
3	20	Yes	Yes	Yes	H1	H2

© 2021 Howell RS et al. *JAMA Network Open.*

Wound EMR Information								
Sequential Number	Gender	Age	Wound Type	Wound Location	Wound Length (cm)	Wound Width (cm)	Wound Depth (cm)	Wound Area (cm^2)
86	M	63	VLU	LLE lat	7.8	11.4	0.2	88.92

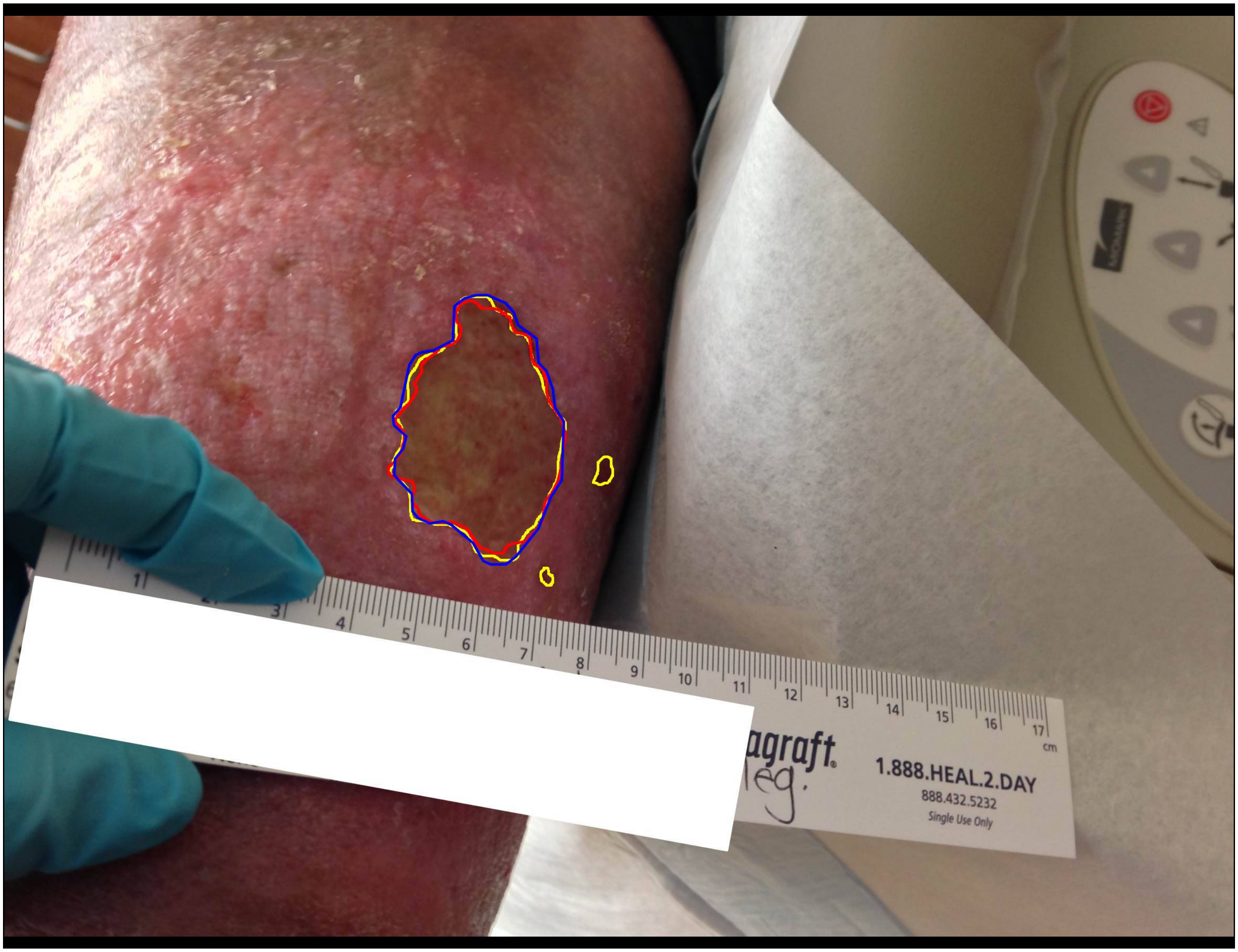

Tracing Data			
Tracer:	Wound Area (px^2)	Ruler Calibration (px/cm)	Wound Area (cm^2)
H1	110226	116.7	8.09
H2	104329	114.8	7.92
AI	114970	117.0	8.40

Tracing Comparisons				
Difference Metric:	Human-Human		Human-AI	
	H1(ref) H2(test)	H2(ref) H1(test)	H1(ref) AI(test)	H2(ref) AI(test)
False Negative Area (%)	7.1	1.8	2.6	0.4
False Positive Area (%)	1.7	7.5	7.0	10.6
|Relative Error (%)|	5.3	5.7	4.3	10.2

Blinded Attending Surgeon Review						
Reviewer	PGT Estimate	H1 meets definition?	H2 meets definition?	AI meets definition?	Which is AI?	Which is most accurate?
1	0	No	Yes	Yes	AI	H2
2	0	Yes	No	No	AI	AI
3	0	Yes	Yes	Yes	H2	H1

© 2021 Howell RS et al. *JAMA Network Open.*

Wound EMR Information								
Sequential Number	Gender	Age	Wound Type	Wound Location	Wound Length (cm)	Wound Width (cm)	Wound Depth (cm)	Wound Area (cm^2)
87	F	66	VLU	LLE medial	2.5	1.1	0.1	2.75

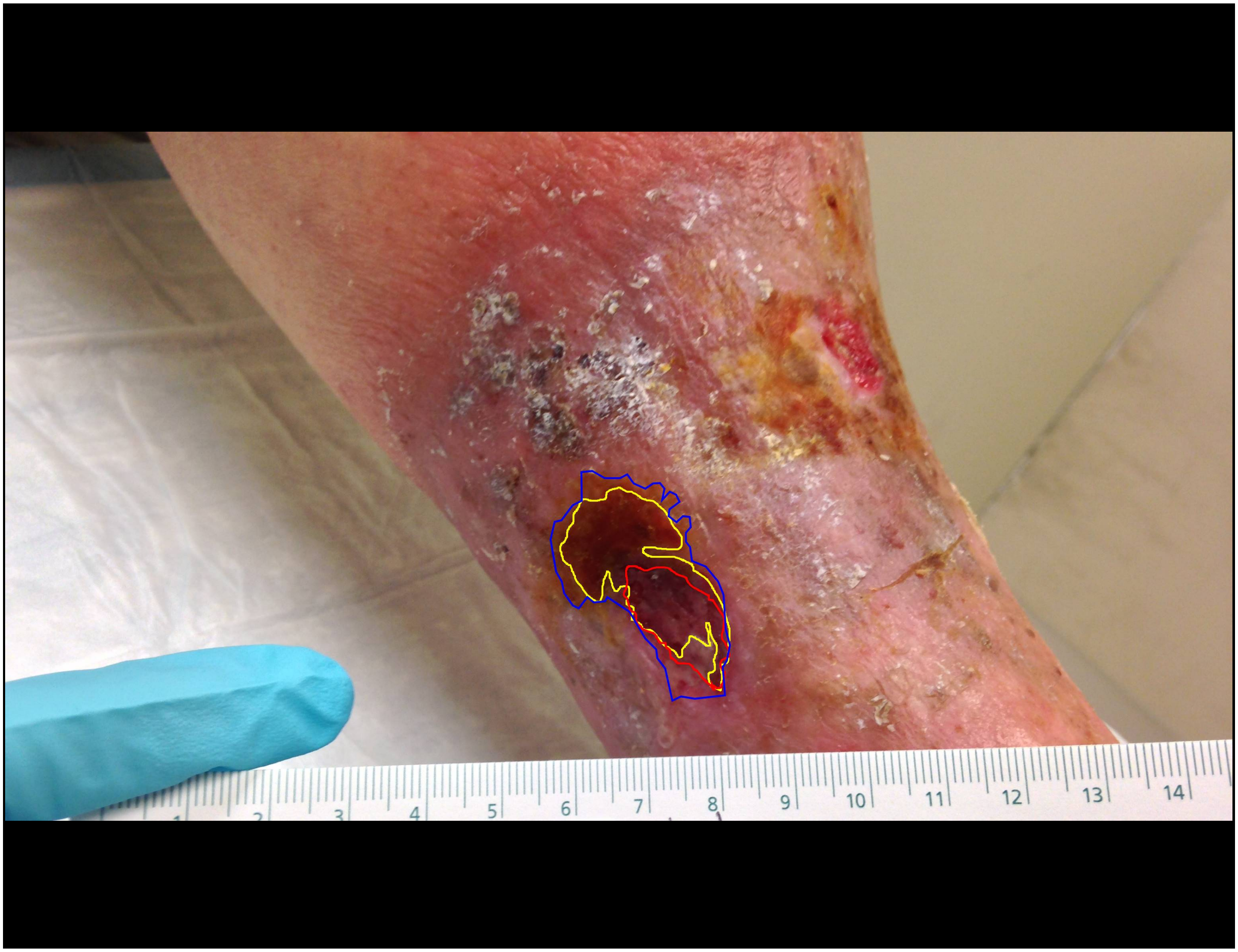

Tracing Data			
Tracer:	Wound Area (px^2)	Ruler Calibration (px/cm)	Wound Area (cm^2)
H1	107222	194.6	2.83
H2	51012	194.0	1.36
AI	161444	203.5	3.90

Tracing Comparisons				
Difference Metric:	Human-Human		Human-AI	
	H1(ref) H2(test)	H2(ref) H1(test)	H1(ref) AI(test)	H2(ref) AI(test)
False Negative Area (%)	60.8	17.6	0.5	0.2
False Positive Area (%)	8.4	127.8	51.0	216.7
|Relative Error (%)|	52.4	110.2	50.6	216.5

Blinded Attending Surgeon Review						
Reviewer	PGT Estimate	H1 meets definition?	H2 meets definition?	AI meets definition?	Which is AI?	Which is most accurate?
1	0	No	Yes	No	H2	H2
2	0	No	Yes	No	AI	H2
3	30	Yes	No	No	AI	H1

© 2021 Howell RS et al. *JAMA Network Open.*

Wound EMR Information								
Sequential Number	Gender	Age	Wound Type	Wound Location	Wound Length (cm)	Wound Width (cm)	Wound Depth (cm)	Wound Area (cm^2)
88	M	75	Surgical	R flank	0.3	2.5	0.4	0.75

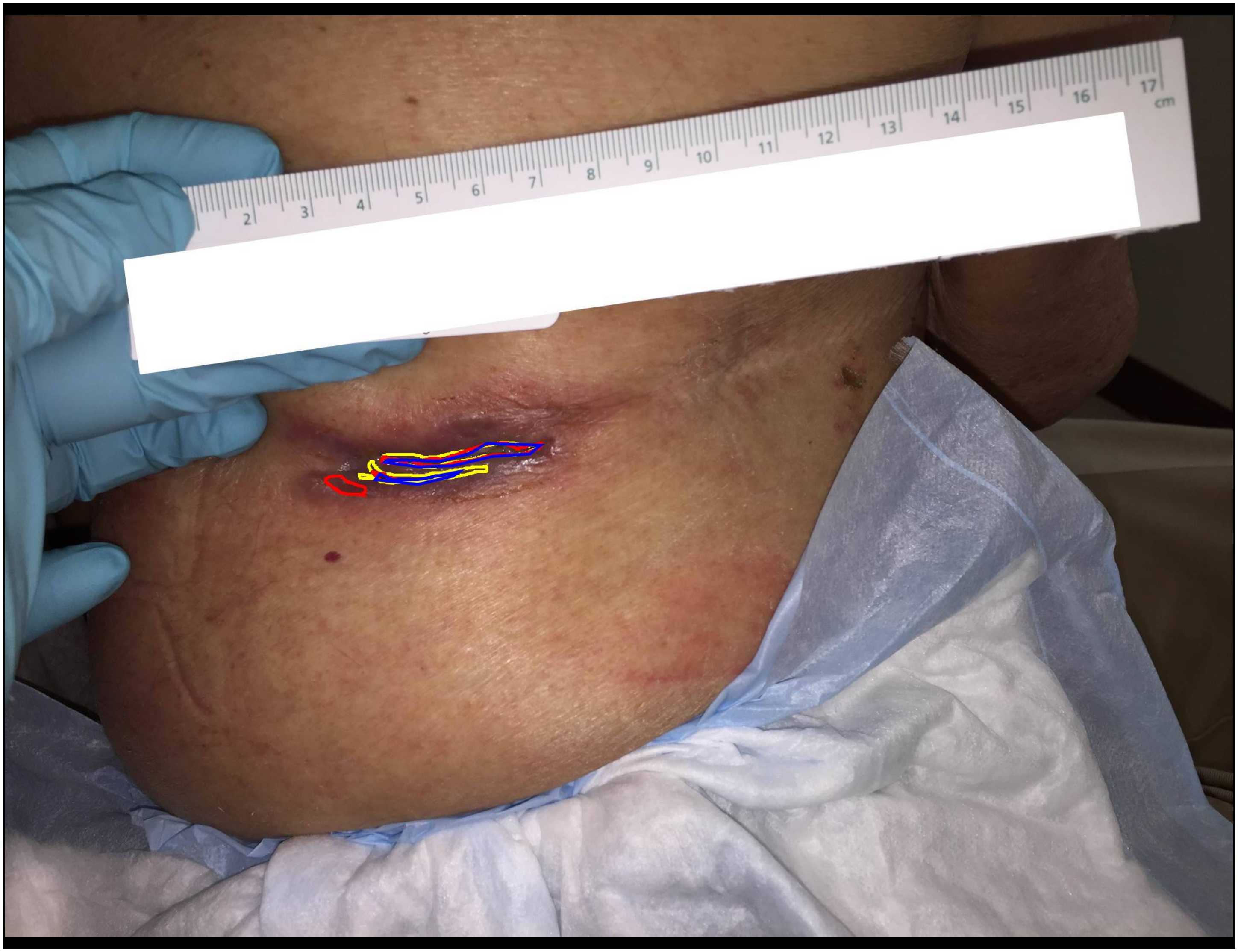

Tracing Data			
Tracer:	Wound Area (px^2)	Ruler Calibration (px/cm)	Wound Area (cm^2)
H1	6910	99.4	0.70
H2	4814	98.9	0.49
AI	4428	105.2	0.40

Tracing Comparisons				
Difference Metric:	Human-Human		Human-AI	
	H1(ref) H2(test)	H2(ref) H1(test)	H1(ref) AI(test)	H2(ref) AI(test)
False Negative Area (%)	53.5	33.3	47.9	41.7
False Positive Area (%)	23.2	76.8	12.0	33.7
|Relative Error (%)|	30.3	43.5	35.9	8.0

Blinded Attending Surgeon Review						
Reviewer	PGT Estimate	H1 meets definition?	H2 meets definition?	AI meets definition?	Which is AI?	Which is most accurate?
1	0	Yes	Yes	Yes	H2	0
2	0	No	Yes	No	AI	H2
3	0	Yes	No	No	AI	H1

© 2021 Howell RS et al. *JAMA Network Open.*

Wound EMR Information								
Sequential Number	Gender	Age	Wound Type	Wound Location	Wound Length (cm)	Wound Width (cm)	Wound Depth (cm)	Wound Area (cm^2)
89	F	33	VLU	LLE lat	9.0	10.0	0.2	90.00

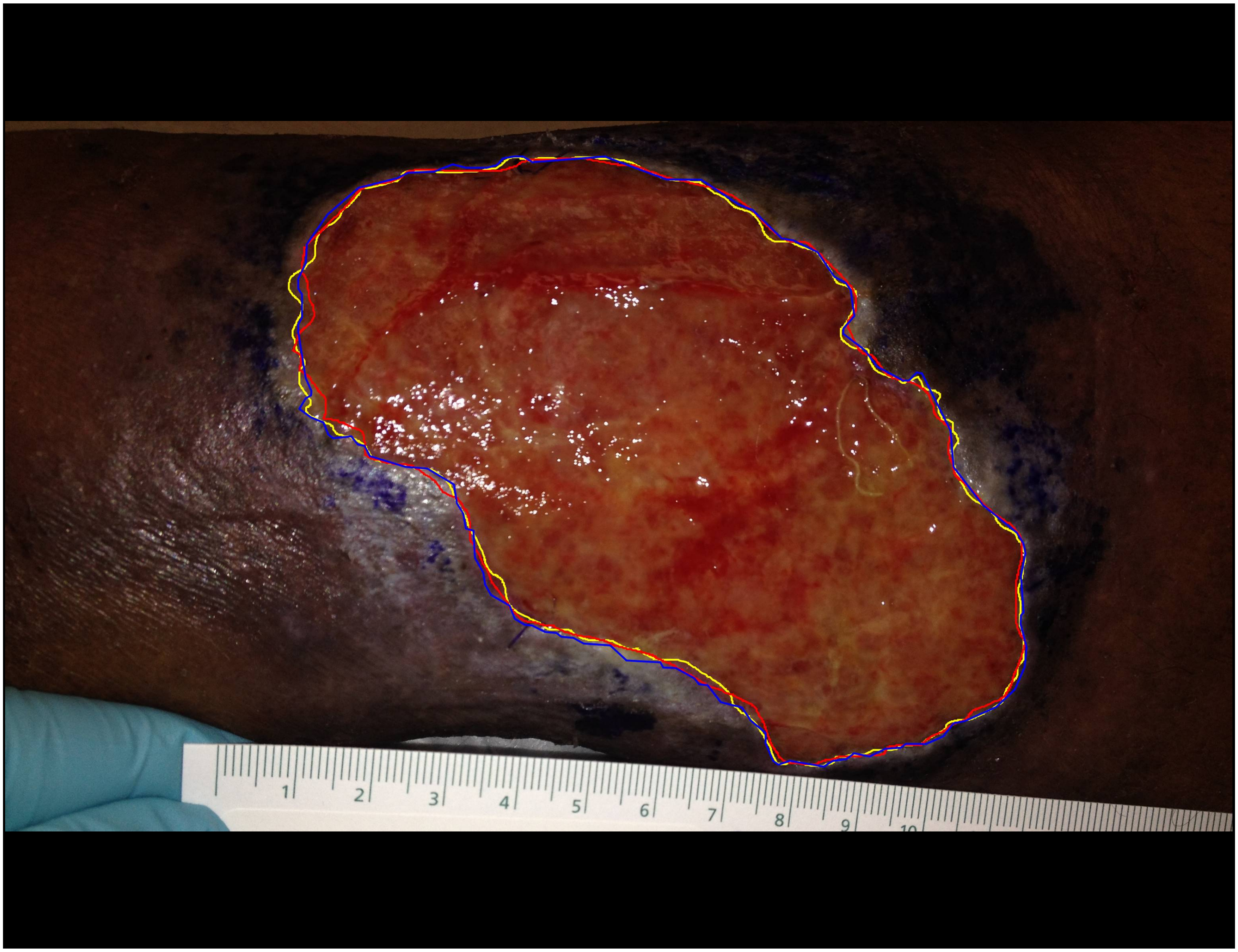

Tracing Data			
Tracer:	Wound Area (px^2)	Ruler Calibration (px/cm)	Wound Area (cm^2)
H1	1961554	204.2	47.06
H2	1955125	189.4	54.51
AI	1993179	211.4	44.60

Tracing Comparisons				
Difference Metric:	Human-Human		Human-AI	
	H1(ref) H2(test)	H2(ref) H1(test)	H1(ref) AI(test)	H2(ref) AI(test)
False Negative Area (%)	1.7	1.3	0.5	0.6
False Positive Area (%)	1.3	1.7	2.1	2.5
|Relative Error (%)|	0.3	0.3	1.6	1.9

Blinded Attending Surgeon Review						
Reviewer	PGT Estimate	H1 meets definition?	H2 meets definition?	AI meets definition?	Which is AI?	Which is most accurate?
1	0	Yes	Yes	Yes	H1	H2
2	<10	Yes	Yes	Yes	H2	AI
3	100	Yes	Yes	Yes	AI	H1

© 2021 Howell RS et al. *JAMA Network Open.*

Wound EMR Information								
Sequential Number	Gender	Age	Wound Type	Wound Location	Wound Length (cm)	Wound Width (cm)	Wound Depth (cm)	Wound Area (cm^2)
90	F	70	VLU	R foot dorsal	3.5	5.0	0.2	17.50

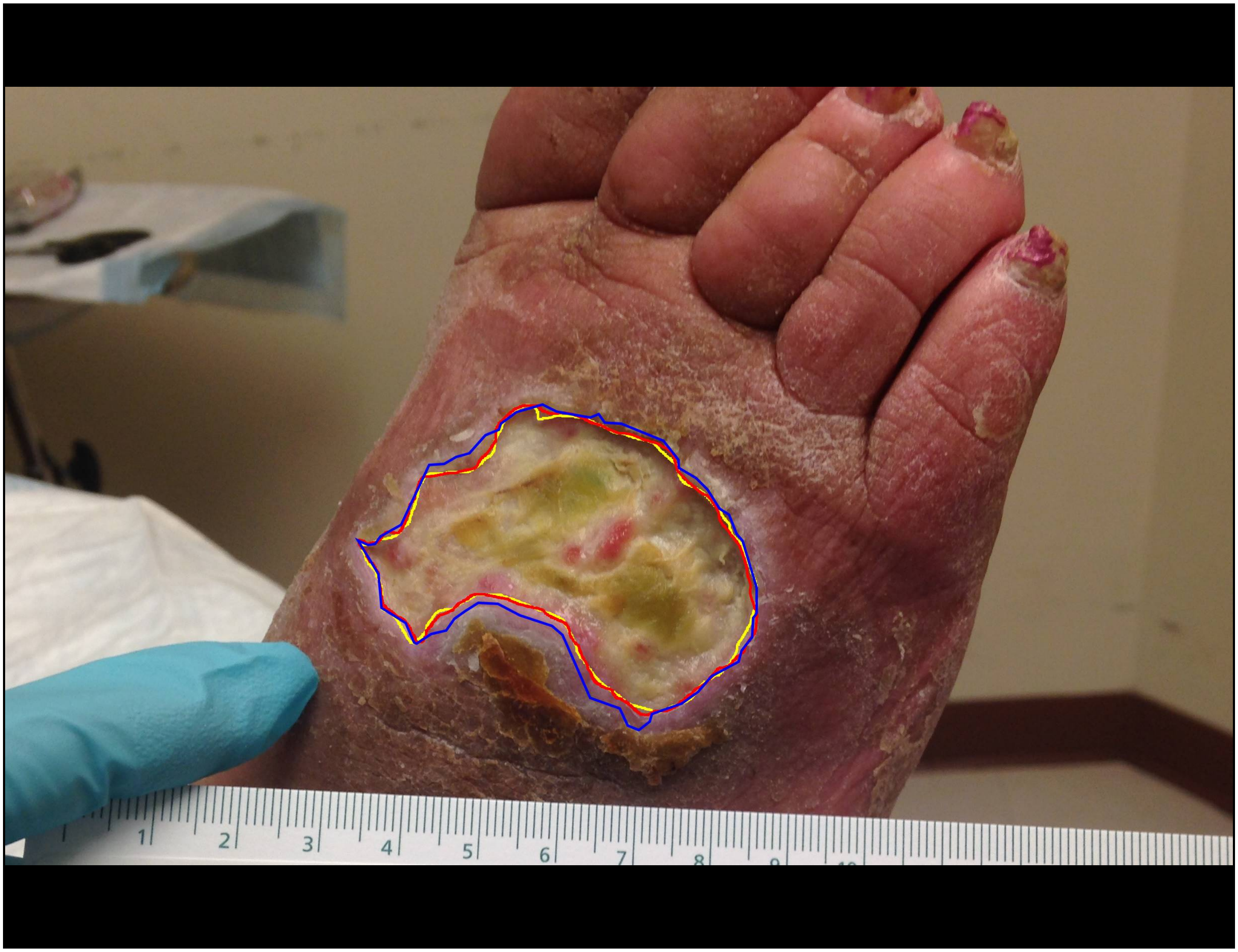

Tracing Data			
Tracer:	Wound Area (px^2)	Ruler Calibration (px/cm)	Wound Area (cm^2)
H1	272164	155.1	11.31
H2	275202	157.3	11.13
AI	297816	164.5	11.00

Tracing Comparisons				
Difference Metric:	Human-Human		Human-AI	
	H1(ref) H2(test)	H2(ref) H1(test)	H1(ref) AI(test)	H2(ref) AI(test)
False Negative Area (%)	1.4	2.5	0.5	0.4
False Positive Area (%)	2.5	1.4	9.9	8.7
|Relative Error (%)|	1.1	1.1	9.4	8.2

Blinded Attending Surgeon Review						
Reviewer	PGT Estimate	H1 meets definition?	H2 meets definition?	AI meets definition?	Which is AI?	Which is most accurate?
1	<10	Yes	Yes	Yes	H1	H2
2	<10	Yes	No	Yes	AI	H1
3	0	No	No	No	AI	H1

© 2021 Howell RS et al. *JAMA Network Open.*

Wound EMR Information								
Sequential Number	Gender	Age	Wound Type	Wound Location	Wound Length (cm)	Wound Width (cm)	Wound Depth (cm)	Wound Area (cm^2)
91	M	80	Trauma	L forearm	2.0	2.0	0.1	4.00

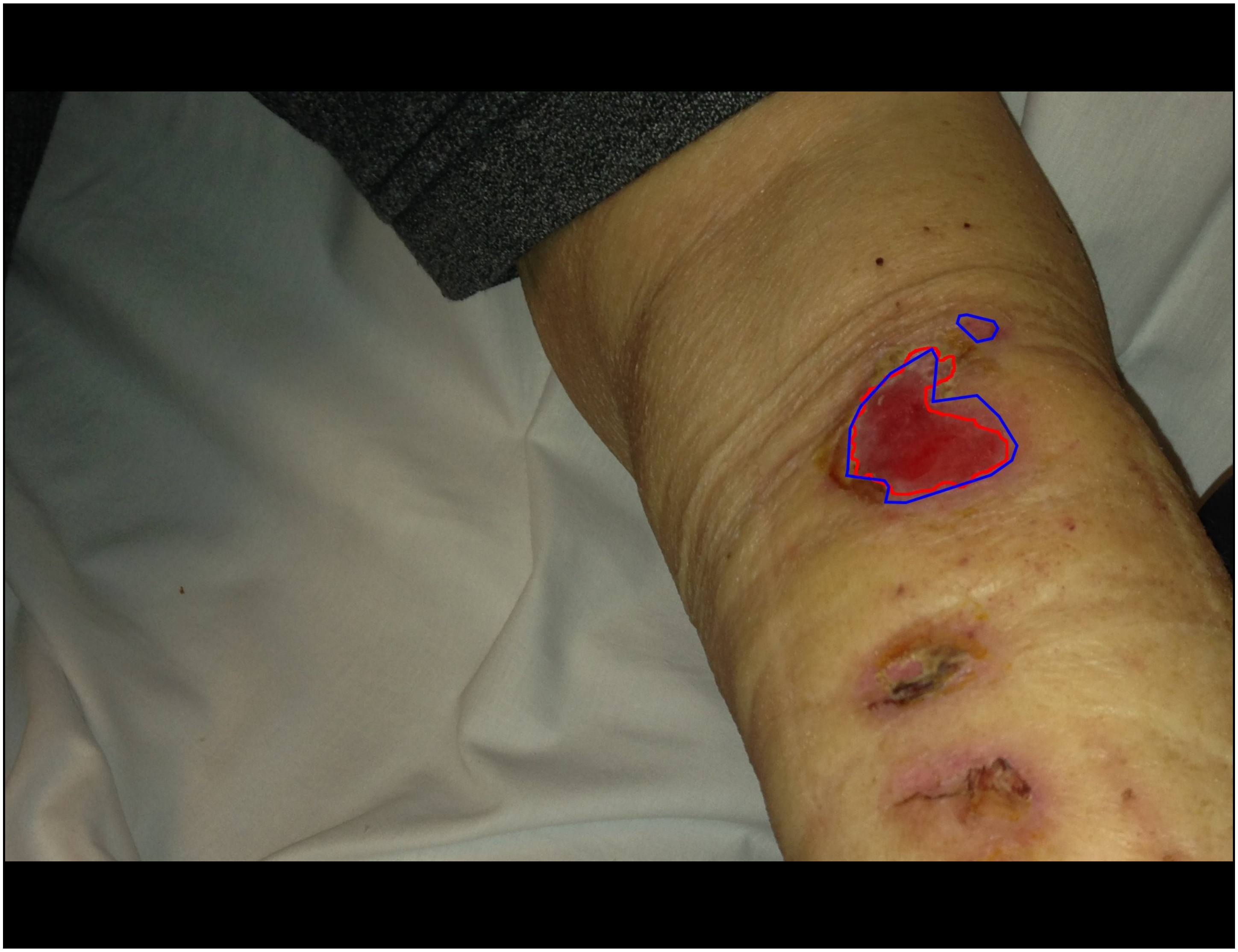

Tracing Data			
Tracer:	Wound Area (px^2)	Ruler Calibration (px/cm)	Wound Area (cm^2)
H1	23603	163.8	0.88
H2	41347	145.6	1.95
AI	50518	151.5	2.20

Tracing Comparisons				
Difference Metric:	Human-Human		Human-AI	
	H1(ref) H2(test)	H2(ref) H1(test)	H1(ref) AI(test)	H2(ref) AI(test)
False Negative Area (%)	100.0	100.0	100.0	5.2
False Positive Area (%)	175.2	57.1	214.0	27.4
|Relative Error (%)|	75.2	42.9	114.0	22.2

Blinded Attending Surgeon Review						
Reviewer	PGT Estimate	H1 meets definition?	H2 meets definition?	AI meets definition?	Which is AI?	Which is most accurate?
1	0	No	Yes	No	H1	H1
2	100	No	No	Yes	AI	AI
3	80	No	No	Yes	AI	AI

© 2021 Howell RS et al. *JAMA Network Open.*

Wound EMR Information								
Sequential Number	Gender	Age	Wound Type	Wound Location	Wound Length (cm)	Wound Width (cm)	Wound Depth (cm)	Wound Area (cm^2)
92	F	86	VLU	R ankle	0.6	0.5	0.2	0.30

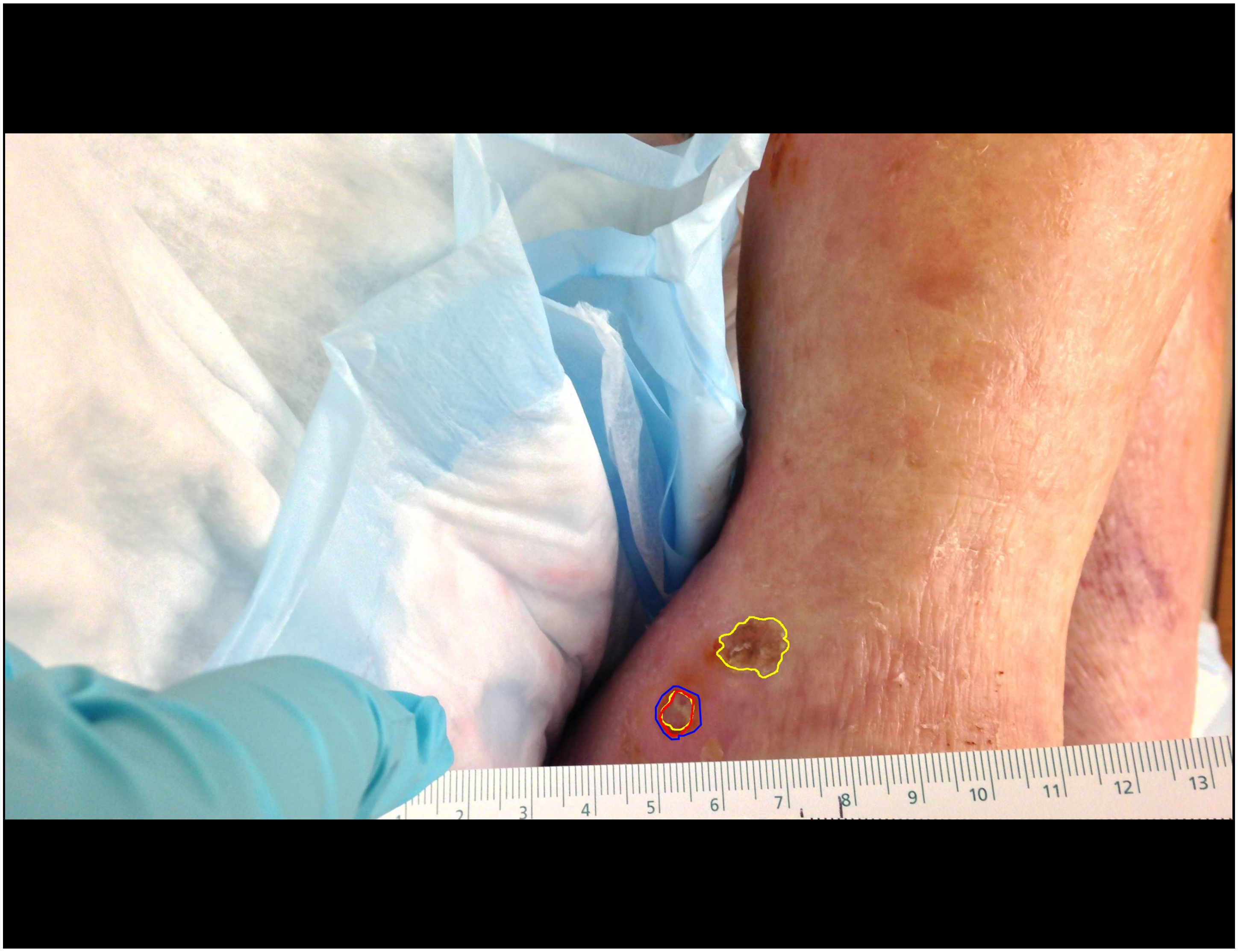

Tracing Data			
Tracer:	Wound Area (px^2)	Ruler Calibration (px/cm)	Wound Area (cm^2)
H1	27091	169.8	0.94
H2	7108	169.0	0.25
AI	12814	179.0	0.40

Tracing Comparisons				
Difference Metric:	Human-Human		Human-AI	
	H1(ref) H2(test)	H2(ref) H1(test)	H1(ref) AI(test)	H2(ref) AI(test)
False Negative Area (%)	78.0	16.2	77.0	0.0
False Positive Area (%)	4.2	297.3	24.3	80.3
|Relative Error (%)|	73.8	281.1	52.7	80.3

Blinded Attending Surgeon Review						
Reviewer	PGT Estimate	H1 meets definition?	H2 meets definition?	AI meets definition?	Which is AI?	Which is most accurate?
1	0	No	Yes	No	H2	AI
2	0	No	Yes	No	H2	AI
3	10	Yes	No	No	H1	H2

© 2021 Howell RS et al. *JAMA Network Open.*

Wound EMR Information								
Sequential Number	Gender	Age	Wound Type	Wound Location	Wound Length (cm)	Wound Width (cm)	Wound Depth (cm)	Wound Area (cm^2)
93	M	54	Surgical	RLE lat	8.5	4.0	0.2	34.00

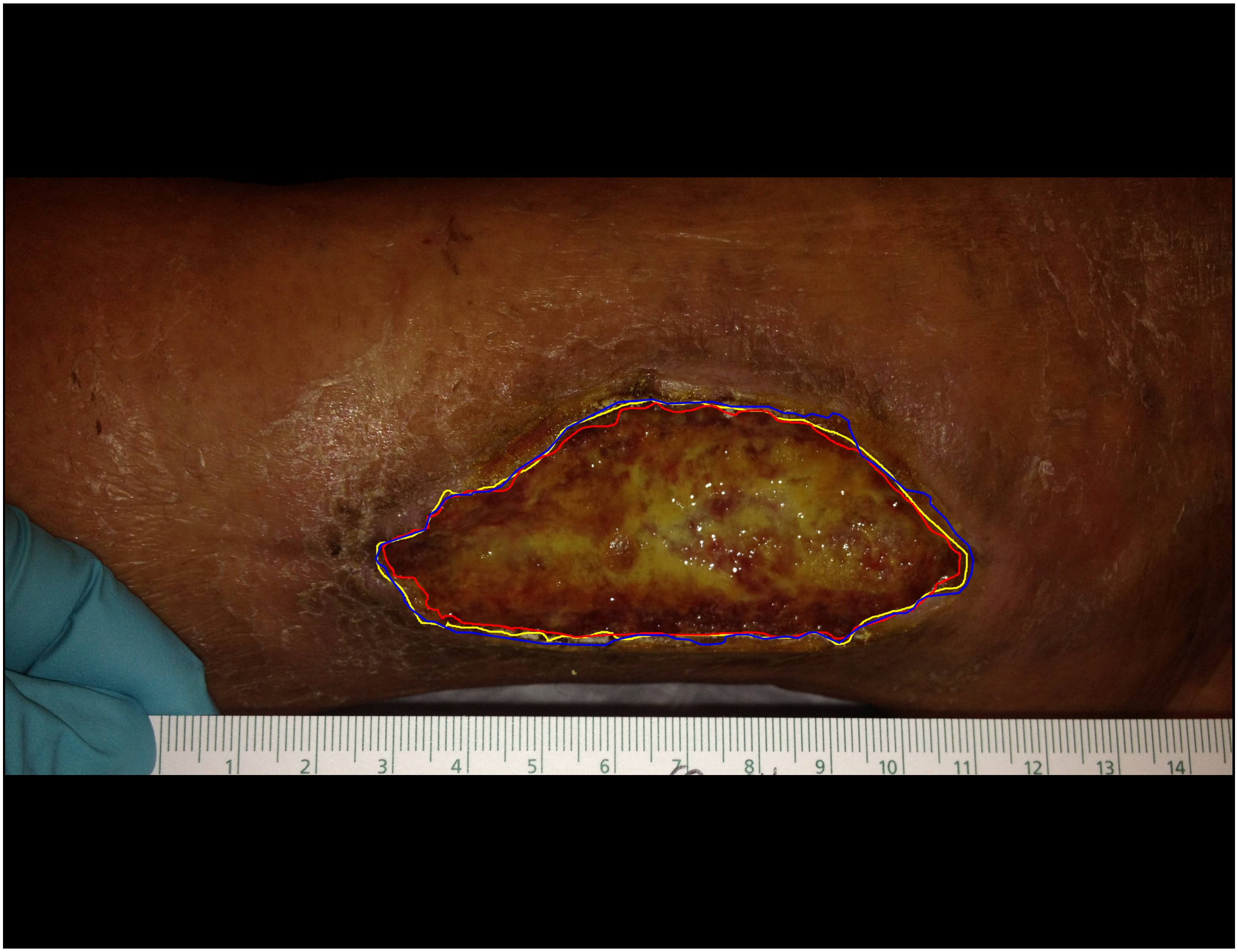

Tracing Data			
Tracer:	Wound Area (px^2)	Ruler Calibration (px/cm)	Wound Area (cm^2)
H1	707044	196.0	18.40
H2	669969	190.0	18.56
AI	735133	197.7	18.80

Tracing Comparisons				
Difference Metric:	Human-Human		Human-AI	
	H1(ref) H2(test)	H2(ref) H1(test)	H1(ref) AI(test)	H2(ref) AI(test)
False Negative Area (%)	5.8	0.6	0.7	0.2
False Positive Area (%)	0.6	6.2	4.7	10.0
|Relative Error (%)|	5.2	5.5	4.0	9.7

Blinded Attending Surgeon Review						
Reviewer	PGT Estimate	H1 meets definition?	H2 meets definition?	AI meets definition?	Which is AI?	Which is most accurate?
1	0	No	Yes	Yes	AI	AI
2	0	Yes	Yes	No	AI	H1
3	40	Yes	Yes	Yes	H1	AI

© 2021 Howell RS et al. *JAMA Network Open.*

Wound EMR Information								
Sequential Number	Gender	Age	Wound Type	Wound Location	Wound Length (cm)	Wound Width (cm)	Wound Depth (cm)	Wound Area (cm^2)
94	M	76	VLU	RLE med	5.7	3.7	0.1	21.09

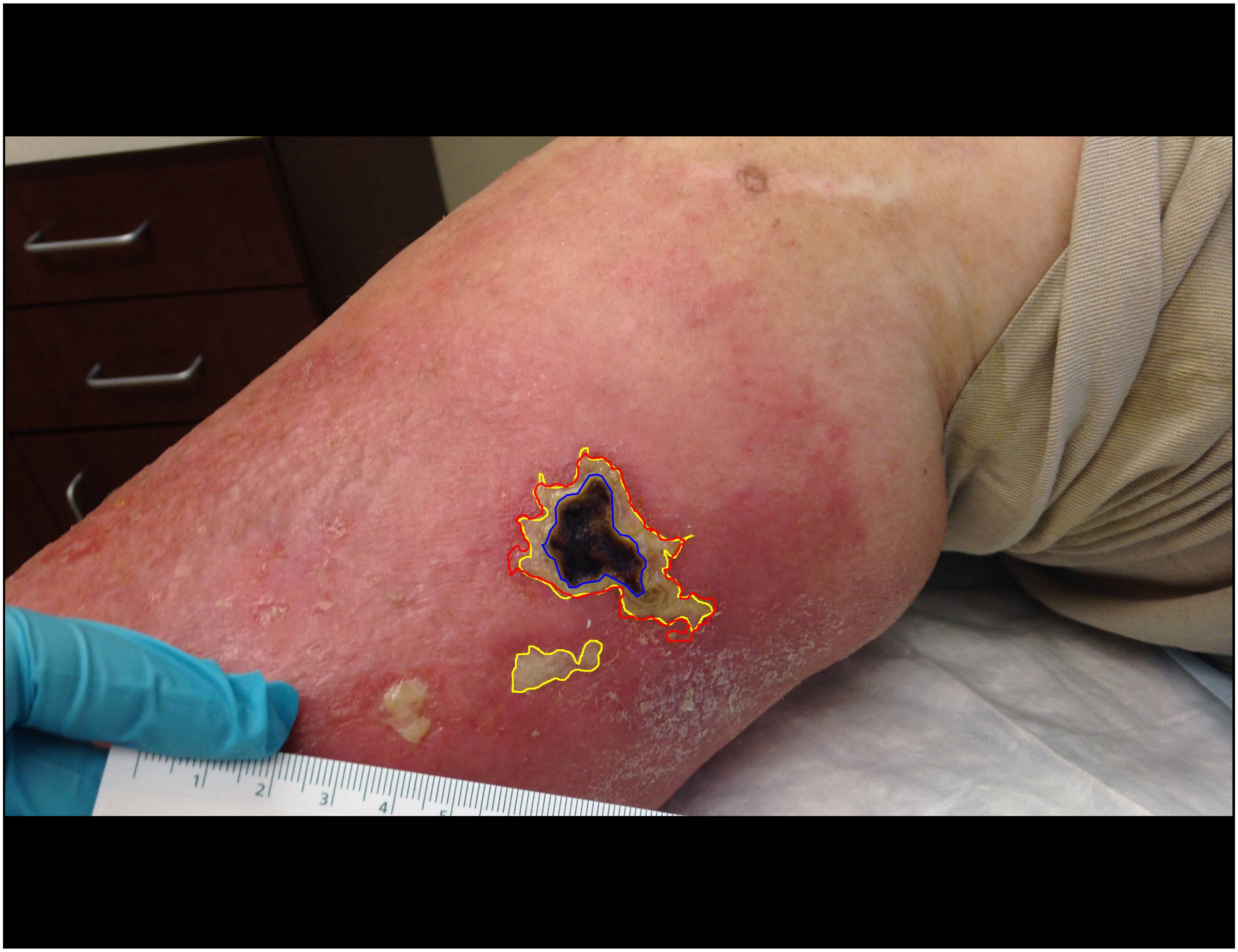

Tracing Data			
Tracer:	Wound Area (px^2)	Ruler Calibration (px/cm)	Wound Area (cm^2)
H1	133664	160.5	5.19
H2	124146	146.8	5.76
AI	49808	166.3	1.80

Tracing Comparisons				
Difference Metric:	Human-Human		Human-AI	
	H1(ref) H2(test)	H2(ref) H1(test)	H1(ref) AI(test)	H2(ref) AI(test)
False Negative Area (%)	14.9	8.3	62.7	59.9
False Positive Area (%)	7.7	16.0	0.0	0.0
|Relative Error (%)|	7.1	7.7	62.7	59.9

Blinded Attending Surgeon Review						
Reviewer	PGT Estimate	H1 meets definition?	H2 meets definition?	AI meets definition?	Which is AI?	Which is most accurate?
1	0	No	No	No	H1	H2
2	0	No	No	No	H1	AI
3	0	No	No	Yes	AI	AI

© 2021 Howell RS et al. *JAMA Network Open.*

Wound EMR Information								
Sequential Number	Gender	Age	Wound Type	Wound Location	Wound Length (cm)	Wound Width (cm)	Wound Depth (cm)	Wound Area (cm^2)
95	F	87	VLU	RLE med	2.0	1.5	0.2	3.00

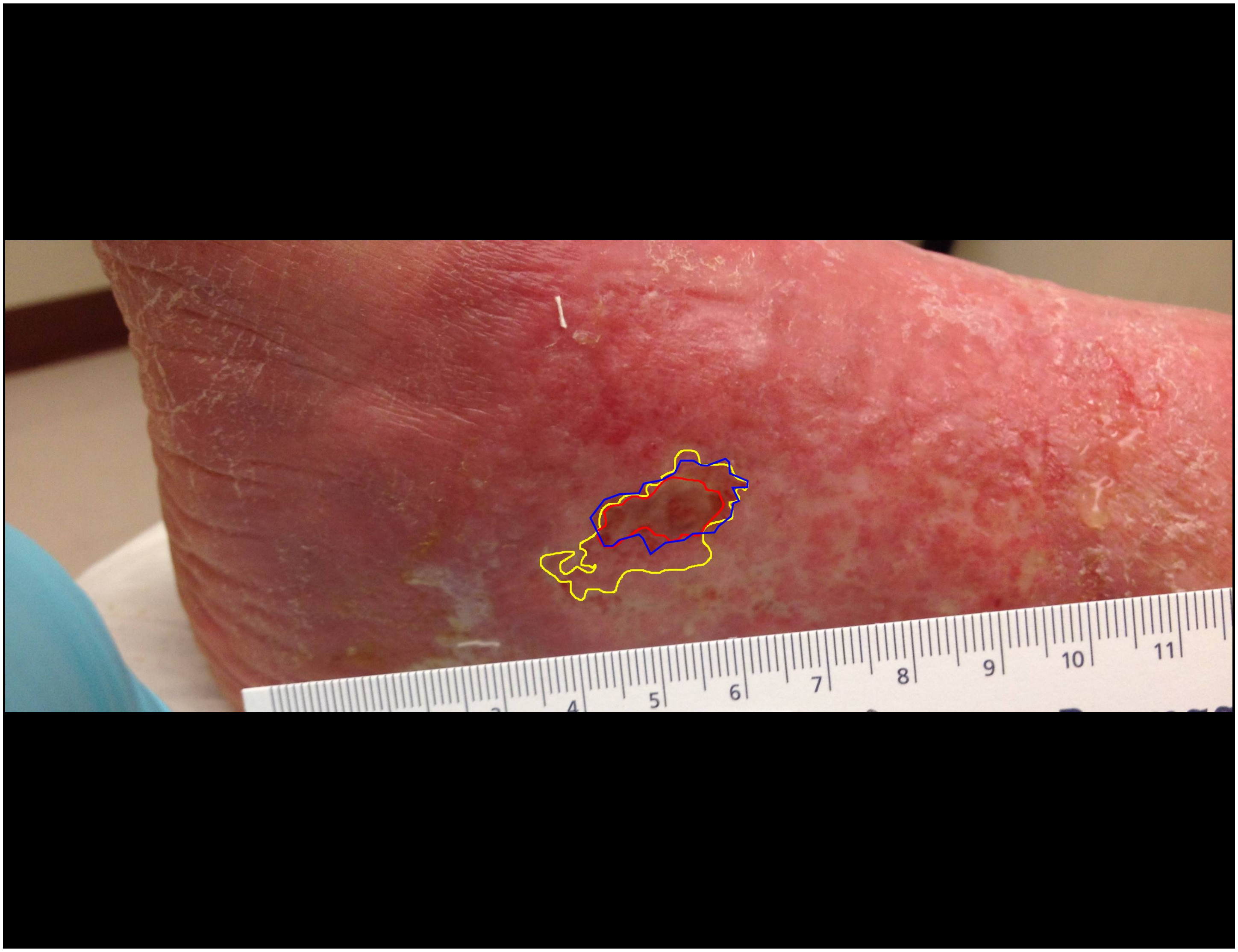

Tracing Data			
Tracer:	Wound Area (px^2)	Ruler Calibration (px/cm)	Wound Area (cm^2)
H1	94007	219.0	1.96
H2	36257	214.1	0.79
AI	58903	212.9	1.30

Tracing Comparisons				
Difference Metric:	Human-Human		Human-AI	
	H1(ref) H2(test)	H2(ref) H1(test)	H1(ref) AI(test)	H2(ref) AI(test)
False Negative Area (%)	61.4	0.0	42.4	1.3
False Positive Area (%)	0.0	159.3	5.0	63.8
|Relative Error (%)|	61.4	159.3	37.3	62.5

Blinded Attending Surgeon Review						
Reviewer	PGT Estimate	H1 meets definition?	H2 meets definition?	AI meets definition?	Which is AI?	Which is most accurate?
1	0	No	Yes	No	H1	H1
2		No	No	No	H2	H1
3	0	No	Yes	Yes	AI	H1

© 2021 Howell RS et al. *JAMA Network Open.*

Wound EMR Information								
Sequential Number	Gender	Age	Wound Type	Wound Location	Wound Length (cm)	Wound Width (cm)	Wound Depth (cm)	Wound Area (cm^2)
96	M	53	PU	L ischium	3.0	1.2	0.1	3.60

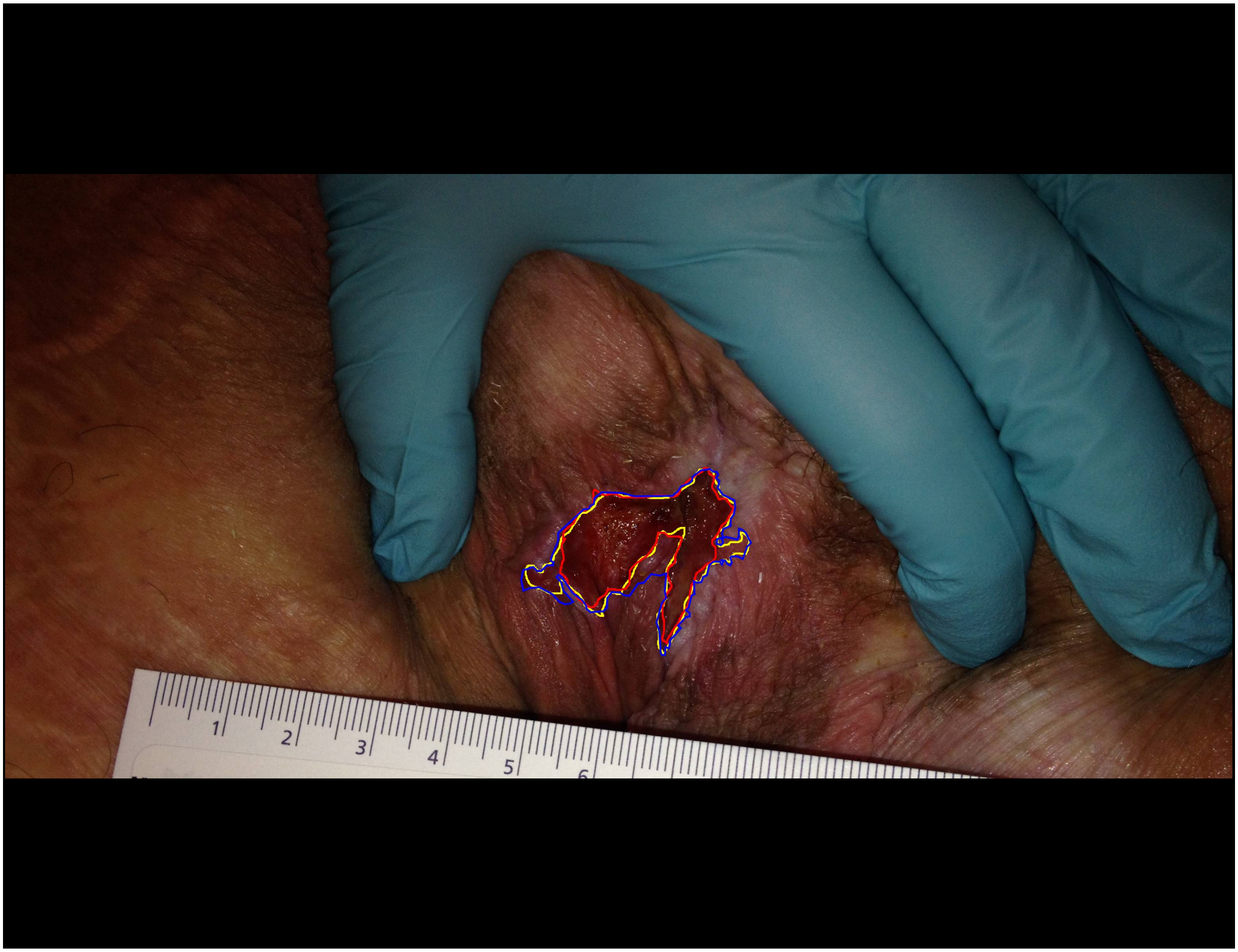

Tracing Data			
Tracer:	Wound Area (px^2)	Ruler Calibration (px/cm)	Wound Area (cm^2)
H1	111742	196.3	2.90
H2	96309	200.4	2.40
AI	131036	196.3	3.40

Tracing Comparisons				
Difference Metric:	Human-Human		Human-AI	
	H1(ref) H2(test)	H2(ref) H1(test)	H1(ref) AI(test)	H2(ref) AI(test)
False Negative Area (%)	17.1	3.8	1.2	0.7
False Positive Area (%)	3.3	19.8	18.4	36.8
|Relative Error (%)|	13.8	16.0	17.3	36.1

Blinded Attending Surgeon Review						
Reviewer	PGT Estimate	H1 meets definition?	H2 meets definition?	AI meets definition?	Which is AI?	Which is most accurate?
1	50	No	Yes	No	H2	AI
2	<10	Yes	Yes	No	H1	H1
3	60	No	Yes	No	H2	AI

© 2021 Howell RS et al. *JAMA Network Open.*

Wound EMR Information								
Sequential Number	Gender	Age	Wound Type	Wound Location	Wound Length (cm)	Wound Width (cm)	Wound Depth (cm)	Wound Area (cm^2)
97	M	71	VLU	RLE lat	10.0	7.0	0.1	70.00

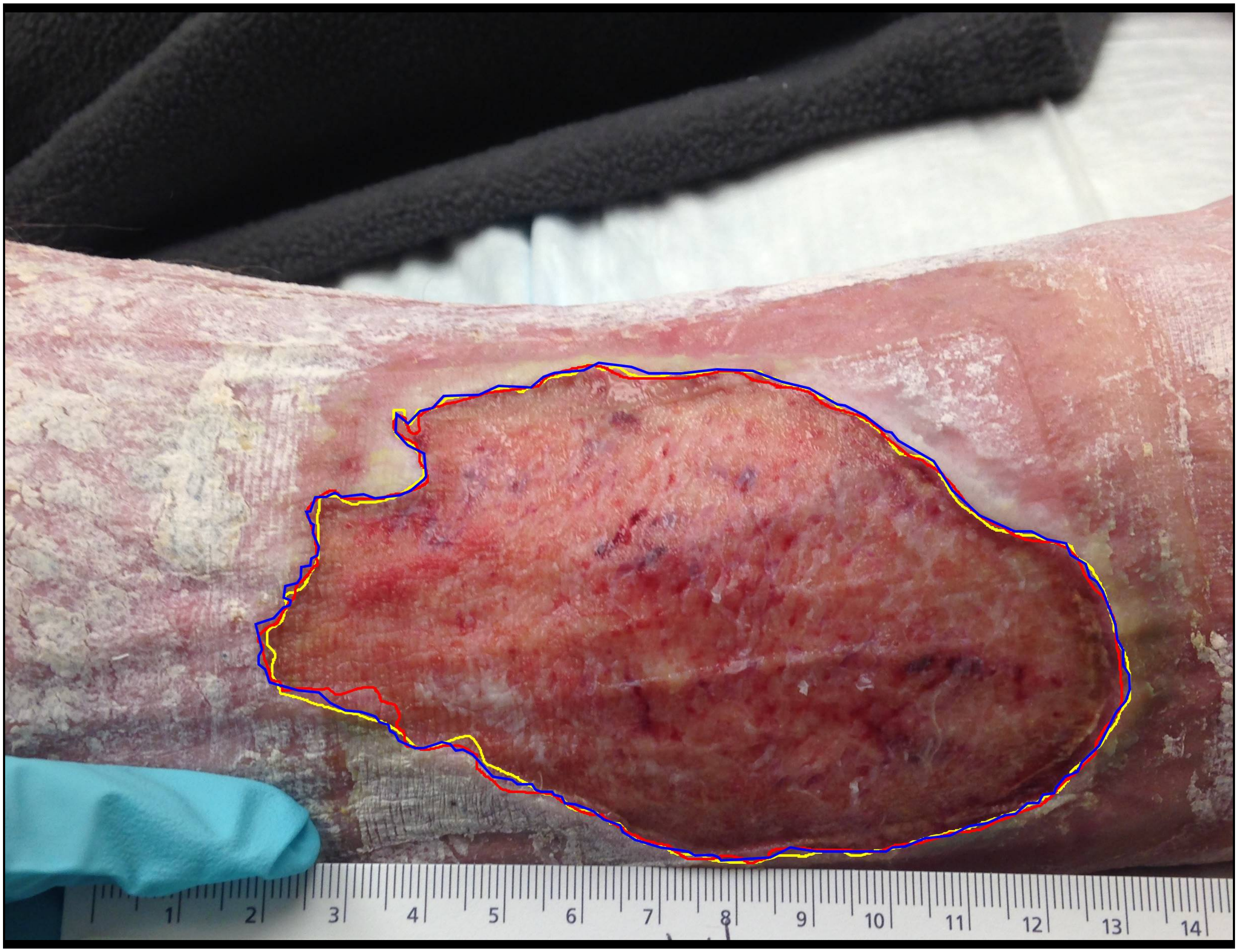

Tracing Data			
Tracer:	Wound Area (px^2)	Ruler Calibration (px/cm)	Wound Area (cm^2)
H1	1184619	154.0	49.92
H2	1176017	154.1	49.55
AI	1194677	160.5	46.40

Tracing Comparisons				
Difference Metric:	Human-Human		Human-AI	
	H1(ref) H2(test)	H2(ref) H1(test)	H1(ref) AI(test)	H2(ref) AI(test)
False Negative Area (%)	2.0	1.3	0.7	0.7
False Positive Area (%)	1.3	2.0	1.6	2.3
|Relative Error (%)|	0.7	0.7	0.8	1.6

Blinded Attending Surgeon Review						
Reviewer	PGT Estimate	H1 meets definition?	H2 meets definition?	AI meets definition?	Which is AI?	Which is most accurate?
1	0	Yes	Yes	Yes	AI	H2
2		Yes	Yes	Yes	H1	H2
3	100	Yes	Yes	Yes	H1	H1

© 2021 Howell RS et al. *JAMA Network Open.*

Wound EMR Information								
Sequential Number	Gender	Age	Wound Type	Wound Location	Wound Length (cm)	Wound Width (cm)	Wound Depth (cm)	Wound Area (cm^2)
98	M	64	VLU	L ankle	1.3	0.9	0.2	1.17

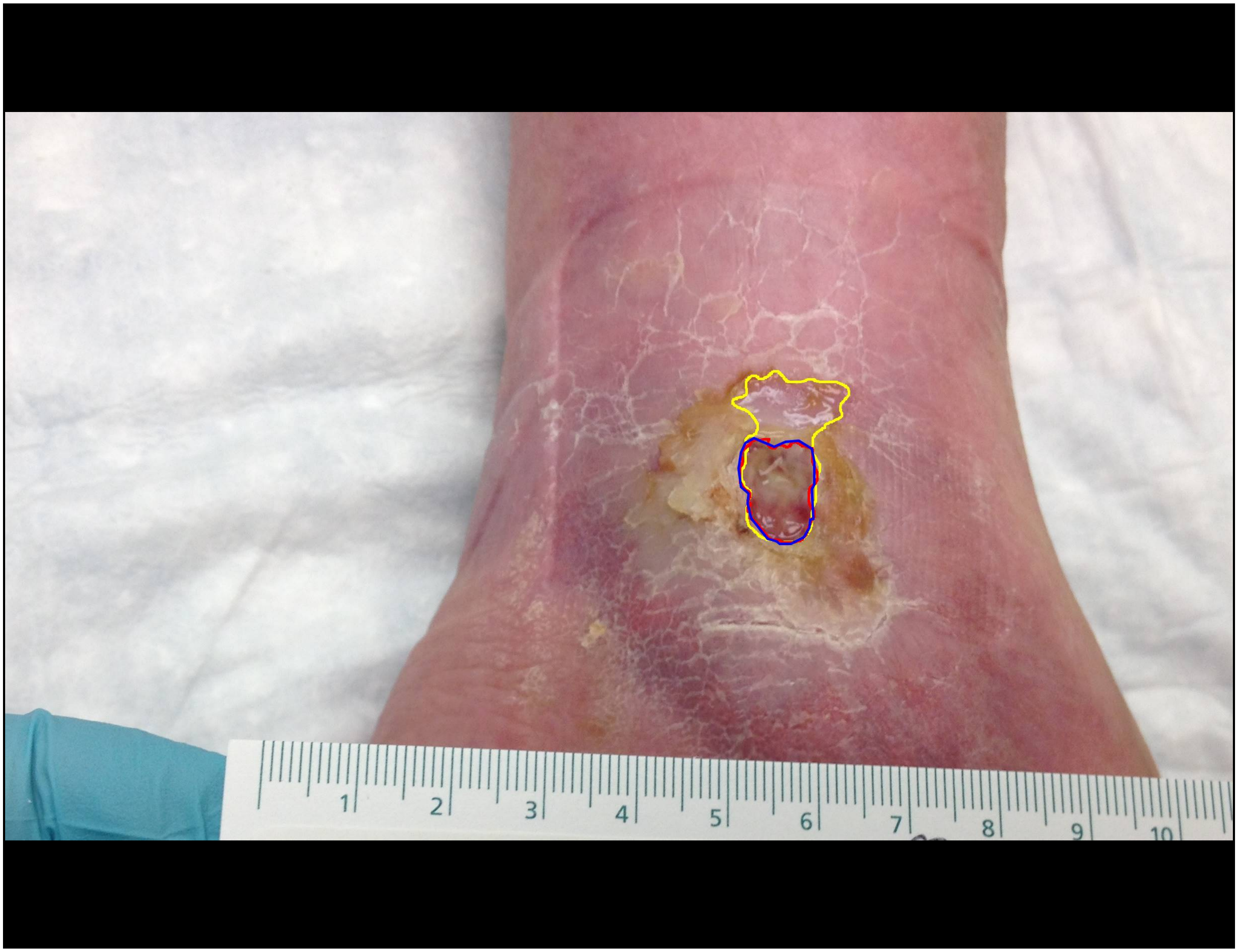

Tracing Data			
Tracer:	Wound Area (px^2)	Ruler Calibration (px/cm)	Wound Area (cm^2)
H1	47790	180.3	1.47
H2	24160	180.8	0.74
AI	25150	189.5	0.70

Tracing Comparisons				
Difference Metric:	Human-Human		Human-AI	
	H1(ref) H2(test)	H2(ref) H1(test)	H1(ref) AI(test)	H2(ref) AI(test)
False Negative Area (%)	50.8	2.7	49.4	1.6
False Positive Area (%)	1.3	100.5	2.0	5.7
|Relative Error (%)|	49.4	97.8	47.4	4.1

Blinded Attending Surgeon Review						
Reviewer	PGT Estimate	H1 meets definition?	H2 meets definition?	AI meets definition?	Which is AI?	Which is most accurate?
1	20	No	Yes	Yes	H1	H1
2	0	Yes	No	No	H1	H2
3	10	No	Yes	Yes	AI	H1

© 2021 Howell RS et al. *JAMA Network Open.*

Wound EMR Information								
Sequential Number	Gender	Age	Wound Type	Wound Location	Wound Length (cm)	Wound Width (cm)	Wound Depth (cm)	Wound Area (cm^2)
99	F	81	VLU	R ankle	0.5	0.2	0.2	0.10

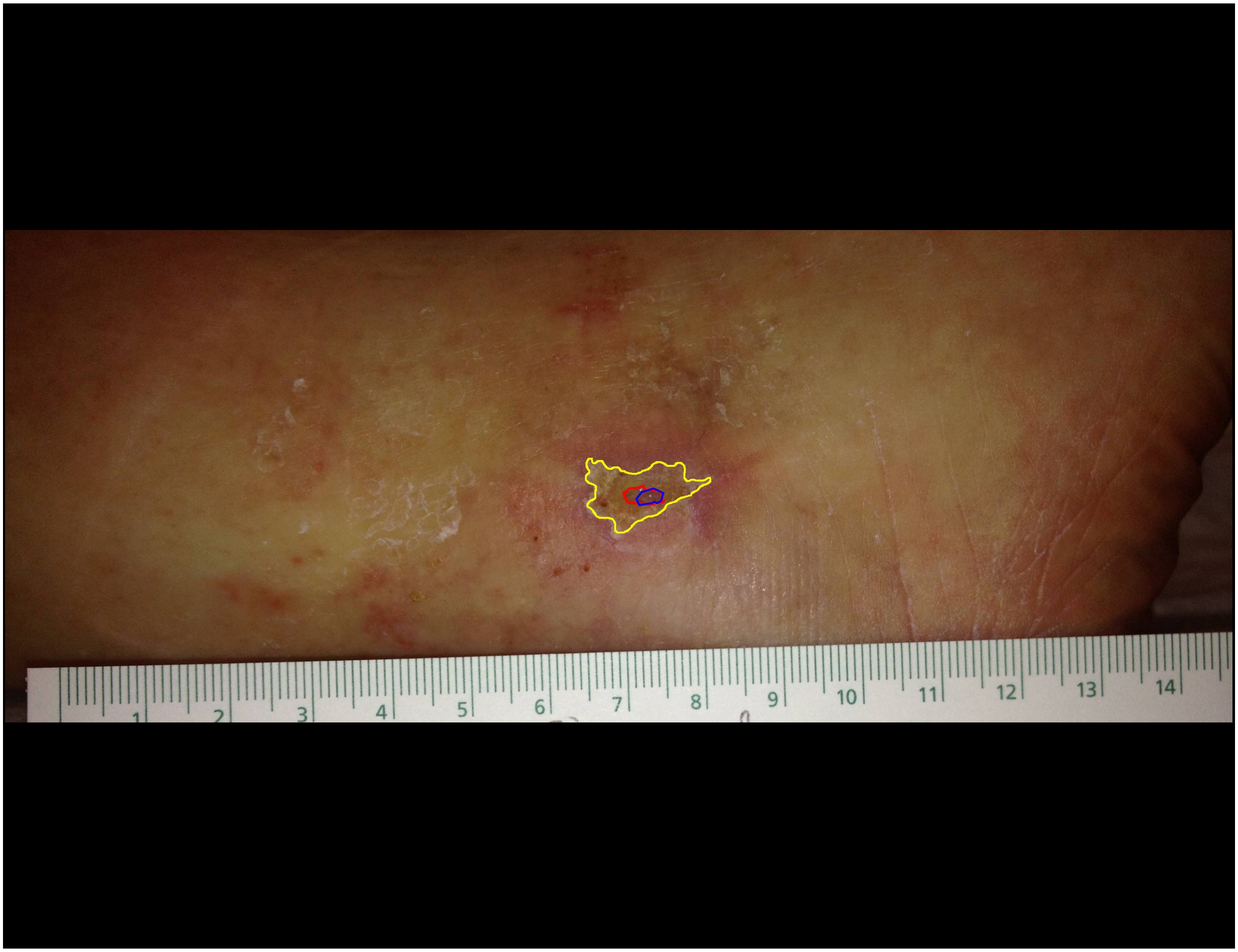

Tracing Data			
Tracer:	Wound Area (px^2)	Ruler Calibration (px/cm)	Wound Area (cm^2)
H1	34720	208.3	0.80
H2	3708	208.9	0.09
AI	2138	208.9	0.05

Tracing Comparisons				
Difference Metric:	Human-Human		Human-AI	
	H1(ref) H2(test)	H2(ref) H1(test)	H1(ref) AI(test)	H2(ref) AI(test)
False Negative Area (%)	89.3	0.0	93.9	46.3
False Positive Area (%)	0.0	836.3	0.0	3.9
|Relative Error (%)|	89.3	836.4	93.8	42.3

Blinded Attending Surgeon Review						
Reviewer	PGT Estimate	H1 meets definition?	H2 meets definition?	AI meets definition?	Which is AI?	Which is most accurate?
1	0	No	Yes	No	H1	H1
2	0	No	Yes	Yes	H2	AI
3	0	No	Yes	Yes	AI	H1

© 2021 Howell RS et al. *JAMA Network Open.*

Wound EMR Information								
Sequential Number	Gender	Age	Wound Type	Wound Location	Wound Length (cm)	Wound Width (cm)	Wound Depth (cm)	Wound Area (cm^2)
100	M	81	VLU	LLE ant	1.1	0.4	0.1	0.44

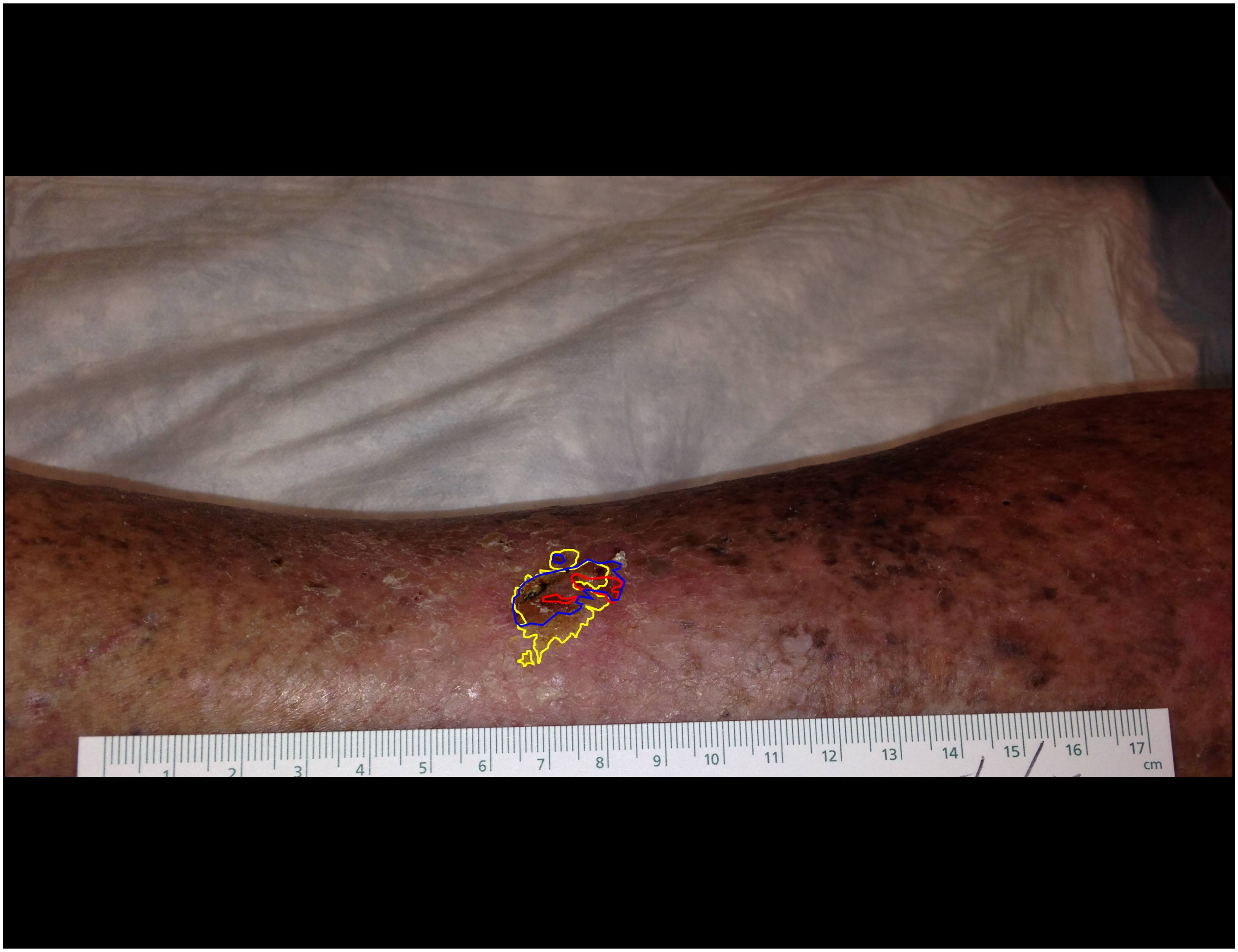

Tracing Data			
Tracer:	Wound Area (px^2)	Ruler Calibration (px/cm)	Wound Area (cm^2)
H1	41459	157.1	1.68
H2	6138	154.8	0.26
AI	29987	165.1	1.10

Tracing Comparisons				
Difference Metric:	Human-Human		Human-AI	
	H1(ref) H2(test)	H2(ref) H1(test)	H1(ref) AI(test)	H2(ref) AI(test)
False Negative Area (%)	92.8	51.2	41.9	6.6
False Positive Area (%)	7.6	626.6	14.2	395.2
|Relative Error (%)|	85.2	575.4	27.7	388.5

Blinded Attending Surgeon Review						
Reviewer	PGT Estimate	H1 meets definition?	H2 meets definition?	AI meets definition?	Which is AI?	Which is most accurate?
1	0	No	No	No	H2	None
2	0	No	No	Yes	H2	AI
3	0	No	Yes	No	AI	H1

© 2021 Howell RS et al. *JAMA Network Open.*

Wound EMR Information								
Sequential Number	Gender	Age	Wound Type	Wound Location	Wound Length (cm)	Wound Width (cm)	Wound Depth (cm)	Wound Area (cm^2)
101	F	94	Trauma	RLE ant	3.0	4.0	0.1	12.00

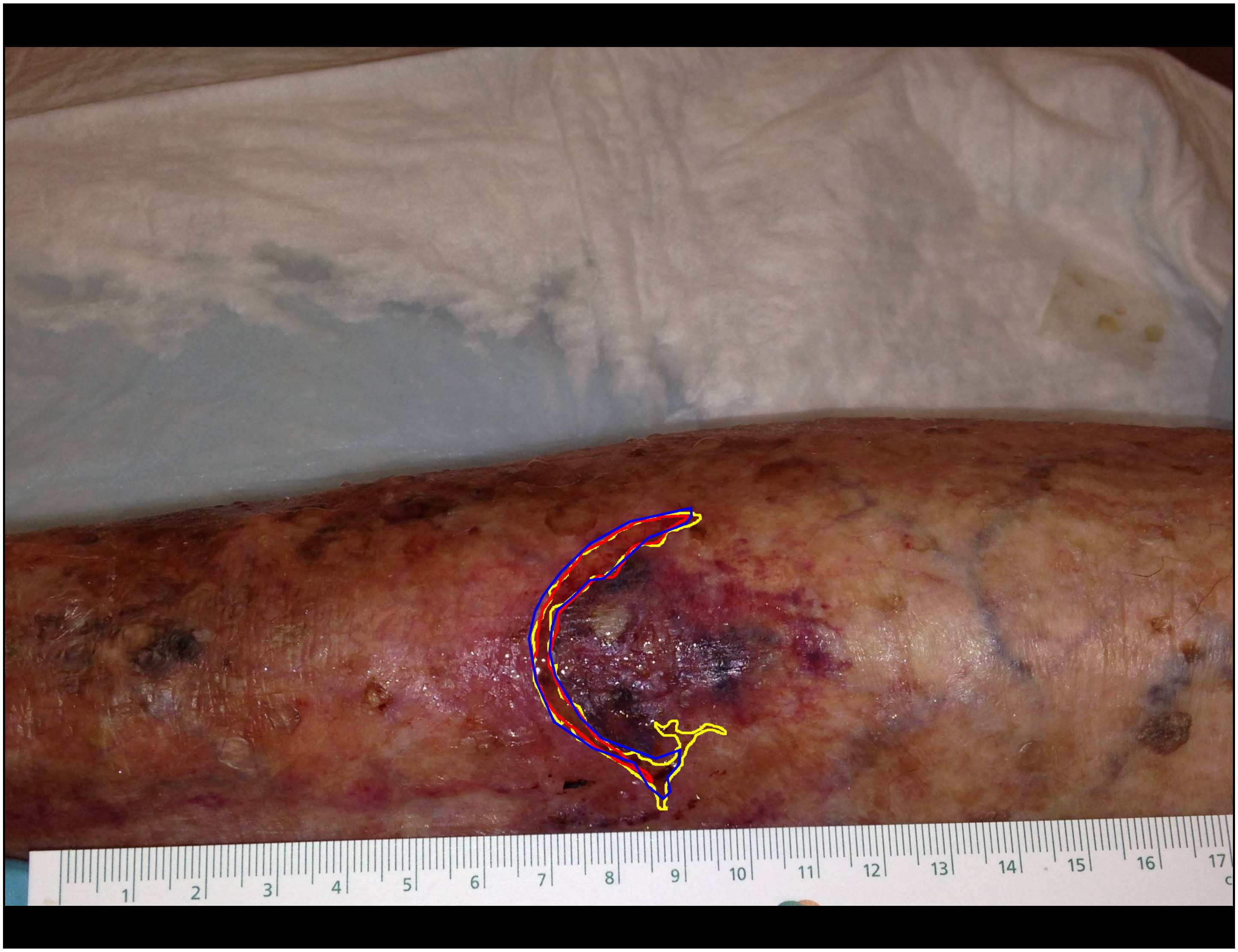

Tracing Data			
Tracer:	Wound Area (px^2)	Ruler Calibration (px/cm)	Wound Area (cm^2)
H1	34422	132.2	1.97
H2	25337	132.1	1.45
AI	36285	142.0	1.80

Tracing Comparisons				
Difference Metric:	Human-Human		Human-AI	
	H1(ref) H2(test)	H2(ref) H1(test)	H1(ref) AI(test)	H2(ref) AI(test)
False Negative Area (%)	33.5	9.6	17.6	8.6
False Positive Area (%)	7.1	45.5	23.0	51.8
|Relative Error (%)|	26.4	35.9	5.4	43.2

Blinded Attending Surgeon Review						
Reviewer	PGT Estimate	H1 meets definition?	H2 meets definition?	AI meets definition?	Which is AI?	Which is most accurate?
1	20	No	No	No	H1	None
2	0	No	No	No	H1	H1
3	10	No	Yes	Yes	H2	AI

© 2021 Howell RS et al. *JAMA Network Open.*

Wound EMR Information								
Sequential Number	Gender	Age	Wound Type	Wound Location	Wound Length (cm)	Wound Width (cm)	Wound Depth (cm)	Wound Area (cm^2)
102	F	67	PU	R foot	5.9	2.8	1.0	16.52

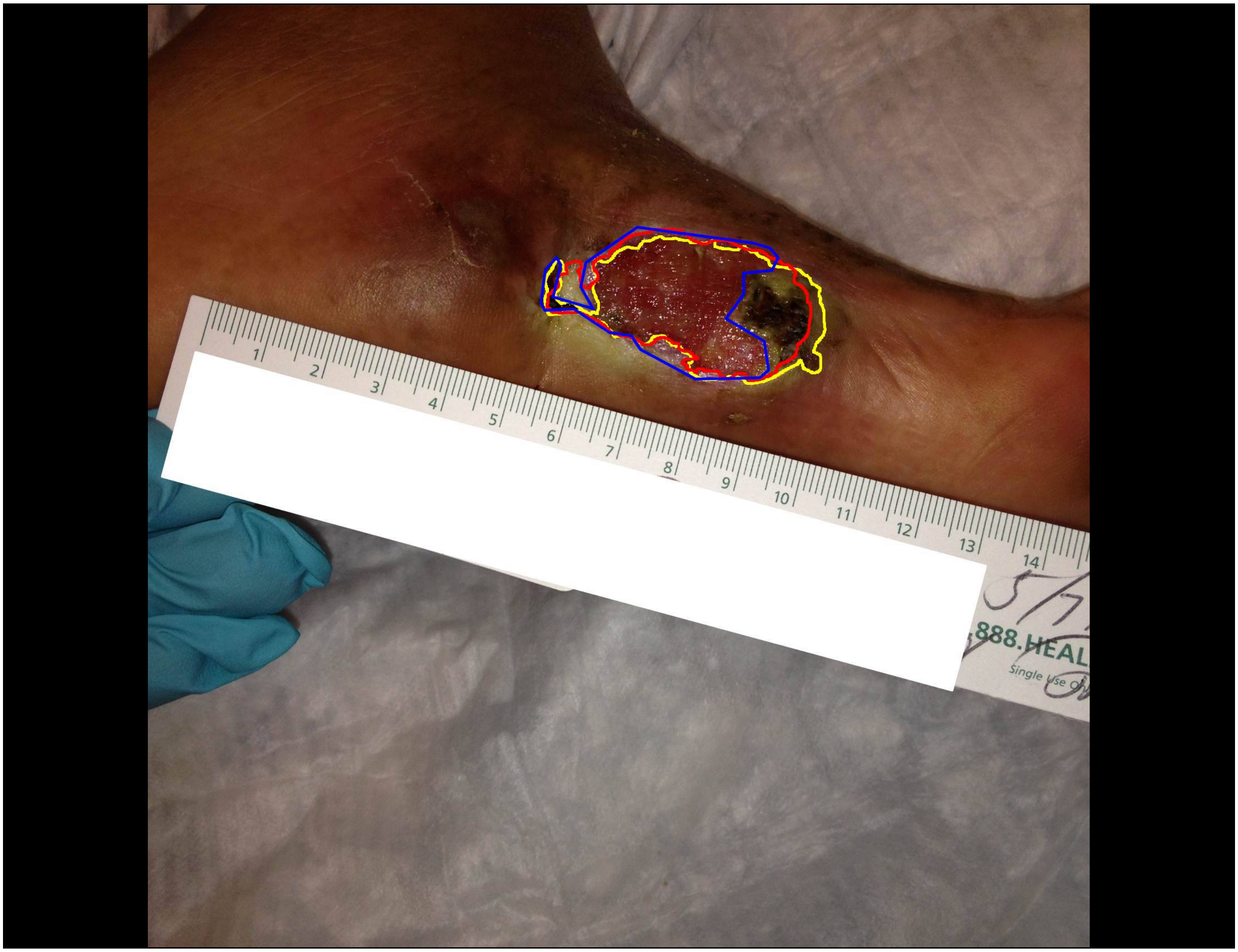

Tracing Data			
Tracer:	Wound Area (px^2)	Ruler Calibration (px/cm)	Wound Area (cm^2)
H1	81323	110.2	6.70
H2	80857	108.3	6.89
AI	69258	104.0	6.40

Tracing Comparisons				
Difference Metric:	Human-Human		Human-AI	
	H1(ref) H2(test)	H2(ref) H1(test)	H1(ref) AI(test)	H2(ref) AI(test)
False Negative Area (%)	10.6	10.1	29.6	26.1
False Positive Area (%)	10.0	10.7	14.8	11.7
|Relative Error (%)|	0.6	0.6	14.8	14.3

Blinded Attending Surgeon Review						
Reviewer	PGT Estimate	H1 meets definition?	H2 meets definition?	AI meets definition?	Which is AI?	Which is most accurate?
1	70	Yes	No	Yes	H2	H2
2	70	Yes	Yes	No	AI	H2
3	80	No	Yes	No	AI	H1

© 2021 Howell RS et al. *JAMA Network Open.*

Wound EMR Information								
Sequential Number	Gender	Age	Wound Type	Wound Location	Wound Length (cm)	Wound Width (cm)	Wound Depth (cm)	Wound Area (cm^2)
103	F	83	DFU	L foot dorsal	2.9	1.0	0.2	2.90

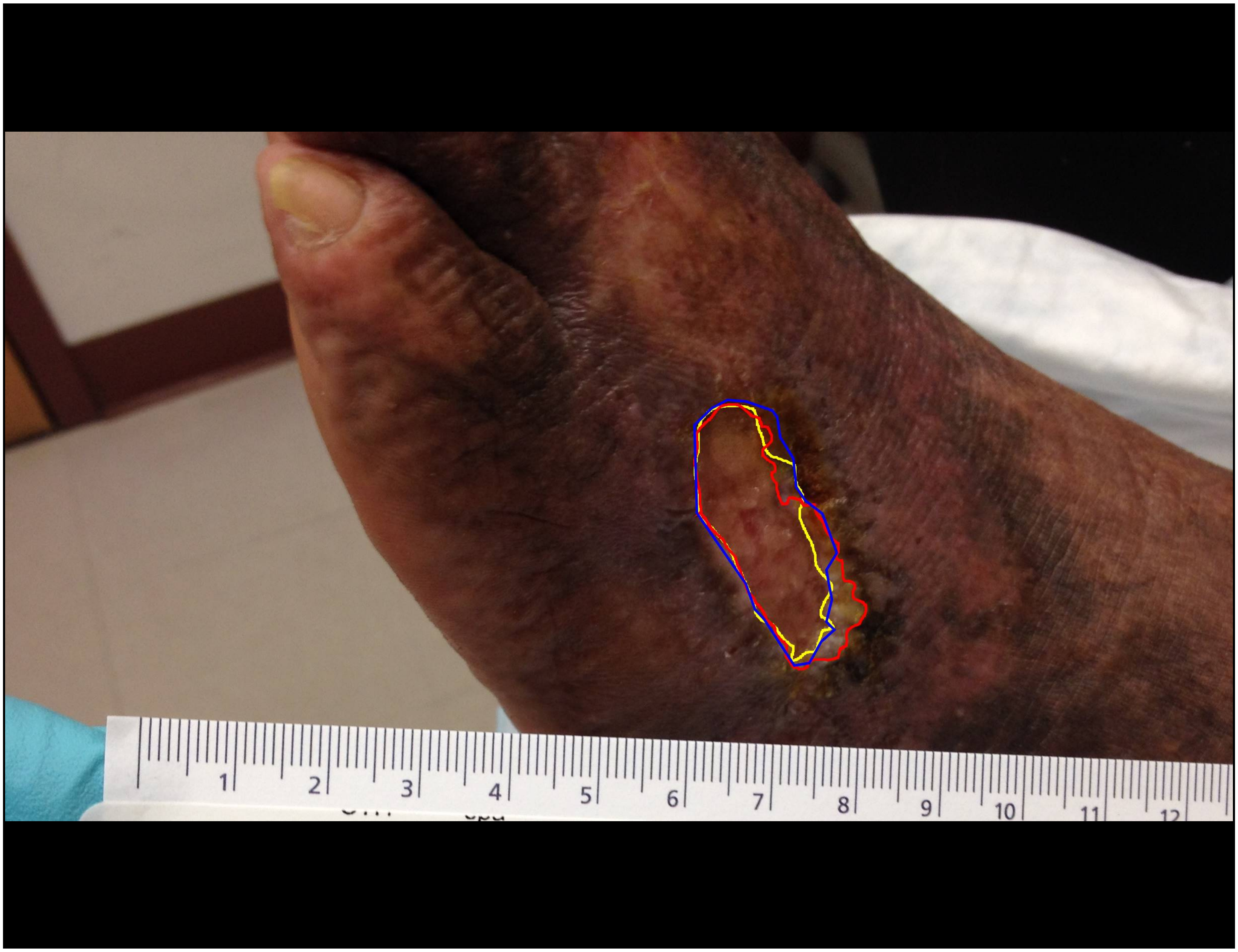

Tracing Data			
Tracer:	Wound Area (px^2)	Ruler Calibration (px/cm)	Wound Area (cm^2)
H1	74327	171.1	2.54
H2	87083	172.2	2.94
AI	89217	172.4	3.00

Tracing Comparisons				
Difference Metric:	Human-Human		Human-AI	
	H1(ref) H2(test)	H2(ref) H1(test)	H1(ref) AI(test)	H2(ref) AI(test)
False Negative Area (%)	7.3	20.9	0.5	11.3
False Positive Area (%)	24.5	6.2	20.6	13.7
|Relative Error (%)|	17.2	14.6	20.0	2.5

Blinded Attending Surgeon Review						
Reviewer	PGT Estimate	H1 meets definition?	H2 meets definition?	AI meets definition?	Which is AI?	Which is most accurate?
1	0	No	Yes	No	H1	AI
2	0	Yes	Yes	No	H1	H2
3	10	Yes	No	Yes	AI	H2

© 2021 Howell RS et al. *JAMA Network Open.*

Wound EMR Information								
Sequential Number	Gender	Age	Wound Type	Wound Location	Wound Length (cm)	Wound Width (cm)	Wound Depth (cm)	Wound Area (cm^2)
104	M	77	VLU	R ankle	2.0	2.8	0.2	5.60

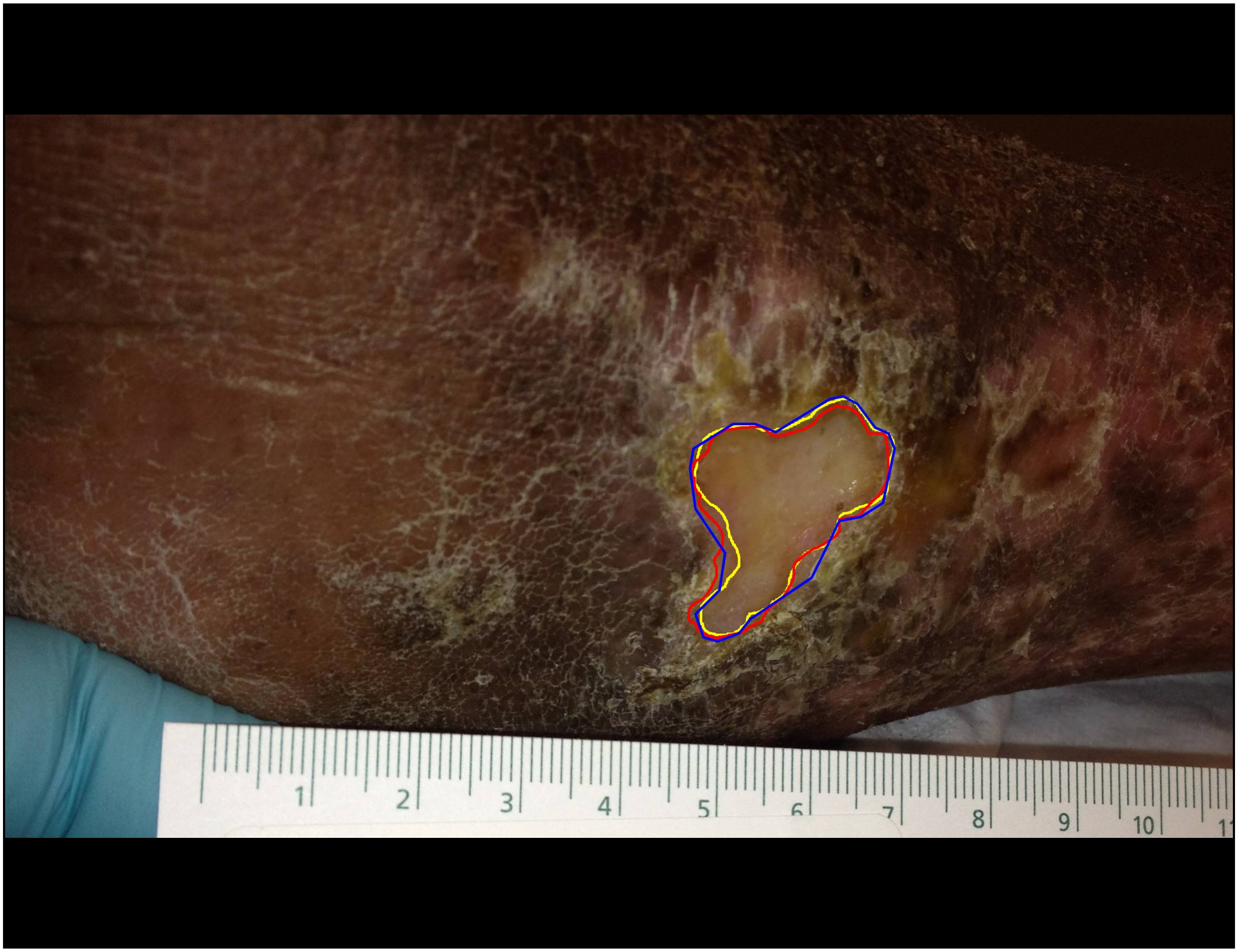

Tracing Data			
Tracer:	Wound Area (px^2)	Ruler Calibration (px/cm)	Wound Area (cm^2)
H1	101790	193.4	2.72
H2	105822	192.2	2.87
AI	115422	196.1	3.00

Tracing Comparisons				
Difference Metric:	Human-Human		Human-AI	
	H1(ref) H2(test)	H2(ref) H1(test)	H1(ref) AI(test)	H2(ref) AI(test)
False Negative Area (%)	5.4	9.0	0.5	3.2
False Positive Area (%)	9.4	5.2	13.9	12.3
|Relative Error (%)|	4.0	3.8	13.4	9.1

Blinded Attending Surgeon Review						
Reviewer	PGT Estimate	H1 meets definition?	H2 meets definition?	AI meets definition?	Which is AI?	Which is most accurate?
1	0	Yes	Yes	Yes	H1	H2
2	0	No	No	No	AI	H1
3	0	Yes	Yes	No	H2	H1

© 2021 Howell RS et al. *JAMA Network Open.*

Wound EMR Information								
Sequential Number	Gender	Age	Wound Type	Wound Location	Wound Length (cm)	Wound Width (cm)	Wound Depth (cm)	Wound Area (cm^2)
105	M	70	Arterial	R heel	1.3	2.0	0.1	2.60

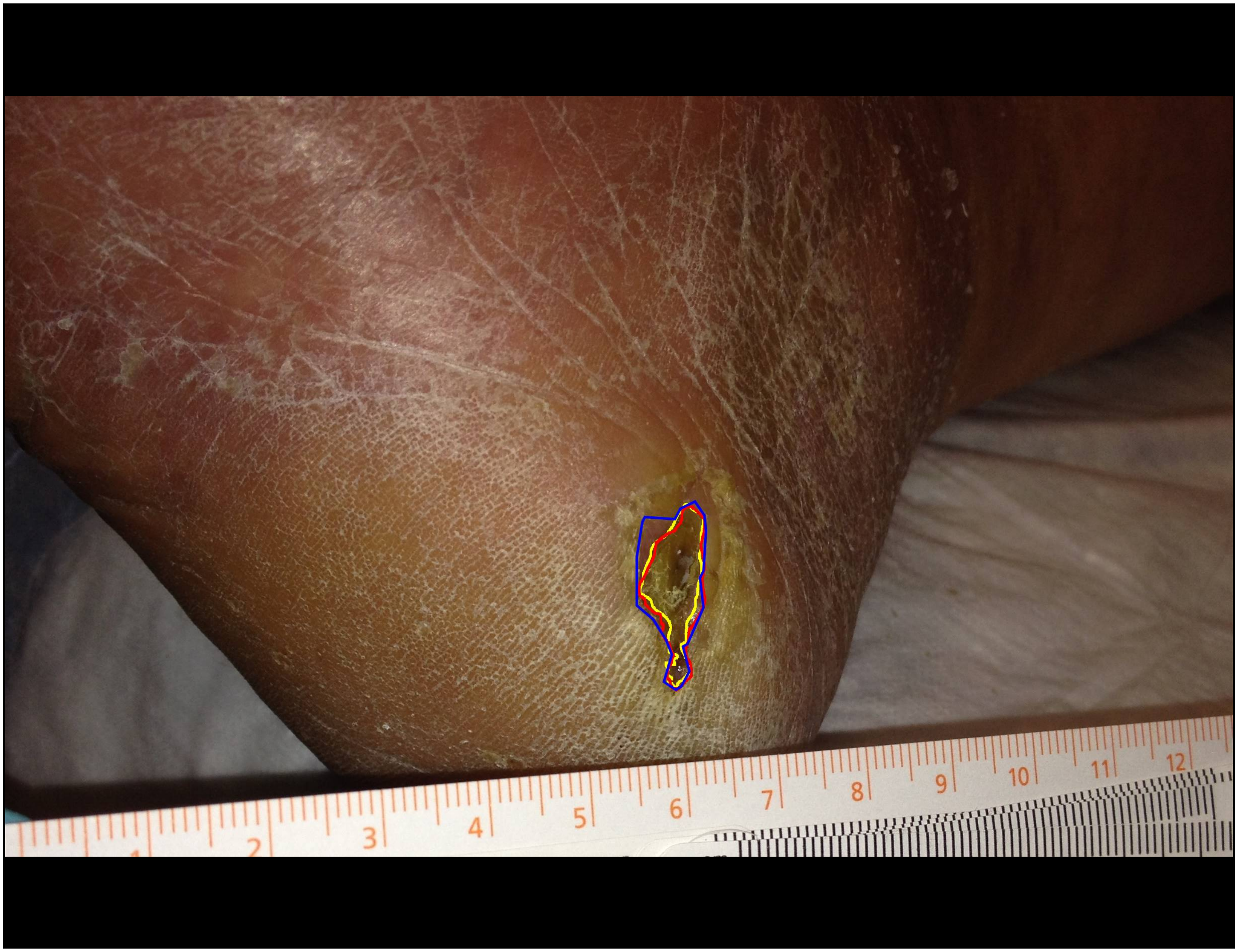

Tracing Data			
Tracer:	Wound Area (px^2)	Ruler Calibration (px/cm)	Wound Area (cm^2)
H1	23067	184.2	0.68
H2	26046	192.6	0.70
AI	33169	235.1	0.60

Tracing Comparisons				
Difference Metric:	Human-Human		Human-AI	
	H1(ref) H2(test)	H2(ref) H1(test)	H1(ref) AI(test)	H2(ref) AI(test)
False Negative Area (%)	4.4	15.3	0.6	1.8
False Positive Area (%)	17.3	3.9	44.4	29.2
|Relative Error (%)|	12.9	11.4	43.8	27.3

Blinded Attending Surgeon Review						
Reviewer	PGT Estimate	H1 meets definition?	H2 meets definition?	AI meets definition?	Which is AI?	Which is most accurate?
1	0	Yes	No	No	AI	AI
2	0	Yes	Yes	No	H1	AI
3	0	Yes	Yes	No	H2	AI

© 2021 Howell RS et al. *JAMA Network Open.*

Wound EMR Information								
Sequential Number	Gender	Age	Wound Type	Wound Location	Wound Length (cm)	Wound Width (cm)	Wound Depth (cm)	Wound Area (cm^2)
106	F	40	Surgical	R forearm	8.5	1.2	0.3	10.20

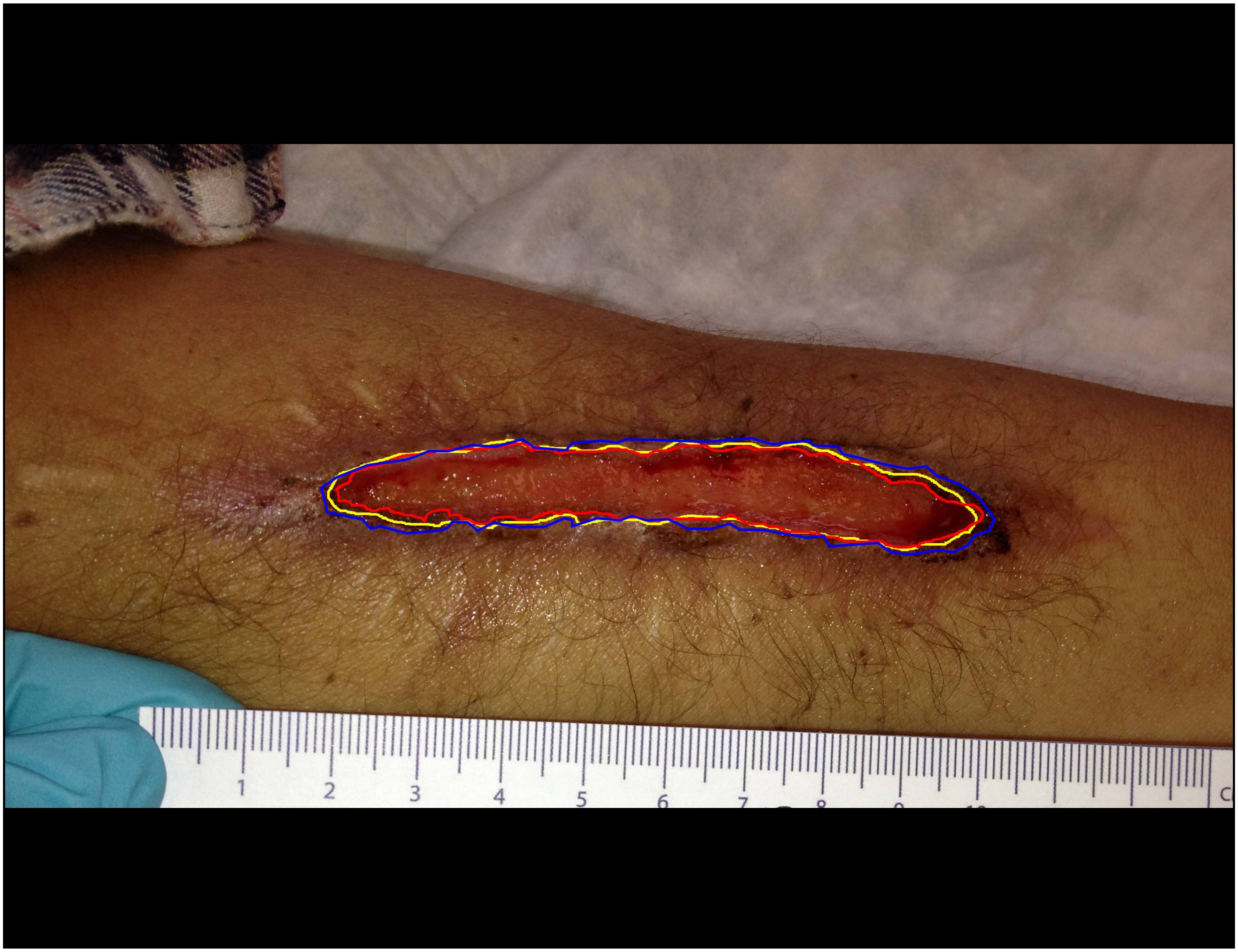

Tracing Data			
Tracer:	Wound Area (px^2)	Ruler Calibration (px/cm)	Wound Area (cm^2)
H1	180006	166.8	6.47
H2	161932	166.9	5.81
AI	205224	171.2	7.00

Tracing Comparisons				
Difference Metric:	Human-Human		Human-AI	
	H1(ref) H2(test)	H2(ref) H1(test)	H1(ref) AI(test)	H2(ref) AI(test)
False Negative Area (%)	11.0	1.0	1.4	0.3
False Positive Area (%)	0.9	12.2	15.4	27.1
|Relative Error (%)|	10.0	11.2	14.0	26.7

Blinded Attending Surgeon Review						
Reviewer	PGT Estimate	H1 meets definition?	H2 meets definition?	AI meets definition?	Which is AI?	Which is most accurate?
1	90	Yes	Yes	Yes	H2	H1
2	<10	No	Yes	No	H1	AI
3	100	Yes	Yes	Yes	H1	H2

© 2021 Howell RS et al. *JAMA Network Open.*

Wound EMR Information								
Sequential Number	Gender	Age	Wound Type	Wound Location	Wound Length (cm)	Wound Width (cm)	Wound Depth (cm)	Wound Area (cm^2)
107	F	30	Arterial	R hallux	1.5	1.4	0.3	2.10

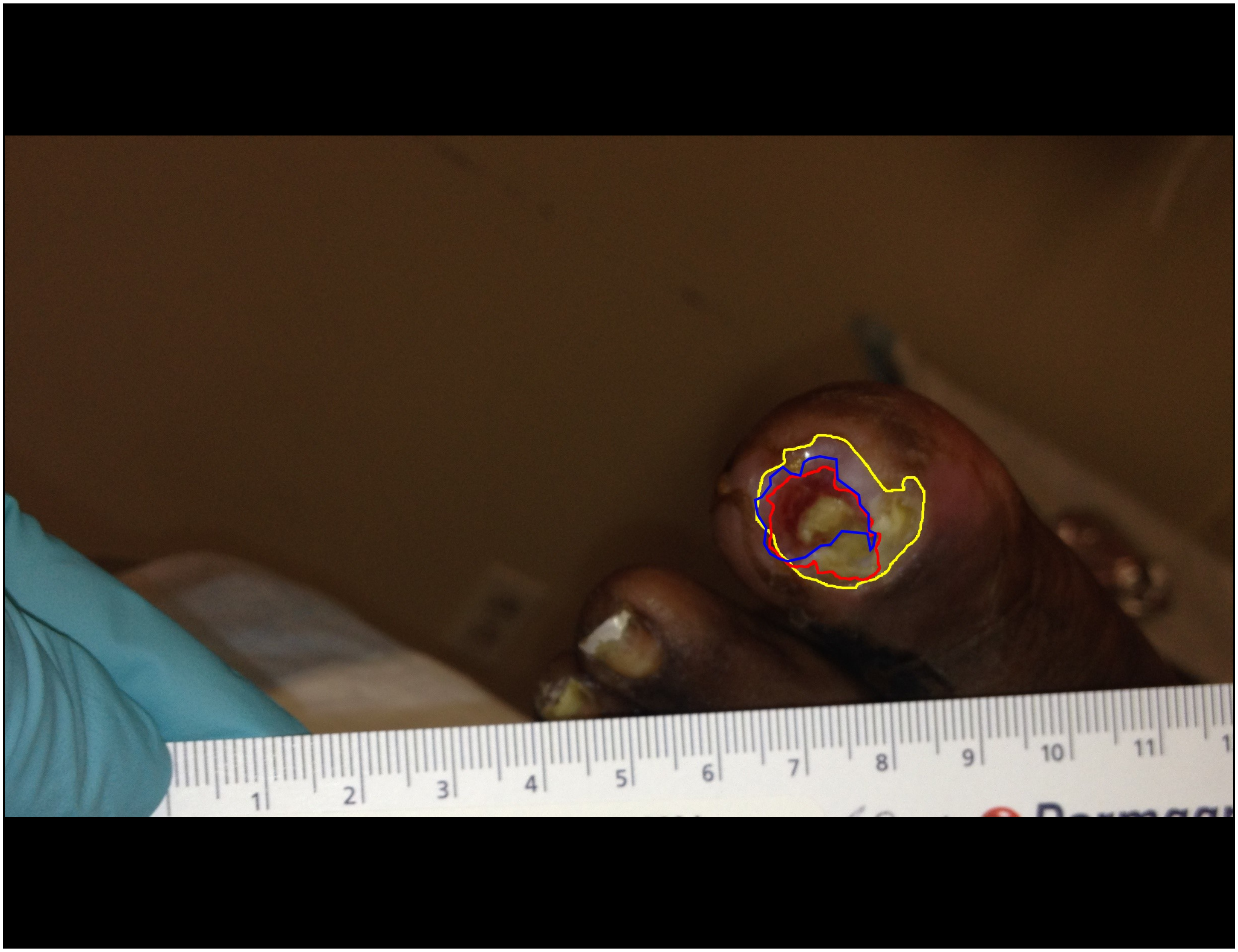

Tracing Data			
Tracer:	Wound Area (px^2)	Ruler Calibration (px/cm)	Wound Area (cm^2)
H1	68414	176.3	2.20
H2	35039	175.5	1.14
AI	30617	175.0	1.00

Tracing Comparisons				
Difference Metric:	Human-Human		Human-AI	
	H1(ref) H2(test)	H2(ref) H1(test)	H1(ref) AI(test)	H2(ref) AI(test)
False Negative Area (%)	49.4	1.3	56.8	29.5
False Positive Area (%)	0.7	96.5	1.6	16.9
|Relative Error (%)|	48.8	95.3	55.2	12.6

Blinded Attending Surgeon Review						
Reviewer	PGT Estimate	H1 meets definition?	H2 meets definition?	AI meets definition?	Which is AI?	Which is most accurate?
1	50	No	Yes	No	H2	H2
2	20	No	Yes	Yes	H1	H1
3	30	Yes	No	Yes	H1	H1

© 2021 Howell RS et al. *JAMA Network Open.*

Wound EMR Information								
Sequential Number	Gender	Age	Wound Type	Wound Location	Wound Length (cm)	Wound Width (cm)	Wound Depth (cm)	Wound Area (cm^2)
108	M	60	DFU	R plantar	0.6	0.5	0.1	0.30

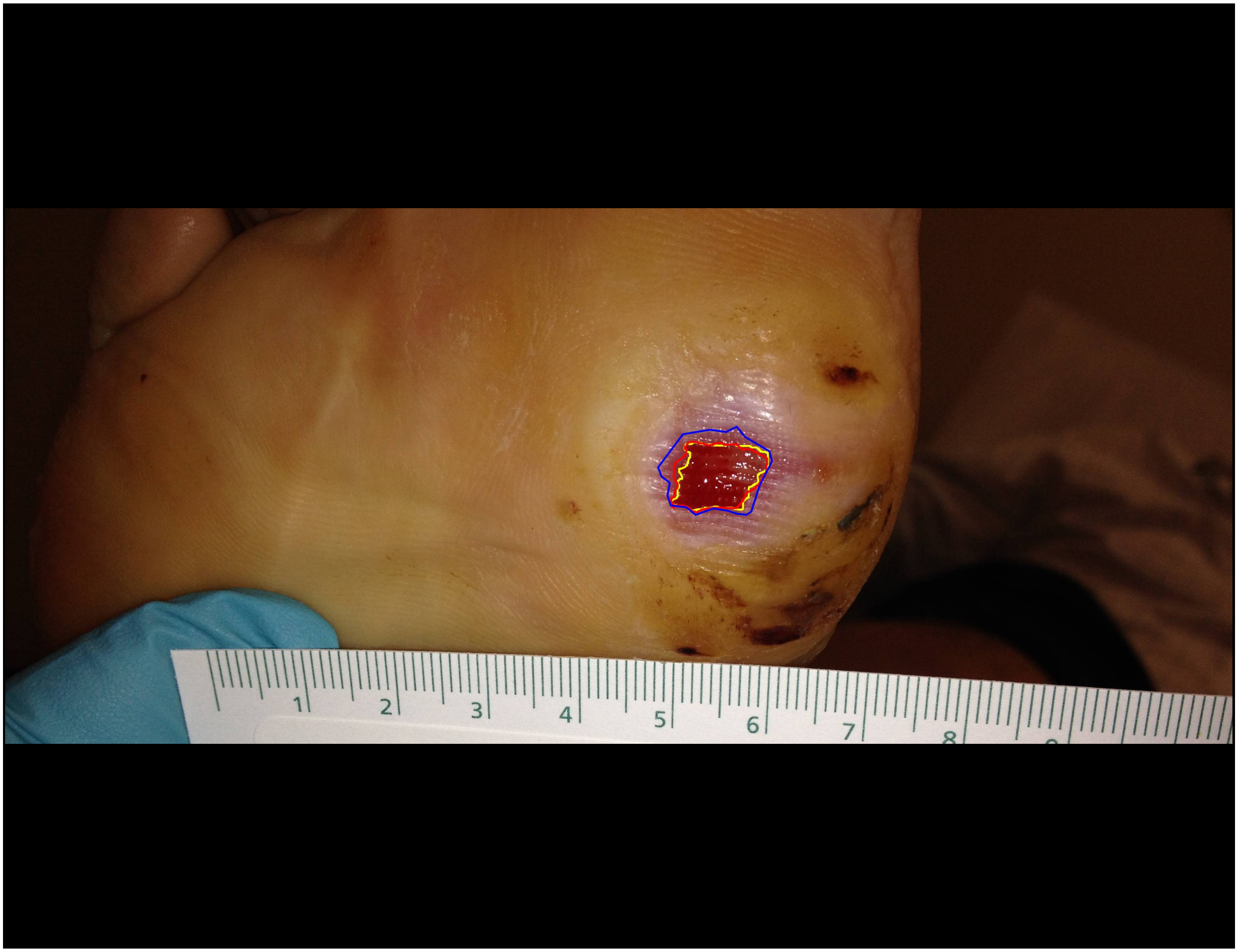

Tracing Data			
Tracer:	Wound Area (px^2)	Ruler Calibration (px/cm)	Wound Area (cm^2)
H1	33759	252.4	0.53
H2	35397	256.3	0.54
AI	51151	226.2	1.00

Tracing Comparisons				
Difference Metric:	Human-Human		Human-AI	
	H1(ref) H2(test)	H2(ref) H1(test)	H1(ref) AI(test)	H2(ref) AI(test)
False Negative Area (%)	4.1	8.5	0.0	0.0
False Positive Area (%)	9.0	3.9	51.5	44.6
|Relative Error (%)|	4.9	4.6	51.5	44.5

Blinded Attending Surgeon Review						
Reviewer	PGT Estimate	H1 meets definition?	H2 meets definition?	AI meets definition?	Which is AI?	Which is most accurate?
1	100	Yes	Yes	No	H2	H1
2	100	No	No	No	H2	H1
3	40	Yes	Yes	No	AI	H2

© 2021 Howell RS et al. *JAMA Network Open.*

Wound EMR Information								
Sequential Number	Gender	Age	Wound Type	Wound Location	Wound Length (cm)	Wound Width (cm)	Wound Depth (cm)	Wound Area (cm^2)
109	F	59	VLU	LLE lat	1.2	0.9	0.3	1.08

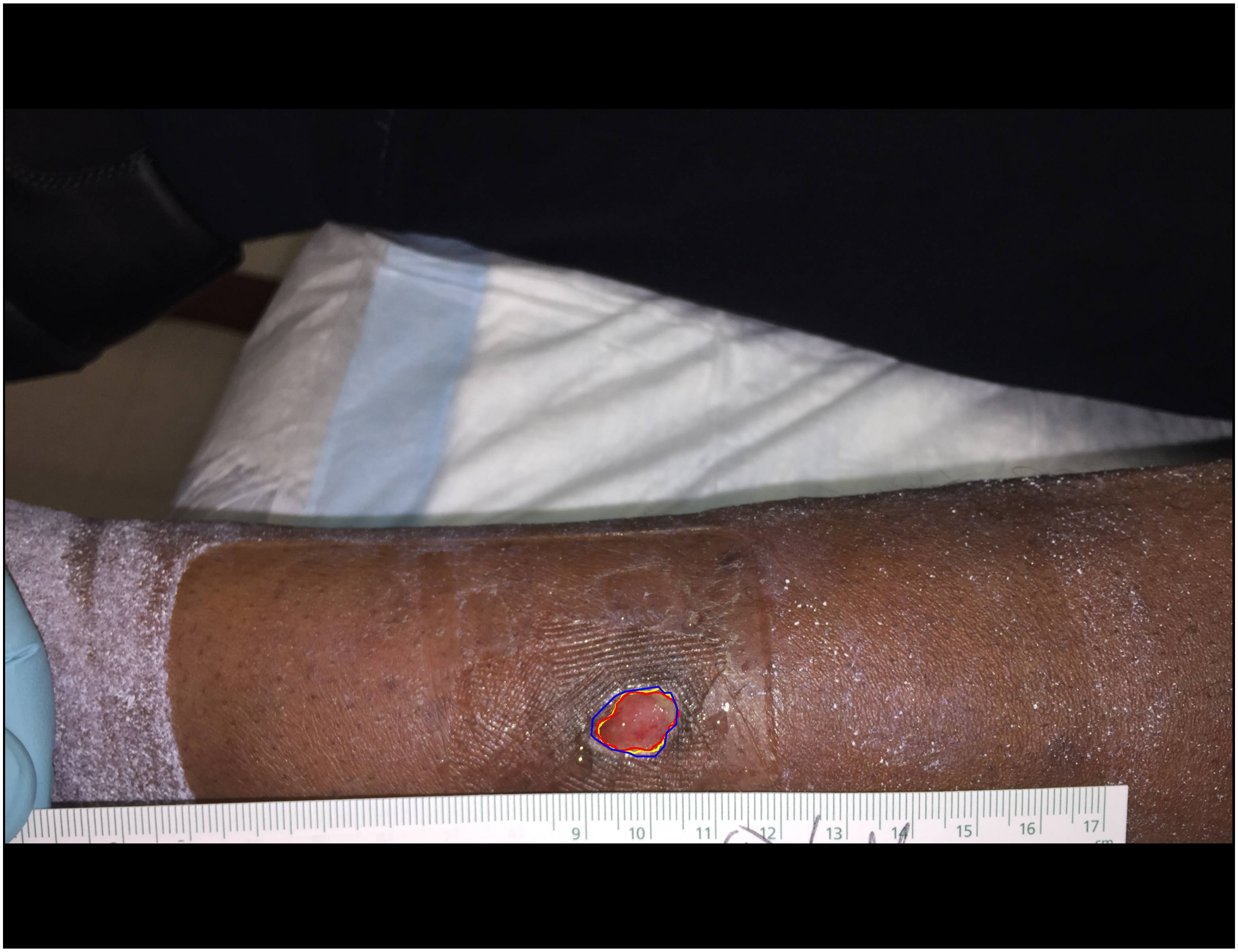

Tracing Data			
Tracer:	Wound Area (px^2)	Ruler Calibration (px/cm)	Wound Area (cm^2)
H1	25447	173.0	0.85
H2	23934	172.0	0.81
AI	30884	175.7	1.00

Tracing Comparisons				
Difference Metric:	Human-Human		Human-AI	
	H1(ref) H2(test)	H2(ref) H1(test)	H1(ref) AI(test)	H2(ref) AI(test)
False Negative Area (%)	7.1	1.2	0.4	0.3
False Positive Area (%)	1.1	7.5	21.8	29.3
|Relative Error (%)|	5.9	6.3	21.4	29.0

Blinded Attending Surgeon Review						
Reviewer	PGT Estimate	H1 meets definition?	H2 meets definition?	AI meets definition?	Which is AI?	Which is most accurate?
1	50	Yes	Yes	No	H2	H2
2	0	Yes	Yes	No	AI	AI
3	90	Yes	Yes	No	H1	AI

© 2021 Howell RS et al. *JAMA Network Open.*

Wound EMR Information								
Sequential Number	Gender	Age	Wound Type	Wound Location	Wound Length (cm)	Wound Width (cm)	Wound Depth (cm)	Wound Area (cm^2)
110	M	65	DFU	R dorsal foot	1.8	2.4	0.2	4.32

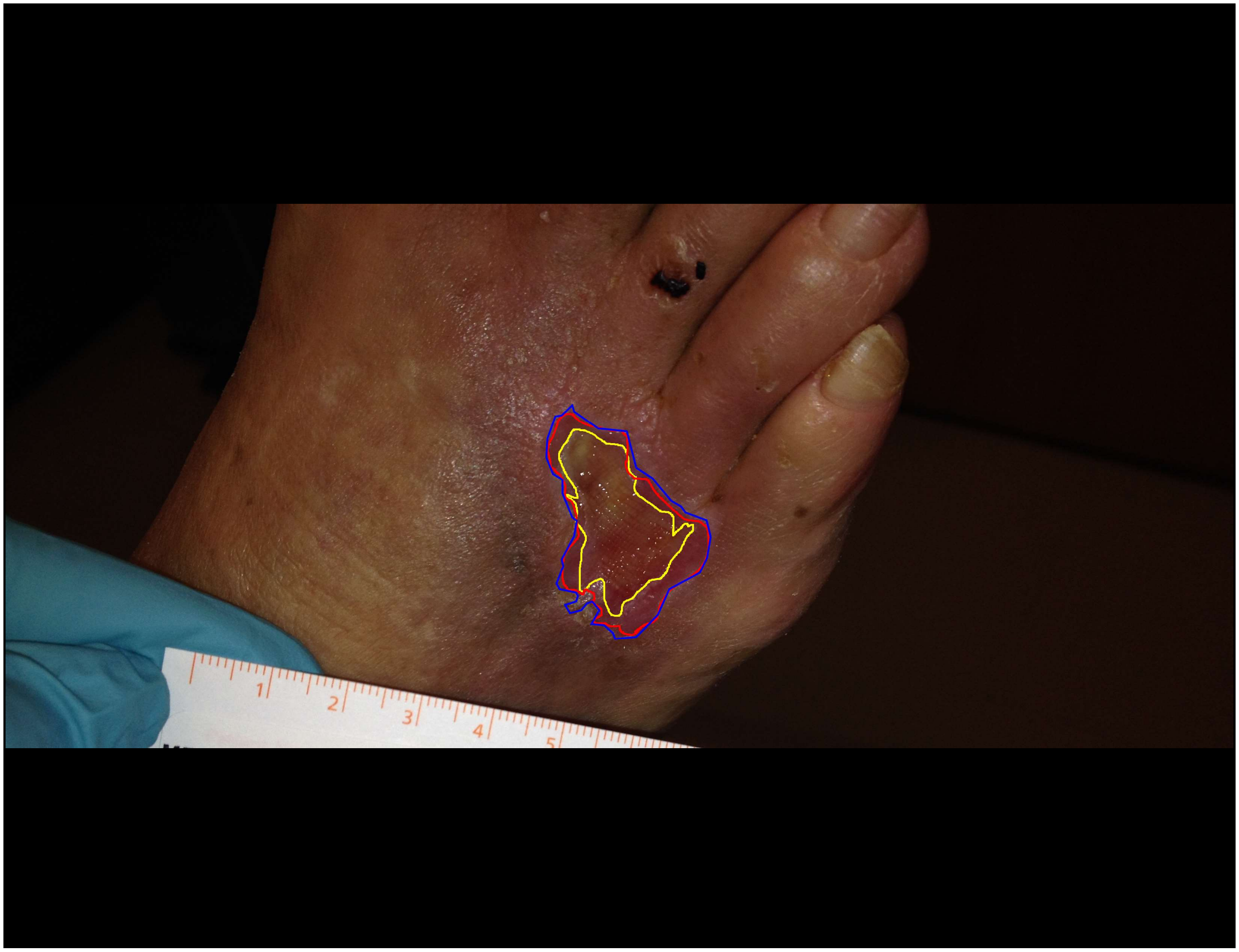

Tracing Data			
Tracer:	Wound Area (px^2)	Ruler Calibration (px/cm)	Wound Area (cm^2)
H1	88247	196.7	2.28
H2	138280	195.6	3.61
AI	153749	201.1	3.80

Tracing Comparisons				
Difference Metric:	Human-Human		Human-AI	
	H1(ref) H2(test)	H2(ref) H1(test)	H1(ref) AI(test)	H2(ref) AI(test)
False Negative Area (%)	0.4	36.4	0.0	0.2
False Positive Area (%)	57.1	0.2	74.2	11.4
|Relative Error (%)|	56.7	36.2	74.2	11.2

Blinded Attending Surgeon Review						
Reviewer	PGT Estimate	H1 meets definition?	H2 meets definition?	AI meets definition?	Which is AI?	Which is most accurate?
1	30	No	Yes	Yes	H2	H2
2	0	Yes	No	No	AI	H1
3	40	No	Yes	Yes	AI	AI

© 2021 Howell RS et al. *JAMA Network Open.*

eAppendix 2. Individual Photographs From Site 2

Wound EMR Information								
**Sequential Number**	**Gender**	**Age**	**Wound Type**	**Wound Location**	**Wound Length (cm)**	**Wound Width (cm)**	**Wound Depth (cm)**	**Wound Area (cm^2)**
1	F	70	Pressure	Sacrum	3.0			

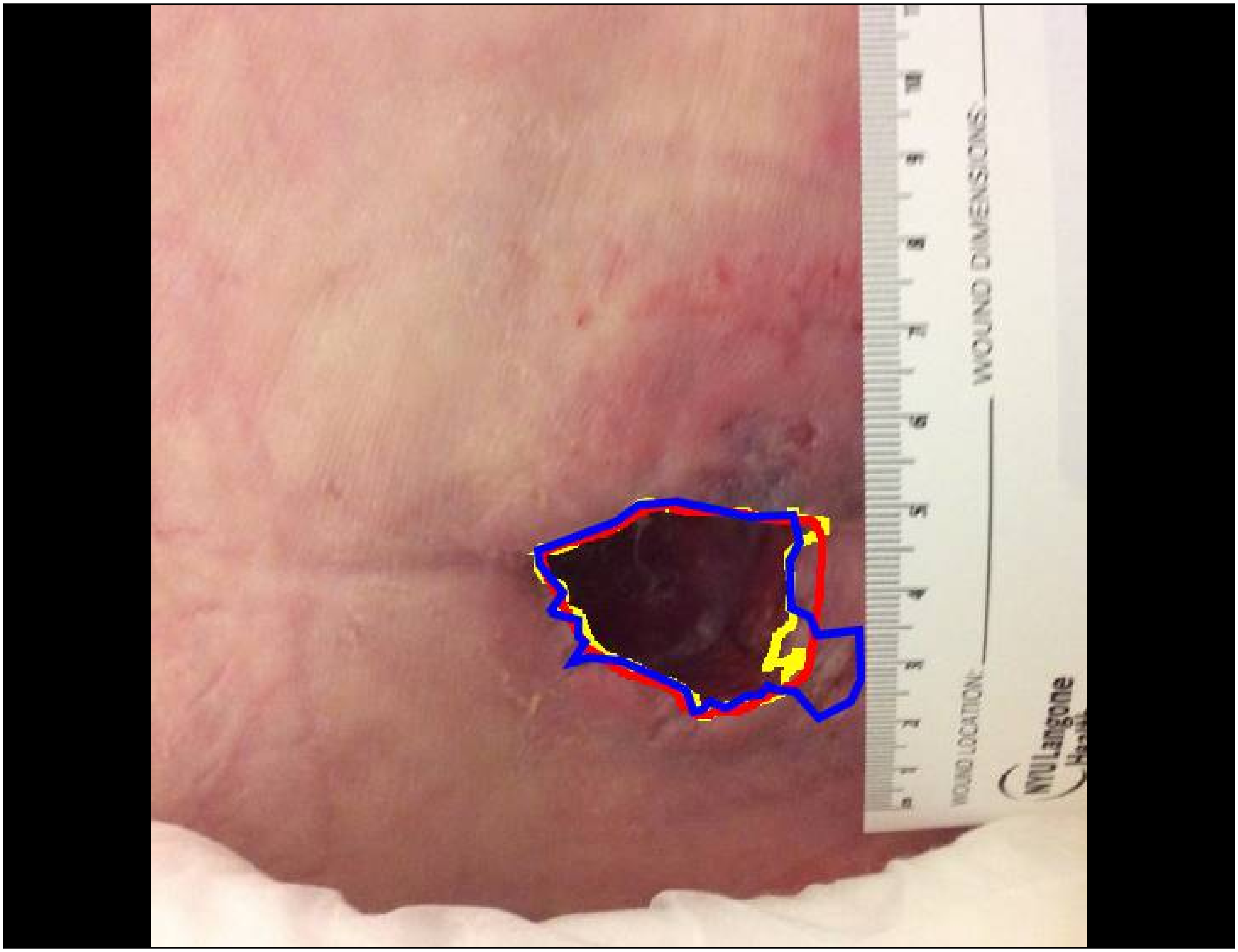

Tracing Data			
**Tracer:**	**Wound Area (px^2)**	**Ruler Calibration (px/cm)**	**Wound Area (cm^2)**
**H1**	13142	42.7	7.22
**H2**	14813	46.0	7.01
**AI**	15354	51.9	5.70

Tracing Comparisons				
**Difference Metric:**	**Human-Human**		**Human-AI**	
	**H1(ref) H2(test)**	**H2(ref) H1(test)**	**H1(ref) AI(test)**	**H2(ref) AI(test)**
**False Negative Area (%)**	2.2	13.2	3.1	9.2
**False Positive Area (%)**	14.9	1.9	20.0	12.9
**|Relative Error (%)|**	12.7	11.3	16.8	3.7

Blinded Attending Surgeon Review						
**Reviewer**	**PGT Estimate**	**H1 meets definition?**	**H2 meets definition?**	**AI meets definition?**	**Which is AI?**	**Which is most accurate?**
**1**	90	Yes	Yes	No	AI	H1
**2**	50	No	Yes	Yes	H2	H1

© 2021 Howell RS et al. *JAMA Network Open.*

**3**	80	Yes	No	No	AI	H2

© 2021 Howell RS et al. *JAMA Network Open.*

Wound EMR Information								
Sequential Number	Gender	Age	Wound Type	Wound Location	Wound Length (cm)	Wound Width (cm)	Wound Depth (cm)	Wound Area (cm^2)
2	F	22	Surgical	Breast-R				

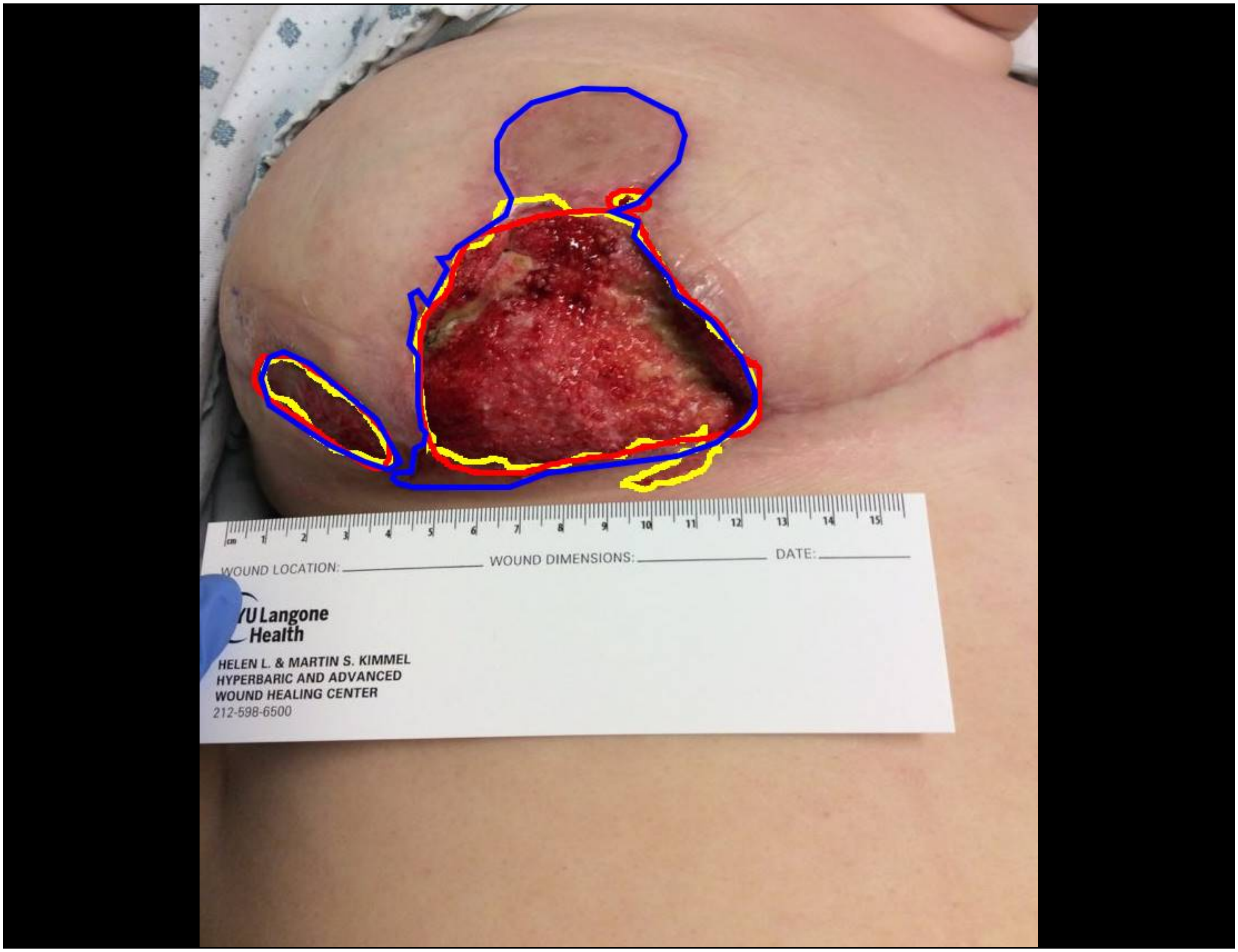

Tracing Data			
Tracer:	Wound Area (px^2)	Ruler Calibration (px/cm)	Wound Area (cm^2)
H1	58778	39.0	38.70
H2	59486	38.8	39.51
AI	78633	41.4	45.80

Tracing Comparisons				
Difference Metric:	Human-Human		Human-AI	
	H1(ref) H2(test)	H2(ref) H1(test)	H1(ref) AI(test)	H2(ref) AI(test)
False Negative Area (%)	5.1	6.2	3.0	2.6
False Positive Area (%)	6.3	5.0	36.8	34.8
|Relative Error (%)|	1.2	1.2	33.8	32.2

Blinded Attending Surgeon Review						
Reviewer	PGT Estimate	H1 meets definition?	H2 meets definition?	AI meets definition?	Which is AI?	Which is most accurate?
1	90	Yes	Yes	No	H1	AI
2	100	Yes	Yes	Yes	AI	H2
3	70	Yes	No	No	H1	H2

© 2021 Howell RS et al. *JAMA Network Open.*

Wound EMR Information								
Sequential Number	Gender	Age	Wound Type	Wound Location	Wound Length (cm)	Wound Width (cm)	Wound Depth (cm)	Wound Area (cm^2)
4	M	59	Surgical	Back	4.0			

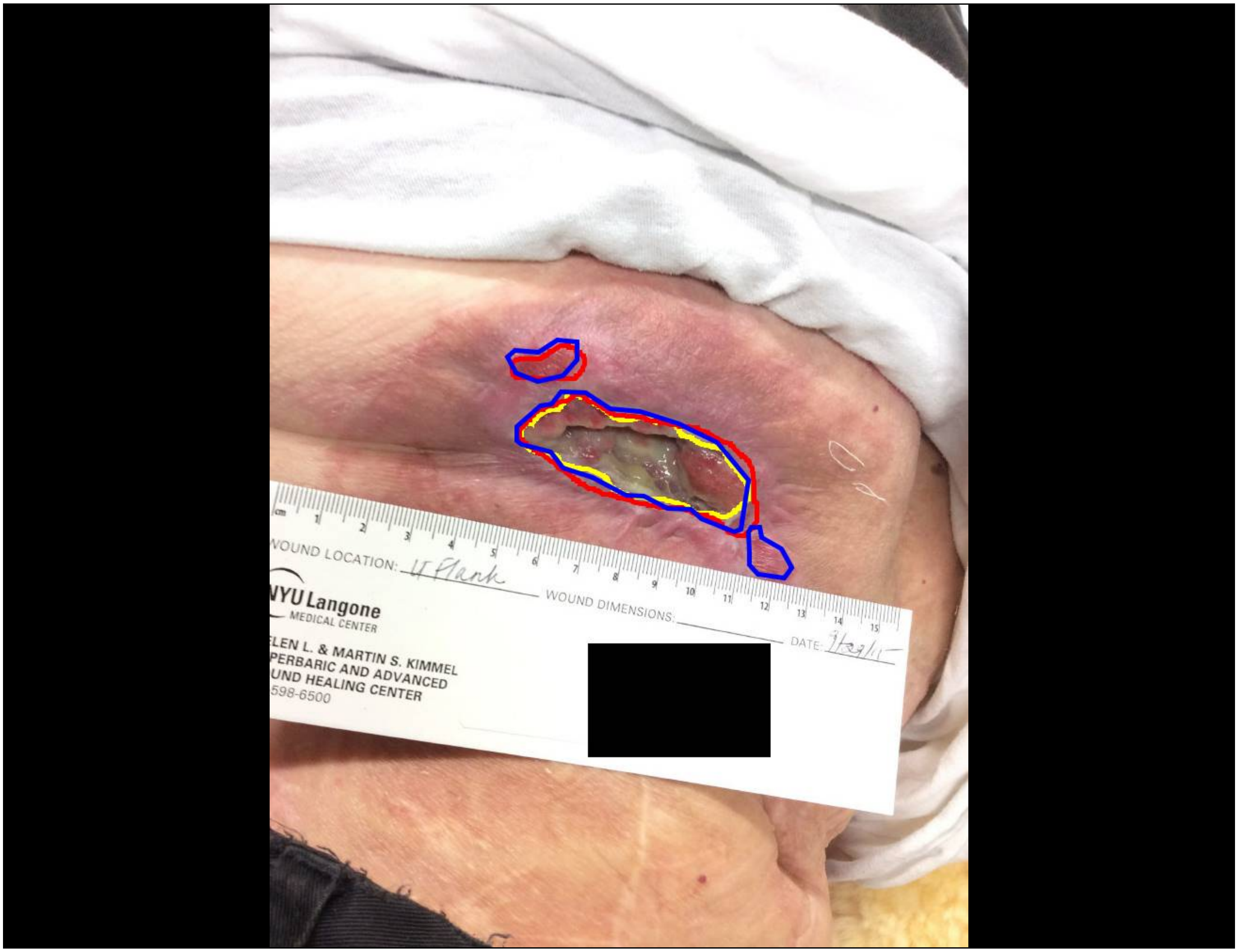

Tracing Data			
Tracer:	Wound Area (px^2)	Ruler Calibration (px/cm)	Wound Area (cm^2)
H1	16050	42.7	8.80
H2	21964	50.1	8.76
AI	22408	38.3	15.30

Tracing Comparisons				
Difference Metric:	Human-Human		Human-AI	
	H1(ref) H2(test)	H2(ref) H1(test)	H1(ref) AI(test)	H2(ref) AI(test)
False Negative Area (%)	0.7	27.5	0.8	12.2
False Positive Area (%)	37.6	0.5	40.4	14.2
|Relative Error (%)|	36.8	26.9	39.6	2.0

Blinded Attending Surgeon Review						
Reviewer	PGT Estimate	H1 meets definition?	H2 meets definition?	AI meets definition?	Which is AI?	Which is most accurate?
1	10	No	Yes	No	H2	H2
2	20	Yes	Yes	Yes	H2	AI
3	20	Yes	No	No	H1	H2

© 2021 Howell RS et al. *JAMA Network Open.*

Wound EMR Information								
Sequential Number	Gender	Age	Wound Type	Wound Location	Wound Length (cm)	Wound Width (cm)	Wound Depth (cm)	Wound Area (cm^2)
5	F	93	Traumatic	Knee				

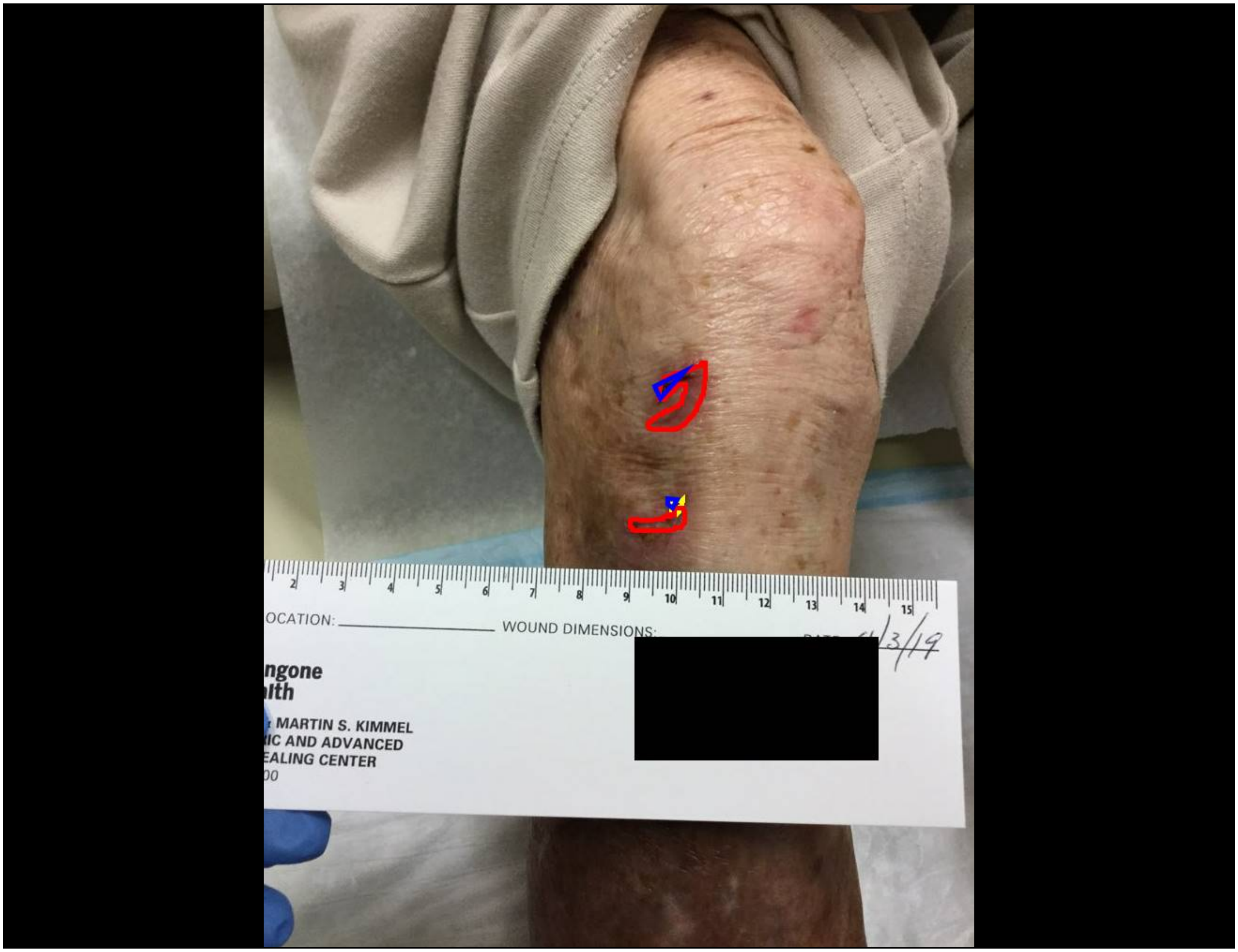

Tracing Data			
Tracer:	Wound Area (px^2)	Ruler Calibration (px/cm)	Wound Area (cm^2)
H1	121	50.7	0.05
H2	2932	57.1	0.90
AI	308	39.2	0.20

Tracing Comparisons				
Difference Metric:	Human-Human		Human-AI	
	H1(ref) H2(test)	H2(ref) H1(test)	H1(ref) AI(test)	H2(ref) AI(test)
False Negative Area (%)	76.0	99.0	81.0	92.4
False Positive Area (%)	2399.2	3.1	235.5	2.9
|Relative Error (%)|	2323.1	95.9	154.5	89.5

Blinded Attending Surgeon Review						
Reviewer	PGT Estimate	H1 meets definition?	H2 meets definition?	AI meets definition?	Which is AI?	Which is most accurate?
1	80	No	Yes	No	H2	H1
2	90	No	Yes	No	H1	H1
3	100	No	No	No	H1	H1

© 2021 Howell RS et al. *JAMA Network Open.*

Wound EMR Information								
Sequential Number	Gender	Age	Wound Type	Wound Location	Wound Length (cm)	Wound Width (cm)	Wound Depth (cm)	Wound Area (cm^2)
6	F	85	Traumatic	Leg	1.8	0.8	0.1	1.44

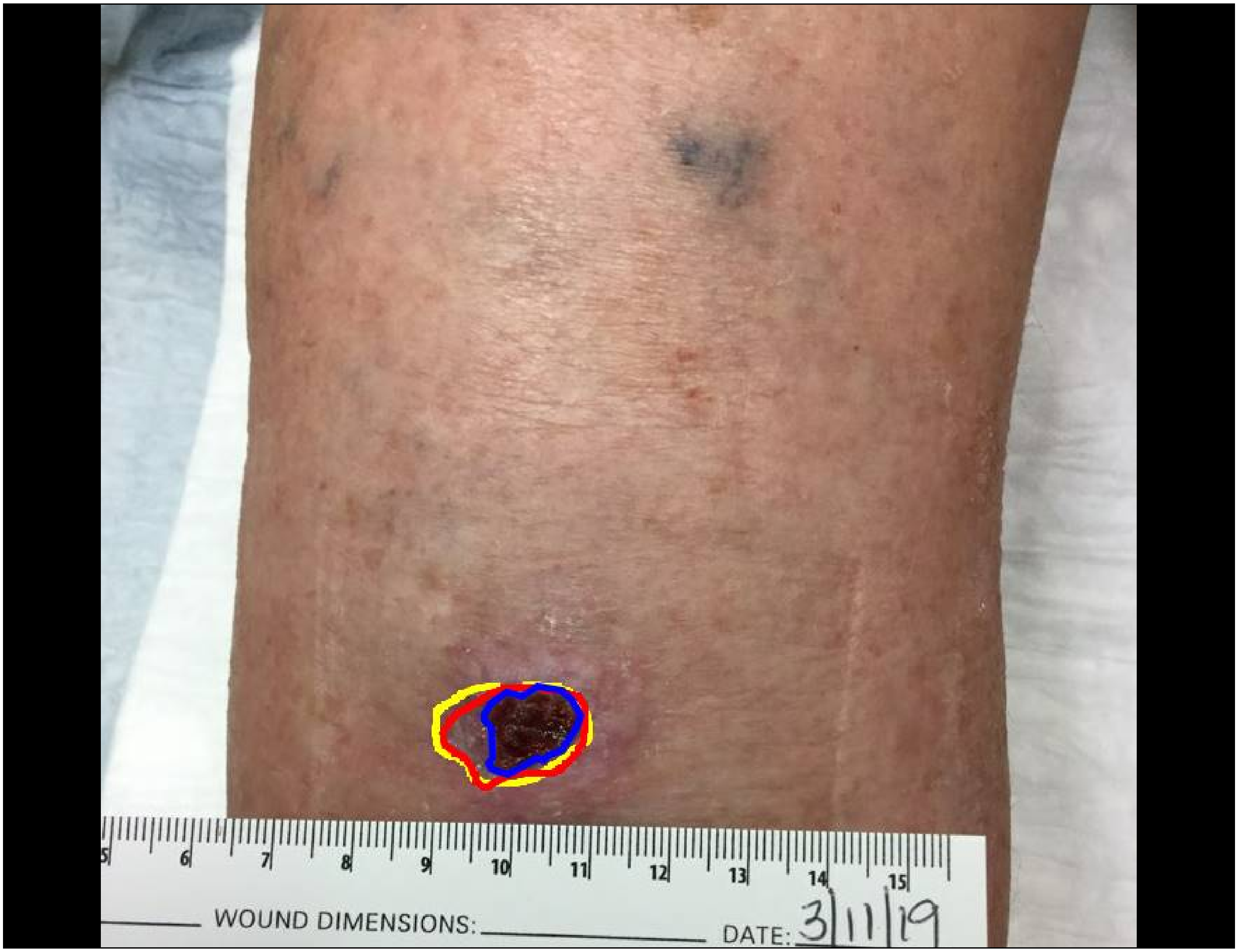

Tracing Data			
Tracer:	Wound Area (px^2)	Ruler Calibration (px/cm)	Wound Area (cm^2)
H1	5886	56.7	1.83
H2	5153	58.2	1.52
AI	2929	57.0	0.90

Tracing Comparisons				
Difference Metric:	Human-Human		Human-AI	
	H1(ref) H2(test)	H2(ref) H1(test)	H1(ref) AI(test)	H2(ref) AI(test)
False Negative Area (%)	15.4	3.4	50.2	43.3
False Positive Area (%)	3.0	17.6	0.0	0.2
|Relative Error (%)|	12.5	14.2	50.2	43.2

Blinded Attending Surgeon Review						
Reviewer	PGT Estimate	H1 meets definition?	H2 meets definition?	AI meets definition?	Which is AI?	Which is most accurate?
1	60	Yes	Yes	No	H1	AI
2	100	Yes	Yes	No	AI	AI
3	30	No	No	Yes	H2	AI

© 2021 Howell RS et al. *JAMA Network Open.*

Wound EMR Information								
Sequential Number	Gender	Age	Wound Type	Wound Location	Wound Length (cm)	Wound Width (cm)	Wound Depth (cm)	Wound Area (cm^2)
7	F	64	Pressure	Right ischium				

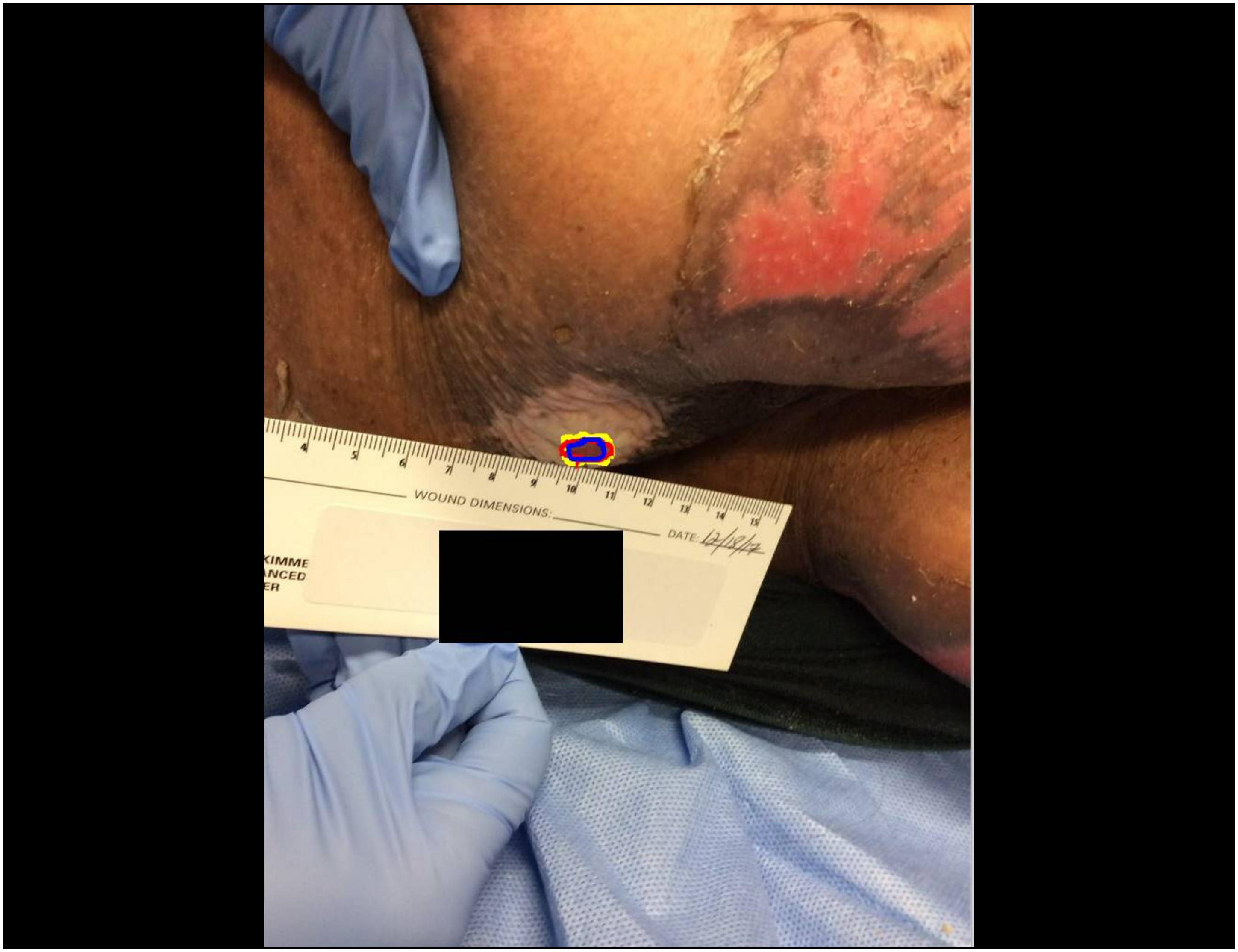

Tracing Data			
Tracer:	Wound Area (px^2)	Ruler Calibration (px/cm)	Wound Area (cm^2)
H1	1370	44.3	0.70
H2	891	44.5	0.45
AI	668	47.2	0.30

Tracing Comparisons				
Difference Metric:	Human-Human		Human-AI	
	H1(ref) H2(test)	H2(ref) H1(test)	H1(ref) AI(test)	H2(ref) AI(test)
False Negative Area (%)	36.1	1.8	51.4	28.7
False Positive Area (%)	1.2	55.6	0.1	3.7
|Relative Error (%)|	35.0	53.8	51.2	25.0

Blinded Attending Surgeon Review						
Reviewer	PGT Estimate	H1 meets definition?	H2 meets definition?	AI meets definition?	Which is AI?	Which is most accurate?
1	100	Yes	Yes	Yes	H2	H1
2	100	Yes	Yes	Yes	AI	H2
3	100	Yes	No	No	AI	H2

© 2021 Howell RS et al. *JAMA Network Open.*

Wound EMR Information								
Sequential Number	Gender	Age	Wound Type	Wound Location	Wound Length (cm)	Wound Width (cm)	Wound Depth (cm)	Wound Area (cm^2)
8	F	65	Traumatic	leg	3.5	1.5	0.2	5.25

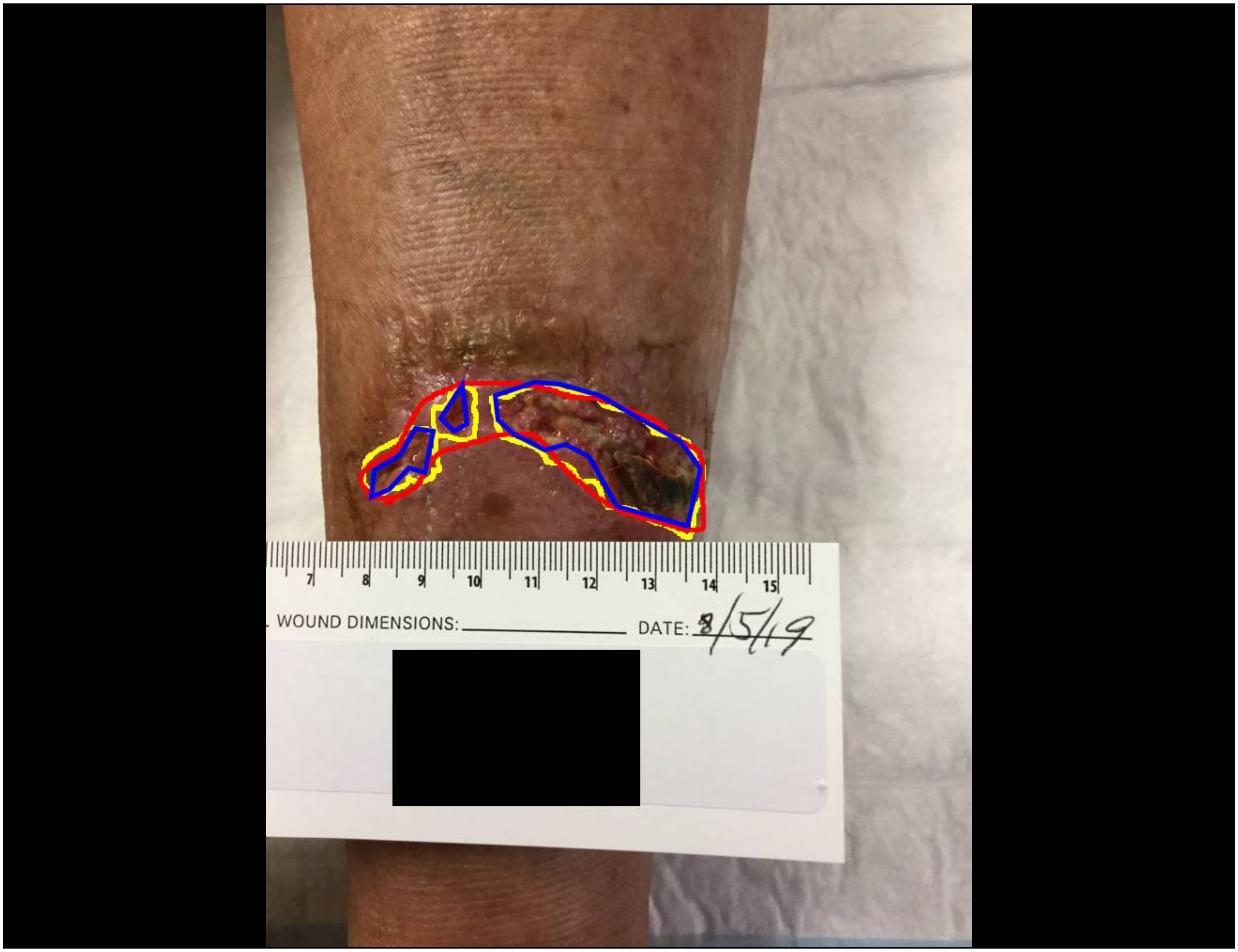

Tracing Data			
Tracer:	Wound Area (px^2)	Ruler Calibration (px/cm)	Wound Area (cm^2)
H1	24211	60.7	6.56
H2	27251	50.7	10.61
AI	18843	60.2	5.20

Tracing Comparisons				
Difference Metric:	Human-Human		Human-AI	
	H1(ref) H2(test)	H2(ref) H1(test)	H1(ref) AI(test)	H2(ref) AI(test)
False Negative Area (%)	6.7	17.1	24.4	33.6
False Positive Area (%)	19.3	6.0	2.3	2.7
|Relative Error (%)|	12.6	11.2	22.2	30.9

Blinded Attending Surgeon Review						
Reviewer	PGT Estimate	H1 meets definition?	H2 meets definition?	AI meets definition?	Which is AI?	Which is most accurate?
1	70	Yes	No	Yes	H1	AI
2	60	Yes	Yes	Yes	AI	AI
3	60	No	No	No	AI	H2

© 2021 Howell RS et al. *JAMA Network Open.*

Wound EMR Information								
Sequential Number	Gender	Age	Wound Type	Wound Location	Wound Length (cm)	Wound Width (cm)	Wound Depth (cm)	Wound Area (cm^2)
10	F	62	Venous	Ankle	4.0	5.0	3.5	20.00

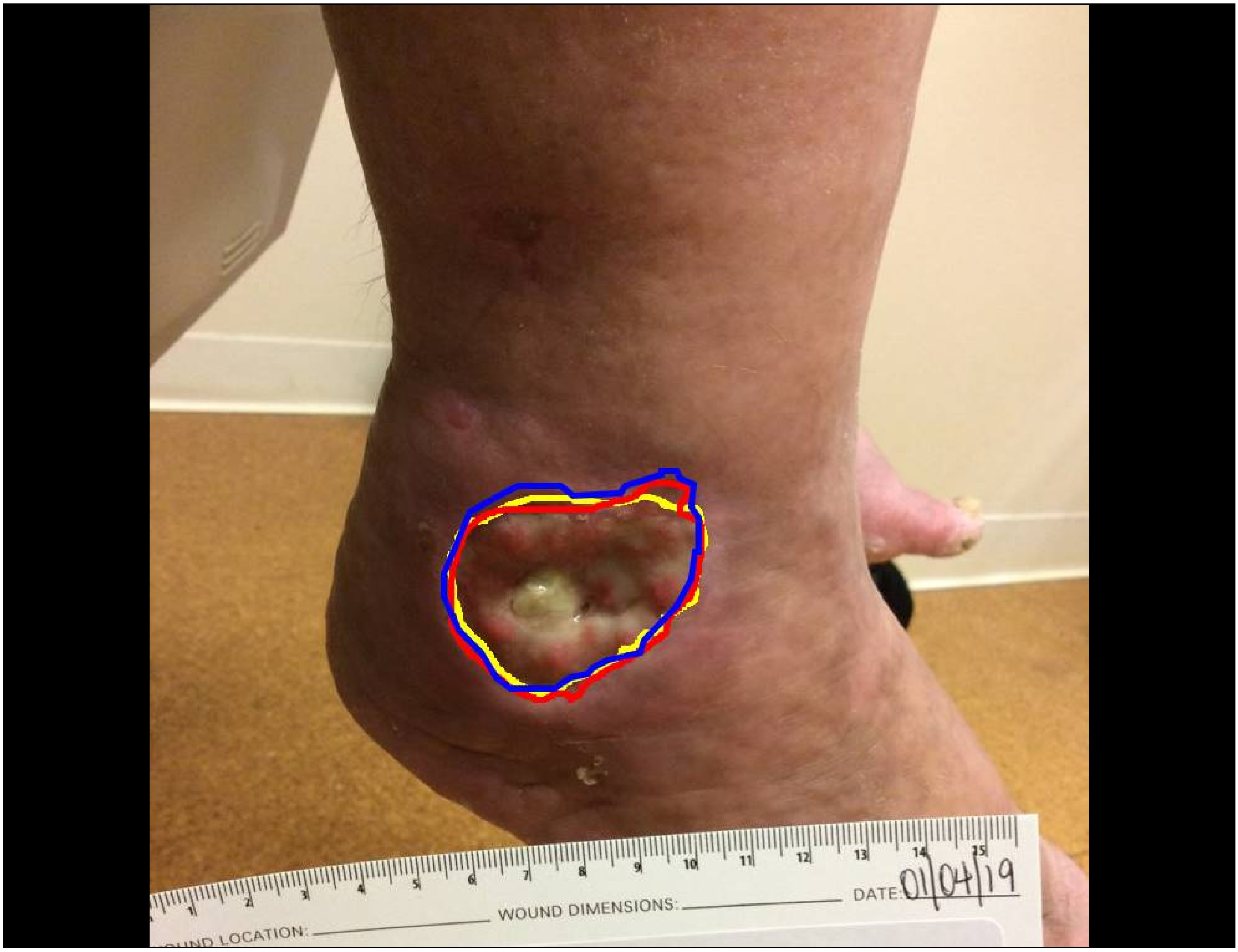

Tracing Data			
Tracer:	Wound Area (px^2)	Ruler Calibration (px/cm)	Wound Area (cm^2)
H1	23714	45.2	11.62
H2	24879	45.4	12.08
AI	26127	44.8	13.00

Tracing Comparisons				
Difference Metric:	Human-Human		Human-AI	
	H1(ref) H2(test)	H2(ref) H1(test)	H1(ref) AI(test)	H2(ref) AI(test)
False Negative Area (%)	4.0	8.5	2.2	5.2
False Positive Area (%)	8.9	3.8	12.3	10.3
|Relative Error (%)|	4.9	4.7	10.2	5.0

Blinded Attending Surgeon Review						
Reviewer	PGT Estimate	H1 meets definition?	H2 meets definition?	AI meets definition?	Which is AI?	Which is most accurate?
1	30	Yes	Yes	Yes	H1	H2
2	50	Yes	Yes	Yes	AI	H2
3	50	No	Yes	No	H2	H1

© 2021 Howell RS et al. *JAMA Network Open.*

Wound EMR Information								
Sequential Number	Gender	Age	Wound Type	Wound Location	Wound Length (cm)	Wound Width (cm)	Wound Depth (cm)	Wound Area (cm^2)
11	F	33	Diabetic	Toe				

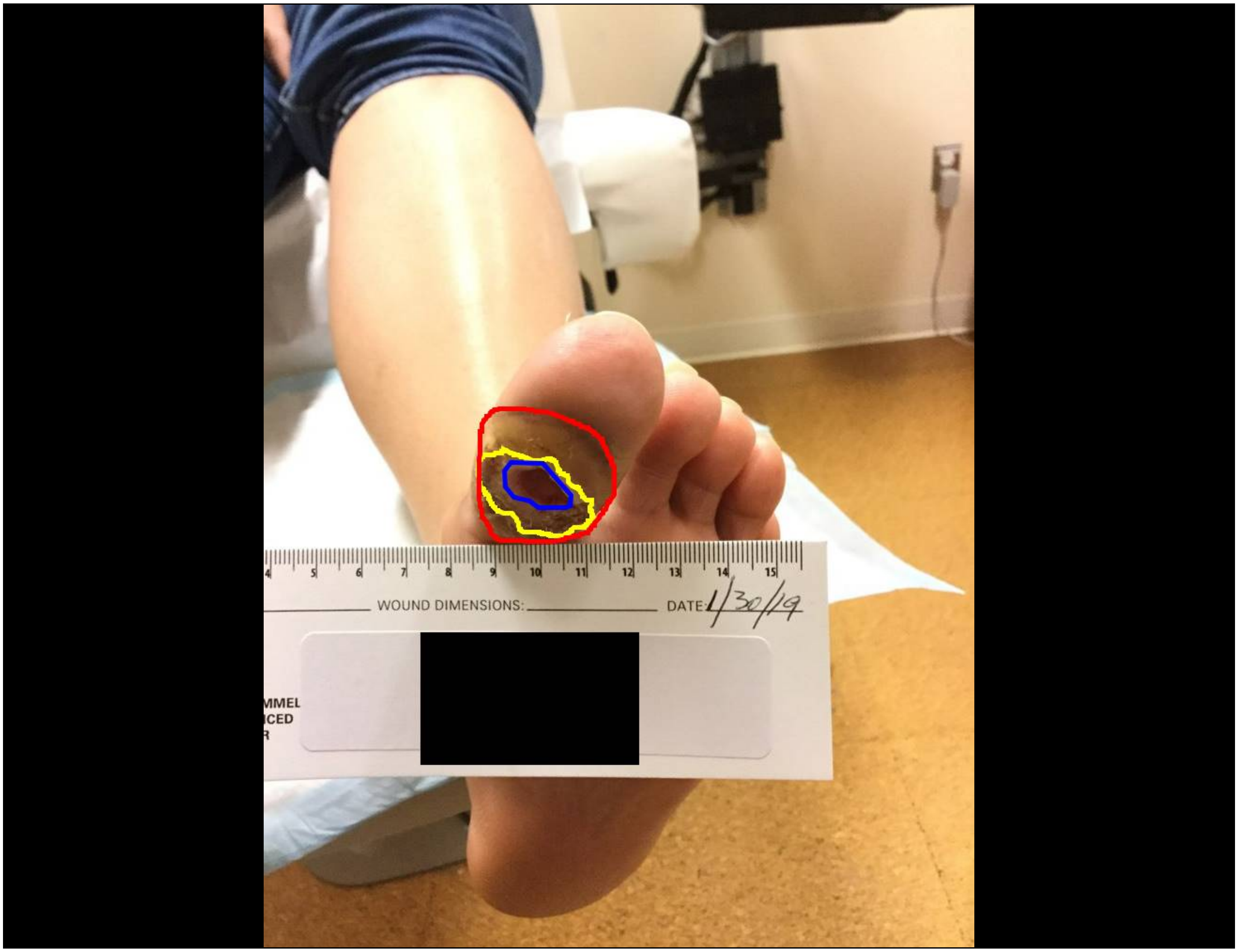

Tracing Data			
Tracer:	Wound Area (px^2)	Ruler Calibration (px/cm)	Wound Area (cm^2)
H1	7269	48.0	3.15
H2	17152	49.2	7.09
AI	2422	51.9	0.90

Tracing Comparisons				
Difference Metric:	Human-Human		Human-AI	
	H1(ref) H2(test)	H2(ref) H1(test)	H1(ref) AI(test)	H2(ref) AI(test)
False Negative Area (%)	0.2	57.7	66.7	85.9
False Positive Area (%)	136.1	0.1	0.0	0.0
|Relative Error (%)|	136.0	57.6	66.7	85.9

Blinded Attending Surgeon Review						
Reviewer	PGT Estimate	H1 meets definition?	H2 meets definition?	AI meets definition?	Which is AI?	Which is most accurate?
1	90	No	No	Yes	H1	H1
2	50	Yes	No	Yes	H2	H1
3	100	No	No	Yes	H1	AI

© 2021 Howell RS et al. *JAMA Network Open.*

Wound EMR Information								
Sequential Number	Gender	Age	Wound Type	Wound Location	Wound Length (cm)	Wound Width (cm)	Wound Depth (cm)	Wound Area (cm²)
12	M	81	Pressure	Ankle				

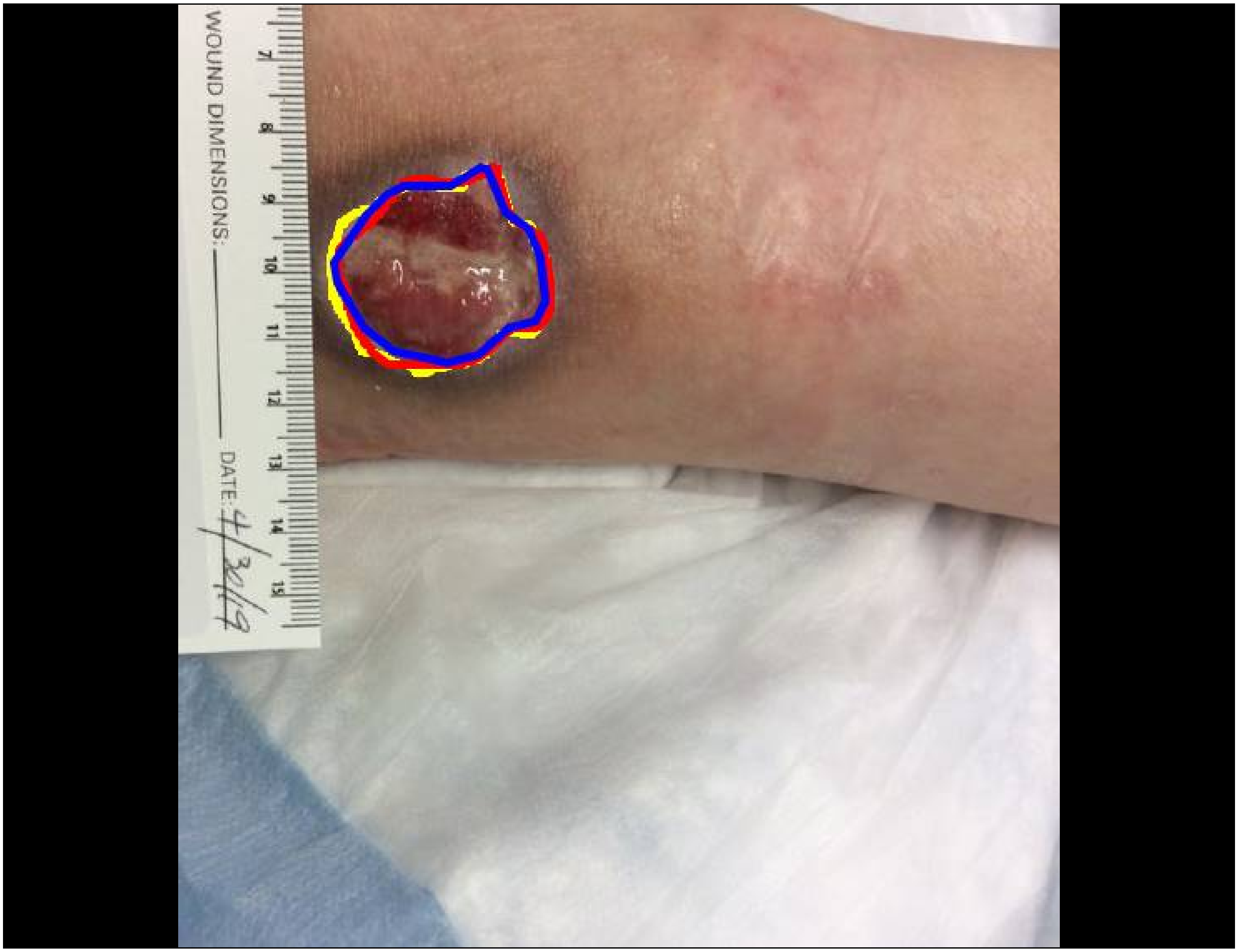

Tracing Data			
Tracer:	Wound Area (px²)	Ruler Calibration (px/cm)	Wound Area (cm²)
H1	11455	41.7	6.59
H2	10924	43.0	5.90
AI	10207	36.9	7.50

Tracing Comparisons				
Difference Metric:	Human-Human		Human-AI	
	H1(ref) H2(test)	H2(ref) H1(test)	H1(ref) AI(test)	H2(ref) AI(test)
False Negative Area (%)	7.0	2.5	11.2	8.2
False Positive Area (%)	2.4	7.4	0.3	1.7
|Relative Error (%)|	4.6	4.9	10.9	6.6

Blinded Attending Surgeon Review						
Reviewer	PGT Estimate	H1 meets definition?	H2 meets definition?	AI meets definition?	Which is AI?	Which is most accurate?
1	100	Yes	Yes	Yes	H1	H2
2	80	Yes	Yes	Yes	H2	AI
3	80	No	No	Yes	H2	AI

© 2021 Howell RS et al. *JAMA Network Open.*

Wound EMR Information								
Sequential Number	Gender	Age	Wound Type	Wound Location	Wound Length (cm)	Wound Width (cm)	Wound Depth (cm)	Wound Area (cm²)
13	F	83	Traumatic	Left anterior				

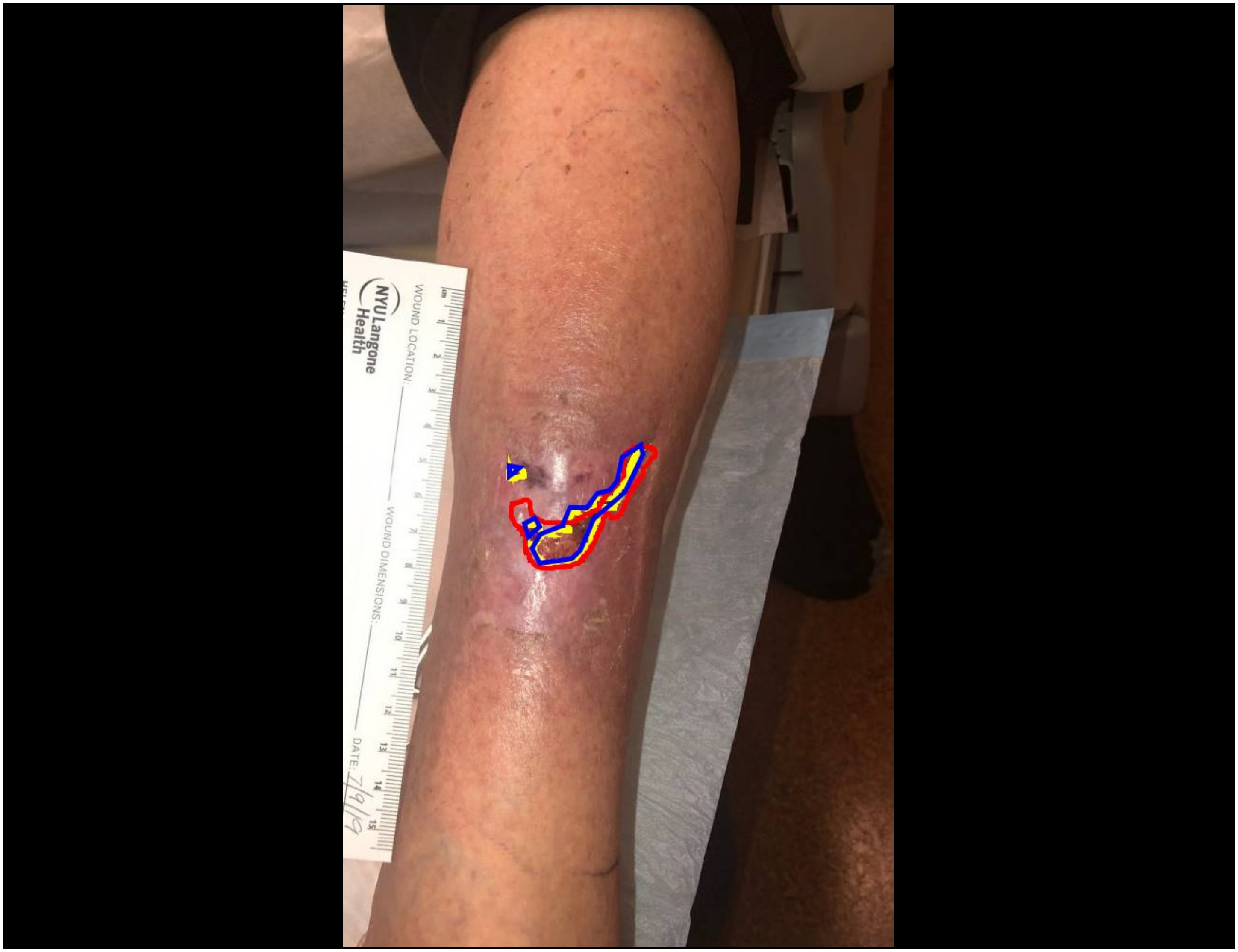

Tracing Data			
Tracer:	Wound Area (px²)	Ruler Calibration (px/cm)	Wound Area (cm²)
H1	3147	37.2	2.28
H2	5711	35.7	4.47
AI	3741	40.3	2.30

Tracing Comparisons				
Difference Metric:	Human-Human		Human-AI	
	H1(ref) H2(test)	H2(ref) H1(test)	H1(ref) AI(test)	H2(ref) AI(test)
False Negative Area (%)	9.6	50.2	19.0	45.5
False Positive Area (%)	91.1	5.3	37.8	11.0
|Relative Error (%)|	81.5	44.9	18.9	34.5

Blinded Attending Surgeon Review						
Reviewer	PGT Estimate	H1 meets definition?	H2 meets definition?	AI meets definition?	Which is AI?	Which is most accurate?
1	90	Yes	No	Yes	H1	H2
2	70	Yes	Yes	Yes	H1	H1
3	20	No	Yes	No	AI	H1

© 2021 Howell RS et al. *JAMA Network Open.*

Wound EMR Information								
Sequential Number	Gender	Age	Wound Type	Wound Location	Wound Length (cm)	Wound Width (cm)	Wound Depth (cm)	Wound Area (cm^2)
14	M	78	Radiation	Hip	3.0			

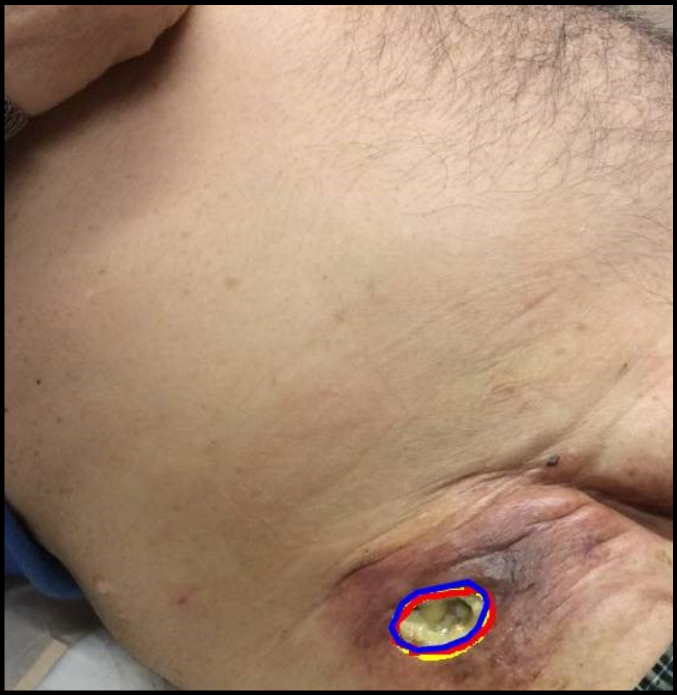

Tracing Data			
Tracer:	Wound Area (px^2)	Ruler Calibration (px/cm)	Wound Area (cm^2)
H1	3357	29.2	3.94
H2	3133	29.3	3.65
AI	3198	29.0	3.80

Tracing Comparisons				
Difference Metric:	Human-Human		Human-AI	
	H1(ref) H2(test)	H2(ref) H1(test)	H1(ref) AI(test)	H2(ref) AI(test)
False Negative Area (%)	8.9	2.4	15.0	11.3
False Positive Area (%)	2.2	9.5	10.3	13.4
|Relative Error (%)|	6.7	7.1	4.7	2.1

Blinded Attending Surgeon Review						
Reviewer	PGT Estimate	H1 meets definition?	H2 meets definition?	AI meets definition?	Which is AI?	Which is most accurate?
1	0	Yes	Yes	Yes	H2	H1
2	10	Yes	Yes	Yes	AI	AI
3	0	Yes	Yes	Yes	H2	AI

© 2021 Howell RS et al. *JAMA Network Open.*

Wound EMR Information								
Sequential Number	Gender	Age	Wound Type	Wound Location	Wound Length (cm)	Wound Width (cm)	Wound Depth (cm)	Wound Area (cm^2)
15	M	56	Pressure	Buttock				

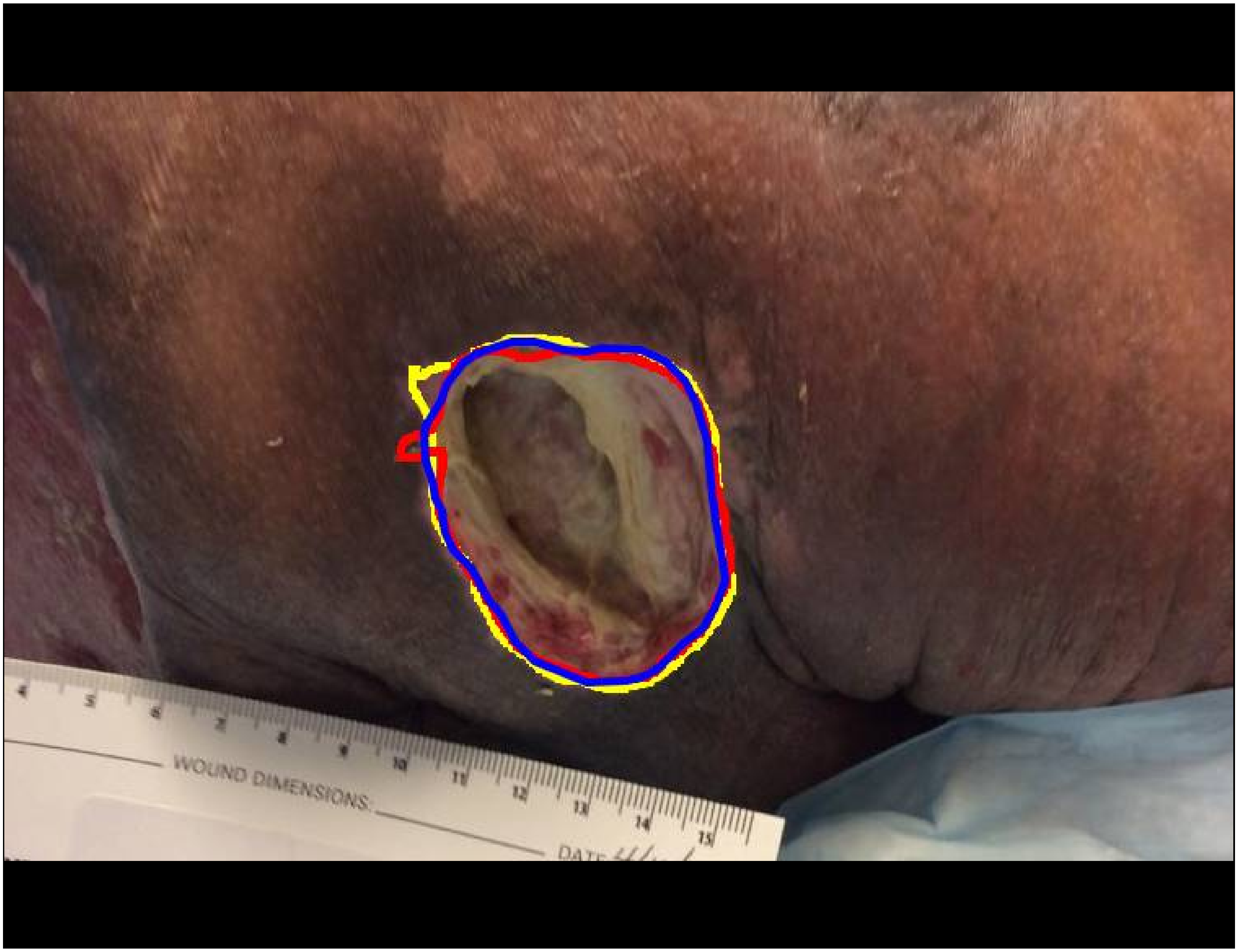

Tracing Data			
Tracer:	Wound Area (px^2)	Ruler Calibration (px/cm)	Wound Area (cm^2)
H1	31310	38.8	20.83
H2	29154	31.9	28.57
AI	29514	40.3	18.20

Tracing Comparisons				
Difference Metric:	Human-Human		Human-AI	
	H1(ref) H2(test)	H2(ref) H1(test)	H1(ref) AI(test)	H2(ref) AI(test)
False Negative Area (%)	7.9	1.1	6.6	2.3
False Positive Area (%)	1.0	8.5	0.8	3.5
|Relative Error (%)|	6.9	7.4	5.7	1.2

Blinded Attending Surgeon Review						
Reviewer	PGT Estimate	H1 meets definition?	H2 meets definition?	AI meets definition?	Which is AI?	Which is most accurate?
1	10	Yes	Yes	Yes	H1	H2
2	30	Yes	Yes	Yes	H1	AI
3	10	Yes	No	No	H2	H2

© 2021 Howell RS et al. *JAMA Network Open.*

Wound EMR Information								
Sequential Number	Gender	Age	Wound Type	Wound Location	Wound Length (cm)	Wound Width (cm)	Wound Depth (cm)	Wound Area (cm^2)
16	F	43	Diabetic	Foot	5.0			

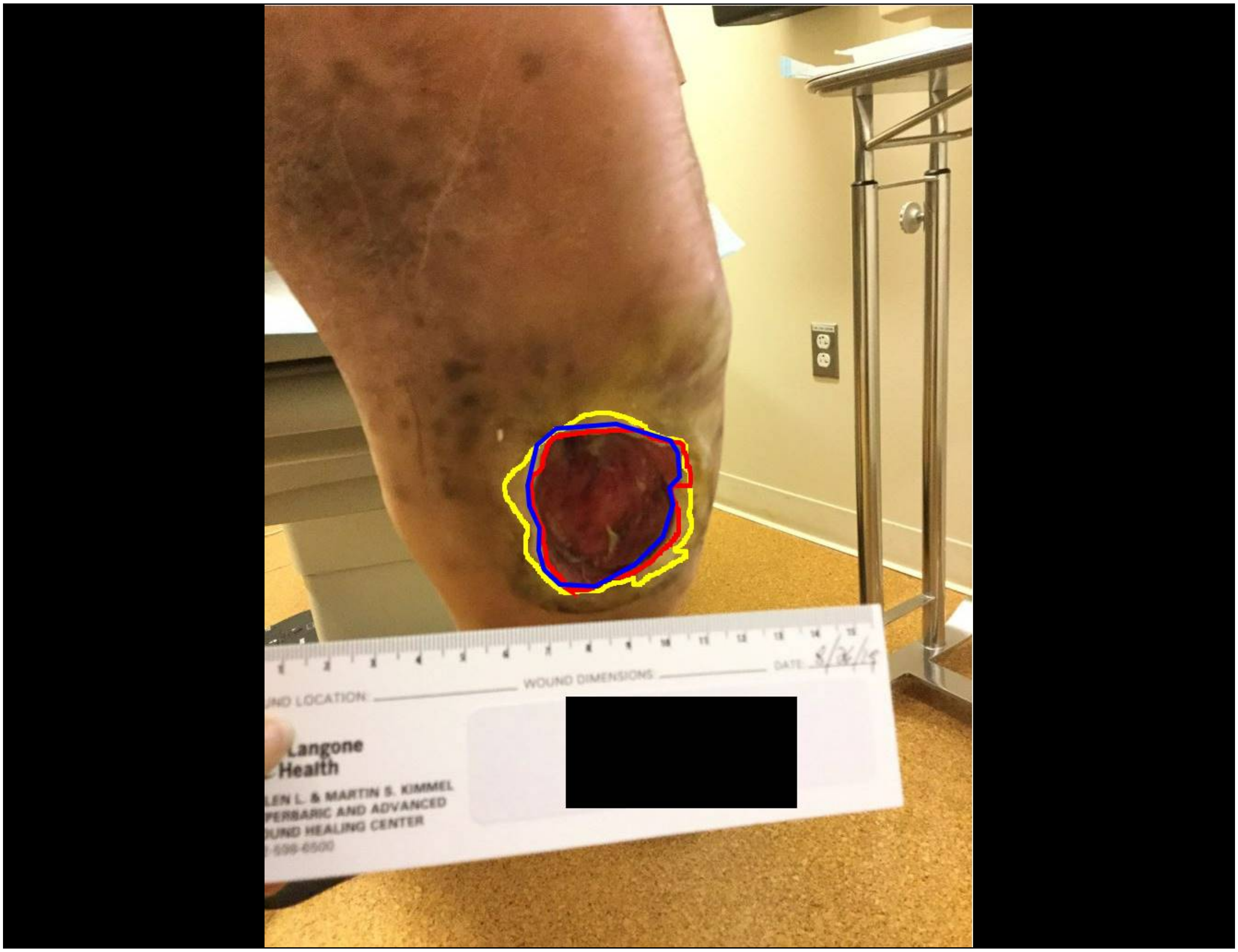

Tracing Data			
Tracer:	Wound Area (px^2)	Ruler Calibration (px/cm)	Wound Area (cm^2)
H1	29634	45.1	14.57
H2	22659	44.4	11.50
AI	22586	46.6	10.40

Tracing Comparisons				
Difference Metric:	Human-Human		Human-AI	
	H1(ref) H2(test)	H2(ref) H1(test)	H1(ref) AI(test)	H2(ref) AI(test)
False Negative Area (%)	23.7	0.3	23.8	7.0
False Positive Area (%)	0.2	31.0	0.0	6.7
|Relative Error (%)|	23.5	30.8	23.8	0.3

Blinded Attending Surgeon Review						
Reviewer	PGT Estimate	H1 meets definition?	H2 meets definition?	AI meets definition?	Which is AI?	Which is most accurate?
1	100	No	Yes	Yes	H2	H2
2	100	No	Yes	Yes	H2	AI
3	90	No	Yes	Yes	H2	H1

© 2021 Howell RS et al. *JAMA Network Open.*

Wound EMR Information								
Sequential Number	Gender	Age	Wound Type	Wound Location	Wound Length (cm)	Wound Width (cm)	Wound Depth (cm)	Wound Area (cm^2)
19	M	60	Arterial	Foot				

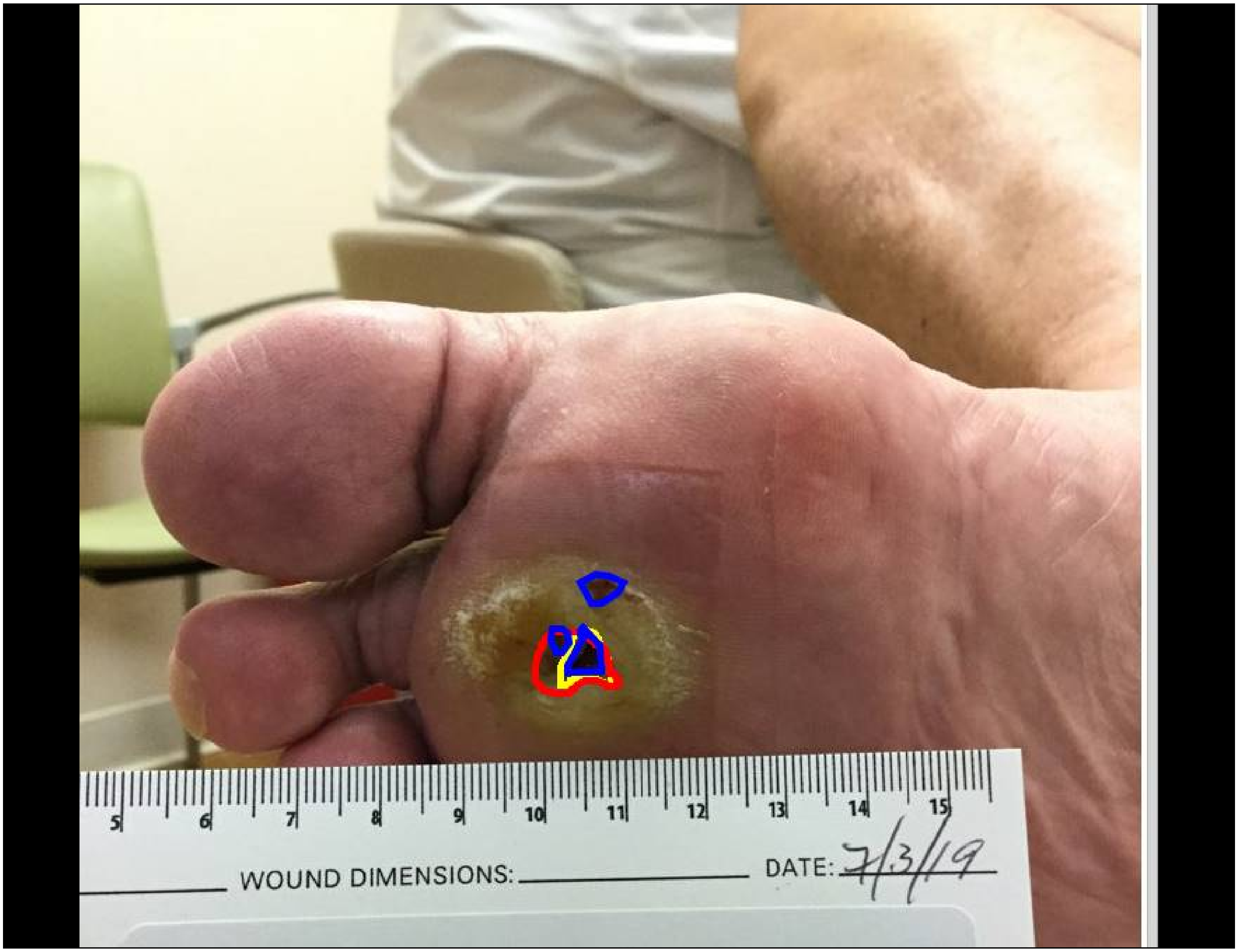

Tracing Data			
Tracer:	Wound Area (px^2)	Ruler Calibration (px/cm)	Wound Area (cm^2)
H1	962	58.6	0.28
H2	1869	58.8	0.54
AI	1056	59.3	0.30

Tracing Comparisons				
Difference Metric:	Human-Human		Human-AI	
	H1(ref) H2(test)	H2(ref) H1(test)	H1(ref) AI(test)	H2(ref) AI(test)
False Negative Area (%)	4.3	50.7	50.4	66.7
False Positive Area (%)	98.5	2.2	60.2	23.2
|Relative Error (%)|	94.3	48.5	9.8	43.5

Blinded Attending Surgeon Review						
Reviewer	PGT Estimate	H1 meets definition?	H2 meets definition?	AI meets definition?	Which is AI?	Which is most accurate?
1	50	No	No	Yes	H2	H2
2	50	Yes	Yes	Yes	AI	AI
3	100	No	Yes	No	H2	H1

© 2021 Howell RS et al. *JAMA Network Open.*

Wound EMR Information								
Sequential Number	Gender	Age	Wound Type	Wound Location	Wound Length (cm)	Wound Width (cm)	Wound Depth (cm)	Wound Area (cm^2)
20	M	84	Pressure	Sacral				

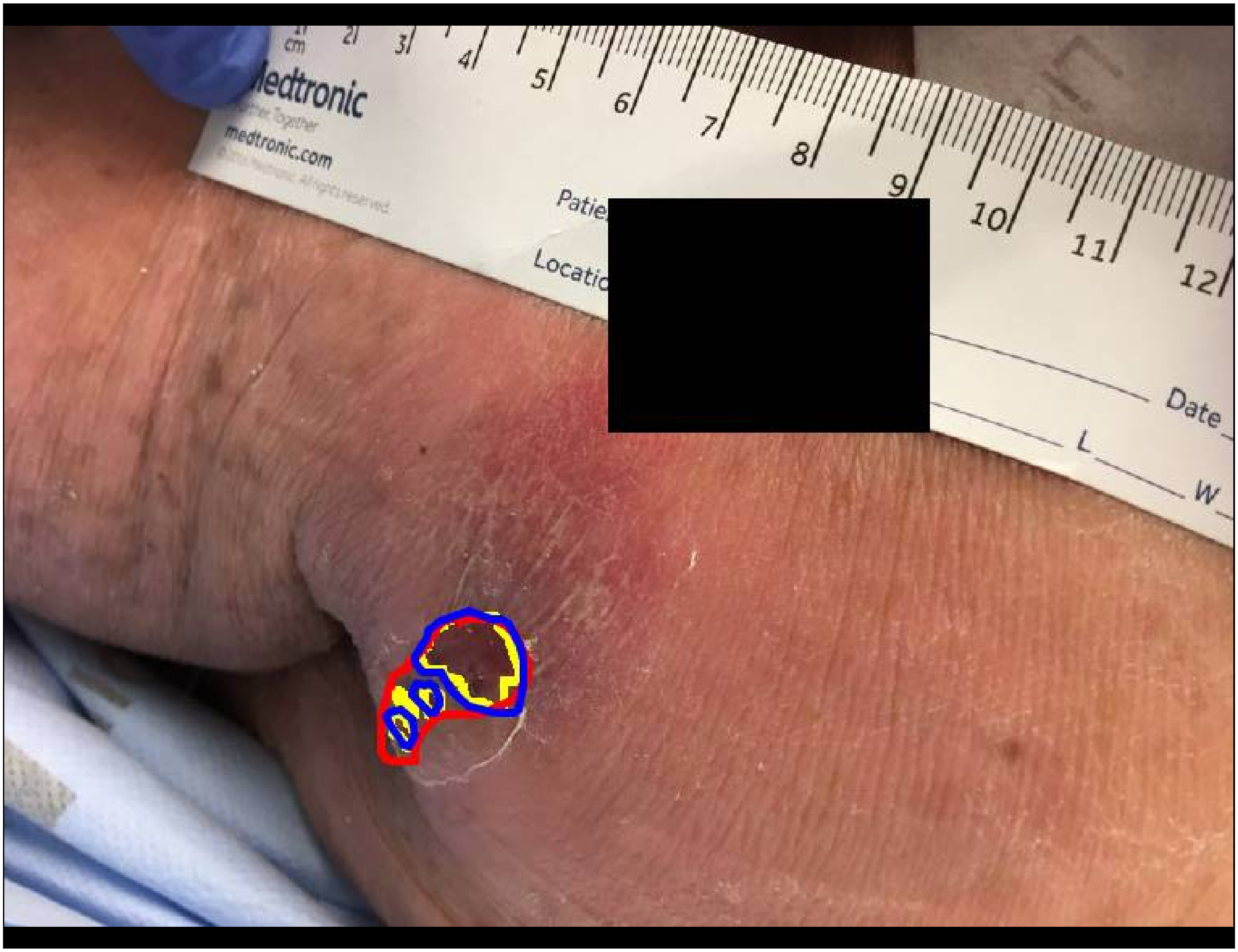

Tracing Data			
Tracer:	Wound Area (px^2)	Ruler Calibration (px/cm)	Wound Area (cm^2)
H1	2678	65.4	0.63
H2	4675	64.6	1.12
AI	3454	61.9	0.90

Tracing Comparisons				
Difference Metric:	Human-Human		Human-AI	
	H1(ref) H2(test)	H2(ref) H1(test)	H1(ref) AI(test)	H2(ref) AI(test)
False Negative Area (%)	1.3	43.5	5.1	31.0
False Positive Area (%)	75.9	0.8	34.1	4.9
|Relative Error (%)|	74.6	42.7	29.0	26.1

Blinded Attending Surgeon Review						
Reviewer	PGT Estimate	H1 meets definition?	H2 meets definition?	AI meets definition?	Which is AI?	Which is most accurate?
1	100	Yes	No	Yes	H1	H1
2	100	Yes	Yes	Yes	H1	H1
3	100	Yes	No	Yes	H1	AI

© 2021 Howell RS et al. *JAMA Network Open.*

Wound EMR Information								
Sequential Number	Gender	Age	Wound Type	Wound Location	Wound Length (cm)	Wound Width (cm)	Wound Depth (cm)	Wound Area (cm^2)
21	F	72	Pressure	Calf/leg	1.5			

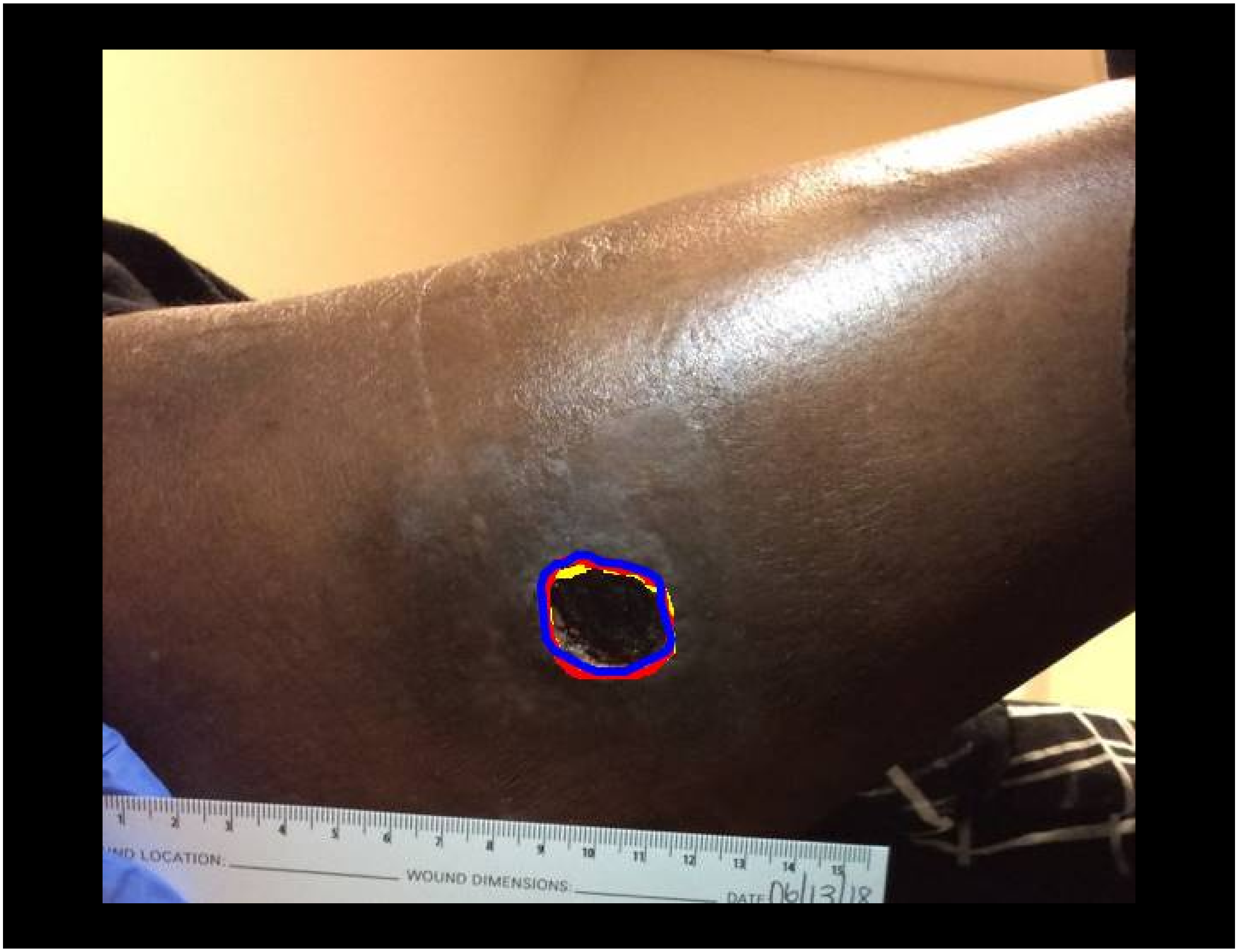

Tracing Data			
Tracer:	Wound Area (px^2)	Ruler Calibration (px/cm)	Wound Area (cm^2)
H1	3782	30.4	4.09
H2	4042	31.1	4.19
AI	3987	31.6	4.00

Tracing Comparisons				
Difference Metric:	Human-Human		Human-AI	
	H1(ref) H2(test)	H2(ref) H1(test)	H1(ref) AI(test)	H2(ref) AI(test)
False Negative Area (%)	1.5	7.8	3.4	6.7
False Positive Area (%)	8.4	1.4	8.8	5.3
|Relative Error (%)|	6.9	6.4	5.4	1.4

Blinded Attending Surgeon Review						
Reviewer	PGT Estimate	H1 meets definition?	H2 meets definition?	AI meets definition?	Which is AI?	Which is most accurate?
1	0	Yes	Yes	Yes	H1	H2
2	0	Yes	Yes	Yes	H2	H2
3	100	Yes	Yes	Yes	H2	H2

© 2021 Howell RS et al. *JAMA Network Open.*

Wound EMR Information								
Sequential Number	Gender	Age	Wound Type	Wound Location	Wound Length (cm)	Wound Width (cm)	Wound Depth (cm)	Wound Area (cm^2)
23	F	46	Abscess	below belly	0.5	0.3	0.2	0.15

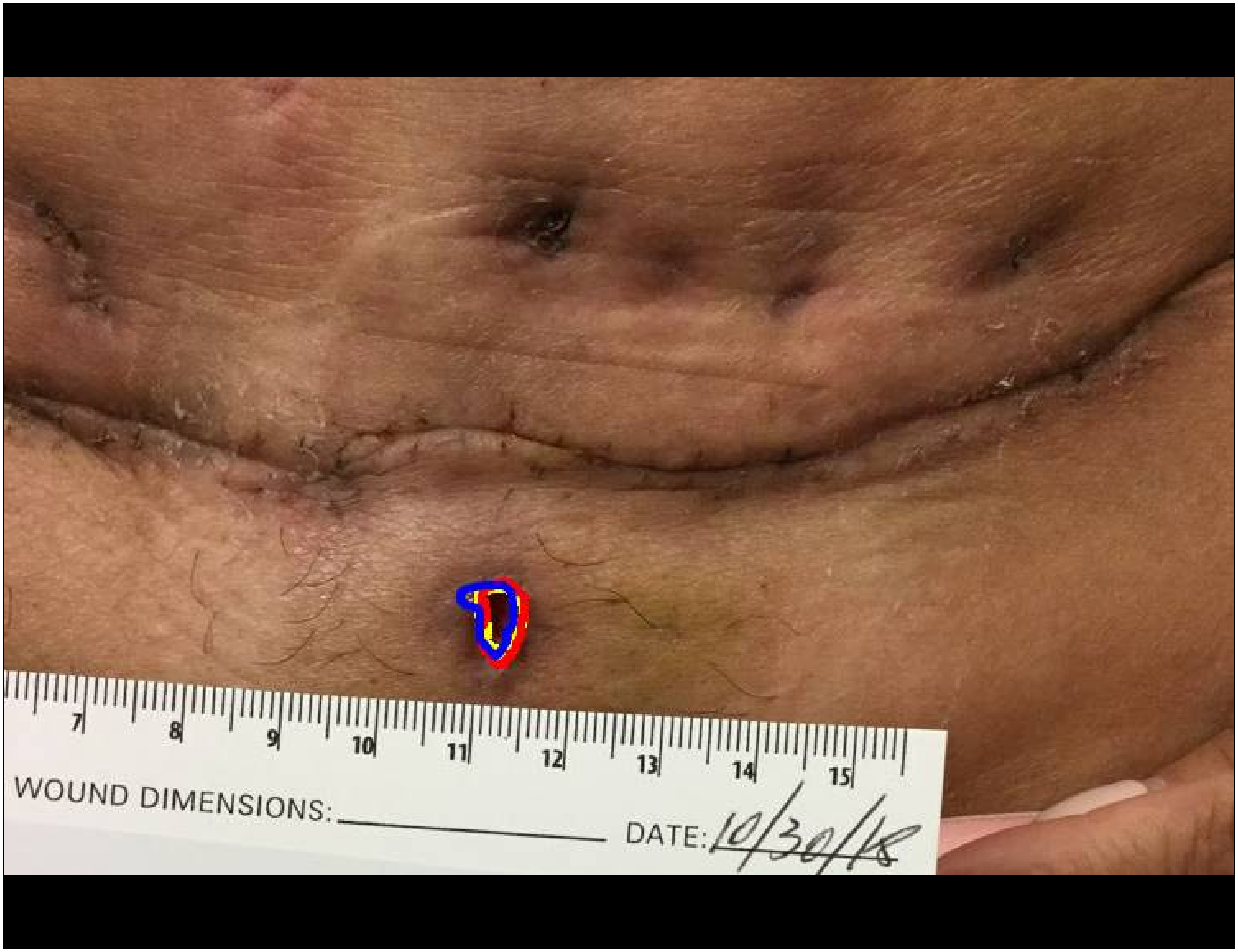

Tracing Data			
Tracer:	Wound Area (px^2)	Ruler Calibration (px/cm)	Wound Area (cm^2)
H1	862	58.7	0.25
H2	997	57.6	0.30
AI	862	65.7	0.20

Tracing Comparisons				
Difference Metric:	Human-Human		Human-AI	
	H1(ref) H2(test)	H2(ref) H1(test)	H1(ref) AI(test)	H2(ref) AI(test)
False Negative Area (%)	8.6	21.0	24.0	33.2
False Positive Area (%)	24.2	7.4	24.0	19.7
|Relative Error (%)|	15.7	13.5	0.0	13.5

Blinded Attending Surgeon Review						
Reviewer	PGT Estimate	H1 meets definition?	H2 meets definition?	AI meets definition?	Which is AI?	Which is most accurate?
1	100	Yes	Yes	Yes	H2	H2
2	100	Yes	Yes	Yes	H2	H2
3	100	Yes	Yes	No	H1	H2

© 2021 Howell RS et al. *JAMA Network Open.*

Wound EMR Information								
Sequential Number	Gender	Age	Wound Type	Wound Location	Wound Length (cm)	Wound Width (cm)	Wound Depth (cm)	Wound Area (cm^2)
24	M	83	Surgical	Chest	3.0			

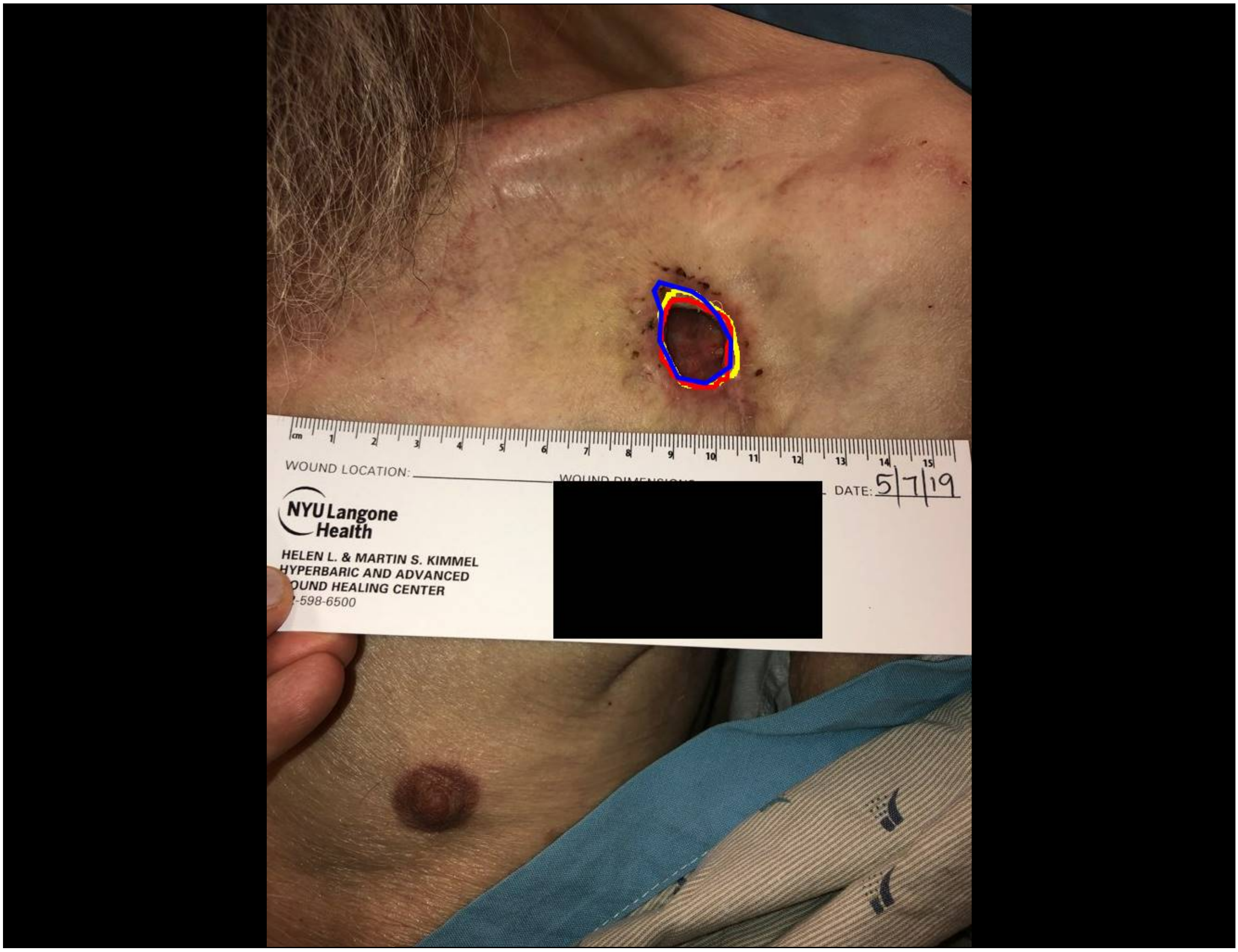

Tracing Data			
Tracer:	Wound Area (px^2)	Ruler Calibration (px/cm)	Wound Area (cm^2)
H1	6508	45.4	3.16
H2	5689	47.0	2.58
AI	6096	45.8	2.90

Tracing Comparisons				
Difference Metric:	Human-Human		Human-AI	
	H1(ref) H2(test)	H2(ref) H1(test)	H1(ref) AI(test)	H2(ref) AI(test)
False Negative Area (%)	15.4	3.2	12.4	10.3
False Positive Area (%)	2.8	17.6	6.1	17.5
|Relative Error (%)|	12.6	14.4	6.3	7.2

Blinded Attending Surgeon Review						
Reviewer	PGT Estimate	H1 meets definition?	H2 meets definition?	AI meets definition?	Which is AI?	Which is most accurate?
1	90	No	No	No	H2	H1
2	80	Yes	Yes	Yes	AI	H2
3	100	No	Yes	No	AI	H1

© 2021 Howell RS et al. *JAMA Network Open.*

Wound EMR Information								
Sequential Number	Gender	Age	Wound Type	Wound Location	Wound Length (cm)	Wound Width (cm)	Wound Depth (cm)	Wound Area (cm^2)
25	M	52	Surgical	Foot-Dorsum				

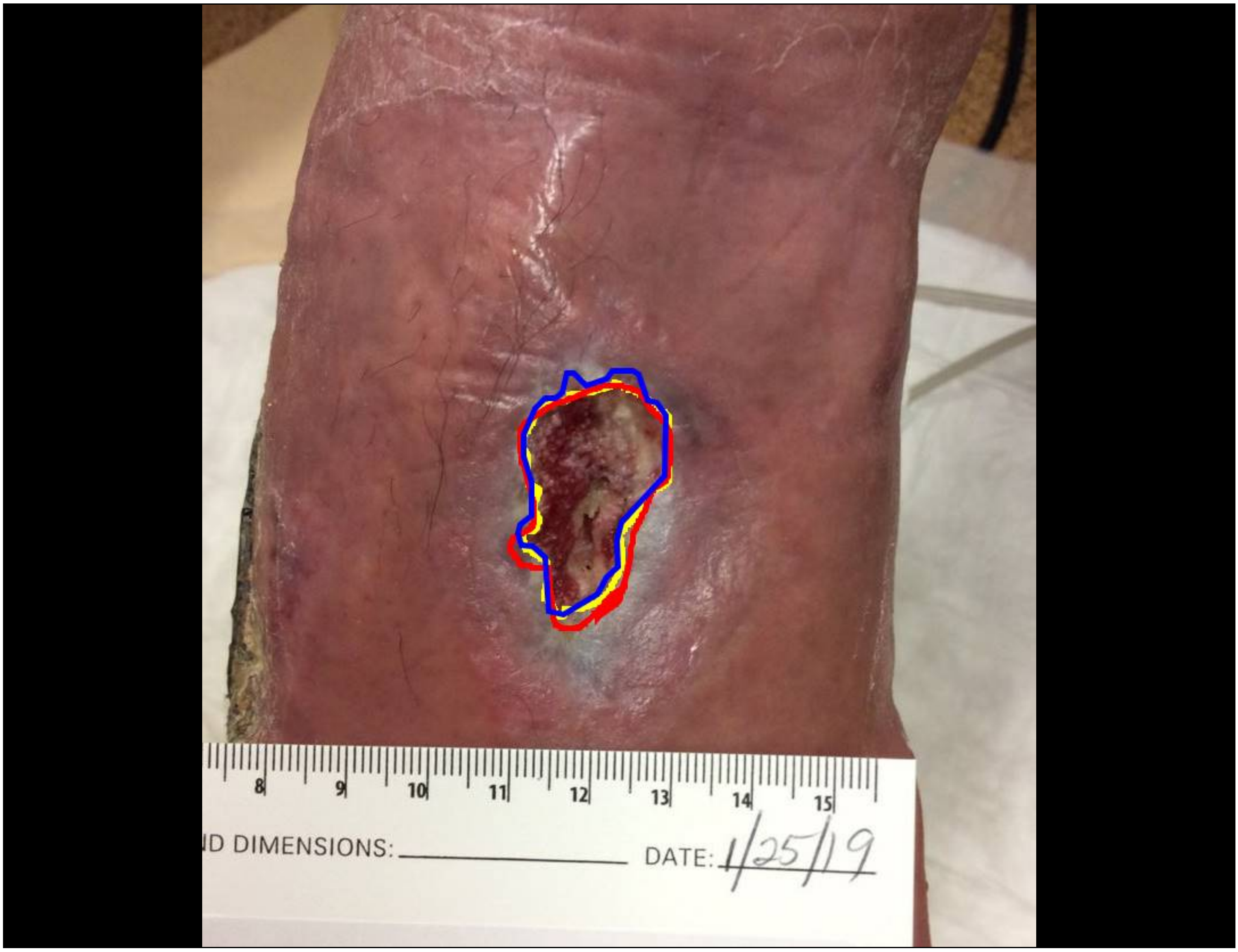

Tracing Data			
Tracer:	Wound Area (px^2)	Ruler Calibration (px/cm)	Wound Area (cm^2)
H1	17650	71.5	3.45
H2	19558	72.0	3.77
AI	18197	72.1	3.50

Tracing Comparisons				
Difference Metric:	Human-Human		Human-AI	
	H1(ref) H2(test)	H2(ref) H1(test)	H1(ref) AI(test)	H2(ref) AI(test)
False Negative Area (%)	2.2	11.8	5.0	13.9
False Positive Area (%)	13.1	2.0	8.1	6.9
|Relative Error (%)|	10.8	9.8	3.1	7.0

Blinded Attending Surgeon Review						
Reviewer	PGT Estimate	H1 meets definition?	H2 meets definition?	AI meets definition?	Which is AI?	Which is most accurate?
**1**	60	Yes	Yes	Yes	H1	H1
**2**	80	Yes	Yes	Yes	H2	H2
**3**	70	Yes	No	Yes	H1	H2

© 2021 Howell RS et al. *JAMA Network Open.*

Wound EMR Information								
Sequential Number	Gender	Age	Wound Type	Wound Location	Wound Length (cm)	Wound Width (cm)	Wound Depth (cm)	Wound Area (cm^2)
26	M	27	Pressure	Ischium	2.5	1.0	7.5	2.50

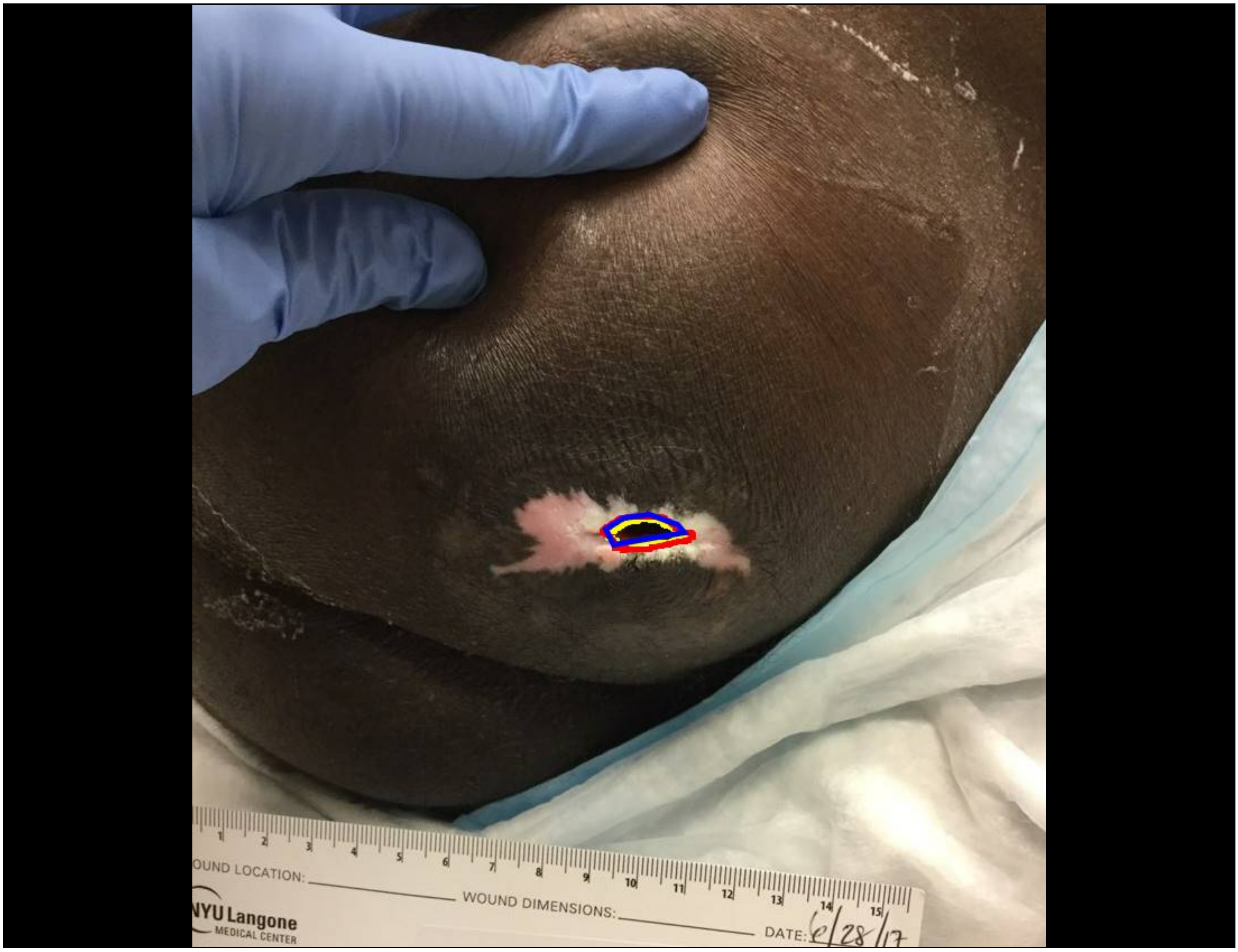

Tracing Data			
Tracer:	Wound Area (px^2)	Ruler Calibration (px/cm)	Wound Area (cm^2)
H1	1086	42.4	0.60
H2	1641	50.9	0.63
AI	1131	40.2	0.70

Tracing Comparisons				
Difference Metric:	Human-Human		Human-AI	
	H1(ref) H2(test)	H2(ref) H1(test)	H1(ref) AI(test)	H2(ref) AI(test)
False Negative Area (%)	0.4	34.1	19.7	33.5
False Positive Area (%)	51.5	0.2	23.8	2.4
|Relative Error (%)|	51.1	33.8	4.1	31.1

Blinded Attending Surgeon Review						
Reviewer	PGT Estimate	H1 meets definition?	H2 meets definition?	AI meets definition?	Which is AI?	Which is most accurate?
1	100	Yes	Yes	Yes	H1	H1
2	50	Yes	Yes	Yes	H2	H2
3	100	Yes	Yes	No	H2	H1

© 2021 Howell RS et al. *JAMA Network Open.*

Wound EMR Information								
Sequential Number	Gender	Age	Wound Type	Wound Location	Wound Length (cm)	Wound Width (cm)	Wound Depth (cm)	Wound Area (cm^2)
27	F	33	Surgical	Abdomen	10.0	8.0	3.0	80.00

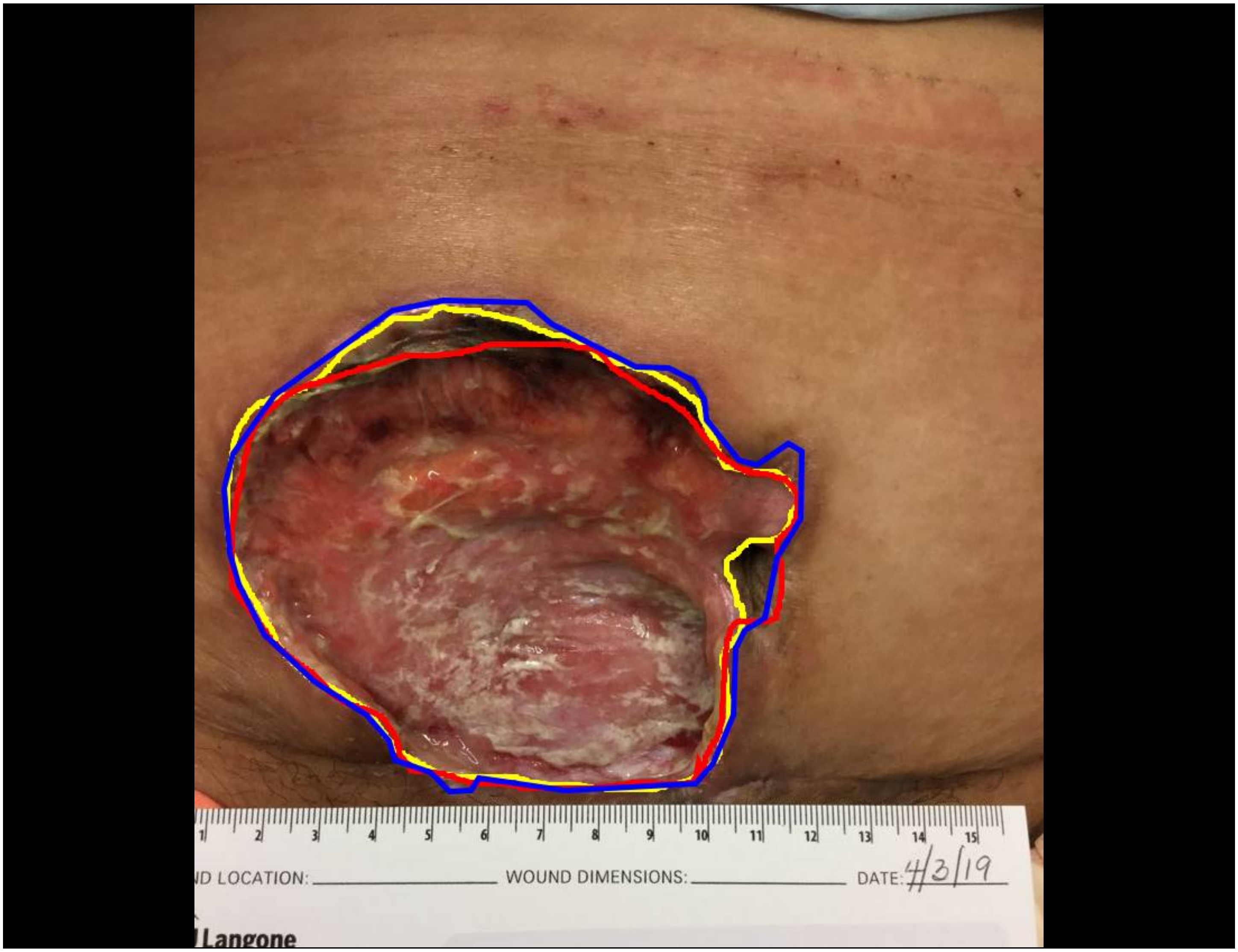

Tracing Data			
Tracer:	Wound Area (px^2)	Ruler Calibration (px/cm)	Wound Area (cm^2)
H1	154896	49.5	63.26
H2	149623	45.0	73.88
AI	164510	49.0	68.60

Tracing Comparisons				
Difference Metric:	Human-Human		Human-AI	
	H1(ref) H2(test)	H2(ref) H1(test)	H1(ref) AI(test)	H2(ref) AI(test)
False Negative Area (%)	6.4	3.1	0.4	0.5
False Positive Area (%)	3.0	6.6	6.6	10.5
|Relative Error (%)|	3.4	3.5	6.2	9.9

Blinded Attending Surgeon Review						
Reviewer	PGT Estimate	H1 meets definition?	H2 meets definition?	AI meets definition?	Which is AI?	Which is most accurate?
1	70	Yes	No	No	H1	H2
2	80	Yes	Yes	Yes	H2	H2
3	70	Yes	No	No	H2	H2

© 2021 Howell RS et al. *JAMA Network Open.*

Wound EMR Information								
Sequential Number	Gender	Age	Wound Type	Wound Location	Wound Length (cm)	Wound Width (cm)	Wound Depth (cm)	Wound Area (cm^2)
29	M	92	Unknown	Chest	2.5			

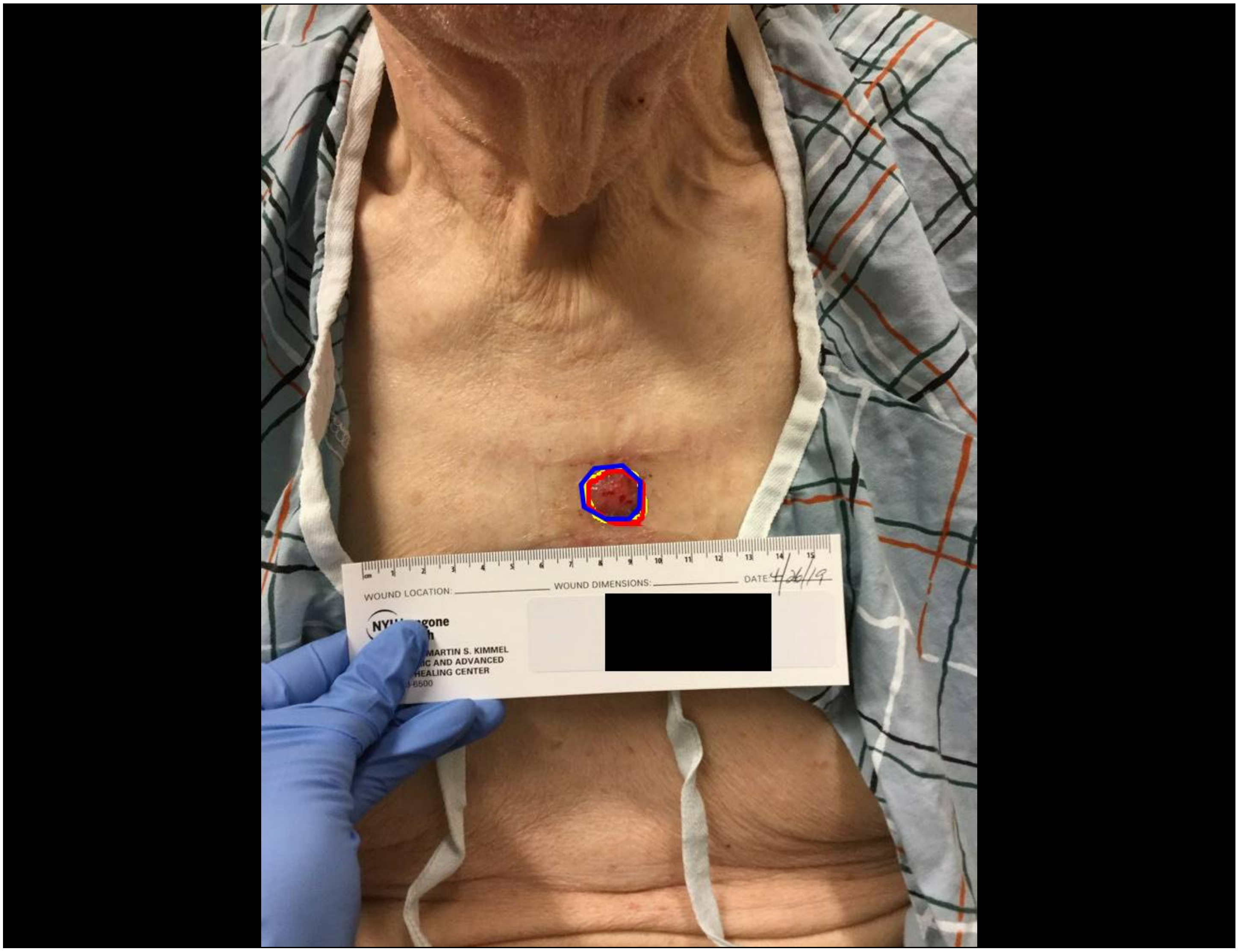

Tracing Data			
Tracer:	Wound Area (px^2)	Ruler Calibration (px/cm)	Wound Area (cm^2)
H1	2803	32.0	2.73
H2	2670	42.8	1.46
AI	2915	37.3	2.10

Tracing Comparisons				
Difference Metric:	Human-Human		Human-AI	
	H1(ref) H2(test)	H2(ref) H1(test)	H1(ref) AI(test)	H2(ref) AI(test)
False Negative Area (%)	8.8	4.3	9.6	11.7
False Positive Area (%)	4.1	9.3	13.6	20.9
|Relative Error (%)|	4.7	5.0	4.0	9.2

Blinded Attending Surgeon Review						
Reviewer	PGT Estimate	H1 meets definition?	H2 meets definition?	AI meets definition?	Which is AI?	Which is most accurate?
1	100	Yes	Yes	Yes	H2	H1
2	100	Yes	Yes	Yes	H2	H2
3	100	Yes	Yes	Yes	H1	H2

© 2021 Howell RS et al. *JAMA Network Open.*

Wound EMR Information								
**Sequential Number**	**Gender**	**Age**	**Wound Type**	**Wound Location**	**Wound Length (cm)**	**Wound Width (cm)**	**Wound Depth (cm)**	**Wound Area (cm^2)**
30	F	72	Traumatic	Leg-R Low Lateral	5.0	4.0		20.00

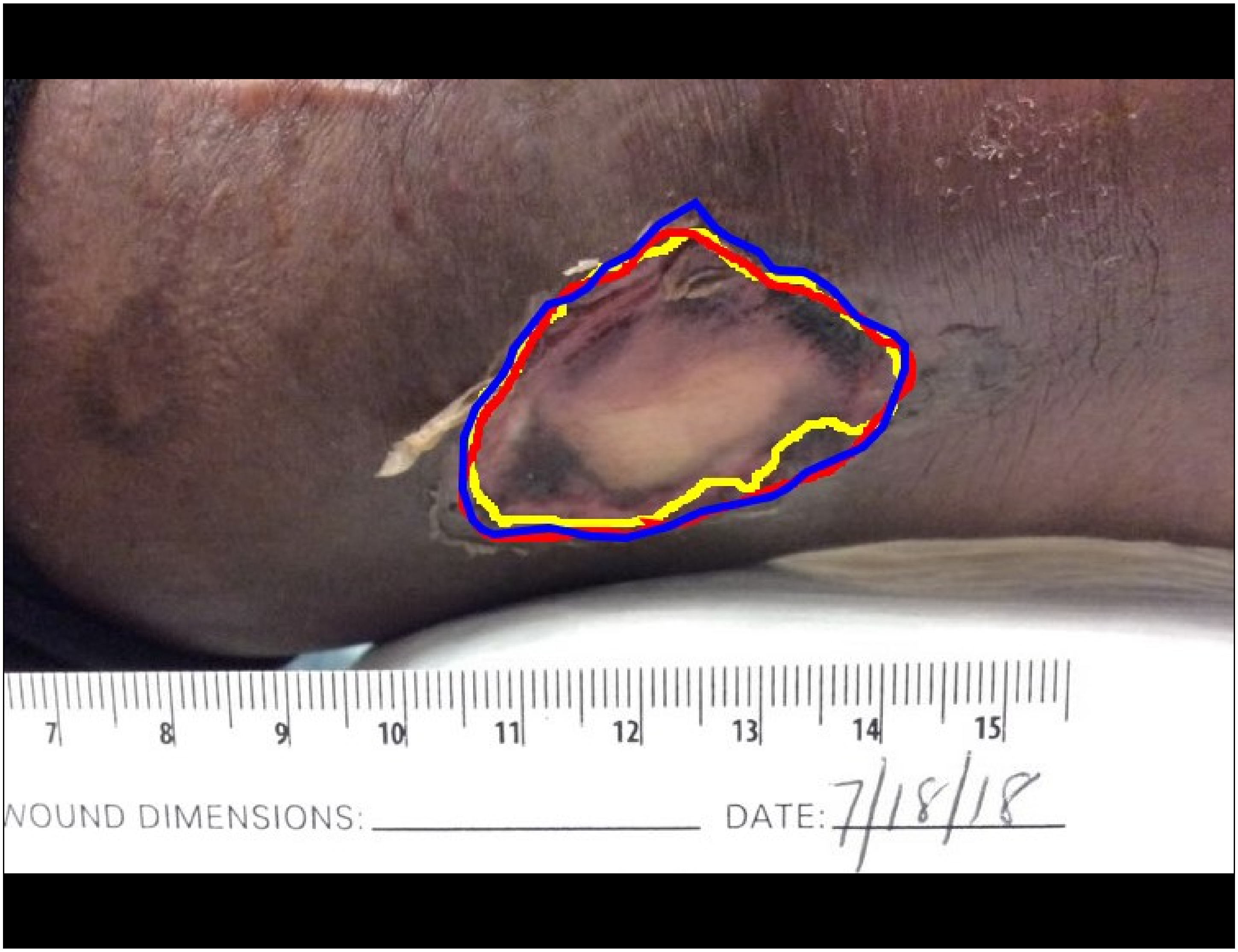

Tracing Data			
**Tracer:**	**Wound Area (px^2)**	**Ruler Calibration (px/cm)**	**Wound Area (cm^2)**
**H1**	29892	72.5	5.69
**H2**	33144	74.3	6.00
**AI**	35297	75.5	6.20

Tracing Comparisons				
**Difference Metric:**	**Human-Human**		**Human-AI**	
	**H1(ref) H2(test)**	**H2(ref) H1(test)**	**H1(ref) AI(test)**	**H2(ref) AI(test)**
**False Negative Area (%)**	2.6	12.2	0.3	1.5
**False Positive Area (%)**	13.5	2.4	18.4	8.0
**|Relative Error (%)|**	10.9	9.8	18.1	6.5

Blinded Attending Surgeon Review						
**Reviewer**	**PGT Estimate**	**H1 meets definition?**	**H2 meets definition?**	**AI meets definition?**	**Which is AI?**	**Which is most accurate?**
**1**	100	No	No	No	H2	H2
**2**	10	Yes	Yes	Yes	H2	H2
**3**	10	Yes	Yes	Yes	H1	AI

© 2021 Howell RS et al. *JAMA Network Open.*

Wound EMR Information								
Sequential Number	Gender	Age	Wound Type	Wound Location	Wound Length (cm)	Wound Width (cm)	Wound Depth (cm)	Wound Area (cm^2)
31	F	51	Surgical	Back	4.0	3.5		14.00

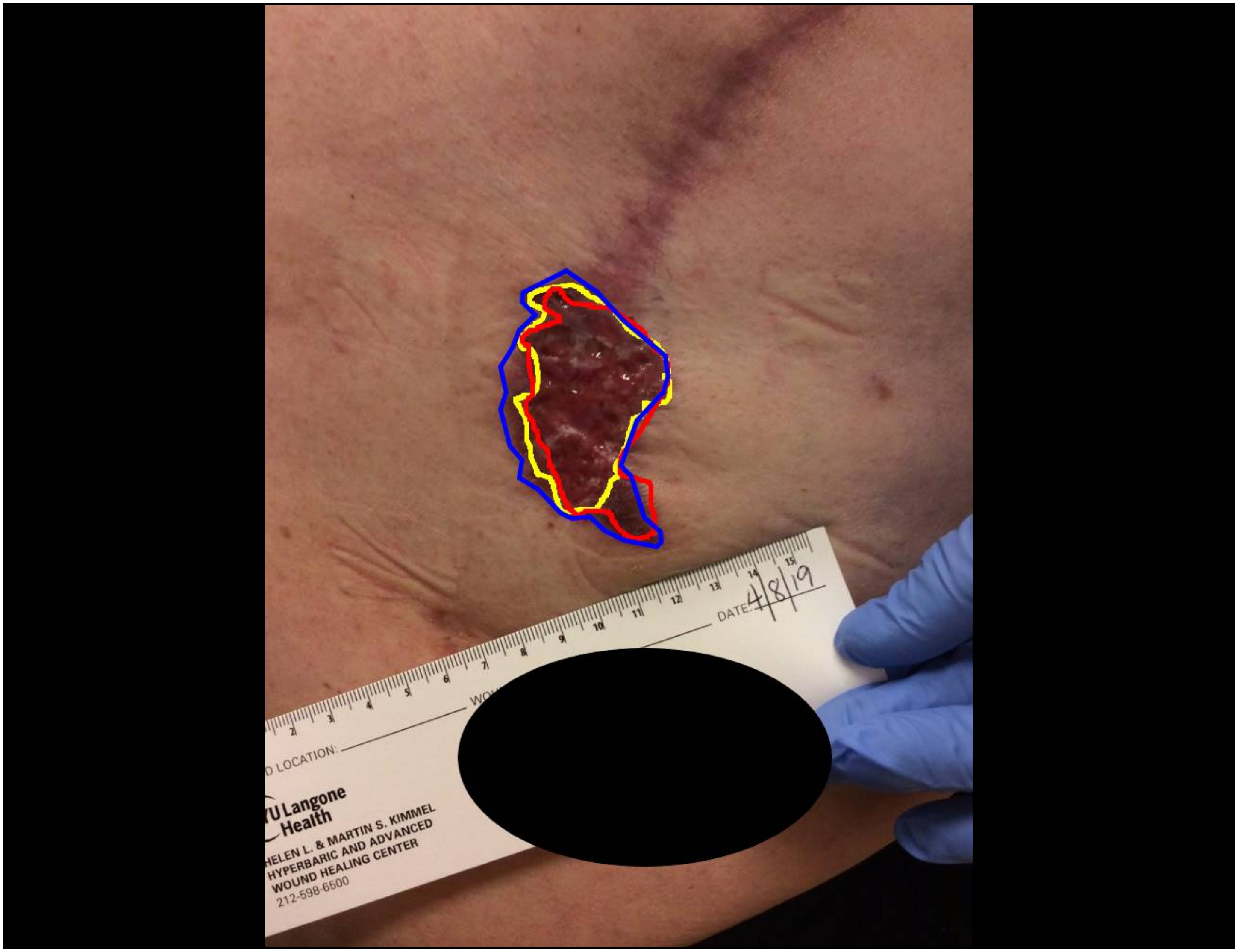

Tracing Data			
Tracer:	Wound Area (px^2)	Ruler Calibration (px/cm)	Wound Area (cm^2)
H1	24480	43.6	12.85
H2	25626	44.0	13.26
AI	33132	42.7	18.20

Tracing Comparisons				
Difference Metric:	Human-Human		Human-AI	
	H1(ref) H2(test)	H2(ref) H1(test)	H1(ref) AI(test)	H2(ref) AI(test)
False Negative Area (%)	11.5	15.4	1.0	4.1
False Positive Area (%)	16.2	11.0	36.3	33.3
|Relative Error (%)|	4.7	4.5	35.3	29.3

Blinded Attending Surgeon Review						
Reviewer	PGT Estimate	H1 meets definition?	H2 meets definition?	AI meets definition?	Which is AI?	Which is most accurate?
1	100	Yes	Yes	Yes	H1	H1
2	100	Yes	Yes	Yes	AI	H2
3	100	Yes	No	No	H1	H2

© 2021 Howell RS et al. *JAMA Network Open.*

Wound EMR Information								
Sequential Number	Gender	Age	Wound Type	Wound Location	Wound Length (cm)	Wound Width (cm)	Wound Depth (cm)	Wound Area (cm^2)
32	F	34	Trauma	Left lower leg				

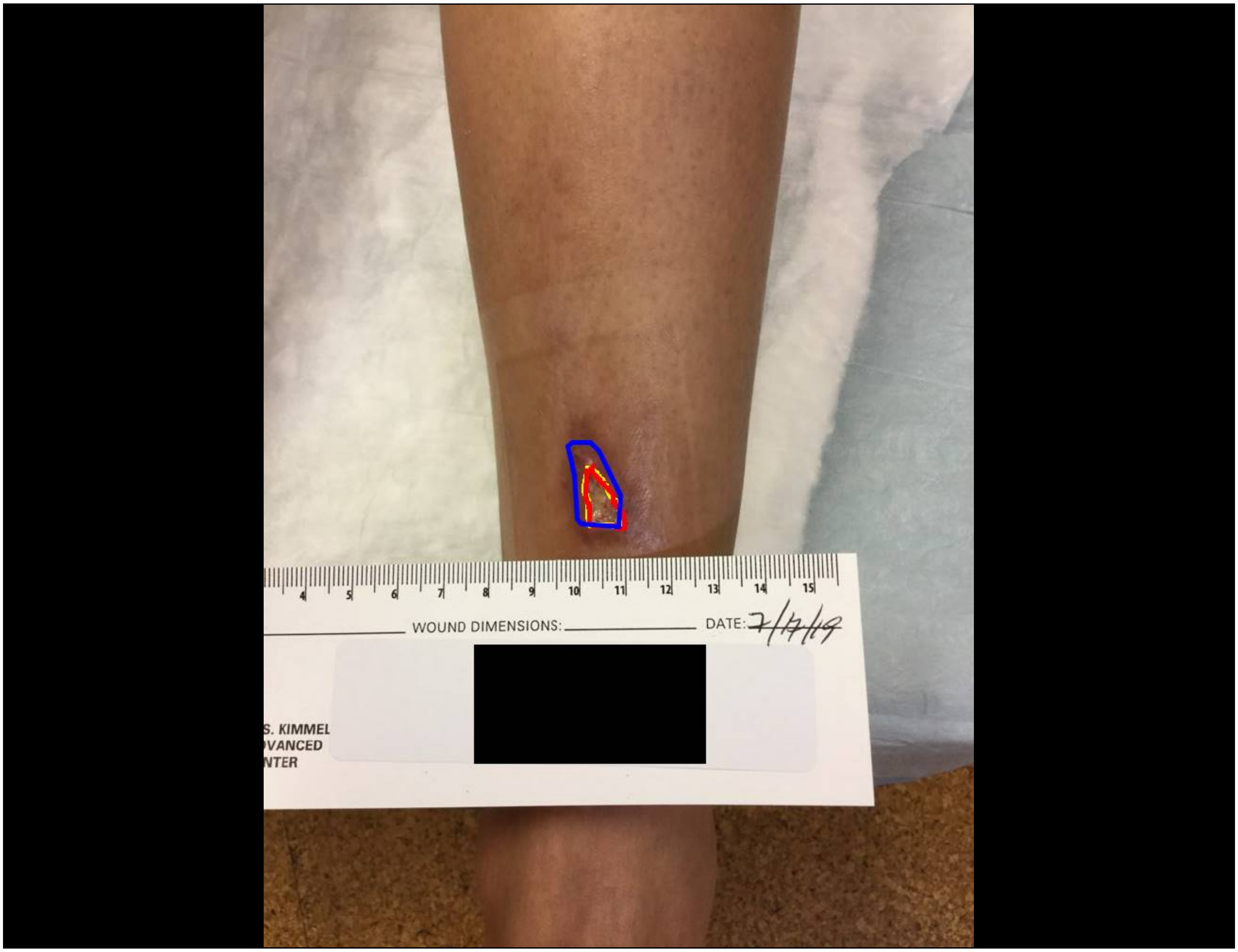

Tracing Data			
Tracer:	Wound Area (px^2)	Ruler Calibration (px/cm)	Wound Area (cm^2)
H1	1547	48.4	0.66
H2	1524	49.9	0.61
AI	3547	54.4	1.20

Tracing Comparisons				
Difference Metric:	Human-Human		Human-AI	
	H1(ref) H2(test)	H2(ref) H1(test)	H1(ref) AI(test)	H2(ref) AI(test)
False Negative Area (%)	8.1	6.7	1.7	4.8
False Positive Area (%)	6.6	8.2	131.0	137.5
|Relative Error (%)|	1.5	1.5	129.3	132.7

Blinded Attending Surgeon Review						
Reviewer	PGT Estimate	H1 meets definition?	H2 meets definition?	AI meets definition?	Which is AI?	Which is most accurate?
1	0	Yes	Yes	No	H2	H1
2	70	Yes	Yes	Yes	H1	H1
3	10	Yes	Yes	No	H2	H2

© 2021 Howell RS et al. *JAMA Network Open.*

Wound EMR Information								
Sequential Number	Gender	Age	Wound Type	Wound Location	Wound Length (cm)	Wound Width (cm)	Wound Depth (cm)	Wound Area (cm^2)
33	F	72	Pressure	Heel	8.0	6.0	1.5	48.00

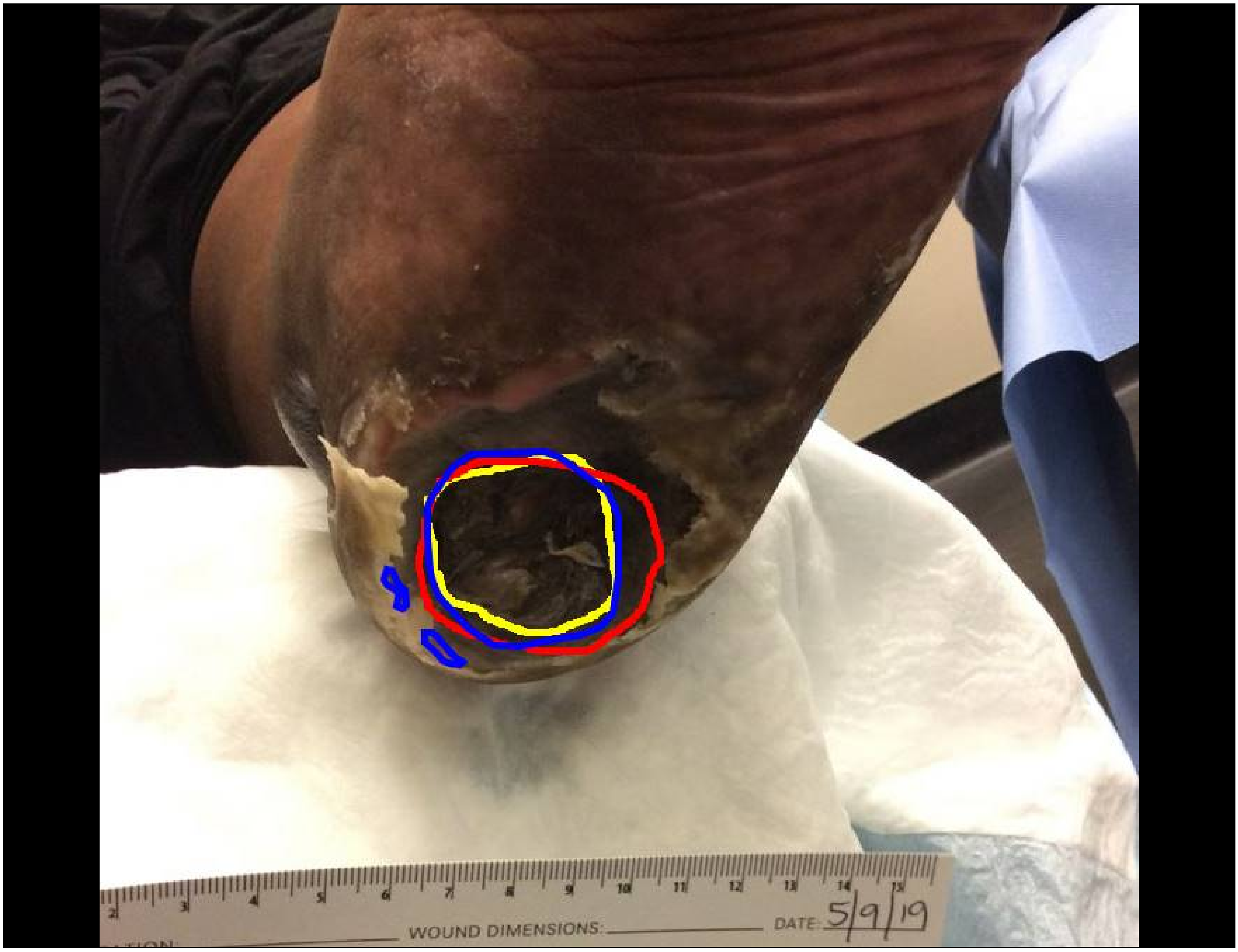

Tracing Data			
Tracer:	Wound Area (px^2)	Ruler Calibration (px/cm)	Wound Area (cm^2)
H1	13352	42.1	7.52
H2	18956	42.9	10.31
AI	16175	39.4	10.40

Tracing Comparisons				
Difference Metric:	Human-Human		Human-AI	
	H1(ref) H2(test)	H2(ref) H1(test)	H1(ref) AI(test)	H2(ref) AI(test)
False Negative Area (%)	1.6	30.7	1.1	19.6
False Positive Area (%)	43.6	1.1	22.3	5.0
|Relative Error (%)|	42.0	29.6	21.1	14.7

Blinded Attending Surgeon Review						
Reviewer	PGT Estimate	H1 meets definition?	H2 meets definition?	AI meets definition?	Which is AI?	Which is most accurate?
1	0	No	No	No	H2	H1
2	10	No	Yes	No	H2	H2
3	0	Yes	No	Yes	AI	H2

© 2021 Howell RS et al. *JAMA Network Open.*

Wound EMR Information								
Sequential Number	Gender	Age	Wound Type	Wound Location	Wound Length (cm)	Wound Width (cm)	Wound Depth (cm)	Wound Area (cm^2)
34	F	52	Dermatologi	Thigh-Hamstrings				

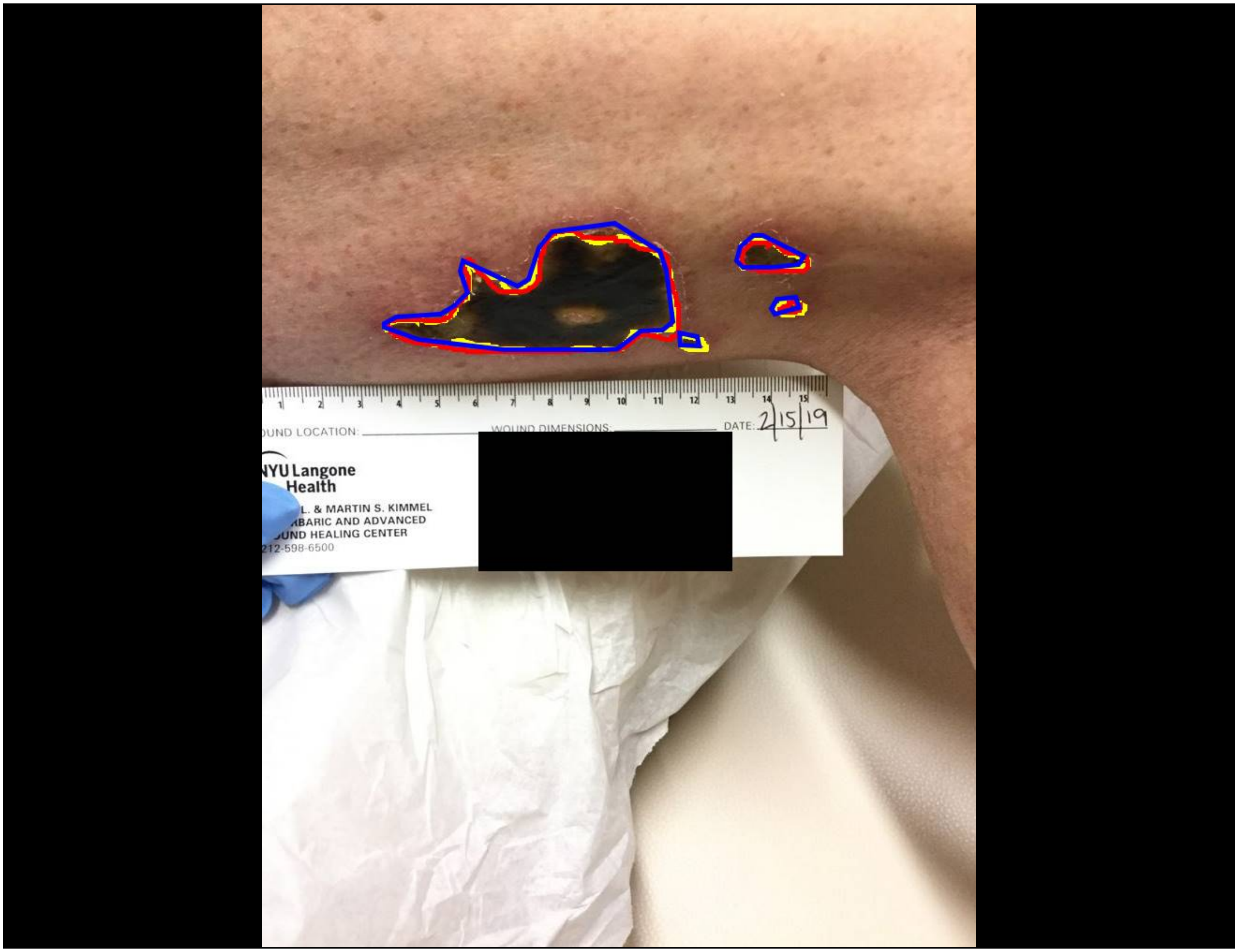

Tracing Data			
Tracer:	Wound Area (px^2)	Ruler Calibration (px/cm)	Wound Area (cm^2)
H1	25279	39.1	16.53
H2	25802	40.2	15.98
AI	27146	42.8	14.80

Tracing Comparisons				
Difference Metric:	Human-Human		Human-AI	
	H1(ref) H2(test)	H2(ref) H1(test)	H1(ref) AI(test)	H2(ref) AI(test)
False Negative Area (%)	5.1	7.0	3.9	6.9
False Positive Area (%)	7.2	5.0	11.2	12.1
|Relative Error (%)|	2.1	2.0	7.4	5.2

Blinded Attending Surgeon Review						
Reviewer	PGT Estimate	H1 meets definition?	H2 meets definition?	AI meets definition?	Which is AI?	Which is most accurate?
1	0	Yes	Yes	Yes	H2	H1
2	10	Yes	Yes	Yes	AI	AI
3	0	Yes	Yes	Yes	H2	AI

© 2021 Howell RS et al. *JAMA Network Open.*

Wound EMR Information								
Sequential Number	Gender	Age	Wound Type	Wound Location	Wound Length (cm)	Wound Width (cm)	Wound Depth (cm)	Wound Area (cm^2)
35	F	36	Abscess	Right Abdomen				

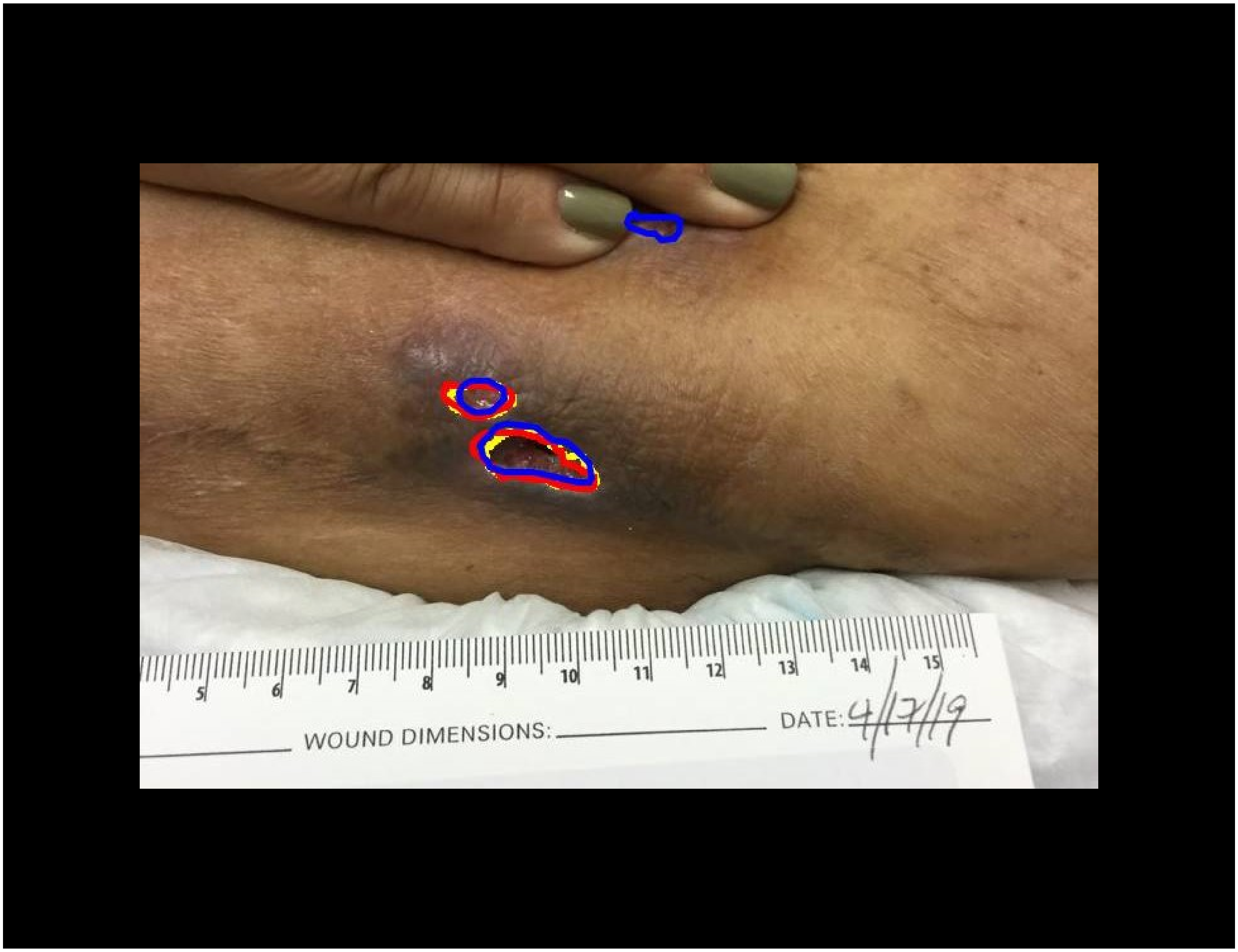

Tracing Data			
Tracer:	Wound Area (px^2)	Ruler Calibration (px/cm)	Wound Area (cm^2)
H1	3242	56.7	1.01
H2	3440	57.2	1.05
AI	3858	56.7	1.20

Tracing Comparisons				
Difference Metric:	Human-Human		Human-AI	
	H1(ref) H2(test)	H2(ref) H1(test)	H1(ref) AI(test)	H2(ref) AI(test)
False Negative Area (%)	9.7	14.9	15.7	23.5
False Positive Area (%)	15.8	9.1	34.7	35.6
|Relative Error (%)|	6.1	5.8	19.0	12.2

Blinded Attending Surgeon Review						
Reviewer	PGT Estimate	H1 meets definition?	H2 meets definition?	AI meets definition?	Which is AI?	Which is most accurate?
1	80	Yes	Yes	Yes	H2	H1
2	100	Yes	Yes	Yes	H2	H2
3	90	Yes	Yes	Yes	H2	H2

© 2021 Howell RS et al. *JAMA Network Open.*

Wound EMR Information								
Sequential Number	Gender	Age	Wound Type	Wound Location	Wound Length (cm)	Wound Width (cm)	Wound Depth (cm)	Wound Area (cm²)
36	F	79	Tumor/Canc	Breast	6.0	6.0		36.00

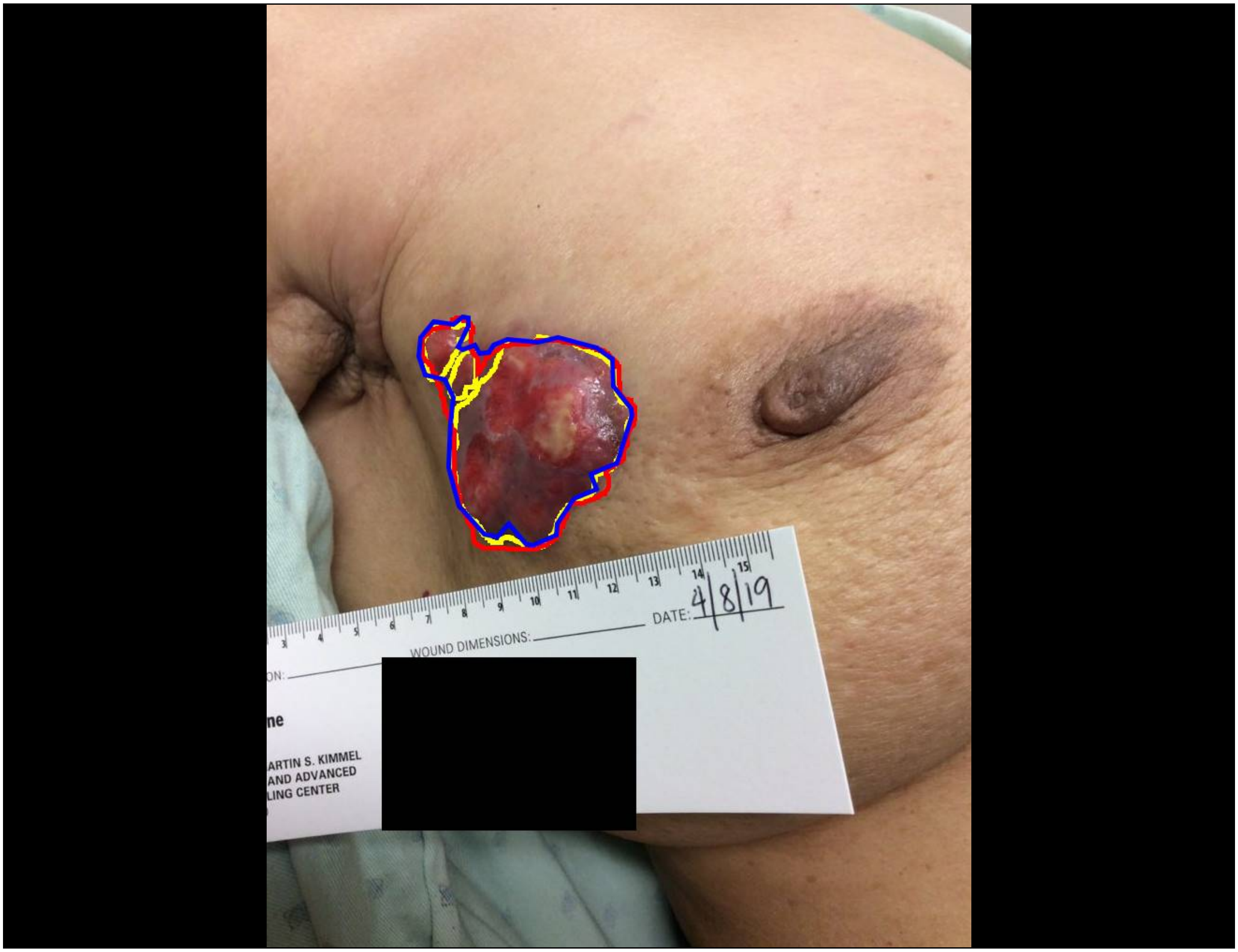

Tracing Data			
Tracer:	Wound Area (px²)	Ruler Calibration (px/cm)	Wound Area (cm²)
H1	33008	41.0	19.65
H2	36635	42.5	20.29
AI	34916	53.3	12.30

Tracing Comparisons				
Difference Metric:	Human-Human		Human-AI	
	H1(ref) H2(test)	H2(ref) H1(test)	H1(ref) AI(test)	H2(ref) AI(test)
False Negative Area (%)	1.0	10.8	2.1	7.9
False Positive Area (%)	11.9	0.9	7.9	3.2
|Relative Error (%)|	11.0	9.9	5.8	4.7

Blinded Attending Surgeon Review						
Reviewer	PGT Estimate	H1 meets definition?	H2 meets definition?	AI meets definition?	Which is AI?	Which is most accurate?
1	90	Yes	No	No	H2	AI
2	90	Yes	Yes	Yes	AI	AI
3	80	Yes	Yes	Yes	AI	H2

© 2021 Howell RS et al. *JAMA Network Open.*

Wound EMR Information								
Sequential Number	Gender	Age	Wound Type	Wound Location	Wound Length (cm)	Wound Width (cm)	Wound Depth (cm)	Wound Area (cm^2)
37	F	67	Surgical	Foot-Dorsum	0.5	0.1		0.05

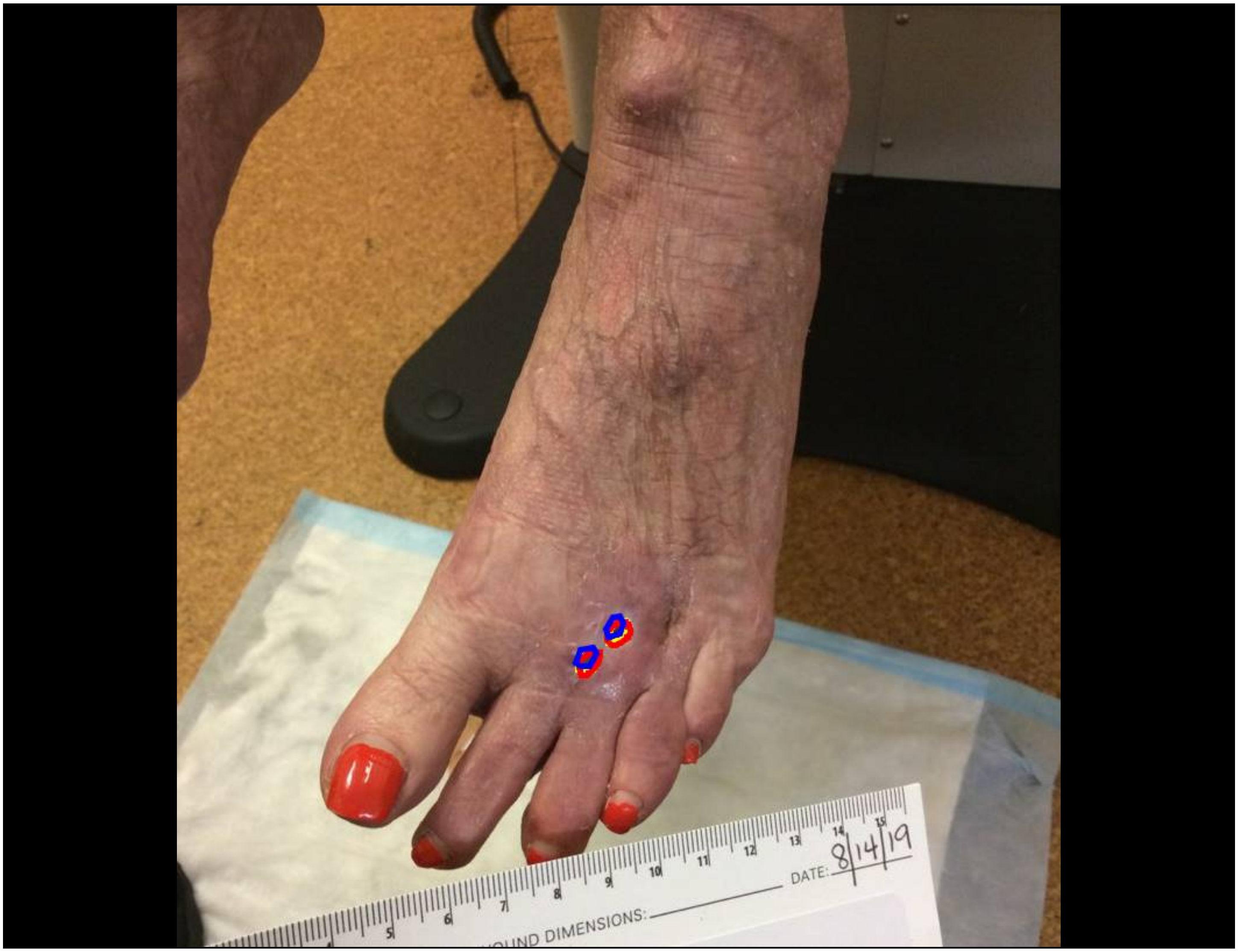

Tracing Data			
Tracer:	Wound Area (px^2)	Ruler Calibration (px/cm)	Wound Area (cm^2)
H1	485	43.9	0.25
H2	535	48.0	0.23
AI	376	35.4	0.30

Tracing Comparisons				
Difference Metric:	Human-Human		Human-AI	
	H1(ref) H2(test)	H2(ref) H1(test)	H1(ref) AI(test)	H2(ref) AI(test)
False Negative Area (%)	10.9	19.3	45.6	57.2
False Positive Area (%)	21.2	9.9	23.1	27.5
|Relative Error (%)|	10.3	9.3	22.5	29.7

Blinded Attending Surgeon Review						
Reviewer	PGT Estimate	H1 meets definition?	H2 meets definition?	AI meets definition?	Which is AI?	Which is most accurate?
1	0	Yes	Yes	Yes	H2	H1
2	50	Yes	Yes	Yes	H2	AI
3	100	Yes	Yes	No	H2	H1

© 2021 Howell RS et al. *JAMA Network Open*.

Wound EMR Information								
Sequential Number	Gender	Age	Wound Type	Wound Location	Wound Length (cm)	Wound Width (cm)	Wound Depth (cm)	Wound Area (cm^2)
38	M	32	Burn	Right medial	10.2	6.5	0.3	66.30

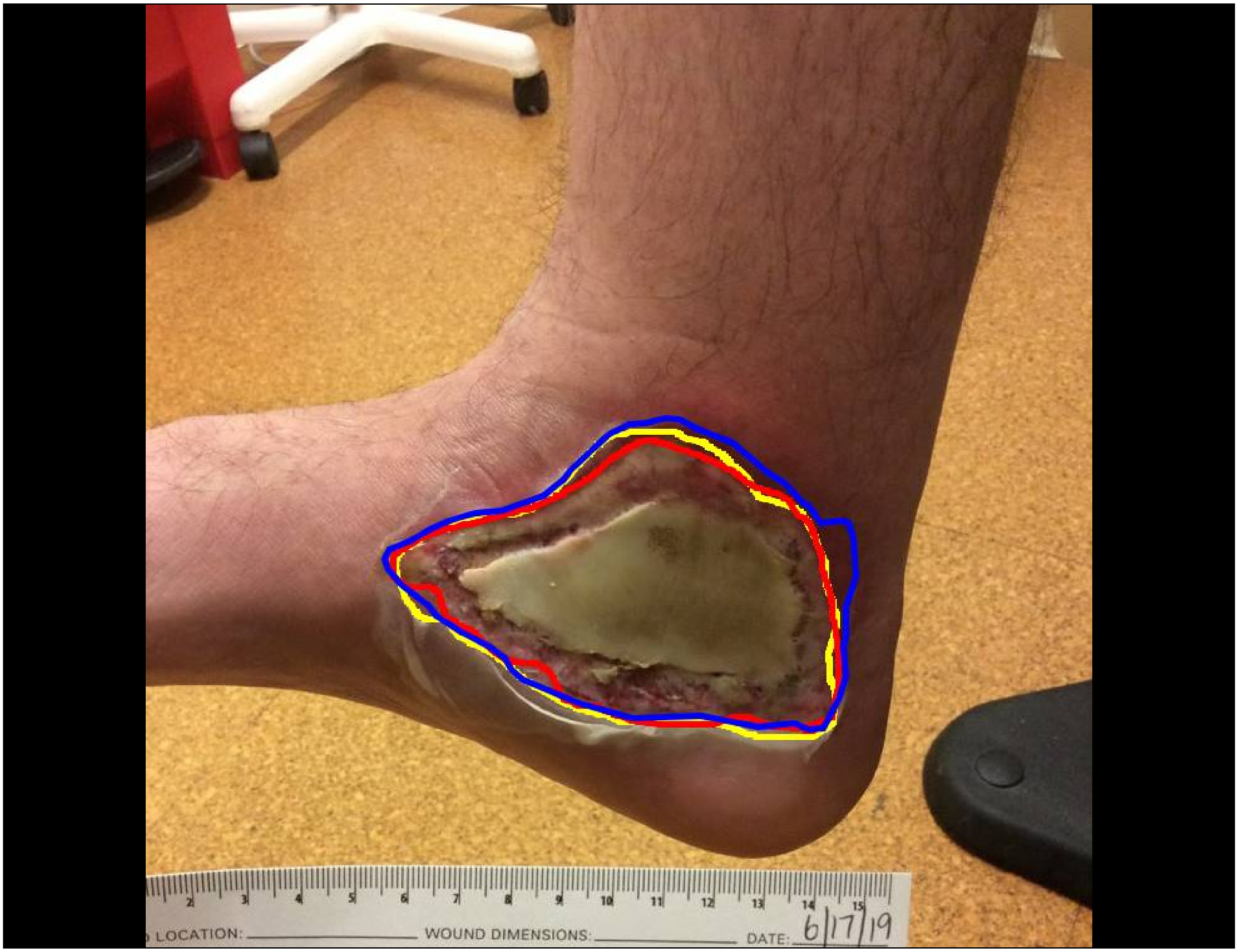

Tracing Data			
Tracer:	Wound Area (px^2)	Ruler Calibration (px/cm)	Wound Area (cm^2)
H1	57592	40.3	35.54
H2	53704	67.2	11.88
AI	61186	45.0	30.20

Tracing Comparisons				
Difference Metric:	Human-Human		Human-AI	
	H1(ref) H2(test)	H2(ref) H1(test)	H1(ref) AI(test)	H2(ref) AI(test)
False Negative Area (%)	7.9	1.2	2.6	0.8
False Positive Area (%)	1.1	8.4	8.9	14.7
|Relative Error (%)|	6.8	7.2	6.2	13.9

Blinded Attending Surgeon Review						
Reviewer	PGT Estimate	H1 meets definition?	H2 meets definition?	AI meets definition?	Which is AI?	Which is most accurate?
1	10	Yes	Yes	No	H1	H2
2	30	Yes	Yes	Yes	H2	H2
3	10	Yes	Yes	Yes	H2	H2

© 2021 Howell RS et al. *JAMA Network Open.*

Wound EMR Information								
Sequential Number	Gender	Age	Wound Type	Wound Location	Wound Length (cm)	Wound Width (cm)	Wound Depth (cm)	Wound Area (cm^2)
39	F	53	Surgical	Ankle				

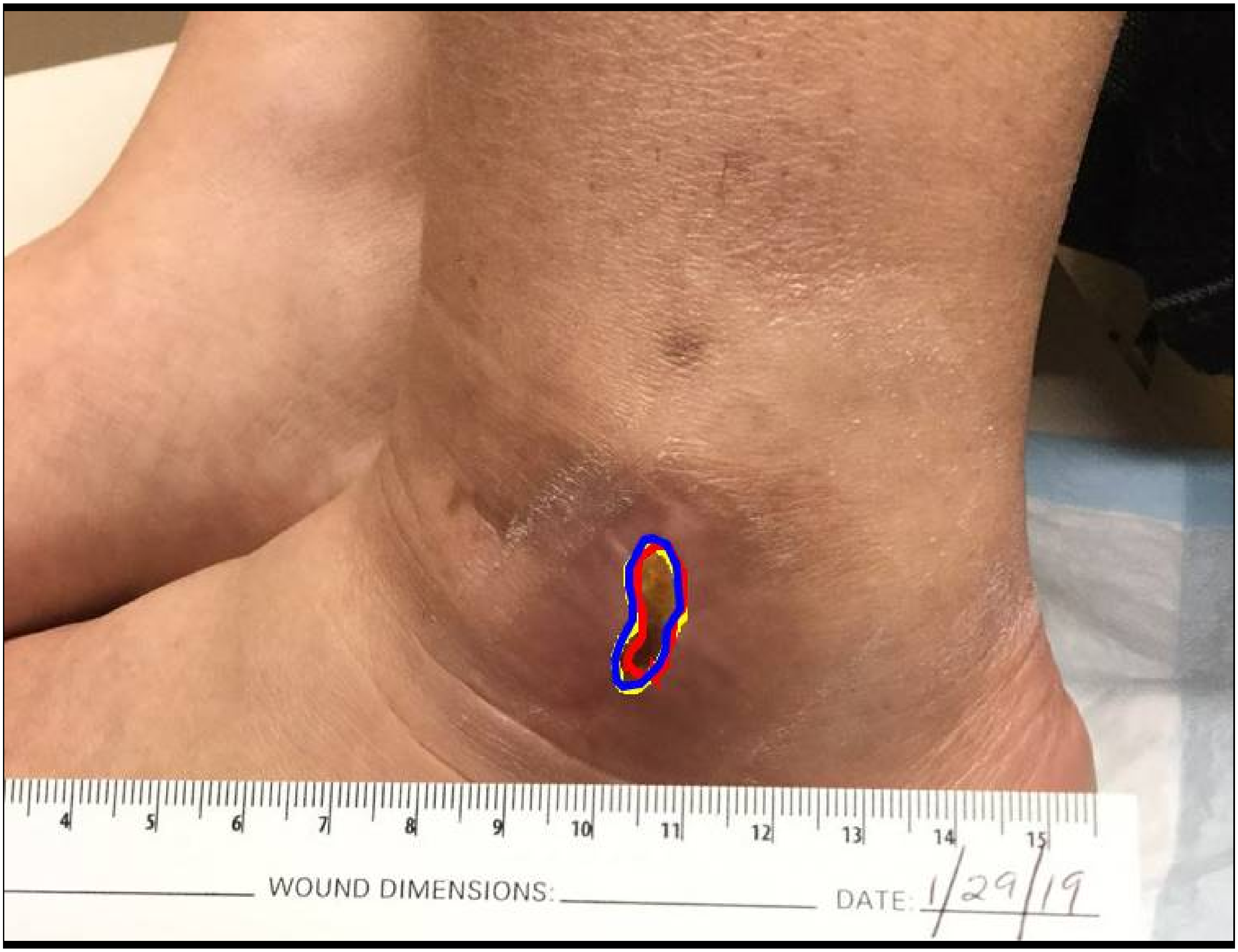

Tracing Data			
Tracer:	Wound Area (px^2)	Ruler Calibration (px/cm)	Wound Area (cm^2)
H1	2294	55.5	0.75
H2	1751	54.0	0.60
AI	2299	53.6	0.80

Tracing Comparisons				
Difference Metric:	Human-Human		Human-AI	
	H1(ref) H2(test)	H2(ref) H1(test)	H1(ref) AI(test)	H2(ref) AI(test)
False Negative Area (%)	29.3	7.4	7.1	7.3
False Positive Area (%)	5.6	38.4	7.3	38.6
|Relative Error (%)|	23.7	31.0	0.2	31.3

Blinded Attending Surgeon Review						
Reviewer	PGT Estimate	H1 meets definition?	H2 meets definition?	AI meets definition?	Which is AI?	Which is most accurate?
1	100	No	No	No	H2	AI
2	70	Yes	Yes	Yes	AI	H1
3	10	Yes	Yes	Yes	H2	AI

© 2021 Howell RS et al. *JAMA Network Open.*

Wound EMR Information								
Sequential Number	Gender	Age	Wound Type	Wound Location	Wound Length (cm)	Wound Width (cm)	Wound Depth (cm)	Wound Area (cm^2)
40	F	68	Venous	Leg-Ankle				

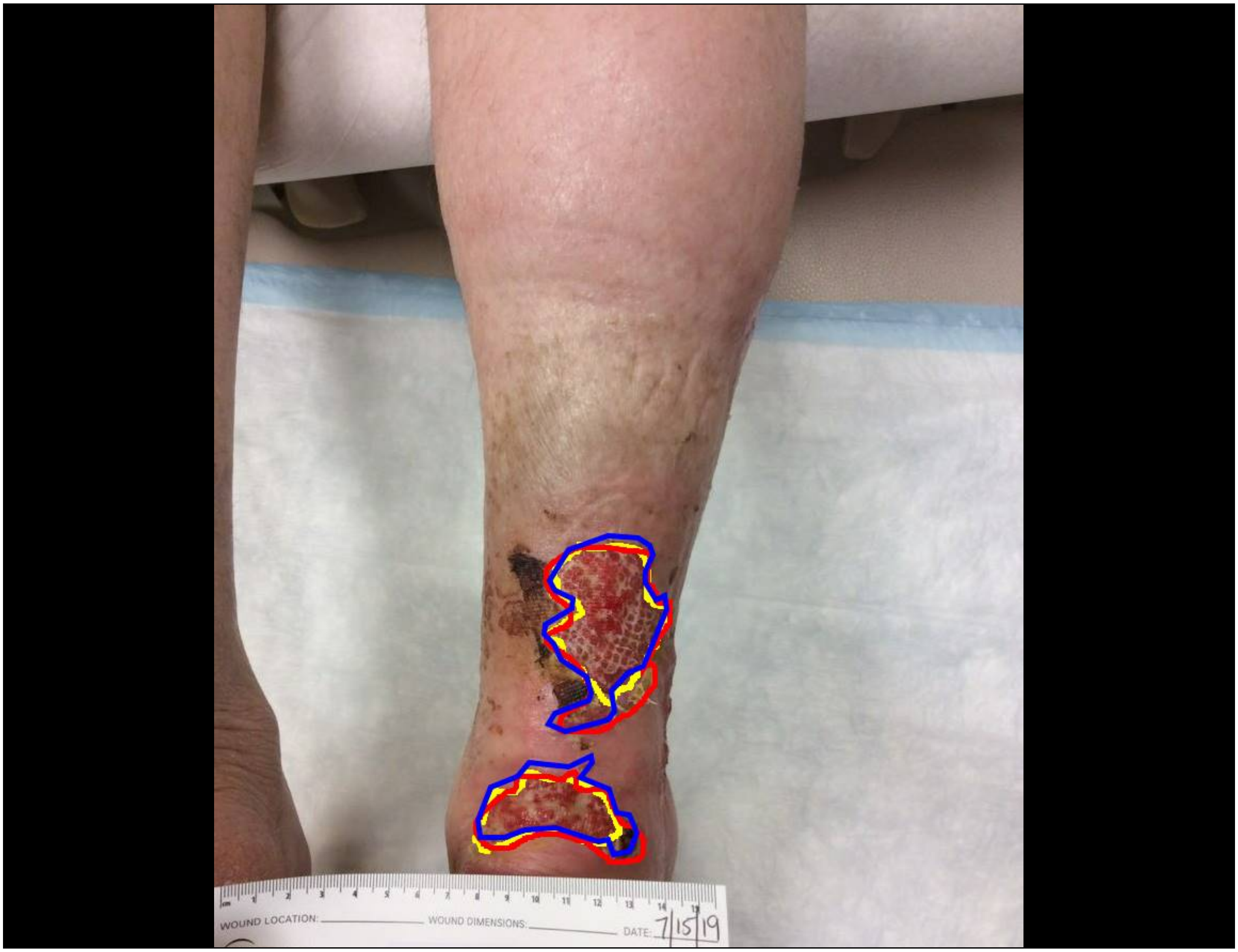

Tracing Data			
Tracer:	Wound Area (px^2)	Ruler Calibration (px/cm)	Wound Area (cm^2)
H1	16519	28.3	20.60
H2	21055	35.8	16.43
AI	20240	32.0	19.80

Tracing Comparisons				
Difference Metric:	Human-Human		Human-AI	
	H1(ref) H2(test)	H2(ref) H1(test)	H1(ref) AI(test)	H2(ref) AI(test)
False Negative Area (%)	3.1	24.0	6.2	16.6
False Positive Area (%)	30.5	2.4	28.7	12.7
|Relative Error (%)|	27.5	21.5	22.5	3.9

Blinded Attending Surgeon Review						
Reviewer	PGT Estimate	H1 meets definition?	H2 meets definition?	AI meets definition?	Which is AI?	Which is most accurate?
1	100	Yes	Yes	Yes	H2	H2
2	50	Yes	Yes	Yes	H2	H2
3	70	No	Yes	Yes	AI	AI

© 2021 Howell RS et al. *JAMA Network Open.*

Wound EMR Information								
Sequential Number	Gender	Age	Wound Type	Wound Location	Wound Length (cm)	Wound Width (cm)	Wound Depth (cm)	Wound Area (cm^2)
41	F	31	Surgical	Abdomen				

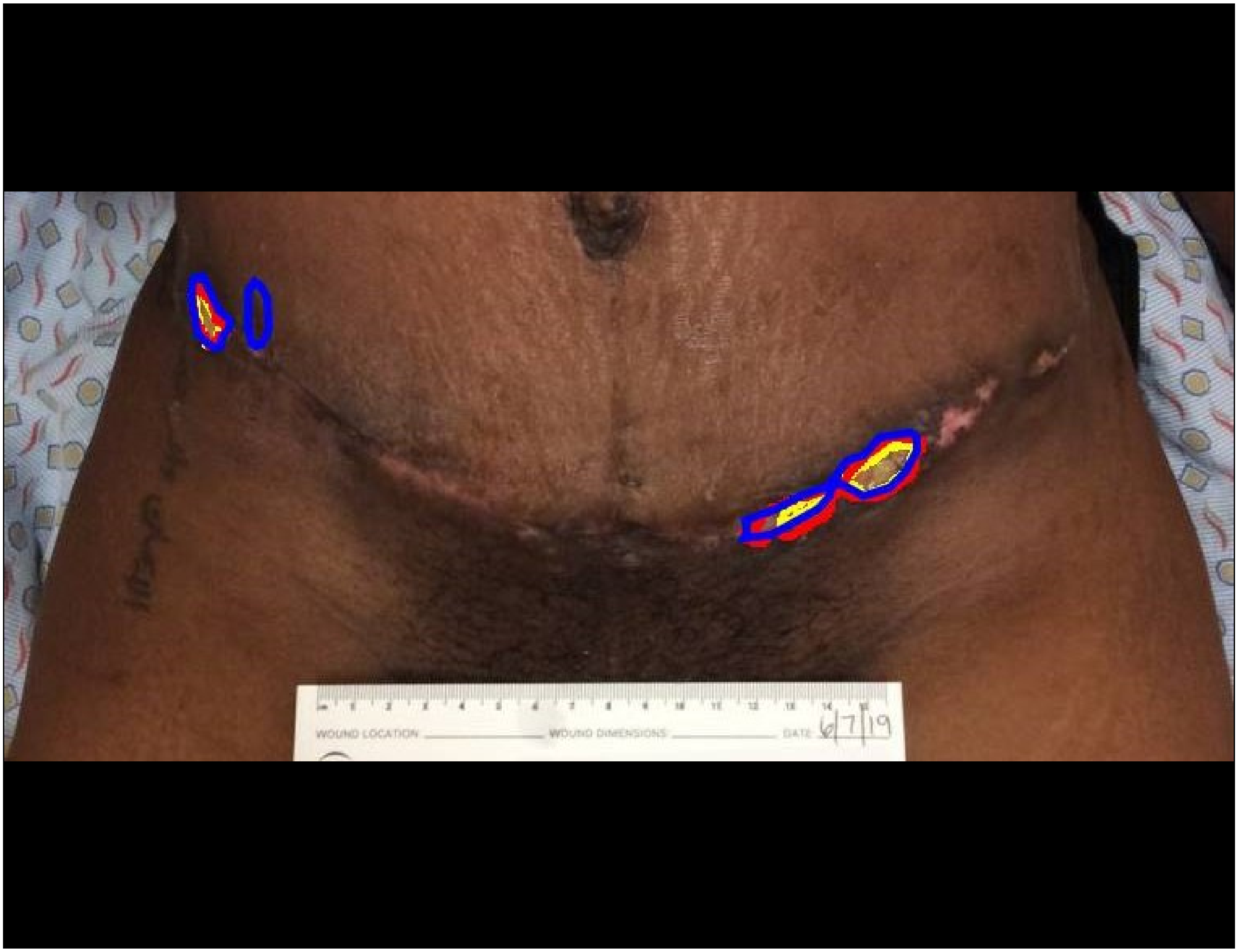

Tracing Data			
Tracer:	Wound Area (px^2)	Ruler Calibration (px/cm)	Wound Area (cm^2)
H1	1235	22.7	2.39
H2	2393	28.0	3.05
AI	2860	24.7	4.70

Tracing Comparisons				
Difference Metric:	Human-Human		Human-AI	
	H1(ref) H2(test)	H2(ref) H1(test)	H1(ref) AI(test)	H2(ref) AI(test)
False Negative Area (%)	0.6	48.7	0.6	18.1
False Positive Area (%)	94.3	0.3	132.2	37.7
|Relative Error (%)|	93.8	48.4	131.6	19.5

Blinded Attending Surgeon Review						
Reviewer	PGT Estimate	H1 meets definition?	H2 meets definition?	AI meets definition?	Which is AI?	Which is most accurate?
1	80	Yes	Yes	Yes	AI	H2
2	70	Yes	Yes	Yes	H2	H2
3	20	Yes	No	No	H1	H2

© 2021 Howell RS et al. *JAMA Network Open.*

Wound EMR Information								
Sequential Number	Gender	Age	Wound Type	Wound Location	Wound Length (cm)	Wound Width (cm)	Wound Depth (cm)	Wound Area (cm^2)
42	M	56	Pressure	Hip-R				

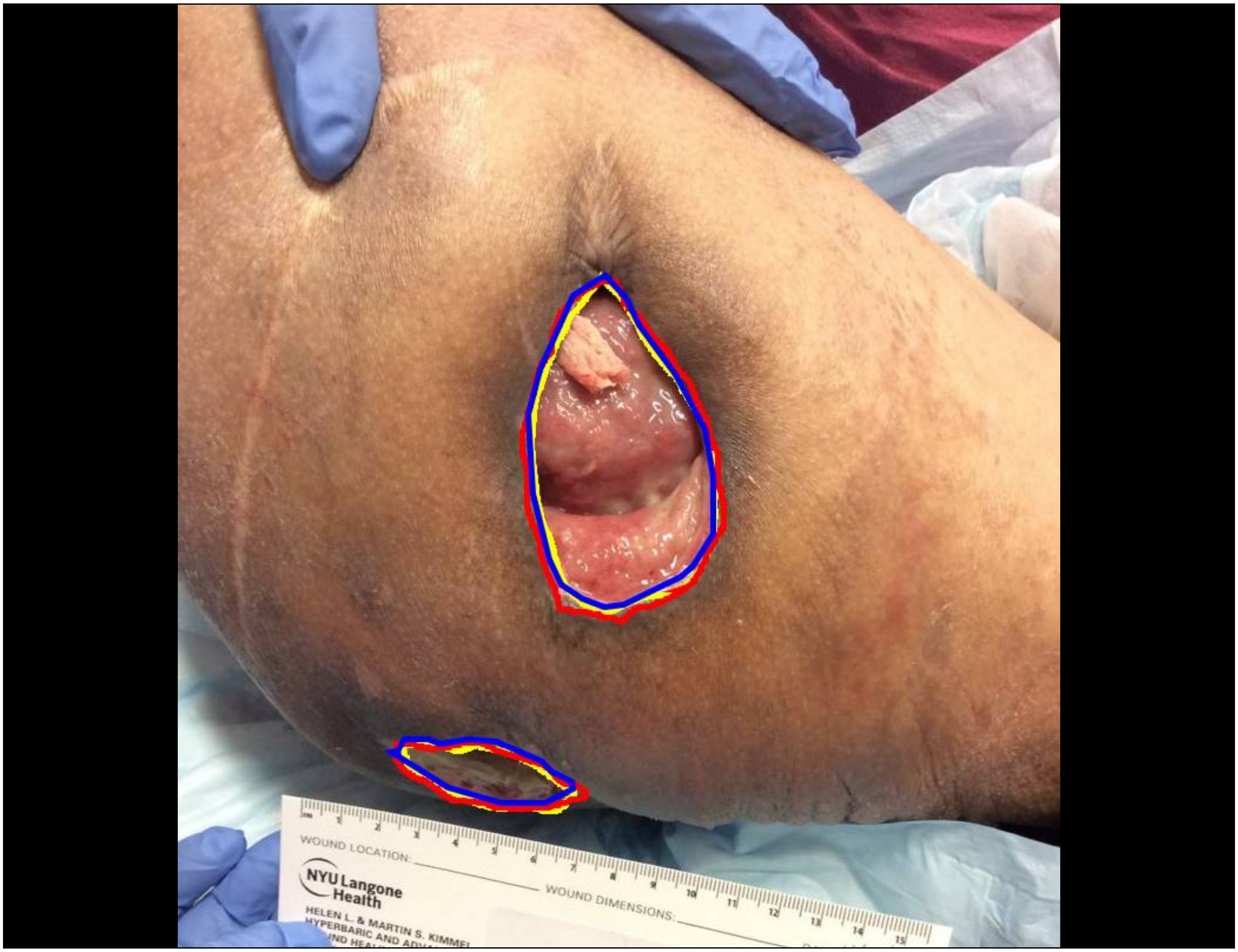

Tracing Data			
Tracer:	Wound Area (px^2)	Ruler Calibration (px/cm)	Wound Area (cm^2)
H1	36016	34.9	29.59
H2	40714	28.5	50.06
AI	36700	36.1	28.20

Tracing Comparisons				
Difference Metric:	Human-Human		Human-AI	
	H1(ref) H2(test)	H2(ref) H1(test)	H1(ref) AI(test)	H2(ref) AI(test)
False Negative Area (%)	0.6	12.0	3.9	11.5
False Positive Area (%)	13.6	0.5	5.8	1.6
|Relative Error (%)|	13.0	11.5	1.9	9.9

Blinded Attending Surgeon Review						
Reviewer	PGT Estimate	H1 meets definition?	H2 meets definition?	AI meets definition?	Which is AI?	Which is most accurate?
1	90	Yes	Yes	Yes	H2	H1
2	100	Yes	Yes	Yes	AI	AI
3	90	Yes	Yes	Yes	H2	AI

© 2021 Howell RS et al. *JAMA Network Open.*

Wound EMR Information								
Sequential Number	Gender	Age	Wound Type	Wound Location	Wound Length (cm)	Wound Width (cm)	Wound Depth (cm)	Wound Area (cm^2)
43	M	56	Pressure	Sacrum				

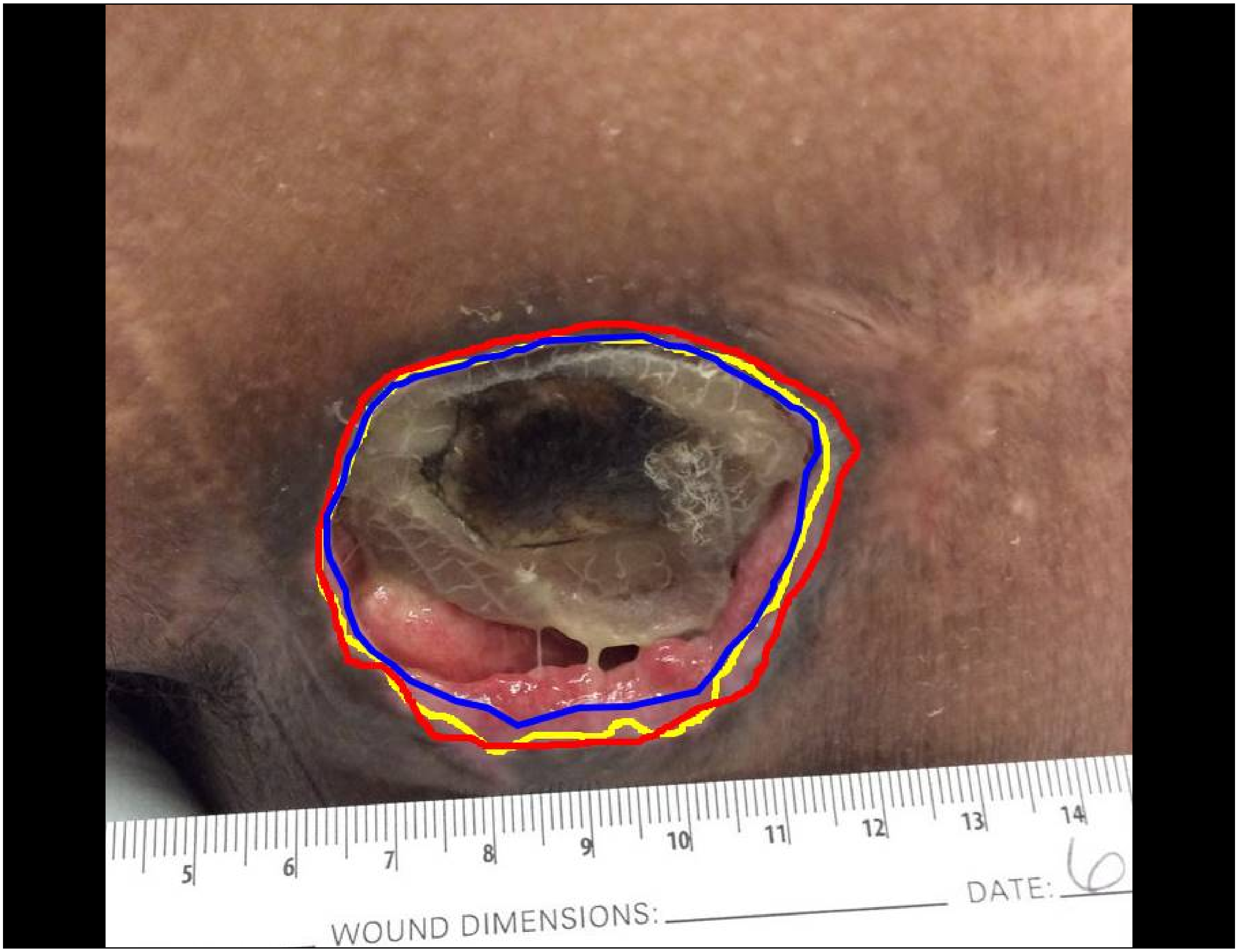

Tracing Data			
Tracer:	Wound Area (px^2)	Ruler Calibration (px/cm)	Wound Area (cm^2)
H1	85057	70.9	16.94
H2	93852	71.7	18.23
AI	78094	73.4	14.50

Tracing Comparisons				
Difference Metric:	Human-Human		Human-AI	
	H1(ref) H2(test)	H2(ref) H1(test)	H1(ref) AI(test)	H2(ref) AI(test)
False Negative Area (%)	0.4	9.8	8.4	16.8
False Positive Area (%)	10.8	0.4	0.2	0.0
|Relative Error (%)|	10.3	9.4	8.2	16.8

Blinded Attending Surgeon Review						
Reviewer	PGT Estimate	H1 meets definition?	H2 meets definition?	AI meets definition?	Which is AI?	Which is most accurate?
1	10	Yes	Yes	Yes	H1	H1
2	100	Yes	Yes	Yes	H2	AI
3	20	Yes	Yes	Yes	H1	AI

© 2021 Howell RS et al. *JAMA Network Open.*

Wound EMR Information								
Sequential Number	Gender	Age	Wound Type	Wound Location	Wound Length (cm)	Wound Width (cm)	Wound Depth (cm)	Wound Area (cm^2)
44	M	60	Abscess	Back				

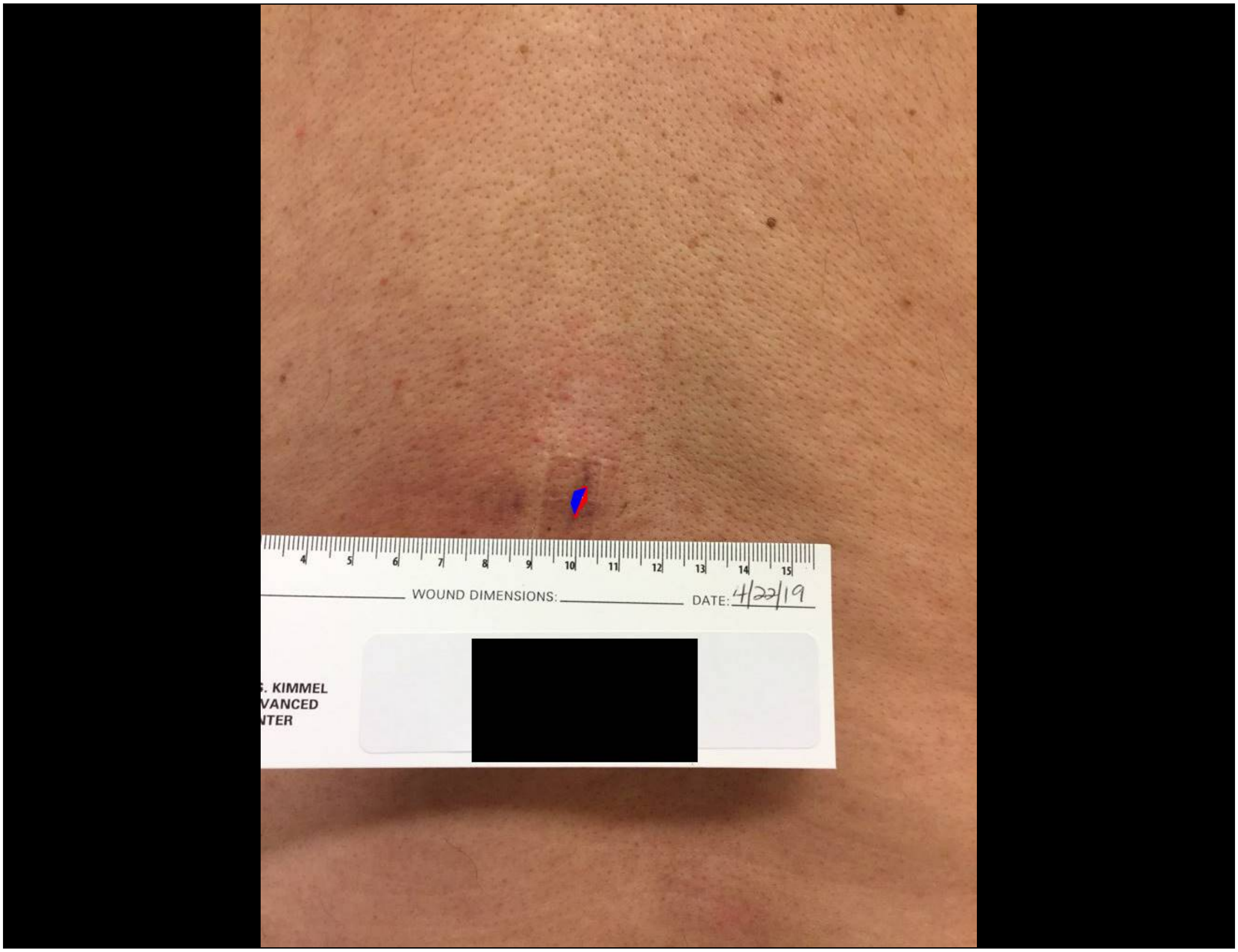

Tracing Data			
Tracer:	Wound Area (px^2)	Ruler Calibration (px/cm)	Wound Area (cm^2)
H1	31	47.1	0.01
H2	124	50.3	0.05
AI	70	49.6	0.00

Tracing Comparisons				
Difference Metric:	Human-Human		Human-AI	
	H1(ref) H2(test)	H2(ref) H1(test)	H1(ref) AI(test)	H2(ref) AI(test)
False Negative Area (%)	0.0	75.0	93.5	80.6
False Positive Area (%)	300.0	0.0	219.4	37.1
|Relative Error (%)|	300.0	75.0	125.8	43.5

Blinded Attending Surgeon Review						
Reviewer	PGT Estimate	H1 meets definition?	H2 meets definition?	AI meets definition?	Which is AI?	Which is most accurate?
1	100	No	No	No	H2	H1
2	0					
3	0	No	No	No	H1	H2

© 2021 Howell RS et al. *JAMA Network Open.*

Wound EMR Information								
Sequential Number	Gender	Age	Wound Type	Wound Location	Wound Length (cm)	Wound Width (cm)	Wound Depth (cm)	Wound Area (cm^2)
45	M	34	Kaposi/HIV	Thigh -Medial	5.0			

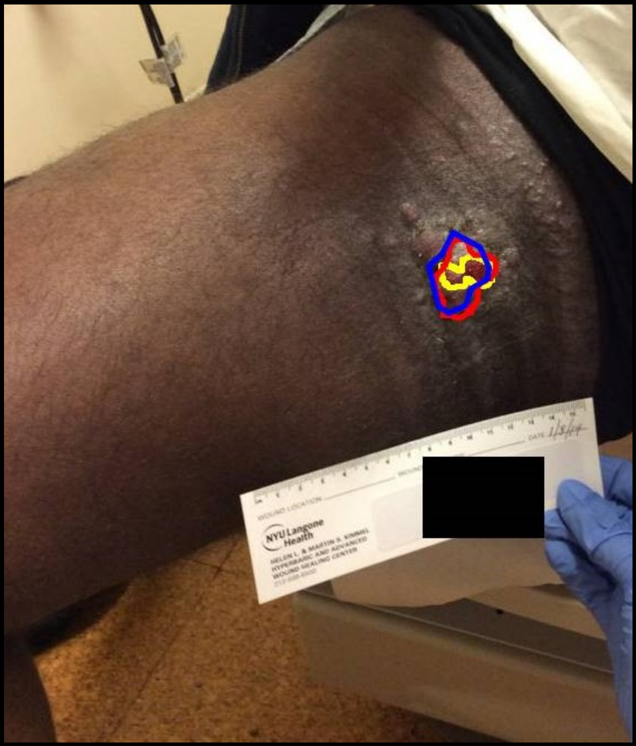

Tracing Data			
Tracer:	Wound Area (px^2)	Ruler Calibration (px/cm)	Wound Area (cm^2)
H1	893	19.2	2.41
H2	2299	21.2	5.10
AI	2353	22.6	4.60

Tracing Comparisons				
Difference Metric:	Human-Human		Human-AI	
	H1(ref) H2(test)	H2(ref) H1(test)	H1(ref) AI(test)	H2(ref) AI(test)
False Negative Area (%)	5.8	63.4	8.3	16.6
False Positive Area (%)	163.3	2.3	171.8	19.0
|Relative Error (%)|	157.4	61.2	163.5	2.3

Blinded Attending Surgeon Review						
Reviewer	PGT Estimate	H1 meets definition?	H2 meets definition?	AI meets definition?	Which is AI?	Which is most accurate?
1	100	Yes	No	No	H1	H2
2	100	Yes	No	No	H1	H2
3	100	Yes	No	No	AI	H1

© 2021 Howell RS et al. *JAMA Network Open.*

Wound EMR Information								
Sequential Number	Gender	Age	Wound Type	Wound Location	Wound Length (cm)	Wound Width (cm)	Wound Depth (cm)	Wound Area (cm^2)
46	F	68	Surgical	Ankle	4.0			

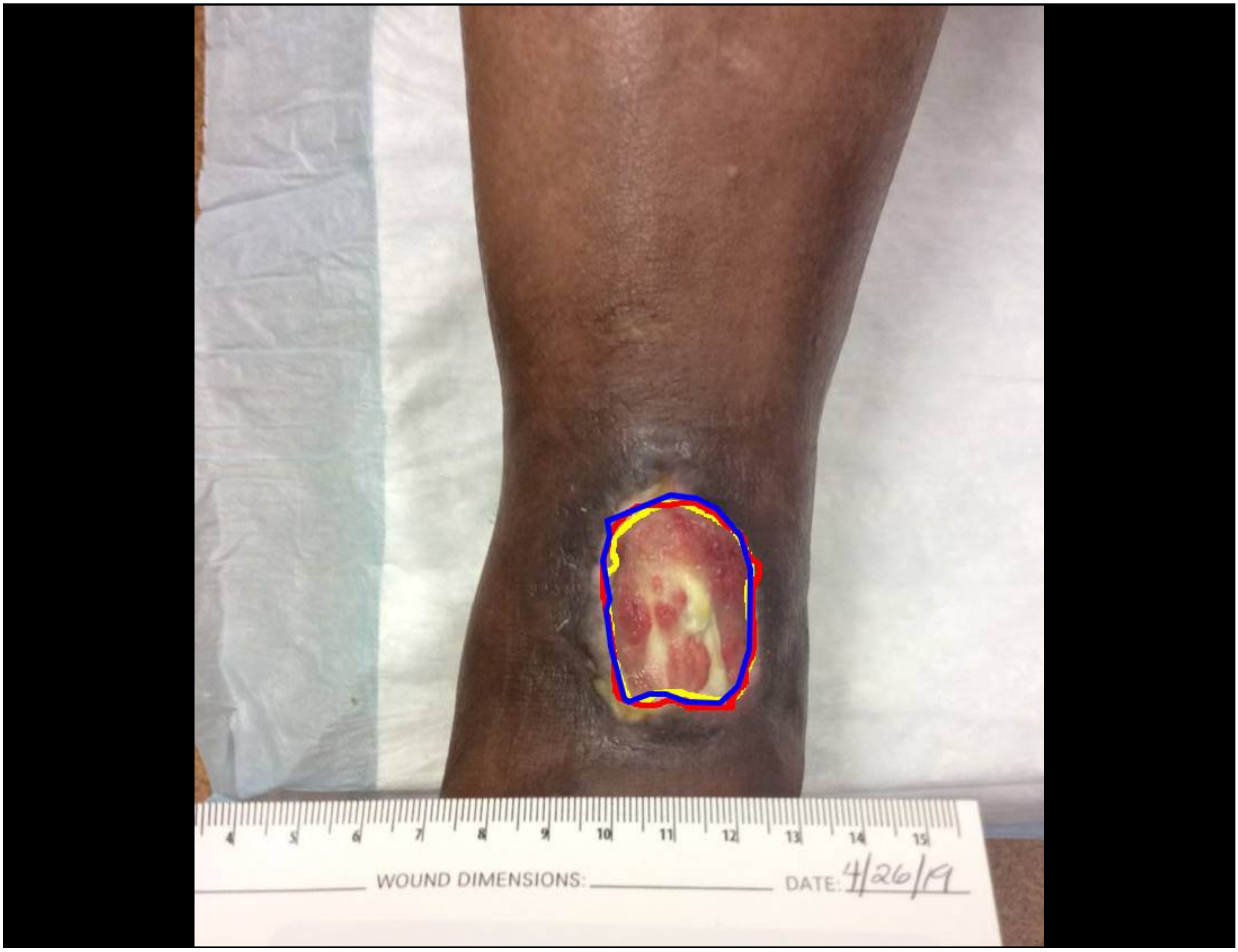

Tracing Data			
Tracer:	Wound Area (px^2)	Ruler Calibration (px/cm)	Wound Area (cm^2)
H1	19184	55.0	6.34
H2	21177	66.4	4.80
AI	20394	58.3	6.00

Tracing Comparisons				
Difference Metric:	Human-Human		Human-AI	
	H1(ref) H2(test)	H2(ref) H1(test)	H1(ref) AI(test)	H2(ref) AI(test)
False Negative Area (%)	0.3	9.7	1.6	5.9
False Positive Area (%)	10.7	0.3	7.9	2.2
|Relative Error (%)|	10.4	9.4	6.3	3.7

Blinded Attending Surgeon Review						
Reviewer	PGT Estimate	H1 meets definition?	H2 meets definition?	AI meets definition?	Which is AI?	Which is most accurate?
1	80	Yes	Yes	Yes	H1	H1
2	70	Yes	Yes	Yes	H2	H1
3	75	Yes	Yes	Yes	H1	H2

© 2021 Howell RS et al. *JAMA Network Open.*

Wound EMR Information								
Sequential Number	Gender	Age	Wound Type	Wound Location	Wound Length (cm)	Wound Width (cm)	Wound Depth (cm)	Wound Area (cm^2)
47	M	44	Trauma	Leg-Knee				

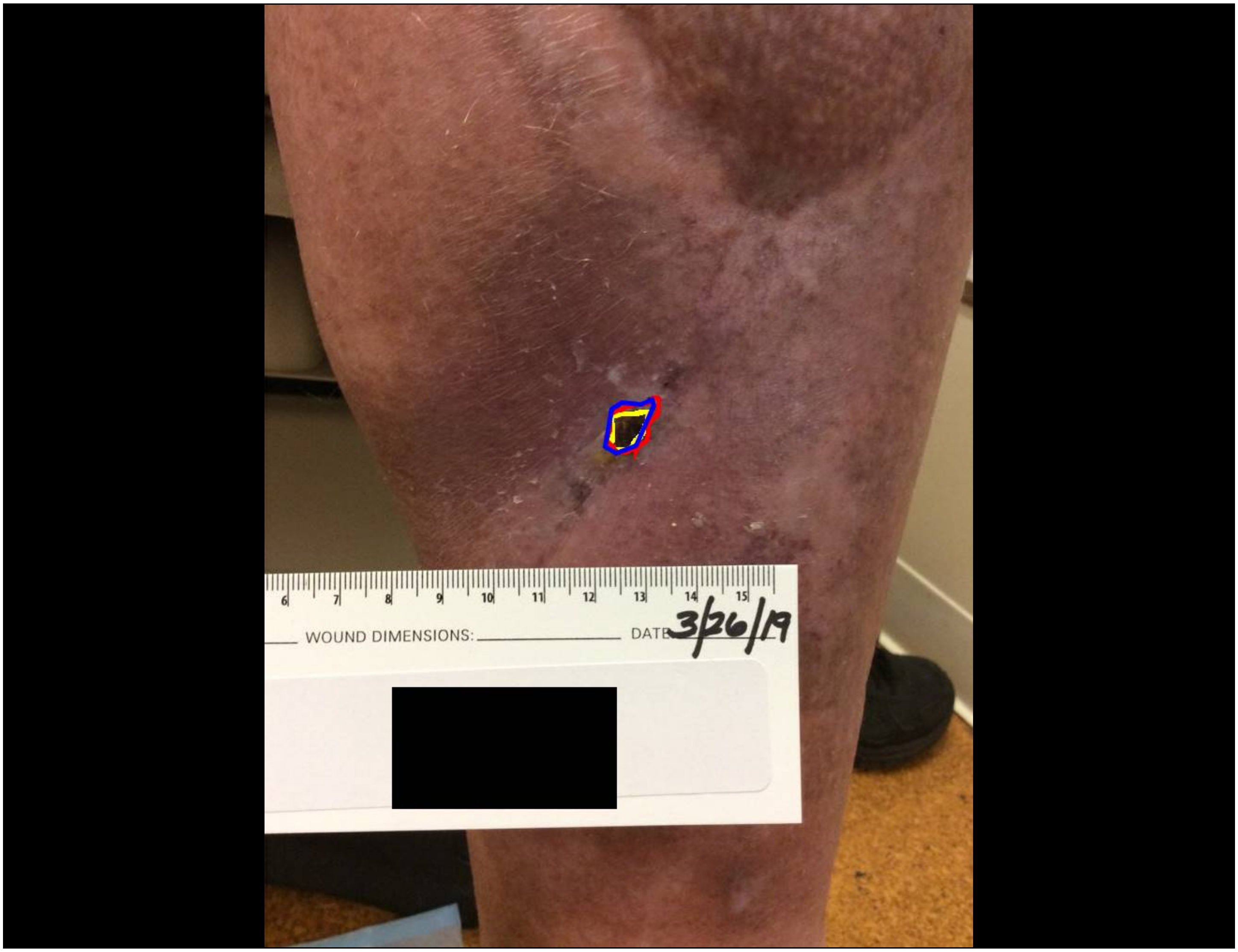

Tracing Data			
Tracer:	Wound Area (px^2)	Ruler Calibration (px/cm)	Wound Area (cm^2)
H1	1189	53.9	0.41
H2	2060	59.2	0.59
AI	1876	55.9	0.60

Tracing Comparisons				
Difference Metric:	Human-Human		Human-AI	
	H1(ref) H2(test)	H2(ref) H1(test)	H1(ref) AI(test)	H2(ref) AI(test)
False Negative Area (%)	0.3	42.5	0.0	17.0
False Positive Area (%)	73.6	0.2	57.8	8.1
|Relative Error (%)|	73.3	42.3	57.8	8.9

Blinded Attending Surgeon Review						
Reviewer	PGT Estimate	H1 meets definition?	H2 meets definition?	AI meets definition?	Which is AI?	Which is most accurate?
**1**	100	Yes	Yes	Yes	H2	H2
**2**	0					
**3**	0	No	Yes	Yes	AI	AI

© 2021 Howell RS et al. *JAMA Network Open.*

Wound EMR Information								
Sequential Number	Gender	Age	Wound Type	Wound Location	Wound Length (cm)	Wound Width (cm)	Wound Depth (cm)	Wound Area (cm^2)
49	M	59	Radiation	Back	1.0			

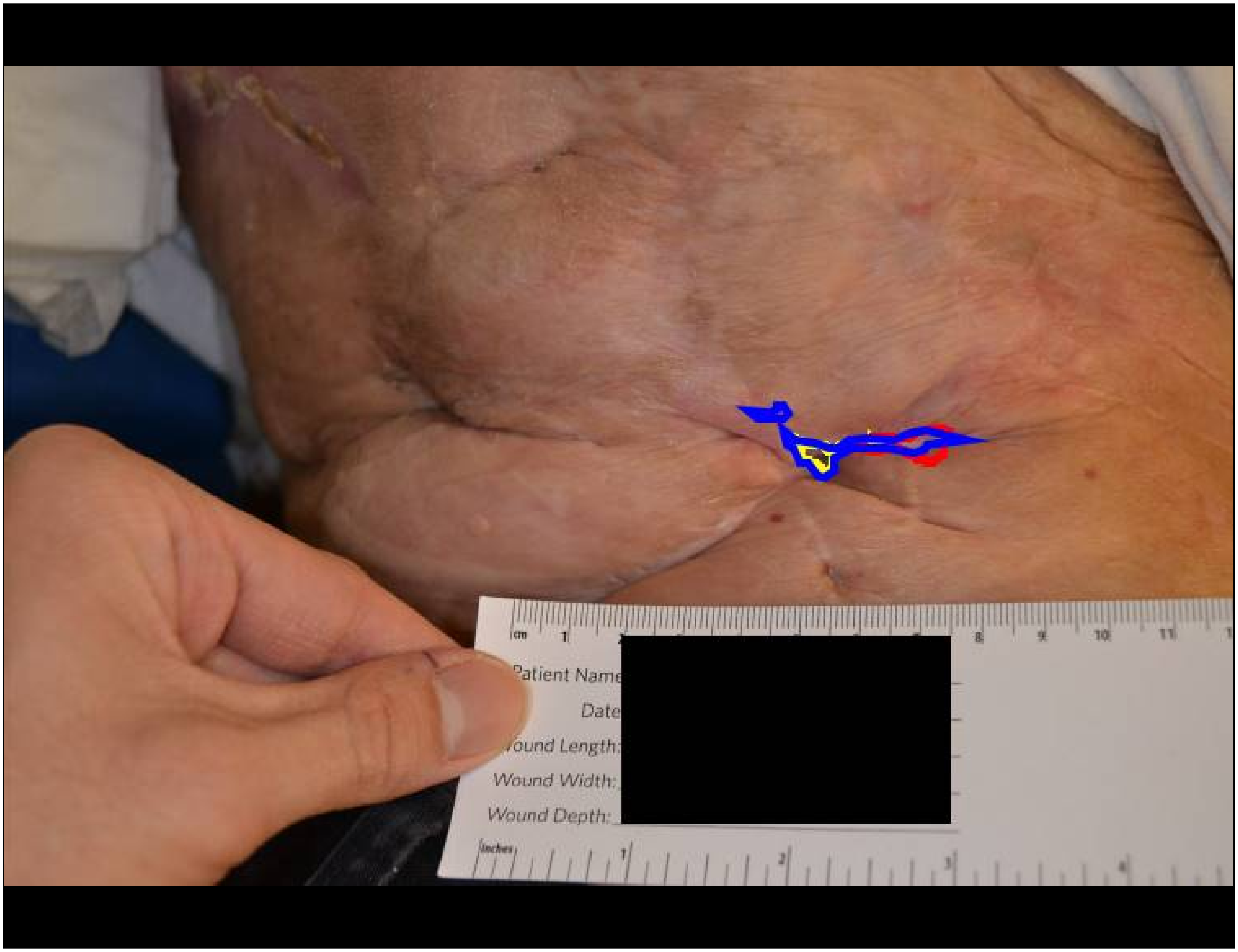

Tracing Data			
Tracer:	Wound Area (px^2)	Ruler Calibration (px/cm)	Wound Area (cm^2)
H1	332	34.9	0.27
H2	626	23.6	1.13
AI	1034	38.4	0.70

Tracing Comparisons				
Difference Metric:	Human-Human		Human-AI	
	H1(ref) H2(test)	H2(ref) H1(test)	H1(ref) AI(test)	H2(ref) AI(test)
False Negative Area (%)	87.0	93.1	1.2	36.6
False Positive Area (%)	175.6	46.2	212.7	101.8
|Relative Error (%)|	88.6	47.0	211.4	65.2

Blinded Attending Surgeon Review						
Reviewer	PGT Estimate	H1 meets definition?	H2 meets definition?	AI meets definition?	Which is AI?	Which is most accurate?
1	100	No	No	Yes	H1	H1
2	100	No	Yes	Yes	H1	H2
3	100	Yes	No	No	AI	H2

© 2021 Howell RS et al. *JAMA Network Open.*

Wound EMR Information								
Sequential Number	Gender	Age	Wound Type	Wound Location	Wound Length (cm)	Wound Width (cm)	Wound Depth (cm)	Wound Area (cm^2)
51	F	65	Venous	Right anterior				

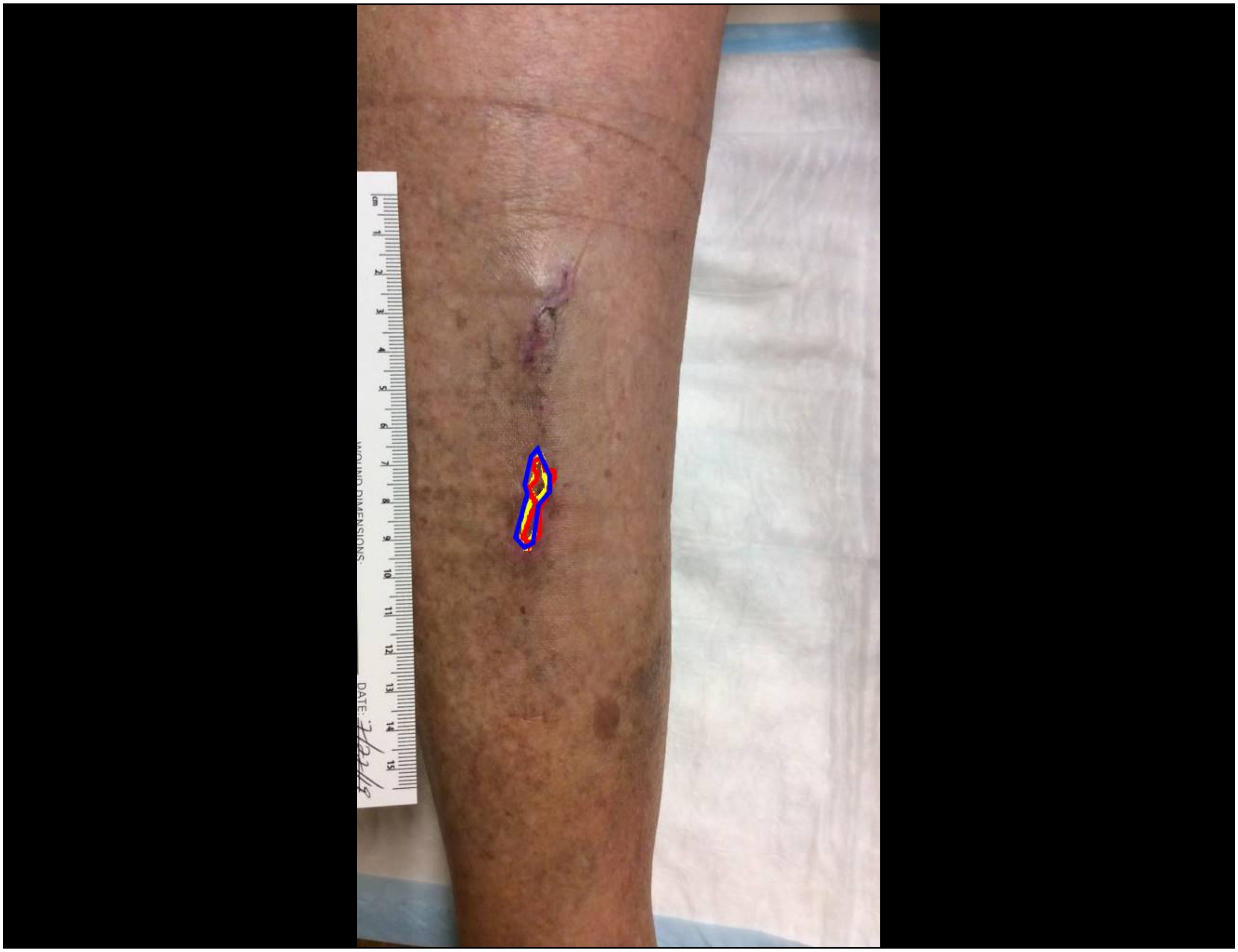

Tracing Data			
Tracer:	Wound Area (px^2)	Ruler Calibration (px/cm)	Wound Area (cm^2)
H1	1075	40.2	0.66
H2	1204	39.5	0.77
AI	1876	41.3	1.10

Tracing Comparisons				
Difference Metric:	Human-Human		Human-AI	
	H1(ref) H2(test)	H2(ref) H1(test)	H1(ref) AI(test)	H2(ref) AI(test)
False Negative Area (%)	24.0	32.1	0.8	15.0
False Positive Area (%)	36.0	21.4	75.3	70.8
|Relative Error (%)|	12.0	10.7	74.5	55.8

Blinded Attending Surgeon Review						
Reviewer	PGT Estimate	H1 meets definition?	H2 meets definition?	AI meets definition?	Which is AI?	Which is most accurate?
1	90	Yes	Yes	Yes	H2	H1
2	100	Yes	Yes	Yes	AI	AI
3	30	Yes	No	Yes	AI	AI

© 2021 Howell RS et al. *JAMA Network Open.*

Wound EMR Information								
Sequential Number	Gender	Age	Wound Type	Wound Location	Wound Length (cm)	Wound Width (cm)	Wound Depth (cm)	Wound Area (cm^2)
52	F	38	Venous	Ankle				

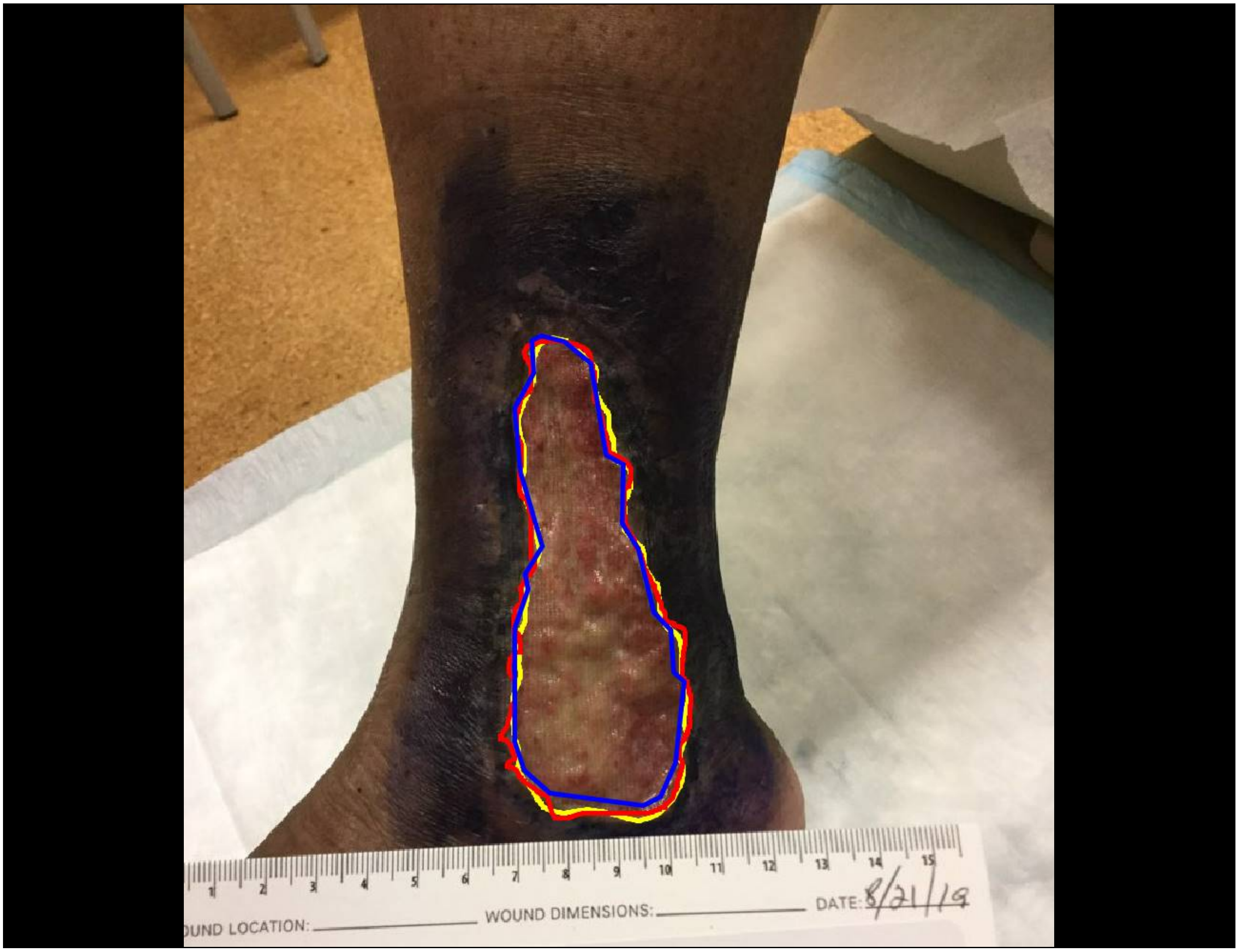

Tracing Data			
Tracer:	Wound Area (px^2)	Ruler Calibration (px/cm)	Wound Area (cm^2)
H1	63066	53.2	22.25
H2	64858	54.1	22.19
AI	58935	54.4	19.90

Tracing Comparisons				
Difference Metric:	Human-Human		Human-AI	
	H1(ref) H2(test)	H2(ref) H1(test)	H1(ref) AI(test)	H2(ref) AI(test)
False Negative Area (%)	2.1	4.8	8.3	9.9
False Positive Area (%)	5.0	2.1	1.8	0.7
|Relative Error (%)|	2.8	2.8	6.6	9.1

Blinded Attending Surgeon Review						
Reviewer	PGT Estimate	H1 meets definition?	H2 meets definition?	AI meets definition?	Which is AI?	Which is most accurate?
1	40	Yes	Yes	Yes	H2	AI
2	100	Yes	Yes	Yes	AI	H2
3	50	Yes	Yes	Yes	H1	H2

© 2021 Howell RS et al. *JAMA Network Open.*

Wound EMR Information								
Sequential Number	Gender	Age	Wound Type	Wound Location	Wound Length (cm)	Wound Width (cm)	Wound Depth (cm)	Wound Area (cm^2)
53	F	51	Surgical	Breast	5.0	0.3		1.50

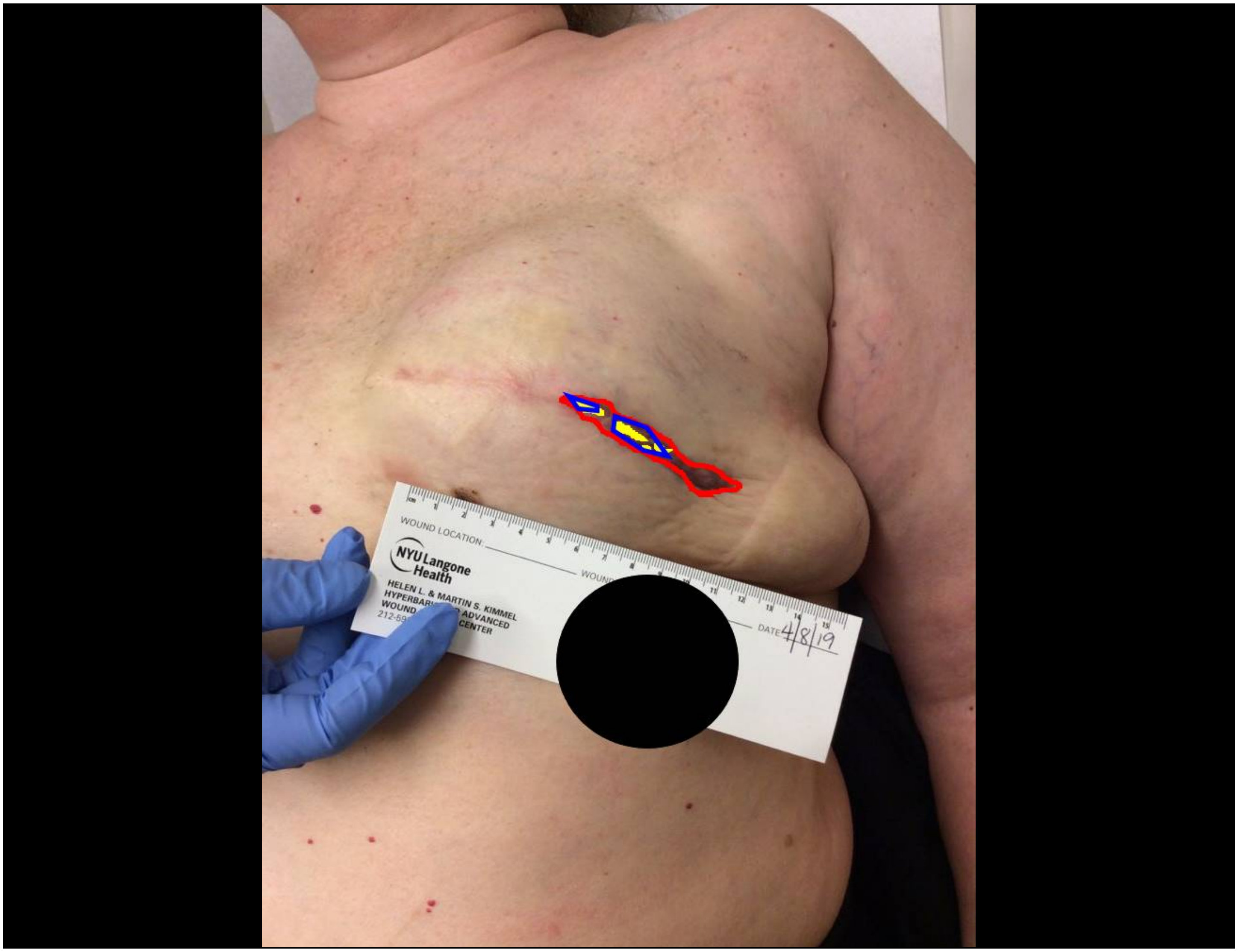

Tracing Data			
Tracer:	Wound Area (px^2)	Ruler Calibration (px/cm)	Wound Area (cm^2)
H1	233	30.9	0.24
H2	4160	31.2	4.26
AI	1185	30.2	1.30

Tracing Comparisons				
Difference Metric:	Human-Human		Human-AI	
	H1(ref) H2(test)	H2(ref) H1(test)	H1(ref) AI(test)	H2(ref) AI(test)
False Negative Area (%)	0.0	94.4	10.3	71.5
False Positive Area (%)	1685.4	0.0	418.9	0.0
|Relative Error (%)|	1685.4	94.4	408.6	71.5

Blinded Attending Surgeon Review						
Reviewer	PGT Estimate	H1 meets definition?	H2 meets definition?	AI meets definition?	Which is AI?	Which is most accurate?
1	100	No	Yes	No	H2	H1
2	100	No	Yes	No	H2	H1
3	100	Yes	No	No	H2	H2

© 2021 Howell RS et al. *JAMA Network Open.*

Wound EMR Information								
Sequential Number	Gender	Age	Wound Type	Wound Location	Wound Length (cm)	Wound Width (cm)	Wound Depth (cm)	Wound Area (cm^2)
54	F	43	Abscess	Breast	3.0			0.00

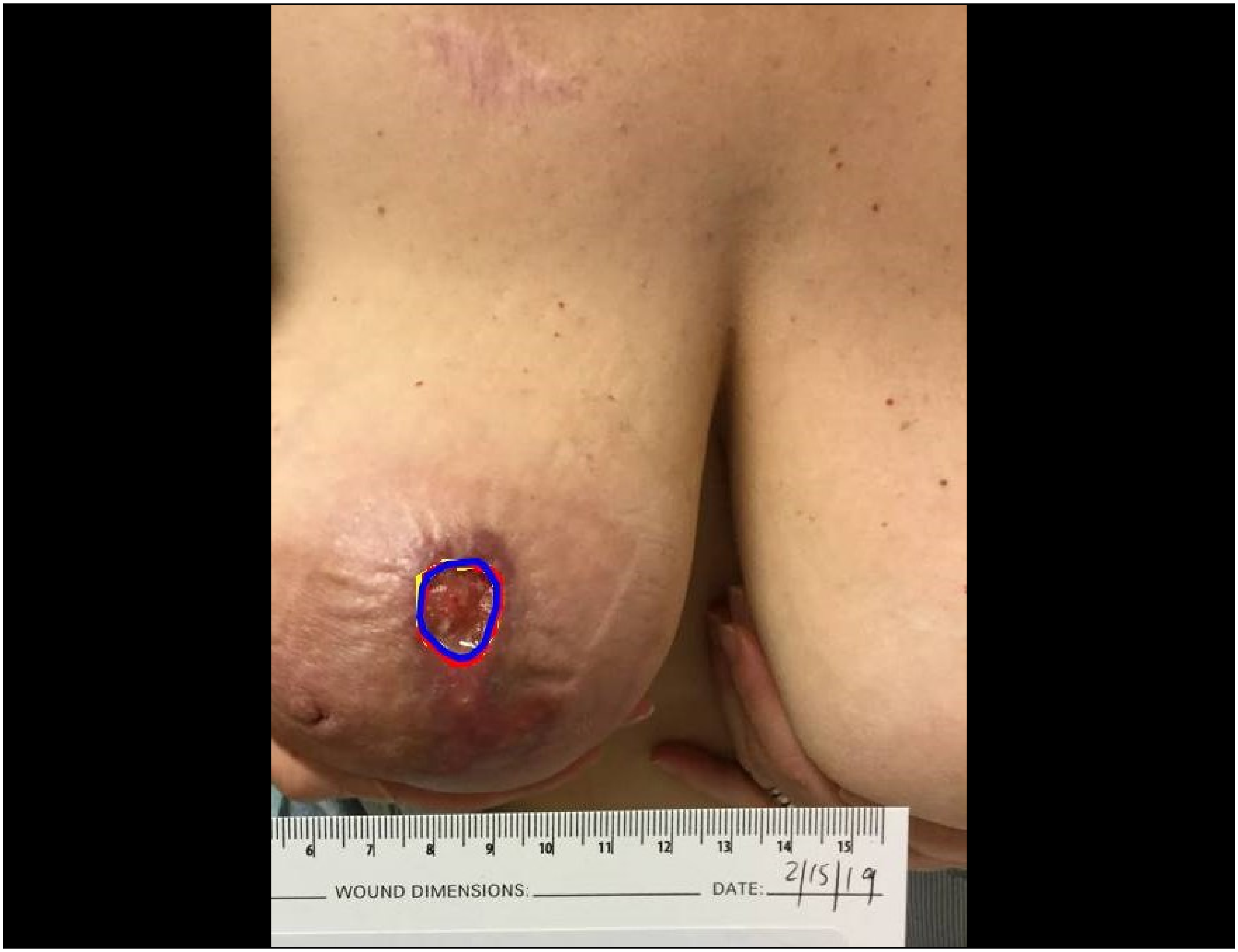

Tracing Data			
Tracer:	Wound Area (px^2)	Ruler Calibration (px/cm)	Wound Area (cm^2)
H1	3116	42.7	1.71
H2	3174	44.7	1.59
AI	2926	42.8	1.60

Tracing Comparisons				
Difference Metric:	Human-Human		Human-AI	
	H1(ref) H2(test)	H2(ref) H1(test)	H1(ref) AI(test)	H2(ref) AI(test)
False Negative Area (%)	3.5	5.2	7.3	9.6
False Positive Area (%)	5.3	3.4	1.2	1.8
|Relative Error (%)|	1.9	1.8	6.1	7.8

Blinded Attending Surgeon Review						
Reviewer	PGT Estimate	H1 meets definition?	H2 meets definition?	AI meets definition?	Which is AI?	Which is most accurate?
1	80	No	No	No	H1	H1
2	100	Yes	Yes	Yes	H2	AI
3	80	Yes	Yes	Yes	AI	H1

© 2021 Howell RS et al. *JAMA Network Open.*

Wound EMR Information								
Sequential Number	Gender	Age	Wound Type	Wound Location	Wound Length (cm)	Wound Width (cm)	Wound Depth (cm)	Wound Area (cm^2)
55	F	85	Trauma	Ankle				0.00

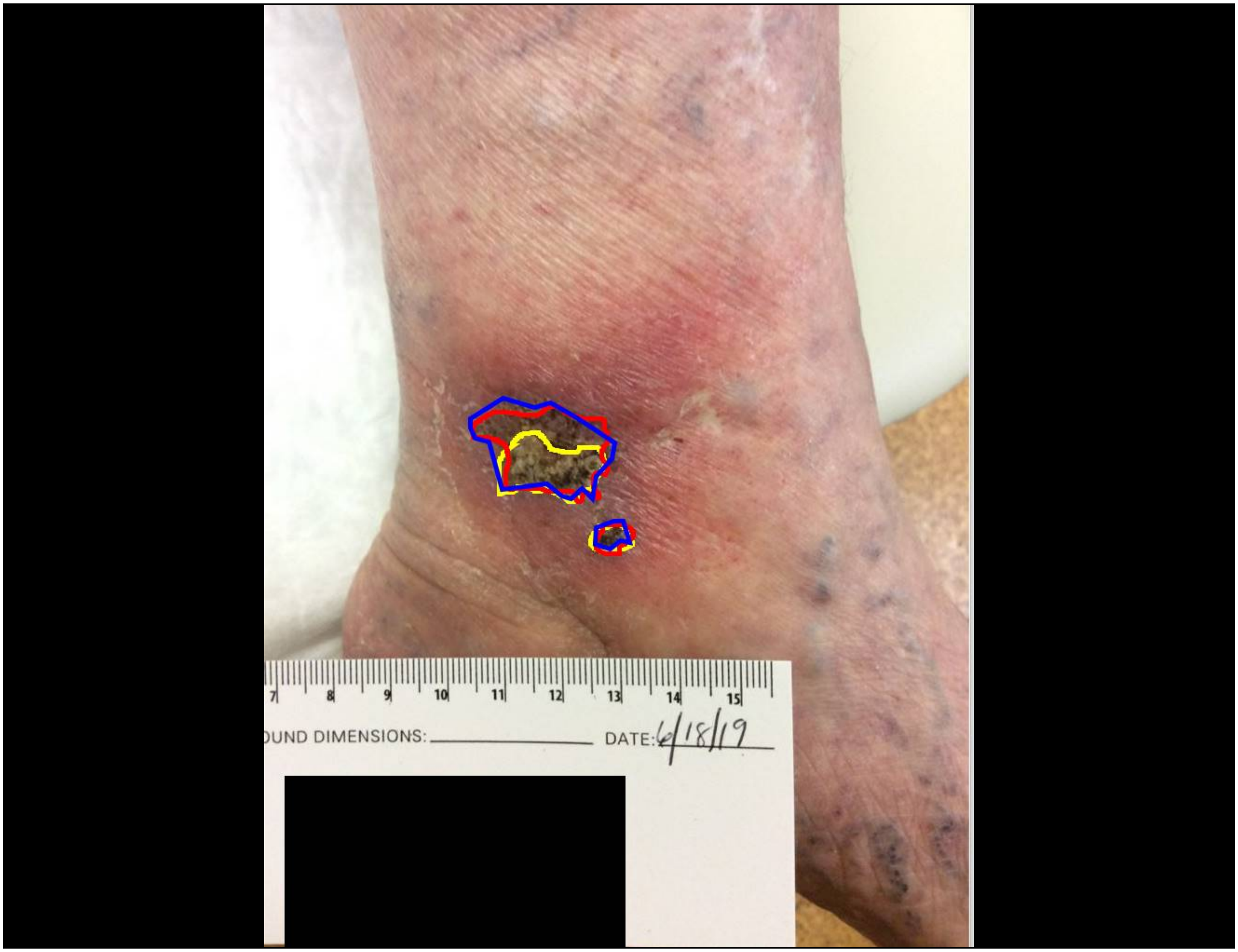

Tracing Data			
Tracer:	Wound Area (px^2)	Ruler Calibration (px/cm)	Wound Area (cm^2)
H1	6447	62.7	1.64
H2	10379	62.0	2.70
AI	11566	63.2	2.90

Tracing Comparisons				
Difference Metric:	Human-Human		Human-AI	
	H1(ref) H2(test)	H2(ref) H1(test)	H1(ref) AI(test)	H2(ref) AI(test)
False Negative Area (%)	12.9	45.9	8.9	8.2
False Positive Area (%)	73.9	8.0	88.3	19.7
|Relative Error (%)|	61.0	37.9	79.4	11.4

Blinded Attending Surgeon Review						
Reviewer	PGT Estimate	H1 meets definition?	H2 meets definition?	AI meets definition?	Which is AI?	Which is most accurate?
1	0	Yes	Yes	Yes	H2	AI
2	0					
3	0	No	Yes	Yes	H2	H1

© 2021 Howell RS et al. *JAMA Network Open.*

Wound EMR Information								
Sequential Number	Gender	Age	Wound Type	Wound Location	Wound Length (cm)	Wound Width (cm)	Wound Depth (cm)	Wound Area (cm²)
56	F	73	Diabetic	Left lateral foot	6.0	4.0		24.00

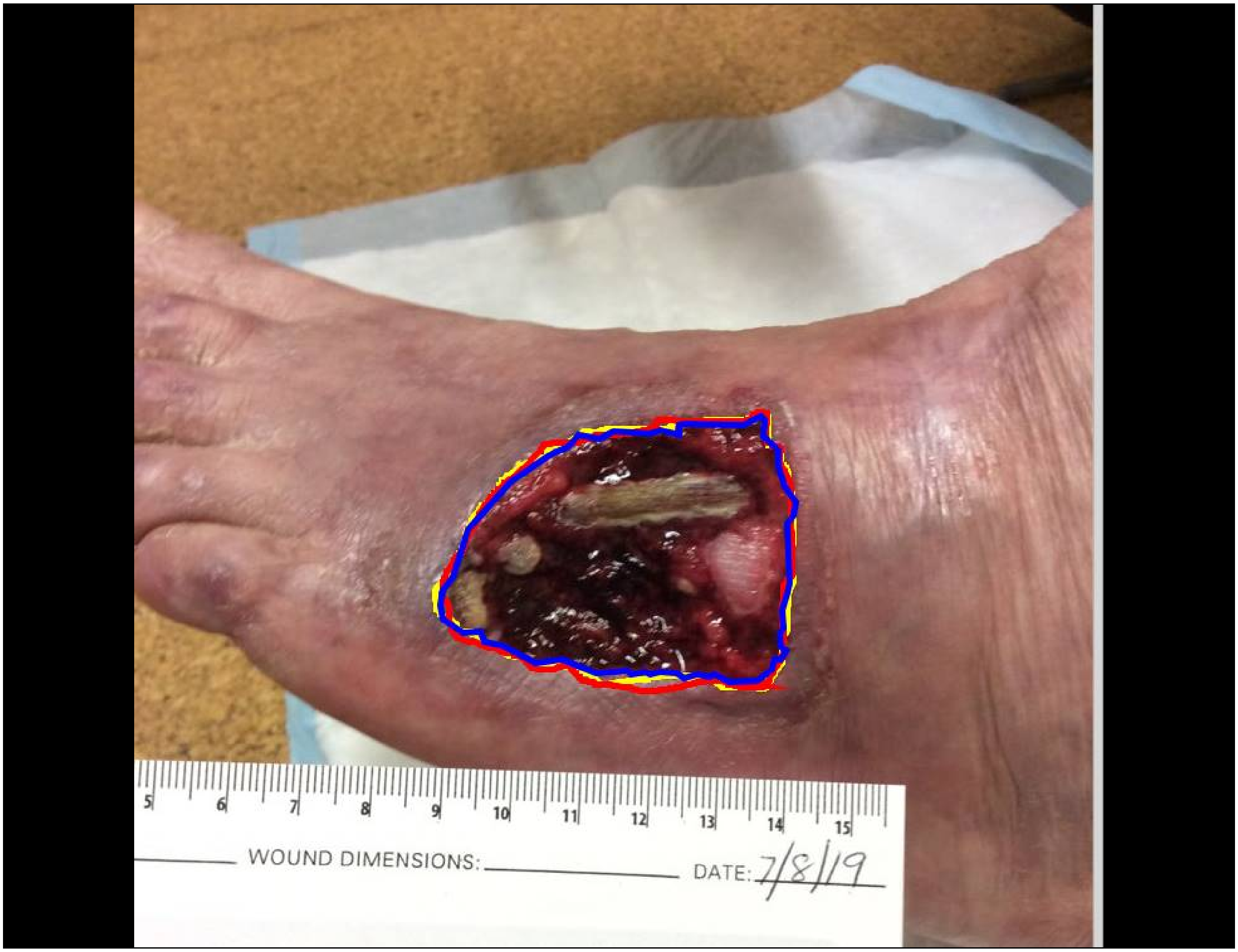

Tracing Data			
Tracer:	Wound Area (px²)	Ruler Calibration (px/cm)	Wound Area (cm²)
H1	47481	54.7	15.86
H2	47738	54.6	16.02
AI	45052	54.8	15.00

Tracing Comparisons				
Difference Metric:	Human-Human		Human-AI	
	H1(ref) H2(test)	H2(ref) H1(test)	H1(ref) AI(test)	H2(ref) AI(test)
False Negative Area (%)	2.2	2.7	5.3	6.3
False Positive Area (%)	2.7	2.2	0.2	0.7
|Relative Error (%)|	0.5	0.5	5.1	5.6

Blinded Attending Surgeon Review						
Reviewer	PGT Estimate	H1 meets definition?	H2 meets definition?	AI meets definition?	Which is AI?	Which is most accurate?
1	10	Yes	Yes	Yes	H2	AI
2	50	Yes	Yes	Yes	H1	AI
3	20	Yes	Yes	Yes	AI	H2

© 2021 Howell RS et al. *JAMA Network Open.*

Wound EMR Information								
Sequential Number	Gender	Age	Wound Type	Wound Location	Wound Length (cm)	Wound Width (cm)	Wound Depth (cm)	Wound Area (cm^2)
57	F	50	Venous	Right medial				

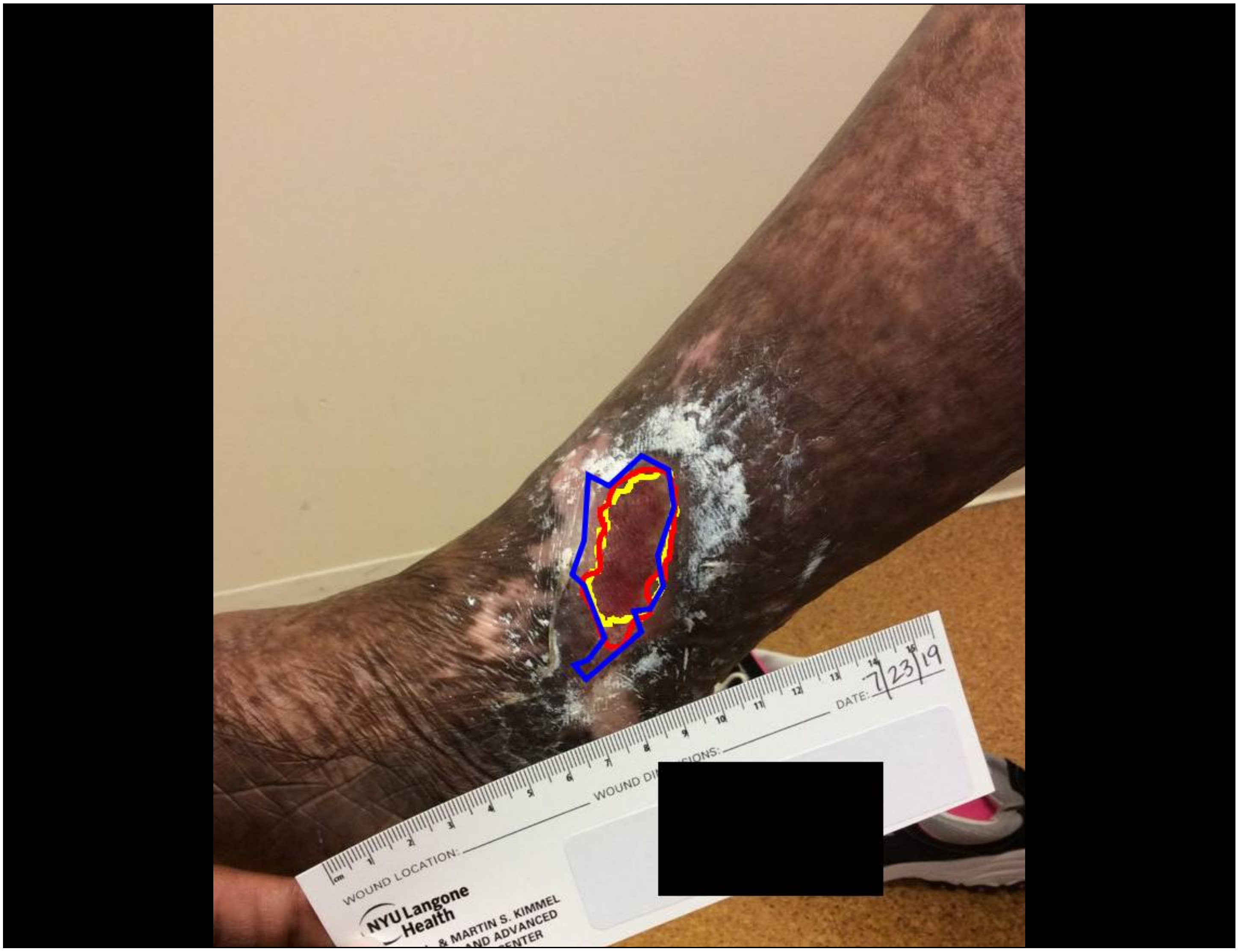

Tracing Data			
Tracer:	Wound Area (px^2)	Ruler Calibration (px/cm)	Wound Area (cm^2)
H1	7643	38.3	5.20
H2	8775	44.9	4.34
AI	11274	37.5	8.00

Tracing Comparisons				
Difference Metric:	Human-Human		Human-AI	
	H1(ref) H2(test)	H2(ref) H1(test)	H1(ref) AI(test)	H2(ref) AI(test)
False Negative Area (%)	4.3	16.6	3.5	6.1
False Positive Area (%)	19.1	3.7	51.0	34.5
|Relative Error (%)|	14.8	12.9	47.5	28.5

Blinded Attending Surgeon Review						
Reviewer	PGT Estimate	H1 meets definition?	H2 meets definition?	AI meets definition?	Which is AI?	Which is most accurate?
1	90	Yes	Yes	Yes	H2	AI
2	100	Yes	Yes	Yes	H1	H2
3	100	Yes	Yes	No	AI	H2

© 2021 Howell RS et al. *JAMA Network Open.*

Wound EMR Information								
Sequential Number	Gender	Age	Wound Type	Wound Location	Wound Length (cm)	Wound Width (cm)	Wound Depth (cm)	Wound Area (cm^2)
59	F	68	Venous	Right lateral leg	8.0	1.0		8.00

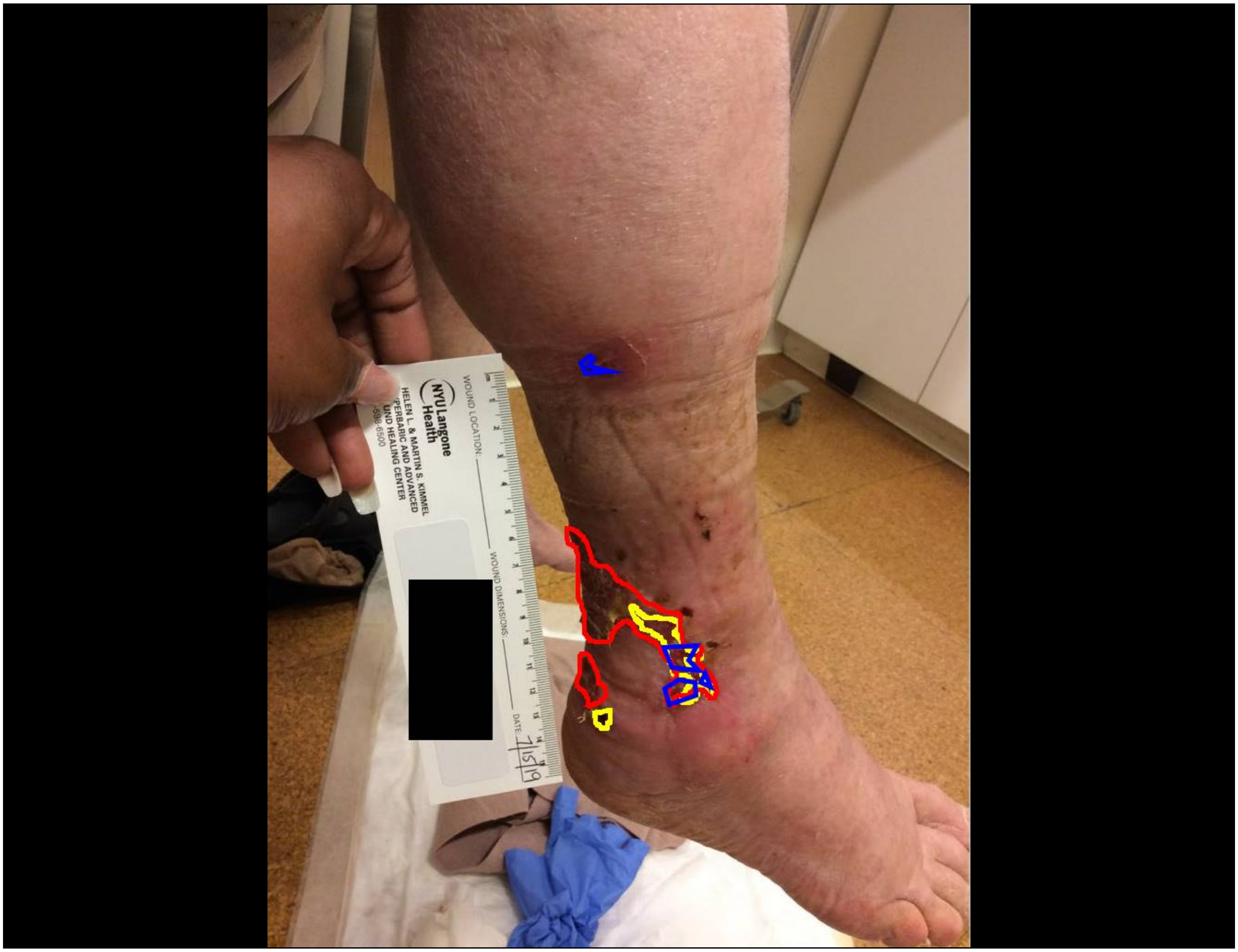

Tracing Data			
Tracer:	Wound Area (px^2)	Ruler Calibration (px/cm)	Wound Area (cm^2)
H1	2616	27.1	3.56
H2	9621	36.1	7.37
AI	1574	26.2	2.30

Tracing Comparisons				
Difference Metric:	Human-Human		Human-AI	
	H1(ref) H2(test)	H2(ref) H1(test)	H1(ref) AI(test)	H2(ref) AI(test)
False Negative Area (%)	17.0	77.4	69.3	87.8
False Positive Area (%)	284.7	4.6	29.4	4.1
|Relative Error (%)|	267.8	72.8	39.8	83.6

Blinded Attending Surgeon Review						
Reviewer	PGT Estimate	H1 meets definition?	H2 meets definition?	AI meets definition?	Which is AI?	Which is most accurate?
1	90	No	Yes	No	H1	H2
2	70	Yes	Yes	No	H1	AI
3	100	No	Yes	No	H2	H1

© 2021 Howell RS et al. *JAMA Network Open.*

Wound EMR Information								
Sequential Number	Gender	Age	Wound Type	Wound Location	Wound Length (cm)	Wound Width (cm)	Wound Depth (cm)	Wound Area (cm²)
60	F	37	Surgical	Left breast	1.0	1.0		1.00

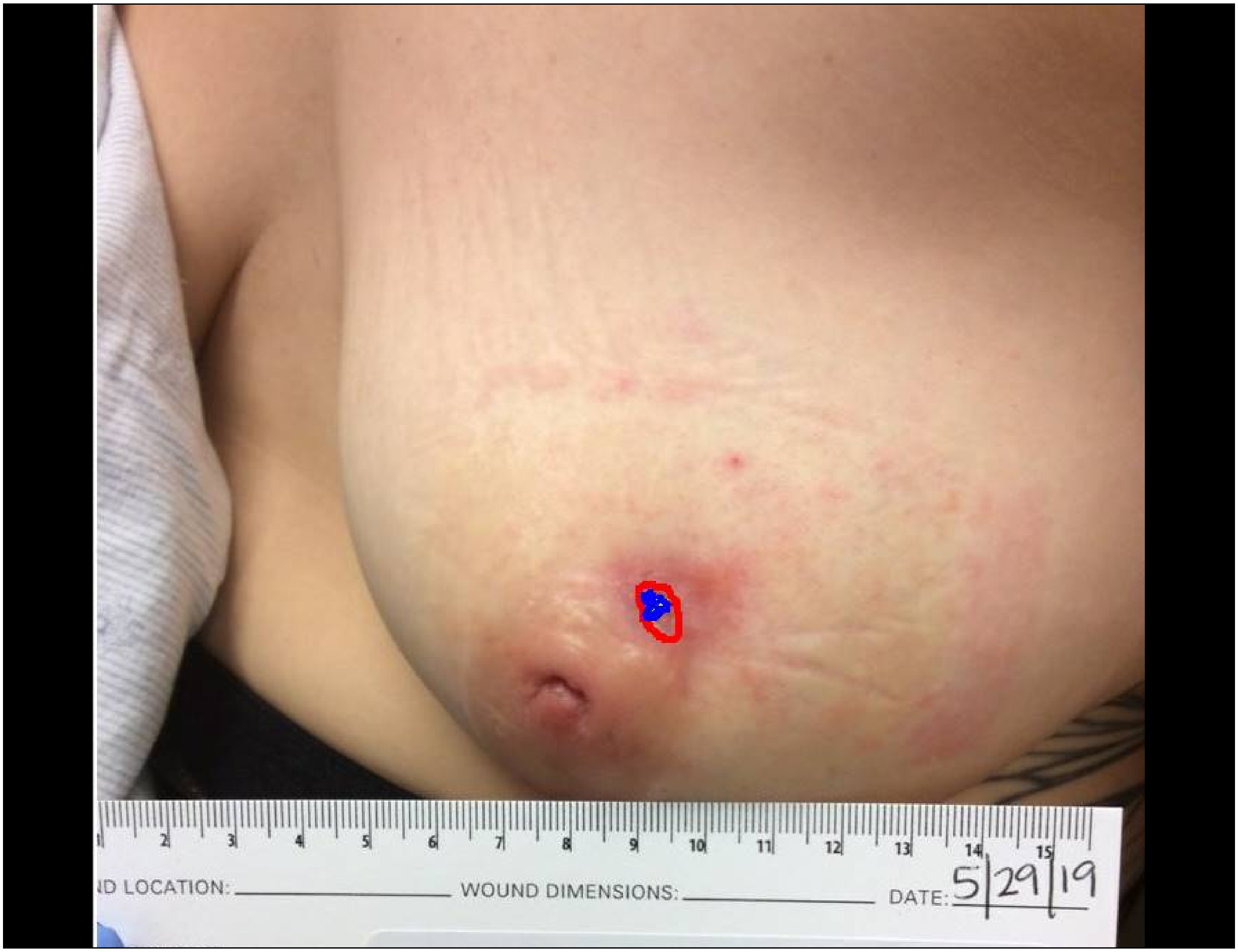

Tracing Data			
Tracer:	Wound Area (px²)	Ruler Calibration (px/cm)	Wound Area (cm²)
H1	96	49.0	0.04
H2	849	50.0	0.34
AI	142	37.7	0.10

Tracing Comparisons				
Difference Metric:	Human-Human		Human-AI	
	H1(ref) H2(test)	H2(ref) H1(test)	H1(ref) AI(test)	H2(ref) AI(test)
False Negative Area (%)	0.0	88.7	28.1	83.3
False Positive Area (%)	784.4	0.0	76.0	0.0
|Relative Error (%)|	784.4	88.7	47.9	83.3

Blinded Attending Surgeon Review						
Reviewer	PGT Estimate	H1 meets definition?	H2 meets definition?	AI meets definition?	Which is AI?	Which is most accurate?
1	90	Yes	Yes	Yes	AI	H1
2	100	Yes	Yes	Yes	AI	H1
3	100	Yes	No	Yes	H1	AI

© 2021 Howell RS et al. *JAMA Network Open.*

Wound EMR Information								
**Sequential Number**	**Gender**	**Age**	**Wound Type**	**Wound Location**	**Wound Length (cm)**	**Wound Width (cm)**	**Wound Depth (cm)**	**Wound Area (cm^2)**
61	F	33	Diabetic	Right first toe				

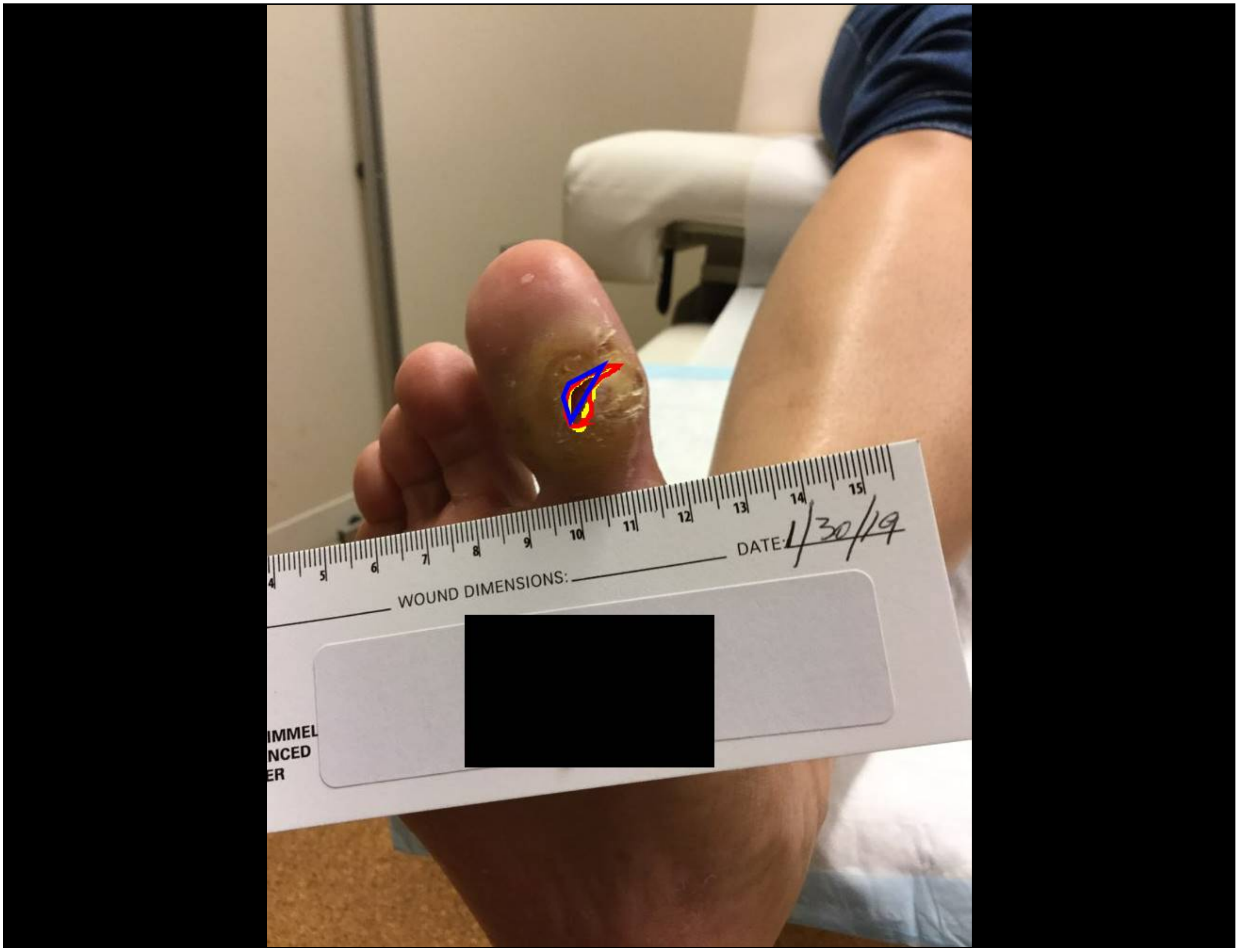

Tracing Data			
**Tracer:**	**Wound Area (px^2)**	**Ruler Calibration (px/cm)**	**Wound Area (cm^2)**
**H1**	1234	57.2	0.38
**H2**	1347	59.0	0.39
**AI**	881	54.2	0.30

Tracing Comparisons				
**Difference Metric:**	**Human-Human**		**Human-AI**	
	**H1(ref) H2(test)**	**H2(ref) H1(test)**	**H1(ref) AI(test)**	**H2(ref) AI(test)**
**False Negative Area (%)**	9.5	17.1	45.5	44.8
**False Positive Area (%)**	18.6	8.7	16.9	10.2
**|Relative Error (%)|**	9.2	8.4	28.6	34.6

Blinded Attending Surgeon Review						
**Reviewer**	**PGT Estimate**	**H1 meets definition?**	**H2 meets definition?**	**AI meets definition?**	**Which is AI?**	**Which is most accurate?**
**1**	90	Yes	Yes	Yes	H2	H1
**2**	30	Yes	Yes	Yes	H2	H1
**3**	100	Yes	Yes	No	AI	H2

© 2021 Howell RS et al. *JAMA Network Open.*

Wound EMR Information								
Sequential Number	Gender	Age	Wound Type	Wound Location	Wound Length (cm)	Wound Width (cm)	Wound Depth (cm)	Wound Area (cm²)
63	M	49	Trauma	Right anterior leg				

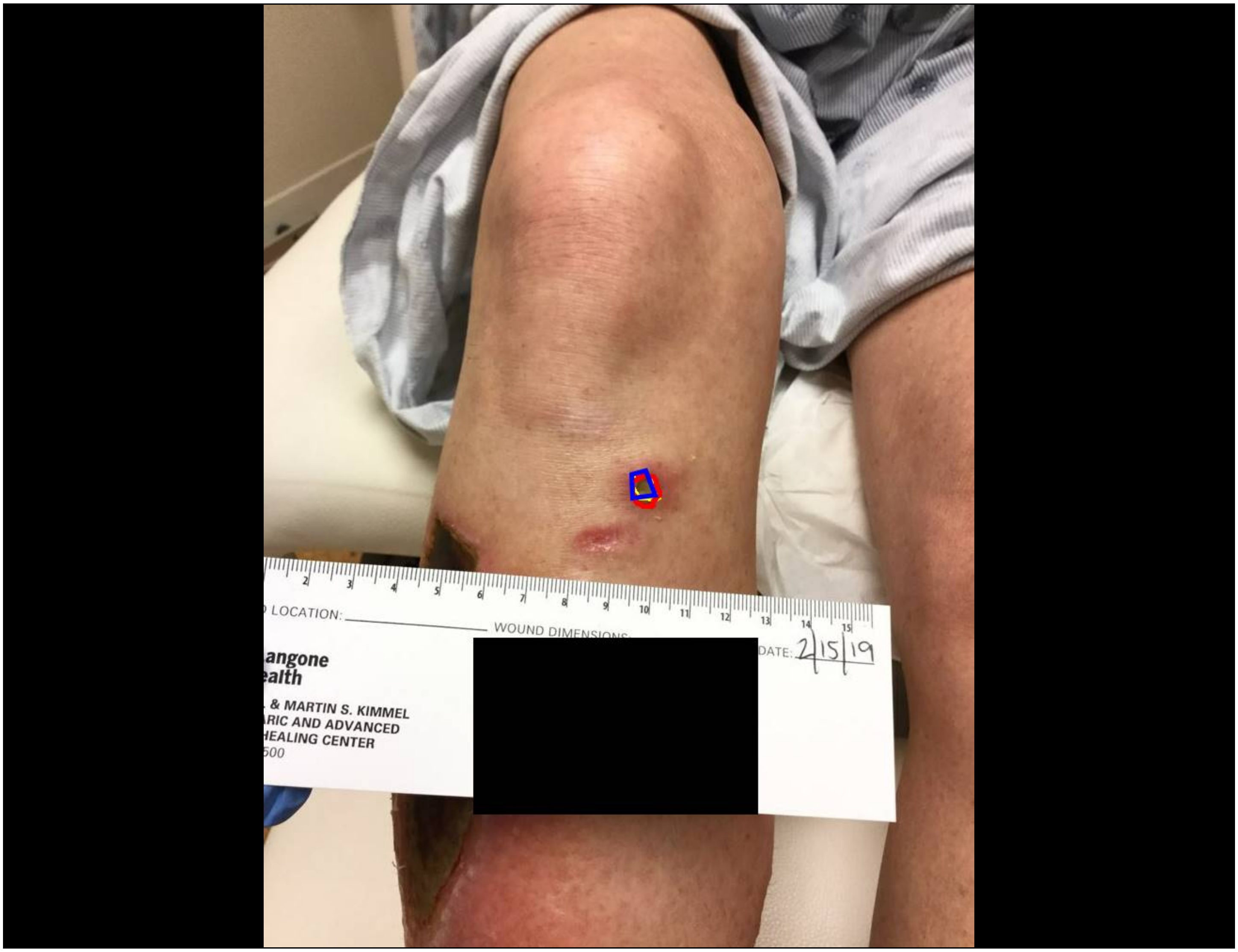

Tracing Data			
Tracer:	Wound Area (px²)	Ruler Calibration (px/cm)	Wound Area (cm²)
H1	7627	47.0	3.45
H2	8081	40.7	4.88
AI	6520	43.8	3.40

Tracing Comparisons				
Difference Metric:	Human-Human		Human-AI	
	H1(ref) H2(test)	H2(ref) H1(test)	H1(ref) AI(test)	H2(ref) AI(test)
False Negative Area (%)	4.4	9.8	18.8	21.9
False Positive Area (%)	10.3	4.1	4.3	2.6
|Relative Error (%)|	6.0	5.6	14.5	19.3

Blinded Attending Surgeon Review						
Reviewer	PGT Estimate	H1 meets definition?	H2 meets definition?	AI meets definition?	Which is AI?	Which is most accurate?
1	100	Yes	Yes	No	H2	H2
2	100	Yes	Yes	Yes	AI	H1
3	100	Yes	Yes	No	H2	H1

© 2021 Howell RS et al. *JAMA Network Open.*

Wound EMR Information								
Sequential Number	Gender	Age	Wound Type	Wound Location	Wound Length (cm)	Wound Width (cm)	Wound Depth (cm)	Wound Area (cm^2)
63	F	52	Unknown	Right lower leg				

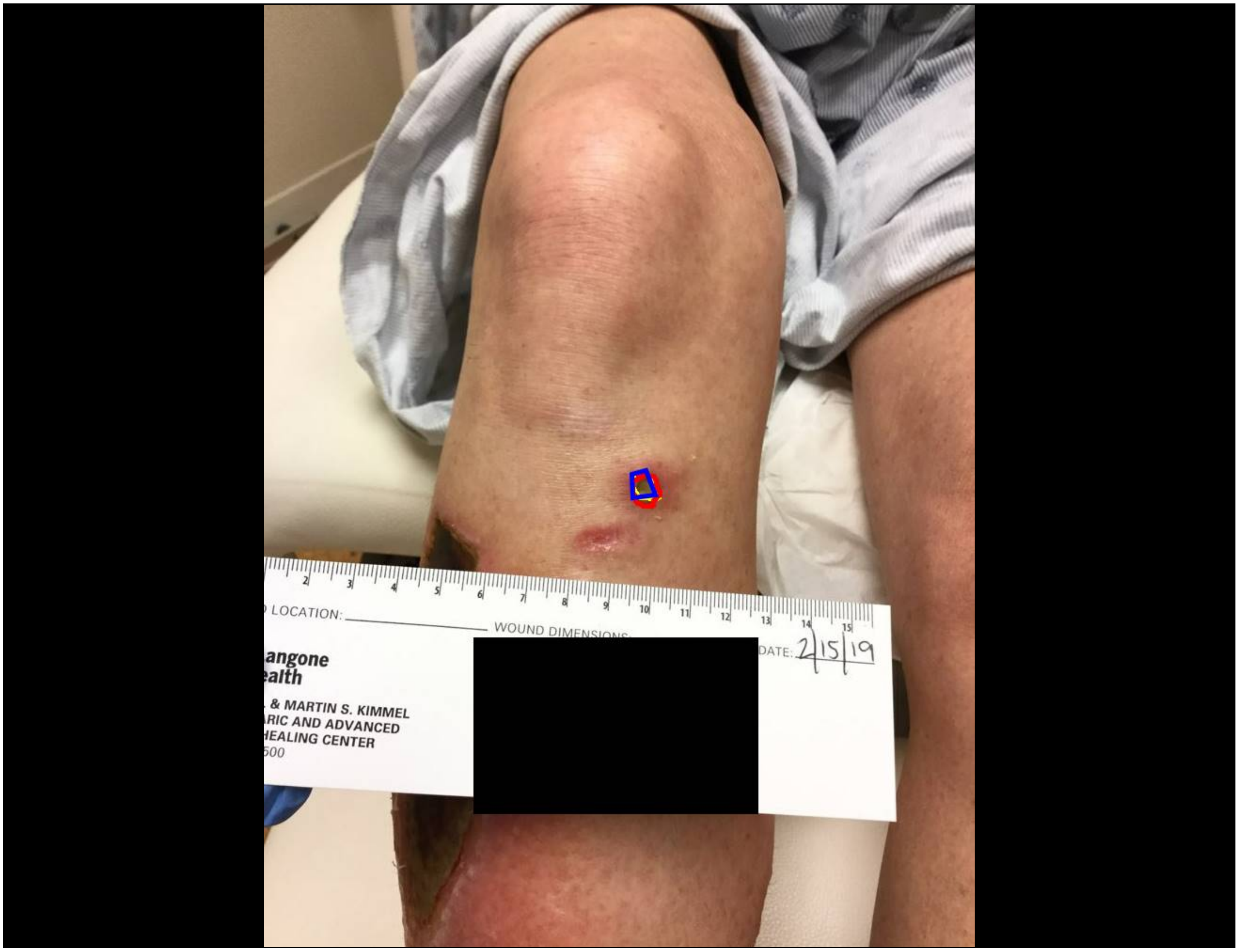

Tracing Data			
Tracer:	Wound Area (px^2)	Ruler Calibration (px/cm)	Wound Area (cm^2)
H1	532	43.3	0.28
H2	723	45.1	0.36
AI	533	36.5	0.40

Tracing Comparisons				
Difference Metric:	Human-Human		Human-AI	
	H1(ref) H2(test)	H2(ref) H1(test)	H1(ref) AI(test)	H2(ref) AI(test)
False Negative Area (%)	6.6	31.3	17.5	40.2
False Positive Area (%)	42.5	4.8	17.7	14.0
|Relative Error (%)|	35.9	26.4	0.2	26.3

Blinded Attending Surgeon Review						
Reviewer	PGT Estimate	H1 meets definition?	H2 meets definition?	AI meets definition?	Which is AI?	Which is most accurate?
1	0	No	No	No	H2	AI
2	0	Yes	Yes	Yes	H2	H1
3	0	Yes	Yes	No	AI	H1

© 2021 Howell RS et al. *JAMA Network Open.*

Wound EMR Information								
Sequential Number	Gender	Age	Wound Type	Wound Location	Wound Length (cm)	Wound Width (cm)	Wound Depth (cm)	Wound Area (cm²)
64	M	41	Pressure	Right posterior				

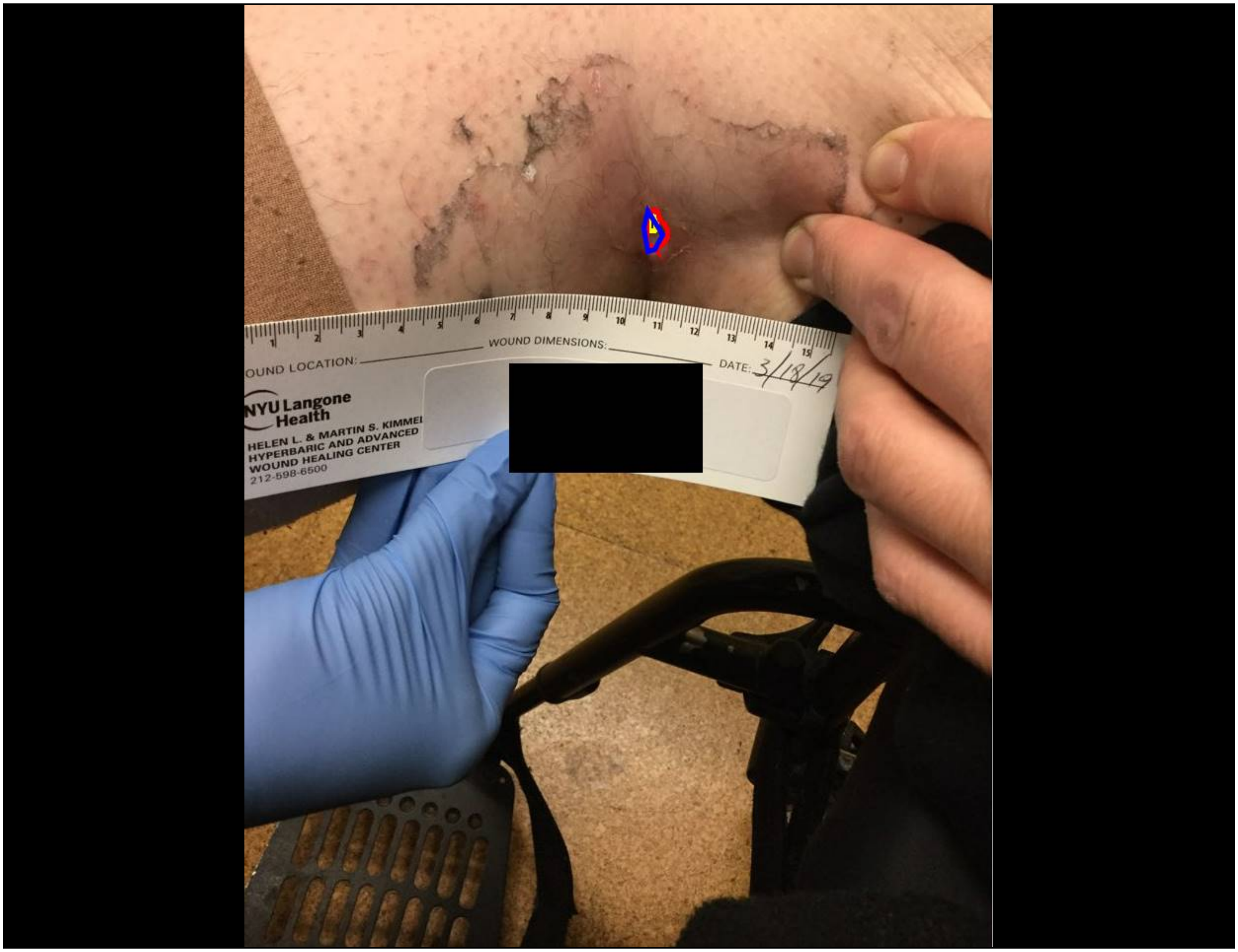

Tracing Data			
Tracer:	Wound Area (px²)	Ruler Calibration (px/cm)	Wound Area (cm²)
H1	111	39.0	0.07
H2	595	39.1	0.39
AI	448	33.5	0.40

Tracing Comparisons				
Difference Metric:	Human-Human		Human-AI	
	H1(ref) H2(test)	H2(ref) H1(test)	H1(ref) AI(test)	H2(ref) AI(test)
False Negative Area (%)	0.0	81.3	26.1	40.0
False Positive Area (%)	436.0	0.0	329.7	15.3
|Relative Error (%)|	436.0	81.3	303.6	24.7

Blinded Attending Surgeon Review						
Reviewer	PGT Estimate	H1 meets definition?	H2 meets definition?	AI meets definition?	Which is AI?	Which is most accurate?
1	20	No	Yes	Yes	H1	H1
2	50	Yes	Yes	Yes	H1	H2
3	0	No	Yes	Yes	H1	AI

© 2021 Howell RS et al. *JAMA Network Open.*

Wound EMR Information								
Sequential Number	Gender	Age	Wound Type	Wound Location	Wound Length (cm)	Wound Width (cm)	Wound Depth (cm)	Wound Area (cm^2)
65	F	22	Surgical	Left breast				

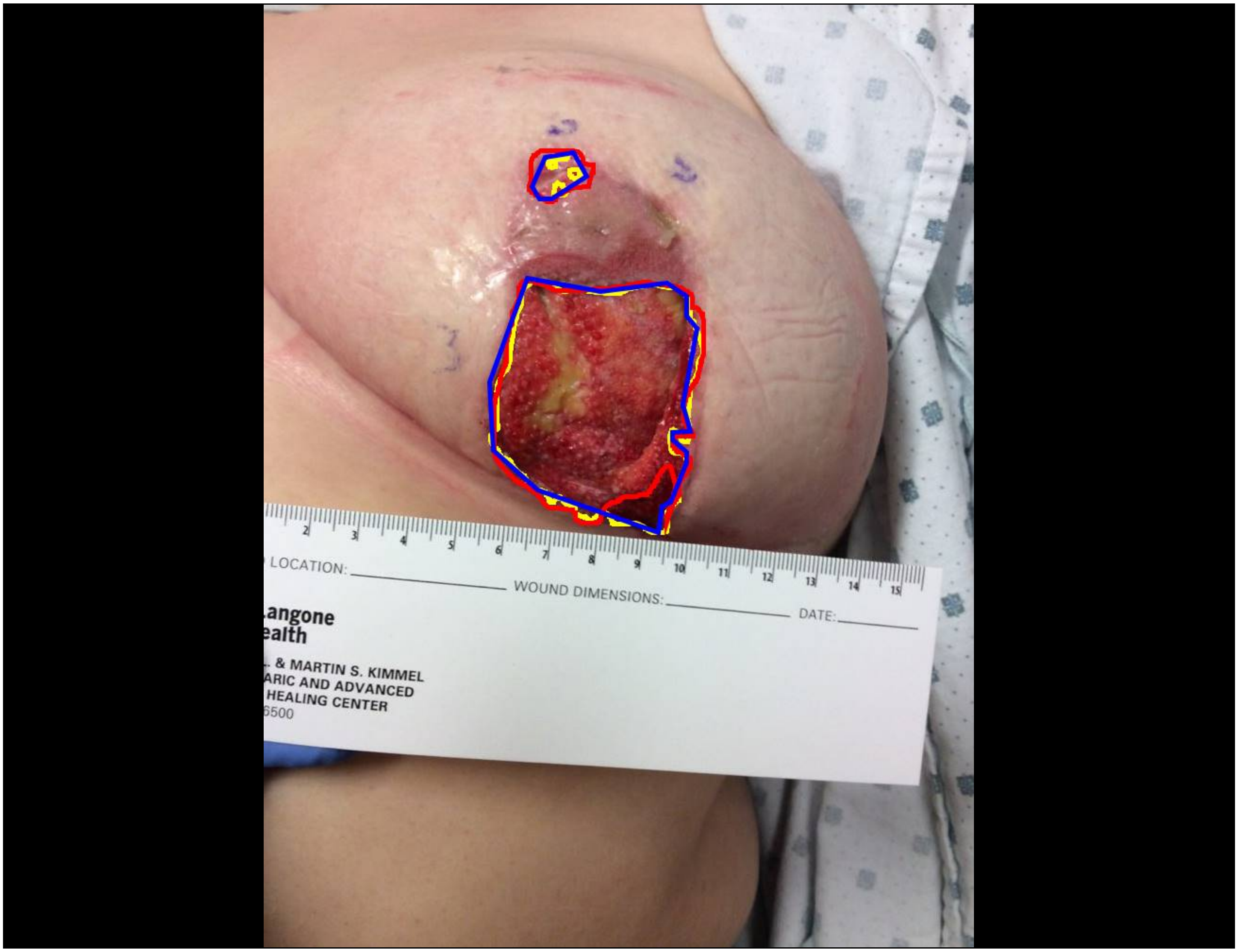

Tracing Data			
Tracer:	Wound Area (px^2)	Ruler Calibration (px/cm)	Wound Area (cm^2)
H1	45414	48.7	19.12
H2	47165	48.8	19.82
AI	46845	48.4	20.00

Tracing Comparisons				
Difference Metric:	Human-Human		Human-AI	
	H1(ref) H2(test)	H2(ref) H1(test)	H1(ref) AI(test)	H2(ref) AI(test)
False Negative Area (%)	6.7	10.1	3.6	7.8
False Positive Area (%)	10.5	6.4	6.8	7.1
|Relative Error (%)|	3.9	3.7	3.2	0.7

Blinded Attending Surgeon Review						
Reviewer	PGT Estimate	H1 meets definition?	H2 meets definition?	AI meets definition?	Which is AI?	Which is most accurate?
1	80	Yes	Yes	Yes	H2	H1
2	90	Yes	Yes	Yes	H2	AI
3	80	No	Yes	Yes	H2	AI

© 2021 Howell RS et al. *JAMA Network Open.*

Wound EMR Information								
Sequential Number	Gender	Age	Wound Type	Wound Location	Wound Length (cm)	Wound Width (cm)	Wound Depth (cm)	Wound Area (cm^2)
67	F	69	Trauma	Right anterior leg				

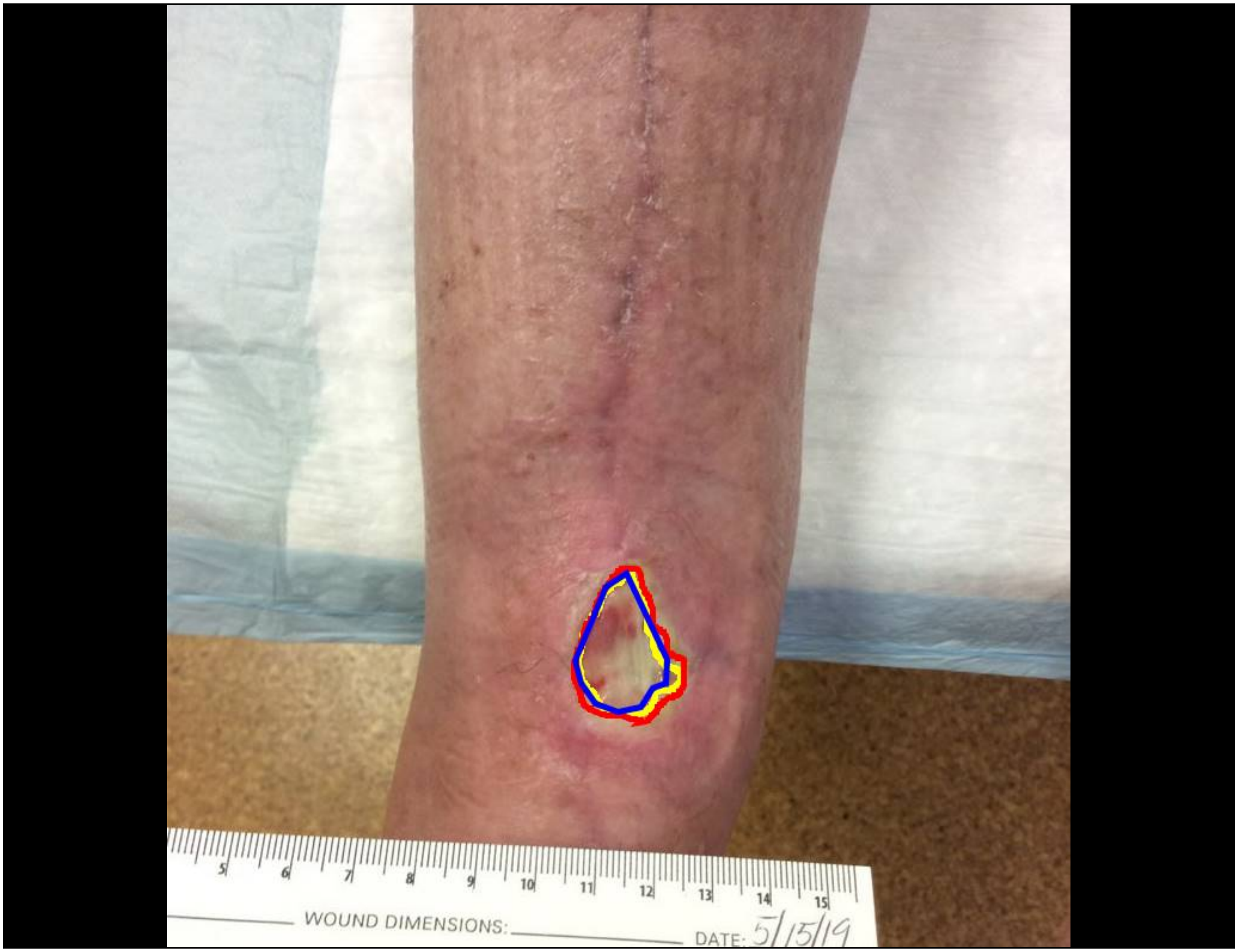

Tracing Data			
Tracer:	Wound Area (px^2)	Ruler Calibration (px/cm)	Wound Area (cm^2)
H1	5985	49.4	2.45
H2	7633	39.7	4.85
AI	5708	48.8	2.40

Tracing Comparisons				
Difference Metric:	Human-Human		Human-AI	
	H1(ref) H2(test)	H2(ref) H1(test)	H1(ref) AI(test)	H2(ref) AI(test)
False Negative Area (%)	0.1	21.7	10.4	25.2
False Positive Area (%)	27.7	0.1	5.8	0.0
|Relative Error (%)|	27.5	21.6	4.6	25.2

Blinded Attending Surgeon Review						
Reviewer	PGT Estimate	H1 meets definition?	H2 meets definition?	AI meets definition?	Which is AI?	Which is most accurate?
1	10	Yes	Yes	Yes	H2	H2
2	50	Yes	Yes	Yes	AI	H2
3	40	Yes	Yes	Yes	H1	H1

© 2021 Howell RS et al. *JAMA Network Open.*

Wound EMR Information								
Sequential Number	Gender	Age	Wound Type	Wound Location	Wound Length (cm)	Wound Width (cm)	Wound Depth (cm)	Wound Area (cm^2)
70	F	73		Right elbow				

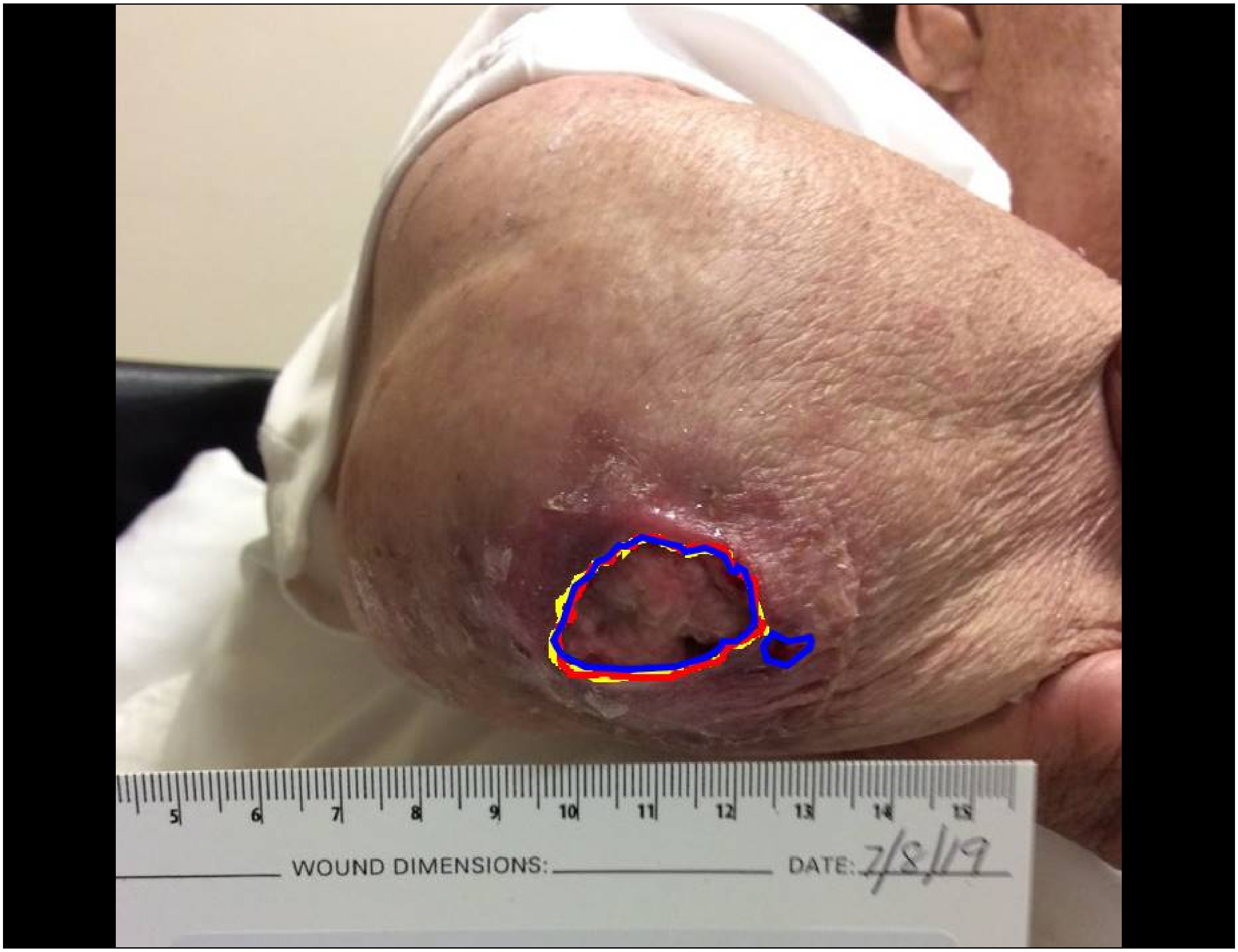

Tracing Data			
Tracer:	Wound Area (px^2)	Ruler Calibration (px/cm)	Wound Area (cm^2)
H1	11976	59.2	3.42
H2	11892	52.3	4.34
AI	11493	57.3	3.50

Tracing Comparisons				
Difference Metric:	Human-Human		Human-AI	
	H1(ref) H2(test)	H2(ref) H1(test)	H1(ref) AI(test)	H2(ref) AI(test)
False Negative Area (%)	4.6	3.9	9.2	9.5
False Positive Area (%)	3.9	4.6	5.2	6.1
|Relative Error (%)|	0.7	0.7	4.0	3.4

Blinded Attending Surgeon Review						
Reviewer	PGT Estimate	H1 meets definition?	H2 meets definition?	AI meets definition?	Which is AI?	Which is most accurate?
1	20	No	No	Yes	H1	H1
2	80	Yes	Yes	Yes	H2	H1
3	20	Yes	Yes	Yes	H1	AI

© 2021 Howell RS et al. *JAMA Network Open.*

Wound EMR Information								
Sequential Number	Gender	Age	Wound Type	Wound Location	Wound Length (cm)	Wound Width (cm)	Wound Depth (cm)	Wound Area (cm^2)
71	F	78	Trauma	Left anterior leg				

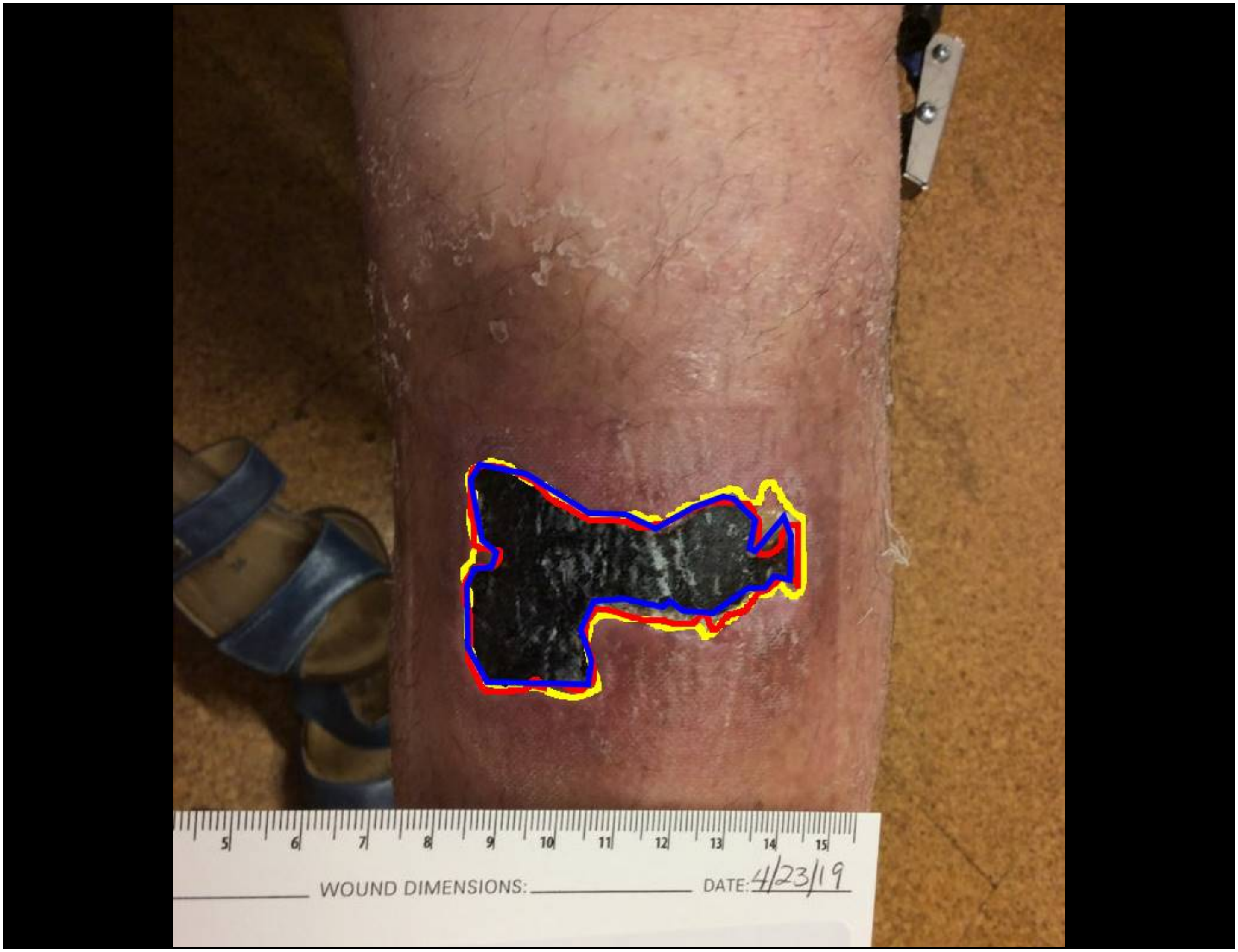

Tracing Data			
Tracer:	Wound Area (px^2)	Ruler Calibration (px/cm)	Wound Area (cm^2)
H1	33908	48.0	14.72
H2	30001	48.0	13.02
AI	27949	50.9	10.80

Tracing Comparisons				
Difference Metric:	Human-Human		Human-AI	
	H1(ref) H2(test)	H2(ref) H1(test)	H1(ref) AI(test)	H2(ref) AI(test)
False Negative Area (%)	12.9	1.5	18.2	10.5
False Positive Area (%)	1.4	14.6	0.7	3.7
|Relative Error (%)|	11.5	13.0	17.6	6.8

Blinded Attending Surgeon Review						
Reviewer	PGT Estimate	H1 meets definition?	H2 meets definition?	AI meets definition?	Which is AI?	Which is most accurate?
1	0	Yes	No	No	H2	H1
2	0	Yes	Yes	Yes	AI	AI
3	0	No	Yes	Yes	H2	H1

© 2021 Howell RS et al. *JAMA Network Open.*

Wound EMR Information								
Sequential Number	Gender	Age	Wound Type	Wound Location	Wound Length (cm)	Wound Width (cm)	Wound Depth (cm)	Wound Area (cm^2)
72	F	51	Surgical	Right breast	16.0	3.0		48.00

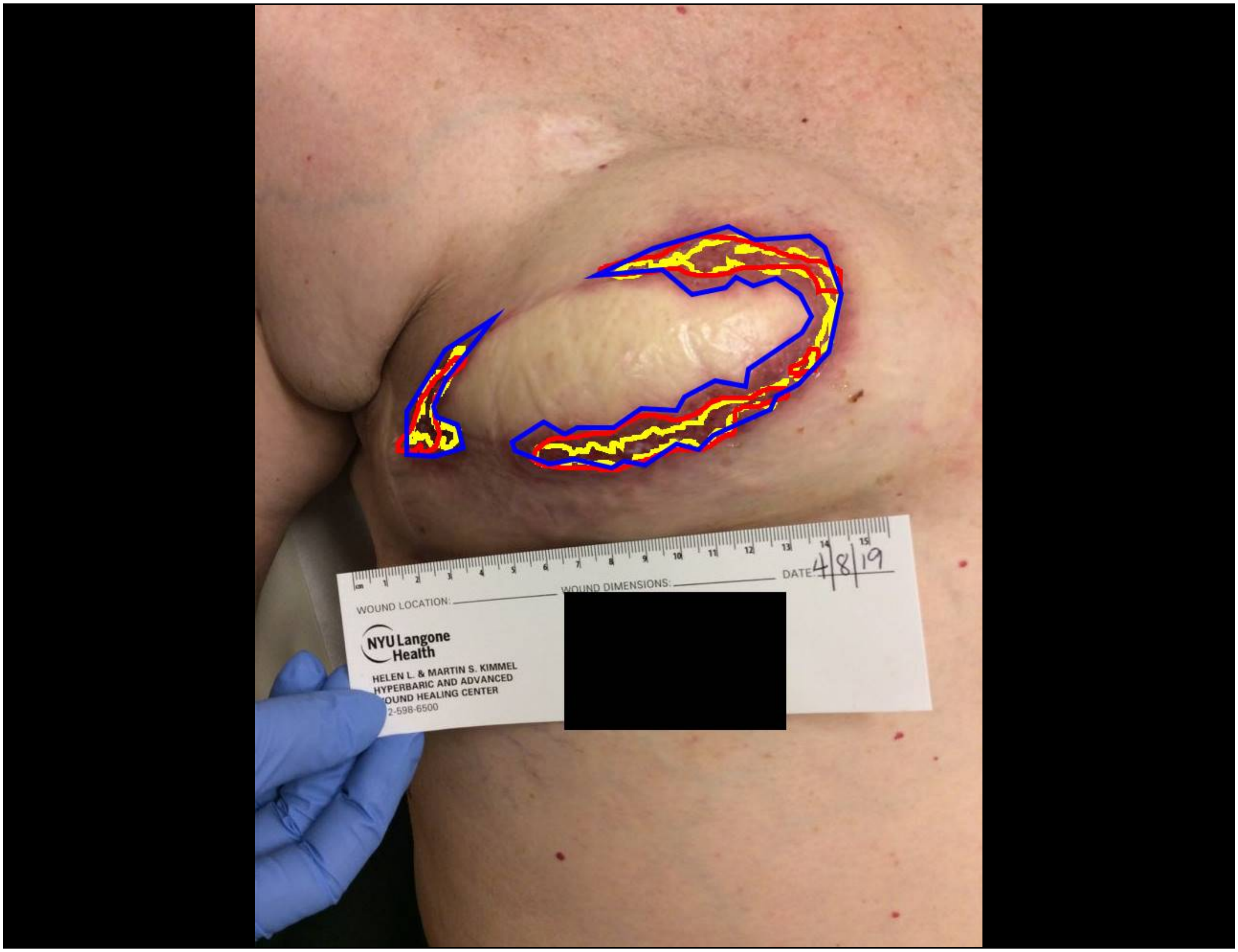

Tracing Data			
Tracer:	Wound Area (px^2)	Ruler Calibration (px/cm)	Wound Area (cm^2)
H1	11407	46.0	5.39
H2	16376	35.8	12.81
AI	29784	33.7	26.20

Tracing Comparisons				
Difference Metric:	Human-Human		Human-AI	
	H1(ref) H2(test)	H2(ref) H1(test)	H1(ref) AI(test)	H2(ref) AI(test)
False Negative Area (%)	14.3	40.3	2.6	7.7
False Positive Area (%)	57.9	10.0	163.8	89.6
|Relative Error (%)|	43.6	30.3	161.1	81.9

Blinded Attending Surgeon Review						
Reviewer	PGT Estimate	H1 meets definition?	H2 meets definition?	AI meets definition?	Which is AI?	Which is most accurate?
1	60	Yes	Yes	Yes	H1	H1
2	100	Yes	Yes	Yes	H2	H2
3	100	No	No	Yes	H2	AI

© 2021 Howell RS et al. *JAMA Network Open.*

Wound EMR Information								
Sequential Number	Gender	Age	Wound Type	Wound Location	Wound Length (cm)	Wound Width (cm)	Wound Depth (cm)	Wound Area (cm^2)
73	F	38	Venous	Left medial ankle				

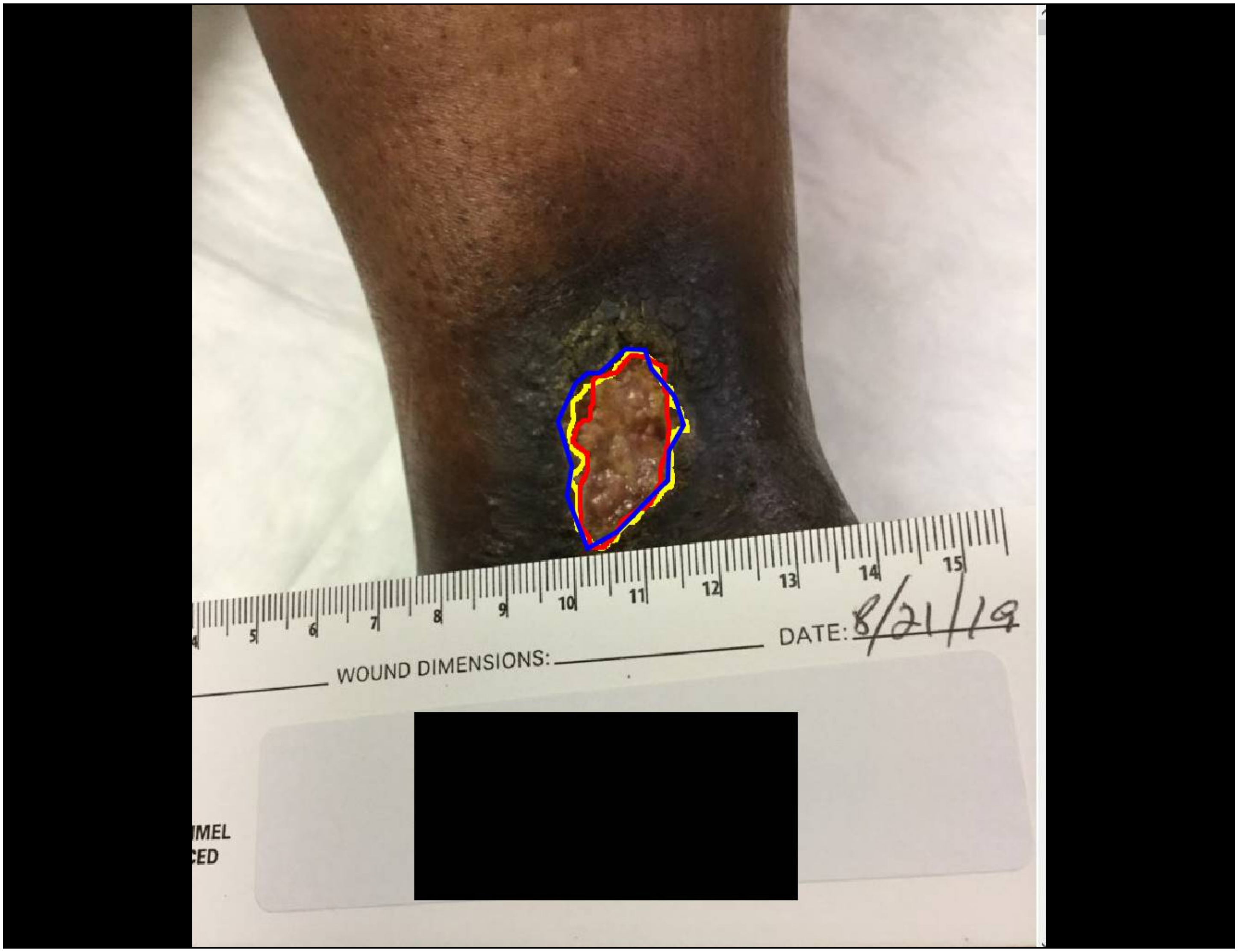

Tracing Data			
Tracer:	Wound Area (px^2)	Ruler Calibration (px/cm)	Wound Area (cm^2)
H1	17200	76.7	2.92
H2	14570	79.1	2.33
AI	18592	80.1	2.90

Tracing Comparisons				
Difference Metric:	Human-Human		Human-AI	
	H1(ref) H2(test)	H2(ref) H1(test)	H1(ref) AI(test)	H2(ref) AI(test)
False Negative Area (%)	16.7	1.6	4.1	2.6
False Positive Area (%)	1.4	19.7	12.2	30.2
|Relative Error (%)|	15.3	18.1	8.1	27.6

Blinded Attending Surgeon Review						
Reviewer	PGT Estimate	H1 meets definition?	H2 meets definition?	AI meets definition?	Which is AI?	Which is most accurate?
**1**	10	Yes	Yes	Yes	H1	H1
**2**	30	Yes	Yes	Yes	AI	H1
**3**	0	Yes	No	No	H1	H2

© 2021 Howell RS et al. *JAMA Network Open.*

Wound EMR Information								
Sequential Number	Gender	Age	Wound Type	Wound Location	Wound Length (cm)	Wound Width (cm)	Wound Depth (cm)	Wound Area (cm^2)
74	F	78	Trauma	Left medial ankle				

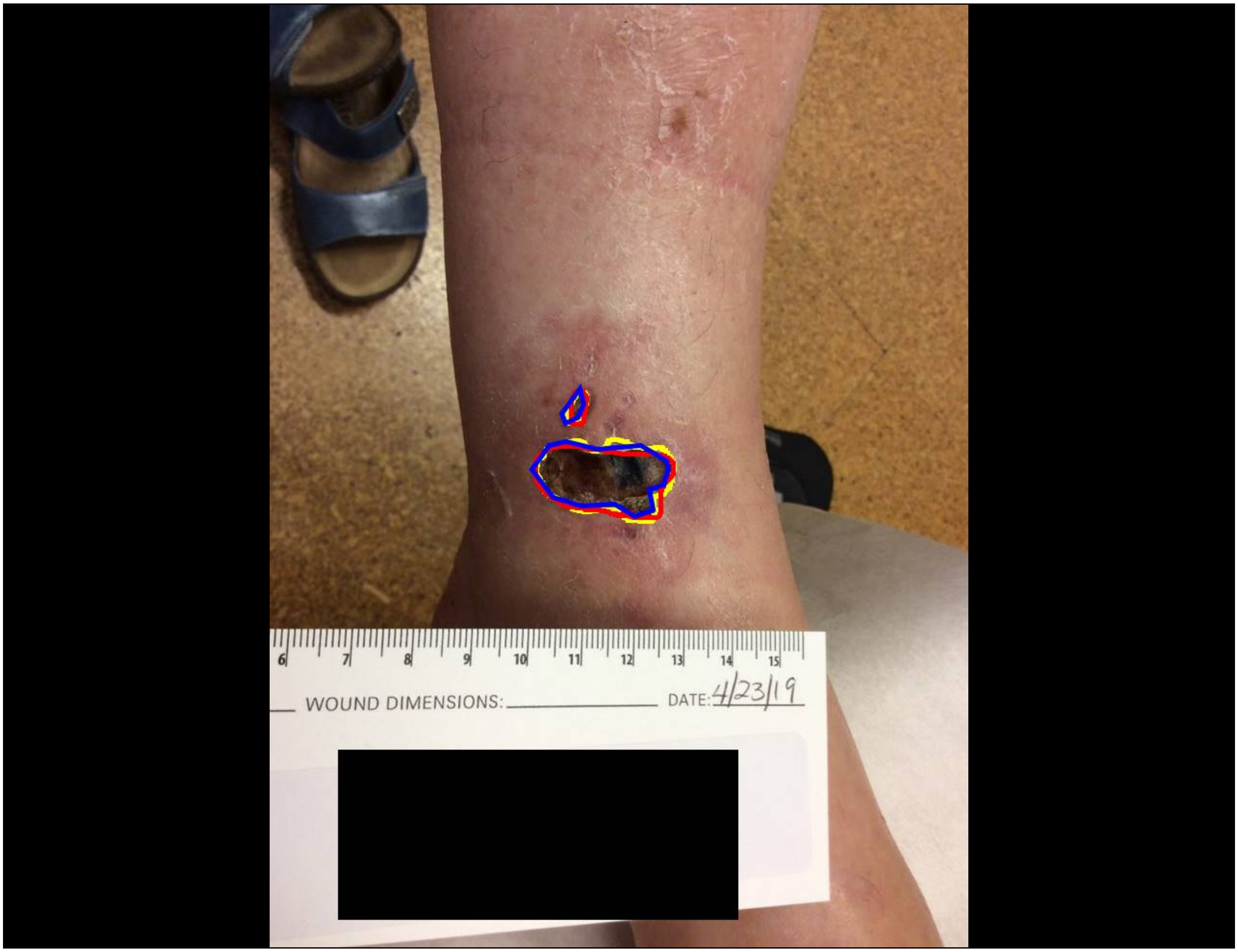

Tracing Data			
Tracer:	Wound Area (px^2)	Ruler Calibration (px/cm)	Wound Area (cm^2)
H1	9240	53.9	3.18
H2	8590	44.0	4.43
AI	7998	52.5	2.90

Tracing Comparisons				
Difference Metric:	Human-Human		Human-AI	
	H1(ref) H2(test)	H2(ref) H1(test)	H1(ref) AI(test)	H2(ref) AI(test)
False Negative Area (%)	11.6	4.9	15.5	13.7
False Positive Area (%)	4.5	12.4	2.1	6.8
|Relative Error (%)|	7.0	7.6	13.4	6.9

Blinded Attending Surgeon Review						
Reviewer	PGT Estimate	H1 meets definition?	H2 meets definition?	AI meets definition?	Which is AI?	Which is most accurate?
1	0	Yes	Yes	Yes	H2	H1
2	0	Yes	Yes	Yes	AI	AI
3	0	Yes	Yes	Yes	H1	H2

© 2021 Howell RS et al. *JAMA Network Open.*

Wound EMR Information								
Sequential Number	Gender	Age	Wound Type	Wound Location	Wound Length (cm)	Wound Width (cm)	Wound Depth (cm)	Wound Area (cm^2)
75	F	42	Surgical	Left knee				

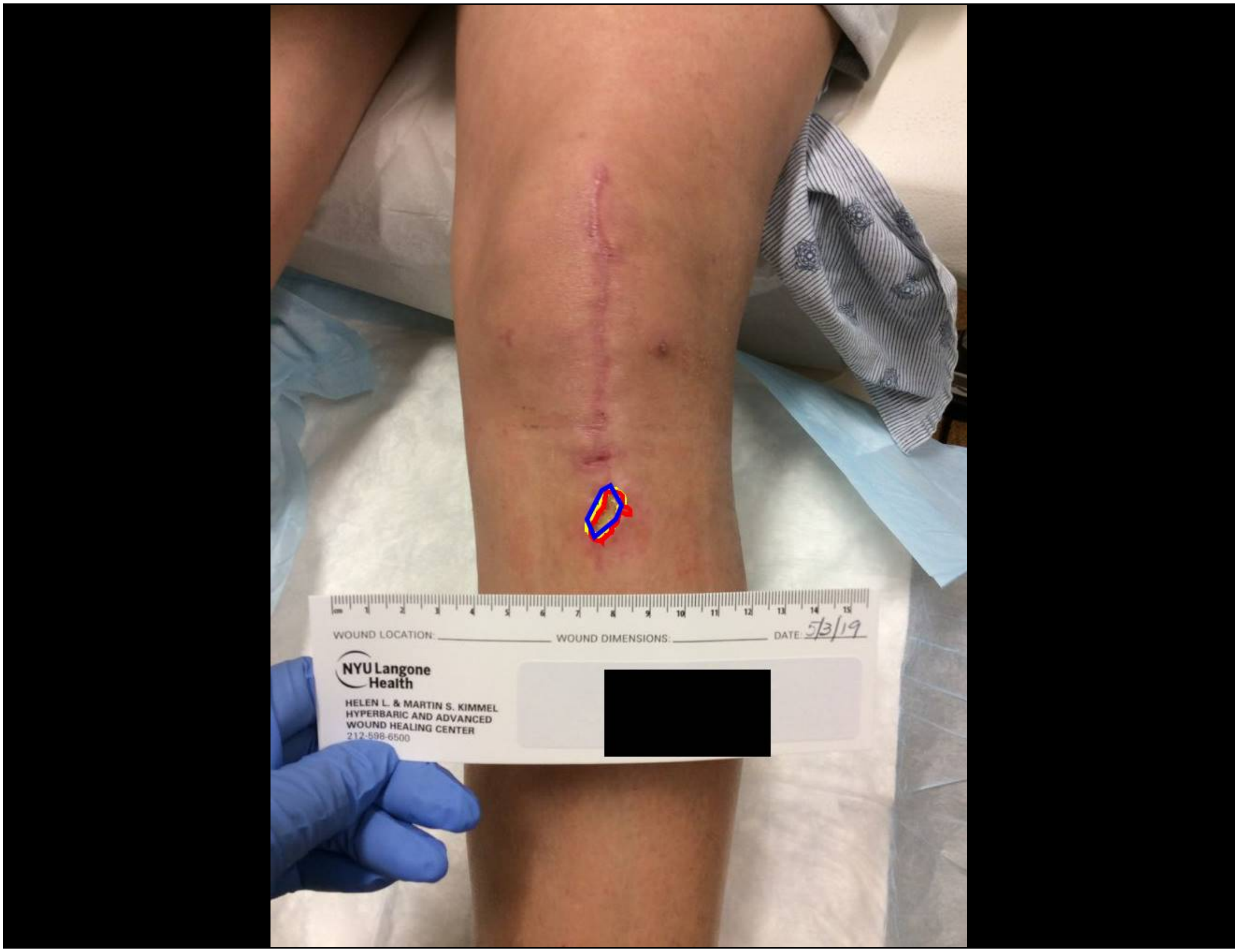

Tracing Data			
Tracer:	Wound Area (px^2)	Ruler Calibration (px/cm)	Wound Area (cm^2)
H1	1164	37.7	0.82
H2	1100	29.5	1.26
AI	1140	35.6	0.90

Tracing Comparisons				
Difference Metric:	Human-Human		Human-AI	
	H1(ref) H2(test)	H2(ref) H1(test)	H1(ref) AI(test)	H2(ref) AI(test)
False Negative Area (%)	19.0	14.3	19.4	28.1
False Positive Area (%)	13.5	20.1	17.4	31.7
|Relative Error (%)|	5.5	5.8	2.1	3.6

Blinded Attending Surgeon Review						
Reviewer	PGT Estimate	H1 meets definition?	H2 meets definition?	AI meets definition?	Which is AI?	Which is most accurate?
1	10	No	No	Yes	H2	H1
2	10	Yes	Yes	Yes	H1	H2
3	10	No	No	Yes	H1	AI

© 2021 Howell RS et al. *JAMA Network Open.*

Wound EMR Information								
Sequential Number	Gender	Age	Wound Type	Wound Location	Wound Length (cm)	Wound Width (cm)	Wound Depth (cm)	Wound Area (cm^2)
76	M	58	Pressure	Sacral	8.0	11.0		88.00

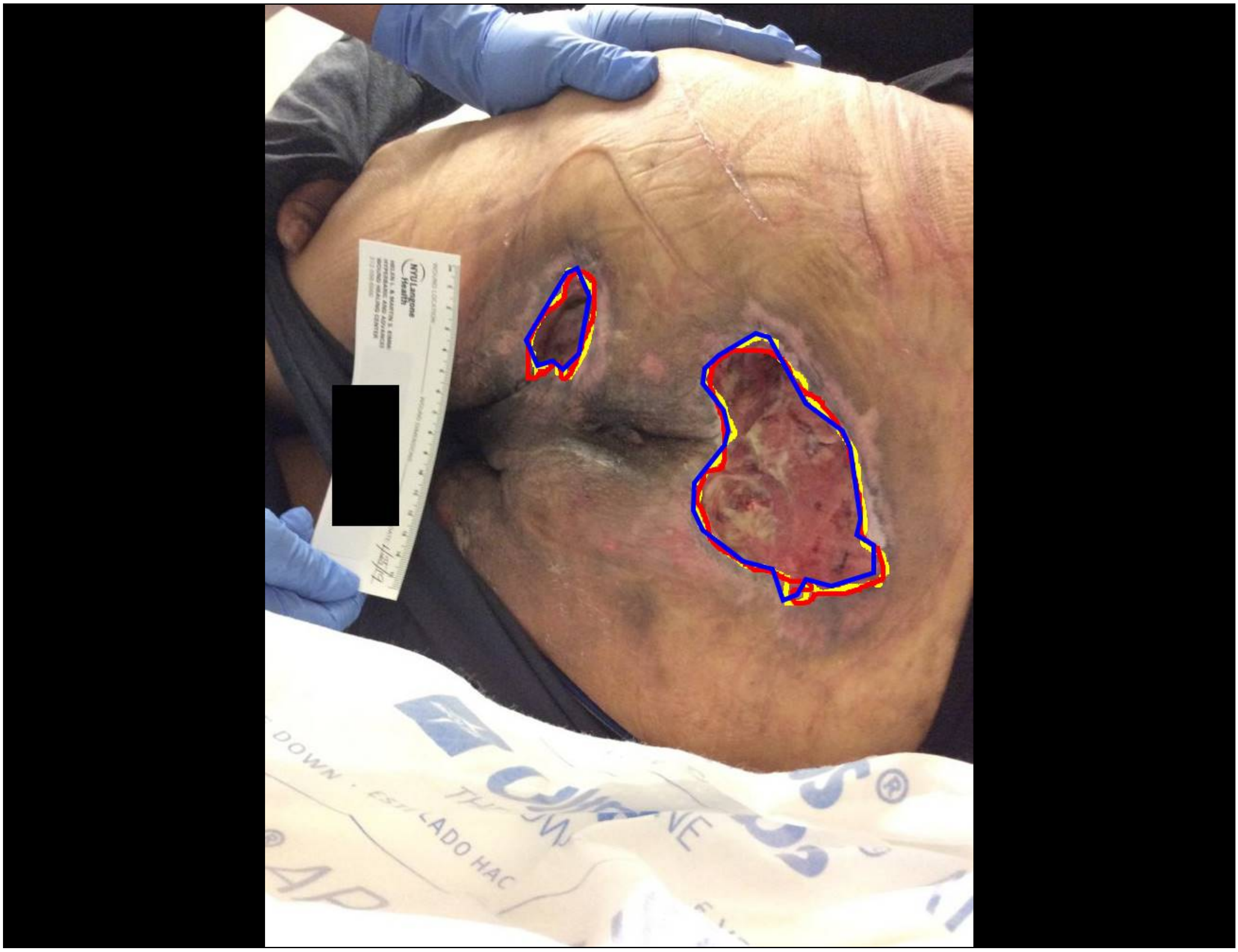

Tracing Data			
Tracer:	Wound Area (px^2)	Ruler Calibration (px/cm)	Wound Area (cm^2)
H1	38502	22.0	79.32
H2	37745	22.2	76.46
AI	36917	23.5	66.90

Tracing Comparisons				
Difference Metric:	Human-Human		Human-AI	
	H1(ref) H2(test)	H2(ref) H1(test)	H1(ref) AI(test)	H2(ref) AI(test)
False Negative Area (%)	6.2	4.3	7.3	8.5
False Positive Area (%)	4.3	6.3	3.2	6.3
|Relative Error (%)|	2.0	2.0	4.1	2.2

Blinded Attending Surgeon Review						
Reviewer	PGT Estimate	H1 meets definition?	H2 meets definition?	AI meets definition?	Which is AI?	Which is most accurate?
1	50	Yes	Yes	Yes	H1	H2
2	90	Yes	Yes	Yes	H1	H1
3	70	Yes	Yes	Yes	H2	H2

© 2021 Howell RS et al. *JAMA Network Open.*

Wound EMR Information								
Sequential Number	Gender	Age	Wound Type	Wound Location	Wound Length (cm)	Wound Width (cm)	Wound Depth (cm)	Wound Area (cm^2)
77	F	17	Pressure	Left ischium	3.0	2.0	7.0	6.00

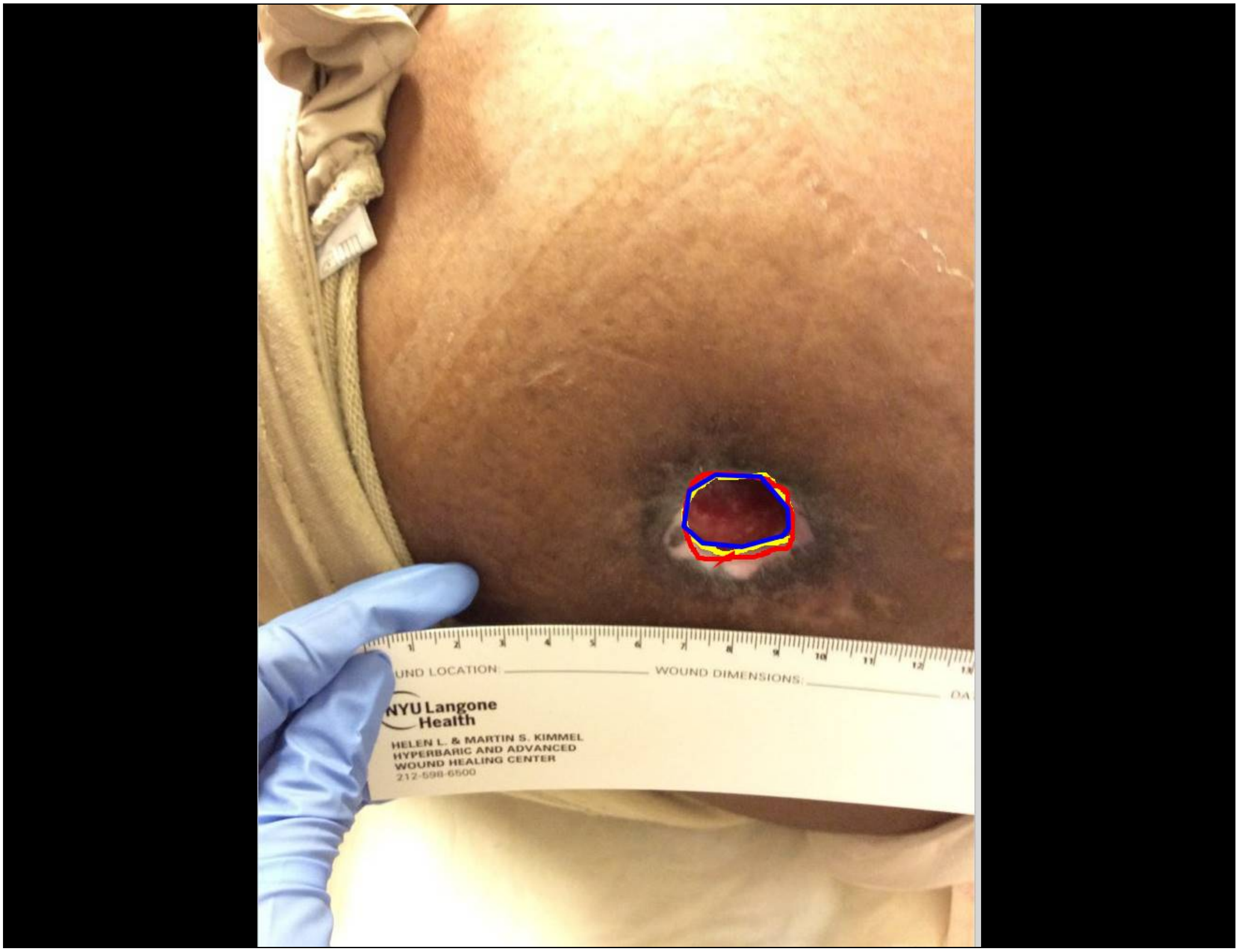

Tracing Data			
Tracer:	Wound Area (px^2)	Ruler Calibration (px/cm)	Wound Area (cm^2)
H1	7598	48.9	3.18
H2	8966	49.6	3.65
AI	6821	47.7	3.00

Tracing Comparisons				
Difference Metric:	Human-Human		Human-AI	
	H1(ref) H2(test)	H2(ref) H1(test)	H1(ref) AI(test)	H2(ref) AI(test)
False Negative Area (%)	0.8	15.9	11.4	24.0
False Positive Area (%)	18.8	0.7	1.1	0.0
|Relative Error (%)|	18.0	15.3	10.2	23.9

Blinded Attending Surgeon Review						
Reviewer	PGT Estimate	H1 meets definition?	H2 meets definition?	AI meets definition?	Which is AI?	Which is most accurate?
1	100	Yes	Yes	Yes	H2	AI
2	100	Yes	Yes	Yes	H2	AI
3	100	No	No	Yes	AI	AI

© 2021 Howell RS et al. *JAMA Network Open.*

Wound EMR Information								
Sequential Number	Gender	Age	Wound Type	Wound Location	Wound Length (cm)	Wound Width (cm)	Wound Depth (cm)	Wound Area (cm^2)
78	F	52	Unknown	Right medial leg	4.5	2.5		11.25

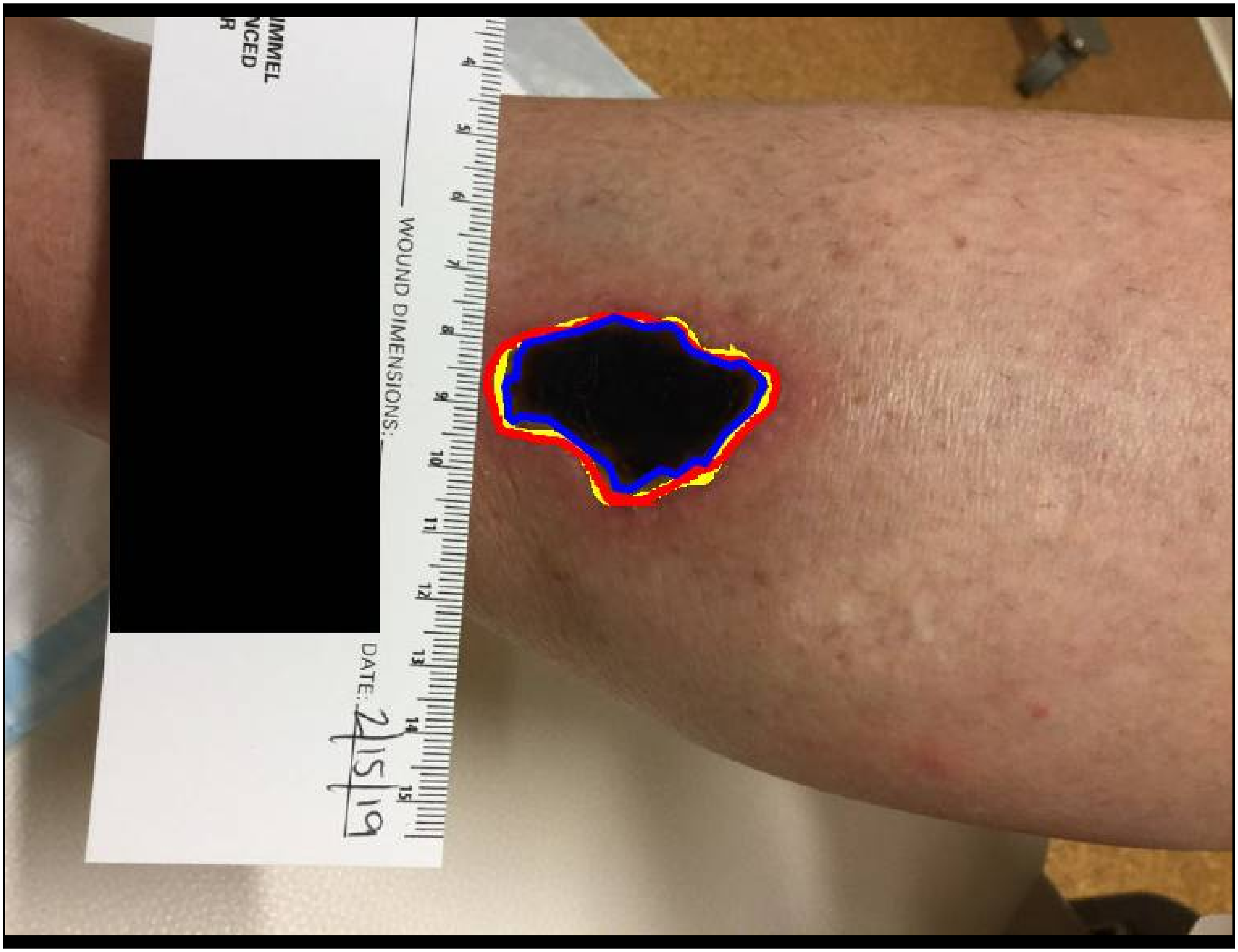

Tracing Data			
Tracer:	Wound Area (px^2)	Ruler Calibration (px/cm)	Wound Area (cm^2)
H1	12689	40.7	7.67
H2	13195	41.8	7.57
AI	10272	40.7	6.20

Tracing Comparisons				
Difference Metric:	Human-Human		Human-AI	
	H1(ref) H2(test)	H2(ref) H1(test)	H1(ref) AI(test)	H2(ref) AI(test)
False Negative Area (%)	2.3	6.0	19.0	22.2
False Positive Area (%)	6.2	2.2	0.0	0.0
|Relative Error (%)|	4.0	3.8	19.0	22.2

Blinded Attending Surgeon Review						
Reviewer	PGT Estimate	H1 meets definition?	H2 meets definition?	AI meets definition?	Which is AI?	Which is most accurate?
1	0	Yes	Yes	Yes	H2	AI
2	100	Yes	Yes	Yes	AI	H2
3	0	Yes	Yes	No	H2	H2

© 2021 Howell RS et al. *JAMA Network Open.*

Wound EMR Information								
Sequential Number	Gender	Age	Wound Type	Wound Location	Wound Length (cm)	Wound Width (cm)	Wound Depth (cm)	Wound Area (cm^2)
79	F	84	Pressure	Sacrum				

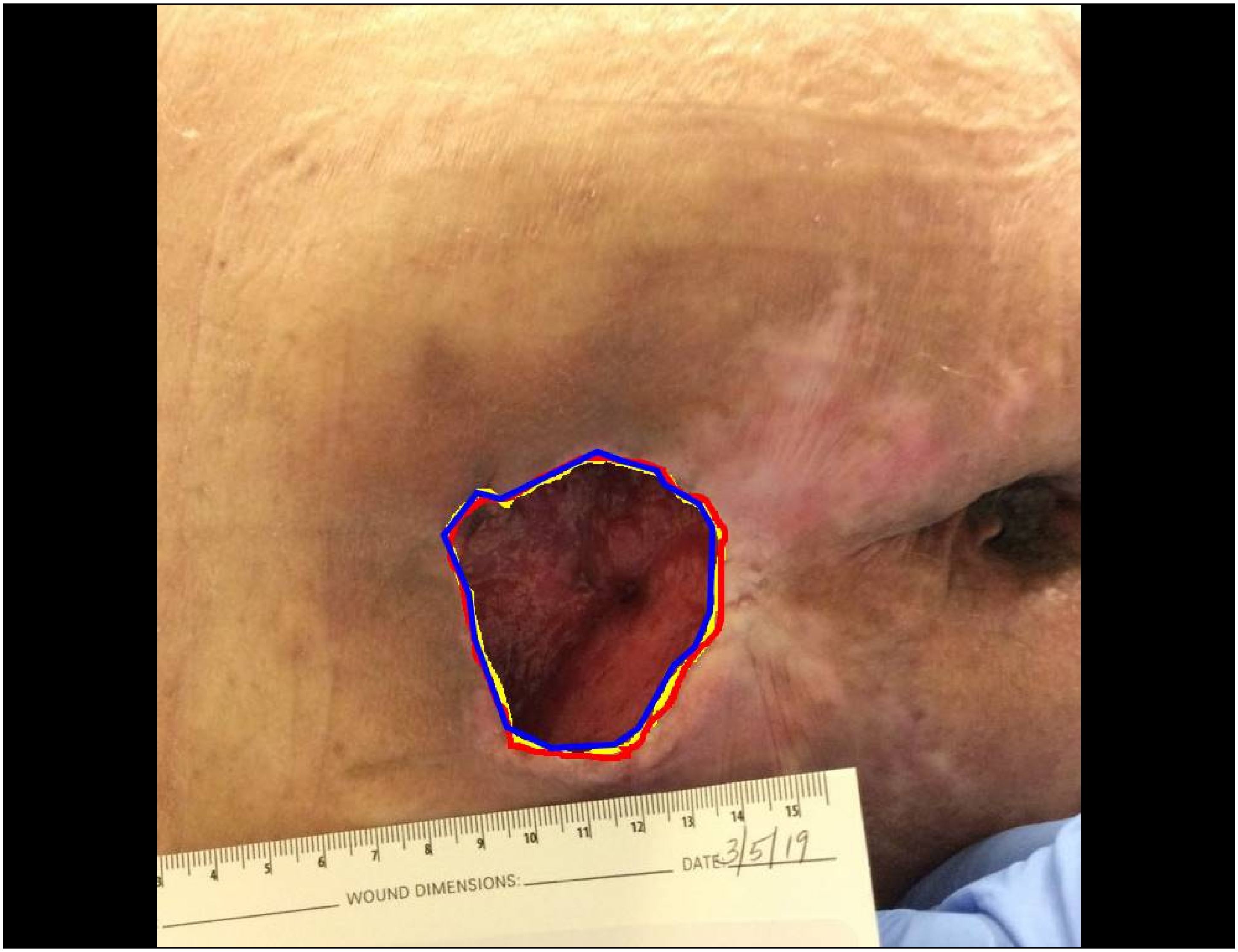

Tracing Data			
Tracer:	Wound Area (px^2)	Ruler Calibration (px/cm)	Wound Area (cm^2)
H1	38128	42.8	20.79
H2	39791	42.4	22.14
AI	36943	43.0	20.00

Tracing Comparisons				
Difference Metric:	Human-Human		Human-AI	
	H1(ref) H2(test)	H2(ref) H1(test)	H1(ref) AI(test)	H2(ref) AI(test)
False Negative Area (%)	0.7	4.9	4.6	7.8
False Positive Area (%)	5.1	0.7	1.5	0.7
|Relative Error (%)|	4.4	4.2	3.1	7.2

Blinded Attending Surgeon Review						
Reviewer	PGT Estimate	H1 meets definition?	H2 meets definition?	AI meets definition?	Which is AI?	Which is most accurate?
1	90	Yes	Yes	Yes	H2	H2
2	100	Yes	Yes	Yes	H1	H1
3	100	Yes	Yes	Yes	H2	H2

© 2021 Howell RS et al. *JAMA Network Open.*

Wound EMR Information								
Sequential Number	Gender	Age	Wound Type	Wound Location	Wound Length (cm)	Wound Width (cm)	Wound Depth (cm)	Wound Area (cm^2)
80	M	19	Pressure	Sacrum	6.0	2.5	1.5	15.00

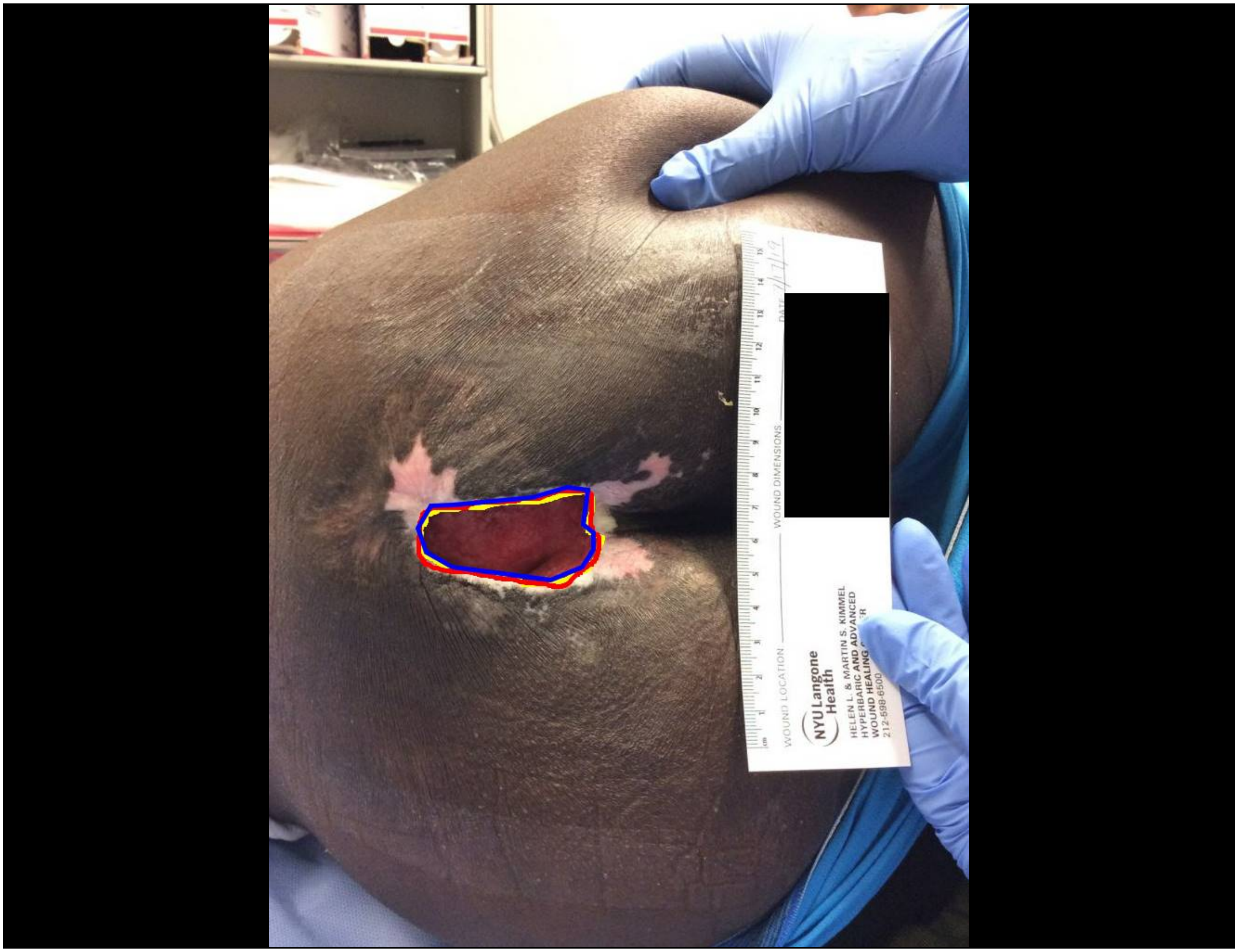

Tracing Data			
Tracer:	Wound Area (px^2)	Ruler Calibration (px/cm)	Wound Area (cm^2)
H1	14111	35.5	11.20
H2	15242	36.5	11.44
AI	13800	36.3	10.50

Tracing Comparisons				
Difference Metric:	Human-Human		Human-AI	
	H1(ref) H2(test)	H2(ref) H1(test)	H1(ref) AI(test)	H2(ref) AI(test)
False Negative Area (%)	0.8	8.2	8.4	11.3
False Positive Area (%)	8.8	0.7	6.2	1.8
|Relative Error (%)|	8.0	7.4	2.2	9.5

Blinded Attending Surgeon Review						
Reviewer	PGT Estimate	H1 meets definition?	H2 meets definition?	AI meets definition?	Which is AI?	Which is most accurate?
1	100	Yes	Yes	Yes	H1	H1
2	100	Yes	Yes	Yes	AI	H2
3	100	Yes	Yes	No	H1	H2

© 2021 Howell RS et al. *JAMA Network Open.*

Wound EMR Information								
Sequential Number	Gender	Age	Wound Type	Wound Location	Wound Length (cm)	Wound Width (cm)	Wound Depth (cm)	Wound Area (cm^2)
81	F	69	Surgical	Left lateral ankle				

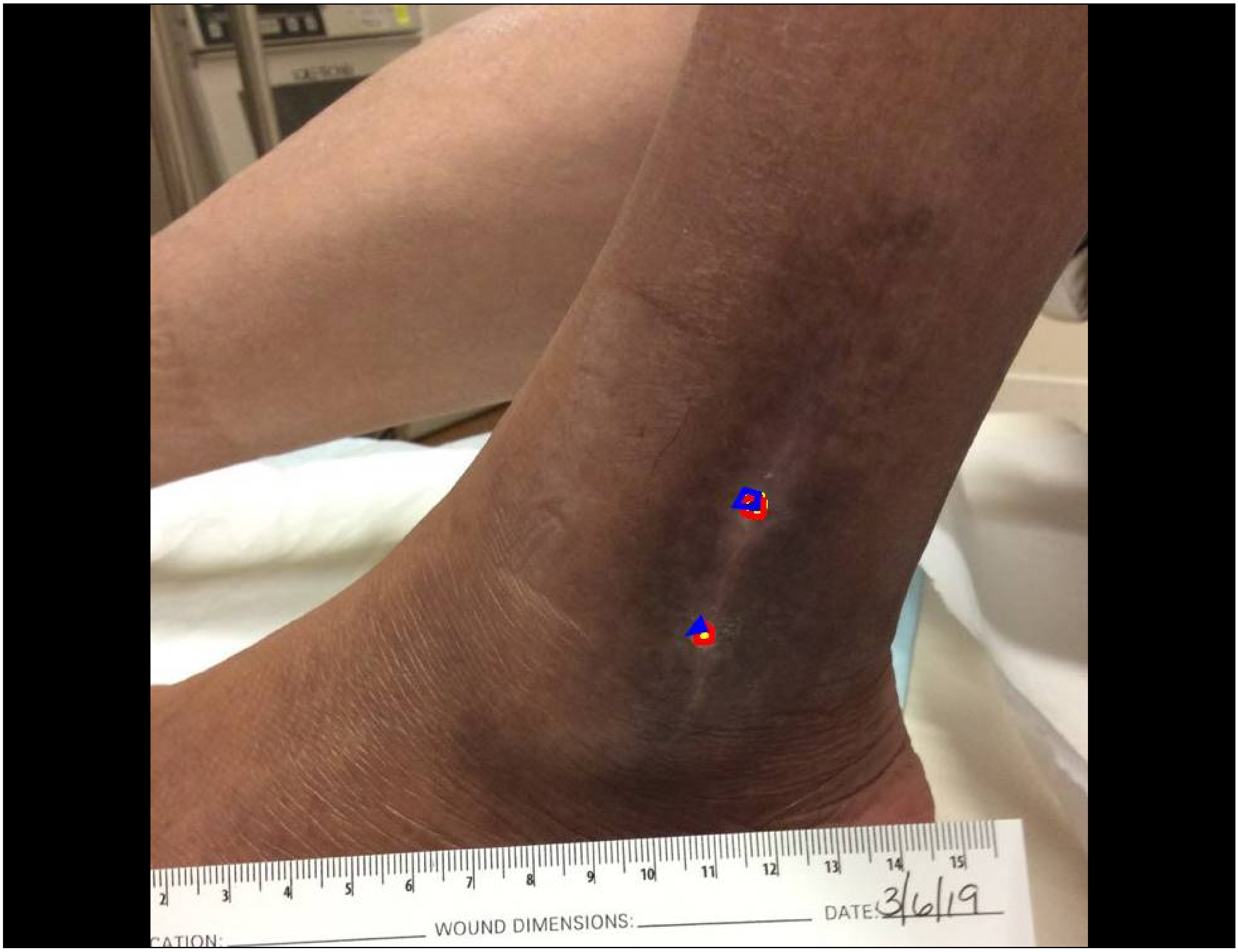

Tracing Data			
Tracer:	Wound Area (px^2)	Ruler Calibration (px/cm)	Wound Area (cm^2)
H1	276	48.4	0.12
H2	352	52.0	0.13
AI	194	44.0	0.10

Tracing Comparisons				
Difference Metric:	Human-Human		Human-AI	
	H1(ref) H2(test)	H2(ref) H1(test)	H1(ref) AI(test)	H2(ref) AI(test)
False Negative Area (%)	11.2	30.4	62.3	72.4
False Positive Area (%)	38.8	8.8	32.6	27.6
|Relative Error (%)|	27.5	21.6	29.7	44.9

Blinded Attending Surgeon Review						
Reviewer	PGT Estimate	H1 meets definition?	H2 meets definition?	AI meets definition?	Which is AI?	Which is most accurate?
1	0	Yes	Yes	Yes	H1	AI
2	100	Yes	Yes	Yes	H2	H2
3	100	Yes	Yes	No	H1	H2

© 2021 Howell RS et al. *JAMA Network Open.*

Wound EMR Information								
Sequential Number	Gender	Age	Wound Type	Wound Location	Wound Length (cm)	Wound Width (cm)	Wound Depth (cm)	Wound Area (cm^2)
82	M	35	Surgical	Abdomen	0.5	0.3		0.15

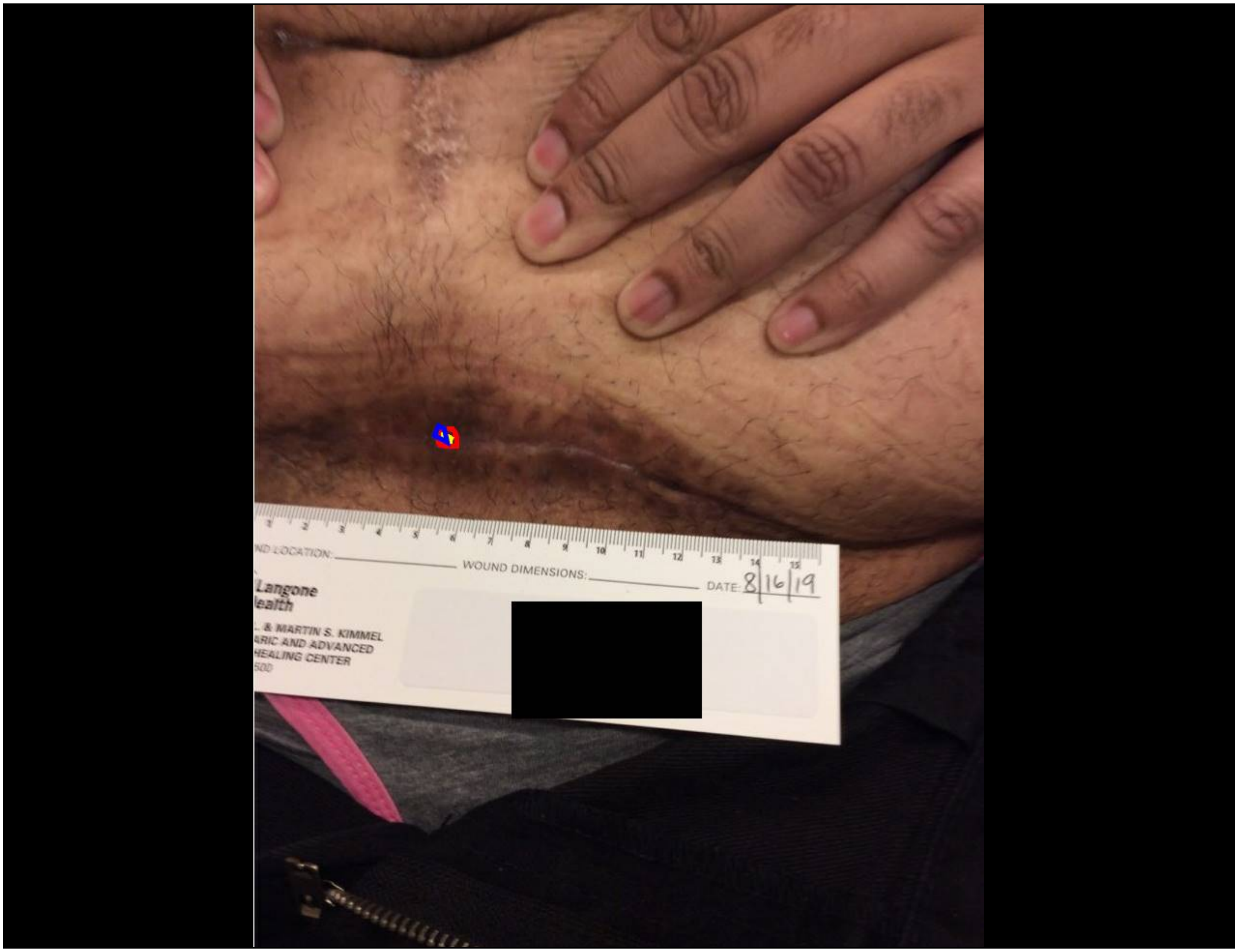

Tracing Data			
Tracer:	Wound Area (px^2)	Ruler Calibration (px/cm)	Wound Area (cm^2)
H1	70	38.2	0.05
H2	302	39.3	0.20
AI	122	34.9	0.10

Tracing Comparisons				
Difference Metric:	Human-Human		Human-AI	
	H1(ref) H2(test)	H2(ref) H1(test)	H1(ref) AI(test)	H2(ref) AI(test)
False Negative Area (%)	0.0	76.8	40.0	71.9
False Positive Area (%)	331.4	0.0	114.3	12.3
|Relative Error (%)|	331.4	76.8	74.3	59.6

Blinded Attending Surgeon Review						
Reviewer	PGT Estimate	H1 meets definition?	H2 meets definition?	AI meets definition?	Which is AI?	Which is most accurate?
1	100	Yes	Yes	Yes	H1	H2
2	100	Yes	Yes	Yes	H2	AI
3	100	Yes	No	No	AI	H2

© 2021 Howell RS et al. *JAMA Network Open.*

Wound EMR Information								
Sequential Number	Gender	Age	Wound Type	Wound Location	Wound Length (cm)	Wound Width (cm)	Wound Depth (cm)	Wound Area (cm²)
83	M	56	Pressure	Right ischium	3.0		8.0	

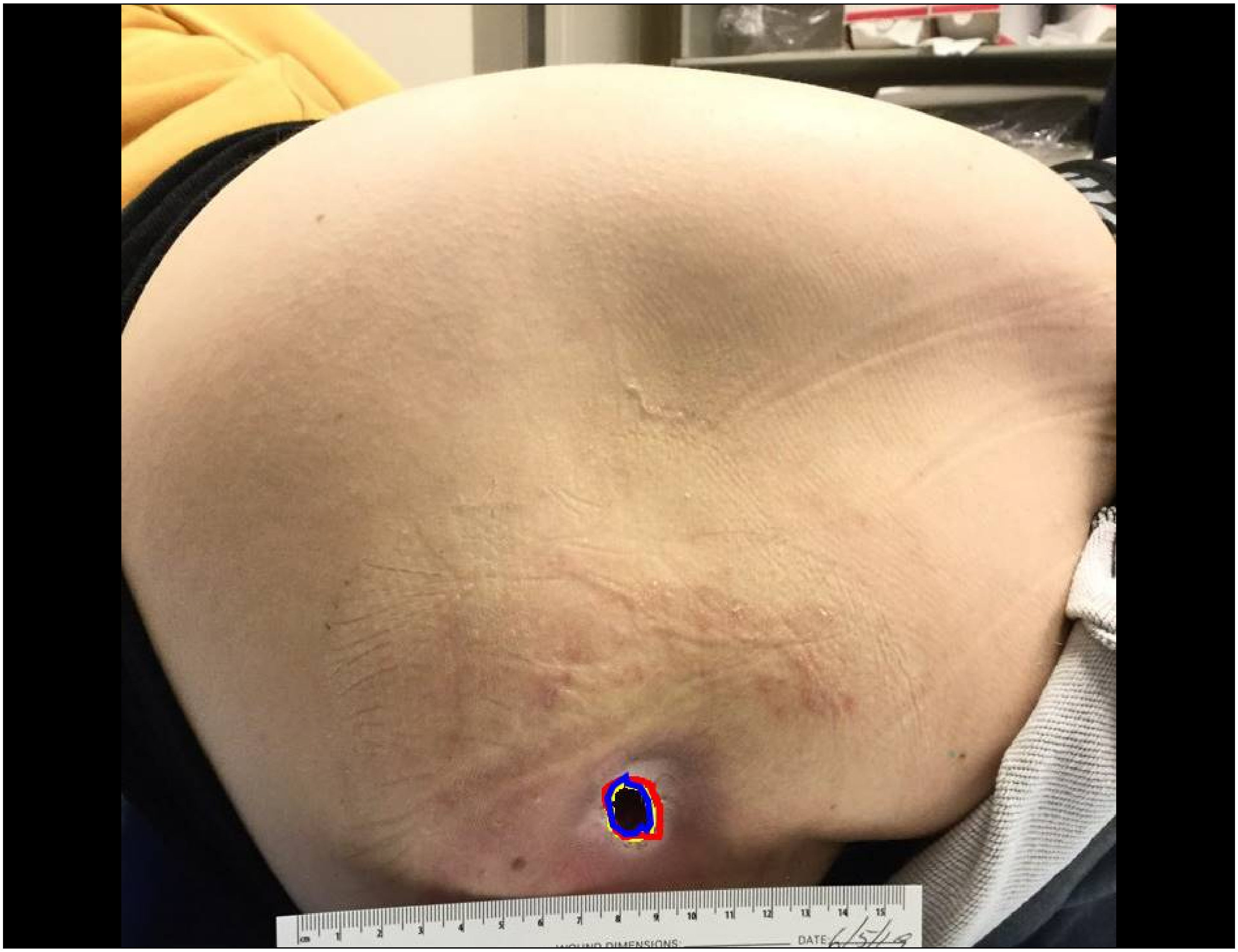

Tracing Data			
Tracer:	Wound Area (px²)	Ruler Calibration (px/cm)	Wound Area (cm²)
H1	1128	29.6	1.29
H2	1511	29.0	1.80
AI	1036	29.4	1.20

Tracing Comparisons				
Difference Metric:	Human-Human		Human-AI	
	H1(ref) H2(test)	H2(ref) H1(test)	H1(ref) AI(test)	H2(ref) AI(test)
False Negative Area (%)	2.7	27.4	12.1	31.7
False Positive Area (%)	36.7	2.1	3.9	0.3
|Relative Error (%)|	34.0	25.3	8.2	31.4

Blinded Attending Surgeon Review						
Reviewer	PGT Estimate	H1 meets definition?	H2 meets definition?	AI meets definition?	Which is AI?	Which is most accurate?
1	0	Yes	Yes	Yes	H1	H2
2	50	Yes	Yes	Yes	H2	AI
3	100	Yes	No	Yes	H2	AI

© 2021 Howell RS et al. *JAMA Network Open.*

Wound EMR Information								
Sequential Number	Gender	Age	Wound Type	Wound Location	Wound Length (cm)	Wound Width (cm)	Wound Depth (cm)	Wound Area (cm^2)
84	F	50	Surgical	Lower abdomen	4.0	6.0		24.00

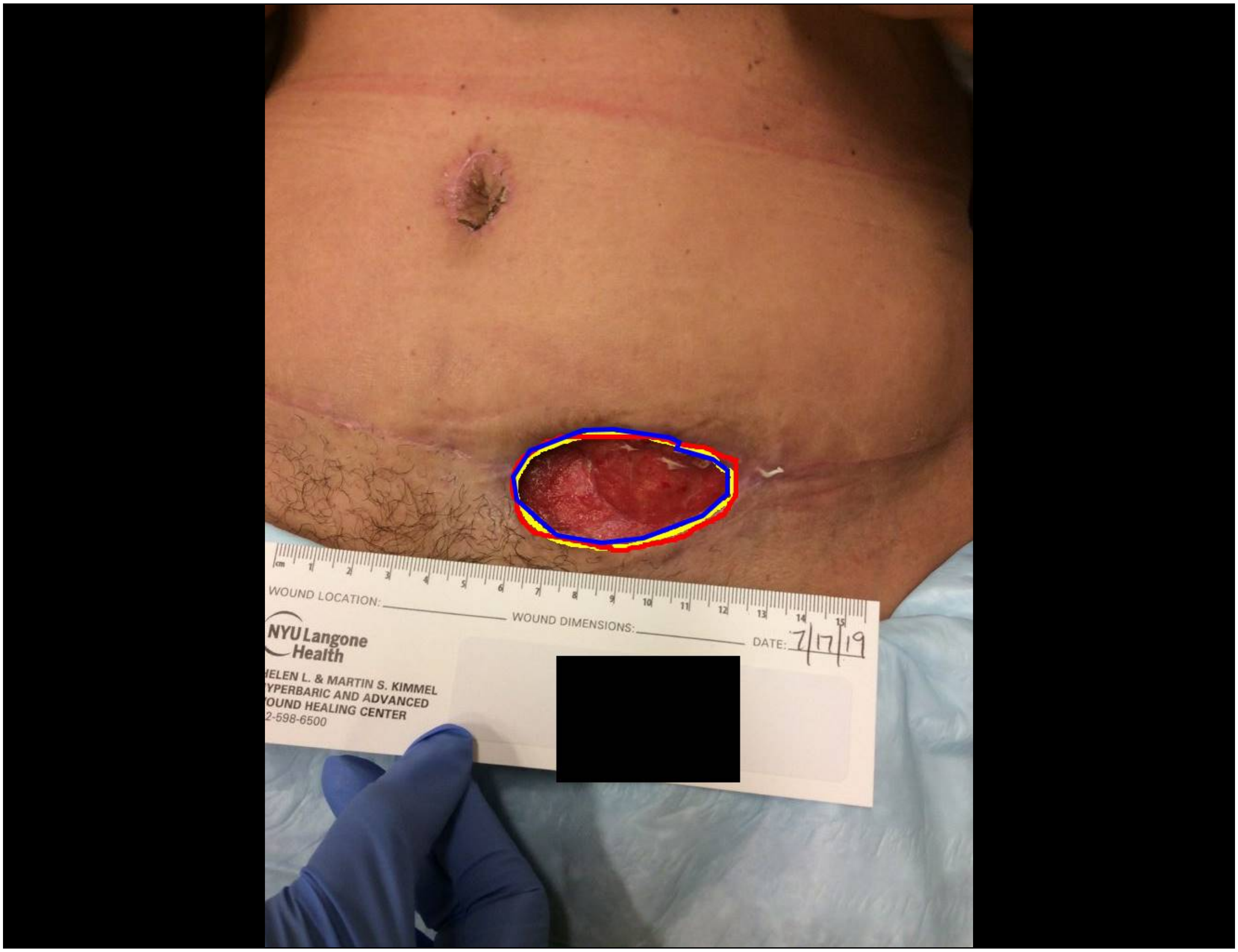

Tracing Data			
Tracer:	Wound Area (px^2)	Ruler Calibration (px/cm)	Wound Area (cm^2)
H1	21793	40.3	13.40
H2	23594	40.6	14.34
AI	20863	41.2	12.30

Tracing Comparisons				
Difference Metric:	Human-Human		Human-AI	
	H1(ref) H2(test)	H2(ref) H1(test)	H1(ref) AI(test)	H2(ref) AI(test)
False Negative Area (%)	1.7	9.2	7.2	13.8
False Positive Area (%)	9.9	1.5	3.0	2.3
|Relative Error (%)|	8.3	7.6	4.3	11.6

Blinded Attending Surgeon Review						
Reviewer	PGT Estimate	H1 meets definition?	H2 meets definition?	AI meets definition?	Which is AI?	Which is most accurate?
1	100	Yes	Yes	Yes	H2	H2
2	100	Yes	Yes	Yes	H2	H1
3	100	Yes	Yes	Yes	H1	H2

© 2021 Howell RS et al. *JAMA Network Open.*

Wound EMR Information								
**Sequential Number**	**Gender**	**Age**	**Wound Type**	**Wound Location**	**Wound Length (cm)**	**Wound Width (cm)**	**Wound Depth (cm)**	**Wound Area (cm²)**
86	F	36	Surgical	Left lower back				

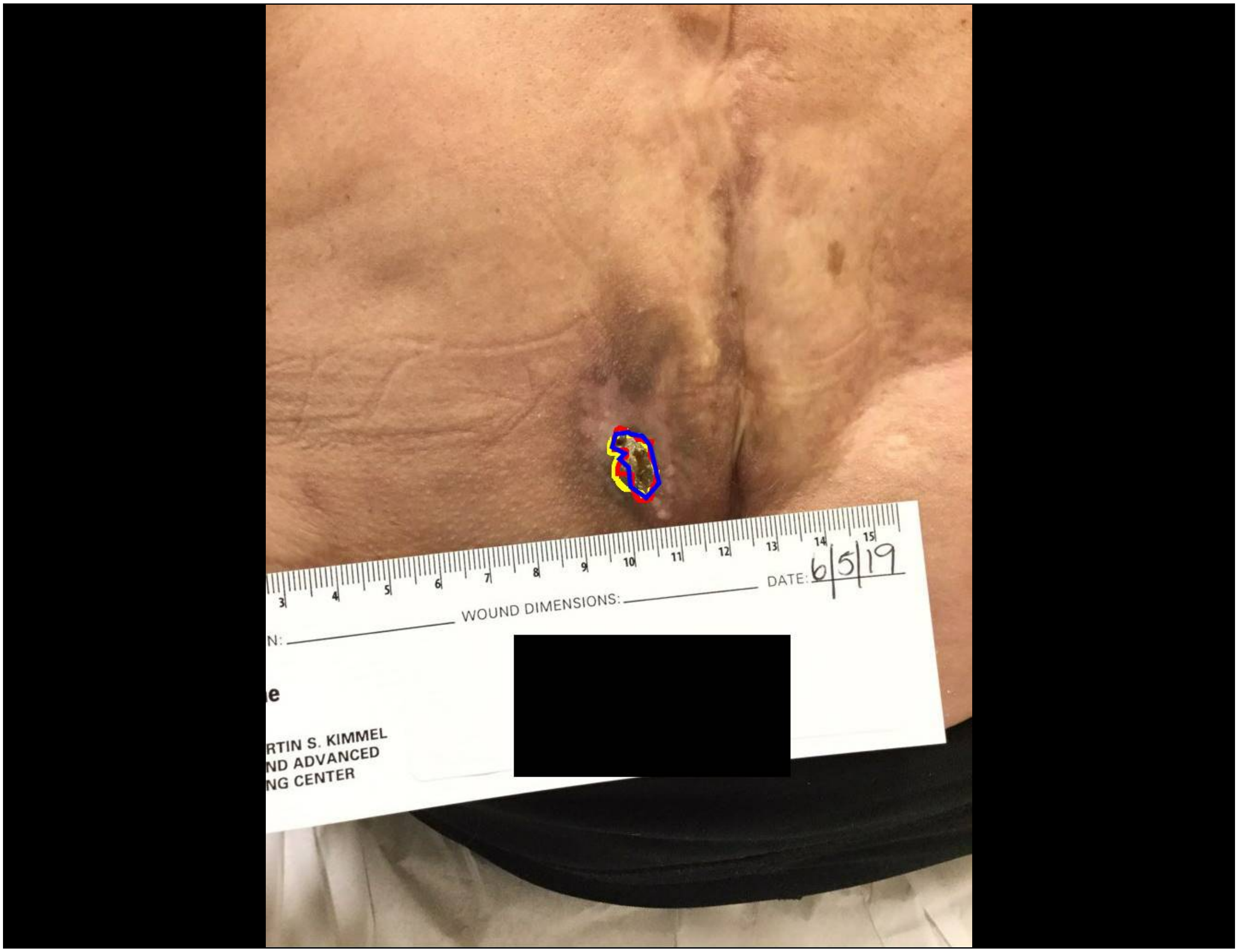

Tracing Data			
**Tracer:**	**Wound Area (px²)**	**Ruler Calibration (px/cm)**	**Wound Area (cm²)**
**H1**	2445	52.2	0.90
**H2**	2134	35.0	1.74
**AI**	2061	54.3	0.70

Tracing Comparisons				
**Difference Metric:**	**Human-Human**		**Human-AI**	
	**H1(ref) H2(test)**	**H2(ref) H1(test)**	**H1(ref) AI(test)**	**H2(ref) AI(test)**
**False Negative Area (%)**	20.9	9.3	22.8	14.9
**False Positive Area (%)**	8.1	23.9	7.1	11.4
**|Relative Error (%)|**	12.7	14.6	15.7	3.4

Blinded Attending Surgeon Review						
**Reviewer**	**PGT Estimate**	**H1 meets definition?**	**H2 meets definition?**	**AI meets definition?**	**Which is AI?**	**Which is most accurate?**
**1**	0	Yes	Yes	Yes	H1	H1
**2**	0					
**3**	0	No	Yes	Yes	H2	AI

© 2021 Howell RS et al. *JAMA Network Open.*

Wound EMR Information								
Sequential Number	Gender	Age	Wound Type	Wound Location	Wound Length (cm)	Wound Width (cm)	Wound Depth (cm)	Wound Area (cm^2)
87	F	93	Arterial	Left knee				

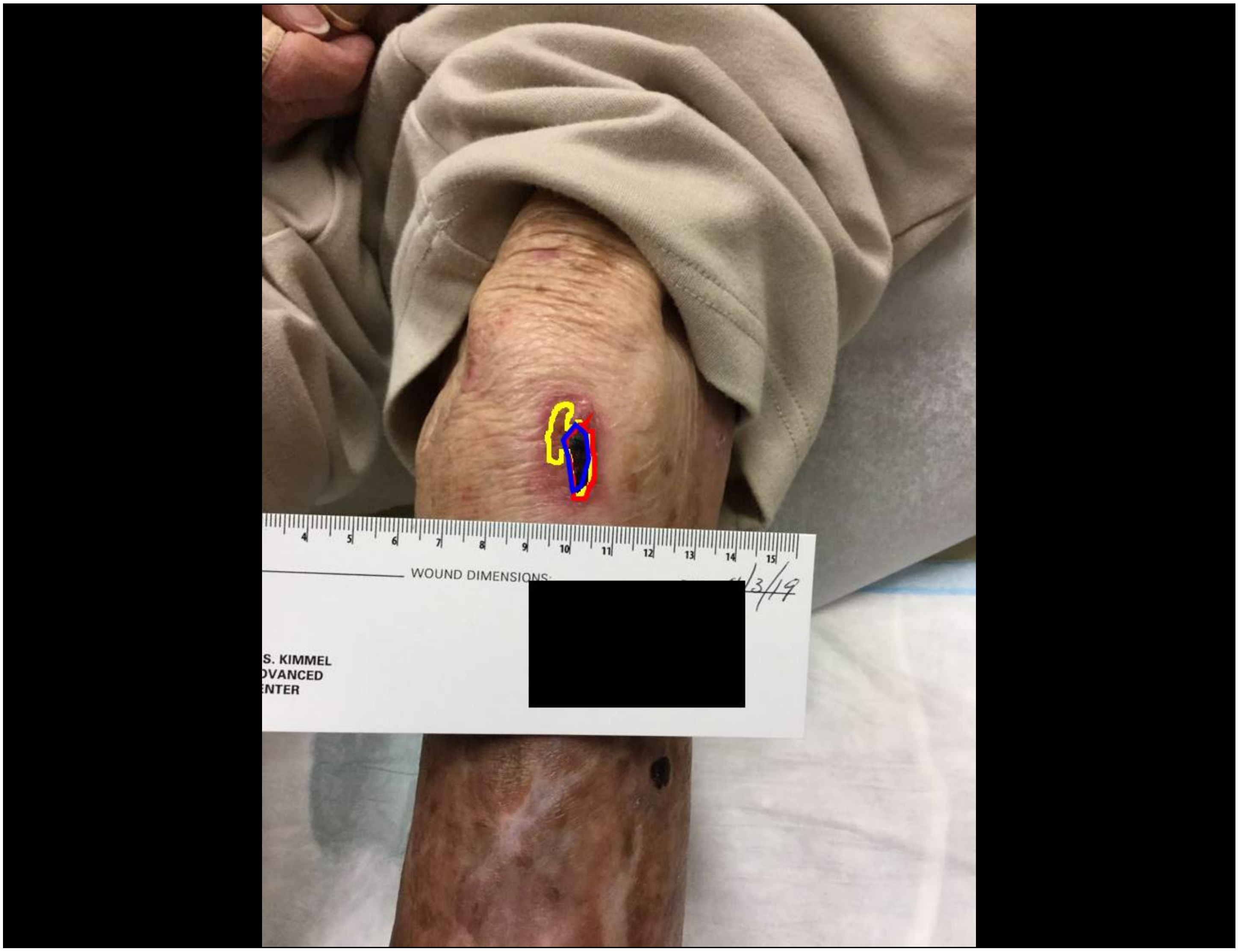

Tracing Data			
Tracer:	Wound Area (px^2)	Ruler Calibration (px/cm)	Wound Area (cm^2)
H1	2483	44.5	1.25
H2	1705	46.1	0.80
AI	1150	43.8	0.60

Tracing Comparisons				
Difference Metric:	Human-Human		Human-AI	
	H1(ref) H2(test)	H2(ref) H1(test)	H1(ref) AI(test)	H2(ref) AI(test)
False Negative Area (%)	43.2	17.3	55.7	37.5
False Positive Area (%)	11.9	62.9	2.1	4.9
|Relative Error (%)|	31.3	45.6	53.7	32.6

Blinded Attending Surgeon Review						
Reviewer	PGT Estimate	H1 meets definition?	H2 meets definition?	AI meets definition?	Which is AI?	Which is most accurate?
1	0	Yes	No	No	H2	H1
2	30	Yes	No	No	H2	H1
3	0	No	Yes	No	H2	H1

© 2021 Howell RS et al. *JAMA Network Open.*

Wound EMR Information								
Sequential Number	Gender	Age	Wound Type	Wound Location	Wound Length (cm)	Wound Width (cm)	Wound Depth (cm)	Wound Area (cm^2)
88	F	50	Burn	Left medial lower	4.0	6.0		24.00

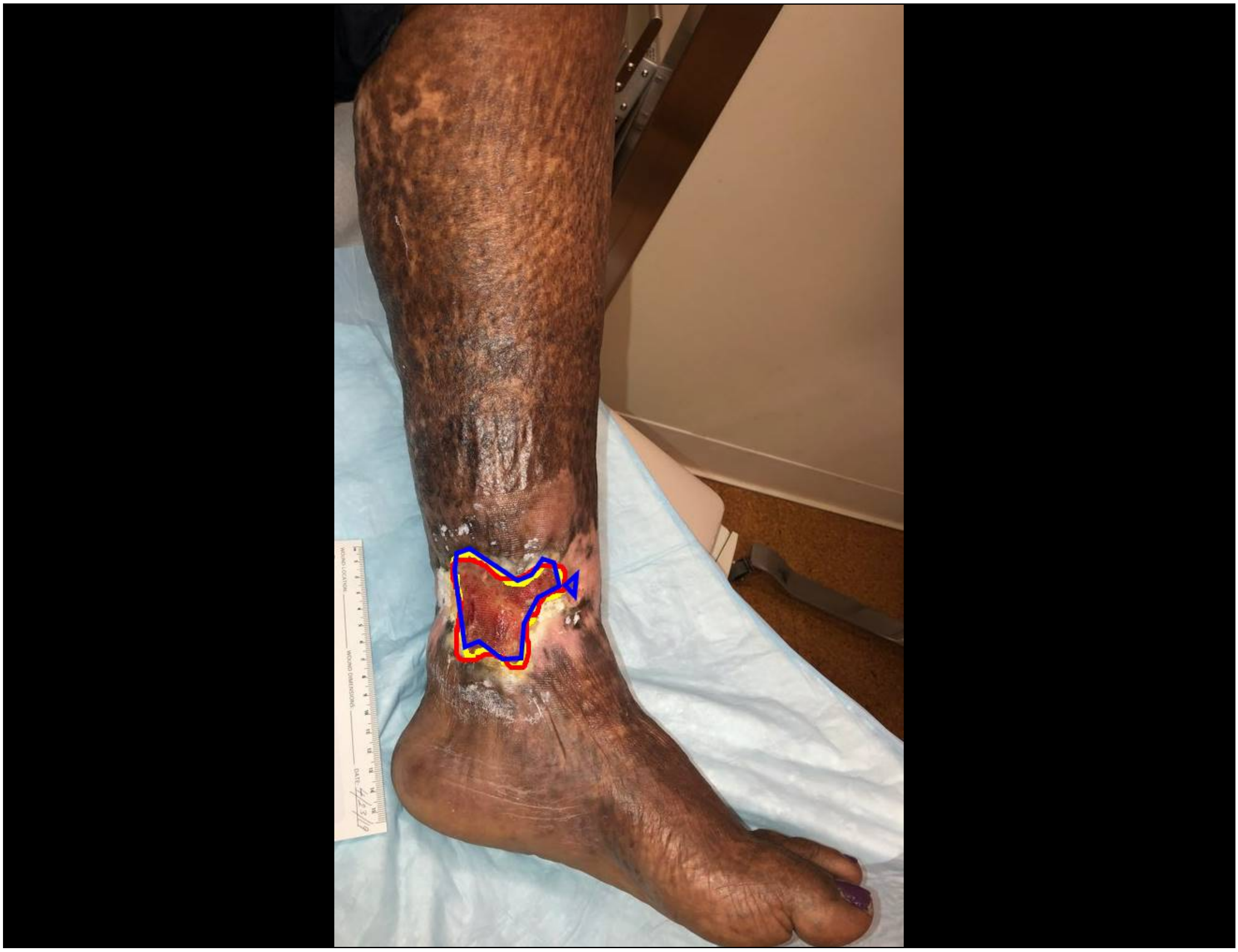

Tracing Data			
Tracer:	Wound Area (px^2)	Ruler Calibration (px/cm)	Wound Area (cm^2)
H1	8095	14.9	36.67
H2	8650	15.6	35.53
AI	7804	20.1	19.30

Tracing Comparisons				
Difference Metric:	Human-Human		Human-AI	
	H1(ref) H2(test)	H2(ref) H1(test)	H1(ref) AI(test)	H2(ref) AI(test)
False Negative Area (%)	5.1	11.2	11.3	17.9
False Positive Area (%)	12.0	4.8	7.7	8.1
|Relative Error (%)|	6.9	6.4	3.6	9.8

Blinded Attending Surgeon Review						
Reviewer	PGT Estimate	H1 meets definition?	H2 meets definition?	AI meets definition?	Which is AI?	Which is most accurate?
1	90	Yes	Yes	Yes	H1	H1
2	100	Yes	Yes	Yes	AI	H1
3	100	Yes	No	No	H1	H2

© 2021 Howell RS et al. *JAMA Network Open.*

Wound EMR Information								
Sequential Number	Gender	Age	Wound Type	Wound Location	Wound Length (cm)	Wound Width (cm)	Wound Depth (cm)	Wound Area (cm^2)
89	M	77	Radiation	Lower back				

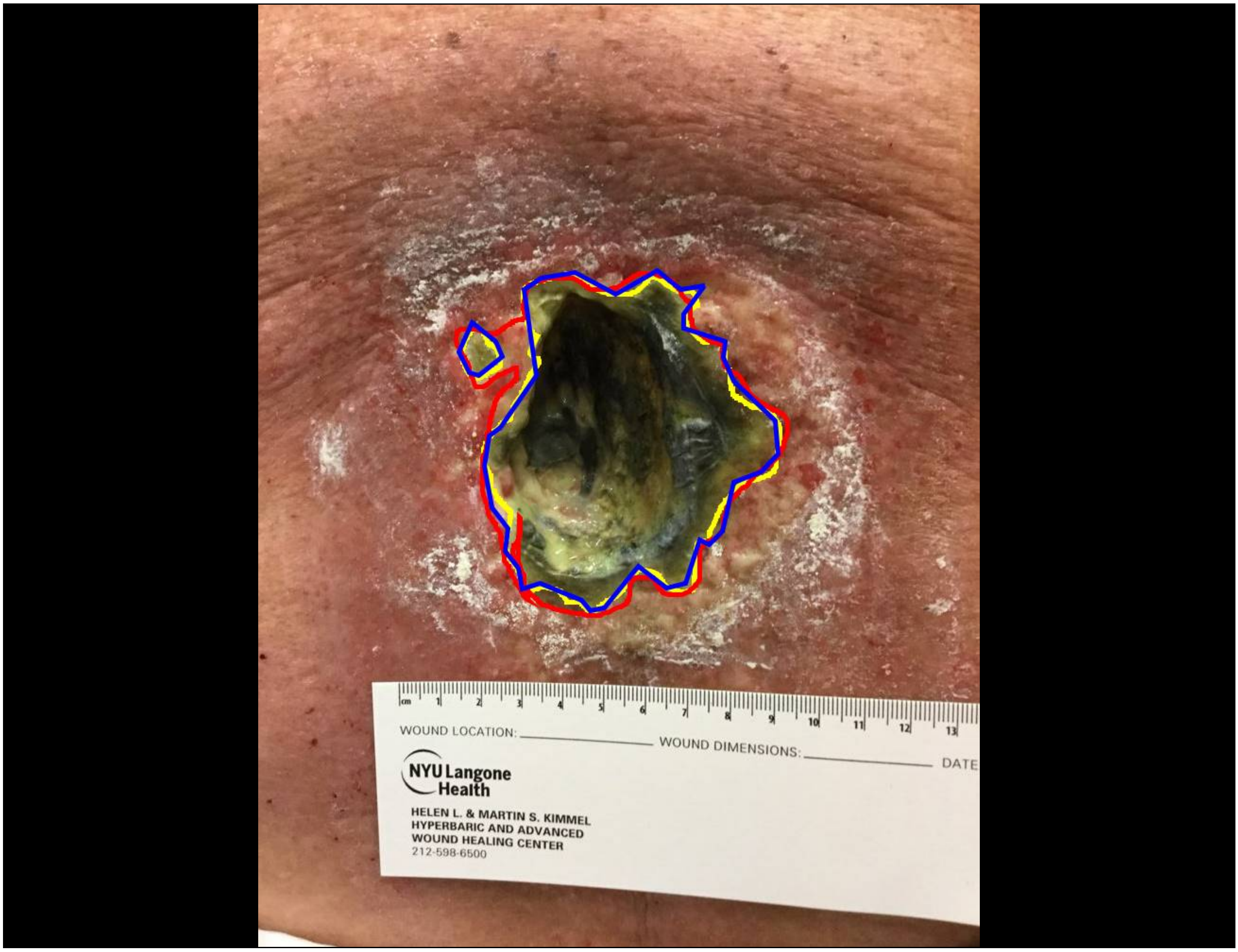

Tracing Data			
Tracer:	Wound Area (px^2)	Ruler Calibration (px/cm)	Wound Area (cm^2)
H1	74898	43.5	39.52
H2	83376	43.7	43.64
AI	75739	42.4	42.20

Tracing Comparisons				
Difference Metric:	Human-Human		Human-AI	
	H1(ref) H2(test)	H2(ref) H1(test)	H1(ref) AI(test)	H2(ref) AI(test)
False Negative Area (%)	1.2	11.2	2.3	10.4
False Positive Area (%)	12.5	1.1	3.4	1.2
|Relative Error (%)|	11.3	10.2	1.1	9.2

Blinded Attending Surgeon Review						
Reviewer	PGT Estimate	H1 meets definition?	H2 meets definition?	AI meets definition?	Which is AI?	Which is most accurate?
1	0	Yes	Yes	Yes	H1	AI
2	0	Yes	Yes	Yes	H1	AI
3	10	Yes	Yes	Yes	AI	H1

© 2021 Howell RS et al. *JAMA Network Open.*

Wound EMR Information								
Sequential Number	Gender	Age	Wound Type	Wound Location	Wound Length (cm)	Wound Width (cm)	Wound Depth (cm)	Wound Area (cm^2)
90	F	26	Pressure	Right ischium	3.0	5.0		15.00

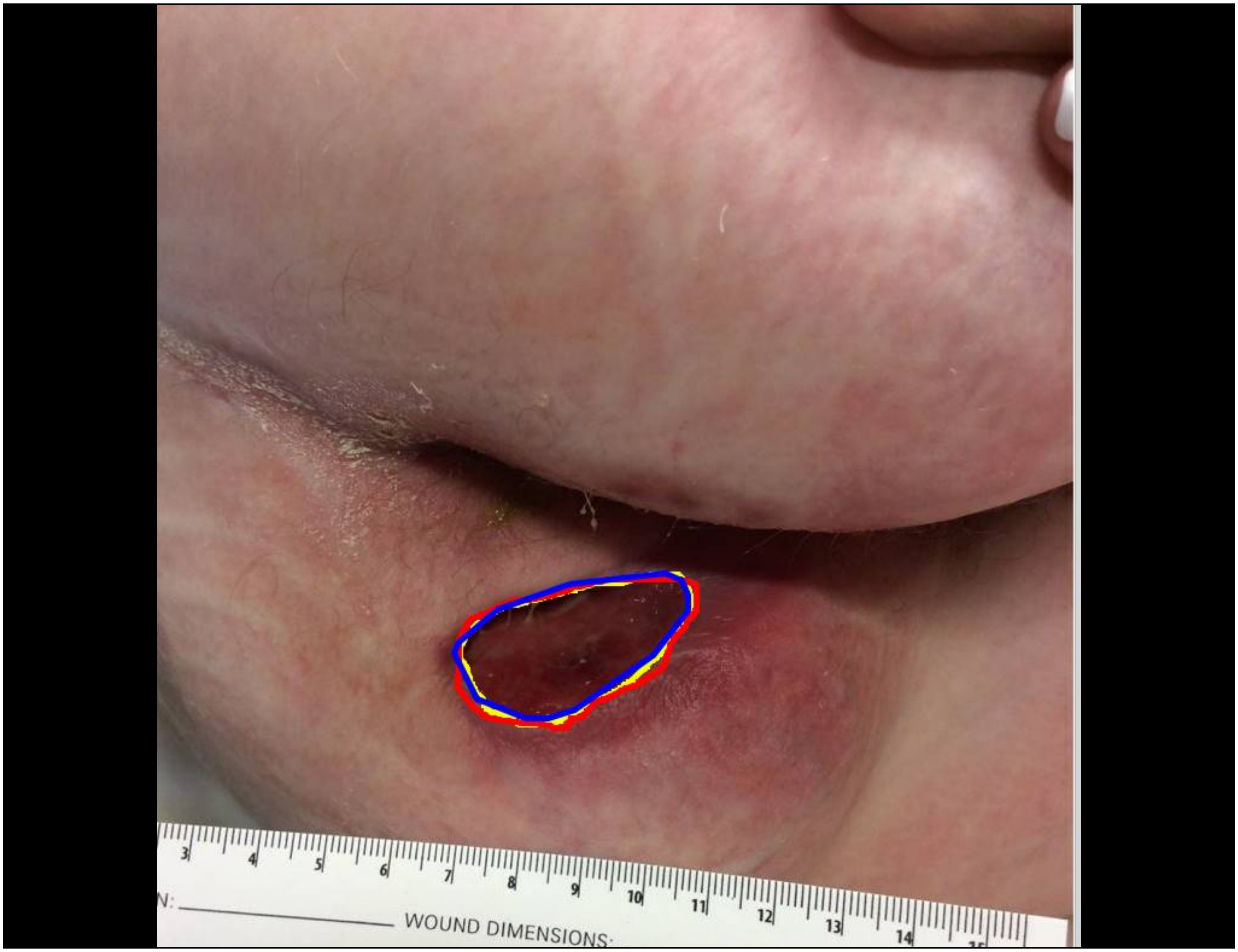

Tracing Data			
Tracer:	Wound Area (px^2)	Ruler Calibration (px/cm)	Wound Area (cm^2)
H1	15591	52.8	5.60
H2	17098	53.3	6.02
AI	14993	52.7	5.40

Tracing Comparisons				
Difference Metric:	Human-Human		Human-AI	
	H1(ref) H2(test)	H2(ref) H1(test)	H1(ref) AI(test)	H2(ref) AI(test)
False Negative Area (%)	0.9	9.7	6.2	13.8
False Positive Area (%)	10.6	0.8	2.4	1.5
|Relative Error (%)|	9.7	8.8	3.8	12.3

Blinded Attending Surgeon Review						
Reviewer	PGT Estimate	H1 meets definition?	H2 meets definition?	AI meets definition?	Which is AI?	Which is most accurate?
1	100	Yes	Yes	Yes	H2	AI
2	100	Yes	Yes	Yes	H1	H1
3	100	Yes	Yes	Yes	H1	AI

© 2021 Howell RS et al. *JAMA Network Open.*

Wound EMR Information								
**Sequential Number**	**Gender**	**Age**	**Wound Type**	**Wound Location**	**Wound Length (cm)**	**Wound Width (cm)**	**Wound Depth (cm)**	**Wound Area (cm^2)**
93	F	72	Pressure	Left lateral leg	21.0	6.5	31.0	136.50

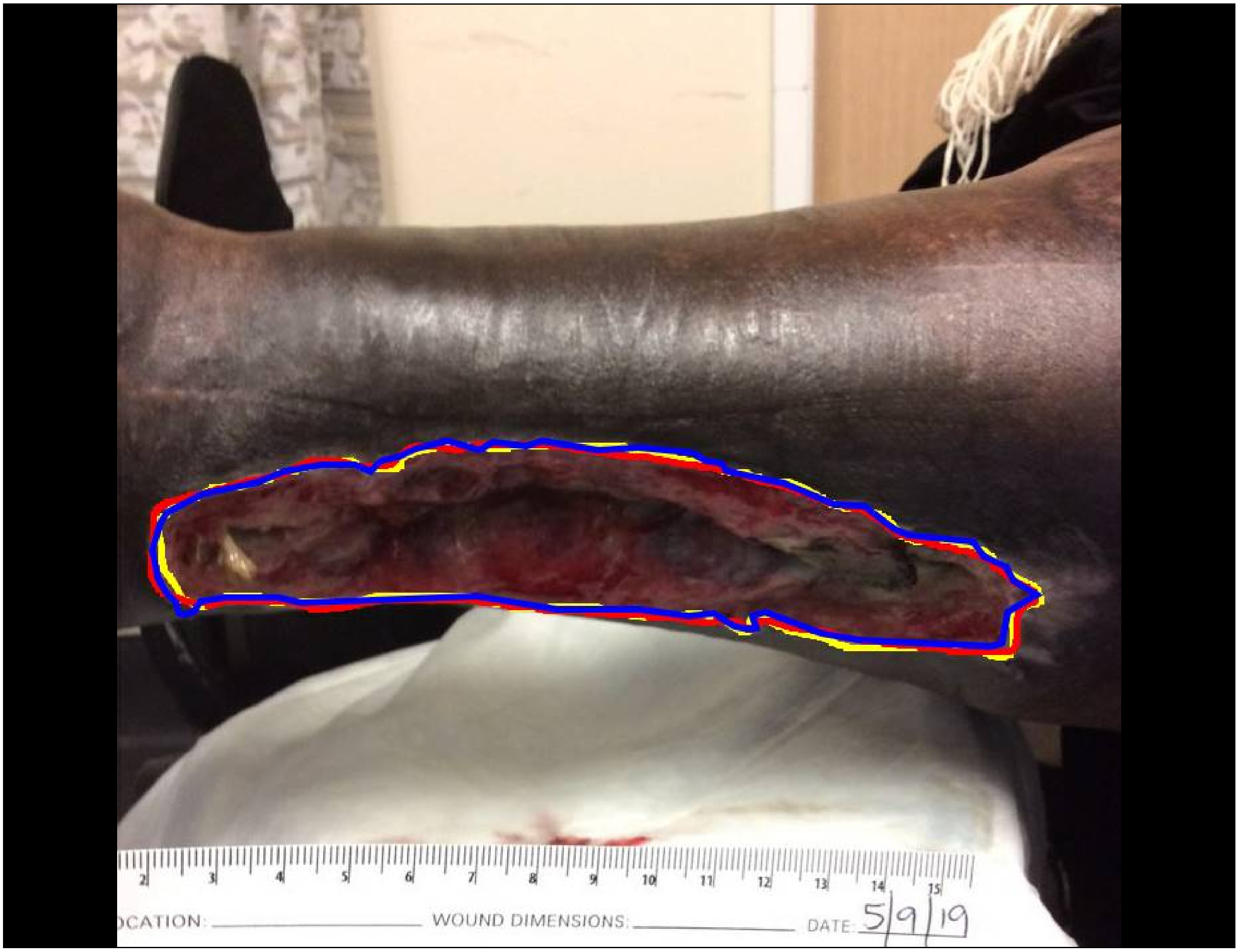

Tracing Data			
**Tracer:**	**Wound Area (px^2)**	**Ruler Calibration (px/cm)**	**Wound Area (cm^2)**
**H1**	65917	45.8	31.38
**H2**	66390	41.5	38.61
**AI**	65366	47.6	28.90

Tracing Comparisons				
**Difference Metric:**	**Human-Human**		**Human-AI**	
	**H1(ref) H2(test)**	**H2(ref) H1(test)**	**H1(ref) AI(test)**	**H2(ref) AI(test)**
**False Negative Area (%)**	2.6	3.3	3.3	3.8
**False Positive Area (%)**	3.3	2.6	2.4	2.3
**|Relative Error (%)|**	0.7	0.7	0.8	1.5

Blinded Attending Surgeon Review						
**Reviewer**	**PGT Estimate**	**H1 meets definition?**	**H2 meets definition?**	**AI meets definition?**	**Which is AI?**	**Which is most accurate?**
**1**	80	Yes	Yes	Yes	H2	H1
**2**	100	Yes	Yes	Yes	H2	AI
**3**	50	Yes	Yes	Yes	H2	H1

© 2021 Howell RS et al. *JAMA Network Open.*

Wound EMR Information								
Sequential Number	Gender	Age	Wound Type	Wound Location	Wound Length (cm)	Wound Width (cm)	Wound Depth (cm)	Wound Area (cm^2)
94	F	72	IV	Right forearm				

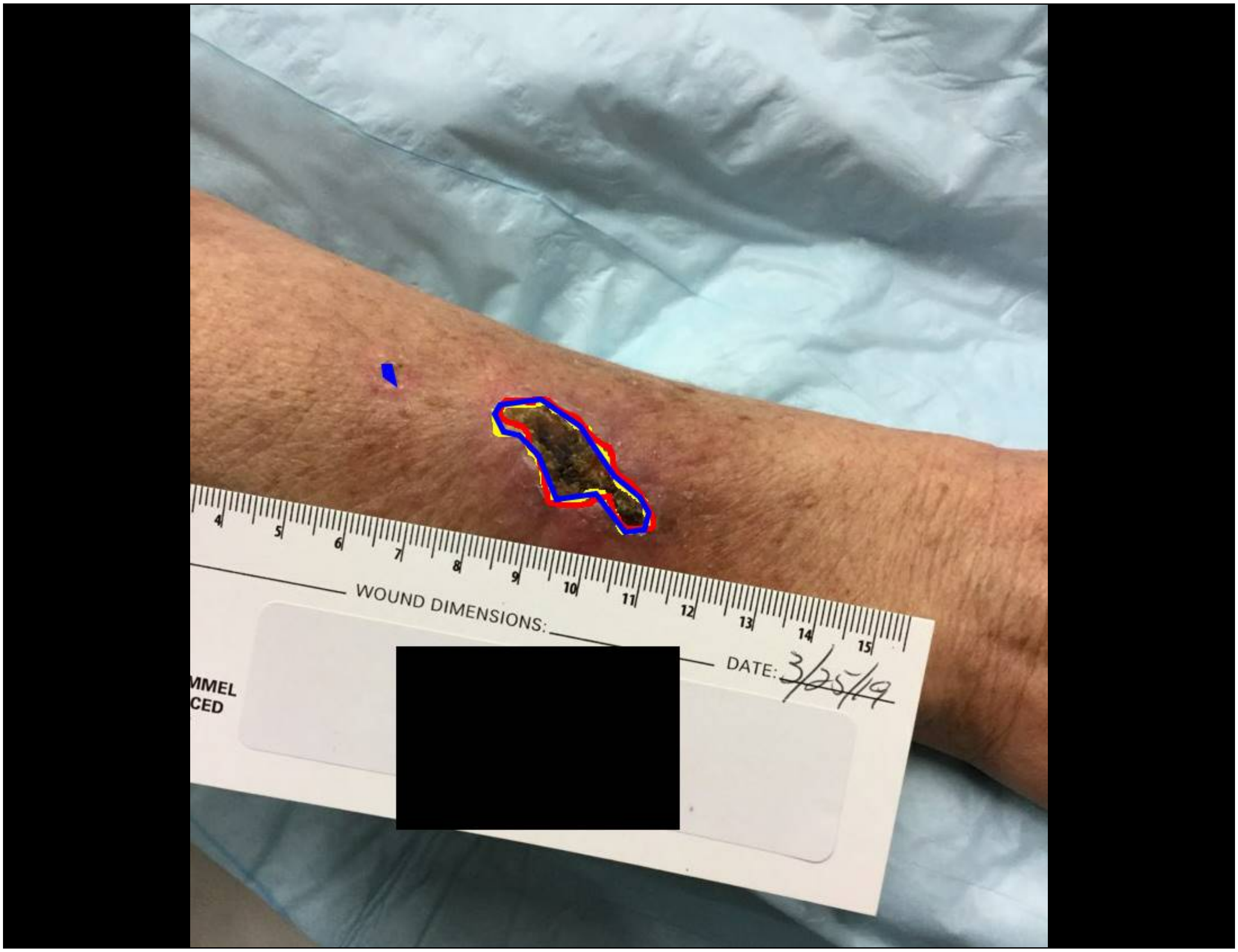

Tracing Data			
Tracer:	Wound Area (px^2)	Ruler Calibration (px/cm)	Wound Area (cm^2)
H1	6197	53.0	2.20
H2	6642	53.9	2.29
AI	6065	51.4	2.30

Tracing Comparisons				
Difference Metric:	Human-Human		Human-AI	
	H1(ref) H2(test)	H2(ref) H1(test)	H1(ref) AI(test)	H2(ref) AI(test)
False Negative Area (%)	7.5	13.7	7.9	16.1
False Positive Area (%)	14.7	7.0	5.7	7.4
|Relative Error (%)|	7.2	6.7	2.1	8.7

Blinded Attending Surgeon Review						
Reviewer	PGT Estimate	H1 meets definition?	H2 meets definition?	AI meets definition?	Which is AI?	Which is most accurate?
1	10	No	No	Yes	H2	H1
2	0	Yes	Yes	Yes	H1	AI
3	0	Yes	Yes	No	H2	H1

© 2021 Howell RS et al. *JAMA Network Open.*

Wound EMR Information								
Sequential Number	Gender	Age	Wound Type	Wound Location	Wound Length (cm)	Wound Width (cm)	Wound Depth (cm)	Wound Area (cm^2)
95	M	63	Pressure	Left medial foot				

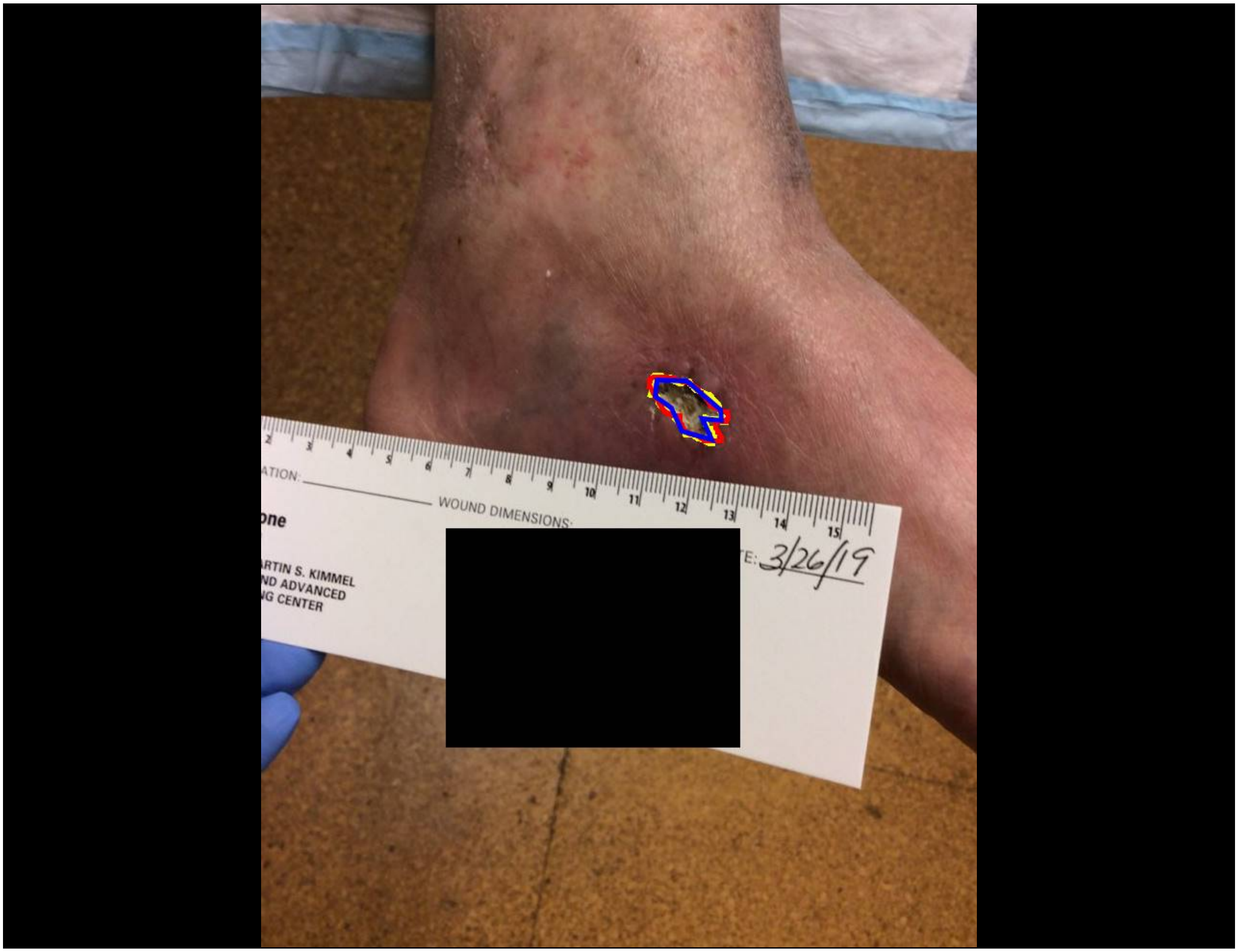

Tracing Data			
Tracer:	Wound Area (px^2)	Ruler Calibration (px/cm)	Wound Area (cm^2)
H1	2708	51.3	1.03
H2	2836	50.5	1.11
AI	2300	48.0	1.00

Tracing Comparisons				
Difference Metric:	Human-Human		Human-AI	
	H1(ref) H2(test)	H2(ref) H1(test)	H1(ref) AI(test)	H2(ref) AI(test)
False Negative Area (%)	8.6	12.7	17.4	22.2
False Positive Area (%)	13.3	8.2	2.4	3.3
|Relative Error (%)|	4.7	4.5	15.1	18.9

Blinded Attending Surgeon Review						
Reviewer	PGT Estimate	H1 meets definition?	H2 meets definition?	AI meets definition?	Which is AI?	Which is most accurate?
1	10	Yes	Yes	Yes	H2	H2
2	20	Yes	Yes	Yes	H1	H2
3	0	Yes	Yes	Yes	H2	H2

© 2021 Howell RS et al. *JAMA Network Open.*

Wound EMR Information								
Sequential Number	Gender	Age	Wound Type	Wound Location	Wound Length (cm)	Wound Width (cm)	Wound Depth (cm)	Wound Area (cm^2)
96	F	72	Pressure	Left ischium				

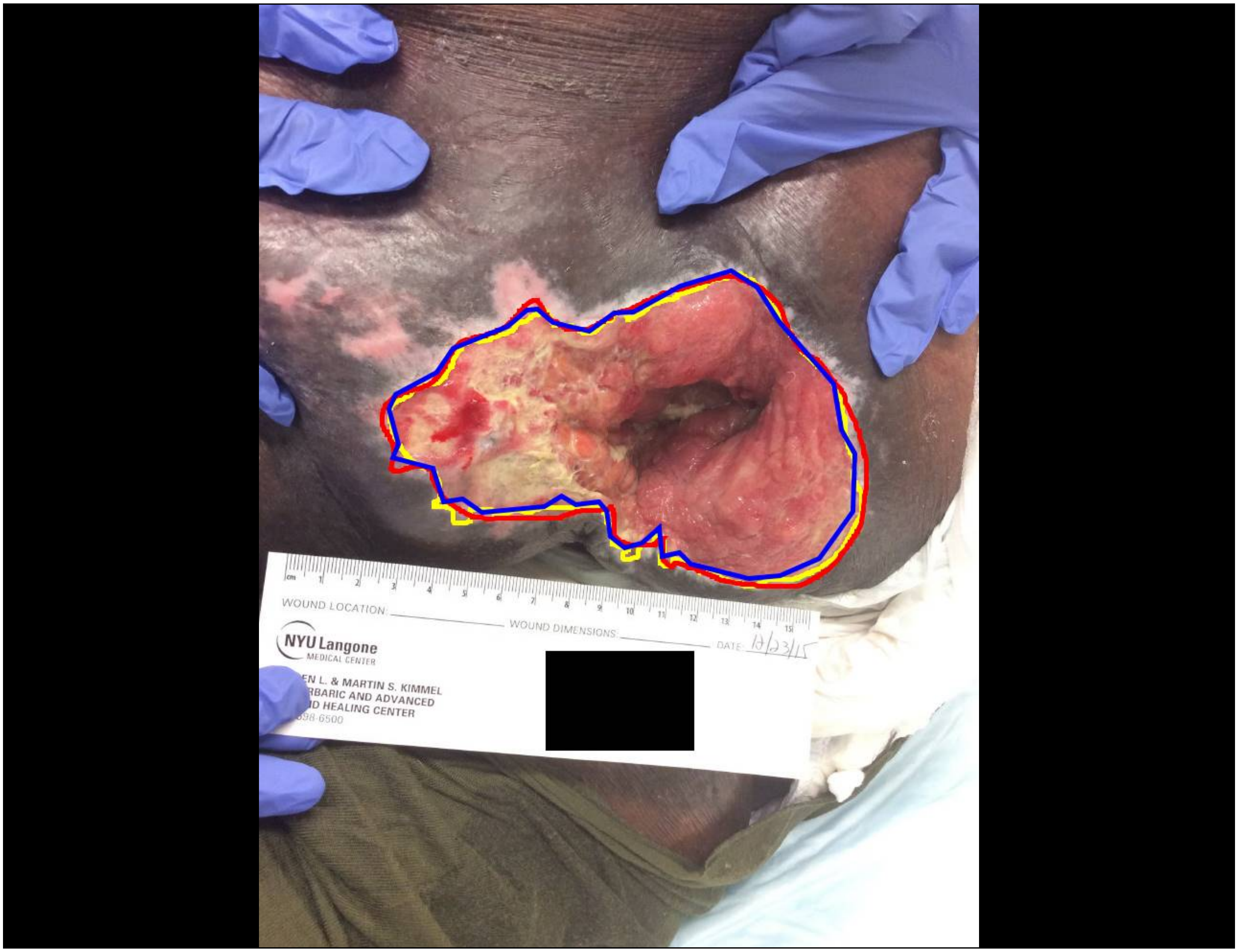

Tracing Data			
Tracer:	Wound Area (px^2)	Ruler Calibration (px/cm)	Wound Area (cm^2)
H1	102584	35.8	80.09
H2	107509	36.1	82.49
AI	100653	35.6	79.20

Tracing Comparisons				
Difference Metric:	Human-Human		Human-AI	
	H1(ref) H2(test)	H2(ref) H1(test)	H1(ref) AI(test)	H2(ref) AI(test)
False Negative Area (%)	1.1	5.6	4.0	7.2
False Positive Area (%)	5.9	1.0	2.1	0.8
|Relative Error (%)|	4.8	4.6	1.9	6.4

Blinded Attending Surgeon Review						
Reviewer	PGT Estimate	H1 meets definition?	H2 meets definition?	AI meets definition?	Which is AI?	Which is most accurate?
1	70	Yes	Yes	Yes	H2	H2
2	90	Yes	Yes	Yes	AI	AI
3	60	No	No	No	H2	H2

© 2021 Howell RS et al. *JAMA Network Open.*

Wound EMR Information								
**Sequential Number**	**Gender**	**Age**	**Wound Type**	**Wound Location**	**Wound Length (cm)**	**Wound Width (cm)**	**Wound Depth (cm)**	**Wound Area (cm^2)**
97	F	72	Insect bite	Rght lateral leg				

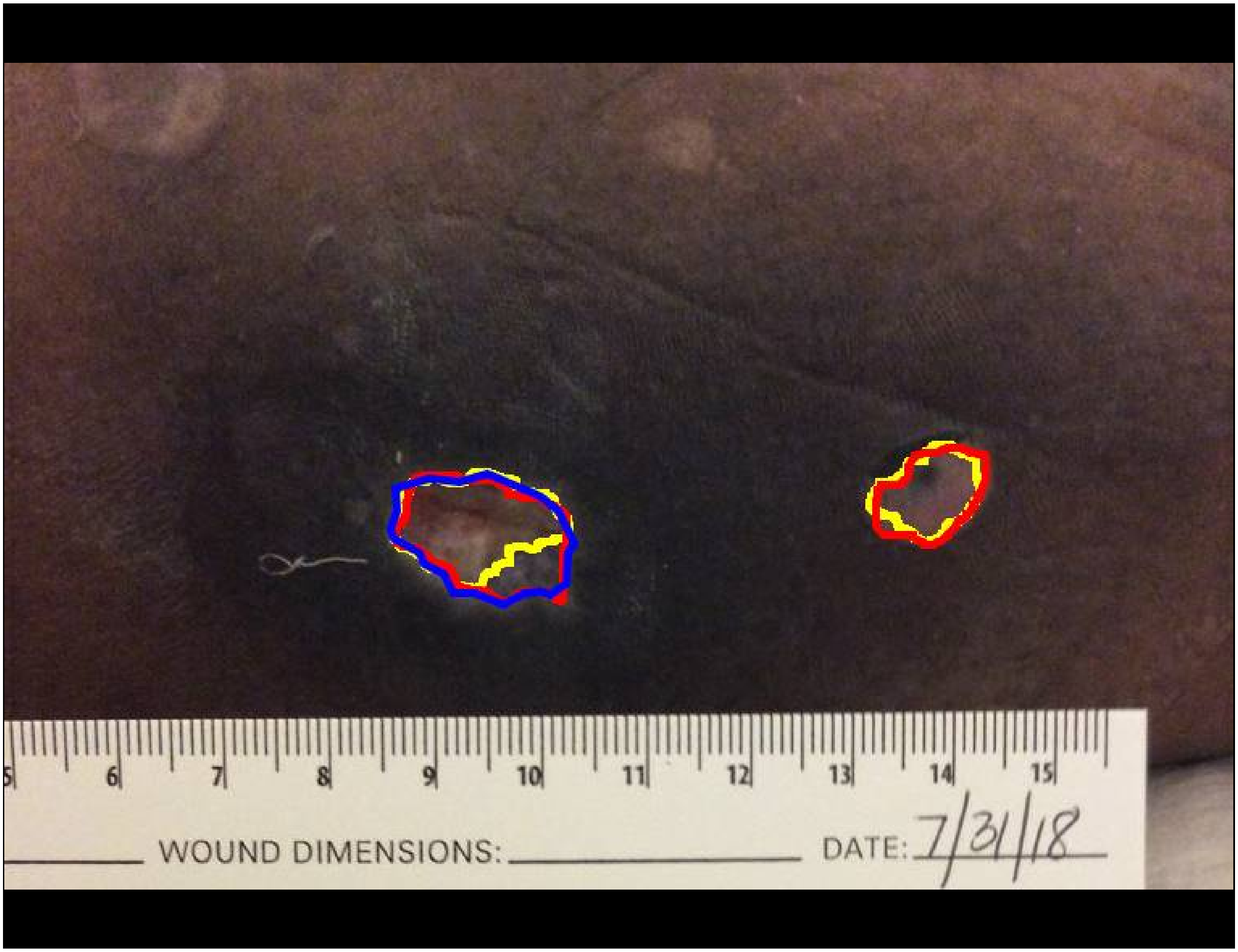

Tracing Data			
**Tracer:**	**Wound Area (px^2)**	**Ruler Calibration (px/cm)**	**Wound Area (cm^2)**
**H1**	7400	65.8	1.71
**H2**	8730	68.0	1.89
**AI**	6442	65.5	1.50

Tracing Comparisons				
**Difference Metric:**	**Human-Human**		**Human-AI**	
	**H1(ref) H2(test)**	**H2(ref) H1(test)**	**H1(ref) AI(test)**	**H2(ref) AI(test)**
**False Negative Area (%)**	8.0	22.0	36.3	34.2
**False Positive Area (%)**	26.0	6.8	23.4	7.9
**|Relative Error (%)|**	18.0	15.2	12.9	26.2

Blinded Attending Surgeon Review						
**Reviewer**	**PGT Estimate**	**H1 meets definition?**	**H2 meets definition?**	**AI meets definition?**	**Which is AI?**	**Which is most accurate?**
**1**	100	No	No	Yes	H1	AI
**2**	50	Yes	Yes	Yes	H1	H1
**3**	100	No	No	No	AI	AI

© 2021 Howell RS et al. *JAMA Network Open.*

Wound EMR Information								
Sequential Number	Gender	Age	Wound Type	Wound Location	Wound Length (cm)	Wound Width (cm)	Wound Depth (cm)	Wound Area (cm^2)
98	F	73	Surgical	Left elbow				

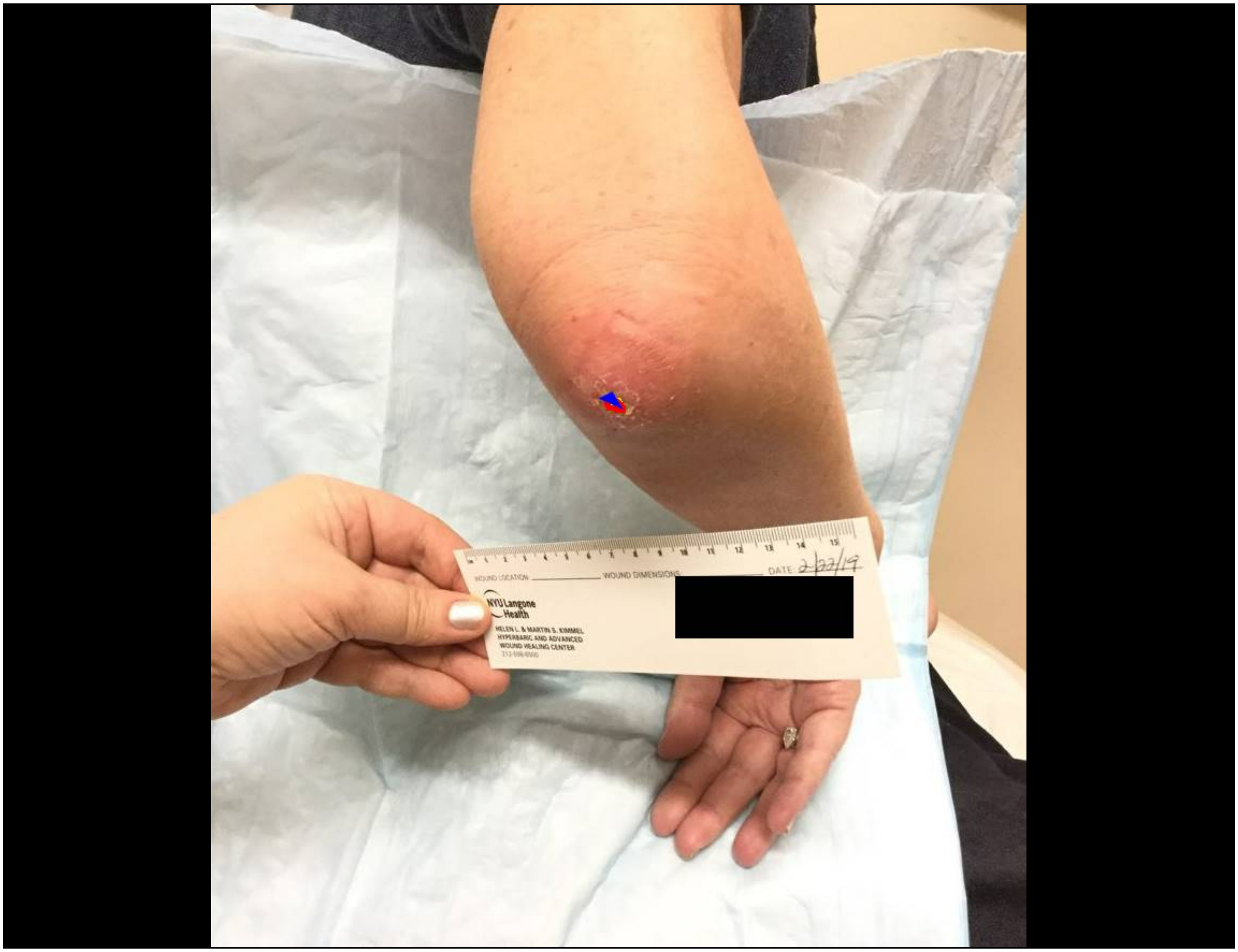

Tracing Data			
Tracer:	Wound Area (px^2)	Ruler Calibration (px/cm)	Wound Area (cm^2)
H1	45	22.6	0.09
H2	94	23.3	0.17
AI	28	16.7	0.10

Tracing Comparisons				
Difference Metric:	Human-Human		Human-AI	
	H1(ref) H2(test)	H2(ref) H1(test)	H1(ref) AI(test)	H2(ref) AI(test)
False Negative Area (%)	6.7	55.3	80.0	86.2
False Positive Area (%)	115.6	3.2	42.2	16.0
|Relative Error (%)|	108.9	52.1	37.8	70.2

Blinded Attending Surgeon Review						
Reviewer	PGT Estimate	H1 meets definition?	H2 meets definition?	AI meets definition?	Which is AI?	Which is most accurate?
1	0	Yes	Yes	Yes	H2	H2
2	50	Yes	Yes	Yes	AI	H1
3	0	Yes	Yes	Yes	H2	H2

© 2021 Howell RS et al. *JAMA Network Open.*

Wound EMR Information								
Sequential Number	Gender	Age	Wound Type	Wound Location	Wound Length (cm)	Wound Width (cm)	Wound Depth (cm)	Wound Area (cm^2)
99	M	54	Traumatic	Left lower leg	10.0	15.0		150.00

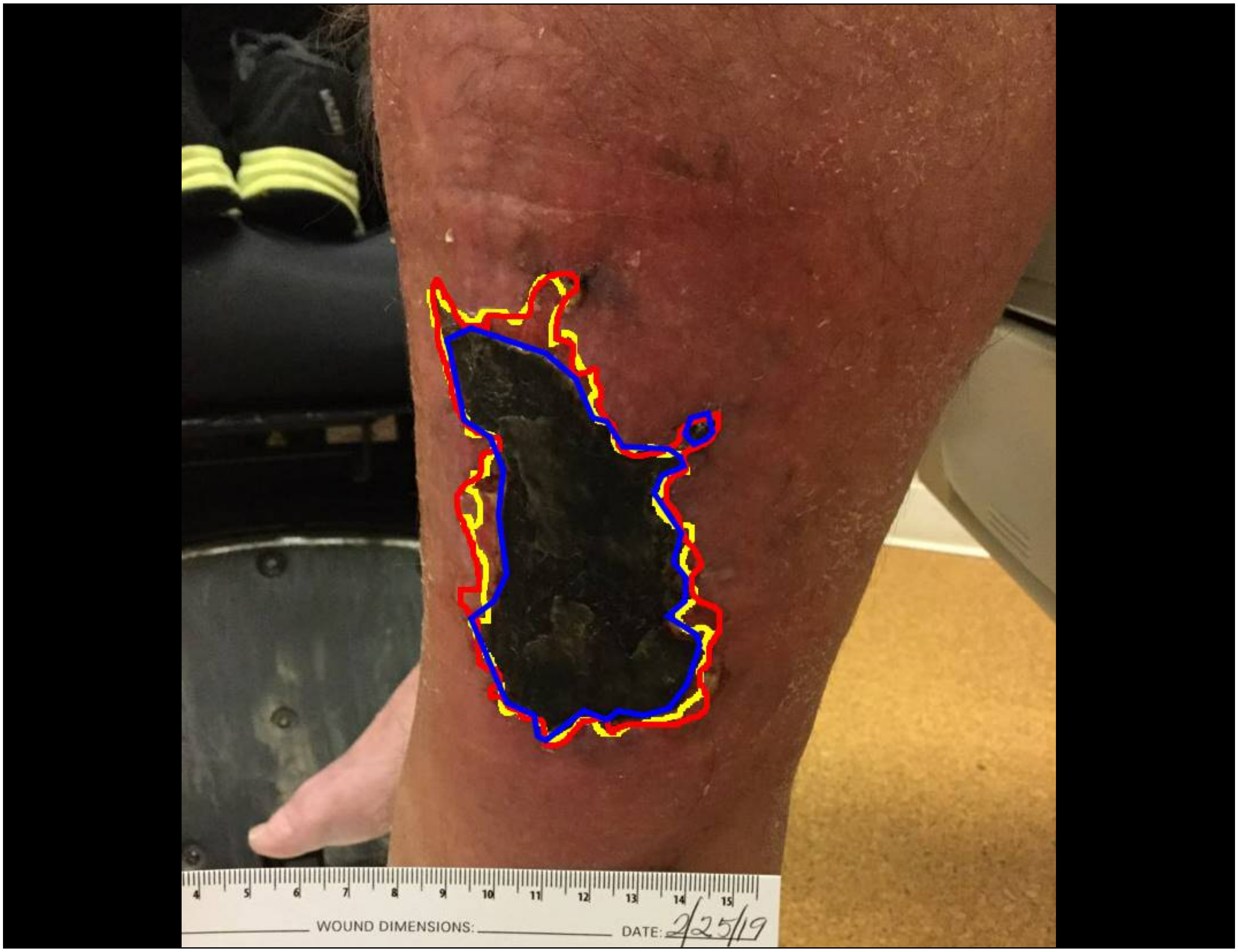

Tracing Data			
Tracer:	Wound Area (px^2)	Ruler Calibration (px/cm)	Wound Area (cm^2)
H1	55915	41.8	31.94
H2	60276	42.3	33.63
AI	47221	41.1	28.00

Tracing Comparisons				
Difference Metric:	Human-Human		Human-AI	
	H1(ref) H2(test)	H2(ref) H1(test)	H1(ref) AI(test)	H2(ref) AI(test)
False Negative Area (%)	2.3	9.4	17.3	22.5
False Positive Area (%)	10.1	2.1	1.8	0.8
|Relative Error (%)|	7.8	7.2	15.5	21.7

Blinded Attending Surgeon Review						
Reviewer	PGT Estimate	H1 meets definition?	H2 meets definition?	AI meets definition?	Which is AI?	Which is most accurate?
1	10	Yes	Yes	No	H1	H1
2	0	Yes	Yes	Yes	H2	AI
3	10	No	Yes	No	H1	H1

© 2021 Howell RS et al. *JAMA Network Open.*

Wound EMR Information								
Sequential Number	Gender	Age	Wound Type	Wound Location	Wound Length (cm)	Wound Width (cm)	Wound Depth (cm)	Wound Area (cm^2)
100	F	62	Diabetic	Left 4th/5th Toe				

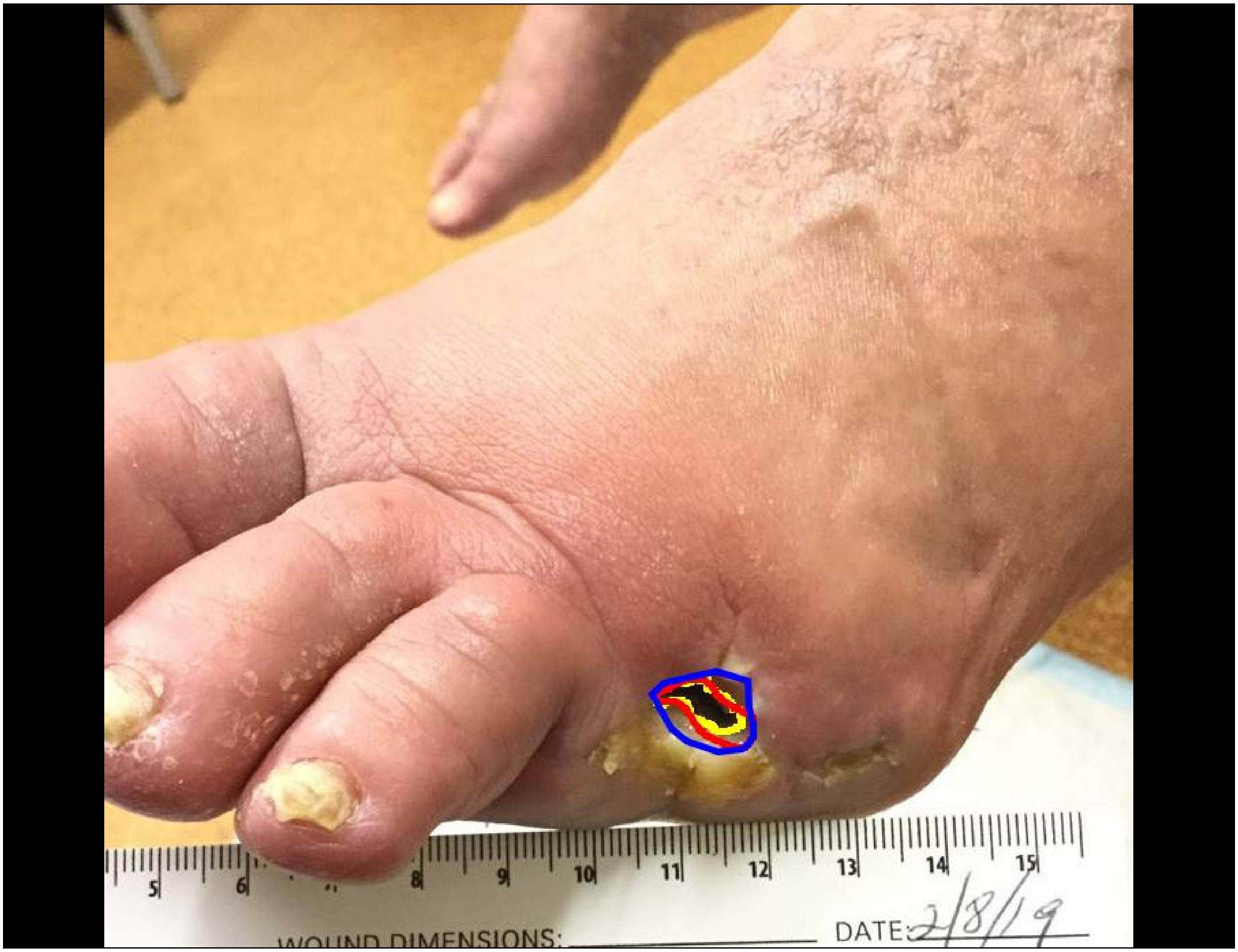

Tracing Data			
Tracer:	Wound Area (px^2)	Ruler Calibration (px/cm)	Wound Area (cm^2)
H1	1221	64.4	0.29
H2	1754	64.0	0.43
AI	3197	63.2	0.80

Tracing Comparisons				
Difference Metric:	Human-Human		Human-AI	
	H1(ref) H2(test)	H2(ref) H1(test)	H1(ref) AI(test)	H2(ref) AI(test)
False Negative Area (%)	5.9	34.5	0.0	0.0
False Positive Area (%)	49.5	4.1	161.8	82.3
|Relative Error (%)|	43.7	30.4	161.8	82.3

Blinded Attending Surgeon Review						
Reviewer	PGT Estimate	H1 meets definition?	H2 meets definition?	AI meets definition?	Which is AI?	Which is most accurate?
1	0	Yes	Yes	No	H2	H1
2	50	Yes	Yes	Yes	AI	H1
3	100	Yes	No	No	H1	H2

© 2021 Howell RS et al. *JAMA Network Open.*

Wound EMR Information								
Sequential Number	Gender	Age	Wound Type	Wound Location	Wound Length (cm)	Wound Width (cm)	Wound Depth (cm)	Wound Area (cm^2)
101	F	17	Pressure	Left ischium	3.0	2.0		6.00

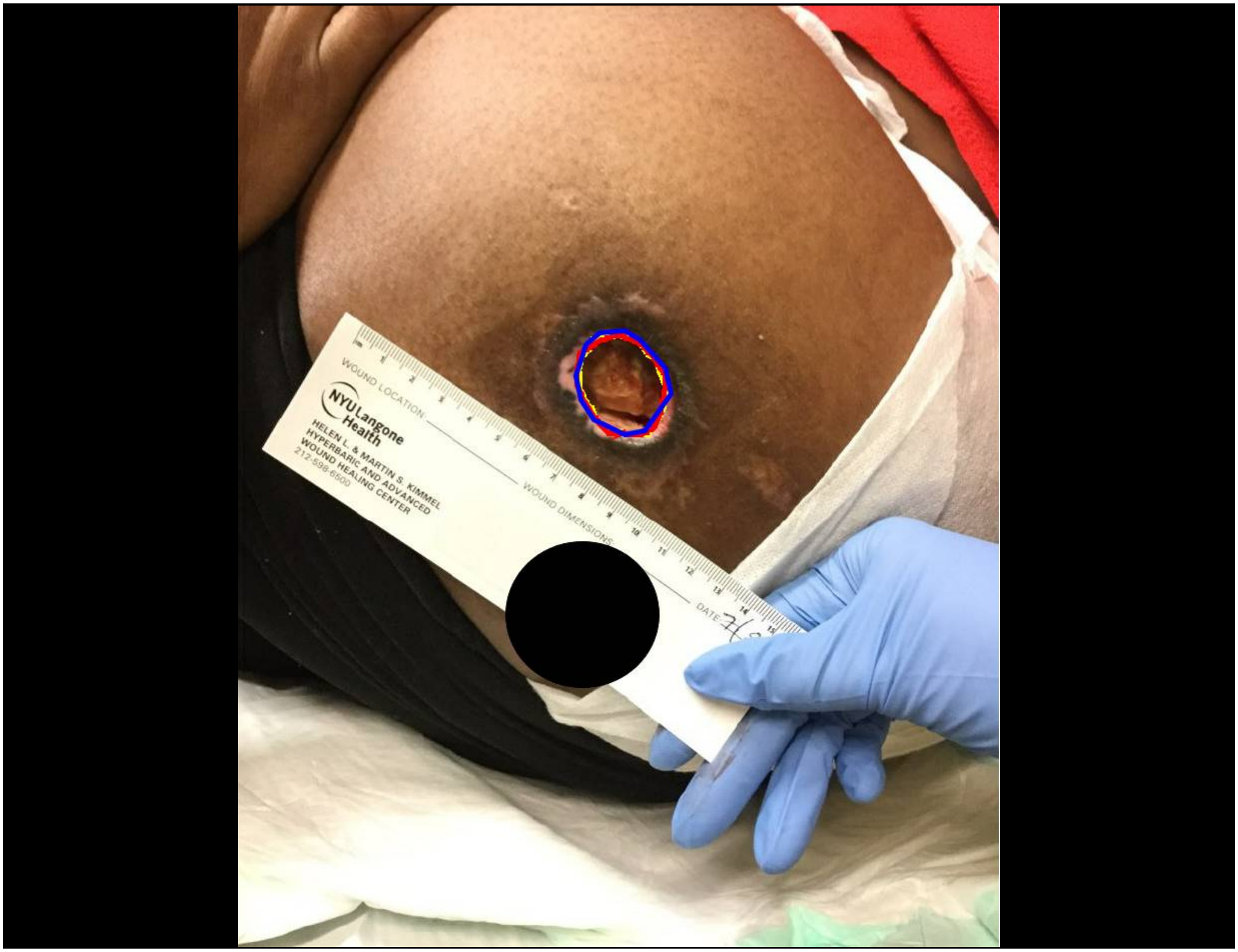

Tracing Data			
Tracer:	Wound Area (px^2)	Ruler Calibration (px/cm)	Wound Area (cm^2)
H1	6607	33.8	5.79
H2	6751	34.9	5.53
AI	7096	34.7	5.90

Tracing Comparisons				
Difference Metric:	Human-Human		Human-AI	
	H1(ref) H2(test)	H2(ref) H1(test)	H1(ref) AI(test)	H2(ref) AI(test)
False Negative Area (%)	2.1	4.2	2.1	3.7
False Positive Area (%)	4.3	2.1	9.5	8.8
|Relative Error (%)|	2.2	2.1	7.4	5.1

Blinded Attending Surgeon Review						
Reviewer	PGT Estimate	H1 meets definition?	H2 meets definition?	AI meets definition?	Which is AI?	Which is most accurate?
1	20	Yes	Yes	Yes	H2	AI
2	90	Yes	Yes	Yes	H2	AI
3	50	Yes	Yes	Yes	AI	H2

© 2021 Howell RS et al. *JAMA Network Open.*

Wound EMR Information								
Sequential Number	Gender	Age	Wound Type	Wound Location	Wound Length (cm)	Wound Width (cm)	Wound Depth (cm)	Wound Area (cm^2)
102	M	55	Surgical	Left knee				

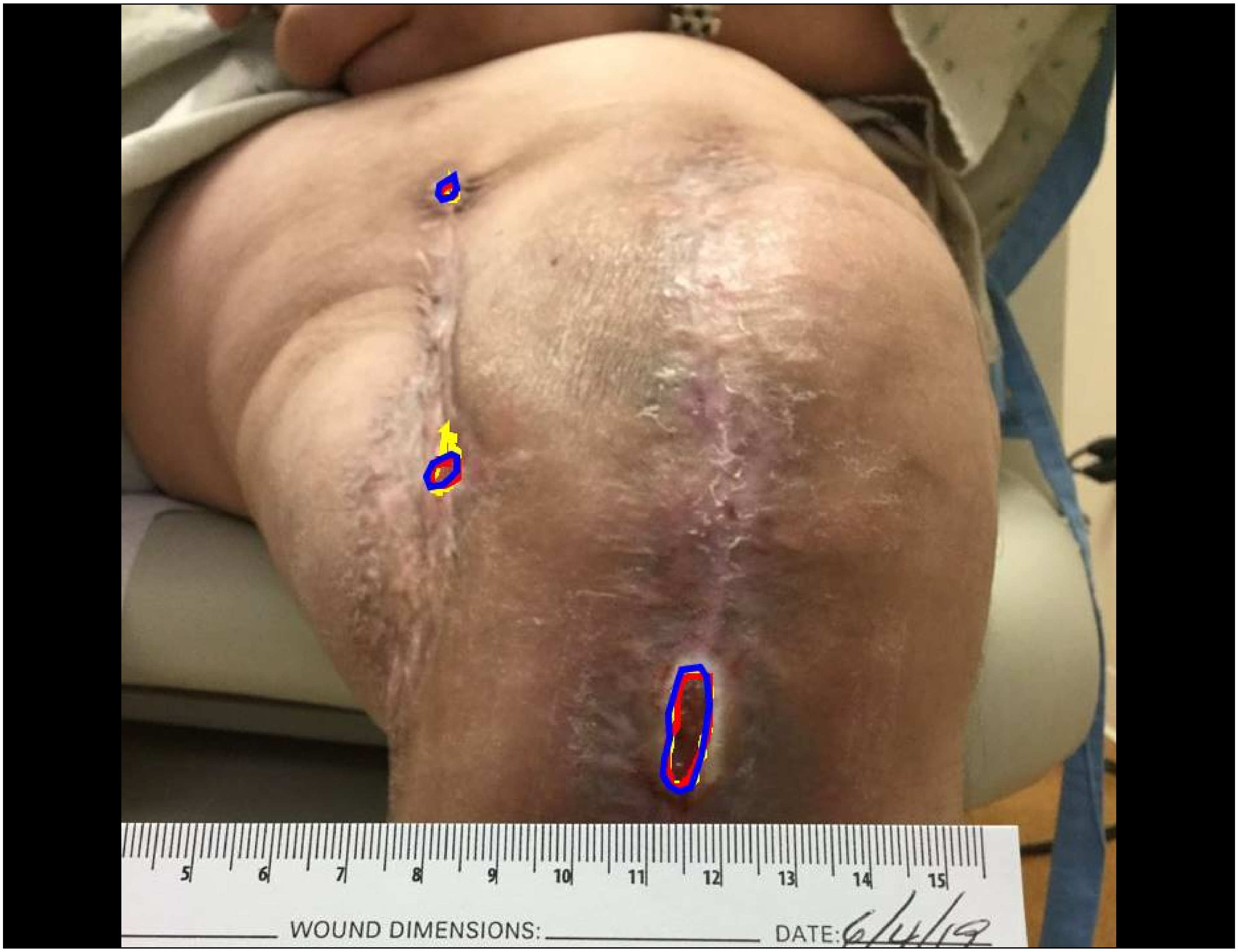

Tracing Data			
Tracer:	Wound Area (px^2)	Ruler Calibration (px/cm)	Wound Area (cm^2)
H1	2554	56.0	0.81
H2	2285	55.0	0.76
AI	2801	55.8	0.90

Tracing Comparisons				
Difference Metric:	Human-Human		Human-AI	
	H1(ref) H2(test)	H2(ref) H1(test)	H1(ref) AI(test)	H2(ref) AI(test)
False Negative Area (%)	17.0	7.2	9.1	4.4
False Positive Area (%)	6.5	19.0	18.8	27.0
|Relative Error (%)|	10.5	11.8	9.7	22.6

Blinded Attending Surgeon Review						
Reviewer	PGT Estimate	H1 meets definition?	H2 meets definition?	AI meets definition?	Which is AI?	Which is most accurate?
1	90	Yes	Yes	Yes	H1	H2
2	100	Yes	Yes	Yes	H1	H2
3	90	Yes	No	No	H2	H2

© 2021 Howell RS et al. *JAMA Network Open.*

Wound EMR Information								
Sequential Number	Gender	Age	Wound Type	Wound Location	Wound Length (cm)	Wound Width (cm)	Wound Depth (cm)	Wound Area (cm^2)
103	F	68	Unknown	Lateral to labia				

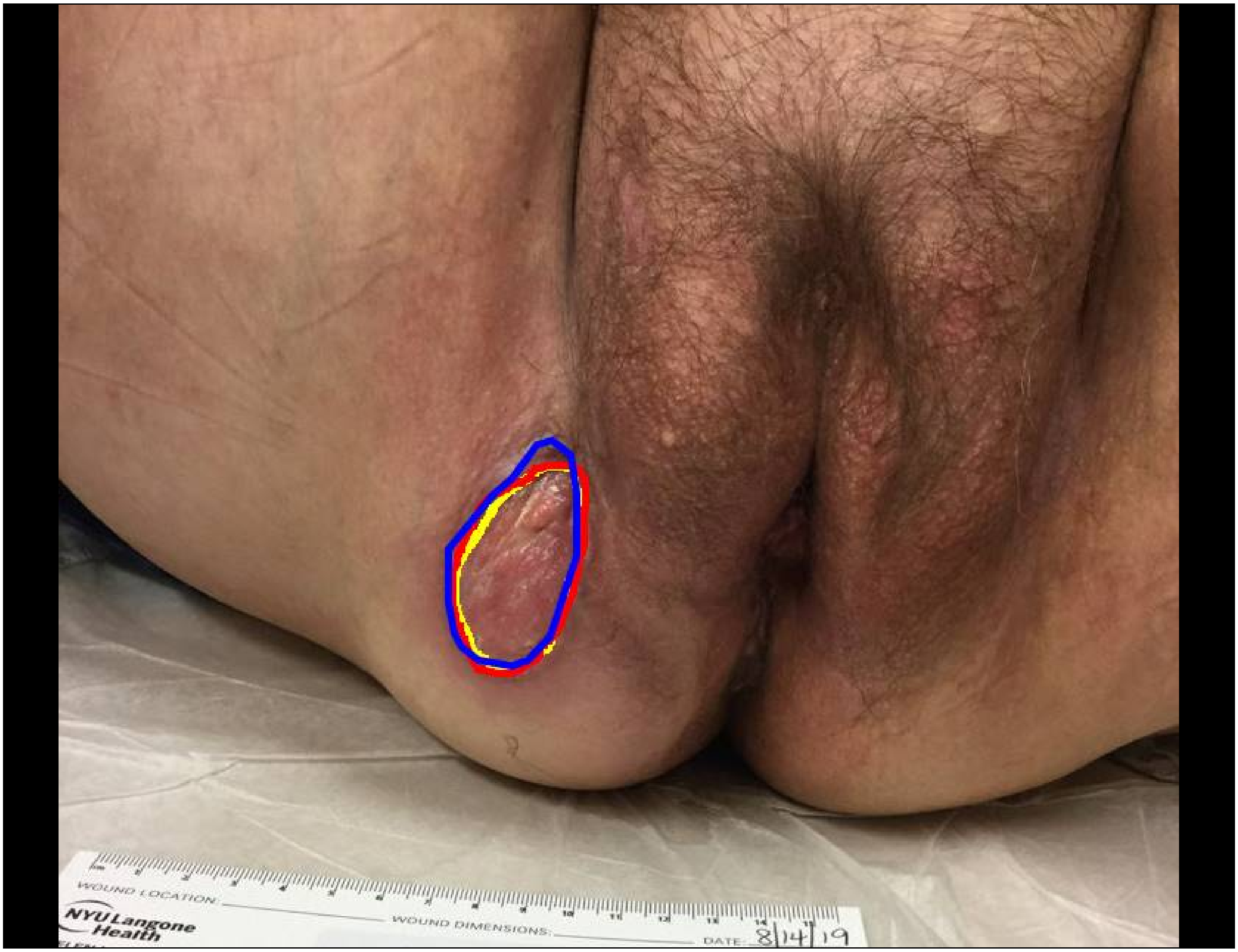

Tracing Data			
Tracer:	Wound Area (px^2)	Ruler Calibration (px/cm)	Wound Area (cm^2)
H1	8174	32.0	7.99
H2	9294	32.4	8.83
AI	9455	31.5	9.50

Tracing Comparisons				
Difference Metric:	Human-Human		Human-AI	
	H1(ref) H2(test)	H2(ref) H1(test)	H1(ref) AI(test)	H2(ref) AI(test)
False Negative Area (%)	0.4	12.4	1.5	7.4
False Positive Area (%)	14.1	0.3	17.2	9.1
|Relative Error (%)|	13.7	12.1	15.7	1.7

Blinded Attending Surgeon Review						
Reviewer	PGT Estimate	H1 meets definition?	H2 meets definition?	AI meets definition?	Which is AI?	Which is most accurate?
**1**	90	No	No	Yes	H2	H1
**2**	100	Yes	Yes	Yes	H1	AI
**3**	90	Yes	Yes	No	AI	H2

© 2021 Howell RS et al. *JAMA Network Open.*

Wound EMR Information								
Sequential Number	Gender	Age	Wound Type	Wound Location	Wound Length (cm)	Wound Width (cm)	Wound Depth (cm)	Wound Area (cm^2)
104	F	54	Surgical	Abdomen	4.5	3.0	4.5	13.50

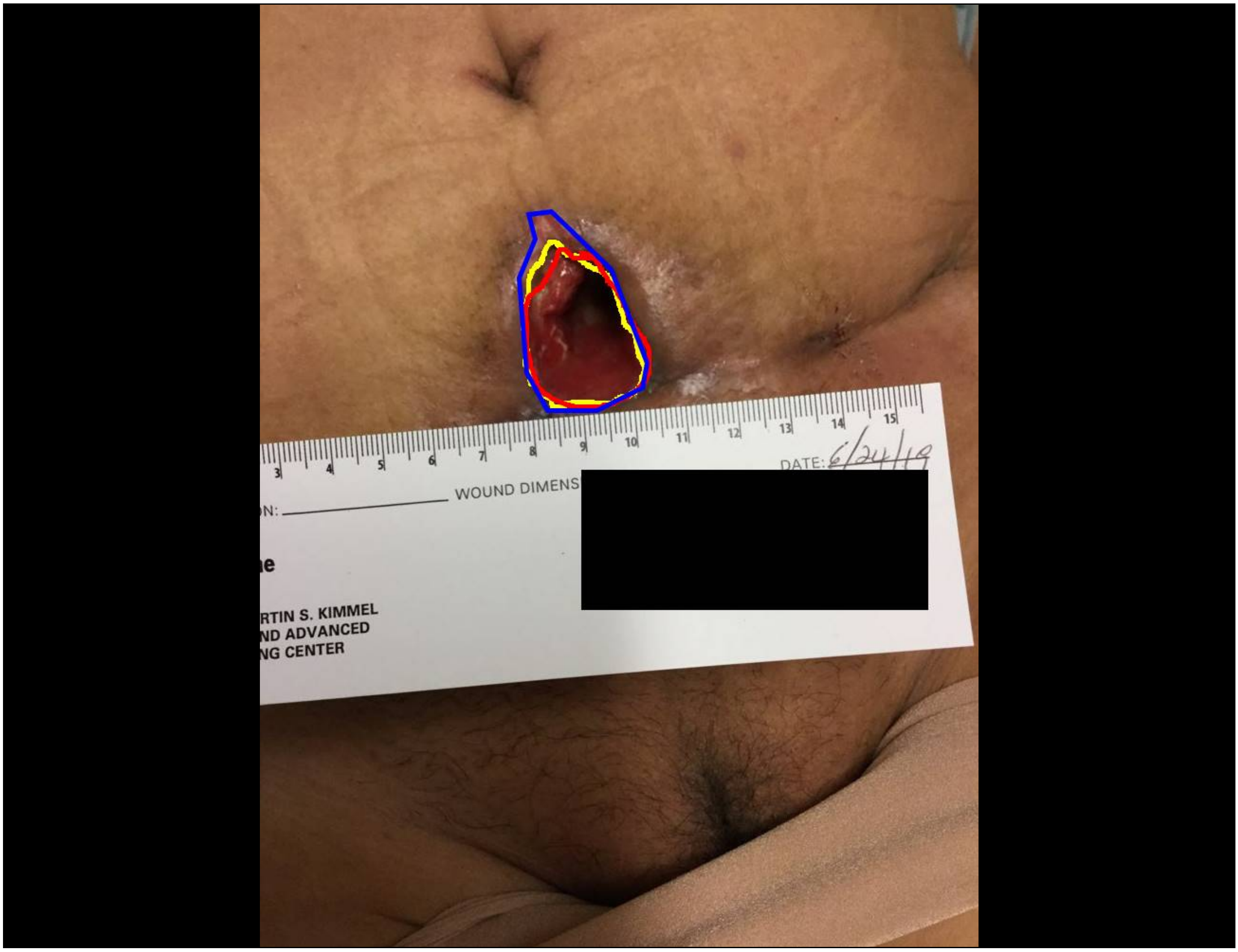

Tracing Data			
Tracer:	Wound Area (px^2)	Ruler Calibration (px/cm)	Wound Area (cm^2)
H1	15619	53.7	5.41
H2	15495	55.4	5.04
AI	19538	56.1	6.20

Tracing Comparisons				
Difference Metric:	Human-Human		Human-AI	
	H1(ref) H2(test)	H2(ref) H1(test)	H1(ref) AI(test)	H2(ref) AI(test)
False Negative Area (%)	7.6	6.9	0.1	1.5
False Positive Area (%)	6.8	7.7	25.2	27.6
|Relative Error (%)|	0.8	0.8	25.1	26.1

Blinded Attending Surgeon Review						
Reviewer	PGT Estimate	H1 meets definition?	H2 meets definition?	AI meets definition?	Which is AI?	Which is most accurate?
1	60	No	No	Yes	H2	H1
2	100	Yes	Yes	Yes	H2	AI
3	90	Yes	No	No	H1	H2

© 2021 Howell RS et al. *JAMA Network Open.*

eAppendix 3. Supplementary Discussion

Analysis of tracing discrepancy subgroups

Visual inspection of the traces revealed that different types of discrepancies can be observed which can be classified into several subgroups:

Wounds annotated with slightly different borders:

In these cases, the human annotators and algorithm have closely overlapping wound boundaries with relatively small error measure values across annotators. Some examples include Site 1 wounds 9, 20, 21, 24, and 27. The bulk of wound photos qualitatively appear to be of this category.

Wounds annotated with highly different borders or with different included regions/satellite areas:

In these cases, there is a distinct region or there are multiple regions of the wound where there is disagreement between human annotators and/or the algorithm whether the region met the definition of wound area (i.e. discrepancies are due to disagreeing whether a patch of a certain color is epithelialized or which contour constitutes the wound boundary rather than being due to hasty/sloppy tracings). Some examples include Site 1, photo numbers 8, 10, 11, 13, and 29. In some cases, satellite wound areas are recognized differently across annotators (for example Site 1 photo numbers 38 and 91). The standard definition of wound area for this study used a distance cutoff for inclusion satellite wounds, but this may be difficult to assess consistently for satellite areas near the cutoff point. For wounds with several open areas of similar size, it may be difficult for annotators to determine the main wound for tracing.

Disagreements regarding complete healing or presence of a wound:

In these cases, there is a lack of consensus on the presence of an open wound (a subset of annotators made a tracing). This appears to happen for example in cases where annotators disagree whether a visibly contrasted region is epithelialized or not, or whether there is adequate contrast in a region to indicate presence of an open wound or wound edge (for example Site 1 photo numbers 17, 28, 34, 43, 47, and 74).

© 2021 Howell RS et al. *JAMA Network Open.*
